# Regioselective intermolecular carboamination of allylamines *via* nucleopalladation: empowering three-component synthesis of vicinal diamines†

**DOI:** 10.1039/d4sc07630c

**Published:** 2024-11-29

**Authors:** Shib Nath Saha, Nityananda Ballav, Suman Ghosh, Mahiuddin Baidya

**Affiliations:** a Department of Chemistry, Indian Institute of Technology Madras Chennai 600036 India mbaidya@iitm.ac.in

## Abstract

An intermolecular carboamination reaction of allyl amines under Pd(ii)-catalysis is reported, expediting the synthesis of valuable vicinal diamines embedded in a functionally enriched linear carbon framework with high yields and exclusive Markovnikov selectivity. Central to our approach is the strategic use of a removable picolinamide auxiliary, which directs the regioselectivity during aminopalladation and stabilizes the crucial 5,5-palladacycle intermediate. This stabilization facilitates oxidative addition to carbon electrophiles, enabling the simultaneous incorporation of diverse aryl/styryl groups as well as important amine motifs, such as sulfoximines and anilines, across carbon–carbon double bonds. The protocol features broad substrate compatibility, tolerance to various functional groups, and scalability. The utility of this method is further demonstrated by the site-selective diversification of pharmaceutical agents. Additionally, these products serve as versatile intermediates for synthesizing heterocycles and function as effective ligands in catalytic transfer hydrogenation reactions. Notably, this work represents a rare instance of nucleopalladation-guided intermolecular carboamination of allylamines.

## Abstract 2

Intermolecular carboamination of allyl amines under Pd(ii)-catalysis is reported, expediting the synthesis of vicinal diamines embedded in a functionally enriched linear carbon framework with high yields and exclusive Markovnikov selectivity.

## Graphical abstract

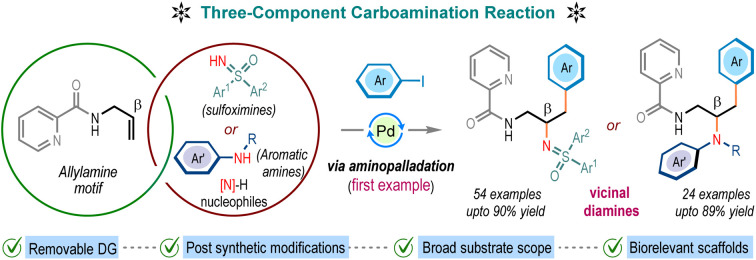

## Introduction

**Fig. 1:**
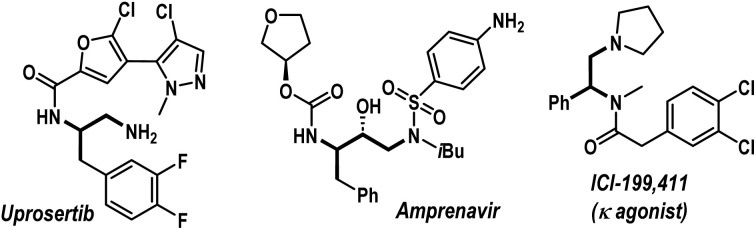
Representative pharmaceuticals with 1,*n*-diamine motifs.

**Scheme 1:**
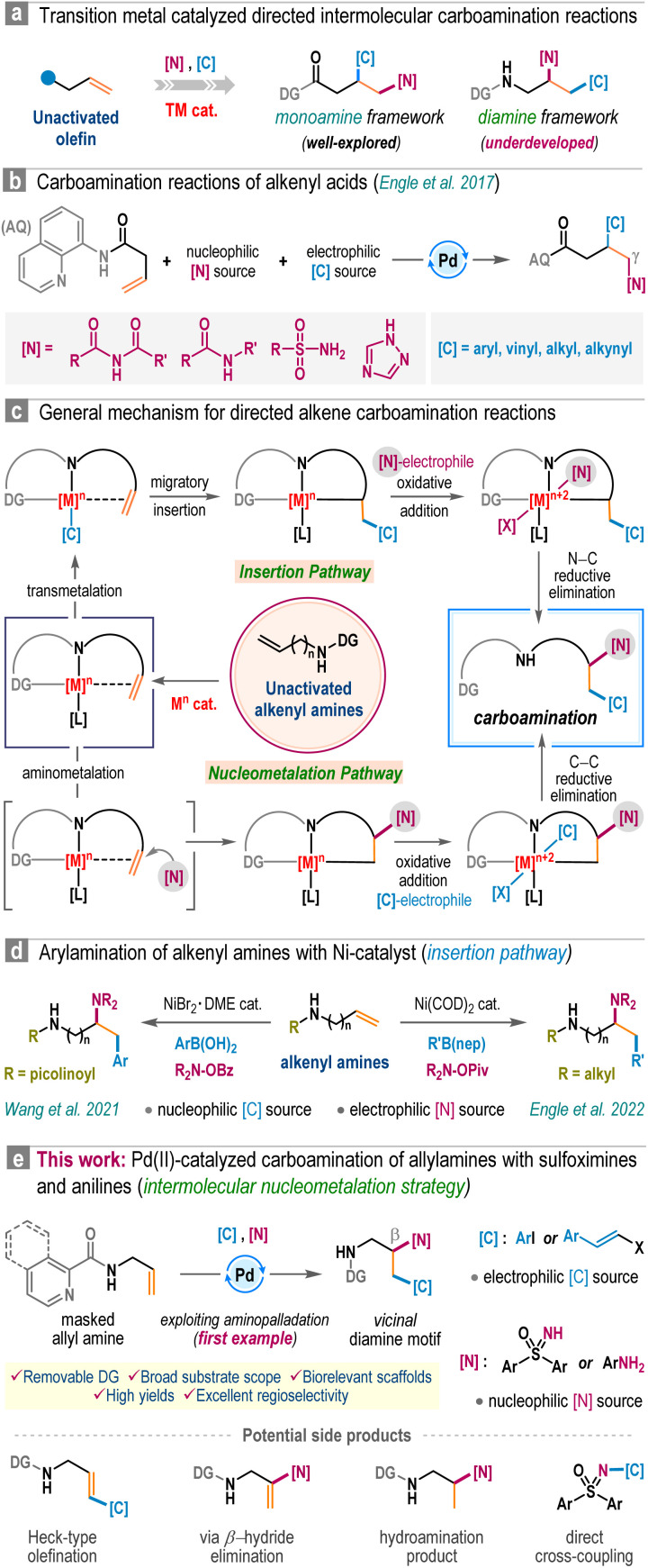
Catalytic intermolecular carboamination of alkenyl amines.

Amines, especially aliphatic congeners with multiple amine functionalities in a linear carbon framework, hold great significance in pharmaceutical science, organic synthesis, and material chemistry.^1,2^ They represent a wide range of valuable natural products and biologically active molecules, including top-selling small-molecule drugs (Fig. 1).^2^ Consequently, developing efficient catalytic techniques for constructing functionally enriched aliphatic amines, particularly vicinal diamines, is a continuous endeavor within the synthetic chemistry community. In this regime, we envisioned that transition-metal-catalyzed three-component carboamination reactions of unactivated olefins would be highly rewarding, potentially enabling the installation of both C–N and C–C bonds onto the olefin motif in a single operation (Scheme 1a).^3,4^ Particularly, the pioneering work from the Engle group unveiling the carboamination reaction of 8-aminoquinoline embedded 3-butenoic amides is a landmark contribution, showcasing the transformative potential of this approach (Scheme 1b).^3*f*^ Surprisingly, such an intermolecular carboamination blueprint with allylamines remains largely unexplored, although it can potentially constitute a powerful platform for accessing high-value vicinal diamine frameworks from inexpensive building blocks (Scheme 1a).^1*a*,*b*,5^ Mechanistically, transition-metal-catalyzed carboamination of alkenyl amines can be initiated either through the insertion mechanism or *via* the aminometalation pathway (Scheme 1c).^6^ In 2021, Wang *et al.* reported 1,2-arylamination of various alkenyl amine derivatives with aryl boronic acids and N–O electrophiles under nickel catalysis (Scheme 1d, left).^7^ Engle *et al.* also delineated a sensitive Ni(COD)_2_ catalyst for the carboamination reaction, engaging alkenyl amines, aryl/alkenyl boronic esters, and N–O reagents (Scheme 1d, right).^8^ To the best of our knowledge, these are few reports addressing the carboamination reactions of alkenyl amines, and these methodologies are based on an insertion pathway. Currently, the conceptually distinct aminometalation strategy, which capitalizes on Wacker reaction modality to enable intriguing olefin functionalization events, remains elusive for this purpose. Apparently, the materialization of the intermolecular aminometalation/carboamination reaction of allylamines is increasingly challenging as the anticipated reaction may prematurely terminate, leading to Heck-type olefination, *N*-alkenylation, or hydroamination products (Scheme 1e, below).^9^ Further, the protocol must overcome the underside direct cross-coupling reaction between carbon electrophiles and nitrogen nucleophiles, a renowned C–N bond-forming methodology under palladium catalysis.^10^

With our continuous interest in the nucleopalladation strategy,^11^ herein, we report the first example of aminopalladation-guided intermolecular three-component carboamination of allylamines (Scheme 1e). By employing removable directing groups in the presence of the Pd(OAc)_2_ catalyst, a variety of sulfoximines as nucleophilic aminating agents and aryl iodides as well as styryl iodides as carbon-electrophiles were regioselectively installed across the carbon–carbon double bond in allyl amines, furnishing a spectrum of densely sp^3^-functionalized vicinal diamines in high yields. The protocol is also operational with aromatic amines and remains effective in the presence of various medicinally relevant motifs, highlighting its synthetic versatility. Notably, our findings on the carboamination of allylamines complement those of the Engle group,^3*f*^ though we observed a distinct site-selectivity. In our study, nucleopalladation initiates at the β-center of allylamine, in contrast to Engle's work, where the nucleophilic attack occurs at the γ-position of the 3-butenoic amide.

## Results and discussion

Our investigations began following the three-component coupling reaction of allylamine 1a bearing the picolinamide (PA) directing group with diphenyl sulfoximine 2a as an *N*-nucleophile and phenyl iodide 3a as a carbon-electrophile (Table 1). This choice is beneficial, as the picolinamide directing group, initially introduced by Daugulis *et al.*, has a rich history in diverse metal-catalyzed coupling processes,^12^ and sulfoximines, the chemically and metabolically stable monoaza S(vi)-congeners of sulfones, are emerging scaffolds in the pharmaceutical and crop protection industries.^13^ Meanwhile, sulfoximine nucleophiles have never been considered in the nucleometalation strategy. Satisfyingly, exposure of 1a, 2a, and 3a to the Pd(OAc)_2_ catalyst (10 mol%) and K_2_CO_3_ base (1.0 equiv.) in 1,2-dichloroethane (DCE) solvent at 100 °C could facilitate the carboamination reaction, furnishing vicinal diamine 4a in a moderate yield of 67% (entry 1). Encouraged by this result, we have screened different aprotic and protic solvents; however, except acetonitrile, where 4a was obtained in 45% yield, this carboamination reaction was unsuccessful in most of the cases (entries 2 and 3). Examination of different bases such as Na_2_CO_3_, NaOAc, and K_3_PO_4_ revealed only poor activity with the Na_2_CO_3_ base giving the desired product in 19% yield (entry 4). When the loading of the K_2_CO_3_ base was increased to 1.5 equiv., the reaction yield improved significantly, furnishing 4a in 84% isolated yield (entry 5). However, a further increase in the K_2_CO_3_ base had a detrimental outcome (entry 6). Similarly, the consideration of a higher loading of sulfoximine 2a (2.0 equiv.) or lowering the amount of aryl iodide 3a (2.0 equiv.) reduced the reaction yield (entry 7).

**Table 1:** Optimization of reaction conditions^a^

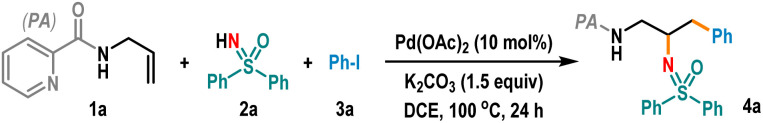		
Entry	Deviation from standard conditions	Yield of 4a^b^ (%)
1	K_2_CO_3_ (1.0 equiv.)	67
2	In 1,4-dioxane/DMA/CH_3_CN with K_2_CO_3_ (1.0 equiv.)	—/—/45
3	In MeOH/^*t*^BuOH/HFIP with K_2_CO_3_ (1.0 equiv.)	—/—/—
4	With Na_2_CO_3_/NaOAc/K_3_PO_4_ (1.0 equiv.)	19/—/—
5	None	84
6	K_2_CO_3_ (2.0 equiv.)	65
7	With 2a (2.0 equiv.)/3a (2.0 equiv.)	60/48
8	80 °C/120 °C	58/71
9	Without Pd(OAc)_2_ catalyst/K_2_CO_3_ base	—/—
10	Ni(OAc)_2_/Co(OAc)_2_·4H_2_O instead of Pd(OAc)_2_	—/—
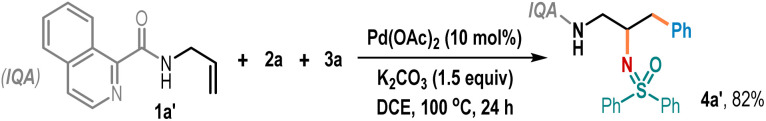		

a Reaction conditions: 1a (0.2 mmol), 2a (0.24 mmol), 3a (4.0 equiv.), K_2_CO_3_ (1.5 equiv.), and solvent (1.0 mL), 24 h.

b Isolated yield.

Also, performing the reaction at a temperature of 80 °C or 120 °C did not improve the outcomes (entry 8). Control experiments revealed that the presence of both the Pd(OAc)_2_ catalyst and the K_2_CO_3_ base was critical for this reaction, and the reaction completely shuts down in the absence of either component (entry 9). Examination of other catalysts, such as Ni(OAc)_2_ and Co(OAc)_2_·4H_2_O, showed them to be unsuitable for promoting this carboamination reaction (entry 10). The efficacy of other directing groups was also examined. For example, the *N*-allyl amide 1a′ derived from isoquinoline-1-carboxylic acid effectively gave the desired product 4a′ in 82% yield (Table 1 below).

**Scheme 2:**
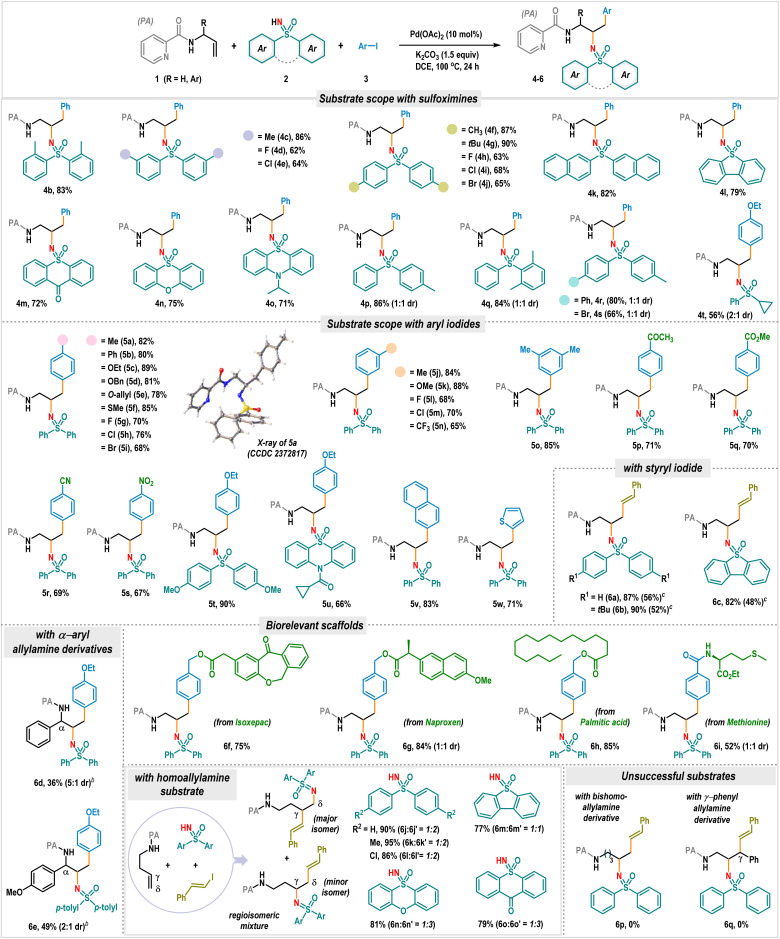
Scope of the three-component carboamination reaction for sulfoximine nucleophiles.^*a a*^Reaction conditions: 1 (0.2 mmol), 2 (0.24 mmol), 3 (4.0 equiv.), K_2_CO_3_ (1.5 equiv.), and DCE (1.0 mL), 24 h. Isolated yields were provided. ^*b*^Reaction was performed with Na_2_CO_3_ (1.5 equiv.). ^*c*^Styryl bromide (4.0 equiv.) was used as the coupling partner.

With the optimized reaction conditions in hand, we then explored the scope of this carboamination reaction (Scheme 2). The protocol is quite general for a wide range of diaryl sulfoximines encompassing electronically diverse substitutions at *ortho*-, *meta*-, and *para*-positions in the arene ring, providing vicinal diamines 4b–4j in good to excellent yields (62–90%). The reaction was fruitful with acyclic dinaphthyl sulfoximine and rigid cyclic dibenzothiophene sulfoximine, giving 4k and 4l in 82% and 79% yields, respectively. Other sulfoximines prepared from medicinally relevant heterocycles such as thioxanthone, phenoxathiin, and phenothiazine also effectively participated in the carboamination reaction, producing 4m–4o in high yields. When diaryl sulfoximines featuring unsymmetrical substitution patterns were employed, the desired products 4p–4s were obtained in high yields with a 1 : 1 mixture of diastereomers (Scheme 2). The reaction was also fruitful with alkyl-substituted sulfoximine, offering the product 4t in 56% yield with dr 2 : 1 (Scheme 2). Next, we turned our attention toward the aryl iodide scope, which also proved to be very general (Scheme 2). Aryl iodides bearing alkyl (5a, 5j, and 5o), aryl (5b), ether (5c–5e, 5k, and 5t–5u), thioether (5f), and trifluoromethyl (5n) motifs smoothly reacted to produce carboamination products in very high to excellent yields. The compound 5a was crystalized, and the single crystal X-ray analysis unambiguously validated the product structure and the regiochemistry. Notably, the presence of halogen functionalities such as fluorine (5g and 5l), chlorine (5h and 5m), and bromine (5i) did not hamper the reaction. Impressively, the protocol tolerates various useful common functional groups, such as ketone (5p), ester (5q), nitrile (5r), and nitro (5s), which are good synthetic handles for post-synthetic modifications. 2-Iodonaphthalene and heteroaryl iodide, such as 2-thienyl iodide, offered 5v and 5w in 83% and 71% yields, respectively. Delightfully, the carboamination reaction can also be effectively carried out with styryl iodide, offering synthetically valuable γ-alkenyl vicinal diamines 6a–6c in excellent yields (Scheme 2). The reaction was also performed using the less reactive styryl bromide. However, products 6a–6c are obtained in moderate yields under the reaction conditions (Scheme 2). The variation in the allylamine framework was also considered. The reactions with α-aryl allyl picolinamides led to the products 6d and 6e in synthetically useful yields, albeit requiring a switch to the Na_2_CO_3_ base (Scheme 2).

To showcase the synthetic versatility further, an array of pharmacophore-coupled aryl iodides were tested under standard reaction conditions, where substrates bearing bio-relevant scaffolds like isoxepac (6f), naproxen (6g), palmitic acid (6h) and methionine (6i) produced functionally enriched vicinal diamines in good to high yields (Scheme 2).

The three-component carboamination reaction was also successfully applied to substrates derived from homoallylic amines. However, we observed the formation of a regioisomeric mixture in these cases, producing carboamination products 6j–6o and 6j′–6o′ in very high yields (77–95%, Scheme 2). The formation of regioisomeric products can be attributed to the competitive generation of 5,5- and 5,6-bispalladacycle intermediates. When the chain length was further extended, the three-component coupling failed (6p, Scheme 2), and the reaction was also unproductive with a γ-phenyl substituted allyl amine substrate (6q, Scheme 2). In these instances, the starting materials were recovered.

**Scheme 3:**
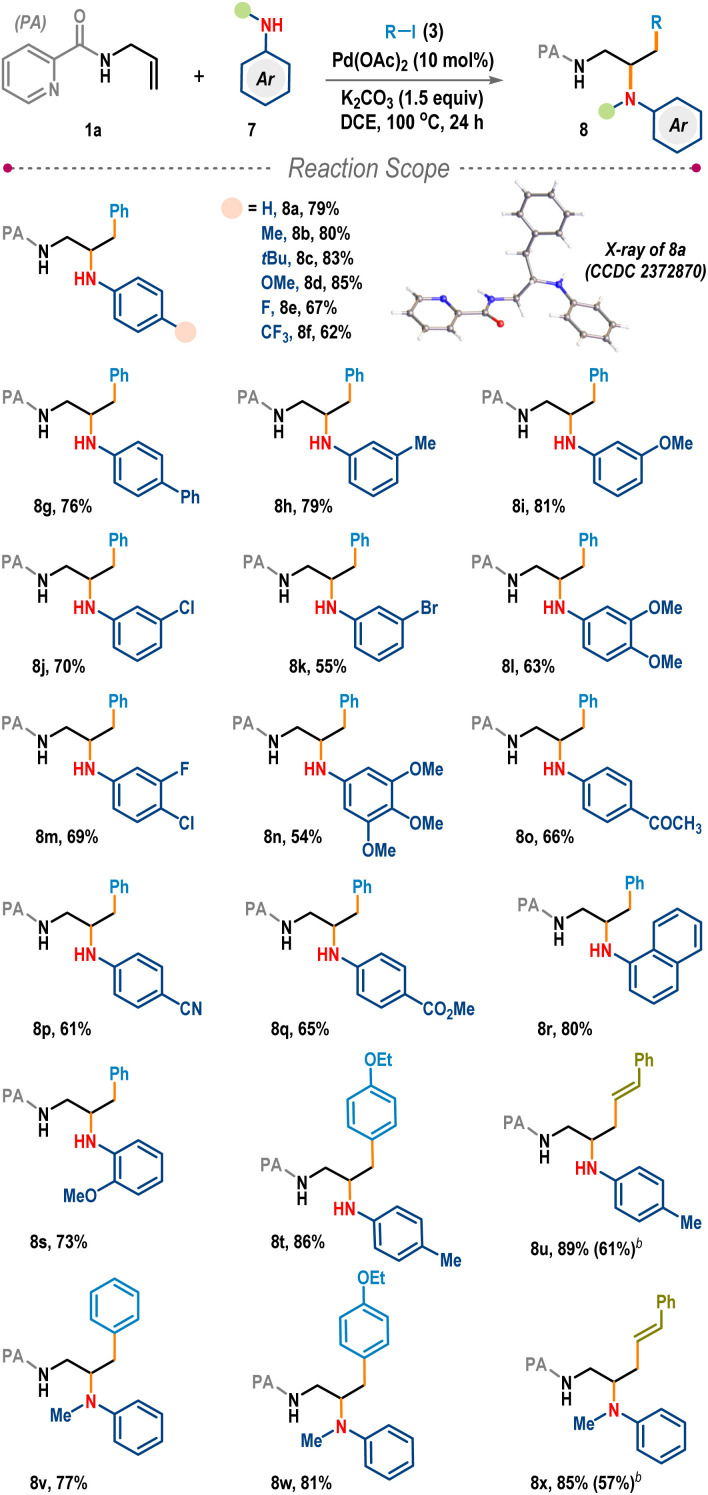
Carboamination reaction of allylamines with anilines.^*a a*^Reaction conditions: 1a (0.2 mmol), 7 (1.2 equiv.), 3 (4.0 equiv.), K_2_CO_3_ (1.5 equiv.), and DCE (1.0 mL), 24 h. Isolated yields were provided. ^*b*^Styryl bromide (4.0 equiv.) was used as the coupling partner.

To advance the reaction generality further, we explored the potential of aromatic amines for this intermolecular carboamination (Scheme 3).^14^ Gratifyingly, under the same reaction conditions, three-component coupling among the allyl amine (1a), phenyl iodide (3a), and aniline (7a) proceeded smoothly to furnish vicinal diamine 8a in 79% yield. The structure of the aniline adduct was also confirmed through single crystal X-ray analysis. Other mono- (8b–8k), di- (8l, 8m), and tri-substituted (8n) anilines with electronically diverse functional groups in the phenyl ring produced desired products in high yields. The carboamination was also fruitful for poorly nucleophilic anilines bearing strongly withdrawing functionalities such as ketone (8o), nitrile (8p), and ester (8q). Bulky anilines, for example, 1-naphthylamine and 2-anisidine, were also suitable, delivering 8r and 8s in 80% and 73% yields, respectively. Reactions with substituted aryl iodide as well as styryl iodide provided 8t and 8u in excellent yields. Interestingly, the three-component carboamination was also productive with *N*-methylaniline, a secondary aromatic amine, and the desired vicinal diamines 8v–8x were isolated in very high yields (77–85%, Scheme 3).

**Scheme 4:**
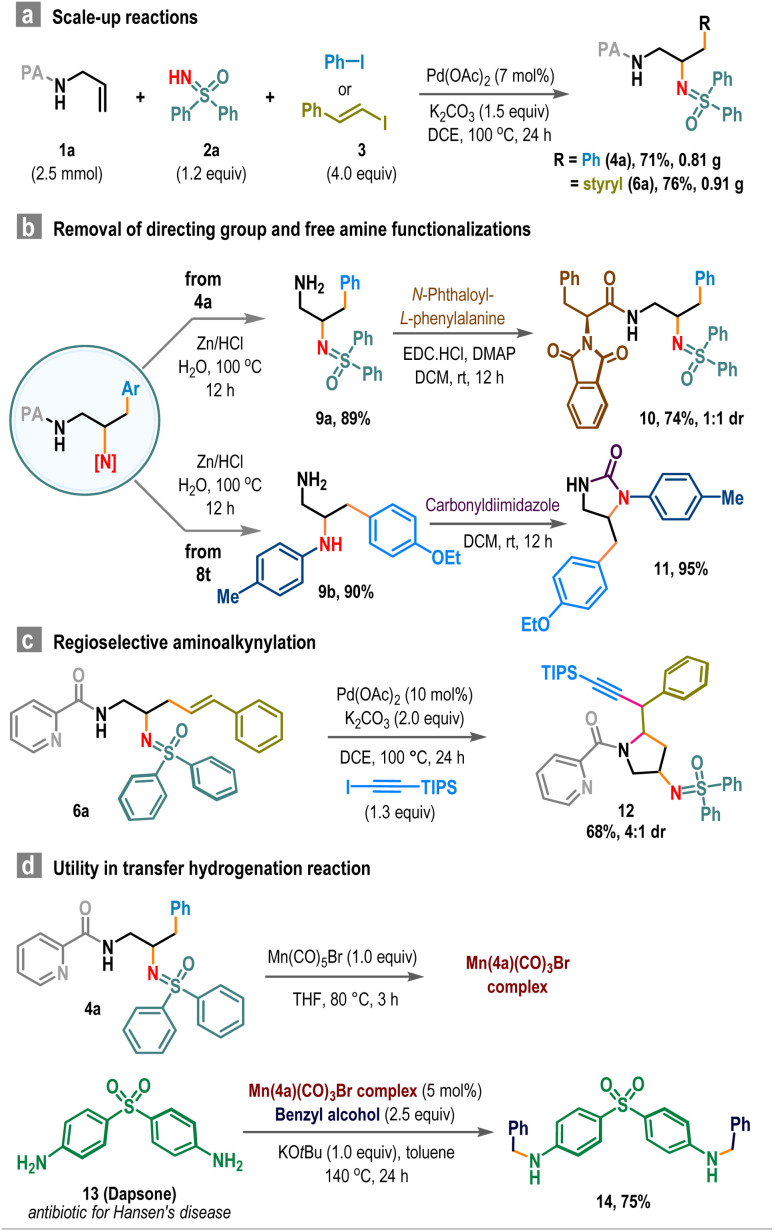
Scale-up reactions and post-synthetic applications.

To demonstrate the synthetic utility, we performed scale-up reactions. Starting from 2.5 mmol scale reactions, compounds 4a and 6a were obtained in 71% and 76% yields, respectively (Scheme 4a). Removal of the picolinamide (PA) directing group enabled access to sp^3^-functionalized primary amine 9, which was subsequently coupled with N-protected phenylalanine to produce sulfoximine-embedded peptide 10 in good yield (Scheme 4b). Similarly, cyclic urea derivative 11 was prepared in two steps from carboamination product 8t in high yield (Scheme 4b, below). Also, the product 6a was employed in an intramolecular amino alkylation reaction to afford trisubstituted pyrrolidine 12 in 68% isolated yield (Scheme 4c).^15^ Additionally, these vicinal diamine products serve as effective ligands for promoting transfer hydrogenation reactions.^16^ For instance, the Mn-complex derived from 4a and Mn(CO)_5_Br efficiently converted dapsone (13), an antibiotic used in treating leprosy (Hansen's disease), to its *N*-benzyl derivative 14 using benzyl alcohol, achieving a 75% yield (Scheme 4d).

**Scheme 5:**
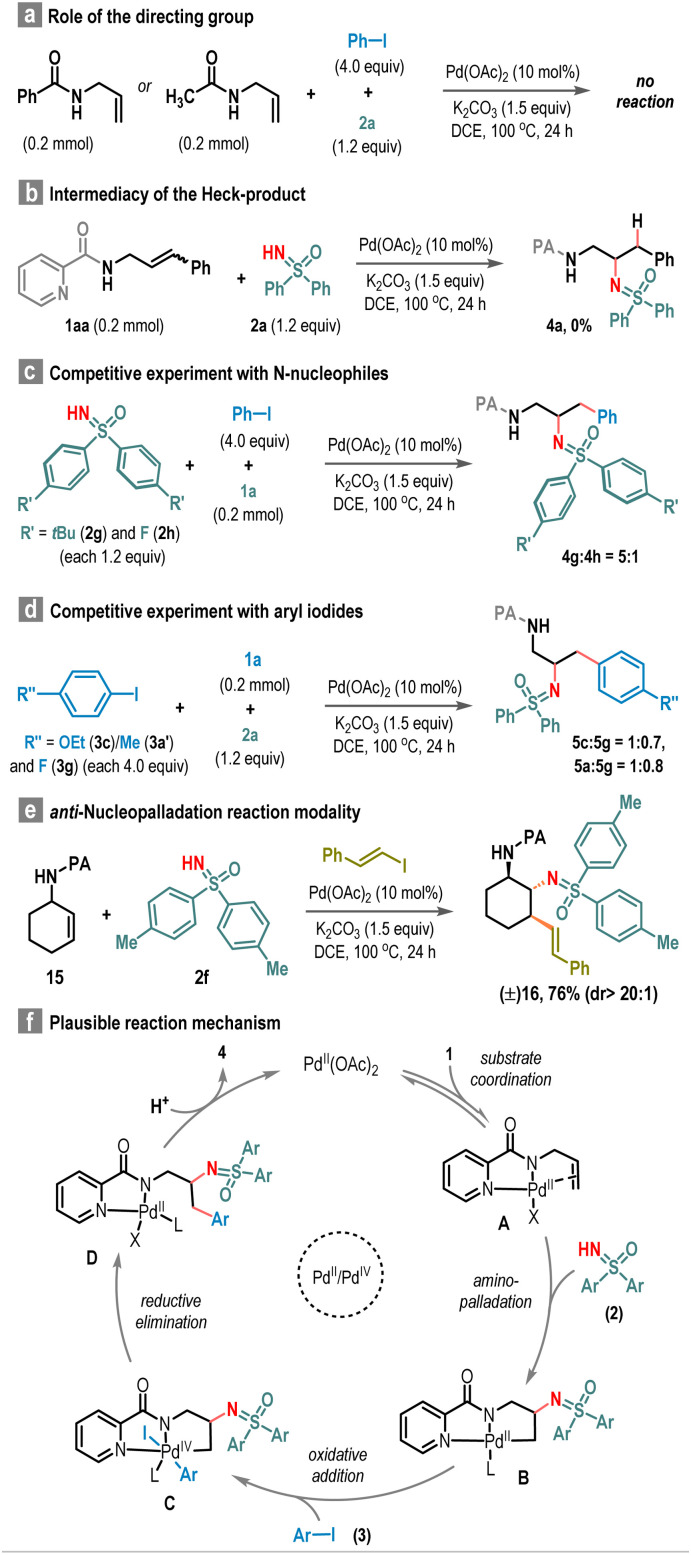
Mechanistic study.

Various control experiments were conducted to shed light on the reaction profile. The necessity of a coordinating directing group became evident as attempts using *N*-benzoyl and *N*-acyl allylamines failed to produce the desired outcome (Scheme 5a). Moreover, the unsuccessful coupling of allylamine 1aa with sulfoximine 2a under standard conditions ruled out the involvement of a Heck-product intermediate in this process (Scheme 5b). Further insights were gained from the competition experiment involving electronically distinct sulfoximines 2g and 2h, emphasizing that carboamination proceeds nearly five times faster with the electron-rich sulfoximine 2g (Scheme 5c). Similarly, competition reactions using electron-rich (3c or 3a′) and electron-deficient (3g) aryl iodides favored the electron-rich variant, albeit to a lesser extent compared to the impact observed with sulfoximines (Scheme 5d). To understand the nature of the nucleopalladation event, we performed the carboamination reaction with cyclic allylic amine (15) under standard reaction conditions, which resulted in three consecutively substituted cyclohexane derivative 16 in 76% yield as a spectroscopically observable single diastereomer. The presence of a *trans*–*trans* substitution pattern in the cyclohexane ring in 16, as confirmed by the NOE study, suggests an *anti*-nucleopalladation pathway for this reaction (Scheme 5e). Based on these findings and literature precedents, a reaction mechanism is proposed (Scheme 5f). Initially, the Pd(ii)-catalyst forms a coordinative complex with the picolinamide directing group and activates the carbon–carbon double bond of the allyl amine, generating intermediate A. Then, regioselective aminopalladation takes place to form more stable 5,5-palladacycle intermediate B. The subsequent reaction with the aryl iodide electrophile (3) leads to the formation of Pd(iv)-intermediate C, which undergoes reductive elimination to yield the desired carboamination product 4 through the intermediacy of D, along with the regeneration of the active Pd(ii)-catalyst to continue the catalytic cycles.

## Conclusions

In summary, we have developed an intermolecular carboamination reaction of allylamines under Pd(ii)-catalysis. Our approach leverages a nucleopalladation strategy facilitated by a removable bidentate PA-auxiliary, which stabilizes the 5,5-palladacycle, suppresses competitive β-hydride elimination and two-component coupling, and facilitates the simultaneous introduction of diverse aryl/styryl groups and important amine motifs like sulfoximines and anilines across carbon–carbon double bonds. This method enables a streamlined three-component synthesis of valuable vicinal diamines with high yields and exclusive Markovnikov selectivity. Furthermore, the reaction is scalable, tolerates a range of synthetically important functional groups, and remains effective in the presence of various medicinally relevant scaffolds. The products were also judiciously used in synthesizing functionally enriched peptides and heterocyclic frameworks, and as a ligand in the catalytic transfer hydrogenation process, showcasing their broad synthetic utility. Notably, this work represents a pioneering achievement in aminopalladation-guided intermolecular three-component carboamination of allylamines.

## Data availability

General information, experimental procedures, characterization data for all new compounds, and NMR spectra are in the ESI.† Data for the crystal structure reported in this paper have been deposited at the Cambridge Crystallographic Data Centre (CCDC) under the deposition numbers CCDC 2372817 and CCDC 2372870.

## Author contributions

The manuscript was written through the contributions of all authors. All authors have given approval to the final version of the manuscript.

## Conflicts of interest

There are no conflicts to declare.

## Supplementary Material

SC-OLF-D4SC07630C-s001

SC-OLF-D4SC07630C-s002
