# Supplementary material for: Regioselective intermolecular carboamination of allylamines via nucleopalladation: empowering three-component synthesis of vicinal diamines
Source: Chem Sci. 2024 Nov 29;16(1):386–92. doi: 10.1039/d4sc07630c (PMC11606157; doi:10.1039/d4sc07630c)
Supplement: SC-016-D4SC07630C-s001 [file SC-016-D4SC07630C-s001.pdf]

Supplementary Information (SI) for Chemical Science.
This journal is © The Royal Society of Chemistry 2024

– Electronic Supplementary Information –

Regioselective Intermolecular Carboamination of Allylamines via Nucleopalladation: Empowering Three-Component Synthesis of Vicinal Diamines

Shib Nath Saha, Nityananda Ballav, Suman Ghosh and Mahiuddin Baidya*

Department of Chemistry, Indian Institute of Technology Madras, Chennai-600036, India

Email: mbaidya@iitm.ac.in

Table of Contents

1) **General information ……………………………………………………..………………..… S3**

2) **Optimization details for vicinal carboamination reaction ………..…………………….S4**

3) **General procedure for three-component vicinal carboamination reaction with sulfoximine nucleophiles…………………….................………………………………………………...S6**

4) **General procedure for three-component vicinal carboamination reaction of homo allylamine with sulfoximine nucleophiles.……………………………….……………...……..S7**

5) **General procedure for three-component vicinal carboamination reaction with aniline nucleophiles………………………………………………………………………………...S7**

6) **Procedure for the scale-up vicinal carboamination reaction ……......………………........S8**

7) **Procedure for the removal of the directing group ………..…………………........…….S8**

8) **Procedure for the synthesis of amide 10 ………….……….…...……..……..…………..S9**

9) **Procedure for the synthesis of cyclic urea 11…….…...…...……..……………………...S9**

10) **Procedure for the aminoalkynylation reaction with 6a …......................…..…..…......S10**

11) **Procedure for the preparation and characterization of Mn-4a complex ……...........…S10**

12) **Procedure for the transfer hydrogenation reaction……...................…..…………….....S12**

13) **X-ray crystals data for products 5a and 8a..…………………………………………......S12**

14) **Spectroscopic data of synthesized compounds ………………………..…..……..S15**

15) **NMR spectra of synthesized compounds ……………………........…………...……. S47**

1. General information:

All reactions, unless mentioned otherwise, were carried out under air in flame-dried glassware and were stirred using a magnetic stir plate. Reactions were performed using commercial-grade solvent unless otherwise noted. CH_3CN and DCE were dried over calcium hydride. Tetrahydrofuran was freshly distilled over sodium ketyl before use.

All reactions were monitored by thin layer chromatography (TLC) on Merck 60 F 254 precoated silica plates and visualized using a UV lamp (366 or 254 nm) or by use of potassium permanganate, 5 g K_2CO_3/100 mL water. Products were isolated by column chromatography (Merck silica gel 100-200 μm).

^{13}C and ^{1}H NMR spectra were recorded on a Bruker 400 MHz or Bruker 500 MHz spectrometers. Chemical shift values (δ) are reported in ppm and calibrated to the residual solvent peak, $CDCl_3$ δ = 7.26 ppm for ^{1}H, δ = 77.16 for ^{13}C. All NMR spectra were recorded at ambient temperature (290 K) unless otherwise noted. ^{1}H NMR spectra are reported as follows: chemical shift (multiplicity, coupling constant, integration). The following abbreviations are used to indicate multiplicities: s, singlet; d, doublet; t, triplet; q, quartet; h, heptate; m, multiplet; dd, doublet of doublet; dt, doublet of triplet; dq, doublet of quartet; td, triplet of doublet; tt, triplet of triplet; dq, doublet of quartet; br, broad. NOESY experiments were performed to determine the relative stereochemistry of synthesize compounds as necessary.

Infrared (IR) spectra were recorded on a JASCO 4100 FT-IR spectrometer.

Mass spectra were recorded by electron spray ionization (ESI) method on a Q-TOF Micro with a lock spray source.

Sulfoximine derivatives were prepared following the literature procedures (*Chem. Commun.*, 2017, **53**, 348–351; *Adv. Synth. Catal.*, 2017, **359**, 4274–4277). Pharmacophore-coupled aryl iodides were synthesized according to the previous report (*Chem. Sci.*, 2024, **15**, 4890–4896).

2. Optimization details for vicinal carboamination reaction

Solvent screening:

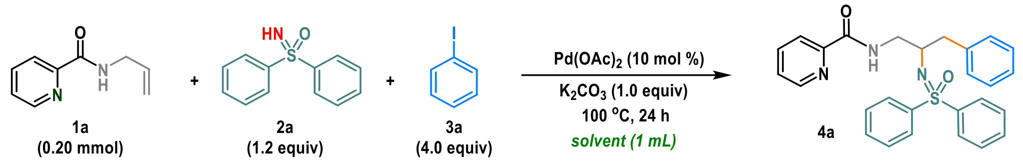

Entry	Solvent	Yield of **4a** (%)
1	HFIP	NR
2	TFE	NR
3	MeOH	NR
4	*t*BuOH	NR
5	Toluene	NR
6	THF	NR
7	MeCN	45
**8**	**DCE**	**67**
9	1,4-Dioxane	NR
10	DMA	NR
11	DMF	NR
12	DMSO	NR

Base screening:

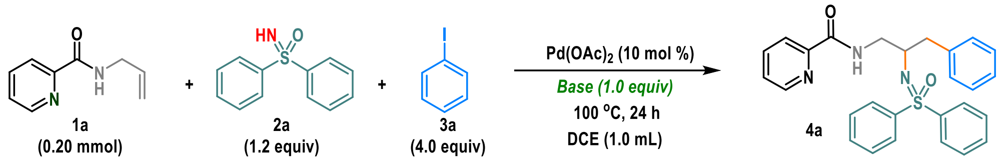

Entry	Base	Yield of **4a** (%)
1	Na_2CO_3	19
**2**	**K_2CO_3**	**67**
3	Cs_2CO_3	NR
4	Ag_2CO_3	NR
5	K_3PO_4	NR
6	Na_2HPO_4	NR
7	K_2HPO_4	NR
8	NaOAc	<5
9	KOAc	10
10	$KHCO_3$	NR
11	$NaHCO_3$	NR

Equivalent of base (K_2CO_3):

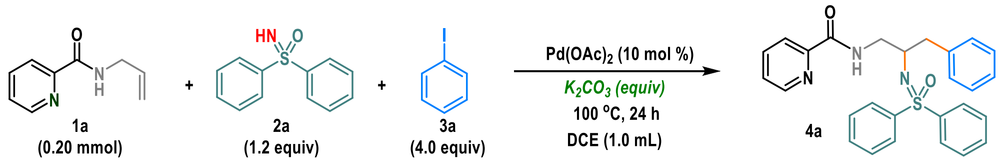

Entry	Loading of K_2CO_3	Yield of **4a** (%)
1	0.5 equiv	41
**2**	**1.5 equiv**	**84**
3	2.0 equiv	65
4	0.0 equiv	NR

Equivalents of nucleophile (2a) and electrophile (3a):

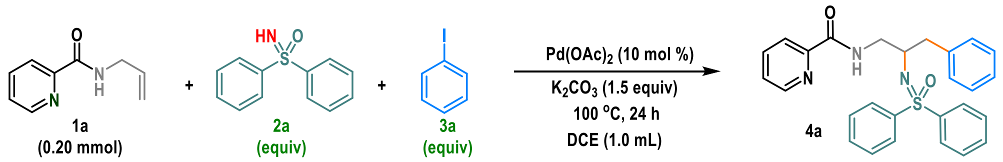

Entry	Equiv of **2a**	Equiv of **3a**	Yield of **4a** (%)
**1**	**1.2**	**4.0**	**84**
2	2.0	4.0	60
3	1.2	3.0	56
4	1.2	2.0	49

Temperature screening:

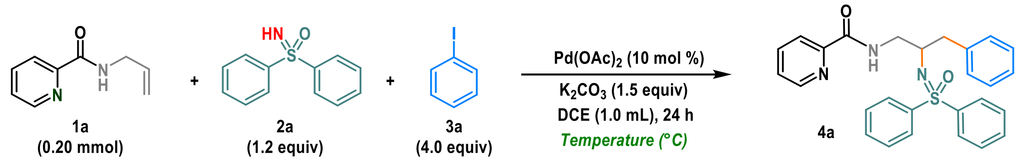

Entry	Temperature (°C)	Yield of **4a** (%)
1	rt	NR
2	60	45
3	80	58
**4**	**100**	**84**
5	120	71

Catalyst screening:

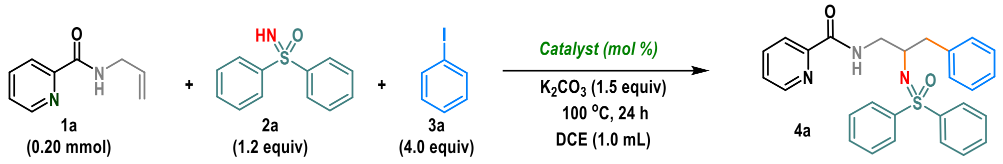

Entry	Catalyst (mol %)	Yield of **4a** (%)
**1**	**$Pd(OAc)_2$ (10.0 mol %)**	**84**
2	$Pd(OAc)_2$ (5.0 mol %)	60
3	$Ni(OAc)_2$ (10.0 mol %)	NR
4	$Co(OAc)_2{\cdot}4H_2O$ (10.0 mol %)	NR
5	$Pd(OAc)_2$ (0.0 mol %)	NR

Unsuccessful substrates:

Nucleophiles:

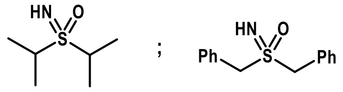

Electrophiles:

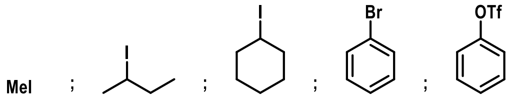

3. General procedure for three-component vicinal carboamination reaction with sulfoximine nucleophiles

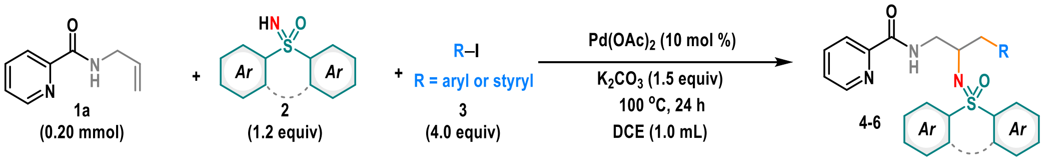

GP-1:

An oven-dried screw cap reaction tube equipped with a magnetic stirrer bar was charged with *N*-allylpicolinamide **1a** (0.20 mmol, 1.0 equiv), corresponding sulfoximine nucleophile **2** (1.2 equiv), aryl iodide or styryl iodide **3** (4.0 equiv), K_2CO_3 (1.5 equiv), and $Pd(OAc)_2$ (10 mol %). Then, DCE (1 mL, 5M) solvent was added via syringe. The reaction tube was capped and placed in a preheated oil bath at 100 °C for 24 h. After completion of the reaction (monitored by TLC),

the crude reaction mixture was diluted with DCM and concentrated under reduced pressure. The crude residue was purified through column chromatography on silica gel using ethyl acetate in hexane to get pure product **4-6**.

4. General procedure for three-component vicinal carboamination reaction of homoallylamine with sulfoximine nucleophiles

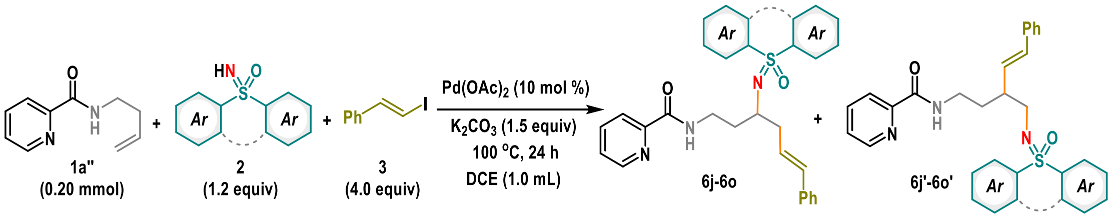

GP-2:

An oven-dried screw cap reaction tube equipped with a magnetic stirrer bar was charged with *N*-homoallylpicolinamide **1a″** (0.20 mmol, 1.0 equiv), corresponding sulfoximine nucleophile **2** (1.2 equiv), styryl iodide **3** (4.0 equiv), K_2CO_3 (1.5 equiv), and $Pd(OAc)_2$ (10 mol %). Then, DCE (1 mL, 5M) solvent was added via syringe. The reaction tube was capped and placed in a preheated oil bath at 100 °C for 24 h. After completion of the reaction (monitored by TLC), the crude reaction mixture was diluted with DCM and concentrated under reduced pressure. The crude residue was purified through column chromatography on silica gel using ethyl acetate in hexane to get pure product **6j-6o** and **6j′-6o′**.

5. General procedure for three-component vicinal carboamination reaction with aniline nucleophiles

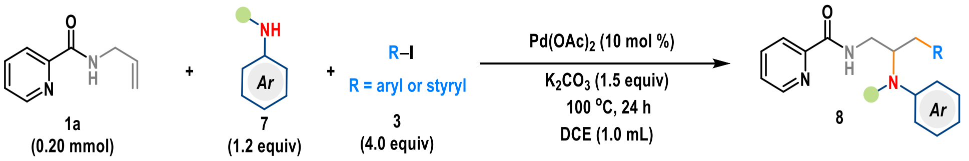

GP-3:

An oven-dried screw cap reaction tube equipped with a magnetic stirrer bar was charged with *N*-allylpicolinamide **1a** (0.20 mmol, 1.0 equiv), corresponding aniline nucleophile **7** (1.2 equiv), aryl iodide or styryl iodide **3** (4.0 equiv), K_2CO_3 (1.5 equiv), and $Pd(OAc)_2$ (10 mol %). Then, DCE (1 mL, 5M) solvent was added via syringe. The reaction tube was capped and placed in a

preheated oil bath at 100 °C for 24 h. After completion of the reaction (monitored by TLC), the crude reaction mixture was diluted with DCM and concentrated under reduced pressure. The crude residue was purified through column chromatography on silica gel using ethyl acetate in hexane to get pure product **8**.

6. Procedure for the scale-up vicinal carboamination reaction

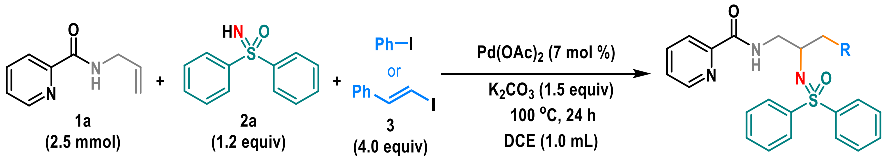

An oven-dried screw cap reaction tube equipped with a magnetic stirrer bar was charged with *N*-allylpicolinamide **1a** (2.50 mmol, 1.0 equiv), sulfoximine **2a** (1.2 equiv), iodo benzene **3a** (4.0 equiv), K_2CO_3 (1.5 equiv), and $Pd(OAc)_2$ (7 mol %). Then, DCE (12.5 mL, 5M) solvent was added via syringe. The reaction tube was capped and placed in a preheated oil bath at 100 °C for 24 h. After completion of the reaction (monitored by TLC), the crude reaction mixture was diluted with DCM and concentrated under reduced pressure. The crude residue was purified through column chromatography on silica gel using ethyl acetate in hexane to get pure product **4a** (0.81 g, 71%).

Similarly, product **6a** (0.91g, 76%) was prepared from *N*-allylpicolinamide **1a** (2.50 mmol, 1.0 equiv), sulfoximine **2a** (1.2 equiv), and styryl iodide (4.0 equiv).

7. Procedure for the removal of the directing group

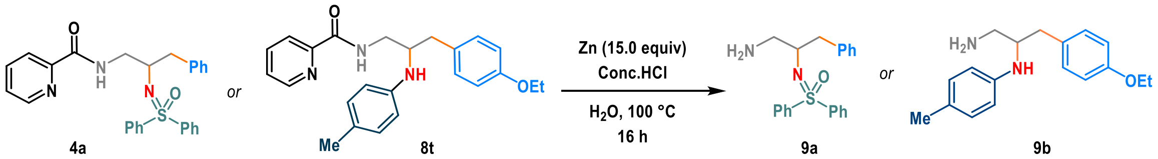

To a suspension of compound **4a** or **8t** (0.3 mmol, 1.0 equiv) in H_2O (6 mL) was added conc.HCl (0.7 mL) and the mixture was stirred for 5 minutes at room temperature. Zinc dust (292 mg, 4.48 mmol, 15 equiv) was then carefully added portion wise and the mixture was stirred at 100 °C for 16 h. Upon completion (TLC monitored), the reaction mixture was filtered through a celite plug. The filtrate was neutralized with 2M NaOH solution and then extracted with DCM. The volatiles were evaporated under reduced pressure and the crude residue was purified through column chromatography on silica gel using 15% MeOH in DCM to get pure product **9a** (93 mg, 89%) or **9b** (77 mg, 90%).

8. Procedure for the synthesis of amide 10

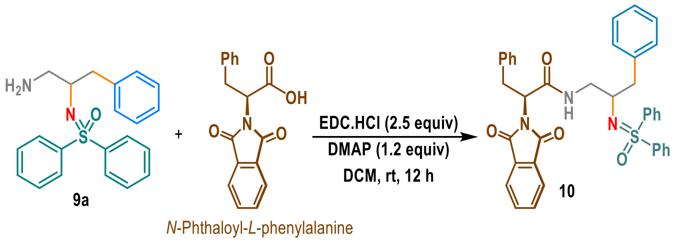

N-Phthaloyl-*L*-phenylalanine (0.165 mmol, 1.1 equiv) and 4-*N*,*N*-dimethylaminopyridine (DMAP, 1.2 equiv) were taken in a 50 mL round bottom flask under nitrogen. Anhydrous DCM (5 mL) was added and the mixture was cooled to 0 °C. *N*'-(3-dimethylaminopropyl)-*N*-ethylcarbodimide hydrochloride salt (EDC·HCl, 2.5 equiv) was added under nitrogen and the mixture was stirred for 10 minutes at the same temperature. Then, compound **9a** (52 mg, 0.15 mmol, 1.0 equiv) was added and the mixture was stirred at room temperature for 12 h. Upon completion (TLC monitored), 10% aqueous $NaHCO_3$ solution (15 mL) was added to the reaction mixture and extracted with DCM. The organic layer was washed with brine, dried over Na_2SO_4, and evaporated under reduced pressure. The crude residue was purified through column chromatography on silica gel using ethyl acetate in hexane to get the pure product **10** (70 mg, 74%).

9. Procedure for the synthesis of cyclic urea 11

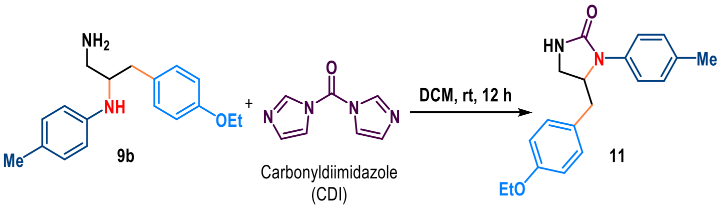

To an oven-dried 50 mL round bottom flask, product **9b** (0.3 mmol, 1.0 equiv) was taken under nitrogen. Then, anhydrous DCM (4.0 mL) followed by carbonyldiimidazole (0.6 equiv) was added and the mixture was allowed to stirred at rt for 12 h under nitrogen. After completion of the reaction (TLC monitored), the crude reaction mixture was diluted with DCM and concentrated under reduced pressure. The crude residue was purified through column chromatography on silica gel using 30% ethyl acetate in hexane to get pure product **11** (88 mg, 95%).

10. Procedure for the aminoalkynylation reaction with 6a

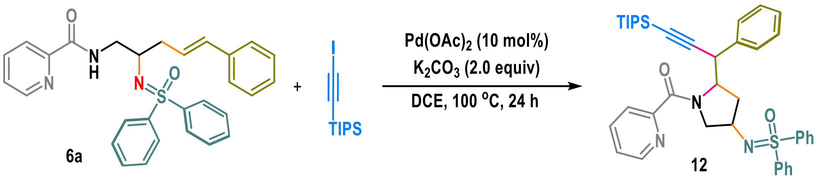

An oven-dried screw cap reaction tube equipped with a magnetic stirrer bar was charged with compound **6a** (0.15 mmol, 1.0 equiv), (iodoethynyl)triisopropylsilane (1.3 equiv), $Pd(OAc)_2$ (10 mol %), and K_2CO_3 (2.0 equiv). Then, DCE (1.5 mL, 1M) solvent was added via syringe. The reaction tube was capped and placed in a preheated oil bath at 100 °C for 24 h. After completion of the reaction (TLC monitored), the crude mixture was diluted with DCM and concentrated under reduced pressure. The crude residue was purified through column chromatography on silica gel using ethyl acetate in hexane to get pure product **12** (67 mg, 68%).

11. Procedure for the preparation and characterization of Mn-4a complex

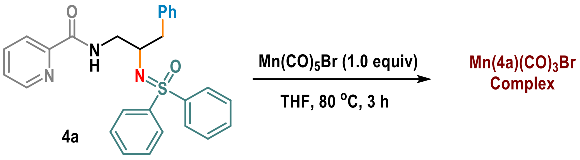

The $[MnBr(CO)_5]$ (0.2 mmol) was taken in a reaction tube under argon atmosphere and degassed dry THF (5 mL) was added via syringe to get an orange-yellow suspension. Then, a solution of compound **4a** (0.2 mmol) in 5 mL THF was added dropwise to it. The mixture was refluxed for 3 h under argon atmosphere. After cooling it down to the room temperature, the solvent was evaporated to obtain the residue, which was further washed with hexane and dried under vacuum to get yellow solid of **Mn(4a)(CO)$_3$Br** complex (114 mg, 85%).

Characterization of Mn-4a Complex:

^{13}C NMR (126 MHz, $CDCl_3$) δ 224.8, 221.7, 221.1, 169.4, 153.7, 149.0, 139.8, 138.7, 137.8, 133.4, 132.9, 132.0, 131.6, 129.5, 129.4, 128.6, 128.5, 128.2, 128.1, 123.6, 120.6, 56.4, 47.3, 42.0.

IR (KBr pellet) ν_{max} 3450, 2929, 2824, 2023, 1922, 1634, 1602, 1449, 1357, 1237, 1136, 1038, 691 cm^{-1}.

HRMS (ESI/TOF-Q) m/z: $[M]^+$ Calculated for $C_{30}H_{25}MnN_3O_5S^+$ 594.0890; Found 594.0895.

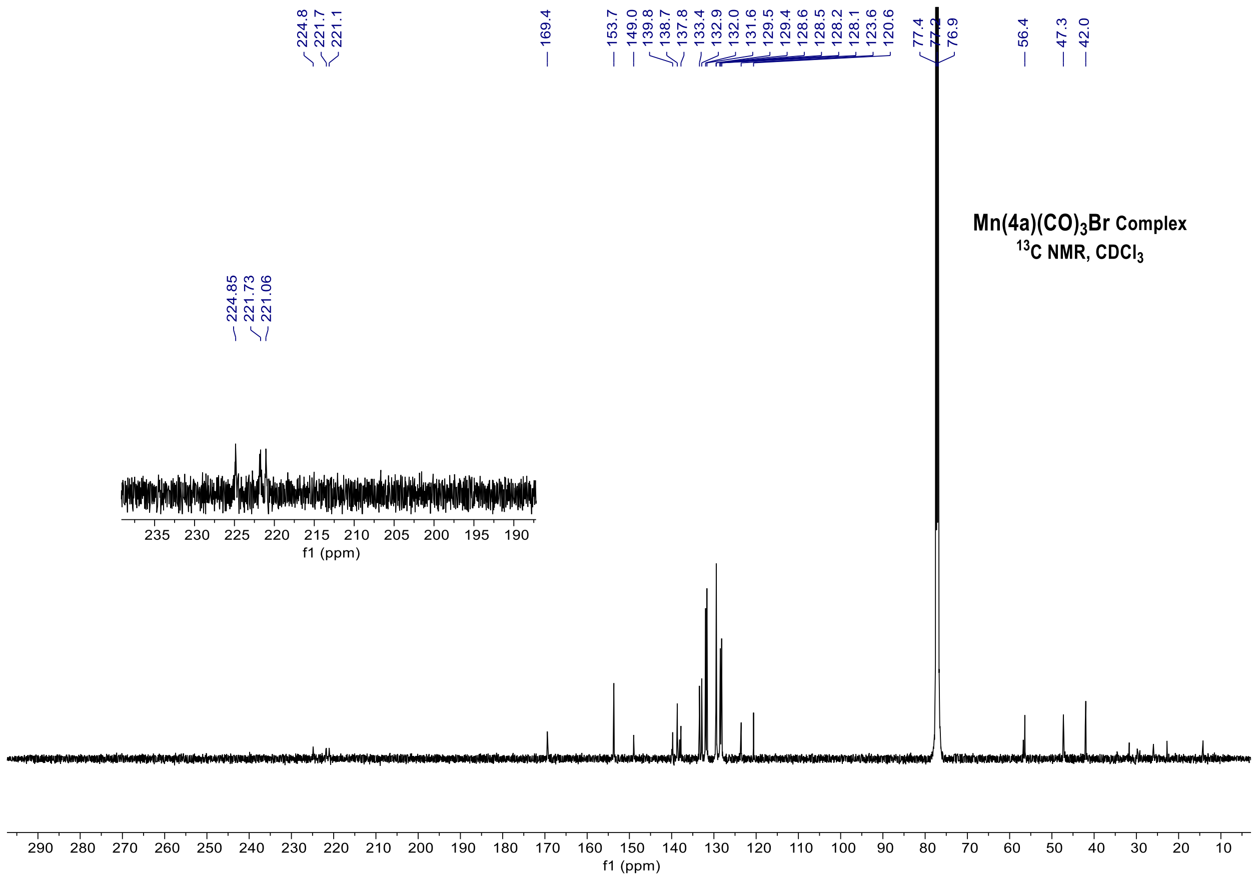

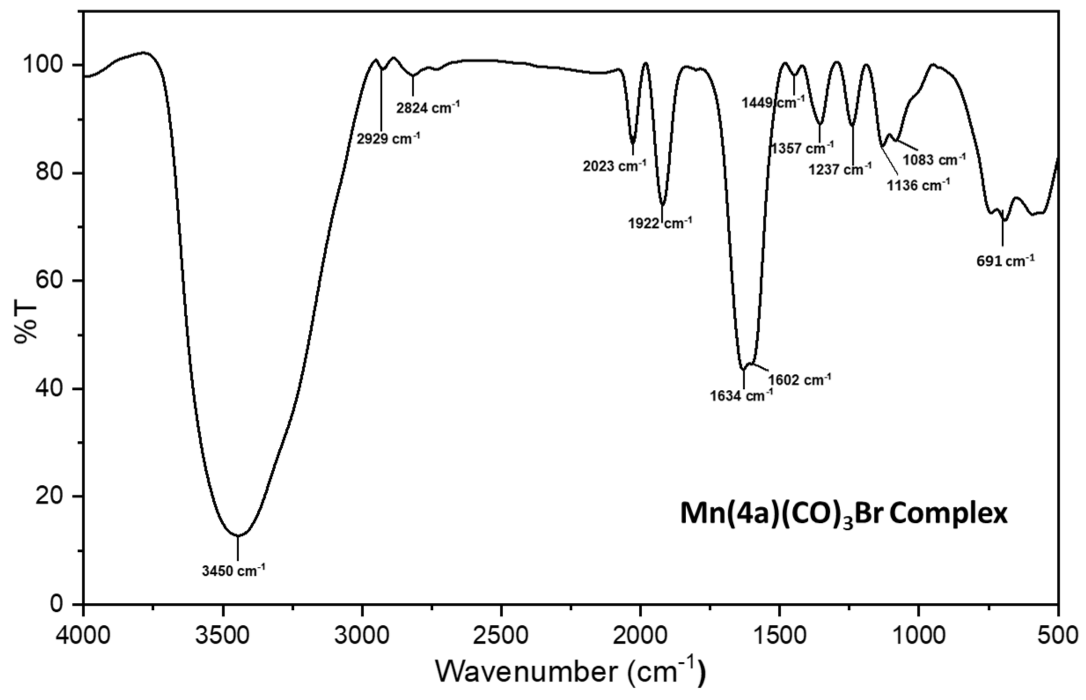

IR spectra of Mn(4a)(CO)$_3$Br Complex

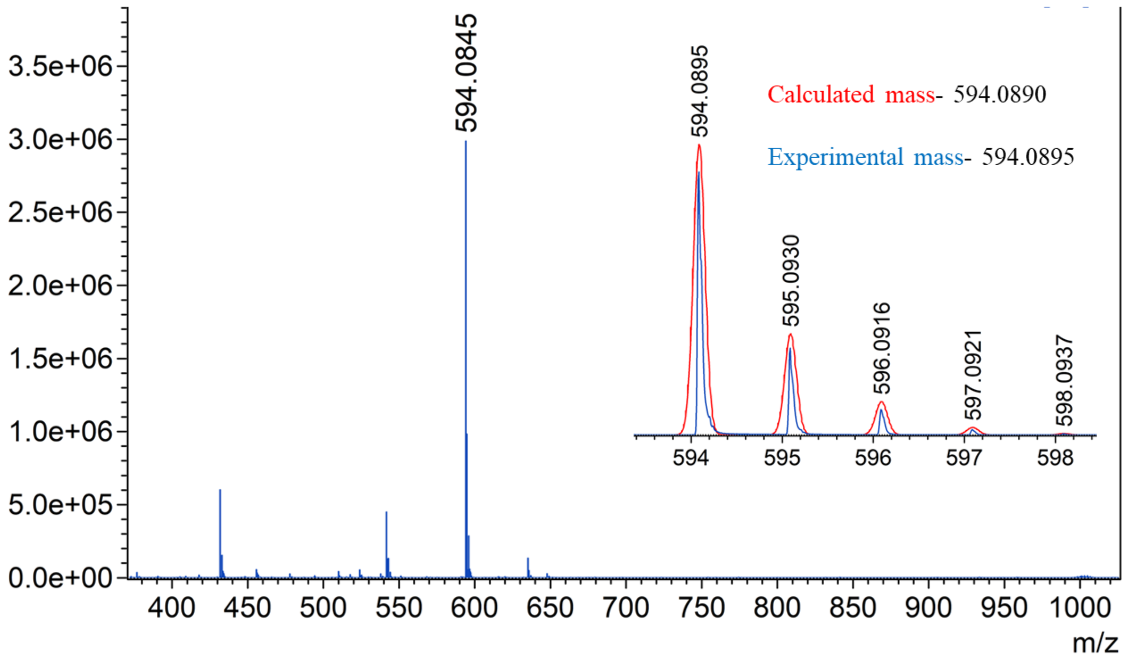

HRMS of Mn(4a)$(CO)_3$Br Complex

12. Procedure for the transfer hydrogenation reaction

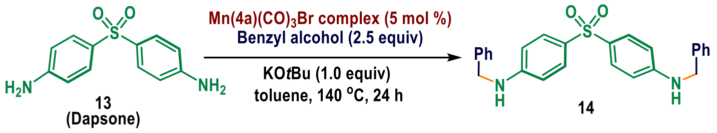

An oven-dried 25 mL pressure tube equipped with magnetic stirrer bar was charged with Dapsone (**13**, 0.2 mmol), benzyl alcohol (0.5 mmol, 2.5 equiv), KO*t*Bu (1.0 equiv), and **Mn(4a)$(CO)_3$Br** complex (5 mol %) under argon atmosphere. Then, degassed dry toluene (2 mL) was added via syringe and the mixture was heated in an oil bath at 140 °C for 24 h. After the completion of the reaction, it was cooled to room temperature and diluted with DCM. Volatiles were removed under reduced pressure and the crude residue was purified through column chromatography on silica gel using 35% ethyl acetate in hexane to get pure product **14** (64 mg, 75%).

13. X-ray crystals data for products 5a and 8a

Crystallographic data of product 5a:

Crystals of compound **5a** were obtained through a slow evaporation technique at room temperature from $CDCl_3$ / hexane solvent mixture.

Crystal structure of compound **5a** (CCDC number: 2372817, Ellipsoid Probability 50%):

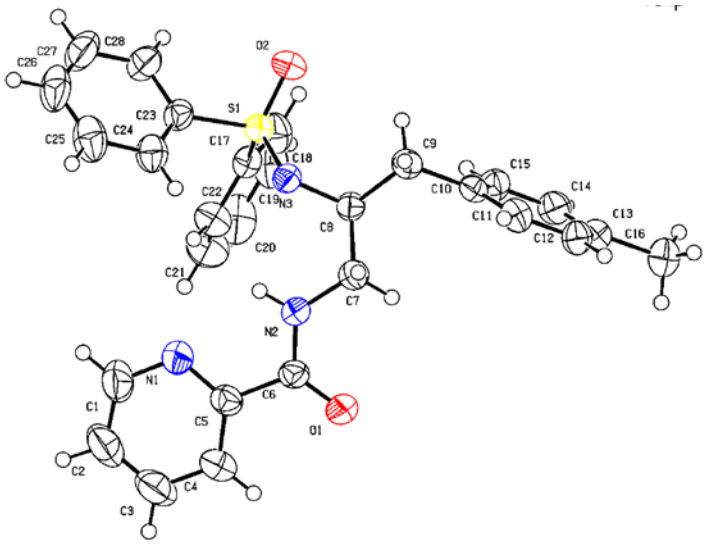

Table S1. Crystal data and structure refinement for compound **5a**.

Identification code	**5a**
Empirical formula	$C_{28}H_{27}N_3O_2S$
Formula weight	469.58
Temperature	298(2) K
Wavelength	0.71073 A
Crystal system, space group	Triclinic, P -1
Unit cell dimensions	a = 9.4004(5) A alpha = 97.488(2) deg.
	b = 10.1110(7) A beta = 104.094(2) deg.
	c = 13.6049(9) A gamma = 95.420(2) deg.
Volume	1232.74(14) A^3
Z, Calculated density	2, 1.265 Mg/m^3
Absorption coefficient	0.161 mm^-1
F(000)	496
Crystal size	0.249 x 0.175 x 0.126 mm
Theta range for data collection	2.840 to 25.657 deg.

Limiting indices -11<=h<=11, -12<=k<=12, -16<=l<=16

Reflections collected / unique 48138 / 4655 [R(int) = 0.0952]

Completeness to theta = 25.242 99.6 %

Absorption correction Semi-empirical from equivalents

Max. and min. transmission 1.0000 and 0.7161

Refinement method Full-matrix least-squares on F^2

Data / restraints / parameters 4655 / 0 / 308

Goodness-of-fit on F^2 1.078

Final R indices [I>2sigma(I)] R1 = 0.0493, wR2 = 0.1052

R indices (all data) R1 = 0.0744, wR2 = 0.1165

Extinction coefficient n/a

Largest diff. peak and hole 0.198 and -0.312 e.A^-3

Crystallographic data of product 8a

Crystals of compound **8a** were obtained through a slow evaporation technique at room temperature from $CDCl_3$ / hexane solvent mixture.

Crystal structure of compound **8a** (CCDC number: 2372870, Ellipsoid Probability 50%):

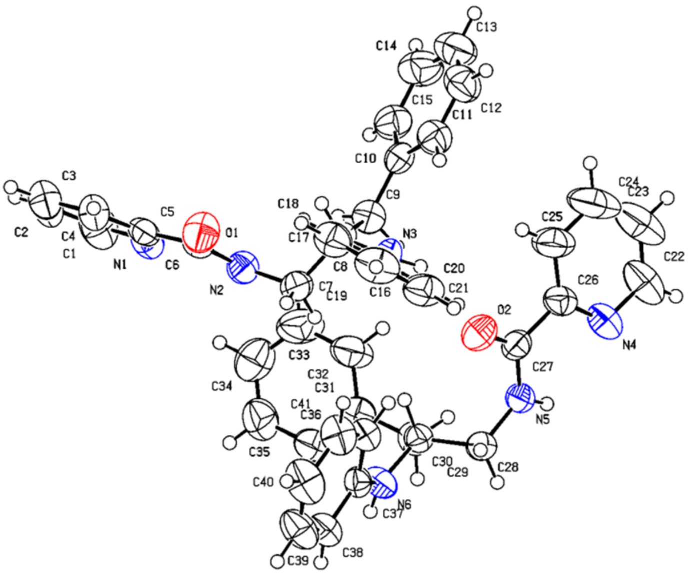

Table S2. Crystal data and structure refinement for **8a** (dimeric structure)

Identification code **8a**

Empirical formula C_{42} H_{42} N_6 O_2

Formula weight 662.81

Temperature 296(2) K

Wavelength 0.71073 A

Crystal system, space group Monoclinic, P2(1)/c

Unit cell dimensions a = 9.9446(3) A alpha = 90 deg.

b = 20.0301(7) A beta = 95.7521(16) deg.

c = 18.2275(6) A gamma = 90 deg.

Volume 3612.5(2) A^3

Z, Calculated density 4, 1.219 Mg/m^3

Absorption coefficient 0.077 mm^-1

F(000) 1408

Crystal size 0.310 x 0.140 x 0.120 mm

Theta range for data collection 1.515 to 24.999 deg.

Limiting indices -11<=h<=11, -23<=k<=23, -21<=l<=20

Reflections collected / unique 25840 / 6367 [R(int) = 0.0310]

Completeness to theta = 24.999 100.0 %

Absorption correction None

Refinement method Full-matrix least-squares on F^2

Data / restraints / parameters 6367 / 0 / 451

Goodness-of-fit on F^2 1.003

Final R indices [I>2sigma(I)] R1 = 0.0481, wR2 = 0.1105

R indices (all data) R1 = 0.0932, wR2 = 0.1388

Extinction coefficient n/a

Largest diff. peak and hole 0.249 and -0.186 e.A^-3

14. Spectroscopic data of synthesized compounds

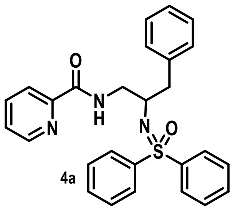
4a

N-(2-((oxodiphenyl-l6-sulfaneylidene)amino)-3-phenylpropyl)picolinamide (4a): Compound **4a** was synthesized according to GP-1 as off white solid; eluent (35% ethyl acetate in hexane); m.p.: 118 – 120 °C; **Yield:** 84% (76 mg); **^{1}H NMR (400 MHz, $CDCl_3$)** δ 8.77 – 8.74 (m, 1H), 8.60 (d, *J* = 4.8 Hz, 1H), 8.12 (d, *J* = 7.8 Hz, 1H), 7.89 (d, *J* = 7.8 Hz, 2H), 7.79 – 7.77 (m, 1H), 7.43 – 7.30 (m, 8H), 7.24 – 7.17 (m, 6H), 3.83 – 3.77 (m, 1H), 3.46 – 3.35 (m, 2H), 2.94 (d, *J* = 6.6 Hz, 2H); **^{13}C NMR (101 MHz, $CDCl_3$)** δ 164.5, 150.5, 148.3, 141.1, 139.7, 137.3, 132.6, 132.3, 130.2, 129.1 (2xC), 129.0, 128.9, 128.7,

128.3, 126.3, 126.0, 122.4, 57.7, 45.9, 43.0; **HRMS** (ESI/TOF-Q) m/z: $[M+H]^+$ Calculated for $C_{27}H_{25}N_3O_2SH^+$ 456.1740; Found 456.1758.

N-(2-((oxodi-o-tolyl-l6-sulfaneylidene)amino)-3-phenylpropyl)picolinamide (4b):

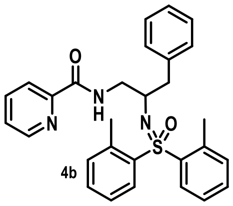

4b

Compound **4b** was synthesized according to GP-1 as brown sticky liquid; eluent (30% ethyl acetate in hexane); **Yield:** 83% (80 mg); **^{1}H NMR (400 MHz, CDCl$_3$)** δ 8.72 (brs, 1H), 8.59 (d, *J* = 4.5 Hz, 1H), 8.41 (d, *J* = 7.8 Hz, 1H), 8.22 (d, *J* = 7.9 Hz, 1H), 8.16 (d, *J* = 7.8 Hz, 1H), 7.83 – 7.79 (m, 1H), 7.41 – 7.33 (m, 4H), 7.22 – 7.19 (m, 3H), 7.15 – 7.07 (m, 5H), 3.66 – 3.61 (m, 1H), 3.56 – 3.50 (m, 1H), 3.46 – 3.42 (m, 1H), 2.94 (d, *J* = 6.3 Hz, 2H), 2.18 (s, 3H), 1.92 (s, 3H); **^{13}C NMR (101 MHz, CDCl$_3$)** δ 164.5, 150.6, 148.3, 139.4, 138.5, 138.08, 138.05, 138.0, 137.2, 132.8, 132.7, 132.6, 131.2, 130.8, 129.8 (2xC), 128.3 (2xC), 126.2, 126.1, 125.9, 122.3, 57.0, 45.3, 43.0, 20.1, 19.7; **HRMS** (ESI/TOF-Q) m/z: $[M+H]^+$ Calculated for $C_{29}H_{29}N_3O_2SH^+$ 484.2053; Found 484.2078.

N-(2-((oxodi-m-tolyl-l6-sulfaneylidene)amino)-3-phenylpropyl)picolinamide (4c):

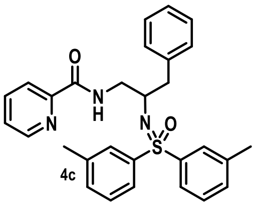

4c

Compound **4c** was synthesized according to GP-1 as sticky brown liquid; eluent (30% ethyl acetate in hexane); **Yield:** 86% (83 mg); **^{1}H NMR (500 MHz, CDCl$_3$)** δ 8.82 – 8.80 (m, 1H), 8.60 (d, *J* = 4.7 Hz, 1H), 8.10 (d, *J* = 7.8 Hz, 1H), 7.76 (t, *J* = 7.7 Hz, 1H), 7.65 (s, 2H), 7.37 – 7.34 (m, 1H), 7.28 – 7.23 (m, 3H), 7.20 – 7.17 (m, 5H), 7.11 (d, *J* = 7.8 Hz, 2H), 7.03 (t, *J* = 7.7 Hz, 1H), 3.84 – 3.80 (m, 1H), 3.38 – 3.29 (m, 2H), 2.92 (d, *J* = 6.2 Hz, 2H), 2.22 (s, 3H), 2.12 (s, 3H); **^{13}C NMR (126 MHz, CDCl$_3$)** δ 164.4, 150.5, 148.3, 140.9, 139.8, 139.4, 139.2, 139.1, 137.2, 133.3, 133.1, 130.1, 129.1, 129.0, 128.84, 128.80, 128.2, 126.2, 126.0 (2xC), 125.9, 122.3, 57.8, 45.9, 43.0, 21.34, 21.29; **HRMS** (ESI/TOF-Q) m/z: $[M+H]^+$ Calculated for $C_{29}H_{29}N_3O_2SH^+$ 484.2053; Found 484.2042.

N-(2-((bis(3-fluorophenyl)(oxo)-l6-sulfaneylidene)amino)-3-phenylpropyl)-picolinamide (4d):

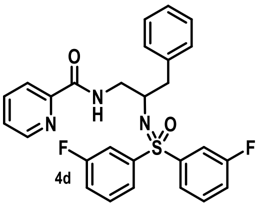

4d

Compound **4d** was synthesized according to GP-1 as sticky colourless liquid; eluent (40% ethyl acetate in hexane); **Yield:** 62% (61 mg); **^{1}H NMR (500 MHz, CDCl$_3$)** δ 8.77 – 8.74 (m, 1H), 8.66 (d, *J* = 4.8 Hz, 1H), 8.16 (d, *J* = 7.8 Hz, 1H), 7.85 (t, *J* = 7.8 Hz, 1H), 7.68 (d, *J* = 7.9 Hz, 1H), 7.64 – 7.62 (m, 1H), 7.45 (t, *J* = 6.3 Hz, 1H), 7.38 – 7.32 (m, 3H), 7.28 (s, 1H), 7.24 (s, 2H), 7.21 – 7.16 (m, 2H), 7.09 (t, *J* = 8.2 Hz, 3H), 3.90 – 3.85 (m, 1H), 3.48 – 3.40 (m, 2H), 3.03 – 2.92 (m, 2H); **^{13}C NMR (126 MHz, CDCl$_3$)** δ 164.6, 162.6 (d, *J* = 251.8 Hz), 162.5 (d, *J* = 252.1 Hz), 150.3, 148.4, 143.0 (d, *J* = 6.6 Hz), 141.5 (d, *J* = 6.1 Hz), 139.5, 137.4, 130.8 (d, *J* = 7.5 Hz), 130.7 (d, *J* = 7.5 Hz), 130.1, 128.4, 126.6, 126.2 124.7 (d, *J* = 3.7 Hz), 124.5 (d, *J* = 3.4 Hz), 122.4, 120.1 (d, *J* = 27.8 Hz), 119.9 (d, *J* = 27.7 Hz), 116.3 (d, *J* = 24.3 Hz), 116.2 (d, *J* = 24.8 Hz), 58.1, 46.0, 42.8; **^{19}F NMR (471 MHz, CDCl$_3$)** δ -109.3, -109.7; **HRMS** (ESI/TOF-Q) m/z: $[M+H]^+$ Calculated for $C_{27}H_{23}F_2N_3O_2SH^+$ 492.1552; Found 492.1575.

N-(2-((bis(3-chlorophenyl)(oxo)-l6-sulfaneylidene)amino)-3-phenylpropyl)-picolinamide (4e):

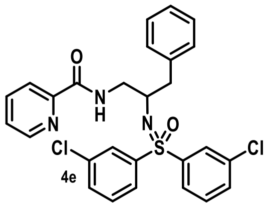

Compound **4e** was synthesized according to GP-1 as sticky colourless liquid; eluent (40% ethyl acetate in hexane); **Yield:** 64% (67 mg); **^{1}H NMR (500 MHz, CDCl$_3$)** δ 8.79 – 8.77 (m, 1H), 8.68 (d, *J* = 4.7 Hz, 1H), 8.16 (d, *J* = 7.8 Hz, 1H), 7.91 (s, 1H), 7.85 (t, *J* = 7.7 Hz, 1H), 7.74 (d, *J* = 8.0 Hz, 1H), 7.49 (s, 1H), 7.46 – 7.44 (m, 2H), 7.37 – 7.34 (m, 3H), 7.32 – 7.26 (m, 4H), 7.12 (t, *J* = 7.9 Hz, 1H), 7.02 (d, *J* = 7.9 Hz, 1H), 3.92 – 3.87 (m, 1H), 3.45 – 3.37 (m, 2H), 3.04 – 2.91 (m, 2H); **^{13}C NMR (126 MHz, CDCl$_3$)** δ 164.6, 150.3, 148.5, 142.5, 140.9, 139.6, 137.4, 135.39, 135.36, 133.1, 132.8, 130.38, 130.35, 130.1, 129.04, 128.96, 128.5, 127.0, 126.9, 126.6, 126.2, 122.5, 58.3, 46.0, 42.7; **HRMS** (ESI/TOF-Q) m/z: [M+H]$^+$ Calculated for $C_{27}H_{23}Cl_2N_3O_2SH^+$ 524.0961; Found 524.0962.

N-(2-((oxodi-p-tolyl-l6-sulfaneylidene)amino)-3-phenylpropyl)picolinamide (4f):

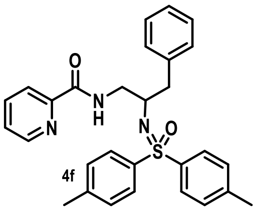

Compound **4f** was synthesized according to GP-1 as sticky colourless liquid; eluent (30% ethyl acetate in hexane); **Yield:** 87% (84 mg); **^{1}H NMR (500 MHz, CDCl$_3$)** δ 8.82 – 8.80 (m, 1H), 8.64 (d, *J* = 4.7 Hz, 1H), 8.16 (d, *J* = 7.9 Hz, 1H), 7.84 – 7.78 (m, 3H), 7.42 (t, *J* = 5.6 Hz, 1H), 7.33 – 7.28 (m, 4H), 7.25 – 7.23 (m, 3H), 7.14 (d, *J* = 7.9 Hz, 2H), 7.02 (d, *J* = 7.9 Hz, 2H), 3.86 – 3.81 (m, 1H), 3.48 – 3.38 (m, 2H), 2.98 (d, *J* = 6.6 Hz, 2H), 2.31 (s, 3H), 2.29 (s, 3H); **^{13}C NMR (126 MHz, CDCl$_3$)** δ 164.4, 150.5, 148.3, 143.1, 142.9, 139.7, 138.3, 137.2, 137.0, 130.1, 129.64, 129.62, 128.8, 128.6, 128.2, 126.2, 126.0, 122.3, 57.6, 45.8, 43.0, 21.45, 21.43; **HRMS** (ESI/TOF-Q) m/z: [M+H]$^+$ Calculated for $C_{29}H_{29}N_3O_2SH^+$ 484.2053; Found 484.2039.

N-(2-((bis(4-(tert-butyl)phenyl)(oxo)-l6-sulfaneylidene)amino)-3-phenylpropyl)picolinamide (4g):

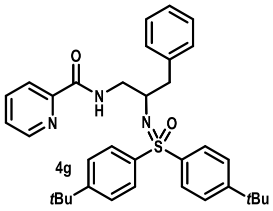

Compound **4g** was synthesized according to GP-1 as sticky colourless liquid; eluent (20% ethyl acetate in hexane); **Yield:** 90% (102 mg); **^{1}H NMR (400 MHz, CDCl$_3$)** δ 8.78 – 8.75 (m, 1H), 8.60 (d, *J* = 4.4 Hz, 1H), 8.12 (d, *J* = 7.8 Hz, 1H), 7.83 – 7.77 (m, 3H), 7.40 – 7.37 (m, 1H), 7.33 – 7.31 (m, 4H), 7.23 (d, *J* = 7.1 Hz, 2H), 7.22 – 7.17 (m, 5H), 3.82 – 3.77 (m, 1H), 3.44 – 3.34 (m, 2H), 2.92 (d, *J* = 6.4 Hz, 2H), 1.21 (d, *J* = 4.0 Hz, 18H); **^{13}C NMR (101 MHz, CDCl$_3$)** δ 164.6, 156.1, 155.8, 150.6, 148.3, 139.9, 138.2, 137.2, 136.9, 130.2, 128.7, 128.5, 128.2, 126.2, 126.1, 126.01, 125.98, 122.4, 57.5, 45.9, 43.1, 35.1, 35.0, 31.16, 31.15; **HRMS** (ESI/TOF-Q) m/z: [M+H]$^+$ Calculated for $C_{35}H_{41}N_3O_2SH^+$ 568.2992; Found 568.2977.

N-(2-((bis(4-fluorophenyl)(oxo)-l6-sulfaneylidene)amino)-3-phenylpropyl)-picolinamide (4h):

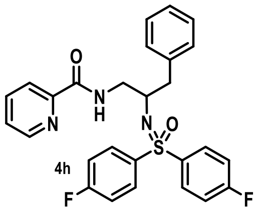

4h

Compound **4h** was synthesized according to GP-1 as sticky colourless liquid; eluent (40% ethyl acetate in hexane); **Yield:** 63% (62 mg); **^{1}H NMR (400 MHz, CDCl$_3$)** δ 8.73 (brs, 1H), 8.66 (d, *J* = 4.3 Hz, 1H), 8.20 (d, *J* = 7.8 Hz, 1H), 7.96 – 7.85 (m, 3H), 7.48 – 7.45 (m, 1H), 7.36 – 7.32 (m, 4H), 7.29 – 7.27 (m, 3H), 7.07 (t, *J* = 8.6 Hz, 2H), 6.90 (t, *J* = 8.5 Hz, 2H), 3.90 – 3.83 (m, 1H), 3.54 – 3.40 (m, 2H), 3.05 – 2.93 (m, 2H); **^{13}C NMR (126 MHz, CDCl$_3$)** δ 165.3 (d, *J* = 255.4 Hz), δ 165.0 (d, *J* = 254.8 Hz), 164.6, 150.4, 148.3, 139.8, 137.4, 137.1 (d, *J* = 3.3 Hz), 135.5 (d, *J* = 3.3 Hz), 131.5 (d, *J* = 26.6 Hz), 131.4 (d, *J* = 26.4 Hz), 130.2, 128.3, 126.4, 126.1, 122.4, 116.4 (d, *J* = 3.9 Hz), 116.2 (d, *J* = 3.7 Hz), 57.9, 46.1, 42.8; **^{19}F NMR (471 MHz, CDCl$_3$)** δ -105.8, -106.1; **HRMS** (ESI/TOF-Q) m/z: $[M+H]^+$ Calculated for $C_{27}H_{23}F_2N_3O_2SH^+$ 492.1552; Found 492.1575.

N-(2-((bis(4-chlorophenyl)(oxo)-l6-sulfaneylidene)amino)-3-phenylpropyl)-picolinamide (4i):

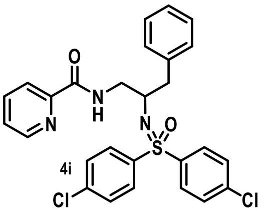

4i

Compound **4i** was synthesized according to GP-1 as sticky colourless liquid; eluent (30% ethyl acetate in hexane); **Yield:** 68% (71 mg); **^{1}H NMR (400 MHz, CDCl$_3$)** δ 8.70 – 8.67(m, 1H), 8.63 (d, *J* = 4.8 Hz, 1H), 8.17 (d, *J* = 7.8 Hz, 1H), 7.87 – 7.81 (m, 3H), 7.46 – 7.43 (m, 1H), 7.35 – 7.27 (m, 5H), 7.25 – 7.21 (m, 4H), 7.16 (d, *J* = 8.5 Hz, 2H), 3.88 – 3.81 (m, 1H), 3.51 – 3.37 (m, 2H), 3.02 – 2.89 (m, 2H); **^{13}C NMR (101 MHz, CDCl$_3$)** δ 164.6, 150.4, 148.3, 139.8, 139.6, 139.5, 139.1, 138.0, 137.4, 130.3, 130.2, 130.1, 129.43, 129.39, 128.4, 126.5, 126.2, 122.5, 57.9, 46.1, 42.8; **HRMS** (ESI/TOF-Q) m/z: $[M+H]^+$ Calculated for $C_{27}H_{23}Cl_2N_3O_2SH^+$ 524.0961; Found 524.0951.

N-(2-((bis(4-bromophenyl)(oxo)-l6-sulfaneylidene)amino)-3-phenylpropyl)-picolinamide (4j):

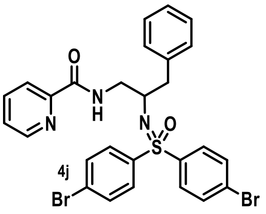

4j

Compound **4j** was synthesized according to GP-1 as brown solid; eluent (20% ethyl acetate in hexane); m.p.: 130 – 132 °C; **Yield:** 65% (79 mg); **^{1}H NMR (500 MHz, CDCl$_3$)** δ 8.68 (t, *J* = 6.4 Hz, 1H), 8.63 (d, *J* = 4.7 Hz, 1H), 8.17 (d, *J* = 7.8 Hz, 1H), 7.88 – 7.84 (m, 1H), 7.75 – 7.73 (m, 2H), 7.50 (d, *J* = 8.5 Hz, 2H), 7.46 – 7.43 (m, 1H), 7.33 – 7.30 (m, 4H), 7.27 (d, *J* = 1.5 Hz, 1H), 7.25 – 7.23 (m, 2H), 7.12 (d, *J* = 8.5 Hz, 2H), 3.87 – 3.82 (m, 1H), 3.50 – 3.44 (m, 1H), 3.42 – 3.38 (m, 1H), 3.01 – 2.98 (m, 1H), 2.94 – 2.89 (m, 1H); **^{13}C NMR (126 MHz, CDCl$_3$)** δ 164.6, 150.4, 148.3, 140.1, 139.7, 138.5, 137.4, 132.42, 132.38, 130.5, 130.24, 130.20, 128.4, 128.1, 127.7, 126.5, 126.2, 122.5, 57.9, 46.1, 42.8; **HRMS** (ESI/TOF-Q) m/z: $[M+H]^+$ Calculated for $C_{27}H_{23}Br_2N_3O_2SH^+$ 611.9950; Found 611.9967.

N-(2-((di(naphthalen-2-yl)(oxo)-l6-sulfaneylidene)amino)-3-phenylpropyl)-picolinamide (4k):

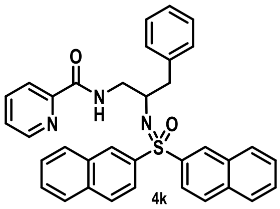

4k

Compound **4k** was synthesized according to GP-1 as sticky brown liquid; eluent (35% ethyl acetate in hexane); **Yield:** 82% (91 mg); **^{1}H NMR (400 MHz, CDCl$_3$)** δ 8.95 – 8.93 (m, 1H), 8.72 (d, *J* = 4.6 Hz, 1H), 8.59 (s, 1H), 8.27 (s, 1H), 8.14 (d, *J* = 7.8 Hz, 1H), 7.90 – 7.85 (m, 3H), 7.80 (d, *J* = 8.0 Hz, 1H), 7.77 (d, *J* = 8.3 Hz, 2H), 7.64 (t, *J* = 8.9 Hz, 2H), 7.59 – 7.53 (m, 3H), 7.50 – 7.46 (m, 2H), 7.38 – 7.35 (m, 2H), 7.32 – 7.30 (m, 3H), 7.26 – 7.24 (m, 1H), 3.94 – 3.89 (m, 1H), 3.54 – 3.48 (m, 2H), 3.05 (d, *J* = 5.9 Hz, 2H); **^{13}C NMR (126 MHz, CDCl$_3$)** δ 164.6, 150.6, 148.4, 139.9, 138.0, 137.3, 136.6, 134.9, 134.7, 132.51, 132.46, 130.6, 130.4, 130.3, 129.4, 129.33, 129.26, 129.2, 128.9, 128.8, 128.4, 127.9, 127.8, 127.4, 127.2, 126.4, 126.1, 124.0, 123.9, 122.5, 58.1, 46.1, 43.1; **HRMS** (ESI/TOF-Q) m/z: $[M+H]^+$ Calculated for $C_{35}H_{29}N_3O_2SH^+$ 556.2053; Found 556.2059.

N-(2-((5-oxido-5l4-dibenzo[b,d]thiophen-5-ylidene)amino)-3-phenylpropyl)-picolinamide (4l):

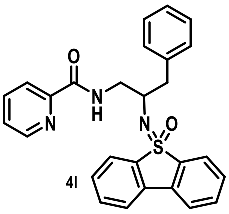

4l

Compound **4l** was synthesized according to GP-1 as brown solid; eluent (45% ethyl acetate in hexane); m.p.: 136 – 138 °C; **Yield:** 79% (72 mg); **^{1}H NMR (400 MHz, CDCl$_3$)** δ 8.46 – 8.42 (m, 2H), 8.13 (d, *J* = 7.8 Hz, 1H), 7.82 – 7.77 (m, 1H), 7.69 – 7.64 (m, 3H), 7.50 (t, *J* = 7.1 Hz, 2H), 7.46 – 7.41 (m, 1H), 7.38 – 7.35 (m, 1H), 7.29 (t, *J* = 7.8 Hz, 2H), 7.18 – 7.11 (m, 5H), 3.99 – 3.92 (m, 1H), 3.77 – 3.71 (m, 1H), 3.37 – 3.31(m, 1H), 2.92 – 2.82 (m, 2H); **^{13}C NMR (101 MHz, CDCl$_3$)** δ 164.5, 150.2, 148.2, 139.6, 139.5, 138.7, 137.2, 132.9, 132.8, 131.9, 131.8, 130.23, 130.20, 129.7, 128.3, 126.3, 125.9, 122.8, 122.5, 122.2, 121.4, 121.3, 56.6, 45.3, 42.7; **HRMS** (ESI/TOF-Q) m/z: $[M+H]^+$ Calculated for $C_{27}H_{23}N_3O_2SH^+$ 454.1584; Found 454.1581.

N-(2-((10-oxido-9-oxo-9H-10l4-thioxanthen-10-ylidene)amino)-3-phenylpropyl)-picolinamide (4m):

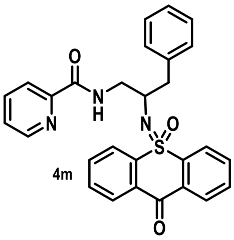

4m

Compound **4m** was synthesized according to GP-1 as off white solid; eluent (45% ethyl acetate in hexane); m.p.: 140 - 142 °C; **Yield:** 72% (69 mg); **^{1}H NMR (400 MHz, CDCl$_3$)** δ 8.60 (d, *J* = 4.8 Hz, 1H), 8.40 – 8.37 (m, 1H), 8.12 – 8.06 (m, 4H), 7.99 (d, *J* = 7.7 Hz, 1H), 7.84 – 7.80 (m, 1H), 7.73 (t, *J* = 7.6 Hz, 1H), 7.69 – 7.66 (m, 1H), 7.61 (t, *J* = 7.5 Hz, 1H), 7.55 (t, *J* = 7.6 Hz, 1H), 7.44 – 7.41 (m, 1H), 7.10 – 7.08 (m, 3H), 6.91 – 6.89 (m, 2H), 3.79 – 3.73 (m, 1H), 3.68 – 3.61 (m, 1H), 3.26 – 3.19 (m, 1H), 2.81 – 2.65 (m, 2H); **^{13}C NMR (101 MHz, CDCl$_3$)** δ 178.9, 164.5, 150.1, 148.3, 141.2, 140.5, 138.5, 137.2, 134.0, 133.7, 132.24, 132.21, 130.42, 130.37, 129.5, 129.3, 129.1, 128.4, 126.3, 126.1, 124.6, 124.4, 122.2, 57.5, 45.4, 42.5; **HRMS** (ESI/TOF-Q) m/z: $[M+H]^+$ Calculated for $C_{28}H_{23}N_3O_3SH^+$ 482.1533; Found 482.1521.

N-(2-((10-oxido-10l4-phenoxathiin-10-ylidene)amino)-3-phenylpropyl)-picolinamide (4n):

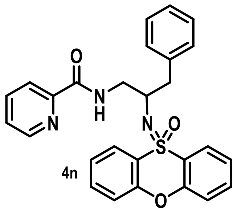

4n

Compound **4n** was synthesized according to GP-1 as sticky colourless liquid; eluent (45% ethyl acetate in hexane); m.p.: 102-104 °C; **Yield:** 75% (70 mg); **^{1}H NMR (400 MHz, CDCl$_3$)** δ 8.59 (d, *J* = 4.9 Hz, 1H), 8.43 – 8.40 (m, 1H), 8.12 (d, *J* = 7.9 Hz, 1H), 7.91 (d, *J* = 8.0, 1.6 Hz, 1H), 7.85 – 7.80 (m, 2H), 7.49 – 7.40 (m, 3H), 7.27 – 7.26 (m, 1H), 7.24 – 7.11 (m, 6H), 7.01 – 6.97 (m, 2H), 3.68 – 3.53 (m, 2H), 3.28 – 3.21 (m, 1H), 2.79 – 2.65 (m, 2H); **^{13}C NMR (101 MHz, CDCl$_3$)** δ 164.4, 151.5, 151.4, 150.3, 148.2, 138.6, 137.2, 133.4, 133.3, 129.5, 128.3, 126.2, 126.0, 125.5, 125.1, 124.7, 124.5, 124.4 (2xC), 122.2, 118.9, 118.7, 56.9, 45.0, 42.4; **HRMS** (ESI/TOF-Q) m/z: $[M+H]^+$ Calculated for $C_{27}H_{23}N_3O_3SH^+$ 470.1533; Found 470.1531.

N-(2-((10-isopropyl-5-oxido-10H-5l4-phenothiazin-5-ylidene)amino)-3-phenylpropyl) picolinamide (4o):

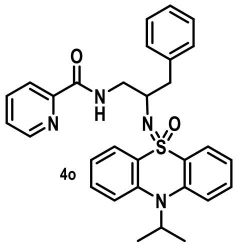

4o

Compound **4o** was synthesized according to GP-1 as sticky colourless liquid; eluent (40% ethyl acetate in hexane); **Yield:** 71% (73 mg); **^{1}H NMR (400 MHz, CDCl$_3$)** δ 8.59 – 8.57 (m, 1H), 8.47 (brs, 1H), δ 8.14 (d, *J* = 10.0 Hz, 1H), 8.07 – 8.04 (m, 1H), 7.99 – 7.97 (m, 1H), 7.83 – 7.79 (m, 1H), 7.53 – 7.44 (m, 2H), 7.41 – 7.35 (m, 3H), 7.23 – 7.18 (m, 2H), 7.15 – 7.07 (m, 3H), 6.90 – 6.87 (m, 2H), 4.48 – 4.41 (m, 1H), 3.64 – 3.50 (m, 2H), 3.33 – 3.27 (m, 1H), 2.72 – 2.66 (m, 1H), 2.58 – 2.53 (m, 1H), 1.70 (d, *J* = 7.0 Hz, 3H), 1.61 (d, *J* = 7.0 Hz, 3H); **^{13}C NMR (101 MHz, CDCl$_3$)** δ 164.5, 150.5, 148.2, 141.7 141.6, 138.9, 137.2, 132.1 (2xC), 129.2, 128.3, 126.1, 126.0, 125.9, 125.3, 125.0, 124.7, 122.3, 122.1, 121.6, 118.2, 117.7, 55.2, 53.5, 45.0, 42.8, 22.1, 21.9; **HRMS** (ESI/TOF-Q) m/z: $[M+H]^+$ Calculated for $C_{30}H_{30}N_4O_2SH^+$ 511.2162; Found 511.2188.

N-(2-((oxo(phenyl)(p-tolyl)-l6-sulfaneylidene)amino)-3-phenylpropyl)-picolinamide (4p):

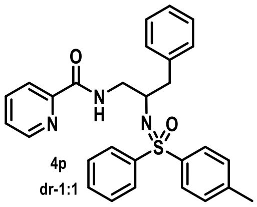

4p
dr-1:1

Compound **4p** was synthesized according to GP-1 as sticky colourless liquid; eluent (30% ethyl acetate in hexane); **Yield:** 86% (81 mg); **^{1}H NMR (400 MHz, CDCl$_3$)** δ 8.81 – 8.78 (m, 2H), 8.65 (d, *J* = 4.8 Hz, 2H), 8.16 (d, *J* = 7.9 Hz, 2H), 7.92 (d, *J* = 7.8 Hz, 2H), 7.86 – 7.80 (m, 4H), 7.46 – 7.41 (m, 5H), 7.39 – 7.28 (m, 10H), 7.25 – 7.21 (m, 7H), 7.16 (d, *J* = 8.0 Hz, 2H), 7.03 (d, *J* = 8.0 Hz, 2H), 3.87 – 3.81 (m, 2H), 3.49 – 3.36 (m, 4H), 2.99 – 2.96 (m, 4H), 2.33 (s, 3H), 2.30 (s, 3H); **^{13}C NMR (101 MHz, CDCl$_3$)** δ 164.5 (2xC), 150.5 (2xC), 148.3 (2xC), 143.4, 143.1, 141.4, 140.1, 139.8, 138.0, 137.3 (2xC), 136.7, 132.4, 132.1, 130.19, 130.17, 129.74, 129.73, 129.0, 128.99, 128.97, 128.8 (3xC), 128.6 (2xC), 128.3 (3xC), 126.3, 126.0, 122.4 (2xC), 57.67, 57.65, 45.91, 45.89, 43.0 (2xC), 21.53, 21.50; **HRMS** (ESI/TOF-Q) m/z: $[M+H]^+$ Calculated for $C_{28}H_{27}N_3O_2SH^+$ 470.1897; Found 470.1883. **dr-1:1**.

N-(2-(((2,6-dimethylphenyl)(oxo)(phenyl)-l6-sulfaneylidene)amino)-3-phenylpropyl)picolinamide (4q):

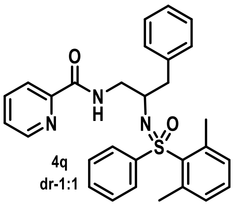

4q
dr-1:1

Compound **4q** was synthesized according to GP-1 as pale yellow liquid; eluent (30% ethyl acetate in hexane); **Yield:** 84% (81 mg); **^{1}H NMR (400 MHz, CDCl$_3$)** δ 8.77 (brs, 1H), 8.58 – 8.55 (m, 3H), 8.16 (t, *J* = 9.2 Hz, 2H), 7.95 (d, *J* = 7.7 Hz, 2H), 7.87 (d, *J* = 7.8 Hz, 2H), 7.83 (t, *J* = 8.0 Hz, 2H), 7.50 – 7.40 (m, 8H), 7.24 – 7.23 (m, 4H), 7.18 – 7.13 (m, 6H), 7.07 – 7.02 (m, 6H), 3.67 – 3.51 (m, 5H), 3.46 –3.42 (m, 1H), 3.11 – 2.98 (m, 2H), 2.90 – 2.79 (m, 2H), 2.50 (s, 6H), 2.33 (s, 6H); **^{13}C NMR (101 MHz, CDCl$_3$)** δ 164.7, 164.4, 150.4, 150.3, 148.3, 148.1, 143.6, 143.5, 140.5, 140.3, 139.3, 139.0, 137.3, 137.2, 136.8 (2xC), 132.10, 132.06, 131.85, 131.84 (2xC), 131.7, 129.7, 129.6, 128.72, 128.69, 128.4, 128.3, 127.51, 127.45, 126.3, 126.2, 126.0, 125.9, 122.3, 122.2, 56.9, 56.7, 45.3, 44.1, 43.3, 42.2, 23.4, 23.1.; **HRMS** (ESI/TOF-Q) m/z: $[M+H]^+$ Calculated for $C_{29}H_{29}N_3O_2SH^+$ 484.2053; Found 484.2060; **dr-1:1**.

N-(2-(([1,1'-biphenyl]-4-yl(oxo)(p-tolyl)-l6-sulfaneylidene)amino)-3-phenylpropyl)picolinamide (4r):

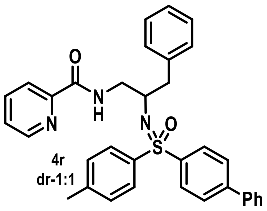

4r
dr-1:1

Compound **4r** was synthesized according to GP-1 as sticky brown liquid; eluent (30% ethyl acetate in hexane); **Yield:** 80% (87 mg); **^{1}H NMR (400 MHz, CDCl$_3$)** δ 8.63 - 8.61 (m, 2H), 8.47 (d, *J* = 4.5 Hz, 2H), 7.99 – 7.97 (m, 2H), 7.78 (d, *J* = 8.3 Hz, 2H), 7.67 – 7.62 (m, 4H), 7.36 (d, *J* = 8.3 Hz, 2H), 7.33 – 7.26 (m, 6H), 7.26 – 7.17 (m, 12H), 7.15 – 7.10 (m, 4H), 7.09 – 7.06 (m, 6H), 6.99 (d, *J* = 8.1 Hz, 2H), 6.86 (d, *J* = 8.0 Hz, 2H), 3.71 – 3.63 (m, 2H), 3.32 – 3.23 (m, 4H), 2.82 – 2.80 (m, 4H), 2.15 (s, 3H), 2.12 (s, 3H); **^{13}C NMR (101 MHz, CDCl$_3$)** δ 164.5 (2xC), 150.5 (2xC), 148.3 (2xC), 145.3, 145.1, 143.4, 143.1, 140.0, 139.82, 139.80, 139.6, 139.5, 138.6 (2xC), 138.2 (2xC), 137.3, 137.2, 136.7, 130.2, 129.8, 129.3, 129.1, 129.05, 129.0, 128.8 (2xC), 128.43, 128.42, 128.3 (2xC), 127.71, 127.68, 127.41, 127.39, 126.3 (2xC), 126.0 (2xC), 122.4 (2xC), 57.73, 57.69, 46.0, 45.9, 43.0, (2xC), 21.54, 21.51; **HRMS** (ESI/TOF-Q) m/z: $[M+H]^+$ Calculated for $C_{34}H_{31}N_3O_2SH^+$ 546.2210; Found 546.2206; **dr-1:1**.

N-(2-(((4-bromophenyl)(oxo)(p-tolyl)-l6-sulfaneylidene)amino)-3-phenylpropyl)-picolinamide (4s):

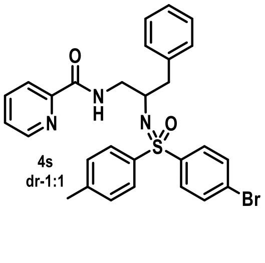

4s
dr-1:1

Compound **4s** was synthesized according to GP-1 as sticky **c**olourless liquid; eluent (30% ethyl acetate in hexane); **Yield:** 66% (72 mg); **^{1}H NMR (500 MHz, CDCl$_3$)** δ 8.82 – 8.75 (m, 2H), 8.67 (d, *J* = 4.6 Hz, 2H), 8.19 (t, *J* = 7.2 Hz, 2H), 7.87 (t, *J* = 7.9 Hz, 2H), 7.81 – 7.77 (m, 4H), 7.50 – 7.45 (m, 4H), 7.34 – 7.33 (m, 6H), 7.29 – 7.28 (m, 8H), 7.21 – 7.19 (m, 4H), 7.06 (d, *J* = 7.9 Hz, 2H), 3.90 – 3.83 (m, 2H), 3.53 – 3.39 (m, 4H), 3.03 – 2.94 (m, 4H), 2.37 (s, 3H), 2.34 (s, 3H); **^{13}C NMR (126 MHz, CDCl$_3$)** δ 164.6, 164.5, 150.5 (2xC), 148.3 (2xC), 143.7, 143.4, 140.7, 139.8, 139.7, 139.3, 139.2, 137.8, 137.3 (2xC), 136.3, 132.24, 132.19, 130.4, 130.23(3xC), 130.18, 129.9 (2xC),128.9, 128.7, 128.3 (2xC), 127.6, 127.3, 126.4, 126.1, 122.4 (2xC), 57.9, 57.7, 46.1, 45.9, 43.0, 42.9, 21.6,

21.5; **HRMS** (ESI/TOF-Q) m/z: [M+H]$^+$ Calculated for $C_{28}H_{26}BrN_3O_2SH^+$ 548.1002; Found 548.1004; **dr-1:1.**

N-(2-((cyclopropyl(oxo)(phenyl)-l6-sulfaneylidene)amino)-3-(4-ethoxyphenyl) propyl)picolinamide (4t):

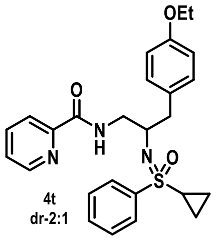

4t
dr-2:1

Compound **4t** was synthesized according to GP-1 as sticky **c**olourless liquid; eluent (40% ethyl acetate in hexane); **Yield:** 56% (52 mg); **1H NMR (400 MHz, CDCl$_3$)** δ 8.70 (brs, 1H), 8.68 – 8.52 (m, 5H), 8.18 – 8.13 (m, 3H), 7.85 – 7.83 (m, 6H), 7.50 – 7.39 (m, 6H), 7.38 – 7.29 (m, 9H) 7.22 – 7.15 (m, 4H), 7.10 – 7.03 (m, 2H), 6.93 – 6.79 (m, 6H), 4.06 – 3.95 (m, 6H), 3.76 – 3.68 (m, 3H), 3.66 – 3.61 (m, 2H), 3.42 – 3.36 (m, 1H) 3.30 – 3.22 (m, 3H), 2.94 – 2.84 (m, 4H), 2.79 – 2.69 (m, 2H), 2.48 – 2.45 (m, 1H), 2.05 – 2.02 (m, 2H), 1.47 – 1.42 (m, 6H) 1.40 – 1.36 (m, 6H), 1.09 – 0.99 (m, 4H), 0.93 – 0.91(m, 2H), 0.79 – 0.66 (m, 3H); **13C NMR (101 MHz, CDCl$_3$)** δ 164.5, 164.3, 157.5 (2xC), 150.5, 150.4, 148.3 (2xC), 140.2, 139.3, 137.3, 137.2, 132.6, 132.4, 131.7, 131.3, 130.9, 130.8, 129.03, 128.98, 128.7, 128.5, 126.0 (2xC), 122.3 (2xC), 114.2 (2xC), 63.5, 63.45, 57.4, 56.5, 45.9, 45.1, 42.3, 41.7, 33.1, 31.9, 22,7, 15.0, 14.2, 6.7, 6.9, 4.9; **HRMS** (ESI/TOF-Q) m/z: [M+H]$^+$ Calculated for $C_{26}H_{29}N_3O_3SH^+$ 464.2002; Found 464.1997; **dr-2:1.**

N-(2-((oxodiphenyl-l6-sulfaneylidene)amino)-3-(p-tolyl)propyl)picolinamide (5a):

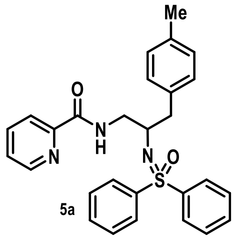

5a

Compound **5a** was synthesized according to GP-1 as off white solid; eluent (30% ethyl acetate in hexane); m.p.: 138 – 140 °C; **Yield:** 82% (77 mg); **^{1}H NMR (400 MHz, CDCl$_3$)** δ 8.69 – 8.66 (m, 1H), 8.55 (d, J = 4.8 Hz, 1H), 8.06 (d, J = 7.8 Hz, 1H), 7.87 – 7.85 (m, 2H), 7.76 – 7.72 (m, 1H), 7.40 (d, J = 8.0 Hz, 2H), 7.37 – 7.28 (m, 5H), 7.18 – 7.13 (m, 2H),7.04 –7.00 (m, 4H), 3.76 – 3.70 (m, 1H), 3.40 – 3.30 (m, 2H), 2.86 (d, J = 6.6 Hz, 2H), 2.25 (s, 3H); **^{13}C NMR (101 MHz, CDCl$_3$)** δ 164.5, 150.5, 148.3, 141.1, 140.0, 137.2, 136.5, 135.7, 132.5, 132.3, 130.0, 129.04, 129.00, 128.95, 128.9, 128.7, 126.0, 122.4, 57.6, 45.8, 42.6, 21.2; **HRMS** (ESI/TOF-Q) m/z: [M+H]$^+$ Calculated for $C_{28}H_{27}N_3O_2SH^+$ 470.1897 ; Found 470.1896.

N-(3-([1,1'-biphenyl]-4-yl)-2-((oxodiphenyl-l6-sulfaneylidene)-amino)propyl) picolinamide (5b):

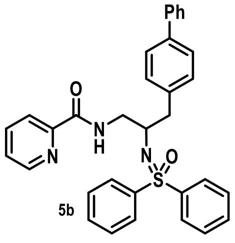

5b

Compound **5b** was synthesized according to GP-1 as brown solid; eluent (30% ethyl acetate in hexane); m.p.: 126 – 128 °C; **Yield:** 80% (85 mg); **^{1}H NMR (400 MHz, CDCl$_3$)** δ 8.81 (t, J = 6.2 Hz, 1H), 8.65 (d, J = 4.8 Hz, 1H), 8.18 (d, J = 7.8 Hz, 1H), 7.98 – 7.95 (m, 2H), 7.87–7.82 (m, 1H), 7.63 – 7.60 (m, 2H), 7.54 (d, J = 7.9 Hz, 2H), 7.48 – 7.43 (m, 6H), 7.38 (t, J = 7.8 Hz, 3H), 7.34 – 7.31 (m, 3H), 7.22 (t, J = 7.7 Hz, 2H), δ 3.91 – 3.85 (m, 1H), 3.56 – 3.43 (m, 2H), 3.03 (d, J = 6.5 Hz, 2H); **^{13}C NMR (101 MHz, CDCl$_3$)** δ 164.6, 150.4, 148.3, 141.2, 141.0, 139.8, 139.2, 138.9, 137.3, 132.6, 132.3, 130.6, 129.1, 129.0, 128.89, 128.86, 128.7, 127.2, 127.1, 126.9, 126.0, 122.4, 57.6, 46.0, 42.6; **HRMS** (ESI/TOF-Q) m/z: [M+H]$^+$ Calculated for $C_{33}H_{29}N_3O_2S_2H^+$ 532.2053; Found 532.2049.

N-(3-(4-ethoxyphenyl)-2-((oxodiphenyl-l6-sulfaneylidene)-amino)propyl)-picolinamide (5c):

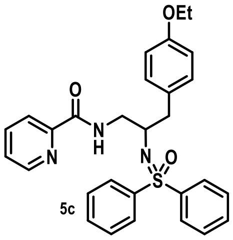

5c

Compound **5c** was synthesized according to GP-1 as sticky colourless liquid; eluent (40% ethyl acetate in hexane); **Yield:** 89% (89 mg); **^{1}H NMR (400 MHz, CDCl3)** δ 8.78 – 8.75 (m, 1H), 8.63 (d, *J* = 4.9 Hz, 1H), 8.15 (d, *J* = 7.8 Hz, 1H), 7.94 (d, *J* = 7.6 Hz, 2H), 7.84 – 7.80 (m, 1H), 7.48 (d, *J* = 8.0 Hz, 2H), 7.44 – 7.34 (m, 5H), 7.26 – 7.22 (m, 2H), 7.12 (d, *J* = 8.3 Hz, 2H), 6.83 (d, *J* = 8.3 Hz, 2H), 4.02 (q, *J* = 7.0 Hz, 2H), 3.84 – 3.78 (m, 1H), 3.47 – 3.35 (m, 2H), 2.90 (d, *J* = 6.7 Hz, 2H), 1.40 (t, *J* = 7.0 Hz, 3H); **^{13}C NMR (101 MHz, CDCl$_3$)** δ 164.5, 157.6, 150.5, 148.3, 141.1, 139.9, 137.2, 132.5, 132.3, 131.6, 131.0, 129.04, 129.00, 128.9, 128.7, 126.0, 122.3, 114.3, 63.6, 57.7, 45.8, 42.1, 15.0; **HRMS** (ESI/TOF-Q) m/z: $[M+H]^+$ Calculated for $C_{29}H_{29}N_3O_3SH^+$ 500.2002; Found 500.2034.

N-(3-(4-(benzyloxy)phenyl)-2-((oxodiphenyl-l6-sulfaneylidene)amino)propyl) picolinamide (5d):

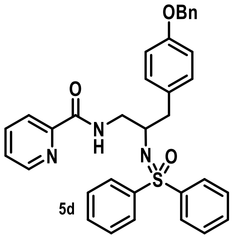

5d

Compound **5d** was synthesized according to GP-1 as sticky colourless liquid; eluent (35% ethyl acetate in hexane); **Yield:** 81% (91 mg); **^{1}H NMR (400 MHz, CDCl$_3$)** δ 8.80 – 8.77 (m, 1H), 8.65 – 8.63 (m, 1H), 8.17 (d, *J* = 7.8 Hz, 1H), 7.96 – 7.93 (m, 2H), 7.85 – 7.81 (m, 1H), 7.49 – 7.42 (m, 6H), 7.38 – 7.34 (m, 5H), 7.31 – 7.29 (m, 1H), 7.22 (t, *J* = 7.7 Hz, 2H), 7.17 – 7.14 (m, 2H), 6.94 – 6.92 (m, 2H), 5.08 (s, 2H), 3.86 – 3.80 (m, 1H), 3.49 – 3.43 (m, 1H), 3.40 – 3.35 (m, 1H), 2.93 (d, *J* = 6.7 Hz, 2H); **^{13}C NMR (101 MHz, CDCl$_3$)** δ 164.5, 157.5, 150.5, 148.3, 141.1, 139.9, 137.4, 137.2, 132.5, 132.3, 132.1, 131.0, 129.05, 129.00, 128.9, 128.73, 128.68, 128.0, 127.5, 126.0, 122.4, 114.8, 70.2, 57.7, 45.8, 42.1; **HRMS** (ESI/TOF-Q) m/z: $[M+H]^+$ Calculated for $C_{34}H_{31}N_3O_3SH^+$ 562.2159; Found 562.2155.

N-(3-(4-(allyloxy)phenyl)-2-((oxodiphenyl-l6-sulfaneylidene)-amino)propyl)-picolinamide (5e):

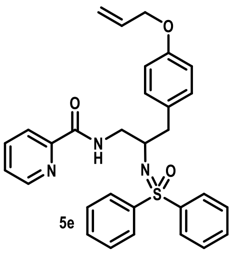

5e

Compound **5e** was synthesized according to GP-1 as pale yellow liquid; eluent (35% ethyl acetate in hexane); **Yield:** 78% (80 mg); **^{1}H NMR (400 MHz, CDCl$_3$)** δ 8.78 – 8.75 (m, 1H), 8.63 – 8.61 (m, 1H), 8.14 (d, *J* = 7.8 Hz, 1H), 7.94 – 7.92 (m, 2H), 7.83 – 7.79 (m, 1H), 7.49 – 7.45 (m, 2H), 7.44 – 7.33 (m, 5H), 7.25 – 7.22 (m, 2H), 7.14 – 7.11 (m, 2H), 6.87 – 6.82 (m, 2H), 6.09 – 6.00 (m, 1H), 5.42 – 5.37 (m, 1H), 5.27 – 5.23 (m, 1H), 4.53 – 4.51 (m, 2H), 3.84 – 3.78 (m, 1H), 3.47 – 3.34 (m, 2H), 2.90 (d, *J* = 6.6 Hz, 2H); **^{13}C NMR (126 MHz, CDCl$_3$)** δ 164.5, 157.3, 150.5, 148.3, 141.1, 139.9, 137.2, 133.6, 132.5, 132.3, 132.0, 131.0, 129.04, 129.0, 128.9, 128.7, 126.0, 122.3, 117.7, 114.6, 69.0, 57.7, 45.7, 42.1; **HRMS** (ESI/TOF-Q) m/z: $[M+H]^+$ Calculated for $C_{30}H_{29}N_3O_3SH^+$ 512.2002; Found 512.1999.

N-(3-(4-(methylthio)phenyl)-2-((oxodiphenyl-l6-sulfaneylidene)-amino)propyl)-picolinamide (5f):

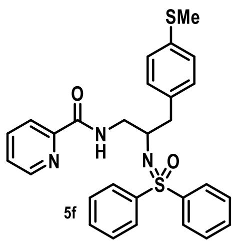

Compound **5f** was synthesized according to GP-1 as Pale yellow solid; eluent (35% ethyl acetate in hexane); m.p.: 128 – 130 °C; **Yield:** 85% (85 mg); **^{1}H NMR (400 MHz, $CDCl_3$)** δ 8.80 – 8.77 (m, 1H), 8.66 (d, *J* = 4.8 Hz, 1H), 8.19 (d, *J* = 7.8 Hz, 1H), 7.97 – 7.95 (m, 2H), 7.88 – 7.84 (m, 1H), 7.52 – 7.50 (m, 2H), 7.46 – 7.38 (m, 5H), 7.30 – 7.28 (m, 2H), 7.24 – 7.22 (m, 2H), 7.19 – 7.17 (m, 2H), 3.87 – 3.81 (m, 1H), 3.53 – 3.39 (m, 2H), 2.96 (d, *J* = 6.6 Hz, 2H), 2.50 (s, 3H); **^{13}C NMR (101 MHz, $CDCl_3$)** δ 164.5, 150.4, 148.3, 141.0, 139.9, 137.3, 136.8, 135.9, 132.5, 132.3, 130.6, 129.1, 129.0, 128.8, 128.6, 127.0, 126.0, 122.3, 57.5, 45.8, 42.4, 16.4; **HRMS** (ESI/TOF-Q) m/z: $[M+H]^+$ Calculated for $C_{28}H_{27}N_3O_2S_2H^+$ 502.1617; Found 502.1639.

N-(3-(4-fluorophenyl)-2-((oxodiphenyl-l6-sulfaneylidene)-amino)propyl)-picolinamide (5g):

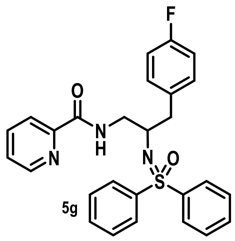

Compound **5g** was synthesized according to GP-1 as sticky colourless liquid; eluent (40% ethyl acetate in hexane); **Yield:** 70% (66 mg); **^{1}H NMR (500 MHz, $CDCl_3$)** δ 8.75 (t, *J* = 6.2 Hz, 1H), 8.65 – 8.63 (m, 1H), 8.17 – 8.15 (m, 1H), 7.93 – 7.91 (m, 2H), 7.86 – 7.82 (m, 1H), 7.52 – 7.50 (m, 2H), 7.48 – 7.43 (m, 2H), 7.42 – 7.36 (m, 3H), 7.29 – 7.26 (m, 2H), 7.20 – 7.17 (m, 2H), 6.97 (t, *J* = 8.8 Hz, 2H), 3.83 – 3.79 (m, 1H), 3.49 – 3.44 (m, 1H), 3.42 – 3.37 (m, 1H), 2.95 – 2.94 (m, 2H); **^{13}C NMR (126 MHz, $CDCl_3$)** δ 164.5, 161.8 (d, *J* = 243.8 Hz), 150.5, 148.3, 141.1, 140.2, 137.3, 135.4 (d, *J* = 3.5 Hz), 132.5 (d, *J* = 27.3 Hz), 131.5 (d, *J* = 7.7 Hz), 129.11, 129.05, 128.8, 128.7, 126.1, 122.4, 115.1, 114.9, 57.5, 45.8, 42.2; **^{19}F NMR (471 MHz, $CDCl_3$)** δ -117.3; **HRMS** (ESI/TOF-Q) m/z: $[M+H]^+$ Calculated for $C_{27}H_{24}FN_3O_2SH^+$ 474.1646; Found 474.1675.

N-(3-(4-chlorophenyl)-2-((oxodiphenyl-l6-sulfaneylidene)-amino)propyl)-picolinamide (5h):

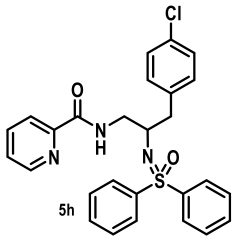

Compound **5h** was synthesized according to GP-1 as sticky brown liquid; eluent (40% ethyl acetate in hexane); **Yield:** 76% (74 mg); **^{1}H NMR (400 MHz, $CDCl_3$)** δ 8.75 (brs, 1H), 8.64 (d, *J* = 4.8 Hz, 1H), 8.16 (d, *J* = 7.8 Hz, 1H), 7.93 – 7.91 (m, 2H), 7.87 – 7.82 (m, 1H), 7.49 – 7.36 (m, 8H), 7.26 – 7.24 (m, 3H), 7.17 – 7.14 (m, 2H), 3.84 – 3.78 (m, 1H), 3.50 – 3.37 (m, 2H), 2.95 – 2.93 (m, 2H); **^{13}C NMR (101 MHz, $CDCl_3$)** δ 164.6, 150.4, 148.3, 140.9, 139.9, 138.2, 137.3, 132.6, 132.4, 132.1, 131.5, 129.13, 129.07, 128.8, 128.7, 128.4, 126.1, 122.4, 57.4, 45.9, 42.3; **HRMS** (ESI/TOF-Q) m/z: $[M+H]^+$ Calculated for $C_{27}H_{24}ClN_3O_2SH^+$ 490.1351; Found 490.1359.

N-(3-(4-bromophenyl)-2-((oxodiphenyl-l6-sulfaneylidene)-amino)propyl) picolinamide (5i):

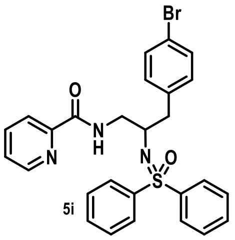

5i

Compound **5i** was synthesized according to GP-1 as sticky colourless liquid; eluent (40% ethyl acetate in hexane). **Yield:** 68% (72 mg); **^{1}H NMR (500 MHz, CDCl$_3$)** δ 8.72 (t, *J* = 6.3 Hz, 1H), 8.61 (d, *J* = 4.7 Hz, 1H), 8.14 (d, *J* = 7.8 Hz, 1H), 7.90 (d, *J* = 7.8 Hz, 2H), 7.83 – 7.80 (m, 1H), 7.46 – 7.43 (m, 3H), 7.42 – 7.34 (m, 6H), 7.24 – 7.23 (m, 2H), 7.08 (d, *J* = 8.0 Hz, 2H), 3.81 – 3.76 (m, 1H), 3.48 – 3.43 (m, 1H), 3.40 – 3.35 (m, 1H), 2.93 – 2.86 (m, 2H); **^{13}C NMR (126 MHz, CDCl$_3$)** δ 164.5, 150.4, 148.3, 140.9, 139.9, 138.8, 137.3, 132.6, 132.4, 131.9, 131.3, 129.12, 129.06, 128.7, 128.6, 126.1, 122.4, 120.1, 57.3, 45.9, 42.3; **HRMS** (ESI/TOF-Q) m/z: [M+H]$^+$ Calculated for $C_{27}H_{24}BrN_3O_2SH^+$ 534.0845; Found 534.0847.

N-(2-((oxodiphenyl-l6-sulfaneylidene)amino)-3-(m-tolyl)propyl)picolinamide (5j):

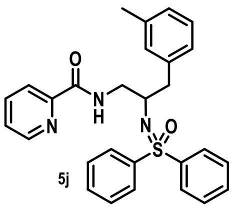

5j

Compound **5j** was synthesized according to GP-1 as sticky colourless liquid; eluent (30% ethyl acetate in hexane); **Yield:** 84% (79 mg); **^{1}H NMR (400 MHz, CDCl$_3$)** δ 8.78 (brs, 1H), 8.64 (d, *J* = 5.1 Hz, 1H), 8.16 (d, *J* = 7.6 Hz, 1H), 7.95 (d, *J* = 7.6 Hz, 2H), 7.85 – 7.81 (m, 1H), 7.47 – 7.35 (m, 7H), 7.23 – 7.20 (m, 3H), 7.05 (brs, 3H), 3.88 – 3.82 (m, 1H), 3.51 – 3.37 (m, 2H), 2.96 – 2.93 (m, 2H), 2.34 (s, 3H); **^{13}C NMR (101 MHz, CDCl$_3$)** δ 164.5, 150.6, 148.3, 141.3, 139.8, 139.7, 137.7, 137.2, 132.5, 132.2, 131.0, 129.01, 128.98, 128.94, 128.8, 128.2, 127.2, 127.0, 126.0, 122.4, 57.8, 46.1, 42.9, 21.5; **HRMS** (ESI/TOF-Q) m/z: [M+H]$^+$ Calculated for $C_{28}H_{27}N_3O_2SH^+$ 470.1897; Found 470.1929.

N-(3-(3-methoxyphenyl)-2-((oxodiphenyl-l6-sulfaneylidene)-amino)propyl) picolinamide (5k):

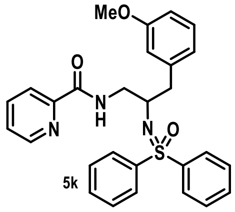

5k

Compound **5k** was synthesized according to GP-1 as sticky colourless liquid; eluent (40% ethyl acetate in hexane); **Yield:** 88% (85 mg); **^{1}H NMR (400 MHz, CDCl$_3$)** δ 8.80 – 8.77 (m, 1H), 8.65 (d, *J* = 4.7 Hz, 1H), 8.16 (d, *J* = 7.8 Hz, 1H), 7.96 – 7.94 (m, 2H), 7.86 – 7.82 (m, 1H), 7.48 – 7.35 (m, 7H), 7.23 – 7.20 (m, 3H), 6.85 (d, *J* = 7.5 Hz, 1H), 6.81 – 6.76 (m, 2H), 3.88 – 3.83 (m, 1H), 3.77 (s, 3H), 3.50 – 3.37 (m, 2H), 2.96 – 2.94 (m, 2H); **^{13}C NMR (101 MHz, CDCl$_3$)** δ 164.6, 159.7, 150.6, 148.4, 141.3, 141.2, 139.8, 137.3, 132.6, 132.3, 129.2, 129.1 (2xC), 129.0, 128.8, 126.0, 122.6, 122.4, 115.4, 112.2, 57.7, 55.3, 46.0, 43.1; **HRMS** (ESI/TOF-Q) m/z: [M+H]$^+$ Calculated for $C_{28}H_{27}N_3O_3SH^+$ 486.1846; Found 486.1846.

N-(3-(3-fluorophenyl)-2-((oxodiphenyl-l6-sulfaneylidene)-amino)propyl) picolinamide (5l):

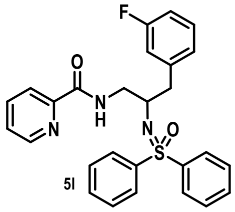

5l

Compound **5l** was synthesized according to GP-1 as sticky colourless liquid; eluent (40% ethyl acetate in hexane); m.p.: 98 – 100 °C; **Yield:** 68% (64 mg); **^{1}H NMR (500 MHz, CDCl$_3$)** δ 8.79 (brs, 1H), 8.66 – 8.65 (m, 1H), 8.18 (d, *J* = 7.8 Hz, 1H), 7.94 (d, *J* = 7.8 Hz, 2H), 7.85 (t, *J* = 7.8 Hz, 1H), 7.49 – 7.37 (m, 7H), 7.29 – 7.27 (m, 3H), 7.04 (d, *J* = 7.6

Hz, 1H), 6.96 – 6.93 (m, 2H). 3.88 – 3.83 (m, 1H), 3.51 – 3.46 (m, 1H), 3.44 – 3.40 (m, 1H), 2.98 (d, *J* = 6.7 Hz, 2H). **13C NMR (126 MHz, CDCl3)** δ 164.5, 162.9 (d, *J* = 245.1 Hz), 150.4, 148.3, 142.3 (d, *J* = 7.3 Hz), 140.9, 139.7, 137.3, 132.5 (d, *J* = 29.7 Hz), 129.6 (d, *J* = 8.2 Hz), 129.1, 129.0, 128.8, 128.7, 126.1, 125.8 (d, *J* = 2.8 Hz), 122.4, 117.0, 116.8, 113.1 (d, *J* = 21.0 Hz), 57.4, 45.9, 42.6; **^{19}F NMR (471 MHz, CDCl$_3$)** δ -114.1; **HRMS** (ESI/TOF-Q) m/z: $[M+H]^+$ Calculated for $C_{27}H_{24}FN_3O_2SH^+$474.1646; Found 474.1648.

N-(3-(3-chlorophenyl)-2-((oxodiphenyl-l6-sulfaneylidene)-amino)propyl) picolinamide (5m):

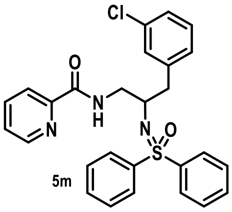

5m

Compound **5m** was synthesized according to GP-1 as white solid; eluent (30% ethyl acetate in hexane); m.p.: 134 – 136 °C; **Yield:** 70% (69 mg); **^{1}H NMR (500 MHz, CDCl$_3$)** δ 8.84 – 8.82 (m, 1H), 8.69 – 8.68 (m, 1H), 8.20 (d, *J* = 7.8 Hz, 1H), 7.96 (d, *J* = 7.7 Hz, 2H), 7.87 (t, *J* = 7.8 Hz, 1H), 7.52 – 7.38 (m, 7H), 7.30 – 7.27 (m, 5H), 7.18 – 7.16 (m, 1H), 3.92 – 3.87 (m, 1H), 3.54 – 3.49 (m, 1H), 3.43 – 3.38 (m, 1H), 3.02 – 2.93 (m, 2H); **^{13}C NMR (126 MHz, CDCl$_3$)** δ 164.6, 150.4, 148.3, 142.0, 141.0, 139.5, 137.3, 134.0, 132.6, 132.3, 130.4, 129.5, 129.1, 129.0, 128.8, 128.7, 128.4, 126.4, 126.1, 122.4, 57.6, 46.1, 42.5; **HRMS** (ESI/TOF-Q) m/z: $[M+H]^+$ Calculated for $C_{27}H_{24}ClN_3O_2SH^+$490.1351; Found 490.1367.

N-(2-((oxodiphenyl-l6-sulfaneylidene)amino)-3-(3-(trifluoromethyl)phenyl)-propyl)picolinamide (5n):

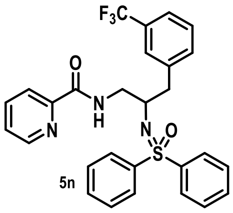

5n

Compound **5n** was synthesized according to GP-1 as sticky colourless liquid; eluent (40% ethyl acetate in hexane); **Yield:** 65% (68 mg); **^{1}H NMR (500 MHz, CDCl$_3$)** δ 8.82 – 8.79 (m, 1H), 8.65 – 8.64 (m, 1H), 8.15 (d, *J* = 7.8 Hz, 1H), 7.89 (d, *J* = 7.7 Hz, 2H), 7.83 (t, *J* = 7.7 Hz, 1H), 7.56 (s, 1H), 7.52 – 7.51 (m, 1H), 7.46 – 7.41 (m, 4H), 7.38 – 7.34 (m, 3H), 7.31 – 7.30 (m, 2H), 7.18 (t, *J* = 7.6 Hz, 2H), 3.90 – 3.85 (m, 1H), 3.54 – 3.49 (m, 1H), 3.42 – 3.37 (m, 1H), 3.06 – 2.97 (m, 2H); **^{13}C NMR (126 MHz, CDCl$_3$)** δ 164.6, 150.3, 148.3, 140.95, 140.88, 139.5, 137.3, 133.6, 132.7, 132.3, 130.4 (q, *J* = 31.8 Hz), 129.1, 129.0, 128.64 (2xC), 128.57, 127.0 (q, *J* = 3.9 Hz), 126.1, 124.4 (q, *J* = 272.2 Hz), 123.1 (q, *J* = 3.8 Hz), 122.4, 57.6, 46.2, 42.5; **^{19}F NMR (471 MHz, CDCl$_3$)** δ -62.3; **HRMS** (ESI/TOF-Q) m/z: $[M+H]^+$ Calculated for $C_{28}H_{24}F_3N_3O_2SH^+$ 524.1614; Found 524.1620

N-(3-(3,5-dimethylphenyl)-2-((oxodiphenyl-l6-sulfaneylidene)amino)-propyl)picolinamide (5o):

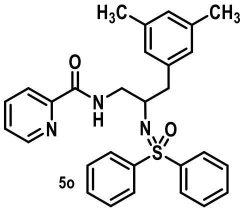

5o

Compound **5o** was synthesized according to GP-1 as sticky brown liquid; eluent (30% ethyl acetate in hexane); **Yield:** 85% (82 mg); **^{1}H NMR (400 MHz, CDCl$_3$)** δ 8.81 – 8.78 (m, 1H), 8.66 (d, *J* = 4.8 Hz, 1H), 8.16 (d, *J* = 7.8 Hz, 1H), 7.96 (d, *J* = 7.9 Hz, 2H), 7.86 – 7.82 (m, 1H), 7.46 – 7.42 (m, 2H), 7.40–7.36 (m, 3H), 7.31 (d, *J* = 7.7 Hz, 2H), 7.19 (t, *J* = 7.6 Hz, 2H), 6.89 – 6.87 (m, 3H), 3.91 – 3.85 (m, 1H), 3.48 – 3.41 (m, 1H), 3.38 – 3.31 (m, 1H), 2.94 – 2.88 (m, 2H), 2.32 (s, 6H); **^{13}C NMR (101 MHz, CDCl$_3$)** δ 164.6, 150.6, 148.4, 141.4, 139.7, 139.6, 137.6, 137.2, 132.5, 132.2, 129.1, 129.0, 128.9, 128.8, 128.1, 127.8, 126.0, 122.4, 58.0, 46.2, 42.8, 21.4; **HRMS** (ESI/TOF-Q) m/z: $[M+H]^+$ Calculated for $C_{29}H_{29}N_3O_2SH^+$ 484.2053; Found 484.2036.

N-(3-(4-acetylphenyl)-2-((oxodiphenyl-l6-sulfaneylidene)amino)-propyl) picolinamide (5p):

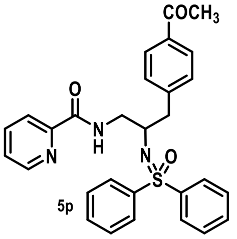

5p

Compound **5p** was synthesized according to GP-1 as sticky pale yellow liquid; eluent (35% ethyl acetate in hexane); **Yield:** 71% (71 mg); **^{1}H NMR (400 MHz, CDCl$_3$)** δ 8.74 (brs, 1H), 8.63 (d, *J* = 4.8 Hz, 1H), 8.16 (d, *J* = 7.8 Hz, 1H), 7.91 – 7.82 (m, 5H), 7.51 – 7.48 (m, 2H), 7.46 – 7.43 (m, 2H), 7.41 – 7.31 (m, 5H), 7.25 – 7.23 (m, 2H), 3.84 – 3.77 (m, 1H), 3.51 – 3.44 (m, 2H), 3.03 (d, *J* = 5.9 Hz, 2H), 2.59 (s, 3H); **^{13}C NMR (101 MHz, CDCl$_3$)** δ 198.1, 164.5, 150.4, 148.3, 145.6, 140.9, 140.0, 137.3, 135.5, 132.7, 132.4, 130.3, 129.2, 129.1, 128.72, 128.67, 128.4, 126.1, 122.4, 57.1, 45.9, 43.0, 26.7; **HRMS** (ESI/TOF-Q) m/z: $[M+H]^+$ Calculated for $C_{29}H_{27}N_3O_3SH^+$ 498.1846 ; Found 498.1845.

Methyl-4-(2-((oxodiphenyl-l6-sulfaneylidene)amino)-3-(picolinamido)propyl)-benzoate (5q):

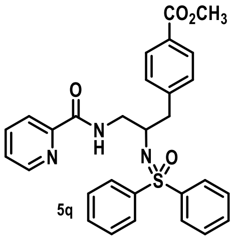

5q

Compound **5q** was synthesized according to GP-1 as white solid; eluent (40% ethyl acetate in hexane); m.p.: 106 – 108 °C; **Yield:** 70% (72 mg); **^{1}H NMR (400 MHz, CDCl$_3$)** δ 8.77 – 8.74 (m, 1H), 8.62 (d, *J* = 4.8 Hz, 1H), 8.15 (d, *J* = 7.8 Hz, 1H), 7.96 (d, *J* = 8.2 Hz, 2H), 7.90 – 7.88 (m, 2H), 7.85 – 7.80 (m, 1H), 7.47 – 7.39 (m, 4H), 7.38 – 7.34 (m, 3H), 7.31 – 7.29 (m, 2H), 7.21 (t, *J* = 7.6 Hz, 2H), 3.90 (s, 3H), 3.85 – 3.80 (m, 1H), 3.52 – 3.41 (m, 2H), 3.02 (d, *J* = 6.3 Hz, 2H); **^{13}C NMR (101 MHz, CDCl$_3$)** δ 167.3, 164.5, 150.3, 148.3, 145.4, 140.9, 139.7, 137.3, 132.6, 132.4, 130.2, 129.6, 129.1, 129.0, 128.7, 128.6, 128.2, 126.1, 122.4, 57.3, 52.1, 46.0, 42.9; **HRMS** (ESI/TOF-Q) m/z: $[M+H]^+$ Calculated for $C_{29}H_{27}N_3O_4SH^+$ 514.1795 ; Found 514.1800.

N-(3-(4-cyanophenyl)-2-((oxodiphenyl-l6-sulfaneylidene)amino)-propyl)-picolinamide (5r):

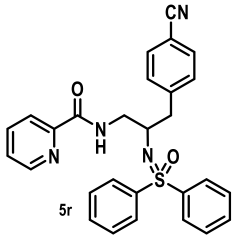

Compound **5r** was synthesized according to GP-1 as off white solid; eluent (40% ethyl acetate in hexane); m.p.: 108 – 110 °C; **Yield:** 69% (66 mg); **^{1}H NMR (400 MHz, CDCl$_3$)** δ 8.72 (brs, 1H), 8.63 – 8.62 (m, 1H), 8.15 (d, *J* = 7.9 Hz, 1H), 7.88 – 7.83 (m, 2H), 7.54 (t, *J* = 9.7 Hz, 4H), 7.47 – 7.41 (m, 4H), 7.38 – 7.29 (m, 6H), 3.80 – 3.74 (m, 1H), 3.53 – 3.45 (m, 2H), 3.03 – 3.01 (m, 2H); **^{13}C NMR (101 MHz, CDCl$_3$)** δ 164.5, 150.2, 148.3, 145.4, 140.6, 140.0, 137.4, 132.7, 132.5, 132.0, 130.9, 129.2, 129.1, 128.6, 128.5, 126.2, 122.4, 119.2, 110.1, 56.9, 45.7, 43.0; **HRMS** (ESI/TOF-Q) m/z: $[M+Na]^+$ Calculated for $C_{28}H_{24}N_4O_2SNa^+$ 503.1512; Found 503.1530.

N-(3-(4-nitrophenyl)-2-((oxodiphenyl-l6-sulfaneylidene)amino)propyl)-picolinamide (5s):

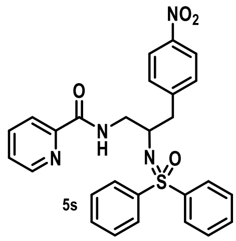

Compound **5s** was synthesized according to GP-1 as sticky yellow liquid; eluent (40% ethyl acetate in hexane); **Yield:** 67% (67 mg); **^{1}H NMR (400 MHz, CDCl$_3$)** δ 8.66 – 8.63 (m, 1H), 8.54 (d, *J* = 4.8 Hz, 1H), 8.08 – 8.02 (m, 3H), 7.81 – 7.74 (m, 3H), 7.46 (d, *J* = 7.8 Hz, 2H), 7.41 – 7.35 (m, 3H), 7.33 – 7.28 (m, 4H), 7.22 – 7.18 (m, 2H), 3.75 – 3.69 (m, 1H), 3.46 – 3.38 (m, 2H), 3.02 – 2.98 (m, 2H); **^{13}C NMR (101 MHz, CDCl$_3$)** δ 164.5, 150.2, 148.3, 147.6, 146.7, 140.5, 140.0, 137.4, 132.8, 132.6, 130.9, 129.2, 129.1, 128.6, 128.5, 126.2, 123.4, 122.4, 56.8, 45.8, 42.7; **HRMS** (ESI/TOF-Q) m/z: $[M+H]^+$ Calculated for $C_{27}H_{24}N_4O_4SH^+$ 501.1591; Found 501.1610.

N-(2-((bis(4-methoxyphenyl)(oxo)-l6-sulfaneylidene)amino)-3-(4-ethoxyphenyl)-propyl)picolinamide (5t):

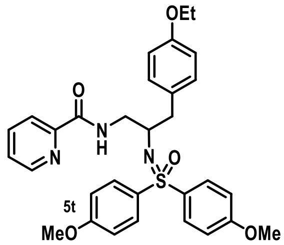

Compound **5t** was synthesized according to GP-1 as sticky colourless liquid; eluent (40% ethyl acetate in hexane); **Yield:** 90% (101 mg); **^{1}H NMR (400 MHz, CDCl$_3$)** δ 8.78 – 8.75 (m, 1H), 8.64 (d, *J* = 4.7 Hz, 1H), 8.16 (d, *J* = 7.9 Hz, 1H), 7.85 – 7.81 (m, 3H), 7.43 – 7.38 (m, 3H), 7.13 (d, *J* = 8.1 Hz, 2H), 6.84 – 6.80 (m, 4H), 6.71 (d, *J* = 8.5 Hz, 2H), 4.02 (q, *J* = 7.0 Hz, 2H), 3.84 – 3.73 (m, 7H), 3.45 – 3.32 (m, 2H), 2.90 (d, *J* = 6.7 Hz, 2H), 1.41 (t, *J* = 7.0 Hz, 3H); **^{13}C NMR (101 MHz, CDCl$_3$)** δ 164.5, 162.8, 162.5, 157.6, 150.6, 148.3, 137.2, 133.2, 131.9, 131.8, 131.0, 130.8, 130.6, 126.0, 122.4, 114.33, 114.26, 114.2, 63.6, 57.6, 55.64, 55.59, 45.8, 42.2, 15.0; **HRMS** (ESI/TOF-Q) m/z$[M+H]^+$ Calculated for $C_{31}H_{33}N_3O_5SH^+$ 560.2214; Found 560.2214.

N-(2-((10-(cyclopropanecarbonyl)-5-oxido-10H-5l4-phenothiazin-5-ylidene)-amino)-3-(4-ethoxyphenyl)propyl)picolinamide (5u):

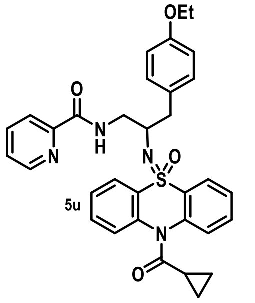

5u

Compound **5u** was synthesized according to GP-1 as brown solid; eluent (40% ethyl acetate in hexane); m.p.: 130 –132 °C; **Yield:** 66% (77 mg); **^{1}H NMR (400 MHz, CDCl$_3$)** δ 8.58 – 8.56 (m, 1H), 8.49 – 8.48 (m, 1H), 8.15 (d, *J* = 7.9 Hz, 1H), 8.06 (d, *J* = 7.8 Hz, 1H), 7.95 (d, *J* = 7.8 Hz, 1H), 7.89 (d, *J* = 8.1 Hz, 1H), 7.82 – 7.79 (m, 2H), 7.59 – 7.54 (m, 2H), 7.45 (t, *J* = 7.8 Hz, 1H), 7.41 – 7.37 (m, 2H), 6.92 (d, *J* = 8.0 Hz, 2H), 6.71 (d, *J* = 8.0 Hz, 2H), 3.95 (q, *J* = 7.1 Hz, 2H), 3.54 – 3.42 (m, 2H), 3.37 – 3.30 (m, 1H), 2.68 – 2.55 (m, 2H), 1.96 – 1.91 (m, 1H), 1.36 (t, *J* = 7.0 Hz, 3H), 1.20 – 1.19 (m, 2H), 0.87 – 0.84 (m, 2H); **^{13}C NMR (101 MHz, CDCl$_3$)** δ 172.3, 164.7, 157.6, 150.6, 148.4, 139.3, 138.9, 137.3, 135.1, 134.0, 132.1, 132.0, 130.6, 127.6, 127.5, 127.2, 126.8, 126.0, 125.8, 125.4, 122.4, 114.6, 63.5, 57.3, 44.3, 41.9, 15.1, 14.4, 11.1, 10.9; **HRMS** (ESI/TOF-Q) m/z: [M+H]$^+$ Calculated for $C_{33}H_{32}N_4O_4SH^+$ 581.2217; Found 581.2214.

N-(3-(naphthalen-2-yl)-2-((oxodiphenyl-l6-sulfaneylidene)amino)propyl)-picolinamide (5v):

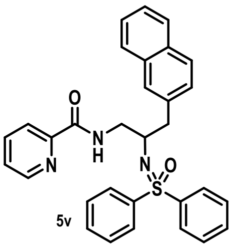

5v

Compound **5v** was synthesized according to GP-1 as brown solid; eluent (35% ethyl acetate in hexane); m.p.: 134 – 136°C; **Yield:** 83% (84 mg); **^{1}H NMR (400 MHz, CDCl$_3$)** δ 8.83 – 8.80 (m, 1H), 8.64 (d, *J* = 4.8 Hz, 1H), 8.16 (d, *J* = 7.8 Hz, 1H), 7.91 (d, *J* = 7.9 Hz, 2H), 7.86 – 7.82 (m, 2H), 7.79 (d, *J* = 8.2 Hz, 2H), 7.70 (s, 1H), 7.49 – 7.38 (m, 5H), 7.33 (t, *J* = 7.7 Hz, 2H), 7.26 – 7.22 (m, 3H), 6.99 (t, *J* = 7.7 Hz, 2H), 3.95 – 3.88 (m, 1H), 3.57 – 3.47 (m, 2H), 3.19 – 3.09 (m, 2H); **^{13}C NMR (101 MHz, CDCl$_3$)** δ 164.6, 150.5, 148.3, 141.1, 139.6, 137.5, 137.3, 133.7, 132.5, 132.3, 132.2, 129.0, 128.88, 128.85, 128.8, 128.7, 128.5, 127.8, 127.7, 127.6, 126.0 (2xC), 125.4, 122.4, 57.9, 46.3, 43.1; **HRMS** (ESI/TOF-Q) m/z: [M+H]$^+$ Calculated for $C_{31}H_{27}N_3O_2SH^+$ 506.1897; Found 506.1914.

N-(2-((oxodiphenyl-l6-sulfaneylidene)amino)-3-(thiophen-2-yl)propyl)-picolinamide (5w):

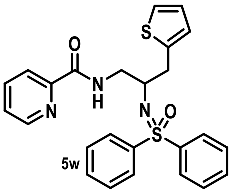

5w

Compound **5w** was synthesized according to GP-1 as off white solid; eluent (30% ethyl acetate in hexane); m.p.: 116 – 118°C; **Yield:** 71% (66 mg); **^{1}H NMR (400 MHz, CDCl$_3$)** δ 8.75 – 8.72 (m, 1H), 8.63 (d, *J* = 4.8 Hz, 1H), 8.16 (d, *J* = 7.8 Hz, 1H), 8.02 – 8.00 (m, 2H), 7.85 – 7.81 (m, 1H), 7.64 (d, *J* = 7.8 Hz, 2H), 7.46 – 7.36 (m, 5H), 7.31 (t, *J* = 7.7 Hz, 2H), 7.16 (d, *J* = 5.1 Hz, 1H), 6.97 – 6.94 (m, 1H), 6.88 (d, *J* = 3.4 Hz, 1H), 3.85 – 3.80 (m, 1H), 3.52 – 3.43 (m, 2H), 3.17 (d, *J* = 5.8 Hz, 2H); **^{13}C NMR (101 MHz, CDCl$_3$)** δ 164.5, 150.4, 148.3, 141.5, 141.0, 140.1, 137.3, 132.6, 132.4, 129.1, 128.9, 128.8, 128.7, 126.8, 126.4, 126.1, 124.0, 122.4, 57.2, 45.6, 36.8; **HRMS** (ESI/TOF-Q) m/z: [M+H]$^+$ Calculated for $C_{25}H_{23}N_3O_2S_2H^+$ 462.1304; Found 462.1309.

(E)-N-(2-((oxodiphenyl-l6-sulfaneylidene)amino)-5-phenylpent-4-en-1-yl)-picolinamide (6a):

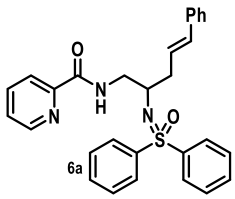

Compound **6a** was synthesized according to GP-1 as pale yellow solid; eluent (30% ethyl acetate in hexane); m.p.: 120 – 122 °C; **Yield:** 87% (84 mg); **^{1}H NMR (400 MHz, CDCl$_3$)** δ 8.75 – 8.72 (m, 1H), 8.61 (d, *J* = 4.8 Hz, 1H), 8.18 (d, *J* = 7.8 Hz, 1H), 7.99 (d, *J* = 7.9 Hz, 2H), 7.95 (d, *J* = 7.8 Hz, 2H), 7.84 (t, *J* = 7.6 Hz, 1H), 7.48 – 7.41 (m, 3H), 7.39 – 7.27 (m, 8H), 7.19 (t, *J* = 7.3 Hz, 1H), 6.48 (d, *J* = 15.8 Hz, 1H), 6.37 – 6.30 (m, 1H), 3.88 – 3.82 (m, 1H), 3.51 – 3.44 (m, 1H), 3.42 – 3.36 (m, 1H), 2.66 – 2.54 (m, 2H); **^{13}C NMR (101 MHz, CDCl$_3$)** δ 164.4, 150.5, 148.3, 140.8, 140.7, 137.8, 137.3, 132.5 (2xC), 132.3, 129.2, 129.1, 128.90, 128.87, 128.6, 127.8, 127.1, 126.2, 126.1, 122.4, 55.6, 45.7, 40.4; **HRMS** (ESI/TOF-Q) m/z: [M+H]$^+$ Calculated for $C_{29}H_{27}N_3O_2SH^+$ 482.1897; Found 482.1888.

(E)-N-(2-((bis(4-(tert-butyl)phenyl)(oxo)-l6-sulfaneylidene)amino)-5-phenylpent-4-en-1-yl)picolinamide (6b):

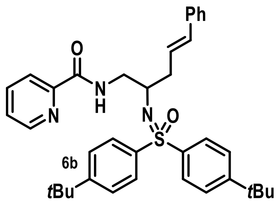

Compound **6b** was synthesized according to GP-1 as pale yellow sticky liquid; eluent (20% ethyl acetate in hexane); **Yield:** 90% (107 mg); **^{1}H NMR (400 MHz, CDCl$_3$)** δ 8.77 – 8.74 (m, 1H), 8.63 (d, *J* = 4.8 Hz, 1H), 8.18 (d, *J* = 7.8 Hz, 1H), 7.92 – 7.82 (m, 5H), 7.45 – 7.42 (m, 1H), 7.38 – 7.27 (m, 8H), 7.19 (t, *J* = 7.3 Hz, 1H), 6.47 (d, *J* = 15.9 Hz, 1H), 6.39 – 6.32 (m, 1H), 3.89 – 3.83 (m, 1H), 3.49 – 3.38 (m, 2H), 2.66 – 2.53 (m, 2H), 1.27 (s, 9H), 1.25 (s, 9H); **^{13}C NMR (101 MHz, CDCl$_3$)** δ 164.4, 156.1 (2xC), 150.5, 148.3, 137.9, 137.84, 137.76, 137.3, 132.1, 128.7 (2xC), 128.6, 128.0, 127.0, 126.22, 126.17, 126.1, 126.0, 122.4, 55.5, 45.8, 40.5, 35.11, 35.09, 31.16, 31.15; **HRMS** (ESI/TOF-Q) m/z: [M+H]$^+$ Calculated for $C_{37}H_{43}N_3O_2SH^+$ 594.3149; Found 594.3185.

(E)-N-(2-((5-oxido-5l4-dibenzo[b,d]thiophen-5-ylidene)amino)-5-phenylpent-4-en-1-yl)picolinamide (6c):

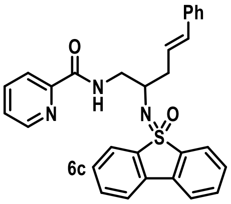

Compound **6c** was synthesized according to GP-1 as sticky colourless liquid; eluent (30% ethyl acetate in hexane); **Yield:** 82% (82 mg); **^{1}H NMR (400 MHz, CDCl$_3$)** δ 8.45 – 8.42 (m, 2H), 8.14 (d, *J* = 7.8 Hz, 1H), 7.82 – 7.75 (m, 3H), 7.72 – 7.66 (m, 2H), 7.49 (t, *J* = 7.7 Hz, 1H), 7.43 – 7.35 (m, 3H), 7.32 – 7.27 (m, 3H), 7.23 – 7.19 (m, 3H), 6.39 (d, *J* = 15.8 Hz, 1H), 6.25 – 6.17(m, 1H), 3.96 – 3.89 (m, 1H), 3.86 – 3.80 (m, 1H), 3.39 – 3.32 (m, 1H), 2.57 – 2.46 (m, 2H); **^{13}C NMR (101 MHz, CDCl$_3$)** δ 164.5, 150.1, 148.1, 139.8, 139.5, 137.6, 137.2, 133.0, 132.8, 132.3, 132.0, 131.8, 130.2, 128.4 (2xC), 127.3, 127.1, 126.3, 126.0, 122.8, 122.7, 122.2, 121.5, 121.4, 55.2, 45.6, 39.9; **HRMS** (ESI/TOF-Q) m/z: [M+Na]$^+$ Calculated for $C_{29}H_{25}N_3O_2SNa^+$ 502.1560; Found 502.1591.

N-(3-(4-ethoxyphenyl)-2-((oxodiphenyl-l6-sulfaneylidene)amino)-1-phenylpropyl)-picolinamide (6d):

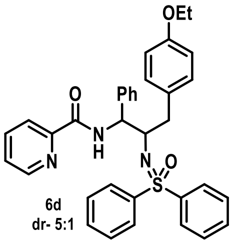

Compound **6d** was obtained as pale yellow liquid; eluent (20% ethyl acetate in hexane); **Yield:** 36% (41 mg); **1H NMR (400 MHz, CDCl₃)** δ 9.44 – 9.42 (m, 5.95H), 8.71 – 8.60 (m, 6.14H), 8.16 – 8.05 (m, 5.95H), 8.04 – 7.95 (m, 2.28H), 7.92 – 7.84 (m, 11.91H), 7.84 – 7.80 (m, 6.04H), 7.78 – 7.76 (m, 1.10H), 7.65 – 7.62 (m, 10.02H), 7.53 – 7.46 (m, 6.06H), 7.45 – 7.41 (m, 21.26H), 7.40 – 7.37 (m, 6.19H), 7.36 – 7.30 (m, 21.07H), 7.29 – 7.27 (m, 6.08H), 7.25 – 7.19 (m, 12.20H), 7.07 – 7.04 (m, 1.96H), 7.01 – 6.98 (m, 10.18H), 6.91 – 6.85 (m, 2.16H), 6.81 – 6.77 (m, 10.13H), 5.28 – 5.25 (m, 5.18H), 5.17 – 5.09 (m, 1.05H), 4.12 – 4.08 (m, 2.08H), 4.05 – 3.99 (m, 10.20H), 3.70 – 3.66 (m, 6.01H), 2.89 – 2.84 (m, 5.90H), 2.78 – 2.72 (m, 6.00H), 1.43 – 1.39 (m, 18.29H); **^{13}C NMR (101 MHz, $CDCl_3$)** δ 163.5, 162.3, 159.0, 157.6, 150.7, 148.4, 147.5, 141.0, 139.7, 139.1, 137.4, 137.1, 132.7, 132.4, 132.2, 131.6, 131.0, 130.4, 130.0, 129.6, 129.0, 128.95, 128.90, 128.1, 127.4, 127.0, 126.4, 126.0, 122.5, 114.7, 114.3, 64.1, 63.6, 62.2, 61.7, 57.4, 41.4, 15.09, 15.05; **HRMS** (ESI/TOF-Q) m/z: $[M+H]^+$ Calculated for $C_{35}H_{33}N_3O_3SH^+$ 576.2315; Found 576.2319; **dr-5:1**.

N-(3-(4-ethoxyphenyl)-1-(4-methoxyphenyl)-2-((oxodi-p-tolyl-l6-sulfaneylidene) amino)propyl)picolinamide (6e):

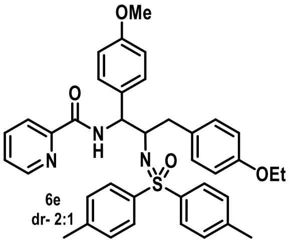

6e
dr- 2:1

Compound **6e** was obtained as pale yellow liquid; eluent (20% ethyl acetate in hexane); **Yield:** 49% (62 mg); **^{1}H NMR (400 MHz, $CDCl_3$)** δ 9.49 – 9.39 (m, 3H), 8.74 – 8.64 (m, 3H), 8.21 – 8.08 (m, 3H), 7.95 (d, *J* = 8.7 Hz, 1H), 7.86 – 7.79 (m, 4H), 7.77 – 7.75 (m, 3H), 7.66 – 7.63 (m, 2H), 7.59 – 7.57 (m, 3H), 7.48 – 7.45 (m, 2H), 7.44 – 7.42 (m, 2H), 7.37 – 7.34 (m, 4H), 7.13 – 7.09 (m, 6H), 7.03 – 6.97 (m, 8H), 6.93 – 6.89 (m, 6H), 6.87 – 6.85 (m, 4H), 6.84 – 6.81 (m, 2H), 6.80 – 6.78 (m, 4H), 6.75 (d, *J* = 2.8 Hz, 1H), 6.74 – 6.72 (m, 2H), 5.72 – 5.69 (m, 1H), 5.19 – 5.15 (m, 2H), 4.06 – 3.99 (m, 8H), 3.80 – 3.79 (m, 9H), 3.68 – 3.64 (m, 2H), 3.42 – 3.38 (m, 1H), 2.84 – 2.73 (m, 4H), 2.33 – 2.31 (m, 15H), 2.23 (s, 3H), 1.43 – 1.41 (m, 6H), 1.41 – 1.40 (m, 3H) **; ^{13}C NMR (101 MHz, $CDCl_3$)** δ 163.6, 163.3, 158.9, 158.3, 158.1, 157.5, 157.4, 151.0, 150.8, 148.4, 148.3, 143.4, 143.0, 142.9, 142.5, 138.2, 137.1, 137.0, 136.9, 136.0, 134.0, 132.4, 131.7, 131.4, 131.3, 131.1, 131.0, 130.7, 129.60, 129.56, 129.4, 129.3, 129.0, 128.9, 128.8, 125.93, 125.89, 122.5, 122.4, 115.2, 114.2, 114.0, 113.5, 113.1, 63.6 (2xC), 63.5, 62.4, 61.6, 56.5, 55.3 (2xC), 53.2, 41.6, 41.5, 41.0, 21.6, 21.5, 15.1 (2xC); **HRMS** (ESI/TOF-Q) m/z: $[M+H]^+$ Calculated for $C_{38}H_{39}N_3O_4SH^+$ 634.2734; Found 634.2723; **dr-2:1**.

4-(2-((oxodiphenyl-l6-sulfaneylidene)amino)-3-(picolinamido)propyl)benzyl 2-(11-oxo-6,11-dihydrodibenzo[b,e]oxepin-2-yl)acetate (6f):

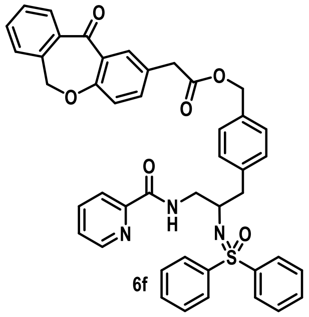

6f

Compound **6f** was synthesized according to GP-1 as colourless sticky liquid; eluent (50% ethyl acetate in hexane); **Yield:** 75% (110 mg); **^{1}H NMR (400 MHz, CDCl$_3$)** δ 8.73 (t, *J* = 6.1 Hz, 1H), 8.63 (d, *J* = 4.7 Hz, 1H), 8.17 (d, *J* = 7.8 Hz, 1H), 8.11 (d, *J* = 2.4 Hz, 1H), 7.93 – 7.91 (m, 2H), 7.88 – 7.81 (m, 2H), 7.56 – 7.52 (m, 1H), 7.48 – 7.42 (m, 5H), 7.39 – 7.33 (m, 5H), 7.29 – 7.27 (m, 1H), 7.26 – 7.24 (m, 2H), 7.22 – 7.20 (m, 3H), 6.95 (d, *J* = 8.4 Hz, 1H), 5.17 – 5.13 (m, 4H), 3.83 – 3.77 (m, 1H), 3.67 (s, 2H), 3.51 – 3.40 (m, 2H), 2.97 (d, *J* = 6.3 Hz, 2H).**^{13}C NMR (101 MHz, CDCl$_3$)** δ 190.9, 171.4, 164.5, 160.6, 150.5, 148.3, 141.0, 140.6, 140.0, 139.8, 137.3, 136.4, 135.7, 133.7, 132.9, 132.6, 132.4, 130.4, 129.6, 129.4, 129.1, 129.0, 128.8, 128.7, 128.4, 127.9, 127.8, 126.1 125.3, 122.4, 121.2, 73.7, 66.9, 57.5, 45.9, 42.7, 40.3; **HRMS** (ESI/TOF-Q) m/z: [M+H]$^+$ Calculated for $C_{44}H_{37}N_3O_6SH^+$ 736.2476; Found 736.2487.

4-(2-((oxodiphenyl-l6-sulfaneylidene)amino)-3-(picolinamido)propyl)benzyl (2R)-2-(6-methoxynaphthalen-2-yl)propanoate (6g):

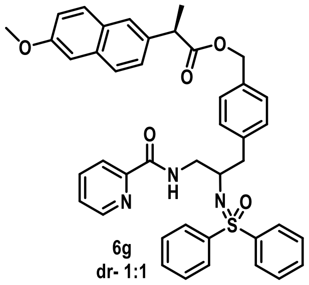

6g
dr- 1:1

Compound **6g** was synthesized according to GP-1 as colourless sticky liquid; eluent (50% ethyl acetate in hexane); **Yield:** 84% (117 mg); **^{1}H NMR (400 MHz, CDCl$_3$)** δ 8.78 – 8.75 (m, 2H), 8.64 (d, *J* = 4.8 Hz, 2H), 8.16 (d, *J* = 7.8 Hz, 2H), 7.92 (d, *J* = 7.8 Hz, 4H), 7.87 – 7.82 (m, 2H), 7.63 – 7.58 (m, 6H), 7.47 – 7.30 (m, 16H), 7.21 – 7.14 (m, 12H), 7.12 – 7.03 (m, 4H), 5.17 – 5.06 (m, 4H), 3.89 (d, *J* = 3.2 Hz, 6H), 3.85 – 3.79 (m, 4H), 3.49 – 3.43 (m, 2H), 3.41 – 3.35 (m, 2H), 2.95 (d, *J* = 6.7 Hz, 4H), 1.56 (d, *J* = 7.2 Hz, 6H); **^{13}C NMR (101 MHz, CDCl$_3$)** δ 174.64, 174.63, 164.5 (2xC), 157.75, 157.73, 150.5 (2xC), 148.3 (2xC), 141.1 (2xC), 139.82 (2xC), 139.78 (2xC), 137.3 (2xC), 135.7 (2xC), 134.00, 133.96, 133.8 (2xC), 132.6, 132.3, 130.3 (2xC), 129.36 (2xC), 129.35 (2xC), 129.1 (2xC), 129.03 (2xC), 128.97 (2xC), 128.8(2xC), 128.7 (2xC), 128.2, 128.1, 127.2 (2xC), 126.4, 126.3, 126.10 (2xC), 126.07, 126.0, 122.4 (2xC), 119.1, 119.0, 105.75, 105.74, 66.65, 66.59, 57.6 (2xC), 55.4 (2xC), 45.9 (2xC), 45.64, 45.62, 42.7 (2xC), 18.72, 18.69; **HRMS** (ESI/TOF-Q) m/z: [M+H]$^+$ Calculated for $C_{42}H_{39}N_3O_5SH^+$ 698.2683; Found 698.2690; **dr-1:1.**

4-(2-((oxodiphenyl-l6-sulfaneylidene)amino)-3-(picolinamido)propyl)benzyl palmitate (6h):

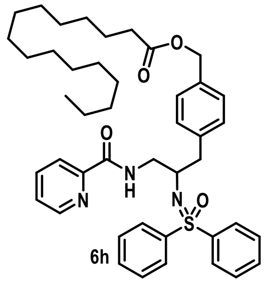

6h

Compound **6h** was synthesized according to GP-1 as brown solid; eluent (45% ethyl acetate in hexane); m.p.: 74 – 76 °C; **Yield:** 85% (123 mg); **^{1}H NMR (400 MHz, CDCl$_3$)** δ 8.83 - 8.80 (t, *J* = 6.2 Hz, 1H), 8.69 (d, *J* = 4.8 Hz, 1H), 8.21 (d, *J* = 7.8 Hz, 1H), 7.98 (d, *J* = 7.8 Hz, 2H), 7.88 (t, *J* = 7.7 Hz, 1H), 7.52 – 7.46 (m, 4H), 7.42 (t, *J* = 7.9 Hz, 3H), 7.34 – 7.28 (m, 6H), 5.15 (s, 2H), 3.90 – 3.84 (m, 1H), 3.55 – 3.43 (m, 2H), 3.03 (d, *J* = 6.5 Hz, 2H), 2.37 (t, *J* = 7.6 Hz, 2H), 1.69 – 1.62 (m, 2H), 1.33 – 1.27 (m, 24H), 0.92 (t, *J* = 6.7 Hz, 3H). **^{13}C NMR (101 MHz, CDCl$_3$)** δ 173.8, 164.5, 150.5, 148.3, 141.0, 139.9, 139.8, 137.3, 134.2, 132.5, 132.3, 130.3, 129.1, 129.0, 128.9, 128.7, 128.3, 126.0, 122.4, 66.1, 57.5, 45.8, 42.7, 34.5, 32.0, 29.80, 29.79, 29.78, 29.76, 29.74, 29.69, 29.54, 29.46, 29.34, 29.27, 25.1, 22.8, 14.2; **HRMS** (ESI/TOF-Q) m/z: $[M+H]^+$ Calculated for $C_{44}H_{57}N_3O_4SH^+$ 724.4143; Found 724.4181.

Ethyl(4-(2-((oxodiphenyl-l6-sulfaneylidene)amino)-3-(picolinamido)propyl)-benzoyl)-L-methioninate (6i):

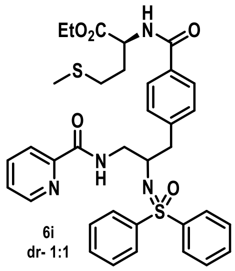

6i
dr- 1:1

Compound **6i** was synthesized according to GP-1 as colourless sticky liquid; eluent (80% ethyl acetate in hexane); **Yield:** 52% (68 mg); **^{1}H NMR (400 MHz, CDCl$_3$)** δ 8.74 – 8.72 (m, 2H), 8.63 (d, *J* = 4.5 Hz, 2H), 8.16 (d, *J* = 7.9 Hz, 2H), 7.91 (d, *J* = 7.8 Hz, 4H), 7.84 (t, *J* = 8.1 Hz, 2H), 7.74 (d, *J* = 7.8 Hz, 4H), 7.49 – 7.35 (m, 14H), 7.30 (d, *J* = 7.9 Hz, 4H), 7.25 – 7.24 (m, 2H), 6.93 (d, *J* = 7.8 Hz, 2H), 4.93 – 4.86 (m, 2H), 4.28 – 4.23 (m, 4H), 3.80 – 3.78 (m, 2H), 3.73 – 3.44 (m, 6H), 3.03 – 3.01 (m, 4H), 2.64 – 2.54 (m, 4H), 2.34 – 2.25 (m, 2H), 2.15 – 2.10 (m, 8H), 1.33 – 1.29 (m, 6H); **^{13}C NMR (126 MHz, CDCl$_3$)** δ 172.3 (2xC), 167.1 (2xC), 164.6 (2xC), 150.4 (2xC), 148.4 (2xC), 144.1 (2xC), 140.9 (2xC), 139.9 (2xC), 137.3 (2xC), 132.7 (2xC), 132.4 (2xC), 131.8 (2xC), 130.4 (2xC), 129.20, 129.16, 129.13, 129.10, 128.73, 128.71, 128.6 (2xC), 127.1 (2xC), 126.1 (2xC), 122.4 (2xC), 61.9 (2xC), 57.3 (2xC), 52.3 (2xC), 46.0, 45.9, 42.8 (2xC), 32.0, 31.9, 30.3 (2xC), 15.7 (2xC), 14.3 (2xC); **HRMS** (ESI/TOF-Q) m/z: $[M+H]^+$ Calculated for $C_{35}H_{38}N_4O_5S_2H^+$ 659.2356; Found 659.2383; **dr-1:1.**

(E)-N-(3-((oxodiphenyl-l6-sulfaneylidene)amino)-6-phenylhex-5-en-1-yl) picolinamide (6j) & (E)-N-(3-(((oxodiphenyl-l6-sulfaneylidene)amino)methyl)-5-phenylpent-4-en-1-yl)picolinamide (6j′):

Compound **(6j+ 6j′)** was synthesized according to GP-2 as colourless sticky liquid; eluent (30% ethyl acetate in hexane); **Yield:** 90% (89 mg); **(6j:6j′ = 1:2)**.

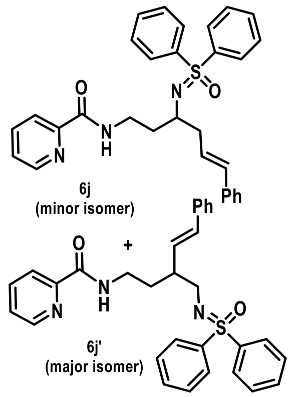

Minor isomer (**6j**): **^{1}H NMR (400 MHz, $CDCl_3$)** δ 8.55 (brs, 1H), 8.45 (d, *J* = 4.3 Hz, 1H), 8.19 – 8.17 (m, 1H), 8.04 – 8.02 (m, 2H), 7.99 – 7.97 (m, 2H), 7.84 – 7.80 (m, 1H), 7.49 – 7.42 (m, 4H), 7.40 – 7.38 (m, 1H), 7.37 – 7.33 (m, 3H), 7.32 – 7.28 (m, 3H), 7.21 – 7.16 (m, 1H), 6.41 (d, *J* = 15.8 Hz, 1H), 6.21 (dt, *J* = 15.8, 7.3 Hz, 1H), 3.80 – 3.72 (m, 1H), 3.70 – 3.61 (m, 1H), 3.38 – 3.32 (m, 1H), 2.58 – 2.48 (m, 2H), 2.06 – 1.98 (m, 1H), 1.92 – 1.83 (m, 1H). **^{13}C NMR (101 MHz, $CDCl_3$)** δ 164.5, 150.5, 148.1, 141.2, 140.9, 137.9, 137.3, 132.5, 132.4, 132.1, 129.2, 129.0, 128.9, 128.8, 128.6, 128.3, 127.1, 126.2, 125.9, 122.2, 53.1, 42.3, 37.0, 36.7; **HRMS** (ESI/TOF-Q) m/z: $[M+H]^+$ Calculated for $C_{30}H_{29}N_3O_2SH^+$ 496.2053; Found 496.2056.

Major isomer **(6j′): ^{1}H NMR (500 MHz, $CDCl_3$)** δ 8.42 (d, *J* = 4.7 Hz, 1H), 8.23 (t, *J* = 6.0 Hz, 1H), 8.17 (d, *J* = 7.7 Hz, 1H), 7.97 – 7.93 (m, 4H), 7.82 – 7.79 (m, 1H), 7.49 – 7.46 (m, 2H), 7.44 – 7.41 (m, 4H), 7.38 – 7.36 (m, 3H), 7.29 (t, *J* = 7.5 Hz, 2H), 7.20 (t, *J* = 7.2 Hz, 1H), 6.54 (d, *J* = 15.9 Hz, 1H), 6.17 (dd, *J* = 15.9, 8.8 Hz, 1H), 3.60 – 3.55 (m, 2H), 3.13 (d, *J* = 6.3 Hz, 2H), 2.68 – 2.61 (m, 1H), 2.19 – 2.13 (m, 1H), 1.83 – 1.77 (m, 1H). **^{13}C NMR (126 MHz, $CDCl_3$)** δ 164.3, 150.3, 148.1, 141.0, 140.9, 137.7, 137.3, 132.9, 132.5, 132.4, 131.3, 129.2 (2xC), 128.8, 128.7, 128.6, 127.2, 126.3, 126.0, 122.2, 48.4, 44.2, 38.1, 32.5; **HRMS** (ESI/TOF-Q) m/z: $[M+H]^+$ Calculated for $C_{30}H_{29}N_3O_2SH^+$ 496.2053; Found 496.2060.

(E)-N-(3-(((oxodiphenyl-l6-sulfaneylidene)amino)methyl)-5-phenylpent-4-en-1-yl)picolinamide (6k) & (E)-N-(3-(((oxodi-p-tolyl-l6-sulfaneylidene)amino)methyl)-5-phenylpent-4-en-1-yl)picolinamide (6k′):

Compound **(6k+ 6k′)** was synthesized according to GP-2 as colourless sticky liquid; eluent (30% ethyl acetate in hexane); **Yield:** 95% (99 mg); **(6k:6k′ = 1:2)**.

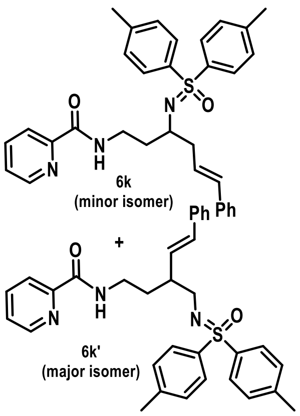

Minor Isomer (**6k**): **^{1}H NMR (400 MHz, $CDCl_3$)** δ 8.55 – 8.53 (m, 1H), 8.48 – 8.46 (m, 1H), 8.20 – 8.17 (m, 1H), 7.90 – 7.85 (m, 3H), 7.84 – 7.80 (m, 2H), 7.40 – 7.37 (m, 1H), 7.34 – 7.28 (m, 4H), 7.23 – 7.16 (m, 3H), 7.13 – 7.11 (m, 2H), 6.40 (d, *J* = 15.9 Hz, 1H), 6.21 (dt, *J* = 15.8, 7.3 Hz, 1H), 3.78 – 3.72 (m, 1H), 3.69 – 3.60 (m, 1H), 3.35 – 3.29 (m, 1H), 2.56 – 2.46 (m, 2H), 2.34 (s, 3H), 2.32 (s, 3H), 2.04 – 1.96 (m, 1H), 1.90 – 1.81 (m, 1H). **^{13}C NMR (101 MHz, $CDCl_3$)** δ 164.5, 150.6, 148.1, 143.2, 142.9, 138.5, 138.2, 137.9, 137.3, 131.9, 129.8, 129.7, 128.9, 128.8, 128.6, 128.4, 127.0, 126.2, 125.9, 122.2, 53.0, 42.4, 37.0, 36.7, 21.5 (2xC); **HRMS** (ESI/TOF-Q) m/z: $[M+H]^+$ Calculated for $C_{32}H_{33}N_3O_2SH^+$ 524.2366; Found 524.2360.

Major isomer **(6k′): ^{1}H NMR (400 MHz, $CDCl_3$)** δ 8.42 (d, *J* = 4.6 Hz, 1H), 8.24 (brs, 1H), 8.16 (d, *J* = 7.7 Hz, 1H), 7.86 – 7.78 (m, 5H), 7.37 – 7.36 (m, 3H), 7.29 (d, *J* = 7.3

Hz, 2H), 7.26 – 7.19 (m, 5H), 6.55 (d, *J* = 15.8 Hz, 1H), 6.13 (dd, *J* = 15.7, 8.2 Hz, 1H), 3.58 – 3.55 (m, 2H), 3.13 (d, *J* = 5.7 Hz, 2H), 2.75 – 2.63 (m, 1H), 2.360 (s, 3H), 2.356 (s, 3H), 2.17 – 2.12 (m, 1H), 1.80 – 1.75 (m, 1H); **^{13}C NMR (101 MHz, $CDCl_3$)** δ 164.3, 150.2 (2xC), 148.1, 143.8, 137.6 (3xC), 137.3, 132.6, 131.5, 130.1(2xC), 128.8, 128.7, 128.5, 127.1, 126.3, 126.0, 122.2, 48.2, 43.9, 38.1, 32.4, 21.6 (2xC); **HRMS** (ESI/TOF-Q) m/z: $[M+H]^+$ Calculated for $C_{32}H_{33}N_3O_2SH^+$ 524.2366; Found 524.2366;

(*E*)-N-(3-((bis(4-chlorophenyl)(oxo)-l6-sulfaneylidene)amino)-6-phenylhex-5-en-1-yl)picolinamide (6l) & (E)-N-(3-(((bis(4-chlorophenyl)(oxo)-l6-sulfaneylidene) amino) methyl)-5-phenylpent-4-en-1-yl)picolinamide (6l′):

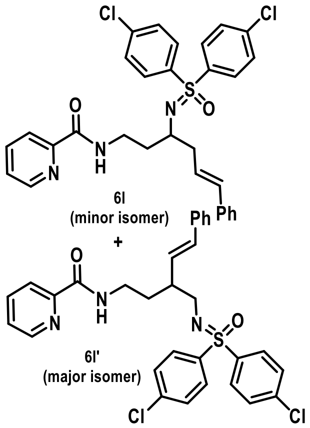

Compound **(6l+6l′)** was synthesized according to GP-2 as colourless sticky liquid; eluent (30% ethyl acetate in hexane); **Yield:** 86% (97 mg); **(6l:6l′= 1:2)**.

Minor isomer (**6l**): **^{1}H NMR (500 MHz, $CDCl_3$)** δ 8.42 – 8.38 (m, 2H), 8.14 (d, *J* = 7.8 Hz, 1H), 7.87 (d, *J* = 8.2 Hz, 2H), 7.82 – 7.81 (m, 2H), 7.78 (d, *J* = 7.8 Hz, 1H), 7.37 – 7.33 (m, 3H), 7.26 – 7.21 (m, 6H), 7.15 (t, *J* = 6.8 Hz, 1H), 6.36 (d, *J* = 15.8 Hz, 1H), 6.11 (dt, *J* = 15.3, 7.3 Hz, 1H), 3.71 – 3.65 (m, 1H), 3.63 – 3.56 (m, 1H), 3.30 – 3.25 (m, 1H), 2.49 – 2.42 (m, 2H), 2.00 – 1.93 (m, 1H), 1.85 – 1.79 (m, 1H); **^{13}C NMR (126 MHz, $CDCl_3$)** δ 164.5, 150.3, 148.1, 139.4 (2xC), 139.2, 139.1, 137.7, 137.4, 132.3, 130.3, 130.2, 129.6, 129.4, 128.7, 128.0, 127.2, 126.1 (2xC), 122.3, 53.1, 42.2, 36.8, 36.7; **HRMS** (ESI/TOF-Q) m/z: $[M+H]^+$ Calculated for $C_{30}H_{27}Cl_2N_3O_2SH^+$ 564.1274; Found 564.1279.

Major isomer (**6l′**): **^{1}H NMR (500 MHz, $CDCl_3$)** δ 8.44 – 8.42 (m, 1H), 8.21 (t, *J* = 6.1 Hz, 1H), 8.17 (d, *J* = 7.8 Hz, 1H), 7.88 – 7.84 (m, 3H), 7.83 – 7.80 (m, 2H), 7.40 – 7.35 (m, 7H), 7.30 (t, *J* = 7.7 Hz, 2H), 7.23 – 7.19 (m, 1H), 6.53 (d, *J* = 15.9 Hz, 1H), 6.13 (dd, *J* = 15.9, 8.8 Hz, 1H), 3.60 – 3.53 (m, 2H), 3.10 (d, *J* = 6.4 Hz, 2H), 2.66 – 2.59 (m, 1H), 2.14 – 2.09 (m, 1H), 1.79 – 1.77 (m, 1H); **^{13}C NMR (126 MHz, $CDCl_3$)** δ 164.3, 150.2, 148.1, 139.4, 139.3, 139.2, 139.1, 137.5, 137.4, 132.6, 131.5, 130.2, 130.1, 129.6 (2xC), 128.6, 127.3, 126.3, 126.1, 122.2, 48.3, 44.1, 38.0, 32.5; **HRMS** (ESI/TOF-Q) m/z: $[M+H]^+$ Calculated for $C_{30}H_{27}Cl_2N_3O_2SH^+$ 564.1274; Found 564.1271.

(E)-N-(3-((5-oxido-5l4-dibenzo[b,d]thiophen-5-ylidene)amino)-6-phenylhex-5-en-1-yl)picolinamide (6m) & (E)-N-(3-(((5-oxido-5l4-dibenzo[b,d]-thiophen-5-ylidene)-amino)methyl)-5-phenylpent-4-en-1-yl) picolinamide (6m′) :

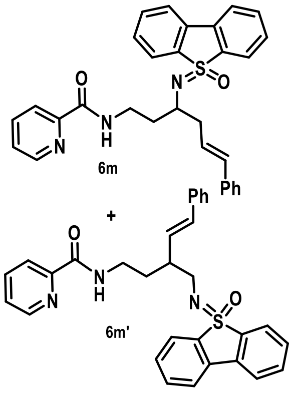

Compound **(6m+6m′)** was synthesized according to GP-2 as colourless sticky liquid; eluent (30% ethyl acetate in hexane); **Yield:** 77% (76 mg); **(6m:6m′=1:1)**.

^{1}H NMR (400 MHz, $CDCl_3$) δ 8.52 – 8.50 (m, 1H), 8.48 – 8.46 (m, 1H), 8.43 – 8.40 (m, 1H), 8.16 (t, *J* = 7.6 Hz, 3H), 7.91 (d, *J* = 7.6 Hz, 1H), 7.83 – 7.76 (m, 7H), 7.73 – 7.68 (m, 2H), 7.59 – 7.54 (m, 3H), 7.48 – 7.42 (m, 4H), 7.40 – 7.35 (m, 2H), 7.32 – 7.28 (m, 4H), 7.24 – 7.20 (m, 3H), 7.19 – 7.18 (m, 1H), 7.16 – 7.10 (m, 3H), 6.46 (d, *J* = 15.9 Hz, 1H), 6.30 (d, *J* = 15.8 Hz, 1H), 6.13 – 6.04 (m, 2H), 3.92 – 3.86 (m, 1H), 3.71 – 3.62 (m, 2H), 3.56 – 3.48 (m, 2H), 3.36 – 3.25 (m, 2H), 2.61 – 2.53 (m, 1H), 2.47 – 2.43 (m, 2H),

2.14 – 2.04 (m, 2H), 1.84 – 1.75 (m, 2H); **^{13}C NMR (126 MHz, CDCl$_3$)** δ 164.5, 164.3, 150.4, 150.1, 148.2, 148.1, 140.2, 139.5, 139.0, 138.9, 137.6, 137.5, 137.35, 137.29, 133.1, 133.05, 133.02, 132.5, 132.2, 132.11(2xC), 132.06, 131.6, 131.5 (2xC), 130.3, 130.22, 130.18, 130.1, 128.5, 128.4, 127.8, 127.2, 127.0, 126.4, 126.2, 126.1, 126.0, 122.72, 122.69, 122.24, 122.19, 121.7, 121.6, 53.8, 49.1, 44.0, 42.2, 38.0, 37.1, 37.0, 32.4; **HRMS** (ESI/TOF-Q) m/z: $[M+H]^+$ Calculated for $C_{30}H_{27}N_3O_2SH^+$ 494.1897; Found 494.1903.

(E)-N-(3-((10-oxido-10l4-phenoxathiin-10-ylidene)amino)-6-phenylhex-5-en-1-yl) picolinamide (6n) & (E)-N-(3-(((10-oxido-10l4-phenoxathiin-10-ylidene)amino) methyl)-5-phenylpent-4-en-1-yl)picolinamide (6n′):

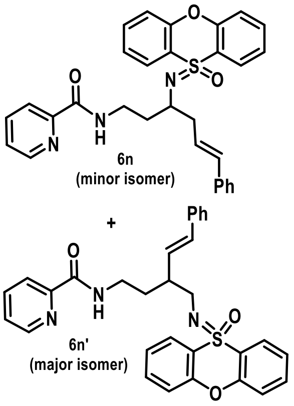

Compound **(6n+6n′)** was synthesized according to GP-2 as pale yellow liquid; eluent (30% ethyl acetate in hexane); **Yield:** 81% (95 mg); **(6n:6n′ =1:3)**.

^{1}H NMR (400 MHz, CDCl$_3$) δ 8.56 – 8.54 (m, 1H), 8.52 – 8.49 (m, 1H), 8.43 – 8.42 (m, 3H), 8.20 – 8.18 (m, 1H), 8.17 – 8.10 (m, 6H), 8.09 – 8.04 (m, 2H), 7.97 – 7.94 (m, 5H), 7.86 – 7.84 (m, 1H), 7.82 – 7.77 (m, 3H), 7.58 – 7.52 (m, 7H), 7.43 – 7.39 (m, 2H), 7.38 – 7.35 (m, 4H), 7.34 – 7.30 (m, 9H), 7.29 – 7.26 (m, 15H), 7.26 – 7.23 (m, 4H), 7.21 – 7.17 (m, 7H), 7.02 – 7.00 (m, 1H), 6.39 (d, *J* = 15.9 Hz, 3H), 6.18 (d, *J* = 15.8 Hz, 1H), 5.98 (dd, *J* = 15.9, 8.8 Hz, 3H), 5.83 (dt, *J* = 15.8, 7.3 Hz, 1H), 3.60 – 3.54 (m, 1H), 3.50 – 3.40 (m, 8H), 3.02 – 2.93 (m, 6H), 2.54 – 2.47 (m, 1H), 2.27 – 2.23 (m, 2H), 2.03 – 1.96 (m, 4H), 1.68 – 1.60 (m, 4H); **^{13}C NMR (101 MHz, CDCl$_3$)** δ 164.4, 164.2, 152.02, 152.01, 151.9, 151.51, 150.50, 150.1, 148.2, 148.11, 148.07, 148.0, 137.8, 137.5 (2xC), 137.3, 133.6 (2xC), 133.1, 132.2, 131.9, 131.4, 128.5 (2xC), 128.3, 127.5, 127.2, 127.0, 126.3 (3xC), 126.04, 125.98, 125.8, 124.8, 124.72, 124.68 (2xC), 124.6 (2xC), 124.4, 124.3, 122.25, 122.16, 119.1, 118.99, 118.96, 118.93, 53.6, 49.9, 43.7, 42.0, 37.9, 36.8, 36.7, 32.3; **HRMS** (ESI/TOF-Q) m/z: $[M+H]^+$ Calculated for $C_{30}H_{27}N_3O_3SH^+$ 510.1846; Found 510.1853.

(E)-N-(3-((10-oxido-9-oxo-9H-10l4-thioxanthen-10-ylidene)amino)-6-phenylhex-5-en-1-yl)picolinamide (6o) & (E)-N-(3-(((10-oxido-9-oxo-9H-10l4-thioxanthen-10-ylidene)amino)methyl)-5-phenylpent-4-en-1-yl)picolinamide (6o′):

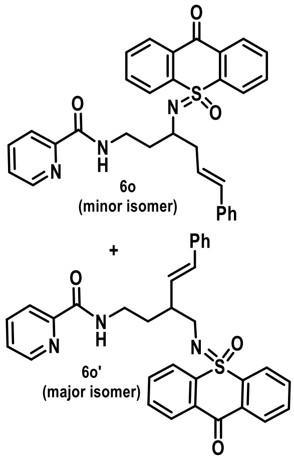

Compound **(6o+6o′)** was synthesized according to GP-2 as pale yellow liquid; eluent (30% ethyl acetate in hexane); **Yield:** 79% (82 mg); **(6o:6o′=1:3)**.

^{1}H NMR (400 MHz, CDCl$_3$) δ 8.54 – 8.52 (m, 1H), 8.51 – 8.47 (m, 1H), 8.41 – 8.40 (m, 3H), 8.26 – 8.18 (m, 9H), 8.13 – 8.07 (m, 12H), 7.97 – 7.95 (m, 1H), 7.85 – 7.81 (m, 1H), 7.81 – 7.69 (m, 12H), 7.68 – 7.60 (m, 7H), 7.45 – 7.39 (m, 2H), 7.36 – 7.33 (m, 3H), 7.28 – 7.26 (m, 2H), 7.26 – 7.23 (m, 12H), 7.21 – 7.16 (m, 4H), 7.13 – 7.11 (m, 2H), 6.36 (d, *J* = 15.1 Hz, 3H), 6.11 (d, *J* = 15.8 Hz, 1H), 5.93 (dd, *J* = 15.9, 8.8 Hz, 3H), 5.75 (dt, *J* = 15.8, 7.3 Hz, 1H), 3.62 – 3.54 (m, 1H), 3.50 – 3.37 (m, *J* = 6.5 Hz, 8H), 3.13 – 3.03 (m, 6H), 2.48 – 2.40 (m, 3H), 2.21 – 2.17 (m, 2H), 1.96 – 1.88 (m, 4H), 1.66 – 1.57 (m, 4H); **^{13}C NMR (101 MHz, CDCl$_3$)** δ 179.7, 179.5, 150.2 (2xC), 148.13, 148.07, 141.4, 141.3,

141.2, 141.1, 137.43 (3xC), 137.38, 137.3 (2xC), 134.1, 134.05, 134.01, 133.5, 132.6, 132.4, 132.2, 132.03, 132.00, 131.5, 131.2, 131.0 (2xC), 130.7, 129.6, 129.33, 129.31, 129.2, 128.5 (2xC), 128.3, 127.22, 127.15, 127.1, 126.4(3xC), 126.0, 124.63, 124.59, 124.56, 124.5, 122.3, 122.2, 53.9, 49.4, 43.7, 41.9, 37.8, 36.9, 32.4, 29.8; **HRMS** (ESI/TOF-Q) m/z: $[M+H]^+$ Calculated for $C_{31}H_{27}N_3O_3SH^+$ 522.1846; Found 522.1840.

N-(2-((oxodiphenyl-l6-sulfaneylidene)amino)-3-phenylpropyl)isoquinoline-1-carboxamide (4a′):

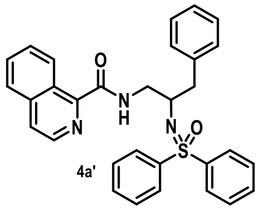
4a'

Compound **4a′** was obtained as pale yellow liquid; eluent (25% ethyl acetate in hexane); **Yield:** 82% (84 mg); **^{1}H NMR (500 MHz, CDCl$_3$)** δ 9.45 – 9.43 (m, 1H), 8.83 – 8.80 (m, 1H), 8.56 (d, *J* = 5.5 Hz, 1H), 7.95 – 7.92 (m, 2H), 7.86 (d, *J* = 8.2 Hz, 1H), 7.81 (d, *J* = 5.5 Hz, 1H), 7.73 – 7.70 (m, 1H), 7.66 – 7.63 (m, 1H), 7.44 – 7.40 (m, 3H), 7.38 – 7.33 (m, 3H), 7.32 – 7.30 (m, 2H), 7.28 (d, *J* = 1.7 Hz, 2H), 7.25 – 7.24 (m, 1H), 7.21 – 7.17 (m, 2H), 3.93 – 3.89 (m, 1H), 3.53 – 3.45 (m, 2H), 3.05 – 3.02 (m, 2H); **^{13}C NMR (126 MHz, CDCl$_3$)** δ 166.3, 149.4, 141.1, 140.6, 139.8, 137.5, 132.5, 132.3, 130.5, 130.2, 129.3, 129.1, 129.0, 128.9, 128.8, 128.5, 128.3, 128.1, 127.1, 126.9, 126.3, 124.1, 57.8, 46.0, 43.1; **HRMS** (ESI/TOF-Q) m/z: $[M+H]^+$ Calculated for $C_{31}H_{27}N_3O_2SH^+$ 506.1897; Found 506.1934.

N-(3-phenyl-2-(phenylamino)propyl)picolinamide (8a):

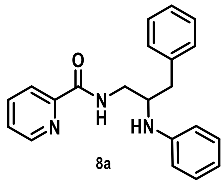
8a

Compound **8a** was synthesized according to GP-3 as off white solid; eluent (15% ethyl acetate in hexane); m.p.: 110 – 112 °C; **Yield:** 79% (52 mg); **^{1}H NMR (400 MHz, CDCl$_3$)** δ 8.53 (d, *J* = 4.7 Hz, 1H), 8.31 (t, *J* = 6.4 Hz, 1H), 8.22 (d, *J* = 7.9 Hz, 1H), 7.86 (t, *J* = 7.7 Hz, 1H), 7.44 – 7.41 (m, 1H), 7.34 (t, *J* = 7.5 Hz, 2H), 7.28 – 7.26 (m, 2H), 7.21 (t, *J* = 7.8 Hz, 3H), 6.74 – 6.72 (m, 3H), 4.03 (brs, 1H), 3.96 (t, *J* = 6.7 Hz, 1H), 3.69 – 3.57 (m, 2H), 3.05 – 3.00 (m, 1H), 2.95 – 2.90 (m, 1H); **^{13}C NMR (101 MHz, CDCl$_3$)** δ 165.2, 149.8, 148.2, 147.4, 137.8, 137.4, 129.52, 129.50, 128.8, 126.7, 126.3, 122.3, 117.8, 113.7, 54.8, 42.6, 38.9; **HRMS** (ESI/TOF-Q) m/z: $[M+H]^+$ Calculated for $C_{21}H_{21}N_3OH^+$ 332.1757; Found 332.1753.

N-(3-phenyl-2-(p-tolylamino)propyl)picolinamide (8b):

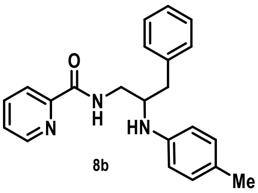
8b

Compound **8b** was synthesized according to GP-3 as pale yellow liquid; eluent (15% ethyl acetate in hexane); **Yield:** 80% (55 mg); **^{1}H NMR (500 MHz, CDCl$_3$)** δ 8.51 – 8.49 (m, 1H), 8.29 (t, *J* = 6.2 Hz, 1H), 8.19 – 8.17 (m, 1H), 7.85 – 7.81 (m, 1H), 7.42 – 7.39 (m, 1H), 7.33 – 7.29 (m, 2H), 7.24 – 7.21 (m, 3H), 7.01 – 6.99 (m, 2H), 6.64 – 6.62 (m, 2H), 3.91 – 3.86 (m, 2H), 3.66 – 3.61 (m, 1H), 3.57 – 3.50 (m, 1H), 3.00 – 2.96 (m, 1H), 2.91 – 2.86 (m, 1H), 2.23 (s, 3H); **^{13}C NMR (126 MHz, CDCl$_3$)** δ 165.1, 149.8, 148.2, 145.0, 137.8, 137.4, 130.0, 129.5, 128.7, 127.1, 126.7, 126.3, 122.3, 113.9, 55.0, 42.6, 38.8, 20.5; **HRMS** (ESI/TOF-Q) m/z: $[M+H]^+$ Calculated for $C_{22}H_{23}N_3OH^+$ 346.1914; Found 346.1912.

N-(2-((4-(tert-butyl)phenyl)amino)-3-phenylpropyl)picolinamide (8c):

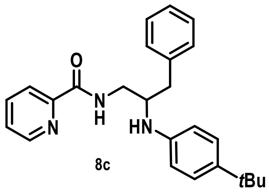

8c

Compound **8c** was synthesized according to GP-3 as pale yellow liquid; eluent (15% ethyl acetate in hexane). **Yield:** 83% (64 mg); **^{1}H NMR (500 MHz, CDCl$_3$)** δ 8.50 – 8.49 (m, 1H), 8.29 – 8.26 (m, 1H), 8.18 (d, *J* = 7.8 Hz, 1H), 7.85 – 7.81 (m, 1H), 7.41 – 7.38 (m, 1H), 7.31 (t, *J* = 7.5 Hz, 2H), 7.25 – 7.21 (m, 5H), 6.68 – 6.67 (m, 2H), 3.92 – 3.87 (m, 2H), 3.64 – 3.54 (m, 2H), 3.01 – 2.98 (m, 1H), 2.90 – 2.85 (m, 1H), 1.28 (s, 9H); **^{13}C NMR (126 MHz, CDCl$_3$)** δ 165.1, 149.8, 148.2, 144.9, 140.6, 137.9, 137.4, 129.5, 128.7, 126.7, 126.32, 126.29, 122.3, 113.4, 54.9, 42.5, 39.0, 34.0, 31.7; **HRMS** (ESI/TOF-Q) m/z: $[M+H]^+$ Calculated for $C_{25}H_{29}N_3OH^+$ 388.2383; Found 388.2387.

N-(2-((4-methoxyphenyl)amino)-3-phenylpropyl)picolinamide (8d):

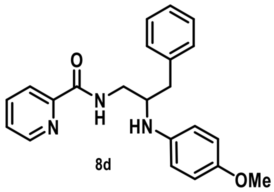

8d

Compound **8d** was synthesized according to GP-3 as pale yellow liquid; eluent (25% ethyl acetate in hexane); **Yield:** 85% (61 mg); **^{1}H NMR (500 MHz, CDCl$_3$)** δ 8.52 – 8.50 (m, 1H), 8.30 – 8.29 (m, 1H), 8.20 – 8.17 (m, 1H), 7.85 – 7.81 (m, 1H), 7.42 – 7.39 (m, 1H), 7.32 – 7.29 (m, 2H), 7.23 (d, *J* = 6.4 Hz, 3H), 6.80 – 6.77 (m, 2H), 6.68 – 6.66 (m, 2H), 3.85 – 3.79 (m, 1H), 3.74 (s, 3H), 3.65 – 3.61 (m, 1H), 3.56 – 3.50 (m, 1H), 2.99 – 2.94 (m, 1H), 2.90 – 2.86 (m, 1H), (signal for NH proton was not detected); **^{13}C NMR (126 MHz, CDCl$_3$)** δ 165.1, 152.5, 149.9, 148.2, 141.4, 137.8, 137.4, 129.5, 128.7, 126.7, 126.3, 122.3, 115.3, 115.2, 55.9, 55.7, 42.7, 38.9; **HRMS** (ESI/TOF-Q) m/z: $[M+H]^+$ Calculated for $C_{22}H_{23}N_3O_2H^+$ 362.1863; Found 362.1860.

N-(2-((4-fluorophenyl)amino)-3-phenylpropyl)picolinamide (8e):

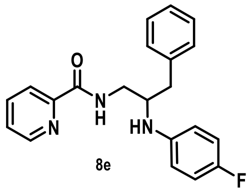

8e

Compound **8e** was synthesized according to GP-3 as brown liquid; eluent (25% ethyl acetate in hexane); **Yield:** 67% (47 mg); **^{1}H NMR (500 MHz, CDCl$_3$)** δ 8.50 (d, *J* = 4.3 Hz, 1H), 8.29 (brs, 1H), 8.18 (d, *J* = 7.7 Hz, 1H), 7.84 (t, *J* = 8.0 Hz, 1H), 7.43 – 7.40 (m, 1H), 7.31 (d, *J* = 7.6 Hz, 2H), 7.24 – 7.21 (m, 3H), 6.87 (t, *J* = 8.1 Hz, 2H), 6.64 – 6.60 (m, 2H), 3.90 – 3.82 (m, 2H), 3.63 – 3.54 (m, 2H), 2.98 – 2.93 (m, 1H), 2.91 – 2.87 (m, 1H); **^{13}C NMR (126 MHz, CDCl$_3$)** ^{13}C NMR (126 MHz, CDCl$_3$) δ 165.2, 156.0 (d, *J* = 235.3 Hz), 149.7, 148.2, 143.7, 137.7, 137.5, 129.5, 128.8, 126.8, 126.4, 122.4, 115.9 (d, *J* = 22.3 Hz), 114.6 (d, *J* = 7.3 Hz), 55.6, 42.7, 38.9; **^{19}F NMR (471 MHz, CDCl$_3$)** δ **-** 127.7; **HRMS** (ESI/TOF-Q) m/z: $[M+H]^+$ Calculated for $C_{21}H_{20}FN_3OH^+$ 350.1663; Found 350.1662.

N-(3-phenyl-2-((4-(trifluoromethyl)phenyl)amino)propyl)picolinamide (8f):

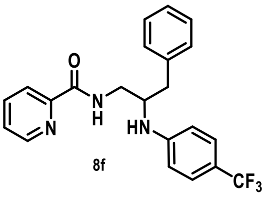

8f

Compound **8f** was synthesized according to GP-3 as pale yellow liquid; eluent (40% ethyl acetate in hexane). **Yield:** 62% (49 mg); **^{1}H NMR (500 MHz, CDCl$_3$)** δ 8.49 (d, *J* = 4.7 Hz, 1H), 8.28 – 8.26 (m, 1H), 8.17 (d, *J* = 7.8 Hz, 1H), 7.86 – 7.82 (m, 1H), 7.43 – 7.40 (m, 1H), 7.37 (d, *J* = 8.5 Hz, 2H), 7.32 (t, *J* = 7.4 Hz, 2H), 7.24 – 7.22 (m, 3H), 6.67 (d, *J* = 8.5 Hz, 2H), 4.56 (d, *J* = 7.9 Hz, 1H), 3.98 – 3.92 (m, 1H), 3.68 – 3.62 (m, 1H), 3.59 – 3.54 (m, 1H), 3.01 – 2.97 (m, 1H), 2.92 – 2.88 (m, 1H); **^{13}C NMR (126 MHz, CDCl$_3$)** δ

165.6, 150.0, 149.5, 148.3, 137.5, 137.4, 129.4, 128.9, 126.9, 126.8 (q, *J* = 3.7 Hz), 126.5, 125.1 (q, *J* = 270.3 Hz), 122.4, 119.0 (q, *J* = 32.6 Hz), 112.5, 55.0, 42.7, 38.7; **^{19}F NMR (471 MHz, CDCl$_3$)** δ -61.0; **HRMS** (ESI/TOF-Q) m/z: [M+H]$^+$ Calculated for $C_{22}H_{20}F_3N_3OH^+$ 400.1631; Found 400.1635.

N-(2-([1,1'-biphenyl]-4-ylamino)-3-phenylpropyl)picolinamide (8g):

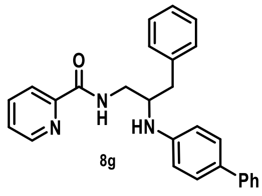
8g

Compound **8g** was synthesized according to GP-3 as brown solid; eluent (15% ethyl acetate in hexane); m.p.: 74 – 76 °C; **Yield:** 76% (62 mg); **^{1}H NMR (400 MHz, CDCl$_3$)** δ 8.48 (d, *J* = 4.8 Hz, 1H), 8.29 (t, *J* = 6.4 Hz, 1H), 8.18 (d, *J* = 7.9 Hz, 1H), 7.83 – 7.79 (m, 1H), 7.52 (d, *J* = 7.8 Hz, 2H), 7.43 (d, *J* = 8.3 Hz, 2H), 7.37 (t, *J* = 7.5 Hz, 3H), 7.33 – 7.30 (m, 2H), 7.24 – 7.22 (m, 4H), 6.76 (d, *J* = 8.4 Hz, 2H), 4.16 (brs, 1H), 3.99 – 3.93 (m, 1H), 3.63 – 3.58 (m, 2H), 3.04 – 2.96 (m, 1H), 2.94 – 2.88 (m, 1H); **^{13}C NMR (101 MHz, CDCl$_3$)** δ 165.3, 149.7, 148.2, 146.8, 141.3, 137.7, 137.4, 130.6, 129.5, 128.77, 128.75, 128.2, 126.8, 126.4, 126.3, 126.2, 122.3, 113.8, 54.9, 42.7, 38.9; **HRMS** (ESI/TOF-Q) m/z: [M+H]$^+$ Calculated for $C_{27}H_{25}N_3OH^+$ 408.2070; Found 408.2080.

N-(3-phenyl-2-(m-tolylamino)propyl)picolinamide (8h):

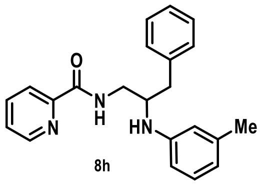
8h

Compound **8h** was synthesized according to GP-3 as pale yellow liquid; eluent (20% ethyl acetate in hexane). **Yield:** 79% (55 mg); **^{1}H NMR (400 MHz, CDCl$_3$)** δ 8.50 (d, *J* = 4.9 Hz, 1H), 8.29 – 8.28 (m, 1H), 8.19 (d, *J* = 7.8 Hz, 1H), 7.85 – 7.81 (m, 1H), 7.42 – 7.39 (m, 1H), 7.31 (t, *J* = 7.5 Hz, 2H), 7.25 – 7.21 (m, 3H), 7.10 – 7.06 (m, 1H), 6.54 – 6.51 (m, 3H), 3.95 – 3.89 (m, 2H), 3.67 – 3.61 (m, 1H), 3.59 – 3.52 (m, 1H), 3.02 – 2.97 (m, 1H), 2.91 – 2.86 (m, 1H), 2.27 (s, 3H); **^{13}C NMR (101 MHz, CDCl$_3$)** δ 165.2, 149.8, 148.2, 147.4, 139.3, 137.8, 137.4, 129.5, 129.4, 128.7, 126.7, 126.3, 122.4, 118.7, 114.5, 110.7, 54.8, 42.6, 38.9, 21.8. **HRMS** (ESI/TOF-Q) m/z: [M+H]$^+$ Calculated for $C_{22}H_{23}N_3OH^+$ 346.1914; Found 346.1929.

N-(2-((3-methoxyphenyl)amino)-3-phenylpropyl)picolinamide (8i):

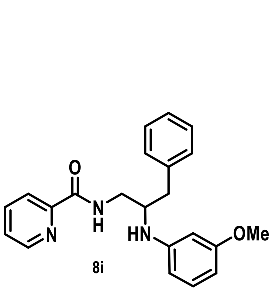
8i

Compound **8i** was synthesized according to GP-3 as colourless sticky liquid; eluent (20% ethyl acetate in hexane); **Yield:** 81% (58 mg); **^{1}H NMR (500 MHz, CDCl$_3$)** δ 8.53 (d, *J* = 4.9 Hz, 1H), 8.31 (brs, 1H), 8.22 – 8.20 (m, 1H), 7.86 (t, *J* = 7.9 Hz, 1H), 7.44 – 7.41 (m, 1H), 7.34 – 7.32 (m, 2H), 7.29 – 7.26 (m, 3H), 7.13 – 7.09 (m, 1H), 6.34 (d, *J* = 8.2 Hz, 1H), 6.30 (d, *J* = 8.5 Hz, 2H), 4.10 (brs, 1H), 3.94 – 3.92 (m, 1H), 3.79 (s, 3H), 3.67 – 3.58 (m, 2H), 3.04 – 3.00 (m, 1H), 2.95 – 2.90 (m, 1H); **^{13}C NMR (126 MHz, CDCl$_3$)** δ 165.2, 161.0, 149.8, 148.7, 148.2, 137.7, 137.4, 130.2, 129.5, 128.7, 126.7, 126.3, 122.3, 106.6, 103.0, 99.6, 55.2, 54.9, 42.6, 38.8; **HRMS** (ESI/TOF-Q) m/z: [M+H]$^+$ Calculated for $C_{22}H_{23}N_3O_2H^+$ 362.1863; Found 362.1883.

N-(2-((3-chlorophenyl)amino)-3-phenylpropyl)picolinamide (8j):

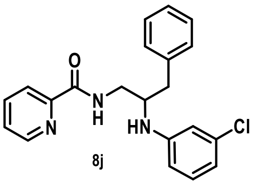

8j

Compound **8j** was synthesized according to GP-3 as pale yellow liquid; eluent (20% ethyl acetate in hexane); **Yield: 70**% (51 mg); **^{1}H NMR (500 MHz, CDCl$_3$)** δ 8.51 – 8.50 (m, 1H), 8.26 (t, *J* = 6.6 Hz, 1H), 8.19 – 8.17 (m, 1H), 7.86 – 7.82 (m, 1H), 7.43 – 7.40 (m, 1H), 7.34 – 7.30 (m, 2H), 7.25 – 7.22 (m, 3H), 7.05 (t, *J* = 7.9 Hz, 1H), 6.65 – 6.62 (m, 2H), 6.55 – 6.53 (m, 1H), 4.23 – 4.21 (m, 1H), 3.90 – 3.85 (m, 1H), 3.59 (t, *J* = 6.3 Hz, 2H), 2.99 – 2.95 (m, 1H), 2.91 – 2.87 (m, 1H); **^{13}C NMR (126 MHz, CDCl$_3$)** δ 165.4, 149.6, 148.6 (2xC), 148.2, 137.5, 135.2, 130.4, 129.5, 128.8, 126.9, 126.4, 122.4, 117.5, 113.2, 111.7, 55.0, 42.6, 38.7; **HRMS** (ESI/TOF-Q) m/z: $[M+H]^+$ Calculated for $C_{21}H_{20}ClN_3OH^+$ 366.1368; Found 366.1363.

N-(2-((3-bromophenyl)amino)-3-phenylpropyl)picolinamide (8k):

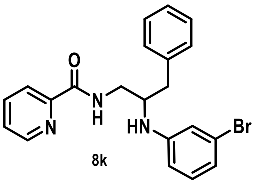

8k

Compound **8k** was synthesized according to GP-3 as pale yellow liquid; eluent (20% ethyl acetate in hexane); **Yield:** 55% (45 mg); **^{1}H NMR (500 MHz, CDCl$_3$)** δ 8.51 – 8.50 (m, 1H), 8.27 (t, *J* = 6.5 Hz, 1H), 8.19 – 8.17 (m, 1H), 7.87 – 7.81 (m, 1H), 7.44 – 7.40 (m, 1H), 7.34 – 7.30 (m, 2H), 7.25 – 7.22 (m, 3H), 6.99 (t, *J* = 8.0 Hz, 1H), 6.80 – 6.77 (m, 2H), 6.59 – 6.57 (m, 1H), 4.22 (brs, 1H), 3.90 – 3.85 (m, 1H), 3.59 (t, *J* = 6.2 Hz, 2H), 2.99 – 2.95 (m, 1H), 2.90 – 2.86 (m, 1H); **^{13}C NMR (126 MHz, CDCl$_3$)** δ 165.4, 149.6, 148.7 (2xC), 148.2, 137.5, 130.7, 129.5, 128.8, 126.9, 126.4, 123.5, 122.4, 120.3, 116.1, 112.1, 55.0, 42.6, 38.7; **HRMS** (ESI/TOF-Q) m/z: $[M+H]^+$ Calculated for $C_{21}H_{20}BrN_3OH^+$ 410.0863; Found 410.0867.

N-(2-((3,4-dimethoxyphenyl)amino)-3-phenylpropyl)picolinamide (8l):

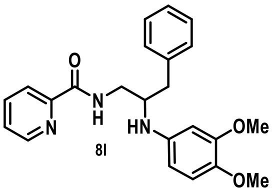

8l

Compound **8l** was synthesized according to GP-3 as pale yellow liquid; eluent (40% ethyl acetate in hexane); **Yield:** 63% (49 mg); **^{1}H NMR (500 MHz, CDCl$_3$)** δ 8.51 – 8.50 (m, 1H), 8.31 (t, *J* = 6.3 Hz, 1H), 8.18 (d, *J* = 7.8 Hz, 1H), 7.85 – 7.82 (m, 1H), 7.42 – 7.39 (m, 1H), 7.32 – 7.29 (m, 2H), 7.24 – 7.21 (m, 3H), 6.74 (d, *J* = 8.5 Hz, 1H), 6.35 (d, *J* = 2.6 Hz, 1H), 6.31 – 6.29 (m, 1H), 3.87 – 3.78 (m, 8H), 3.63 – 3.55 (m, 2H), 3.01 – 2.97 (m, 1H), 2.92 – 2.88 (m, 1H); **^{13}C NMR (126 MHz, CDCl$_3$)** δ 165.3, 150.2 (2xC), 149.7, 148.2, 137.7 (2xC), 137.5, 129.5, 128.8, 126.8, 126.4, 122.3, 113.3, 105.2, 100.4, 56.8, 56.3, 55.9, 42.7, 38.8; **HRMS** (ESI/TOF-Q) m/z: $[M+H]^+$ Calculated for $C_{23}H_{25}N_3O_3H^+$ 392.1969; Found 392.1969.

N-(2-((4-chloro-3-fluorophenyl)amino)-3-phenylpropyl)picolinamide (8m):

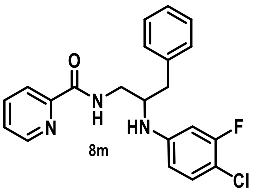

8m

Compound **8m** was synthesized according to GP-3 as pale yellow liquid; eluent (30% ethyl acetate in hexane); **Yield:** 69% (53 mg); **^{1}H NMR (500 MHz, CDCl$_3$)** δ 8.51 – 8.50 (m, 1H), 8.27 (t, *J* = 6.5 Hz, 1H), 8.18 (d, *J* = 7.8 Hz, 1H), 7.86 – 7.83 (m, 1H), 7.43 – 7.40 (m, 1H), 7.32 (t, *J* = 7.5 Hz, 2H), 7.24 – 7.21 (m, 3H), 6.90 (t, *J* = 8.8 Hz, 1H), 6.65 – 6.64 (m, 1H), 6.49 – 6.46 (m, 1H), 4.05 (brs, 1H), 3.83 – 3.78 (m, 1H), 3.58 (t, *J* = 6.2 Hz, 2H), 2.96 – 2.86 (m, 2H); **^{13}C NMR (126 MHz, CDCl$_3$)** **^{13}C NMR (126 MHz, CDCl$_3$)** δ 165.4,

151.1 (d, *J* = 237.6 Hz), 149.6, 148.2, 144.4 (d, *J* = 1.6 Hz), 137.53, 137.47, 129.4, 128.9, 126.9, 126.5, 122.4, 121.3 (d, *J* = 18.3 Hz), 117.0 (d, *J* = 21.9 Hz), 114.6, 112.7 (d, *J* = 6.2 Hz), 55.7, 42.8, 38.9; **^{19}F NMR (471 MHz, CDCl$_3$)** δ -131.1; **HRMS** (ESI/TOF-Q) m/z: [M+H]$^+$ Calculated for $C_{21}H_{19}ClFN_3OH^+$ 384.1273; Found 384.1272.

N-(3-phenyl-2-((3,4,5-trimethoxyphenyl)amino)propyl)picolinamide (8n):

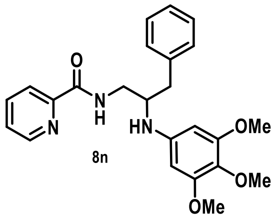

8n

Compound **8n** was synthesized according to GP-3 as pale yellow liquid; eluent (40% ethyl acetate in hexane); **Yield:** 54% (45 mg); **^{1}H NMR (500 MHz, CDCl$_3$)** δ 8.50 (d, *J* = 4.5 Hz, 1H), 8.30 (t, *J* = 6.4 Hz, 1H), 8.18 (d, *J* = 7.8 Hz, 1H), 7.85 – 7.82 (m, 1H), 7.42 – 7.40 (m, 1H), 7.31 (t, *J* = 7.5 Hz, 2H), 7.24 (d, *J* = 7.5 Hz, 3H), 5.92 (s, 2H), 3.87 – 3.84 (m, 2H), 3.80 (s, 6H), 3.74 (s, 3H), 3.63 – 3.58 (m, 2H), 2.99 – 2.95 (m, 1H), 2.93 – 2.88 (m, 1H); **^{13}C NMR (126 MHz, CDCl$_3$)** δ 165.3, 154.1, 149.7, 148.2, 144.2, 137.8, 137.5, 130.3, 129.5, 128.8, 126.8, 126.4, 122.3, 91.3, 61.2, 56.1, 55.6, 43.0, 39.3; **HRMS** (ESI/TOF-Q) m/z: [M+H]$^+$ Calculated for $C_{24}H_{27}N_3O_4H^+$ 422.2074; Found 422.2089.

N-(2-((4-acetylphenyl)amino)-3-phenylpropyl)picolinamide (8o):

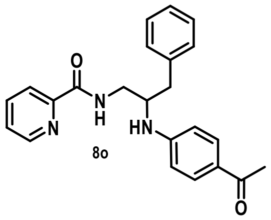

8o

Compound **8o** was synthesized according to GP-3 as pale yellow liquid; eluent (35% ethyl acetate in hexane); **Yield:** 66% (49 mg); **^{1}H NMR (400 MHz, CDCl$_3$)** δ 8.53 – 8.52 (m, 1H), 8.35 – 8.32 (m, 1H), 8.22 – 8.20 (m, 1H), 7.89 – 7.81 (m, 3H), 7.46 – 7.43 (m, 1H), 7.37 – 7.33 (m, 2H), 7.30 – 7.26 (m, 3H), 6.68 – 6.65 (m, 2H), 4.95 (d, *J* = 7.6 Hz, 1H), 4.06 – 4.01 (m, 1H), 3.74 – 3.57 (m, 2H), 3.07– 2.91 (m, 2H), 2.50 (s, 3H); **^{13}C NMR (101 MHz, CDCl$_3$)** δ 196.4, 165.7, 151.6, 149.5, 148.2, 137.5, 137.3, 131.0, 129.4, 128.9, 126.93, 126.91, 126.5, 122.4, 112.0, 55.0, 42.6, 38.7, 26.1. **HRMS** (ESI/TOF-Q) m/z: [M+H]$^+$ Calculated for $C_{23}H_{23}N_3O_2H^+$ 374.1863; Found 374.1879.

N-(2-((4-cyanophenyl)amino)-3-phenylpropyl)picolinamide (8p):

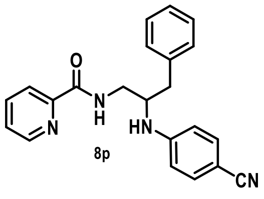

8p

Compound **8p** was synthesized according to GP-3 as yellow liquid; eluent (30% ethyl acetate in hexane); **Yield:** 61% (43 mg); **^{1}H NMR (500 MHz, CDCl$_3$)** δ 8.51(d, *J* = 4.4 Hz, 1H), 8.30 (brs, 1H), 8.18 (d, *J* = 7.9 Hz, 1H), 7.87 (t, *J* = 7.8 Hz, 1H), 7.48 – 7.43 (m, 1H), 7.41 – 7.37 (m, 2H), 7.33 – 7.32 (m, 2H), 7.28 – 7.27 (m, 1H), 7.23 (d, *J* = 7.8 Hz, 2H), 6.63 – 6.61 (m, 2H), 4.98 (d, *J* = 7.6 Hz, 1H), 3.97 – 3.93 (m, 1H), 3.72 – 3.66 (m, 1H), 3.57 – 3.52 (m, 1H), 3.02 – 2.97 (m, 1H), 2.93 – 2.88 (m, 1H); **^{13}C NMR (126 MHz, CDCl$_3$)** δ 165.8, 150.8, 149.4, 148.3, 137.6, 137.2, 133.9, 129.3, 128.9, 127.0, 126.6, 122.4, 120.5, 112.8, 98.9, 55.2, 42.7, 38.7; **HRMS** (ESI/TOF-Q) m/z: [M+H]$^+$ Calculated for $C_{22}H_{20}N_4OH^+$ 357.1710; Found 357.1730.

Methyl 4-((1-phenyl-3-(picolinamido)propan-2-yl)amino)benzoate (8q):

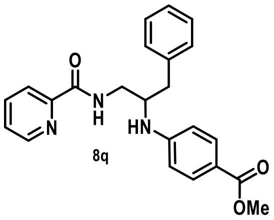

8q

Compound **8q** was synthesized according to GP-3 as pale yellow liquid; eluent (35% ethyl acetate in hexane); **Yield:** 65% (51 mg); **^{1}H NMR (500 MHz, CDCl$_3$)** δ 8.51 (d, *J* = 3.2 Hz, 1H), 8.31 (t, *J* = 6.5 Hz, 1H), 8.20 (d, *J* = 7.8 Hz, 1H), 7.87 – 7.84 (m, 3H), 7.44 – 7.42 (m, 1H), 7.35 – 7.32 (m, 2H), 7.28 (d, *J* = 5.4 Hz, 1H), 7.26 – 7.24 (m, 2H), 6.64 (d, *J* = 8.4 Hz, 2H), 4.79 (d, *J* = 7.8 Hz, 1H), 4.04 – 3.97 (m, 1H), 3.85 (s, 3H), 3.70 – 3.58 (m, 2H), 3.04 – 3.00 (m, 1H), 2.94 – 2.90 (m, 1H); **^{13}C NMR (126 MHz, CDCl$_3$)** δ 167.4, 165.6, 151.3, 149.5, 148.2, 137.5, 137.4, 131.7, 129.4, 128.9, 126.9, 126.5, 122.4, 118.6, 112.1, 54.9, 51.6, 42.6, 38.7; **HRMS** (ESI/TOF-Q) m/z: [M+H]$^+$ Calculated for $C_{23}H_{23}N_3O_3H^+$ 390.1812; Found 390.1837.

N-(2-(naphthalen-1-ylamino)-3-phenylpropyl)picolinamide (8r):

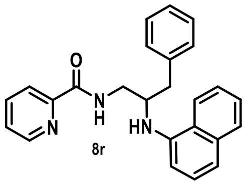

8r

Compound **8r** was synthesized according to GP-3 as brown liquid; eluent (20% ethyl acetate in hexane); **Yield:** 80% (61 mg); **^{1}H NMR (500 MHz, CDCl$_3$)** δ 8.42 (d, *J* = 4.7 Hz, 1H), 8.33 (d, *J* = 6.6 Hz, 1H), 8.16 (d, *J* = 8.0 Hz, 1H), 7.83 (d, *J* = 7.3 Hz, 1H), 7.77 – 7.72 (m, 2H), 7.40 – 7.39 (m, 2H), 7.34 – 7.30 (m, 3H), 7.28 – 7.25 (m, 3H), 7.22 – 7.17 (m, 2H), 6.76 (d, *J* = 7.6 Hz, 1H), 5.20 (brs, 1H), 4.06 – 4.05 (m, 1H), 3.81 – 3.75 (m, 1H), 3.63 – 3.58 (m, 1H), 3.17 – 3.14 (m, 1H), 2.93 – 2.88 (m, 1H); **^{13}C NMR (126 MHz, CDCl$_3$)** δ 165.8, 149.6, 148.2, 142.5, 137.9, 137.4, 134.7, 129.5, 128.8, 128.6, 126.8, 126.7, 126.4, 125.8, 124.9, 123.7, 122.4, 120.5, 117.3, 104.3, 55.5, 42.4, 38.2; **HRMS** (ESI/TOF-Q) m/z: [M+H]$^+$ Calculated for $C_{25}H_{23}N_3OH^+$ 382.1914; Found 382.1915.

N-(2-((2-methoxyphenyl)amino)-3-phenylpropyl)picolinamide (8s):

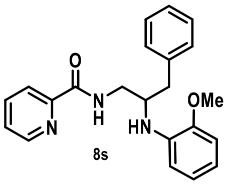

8s

Compound **8s** was synthesized according to GP-3 as pale yellow liquid; eluent (20% ethyl acetate in hexane); **Yield:** 73% (53 mg); **^{1}H NMR (500 MHz, CDCl$_3$)** δ 8.53 (d, *J* = 5.0 Hz, 1H), 8.32 (brs, 1H), 8.23 – 8.21 (m, 1H), 7.87 – 7.84 (m, 1H), 7.44 – 7.41 (m, 1H), 7.34 – 7.29 (m, 3H), 7.26 – 7.22 (m, 2H), 6.93 – 6.90 (m, 1H), 6.86 – 6.81 (m, 2H), 6.74 – 6.70 (m, 1H), 4.43 (brs, 1H), 4.00 – 3.96 (m, 1H), 3.86 (s, 3H), 3.75 – 3.69 (m, 1H), 3.60 – 3.54 (m, 1H), 3.03 – 2.92 (m, 2H); **^{13}C NMR (126 MHz, CDCl$_3$)** δ 164.9, 149.9, 148.2, 147.3, 137.9, 137.4, 137.3, 129.5, 128.6, 126.6, 126.2, 122.3, 121.5, 117.1, 111.1, 110.1, 55.7, 54.3, 42.7, 39.0; **HRMS** (ESI/TOF-Q) m/z: [M+H]$^+$ Calculated for $C_{22}H_{23}N_3O_2H^+$ 362.1863; Found 362.1882.

N-(3-(4-ethoxyphenyl)-2-(p-tolylamino)propyl)picolinamide (8t):

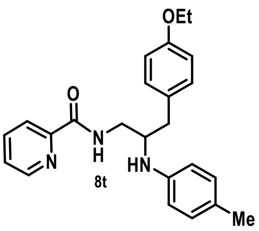

8t

Compound **8t** was synthesized according to GP-3 as pale yellow liquid; eluent (20% ethyl acetate in hexane); **Yield:** 86% (67 mg); **^{1}H NMR (400 MHz, CDCl$_3$)** δ 8.49 (d, *J* = 4.8 Hz, 1H), 8.27 (t, *J* = 6.1 Hz, 1H), 8.18 (d, *J* = 7.9 Hz, 1H), 7.82 (t, *J* = 7.8 Hz, 1H), 7.41 – 7.38 (m, 1H), 7.13 (d, *J* = 8.2 Hz, 2H), 7.00 (d, *J* = 8.0 Hz, 2H), 6.84 (d, *J* = 8.1 Hz, 2H), 6.63 (d, *J* = 8.0 Hz, 2H), 4.04 –3.97 (m, 2H), 3.87 – 3.80 (m, 2H), 3.66 – 3.60 (m, 1H), 3.55 – 3.48 (m, 1H), 2.93 – 2.88 (m, 1H), 2.85 – 2.80 (m, 1H), 2.23 (s, 3H), 1.41 (t, *J* = 7.0 Hz, 3H); **^{13}C NMR (101 MHz, CDCl$_3$)** δ 165.1, 157.8, 149.9, 148.2, 145.1, 137.4,

130.5, 130.0, 129.6, 127.0, 126.2, 122.3, 114.8, 113.9, 63.6, 55.1, 42.6, 37.9, 20.5, 15.0; **HRMS** (ESI/TOF-Q) m/z: $[M+H]^+$ Calculated for $C_{24}H_{27}N_3O_2H^+$ 390.2176; Found 390.2180.

(E)-N-(5-phenyl-2-(p-tolylamino)pent-4-en-1-yl)picolinamide (8u):

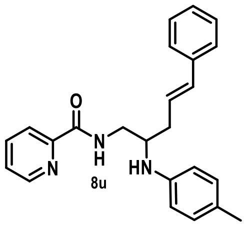

8u

Compound **8u** was synthesized according to GP-3 as pale yellow liquid; eluent (15% ethyl acetate in hexane); **Yield:** 89% (66 mg); **^{1}H NMR (500 MHz, CDCl$_3$)** δ 8.50 – 8.48 (m, 1H), 8.35 (d, *J* = 7.1 Hz, 1H), 8.21 – 8.19 (m, 1H), 7.83 (t, *J* = 7.8 Hz, 1H), 7.42 – 7.38 (m, 1H), 7.33 (d, *J* = 7.5 Hz, 2H), 7.30 – 7.27 (m, 2H), 7.22 – 7.19 (m, 1H), 7.00 – 6.99 (m, 2H), 6.65 – 6.62 (m, 2H), 6.49 (d, *J* = 15.7 Hz, 1H), 6.30 – 6.23 (m, 1H), 3.82 (brs, 1H), 3.77 – 3.74 (m, 1H), 3.68 (d, *J* = 6.3 Hz, 2H), 2.55 (d, *J* = 7.1 Hz, 2H), 2.23 (s, 3H); **^{13}C NMR (126 MHz, CDCl$_3$)** δ 165.1, 149.8, 148.2, 145.1, 137.5, 137.3, 133.4, 130.0, 128.6, 127.4, 127.1, 126.3 (2xC), 125.8, 122.4, 114.0, 54.1, 42.9, 36.6, 20.5; **HRMS** (ESI/TOF-Q) m/z: $[M+H]^+$ Calculated for $C_{24}H_{25}N_3OH^+$ 372.2070; Found 372.2072.

N-(2-(methyl(phenyl)amino)-3-phenylpropyl)picolinamide (8v):

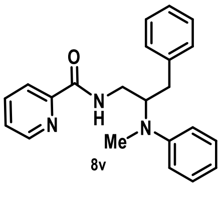

8v

Compound **8v** was synthesized according to GP-3 as pale yellow liquid; eluent (15% ethyl acetate in hexane); **Yield:** 77% (53 mg); **^{1}H NMR (500 MHz, CDCl$_3$^{1}H NMR (500 MHz, CDCl$_3$)** δ 8.43 – 8.41 (m, 1H), 8.13 – 8.11 (m, 2H), 7.81 – 7.77 (m, 1H), 7.37 – 7.34 (m, 1H), 7.23 (d, *J* = 7.5 Hz, 2H), 7.19 – 7.16 (m, 5H), 6.83 – 6.81 (m, 2H), 6.70 (t, *J* = 7.3 Hz, 1H), 4.37 – 4.31 (m, 1H), 3.78 – 3.73 (m, 1H), 3.62 – 3.56 (m, 1H), 2.98 – 2.93 (m, 1H), 2.86 (s, 3H), 2.83 – 2.80 (m, 1H); **13C NMR (126 MHz, CDCl3)** δ 163.6, 149.9, 148.8, 147.2, 137.5, 136.3, 128.2, 128.1, 127.7, 125.5, 125.1, 121.2, 116.7, 113.5, 59.8, 39.5, 35.6, 29.9; **HRMS** (ESI/TOF-Q) m/z: $[M+H]^+$ Calculated for $C_{22}H_{23}N_3OH^+$ 346.1914; Found 346.1927.

N-(3-(4-ethoxyphenyl)-2-(methyl(phenyl)amino)propyl)picolinamide (8w):

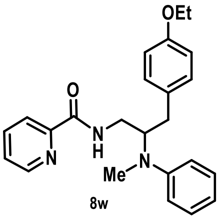

8w

Compound **8w** was synthesized according to GP-3 as pale yellow liquid; eluent (20% ethyl acetate in hexane); **Yield:** 81% (63 mg); **^{1}H NMR (400 MHz, CDCl$_3$)** δ 8.45 – 8.41 (m, 1H), 8.19 – 8.11 (m, 2H), 7.78 (t, *J* = 7.7 Hz, 1H), 7.35 (t, *J* = 6.2 Hz, 1H), 7.17 (t, *J* = 7.7 Hz, 2H), 7.08 (d, *J* = 8.1 Hz, 2H), 6.83 – 6.76 (m, 4H), 6.70 (t, *J* = 7.4 Hz, 1H), 4.32 – 4.23 (m, 1H), 4.01 – 3.95 (m, 2H), 3.78 – 3.72 (m, 1H), 3.61 – 3.54 (m, 1H), 2.95 – 2.85 (m, 4H), 2.79 – 2.73 (m, 1H), 1.42 – 1.36 (m, 3H); **^{13}C NMR (126 MHz, CDCl$_3$)** δ 164.5, 157.6, 150.9, 149.8, 148.1, 137.3, 130.4, 130.0, 129.2, 126.1, 122.1, 117.6, 114.7, 114.4, 63.5, 60.8, 40.5, 35.7, 30.9, 15.0; **HRMS** (ESI/TOF-Q) m/z: $[M+H]^+$ Calculated for $C_{24}H_{27}N_3O_2H^+$ 390.2176; Found 390.2176.

(E)-N-(2-(methyl(phenyl)amino)-5-phenylpent-4-en-1-yl)picolinamide (8x):

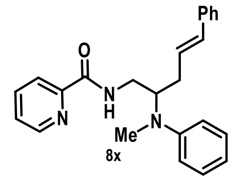

8x

Compound **8x** was synthesized according to GP-3 as pale yellow liquid; eluent (15% ethyl acetate in hexane); **Yield:** 85% (63 mg); **^{1}H NMR (500 MHz, CDCl$_3$)** δ 8.44 – 8.43 (m, 1H), 8.25 – 8.22 (m, 1H), 8.18 – 8.16 (m, 1H), 7.82 – 7.79 (m, 1H), 7.38 – 7.35 (m, 1H), 7.25 – 7.16 (m, 7H), 6.90 – 6.88 (m, 2H), 6.74 – 6.71 (m, 1H), 6.45 (d, *J* = 15.8 Hz, 1H), 6.14 – 6.08 (m, 1H), 4.27 – 4.21 (m, 1H), 3.89 – 3.84 (m, 1H), 3.59 – 3.54 (m, 1H), 2.87 (s, 3H), 2.63 – 2.57 (m, 1H), 2.53 – 2.48 (m, 1H); **^{13}C NMR (126 MHz, CDCl$_3$)** δ 164.6, 151.1, 149.8, 148.2, 137.4, 137.3, 132.3, 129.3, 128.5, 127.2, 126.8, 126.23, 126.17, 122.2, 117.7, 114.5, 59.3, 40.9, 34.3, 30.6; **HRMS** (ESI/TOF-Q) m/z: $[M+H]^+$ Calculated for $C_{24}H_{25}N_3OH^+$ 372.2070; Found 372.2093.

((1-amino-3-phenylpropan-2-yl)imino)diphenyl-l6-sulfanone (9a):

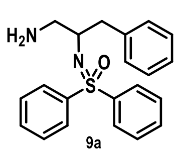

9a

Compound **9a** was obtained as pale yellow liquid; eluent (10% MeOH in DCM); **Yield:** 89% (93 mg); **^{1}H NMR (400 MHz, CDCl$_3$)** δ 7.97 (d, *J* = 7.6 Hz, 2H), 7.52 – 7.43 (m, 3H), 7.41 – 7.37 (t, *J* = 7.3 Hz, 1H), 7.34 – 7.30 (m, 2H), 7.27 – 7.22 (m, 5H), 7.18 (d, *J* = 7.2 Hz, 2H), 4.25 (brs, 2H), 3.37 – 3.31 (m, 1H), 3.04 – 3.00 (m, 1H), 2.91 – 2.82 (m, 3H); **^{13}C NMR (101 MHz, CDCl$_3$)** δ 140.8, 139.44, 139.37, 132.8, 132.4, 130.2, 129.22, 129.20, 129.0, 128.5, 128.4, 126.4, 58.0, 47.2, 42.6; **HRMS** (ESI/TOF-Q) m/z: $[M+H]^+$ Calculated for $C_{21}H_{22}N_2OSH^+$ 351.1526; Found 351.1531.

3-(4-ethoxyphenyl)-N2-(p-tolyl)propane-1,2-diamine (9b):

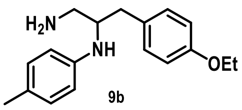

9b

Compound **9b** was obtained as pale yellow liquid; eluent (10% MeOH in DCM); **Yield:** 90% (77 mg); **^{1}H NMR (400 MHz, CDCl$_3$)** δ 7.03 (d, *J* = 8.6 Hz, 2H), 6.98 (d, *J* = 8.1 Hz, 2H), 6.81 (d, *J* = 8.6 Hz, 2H), 6.60 (d, *J* = 8.3 Hz, 2H), 4.00 (q, *J* = 6.9 Hz, 2H), 3.68 – 3.62 (m, 1H), 3.39 (brs, 3H), 2.88 – 2.79 (m, 2H), 2.68 – 2.56 (m, 2H), 2.23 (s, 3H), 1.40 (t, *J* = 7.0 Hz, 3H); **^{13}C NMR (101 MHz, CDCl$_3$)** δ 157.7, 145.1, 130.4, 130.0, 129.7, 127.1, 114.7, 114.2, 63.5, 55.6, 43.7, 37.4, 20.5, 15.0; **HRMS** (ESI/TOF-Q) m/z: $[M+H]^+$ Calculated for $C_{18}H_{24}N_2OH^+$ 285.1961; Found 285.1974.

2-(1,3-dioxoisoindolin-2-yl)-N-(2-((oxodiphenyl-l6-sulfaneylidene)amino)-3-phenylpropyl)-3-phenylpropanamide (10):

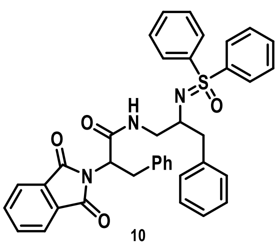

10
dr- 1:1

Compound **10** was obtained as pale yellow liquid; eluent (30% ethyl acetate in hexane); **Yield:** 74% (70 mg); **^{1}H NMR (500 MHz, CDCl$_3$)** δ 7.80 – 7.75 (m, 6H), 7.70 – 7.64 (m, 4H), 7.58 (d, *J* = 8.0 Hz, 2H), 7.51 – 7.37 (m, 6H), 7.35 – 7.28 (m, 10H), 7.21 – 7.14 (m, 14H), 7.12 – 7.08 (m, 4H), 6.95 (d, *J* = 7.8 Hz, 2H), 6.82 (d, *J* = 7.7 Hz, 2H), 5.20 – 5.13 (m, 2H), 3.98 – 3.89 (m, 2H), 3.75 – 3.69 (m, 2H), 3.65 – 3.59 (m, 2H), 3.24 – 3.15 (m, 2H), 3.06 – 2.97 (m, 2H), 2.91 – 2.87 (m, 2H), 2.85 – 2.79 (m, 2H); **^{13}C NMR (126 MHz, CDCl$_3$)** δ 168.4, 168.3, 168.14, 168.08, 140.7 (2xC), 139.6, 139.4, 139.1, 138.8, 137.8, 137.6 (2xC), 133.8, 133.7, 132.74, 132.66, 132.4, 132.3, 132.2, 130.4, 130.3, 129.2, 129.14, 129.11, 129.08, 129.04, 128.98, 128.9, 128.8, 128.6 (2xC), 128.44, 128.39,

128.21, 128.17, 126.71, 126.68, 126.48, 126.45, 123.29, 123.26, 57.9, 57.4, 55.5, 55.4, 46.3, 46.0, 43.0, 42.9, 34.5, 34.4.; **HRMS** (ESI/TOF-Q) m/z: $[M+H]^+$ Calculated for $C_{38}H_{33}N_3O_4SH^+$ 628.2265; Found 628.2275; **dr-1:1**.

5-(4-ethoxybenzyl)-1-(p-tolyl)imidazolidin-2-one (11):

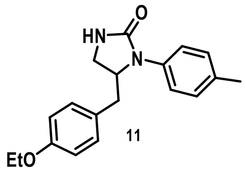

11

Compound **11** was obtained as white solid; eluent (30% ethyl acetate in hexane); m.p.: 148 – 150 °C; **Yield:** 95% (88 mg); **^{1}H NMR (500 MHz, $CDCl_3$)** δ 7.38 (d, *J* = 8.4 Hz, 2H), 7.20 (d, *J* = 8.2 Hz, 2H), 7.03 (d, *J* = 8.6 Hz, 2H), 6.82 (d, *J* = 8.6 Hz, 2H), 5.09 – 5.00 (m, 1H), 4.49 – 4.44 (m, 1H), 4.00 (q, *J* = 7.0 Hz, 2H), 3.46 – 3.42 (m, 1H), 3.28 – 3.25 (m, 1H), 3.02 – 2.99 (m, 1H), 2.69 – 2.65 (m, 1H), 2.34 (s, 3H), 1.40 (t, *J* = 7.0 Hz, 3H); **^{13}C NMR (126 MHz, $CDCl_3$)** δ 160.3, 158.0, 135.8, 134.1, 130.3, 129.8, 128.5, 122.3, 114.8, 63.6, 58.2, 42.7, 37.3, 21.0, 15.0; **HRMS** (ESI/TOF-Q) m/z: $[M+H]^+$ Calculated for $C_{19}H_{22}N_2O_2H^+$ 311.1754; Found 311.1743.

Diphenyl((5-(1-phenyl-3-(triisopropylsilyl)prop-2-yn-1-yl)-1-picolinoylpyrrolidin -3-yl)imino)-l6-sulfanone (12):

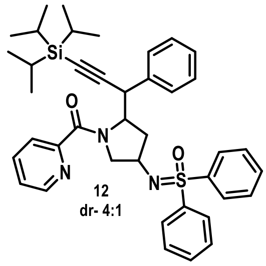

12
dr- 4:1

Compound **12** was obtained as brown liquid; eluent (30% ethyl acetate in hexane); **Yield:** 68% (67 mg); **^{1}H NMR (400 MHz, $CDCl_3$)** δ 8.58 – 8.57 (m, 5.07 H), 7.94 – 7.83 (m, 20.77 H), 7.78 – 7.73 (m, 5.08 H), 7.66 – 7.64 (m, 4.97 H), 7.53 – 7.48 (m, 8.23 H), 7.46 – 7.39 (m, 32.67 H), 7.36 – 7.28 (m, 20.89 H), 4.98 – 4.96 (m, 5.01 H), 4.67 – 4.62 (m, 4.97 H), 3.58 – 3.54 (m, 5.03 H), 3.45 – 3.41 (m, 4.07 H), 3.37 **-** 3.35 (m, 1.05 H) 2.75 – 2.70 (m, 4.06 H), 2.67 – 2.64 (m, 1.04 H), 2.40 – 2.34 (m, 4.06 H), 2.33 – 2.32 (m, 1H), 2.04 – 2.02 (m, 1.06H), 1.97 – 1.91 (m, 4.05H), 1.14 – 1.04 (m, 105.82 H); **^{13}C NMR (126 MHz, $CDCl_3$)** δ 166.4, 153.8, 153.3, 147.7, 147.5, 140.1, 140.0, 136.0, 135.7, 135.6, 135.4, 131.7, 131.6, 131.5, 131.4, 129.1, 128.3, 128.23, 128.18, 128.13, 128.08, 127.7, 127.6, 127.34, 127.26, 127.2, 127.0, 126.3, 126.0, 123.8, 123.6, 122.7, 122.6, 106.5, 84.0, 83.7, 61.4, 59.7, 57.0, 56.5, 50.9, 50.8, 37.8, 37.1, 34.6, 33.8, 17.9, 10.5; **HRMS** (ESI/TOF-Q) m/z: $[M+H]^+$ Calculated for $C_{40}H_{47}N_3O_2SSiH^+$ 662.3231; Found 662.3236; **dr-4:1.**

4,4'-sulfonylbis(N-benzylaniline) (14):

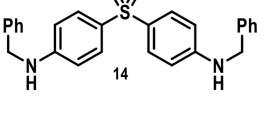

14

Compound **14** was obtained as brown solid; eluent (30% ethyl acetate in hexane); **Yield:** 75% (64 mg); **^{1}H NMR (400 MHz, $CDCl_3$)** δ 7.65 (d, *J* = 8.5 Hz, 4H), 7.36 – 7.28 (m, 10H), 6.56 (d, *J* = 8.5 Hz, 4H), 4.60 (brs, 2H), 4.33 (s, 4H); **^{13}C NMR (101 MHz, $CDCl_3$)** δ 151.3, 138.2, 130.3, 129.2, 128.9, 127.7, 127.4, 112.1, 47.7. **HRMS** (ESI/TOF-Q) m/z: $[M+H]^+$ Calculated for $C_{26}H_{24}N_2O_2SH^+$ 429.1631; Found 429.1625.

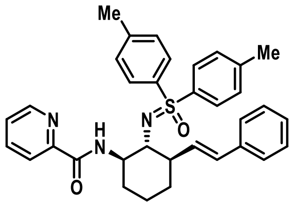

(±)16
dr> 20:1

N-((1R,2R,3R)-2-((oxodi-p-tolyl-l6-sulfaneylidene)amino)-3-((E)-styryl) cyclohexyl) picolinamide (16):

Compound **16** was obtained as brown gummy solid; eluent (30% ethyl acetate in hexane); **Yield:** 76% (83 mg); **^{1}H NMR (500 MHz, CDCl$_3$)** δ 8.51 (d, *J* = 4.7 Hz, 1H), 8.16 (d, *J* = 7.8 Hz, 1H), 8.11 (d, *J* = 8.7 Hz, 1H), 7.80 – 7.77 (m, 1H), 7.67 (d, *J* = 8.0 Hz, 2H), 7.58 (d, *J* = 7.9 Hz, 2H), 7.36 – 7.28 (m, 5H), 7.19 (t, *J* = 7.1 Hz, 1H), 6.96 (t, *J* = 8.2 Hz, 4H), 6.47 (d, *J* = 15.9 Hz, 1H), 6.12 (dd, *J* = 15.9, 8.2 Hz, 1H), 4.14 – 4.07 (m, 1H), 3.03 (t, *J* = 9.6 Hz, 1H), 2.62 – 2.55 (m, 1H), 2.25 (s, 3H), 2.22 (s, 3H), 2.20 – 2.19 (m, 1H), 1.85 – 1.81 (m, 1H), 1.78 – 1.74 (m, 1H), 1.60 – 1.51 (m, 1H), 1.41 – 1.28 (m, 2H); **^{13}C NMR (126 MHz, CDCl$_3$)** δ 164.0, 150.9, 147.8, 142.4, 142.3, 139.2, 139.0, 138.1, 137.0, 135.2, 129.7, 129.3, 129.2, 128.7, 128.5, 128.4, 126.8, 126.2, 125.7, 122.2, 62.1, 55.4, 49.5, 32.4, 31.9, 24.0, 21.43, 21.40; **HRMS** (ESI/TOF-Q) m/z: $[M+H]^+$ Calculated for $C_{34}H_{35}N_3O_2SH^+$ 550.2523; Found 550.2511.

15. NMR spectra of synthesized compounds

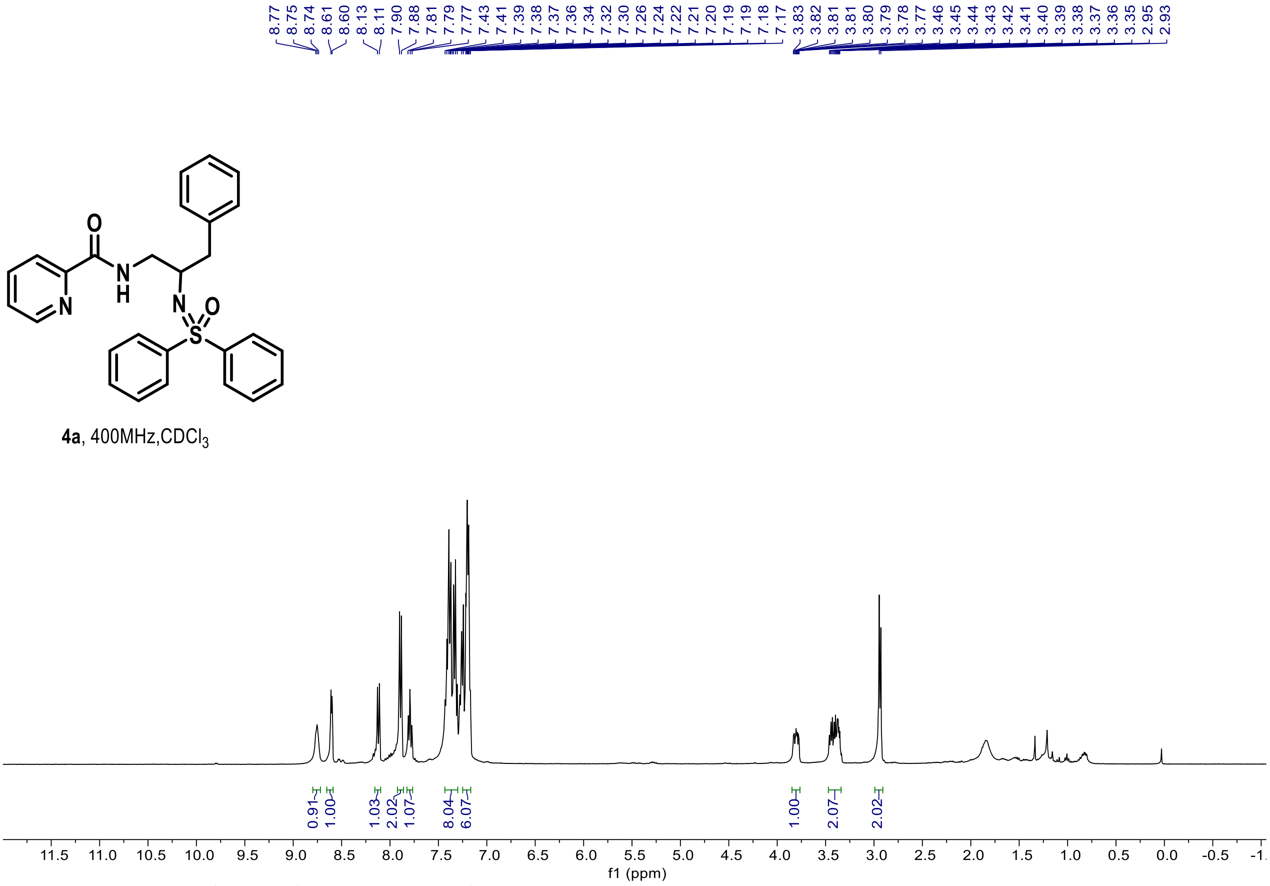

4a, 400MHz,CDCl$_3$

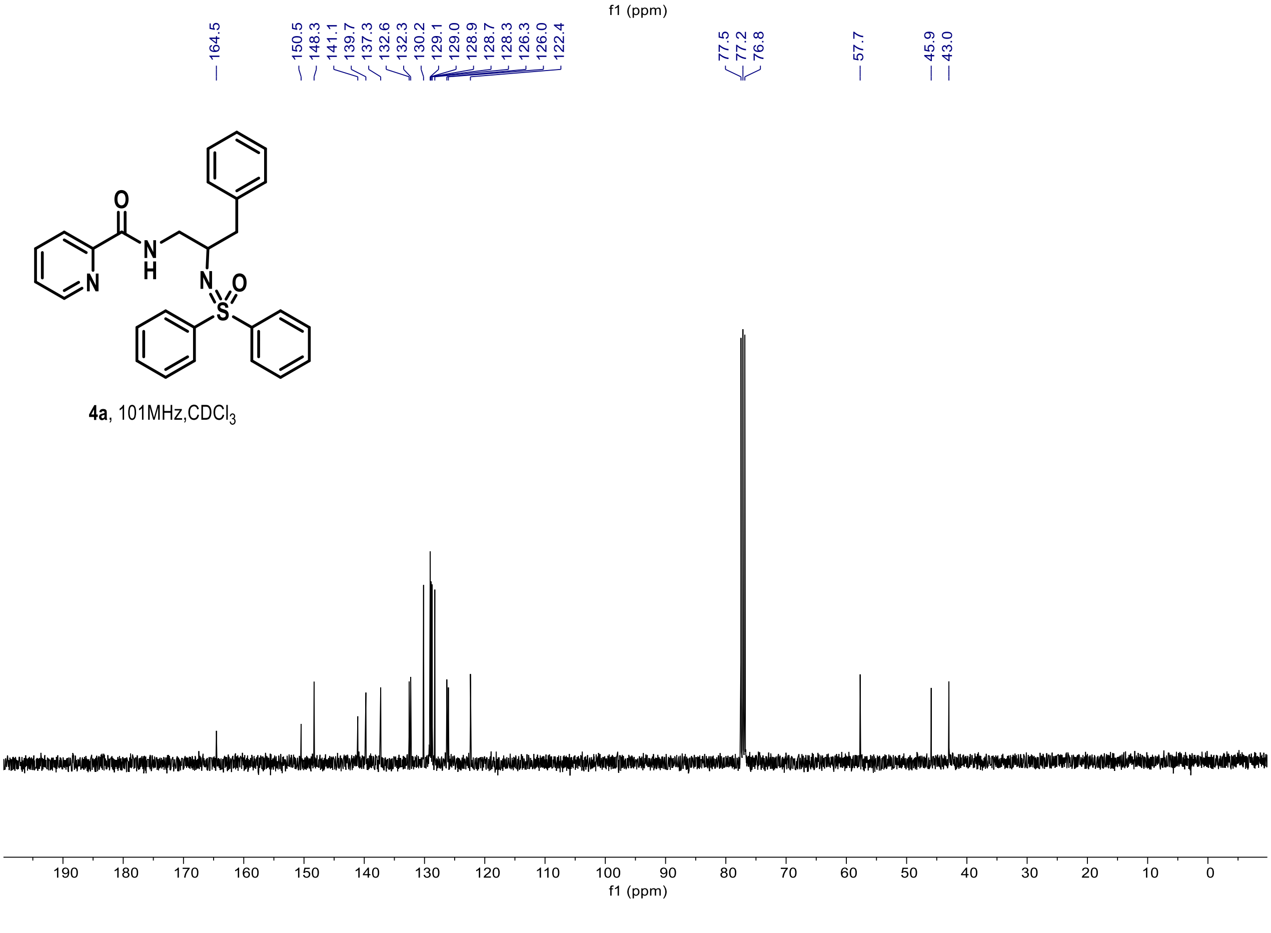

4a, 101MHz,CDCl$_3$

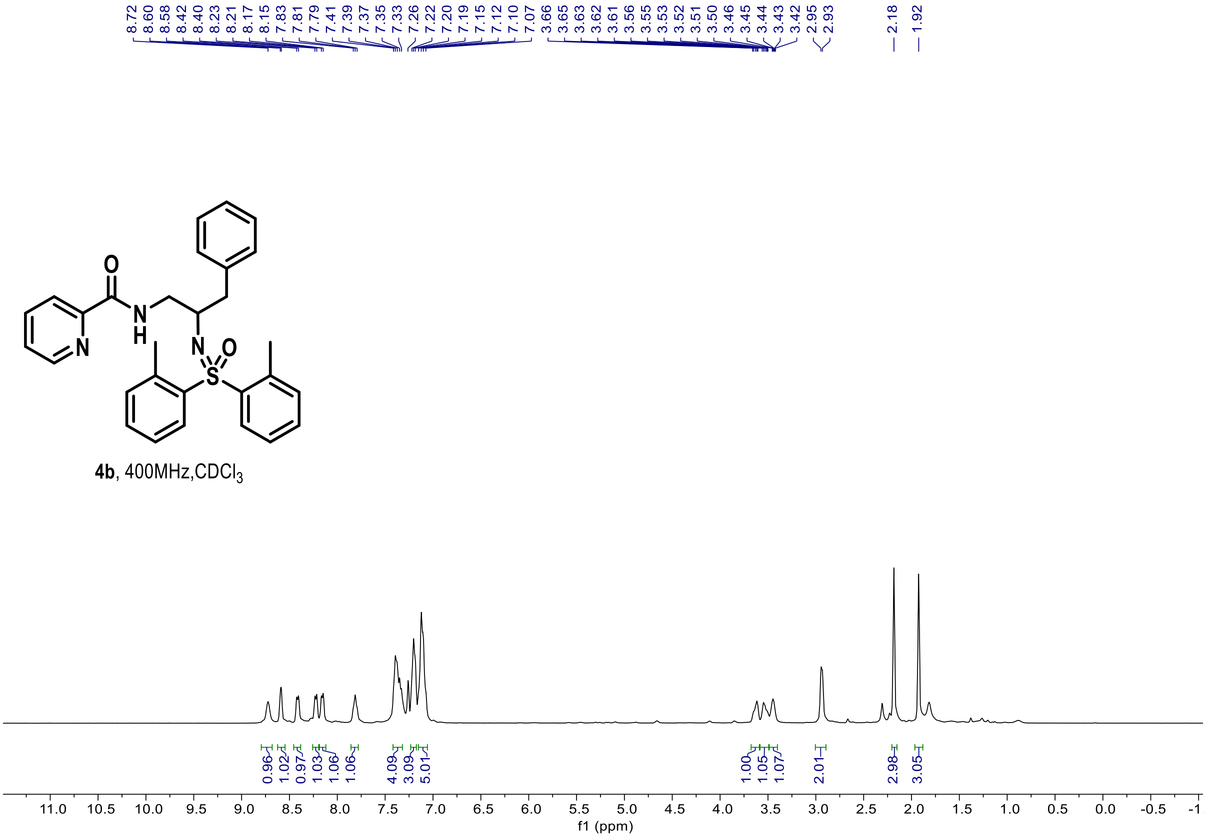
8.72 8.60 8.58 8.42 8.40 8.23 8.21 8.17 8.15 7.83 7.81 7.79 7.41 7.39 7.37 7.35 7.33 7.26 7.22 7.20 7.19 7.15 7.12 7.10 7.07 3.66 3.65 3.63 3.62 3.61 3.56 3.55 3.53 3.52 3.51 3.50 3.46 3.45 3.44 3.43 3.42 2.95 2.93
2.18
1.92
4b, 400MHz,CDCl3
0.96 1.02 0.97 1.03 1.06 1.06 4.09 3.09 5.01 1.00 1.05 1.07 2.01 2.98 3.05
f1 (ppm)

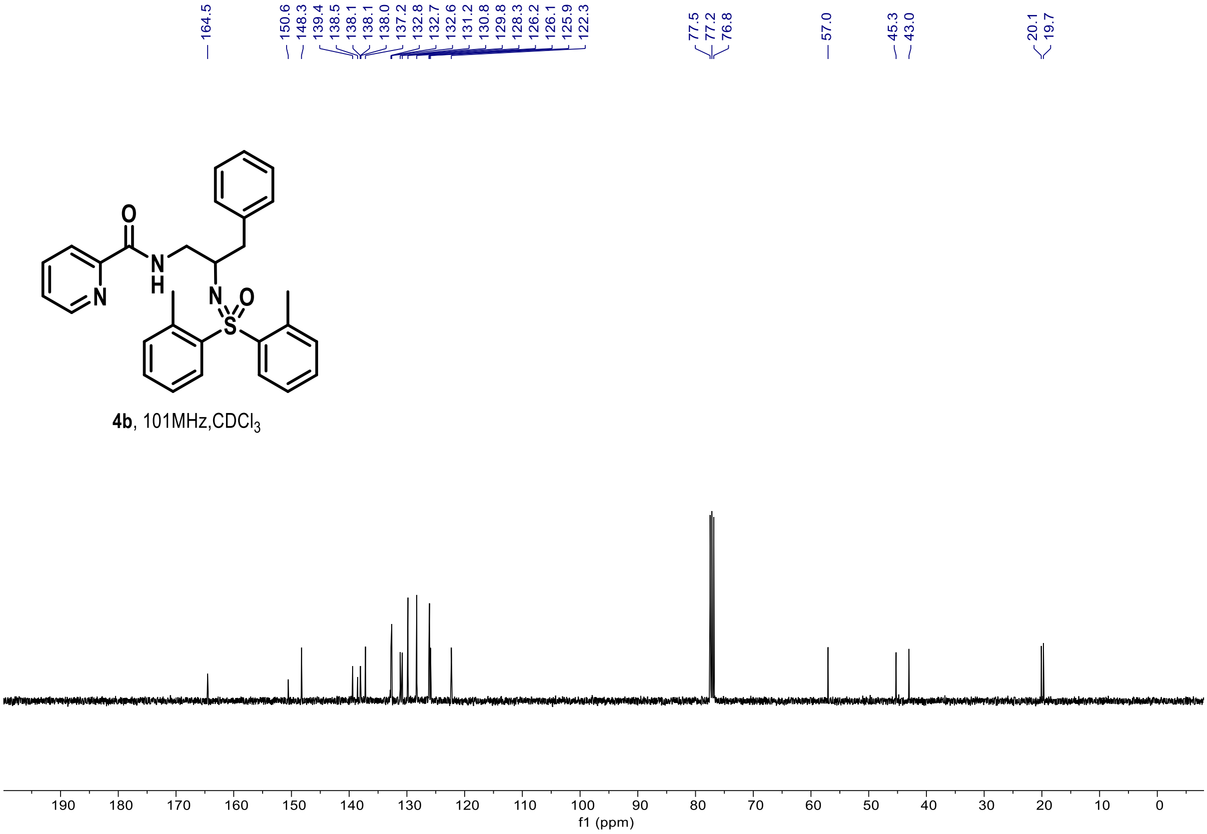
164.5
150.6 148.3 139.4 138.5 138.1 138.1 138.0 137.2 132.8 132.7 132.6 131.2 130.8 129.8 128.3 126.2 126.1 125.9 122.3
77.5 77.2 76.8
57.0
45.3 43.0
20.1 19.7
4b, 101MHz,CDCl3
f1 (ppm)

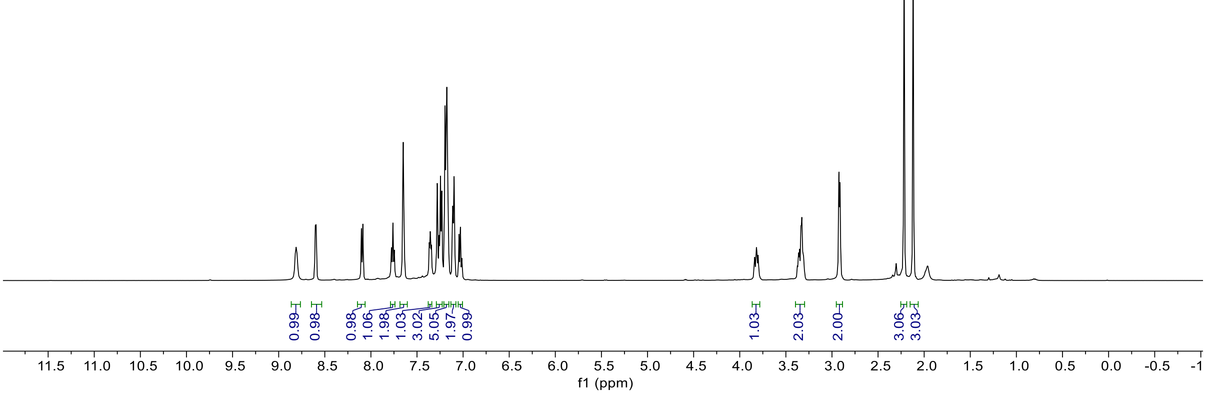

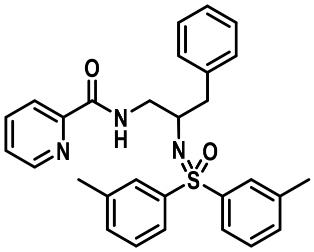

4c, 500MHz,CDCl$_3$

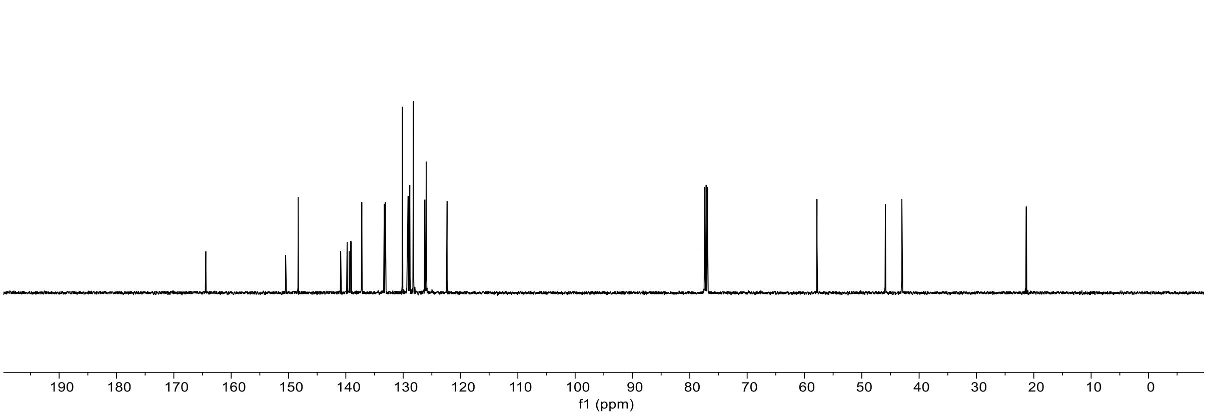

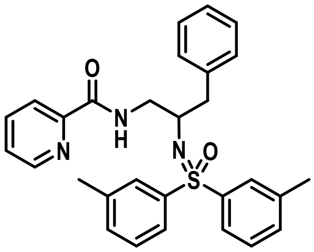

4c, 126MHz,CDCl$_3$

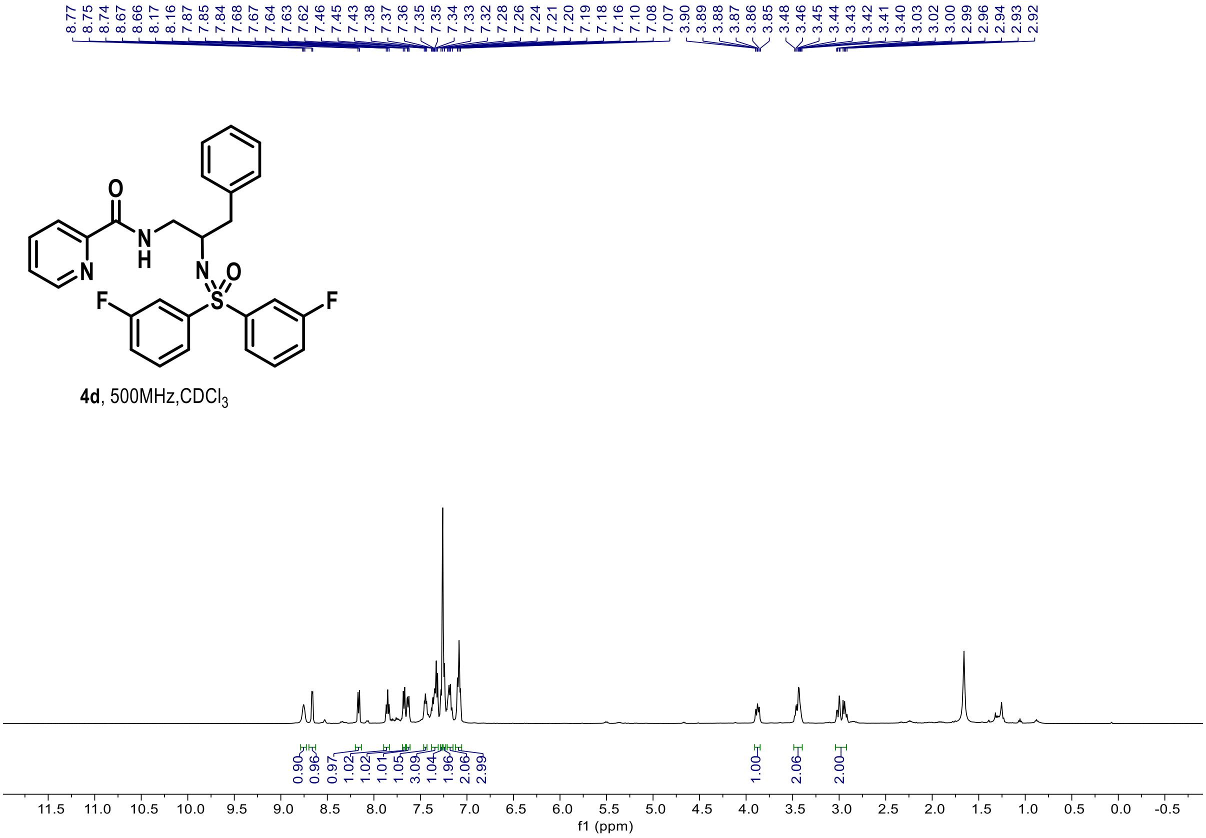

4d, 500MHz,$CDCl_3$

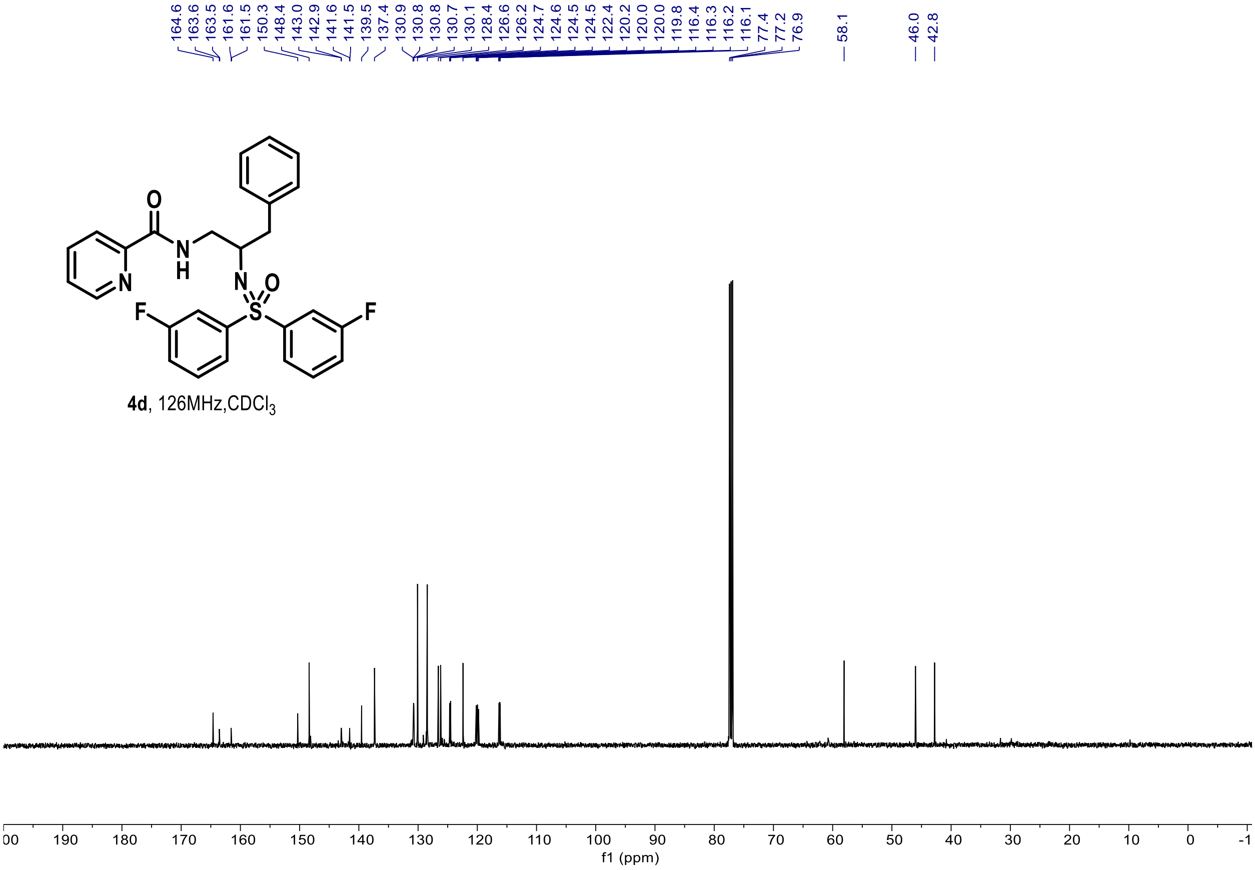

4d, 126MHz,$CDCl_3$

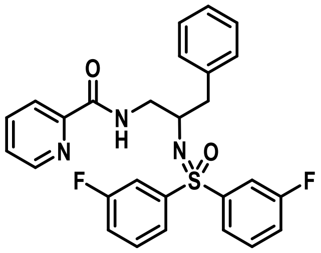

4d, 471MHz,CDCl$_3$

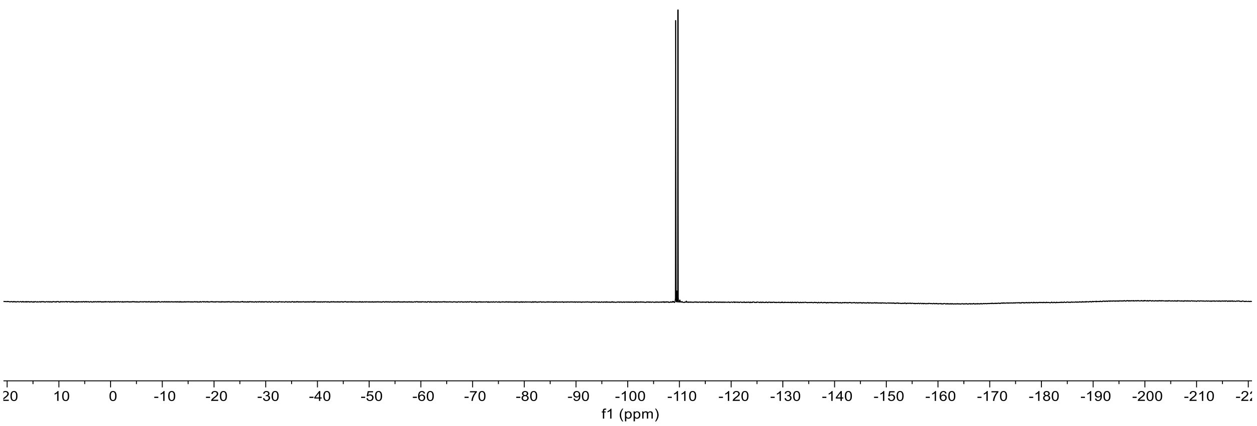

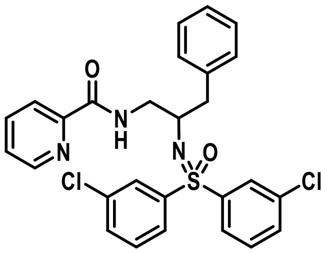

4e, 500MHz,CDCl$_3$

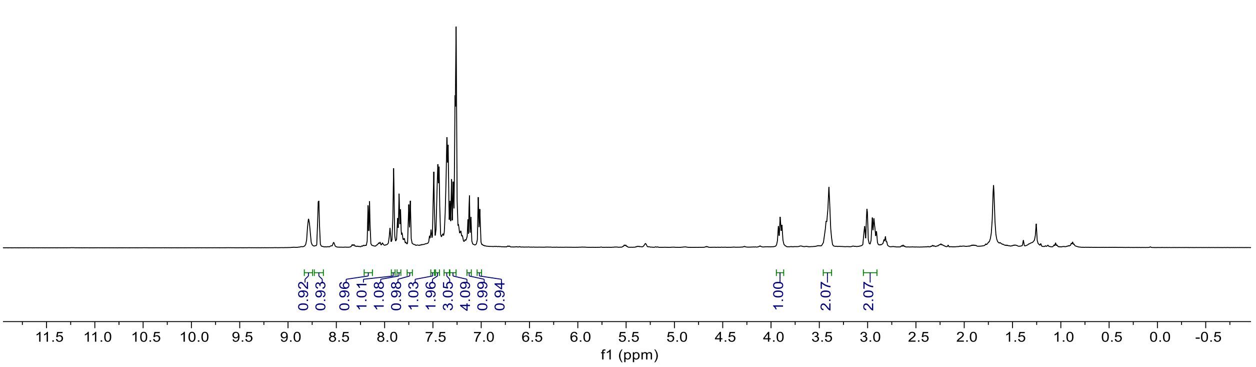

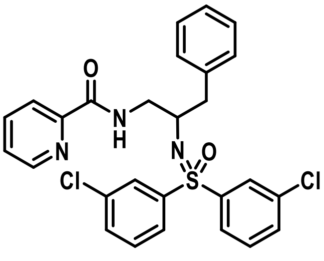

4e, 126MHz,$CDCl_3$

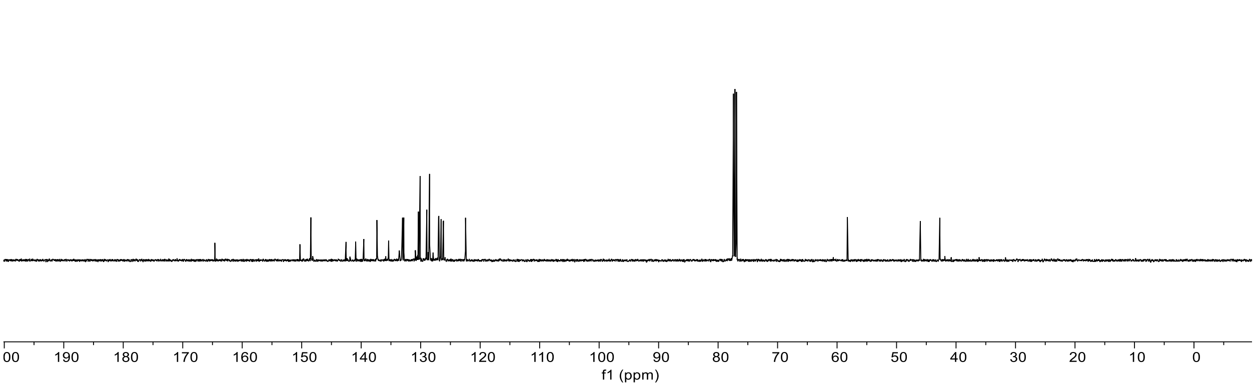

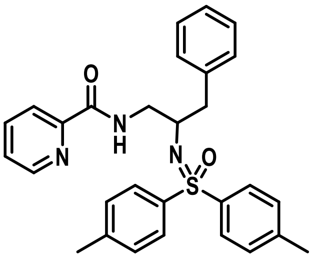

4f, 500MHz,$CDCl_3$

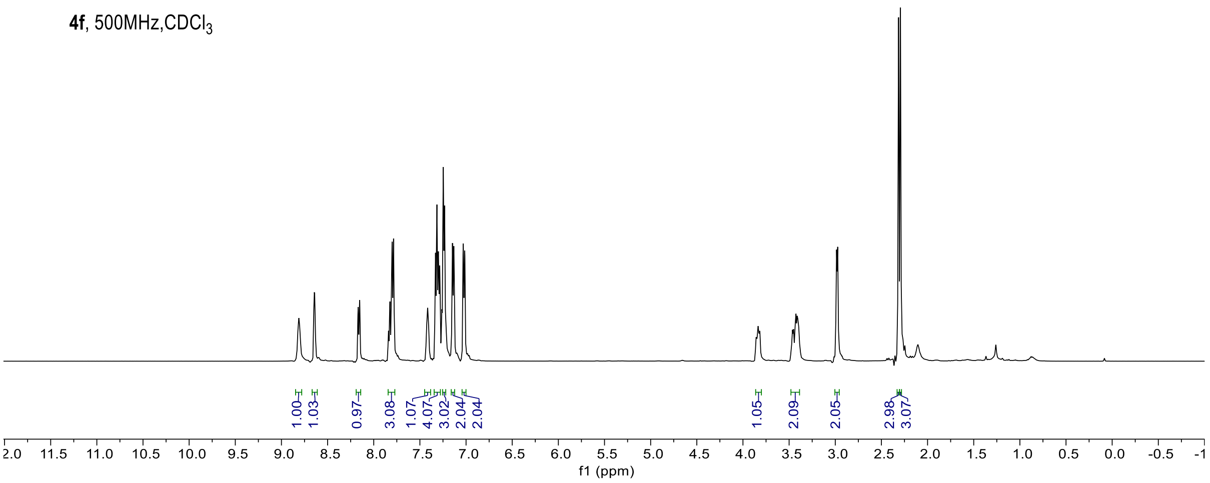

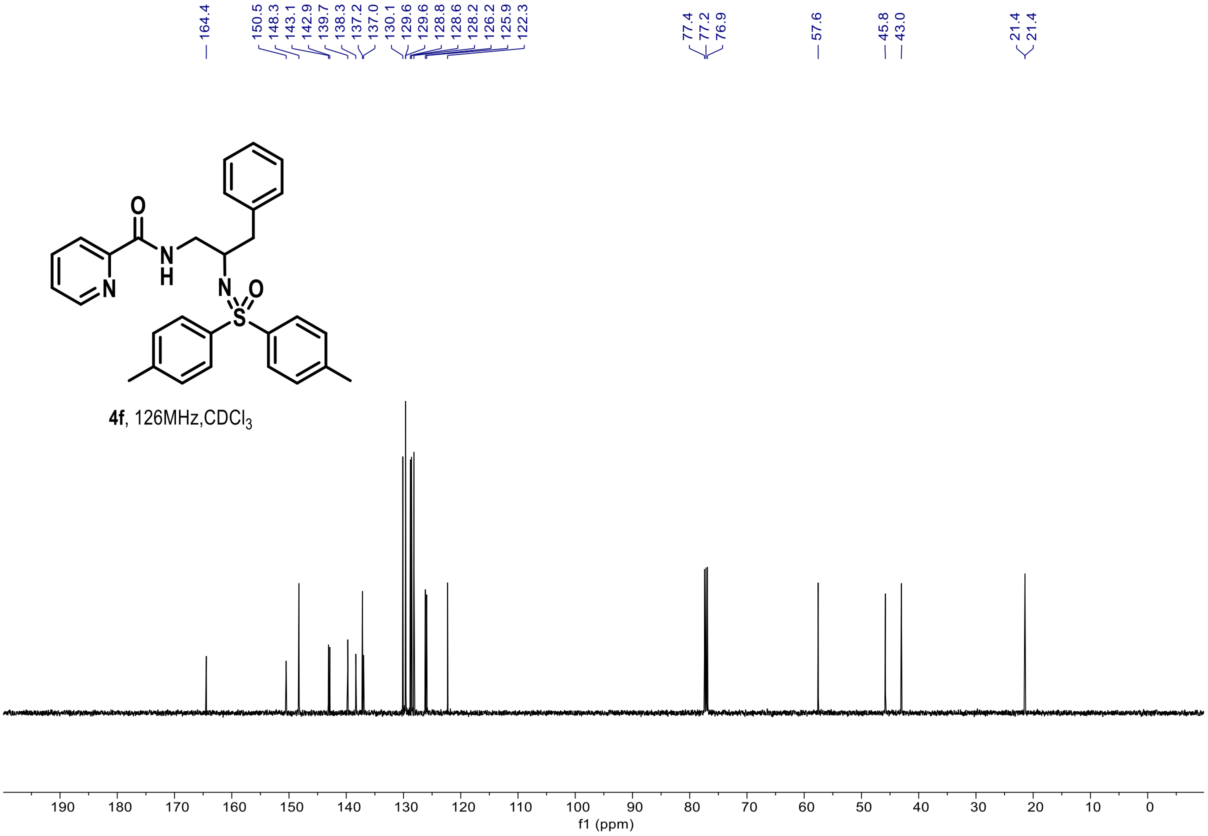

4f, 126MHz,$CDCl_3$

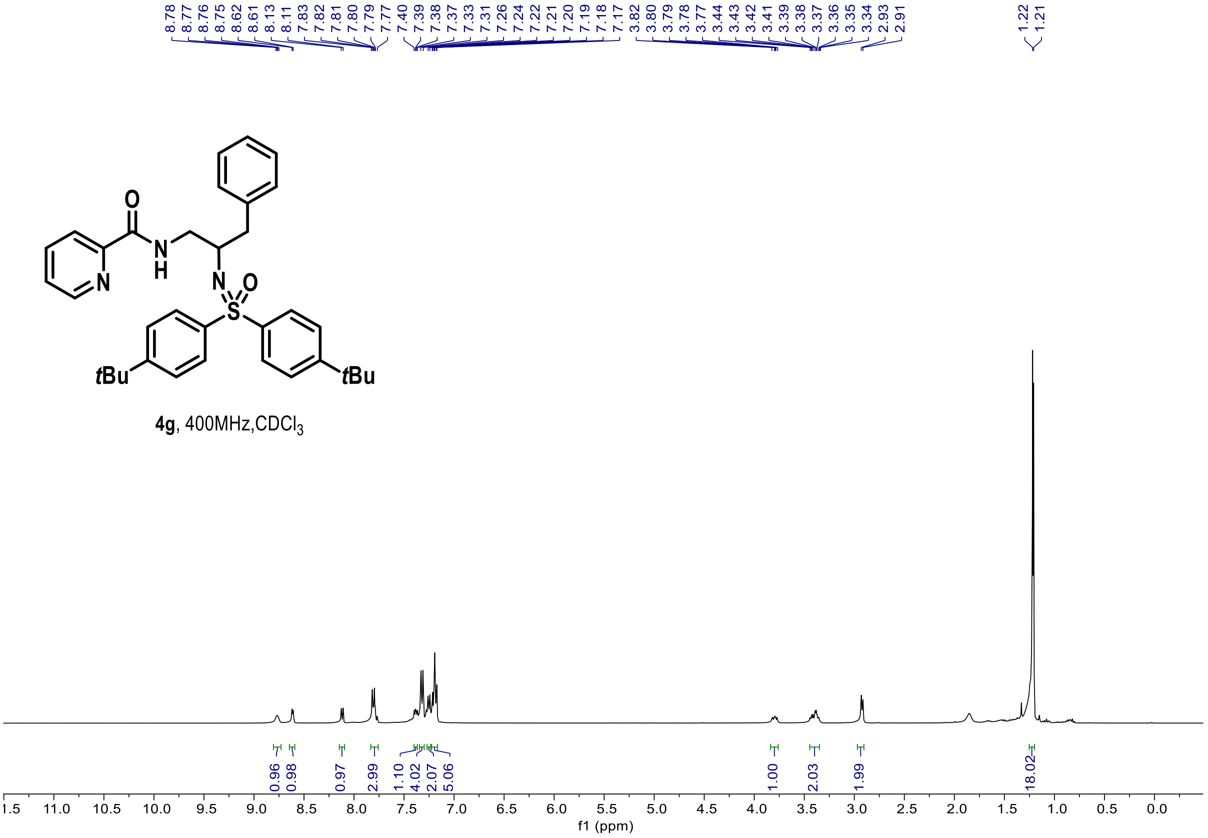

4g, 400MHz,$CDCl_3$

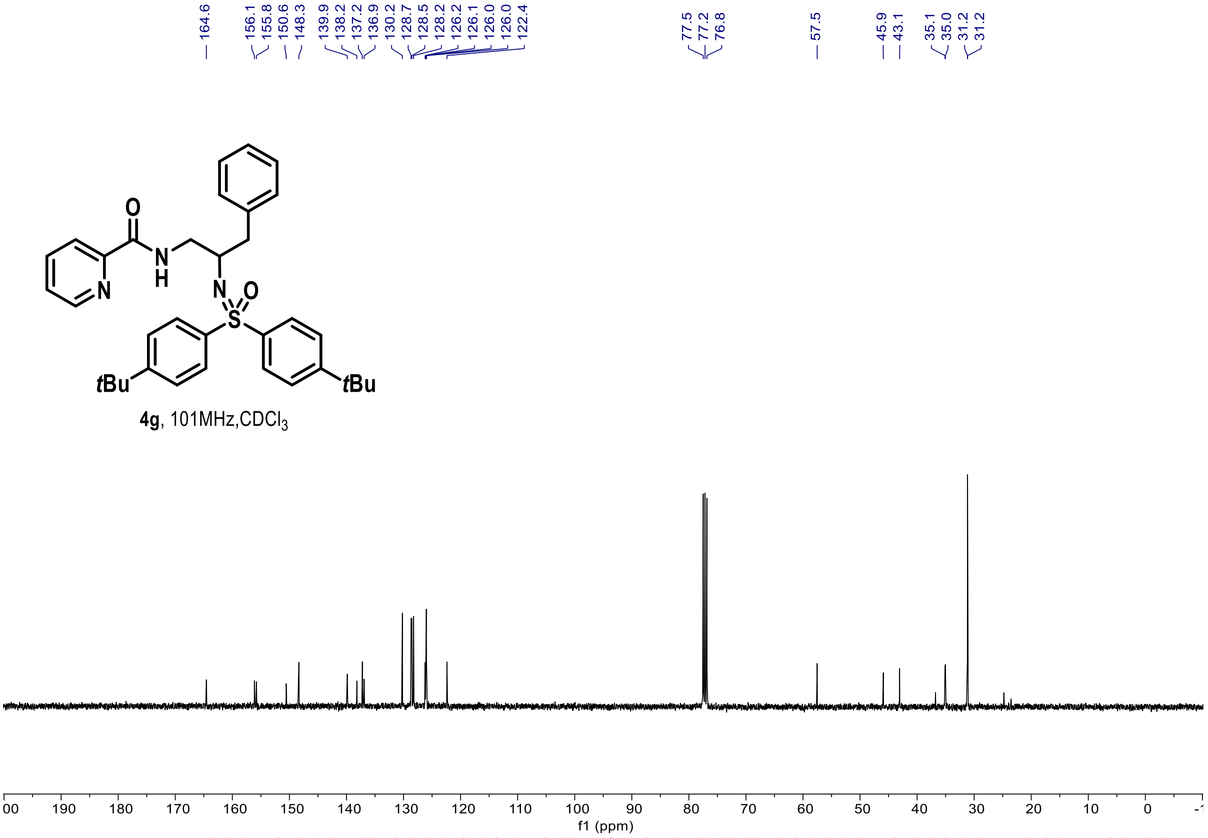

4g, 101MHz,$CDCl_3$

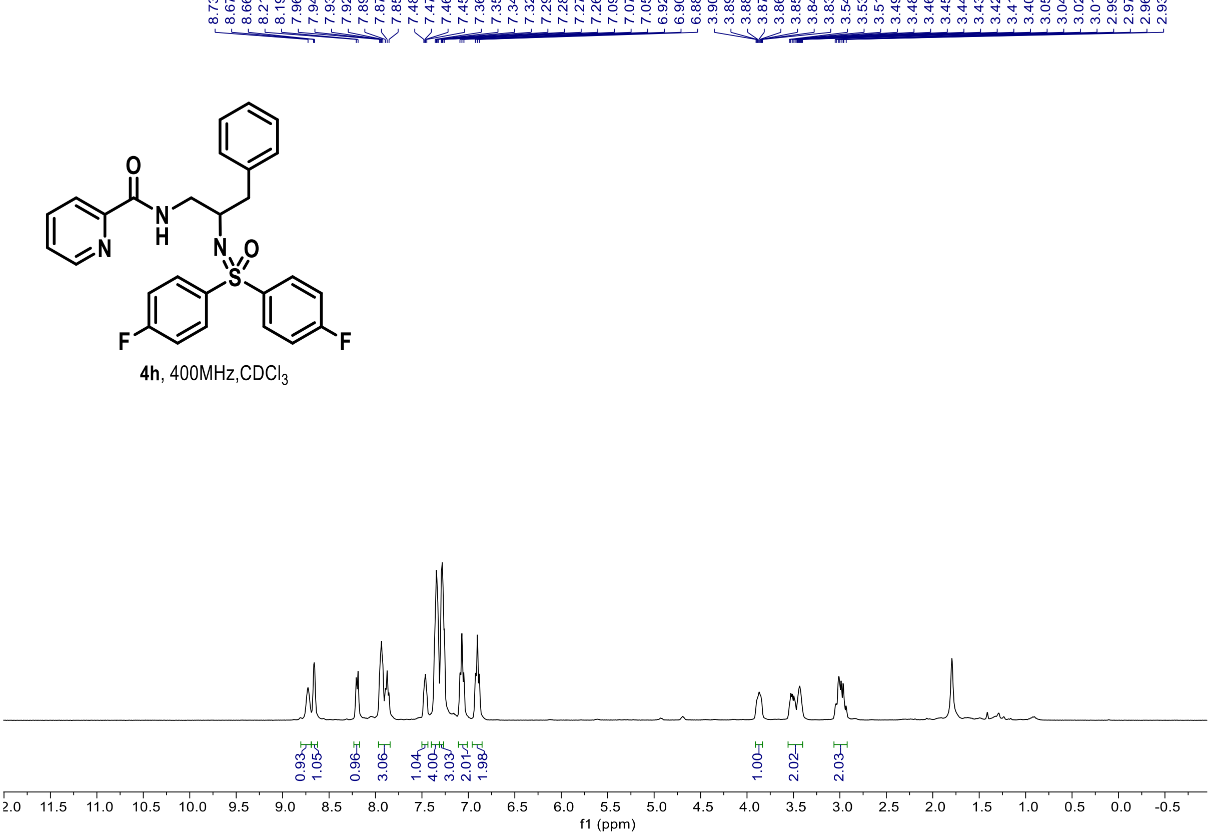

4h, 400MHz,$CDCl_3$

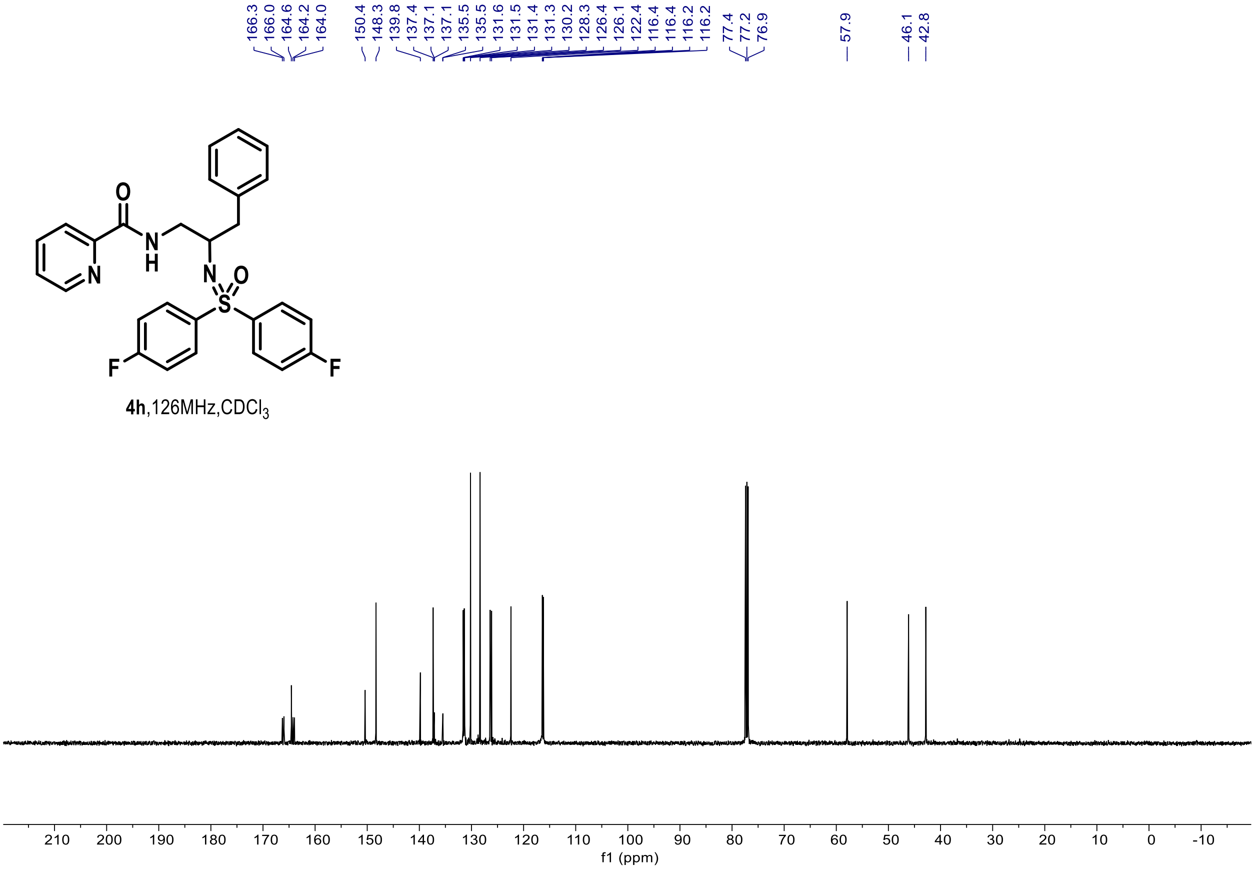

4h,126MHz,$CDCl_3$

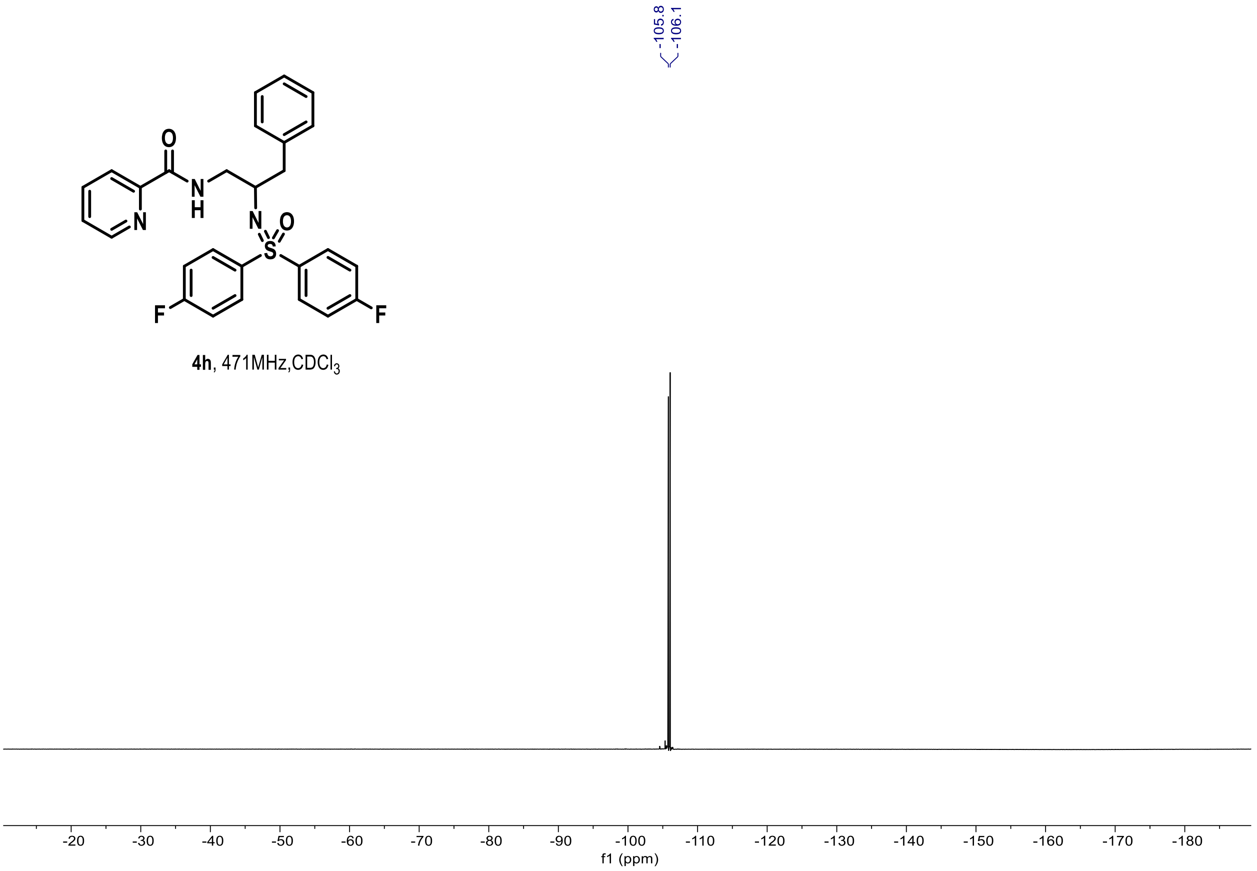

4h, 471MHz,$CDCl_3$

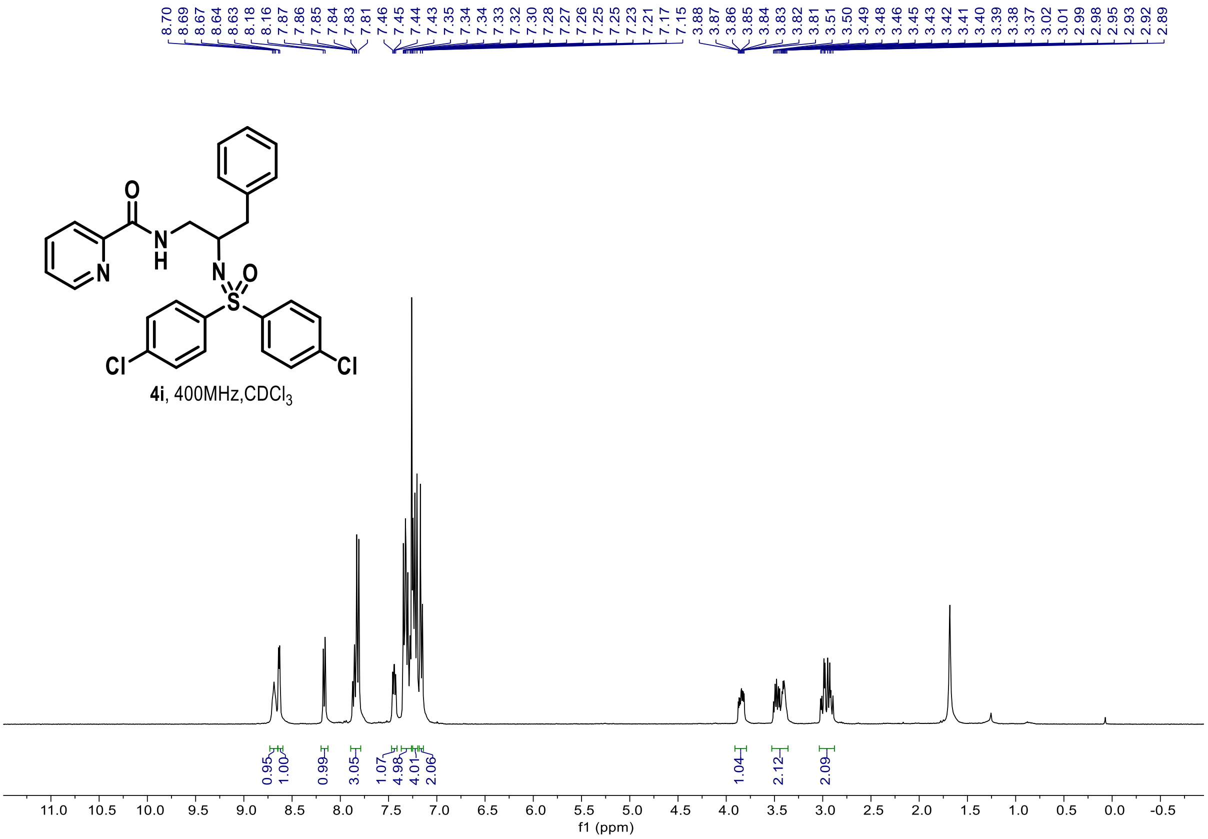
8.70 8.69 8.67 8.64 8.63 8.18 8.16 7.87 7.86 7.85 7.84 7.83 7.81 7.46 7.45 7.44 7.43 7.35 7.34 7.34 7.33 7.32 7.30 7.28 7.27 7.26 7.25 7.25 7.23 7.21 7.17 7.15 3.88 3.87 3.86 3.85 3.84 3.83 3.82 3.81 3.51 3.50 3.49 3.48 3.46 3.45 3.43 3.42 3.41 3.40 3.39 3.38 3.37 3.02 3.01 2.99 2.98 2.95 2.93 2.92 2.89
4i, 400MHz,CDCl3
0.95 1.00 0.99 3.05 1.07 4.98 4.01 2.06 1.04 2.12 2.09
f1 (ppm)

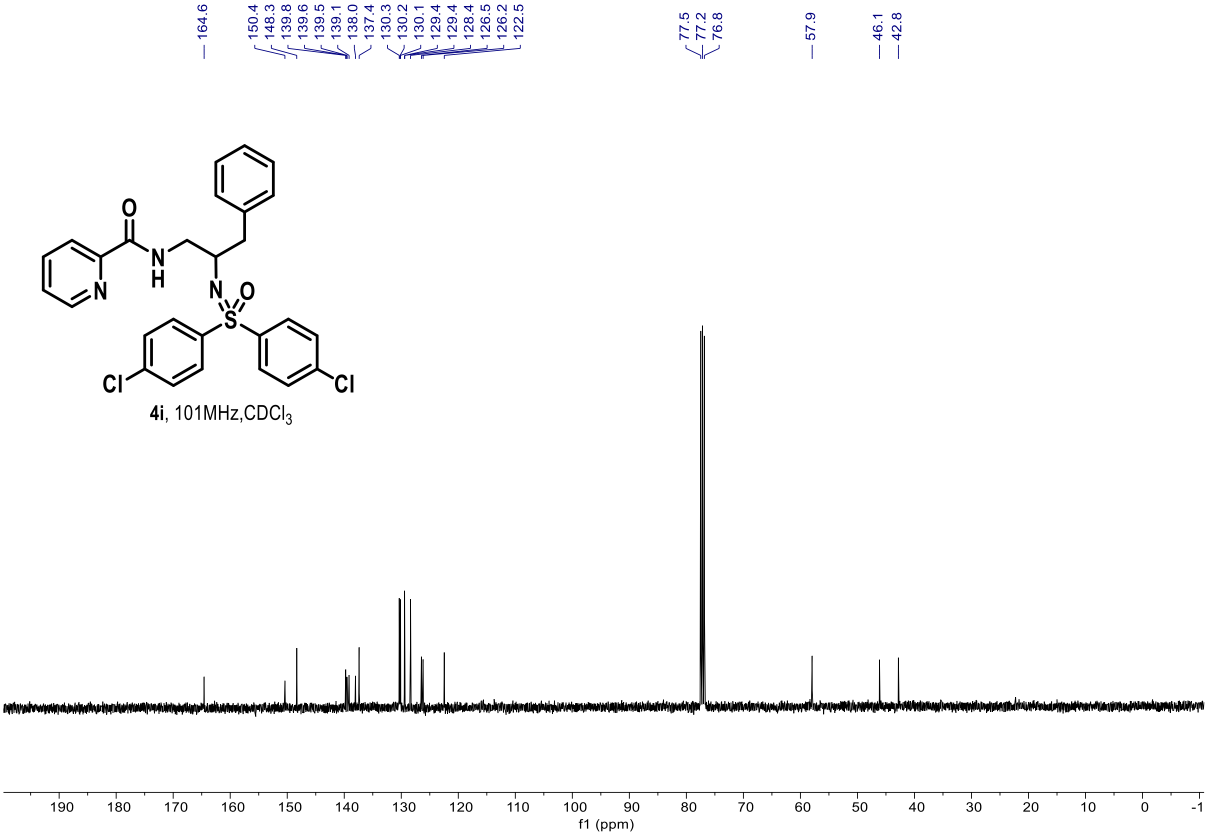
164.6 150.4 148.3 139.8 139.6 139.5 139.1 138.0 137.4 130.3 130.2 130.1 129.4 129.4 128.4 126.5 126.2 122.5 77.5 77.2 76.8 57.9 46.1 42.8
4i, 101MHz,CDCl3
f1 (ppm)

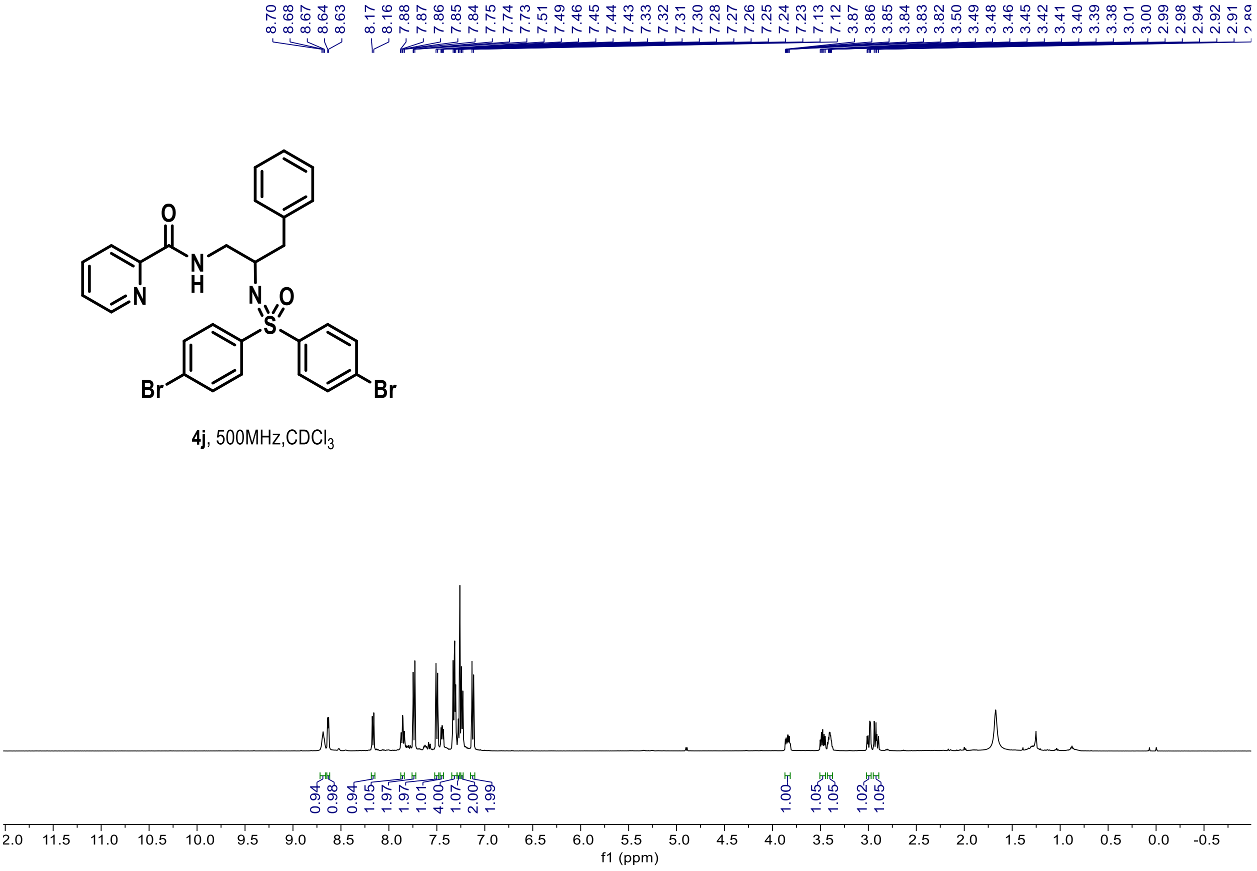

4j, 500MHz,$CDCl_3$

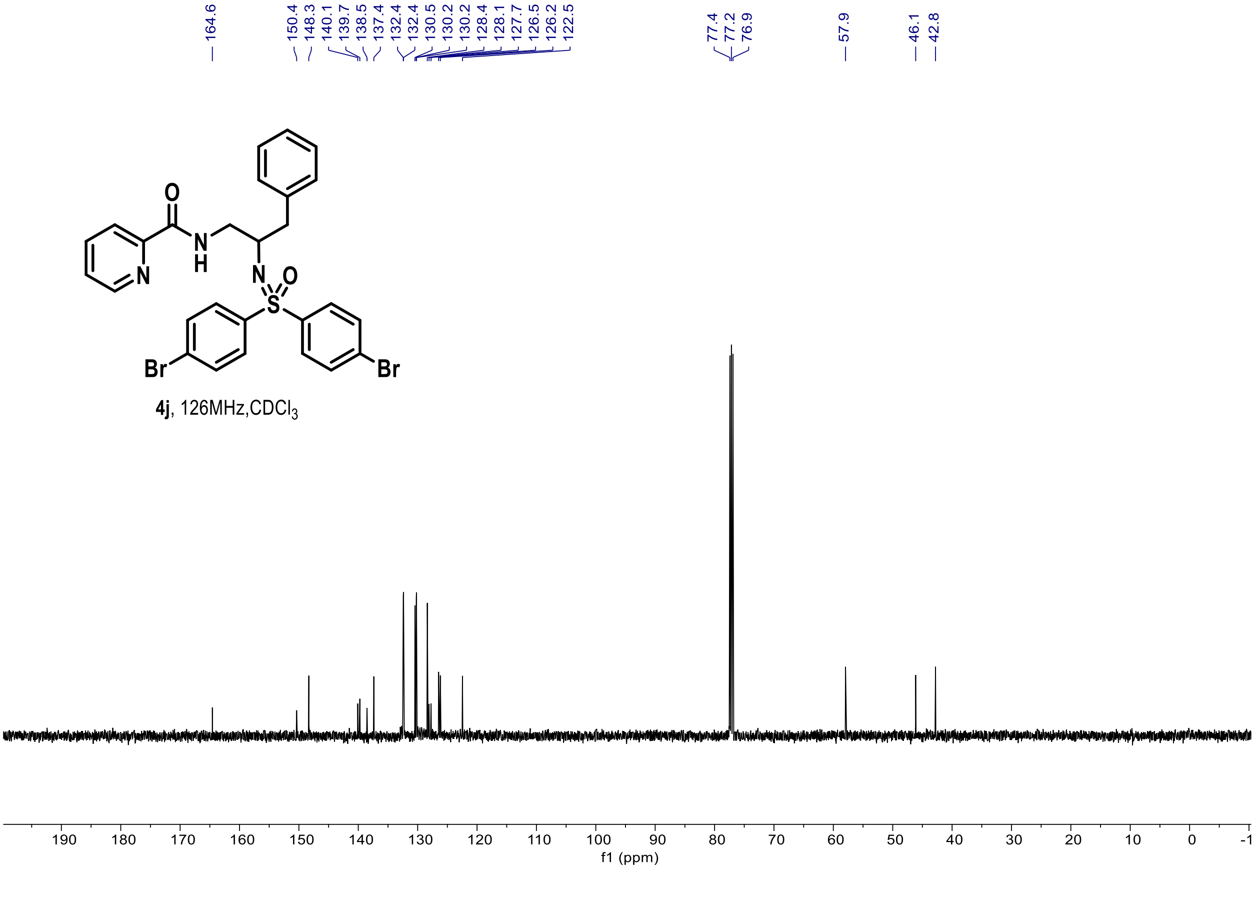

4j, 126MHz,$CDCl_3$

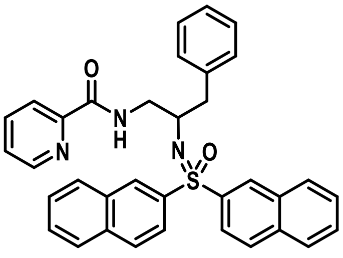

4k, 400MHz,$CDCl_3$

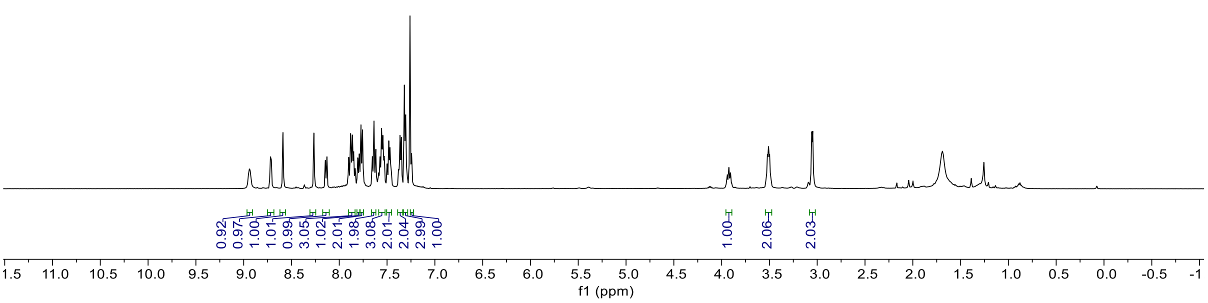

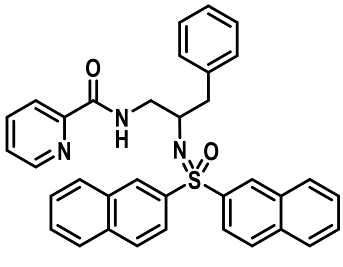

4k, 126MHz,$CDCl_3$

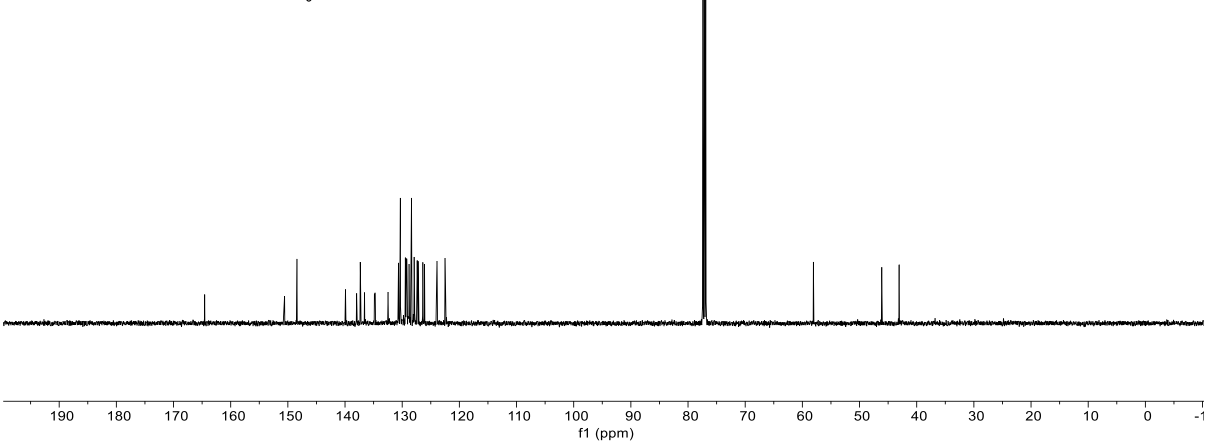

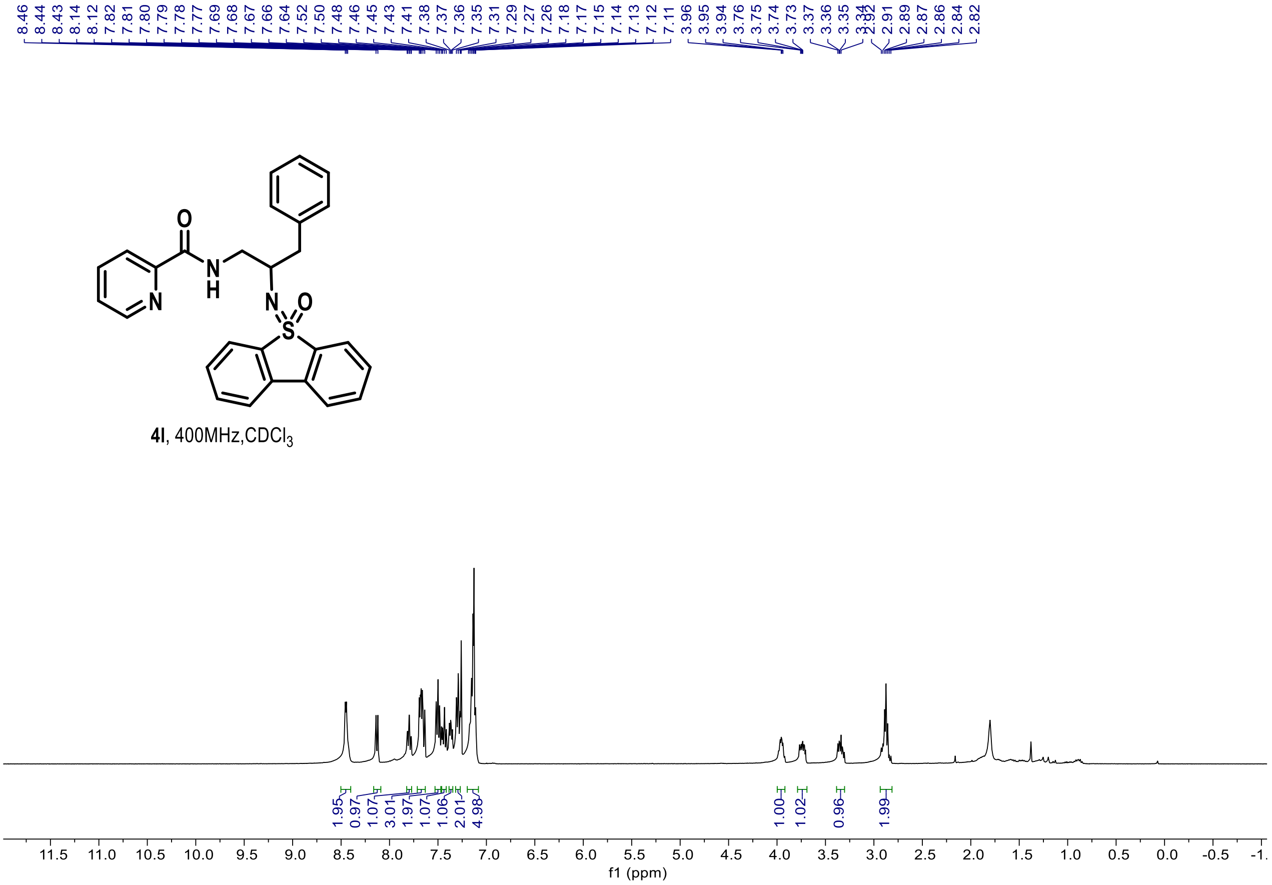

4l, 400MHz,$CDCl_3$

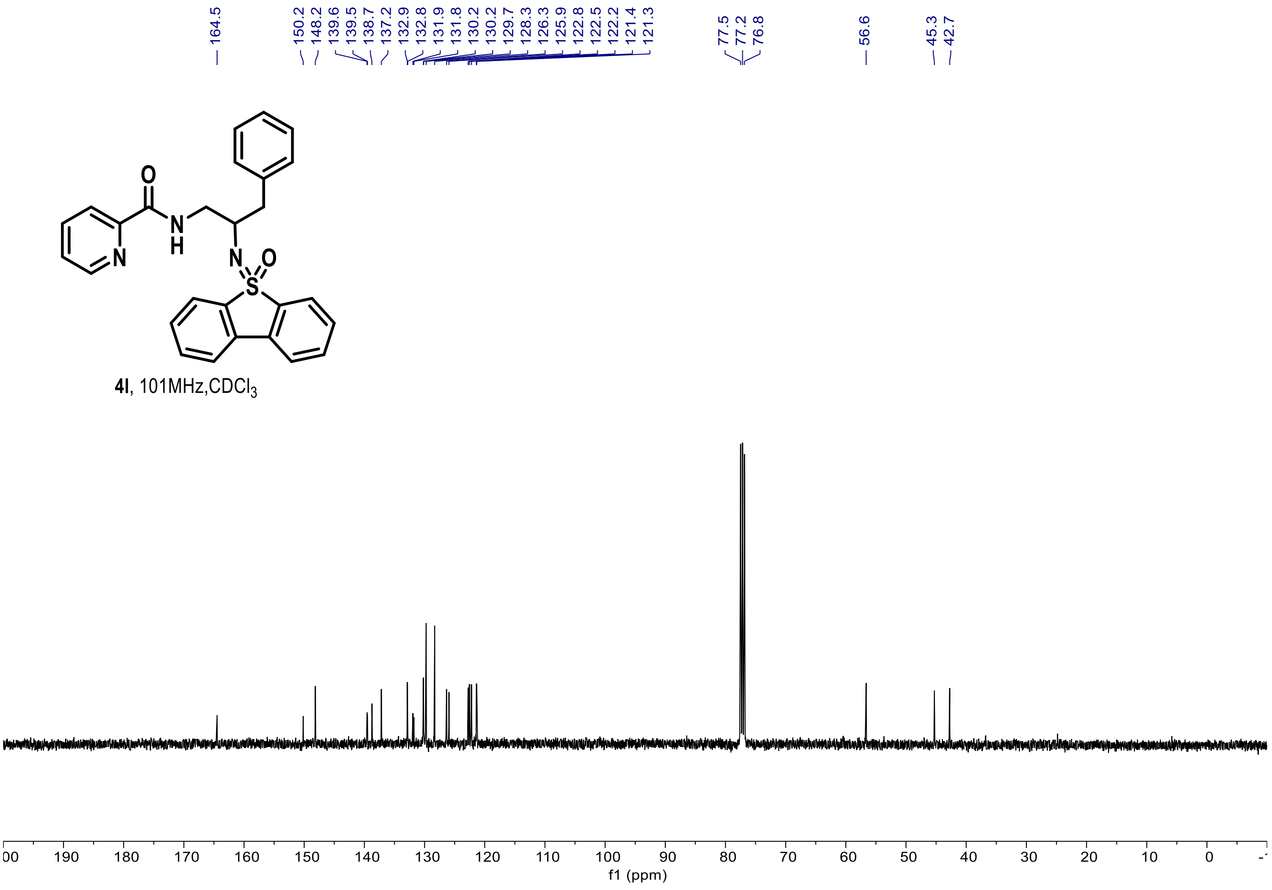

4l, 101MHz,$CDCl_3$

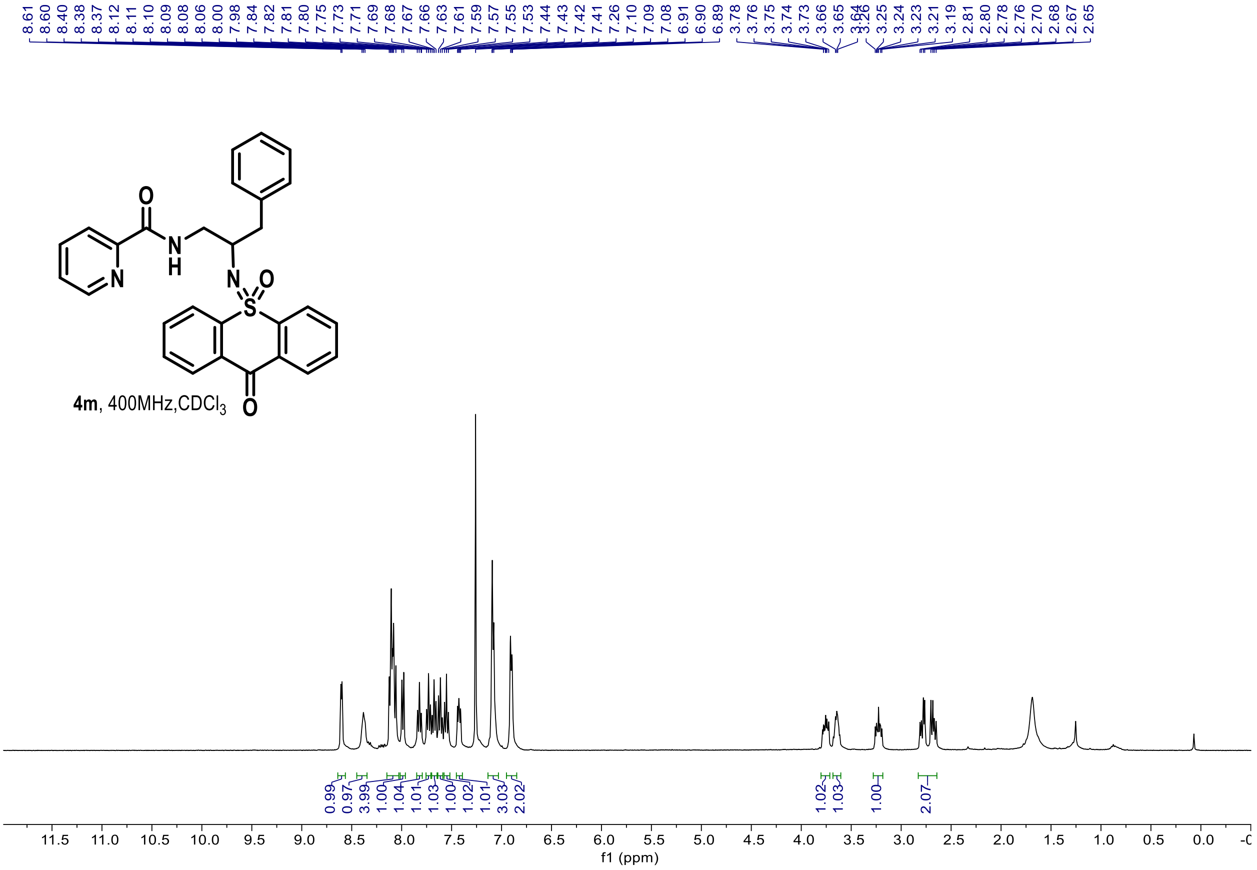

4m, 400MHz,CDCl$_3$

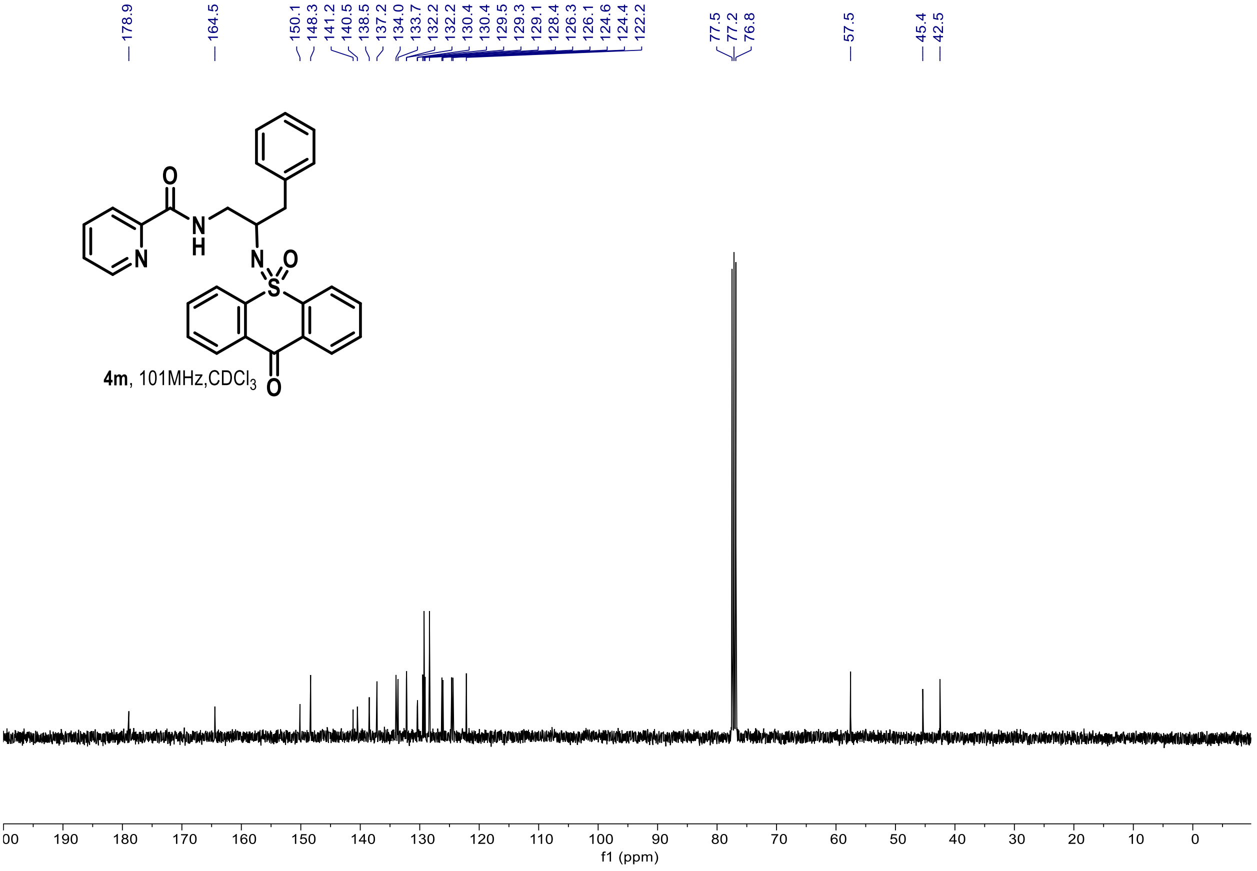

4m, 101MHz,CDCl$_3$

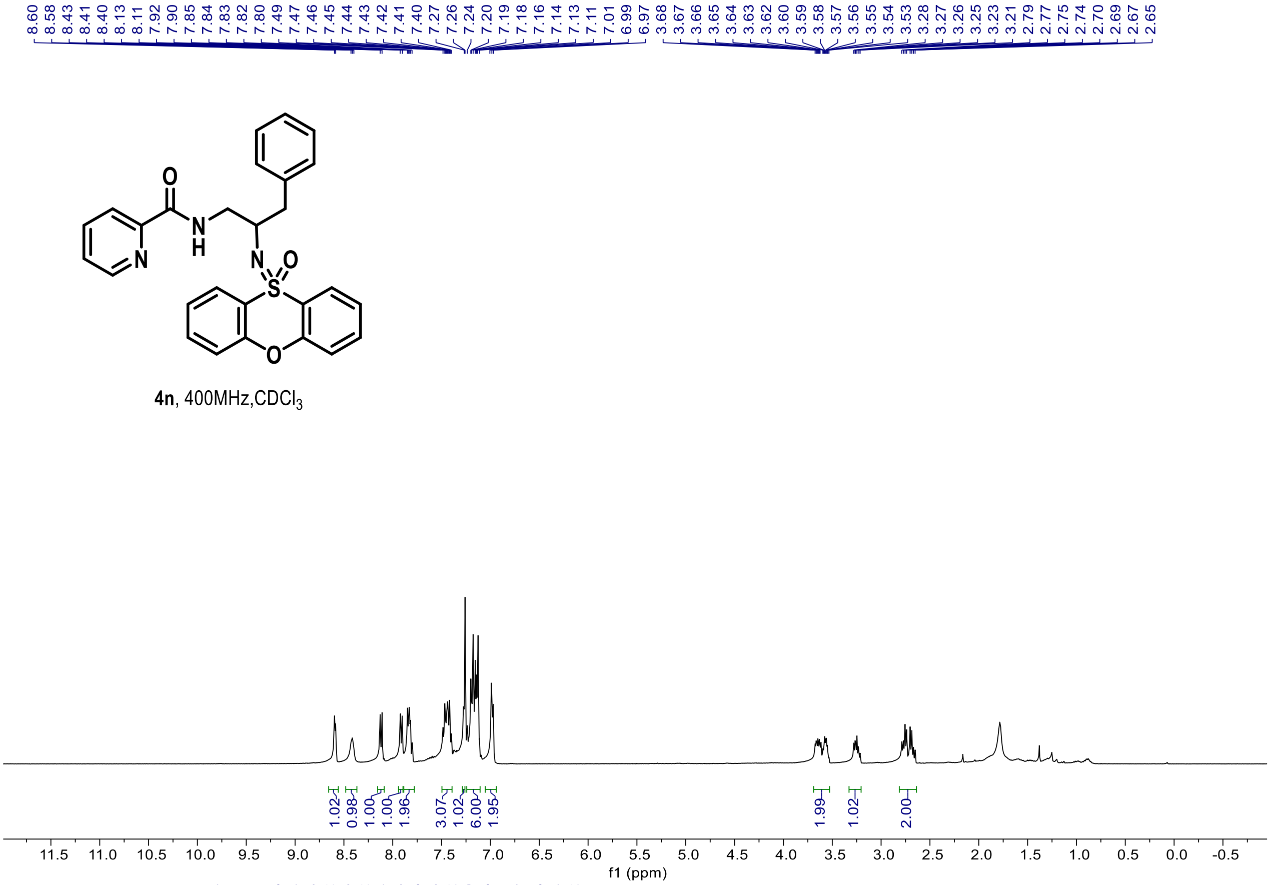

4n, 400MHz,$CDCl_3$

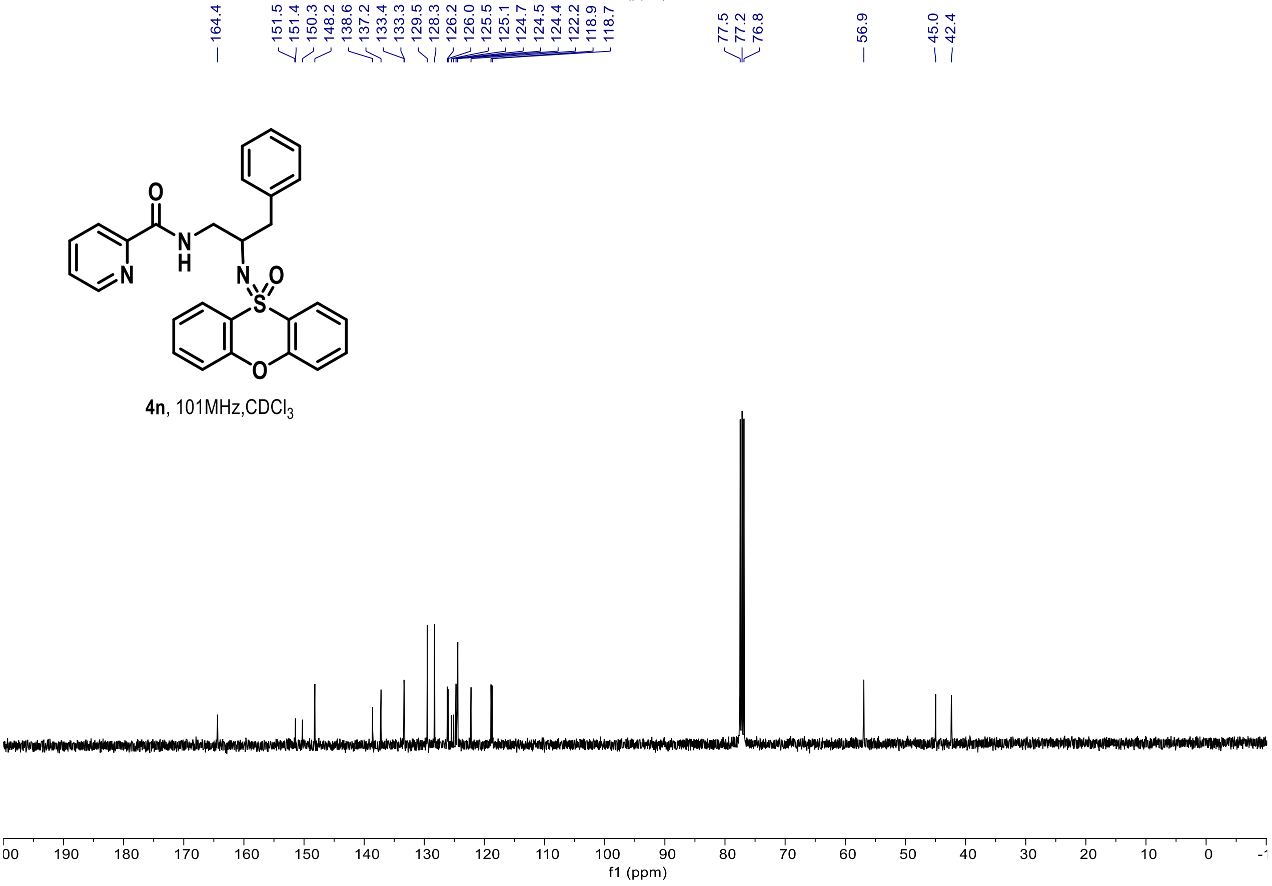

4n, 101MHz,$CDCl_3$

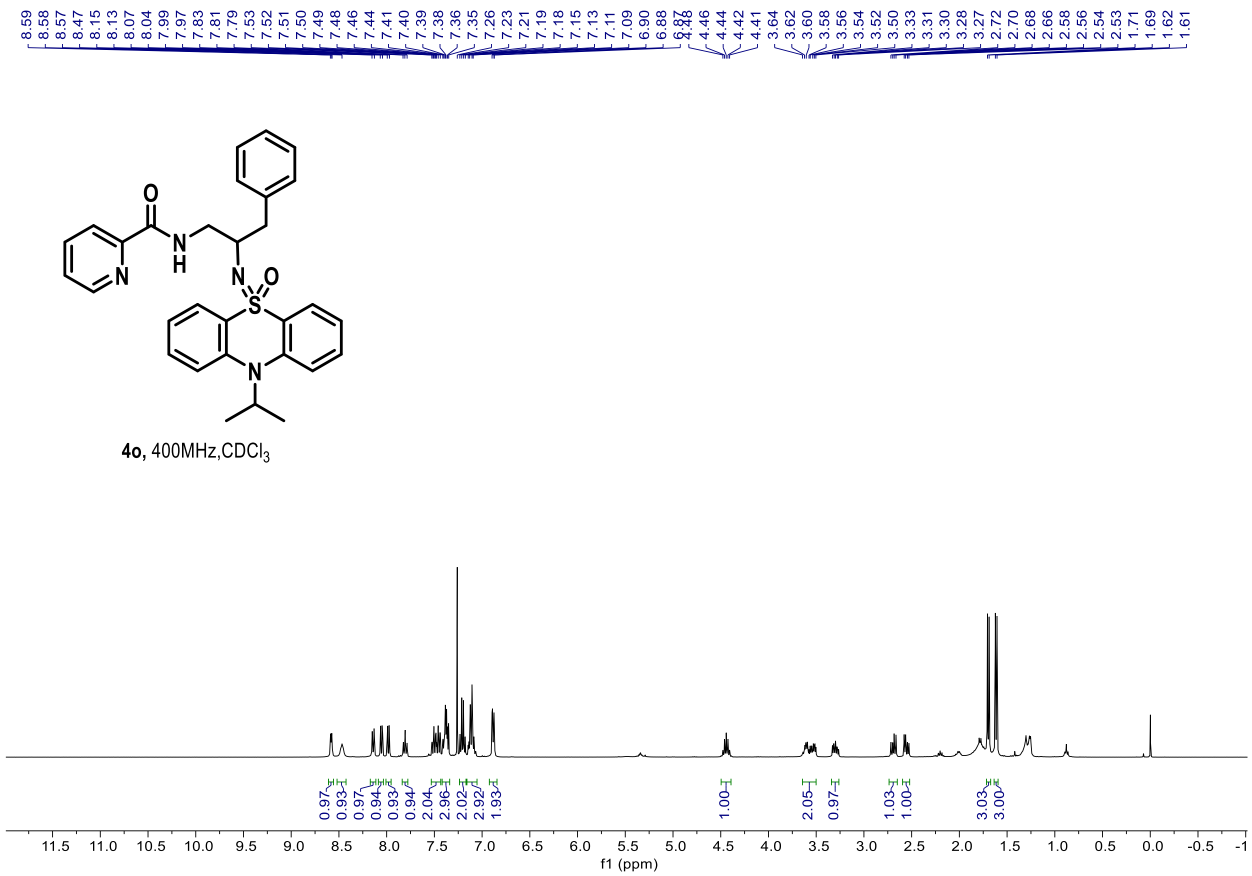

4o, 400MHz,$CDCl_3$

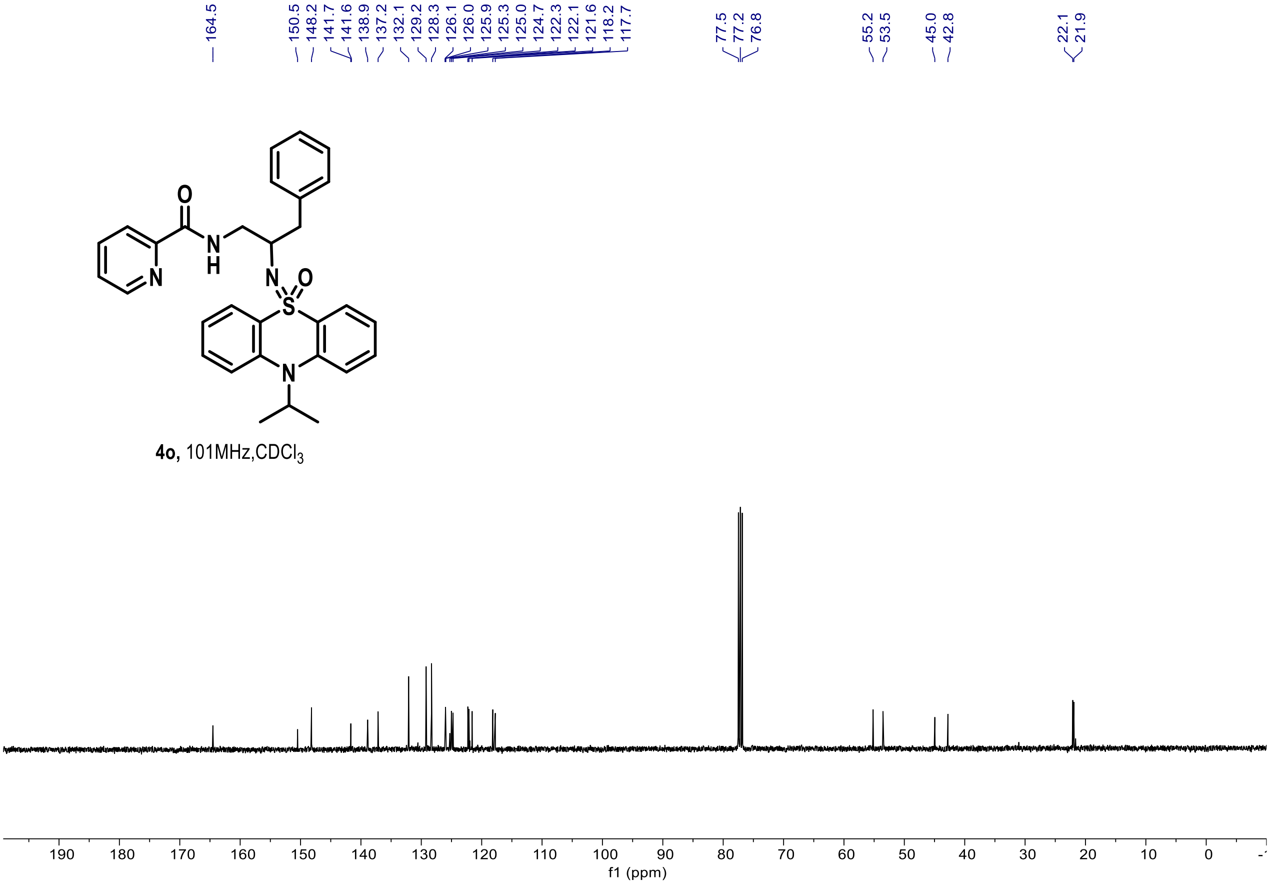

4o, 101MHz,$CDCl_3$

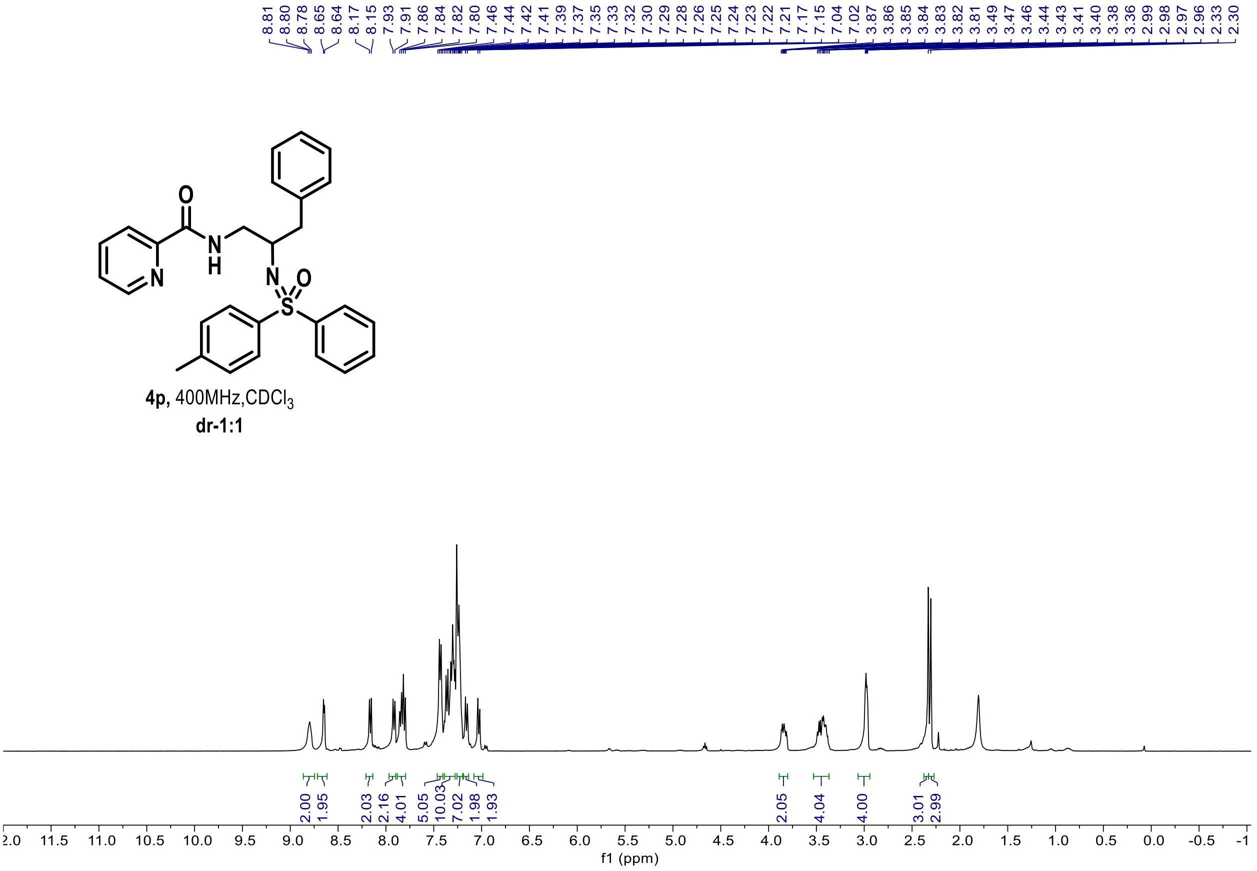
8.81 8.80 8.78 8.65 8.64 8.17 8.15 7.93 7.91 7.86 7.84 7.82 7.80 7.46 7.44 7.42 7.41 7.39 7.37 7.35 7.33 7.32 7.30 7.29 7.28 7.26 7.25 7.24 7.23 7.22 7.21 7.17 7.15 7.04 7.02 3.87 3.86 3.85 3.84 3.83 3.82 3.81 3.49 3.47 3.46 3.44 3.43 3.41 3.40 3.38 3.36 2.99 2.98 2.97 2.96 2.33 2.30
O
N
H
N
N
O
S
4p, 400MHz,CDCl$_3$
dr-1:1
2.00 1.95 2.03 2.16 4.01 5.05 10.03 7.02 1.98 1.93 2.05 4.04 4.00 3.01 2.99
f1 (ppm)

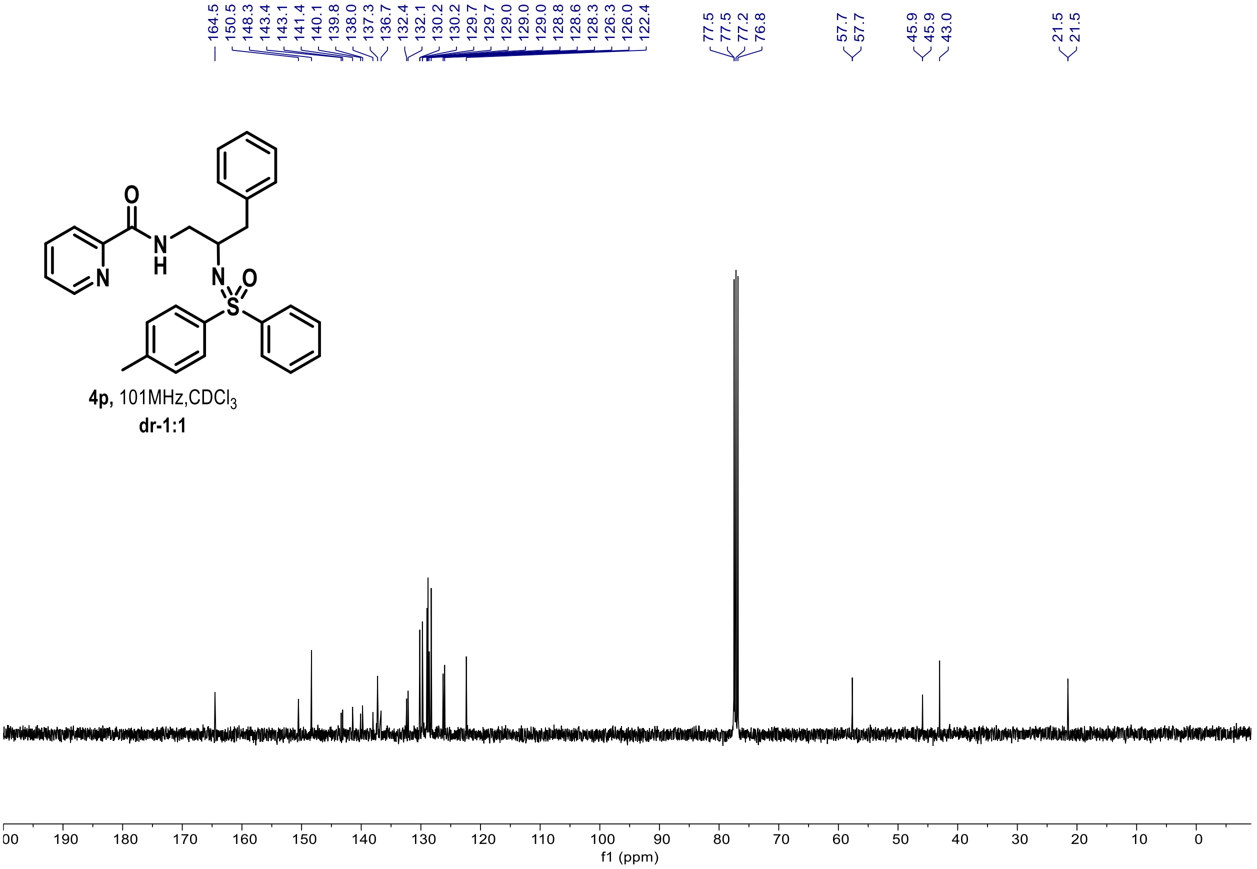
164.5 150.5 148.3 143.4 143.1 141.4 140.1 139.8 138.0 137.3 136.7 132.4 132.1 130.2 130.2 129.7 129.7 129.0 129.0 128.8 128.6 128.3 126.3 126.0 122.4 77.5 77.5 77.2 76.8 57.7 57.7 45.9 45.9 43.0 21.5 21.5
O
N
H
N
N
O
S
4p, 101MHz,CDCl$_3$
dr-1:1
f1 (ppm)

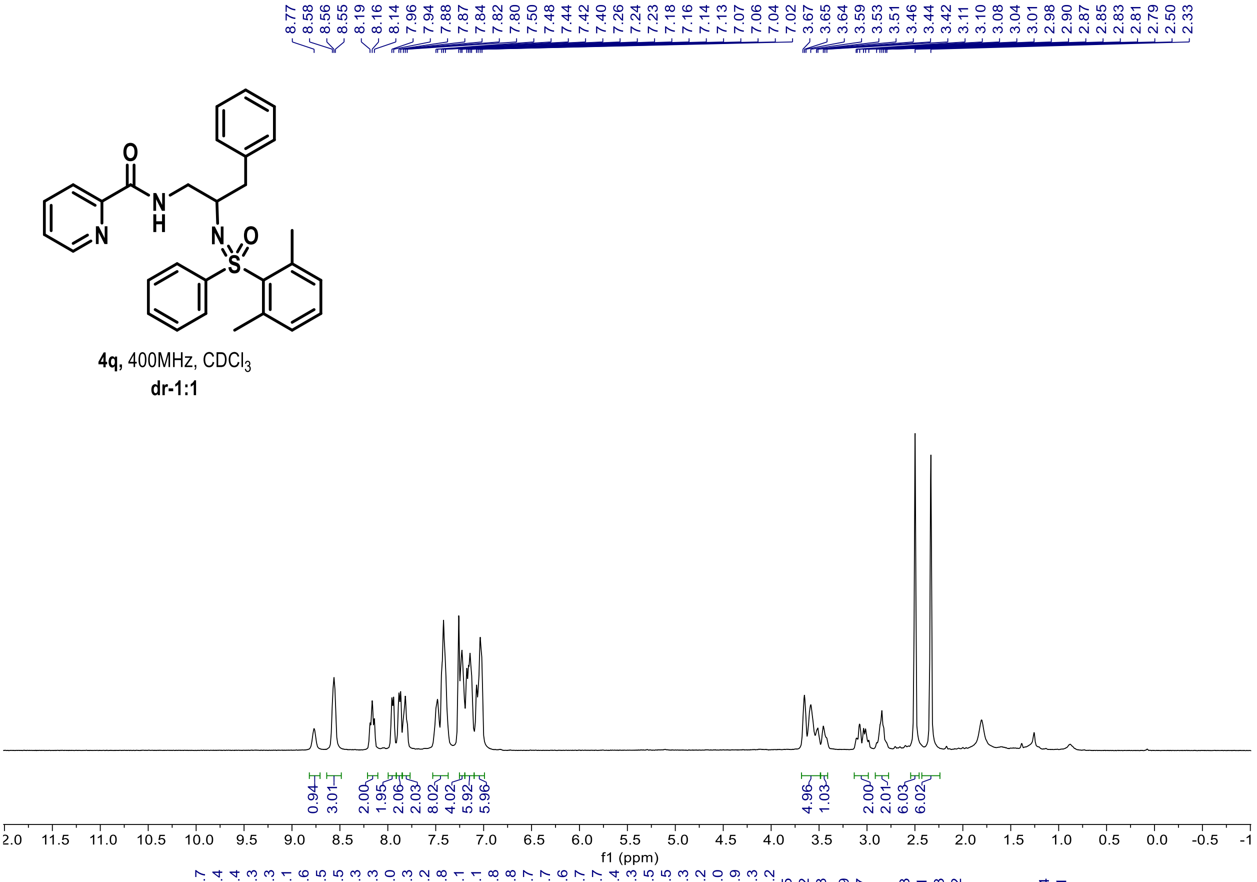
8.77 8.58 8.56 8.55 8.19 8.16 8.14 7.96 7.94 7.88 7.87 7.84 7.82 7.80 7.50 7.48 7.44 7.42 7.40 7.26 7.24 7.23 7.18 7.16 7.14 7.13 7.07 7.06 7.04 7.02 3.67 3.65 3.64 3.59 3.53 3.51 3.46 3.44 3.42 3.11 3.10 3.08 3.04 3.01 2.98 2.90 2.87 2.85 2.83 2.81 2.79 2.50 2.33
4q, 400MHz, CDCl$_3$
dr-1:1
0.94 3.01 2.00 1.95 2.06 2.03 8.02 4.02 5.92 5.96 4.96 1.03 2.00 2.01 6.03 6.02
f1 (ppm)

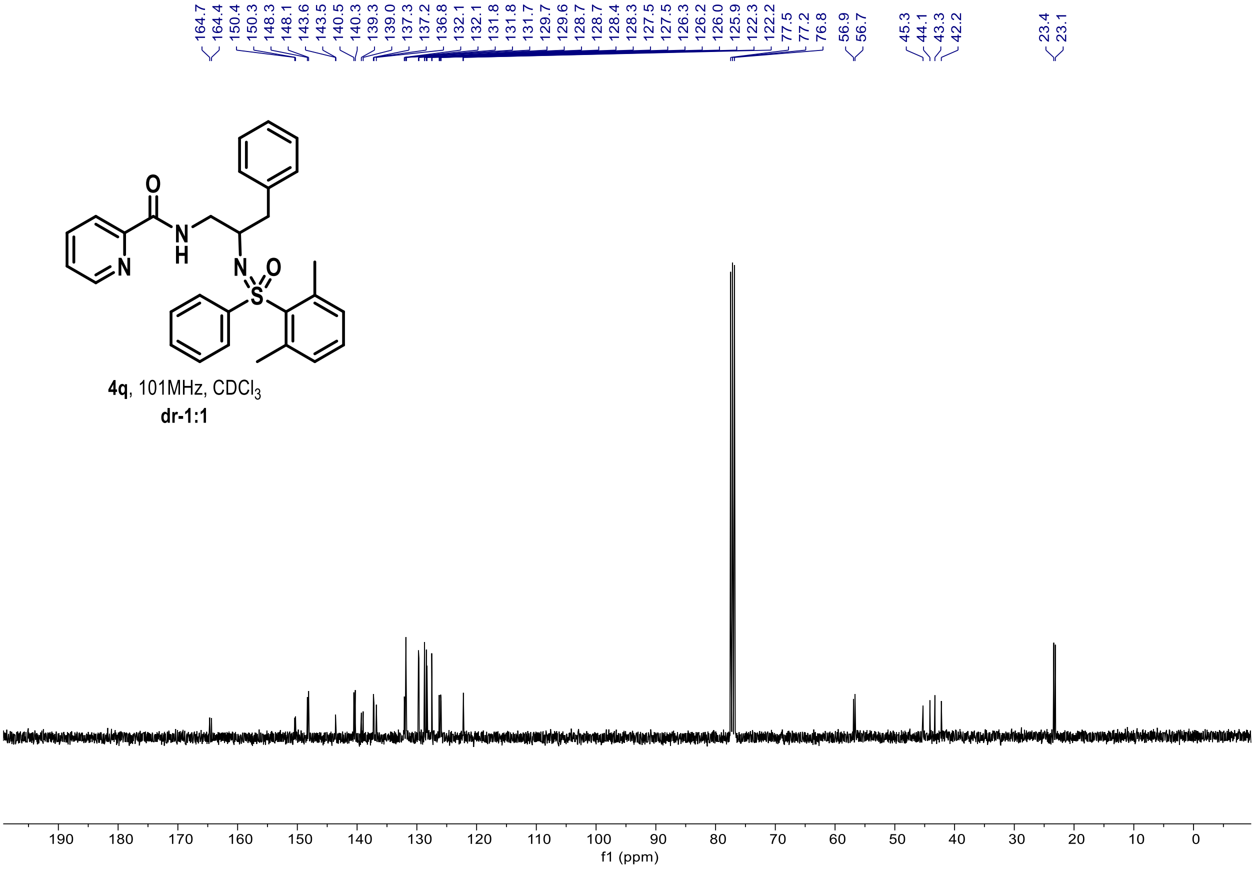
164.7 164.4 150.4 150.3 148.3 148.1 143.6 143.5 140.5 140.3 139.3 139.0 137.3 137.2 136.8 132.1 132.1 131.8 131.7 129.7 129.6 128.7 128.7 128.4 128.3 127.5 127.5 126.3 126.2 126.0 125.9 122.3 122.2 77.5 77.2 76.8 56.9 56.7 45.3 44.1 43.3 42.2 23.4 23.1
4q, 101MHz, CDCl$_3$
dr-1:1
f1 (ppm)

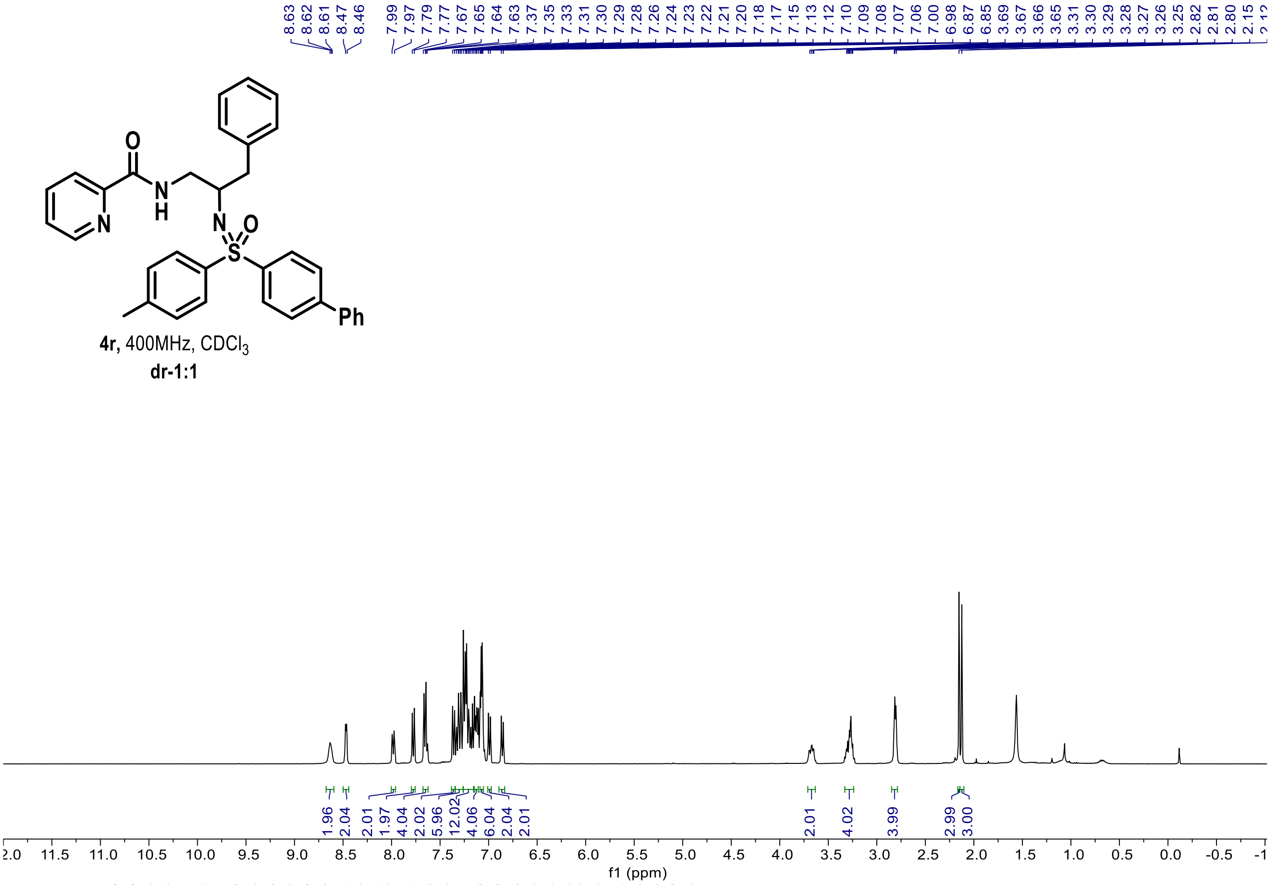
4r, 400MHz, CDCl3
dr-1:1
f1 (ppm)

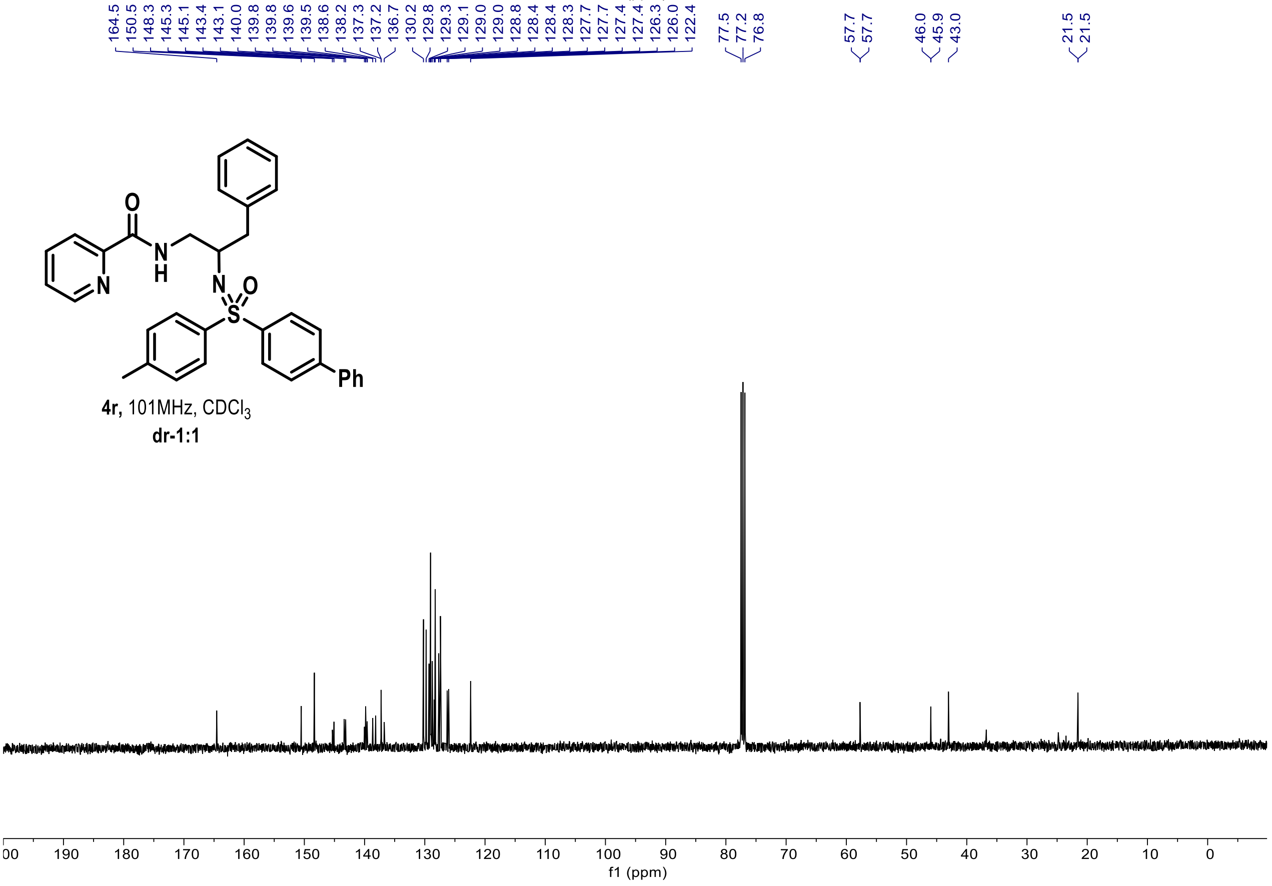
4r, 101MHz, CDCl3
dr-1:1
f1 (ppm)

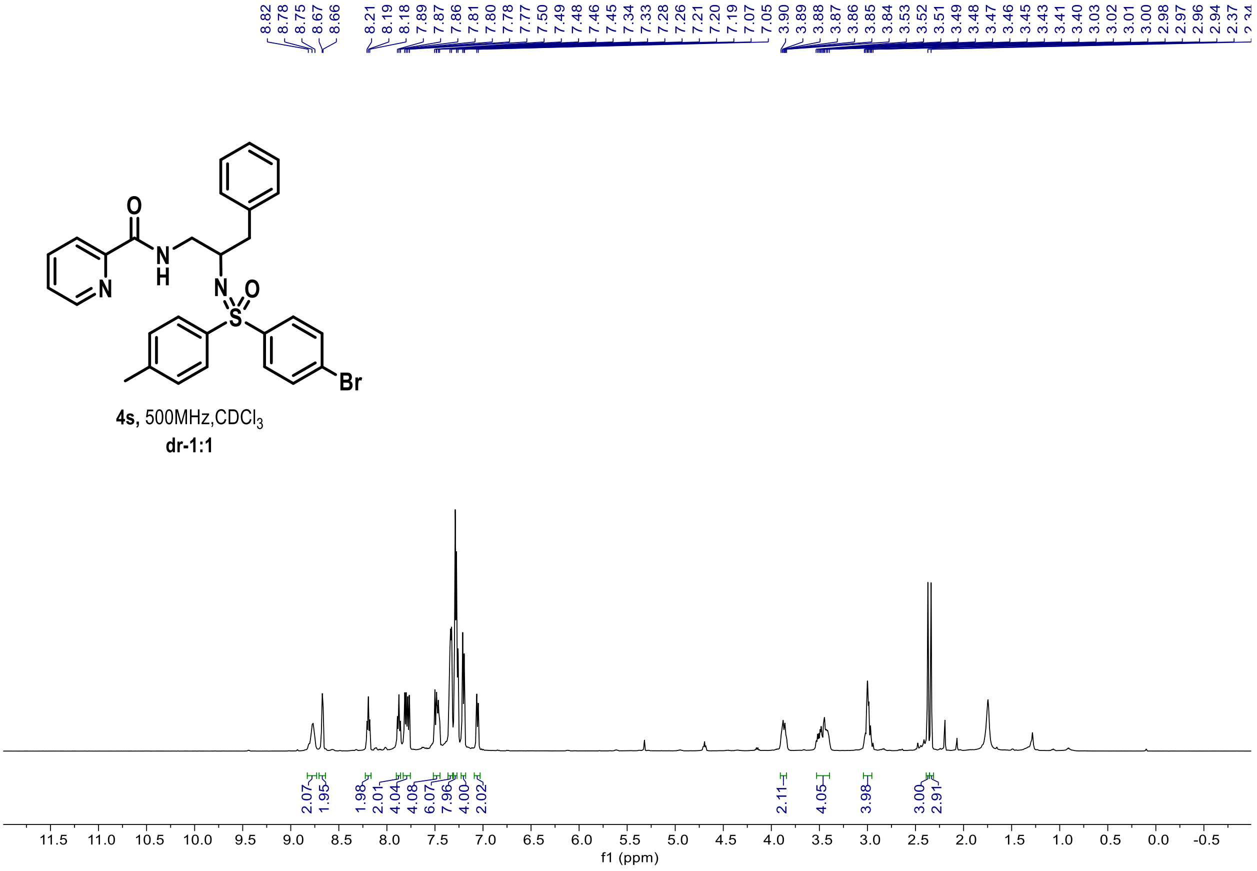
8.82 8.78 8.75 8.67 8.66 8.21 8.19 8.18 7.89 7.87 7.86 7.81 7.80 7.78 7.77 7.50 7.49 7.48 7.46 7.45 7.34 7.33 7.28 7.26 7.21 7.20 7.19 7.07 7.05 3.90 3.89 3.88 3.87 3.86 3.85 3.84 3.53 3.52 3.51 3.49 3.48 3.47 3.46 3.45 3.43 3.41 3.40 3.03 3.02 3.01 3.00 2.98 2.97 2.96 2.94 2.37
4s, 500MHz,CDCl3
dr-1:1
2.07 1.95 1.98 2.01 4.04 4.08 6.07 7.96 4.00 2.02 2.11 4.05 3.98 3.00 2.91
11.5 11.0 10.5 10.0 9.5 9.0 8.5 8.0 7.5 7.0 6.5 6.0 5.5 5.0 4.5 4.0 3.5 3.0 2.5 2.0 1.5 1.0 0.5 0.0 -0.5
f1 (ppm)

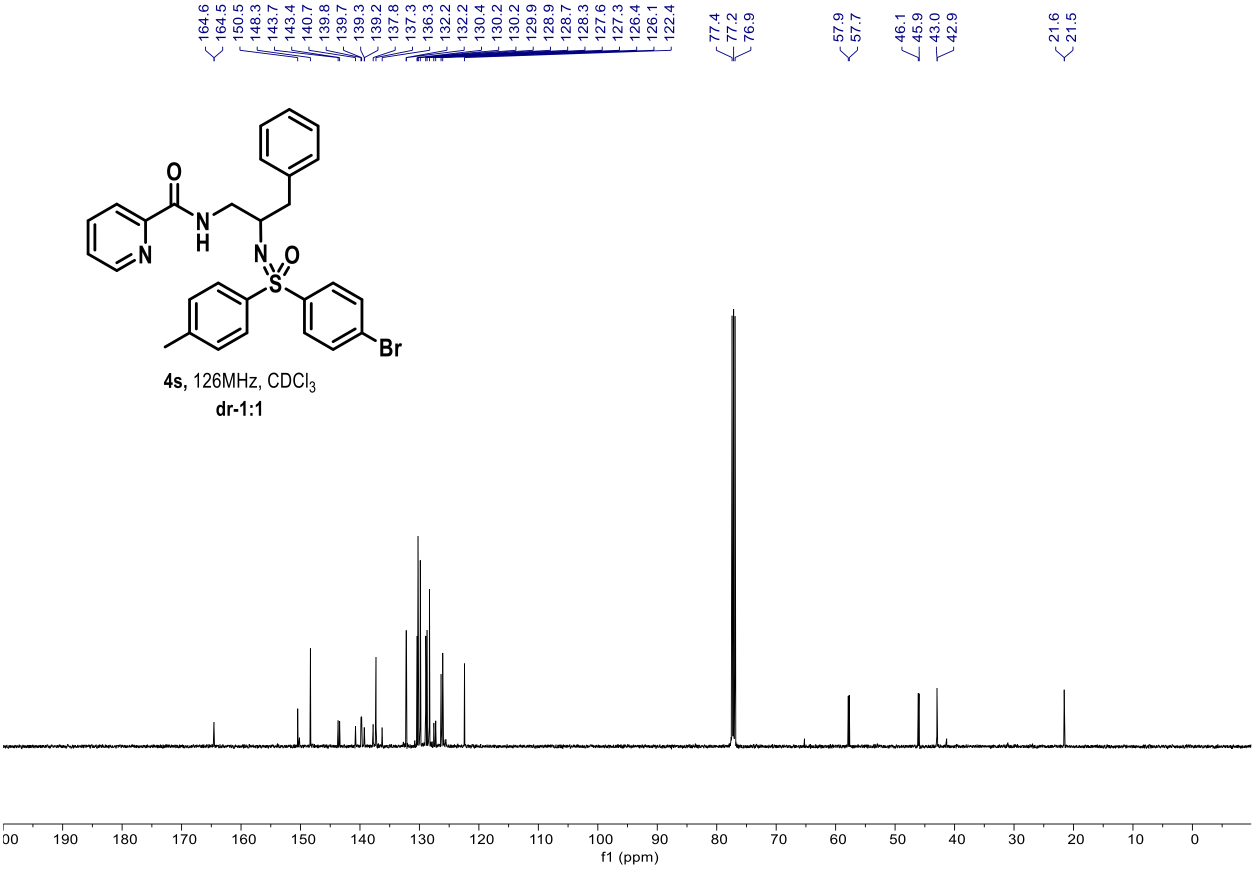
164.6 164.5 150.5 148.3 143.7 143.4 140.7 139.8 139.7 139.3 139.2 137.8 137.3 136.3 132.2 132.2 130.4 130.2 130.2 129.9 128.9 128.7 128.3 127.6 127.3 126.4 126.1 122.4 77.4 77.2 76.9 57.9 57.7 46.1 45.9 43.0 42.9 21.6 21.5
4s, 126MHz, CDCl3
dr-1:1
190 180 170 160 150 140 130 120 110 100 90 80 70 60 50 40 30 20 10 0
f1 (ppm)

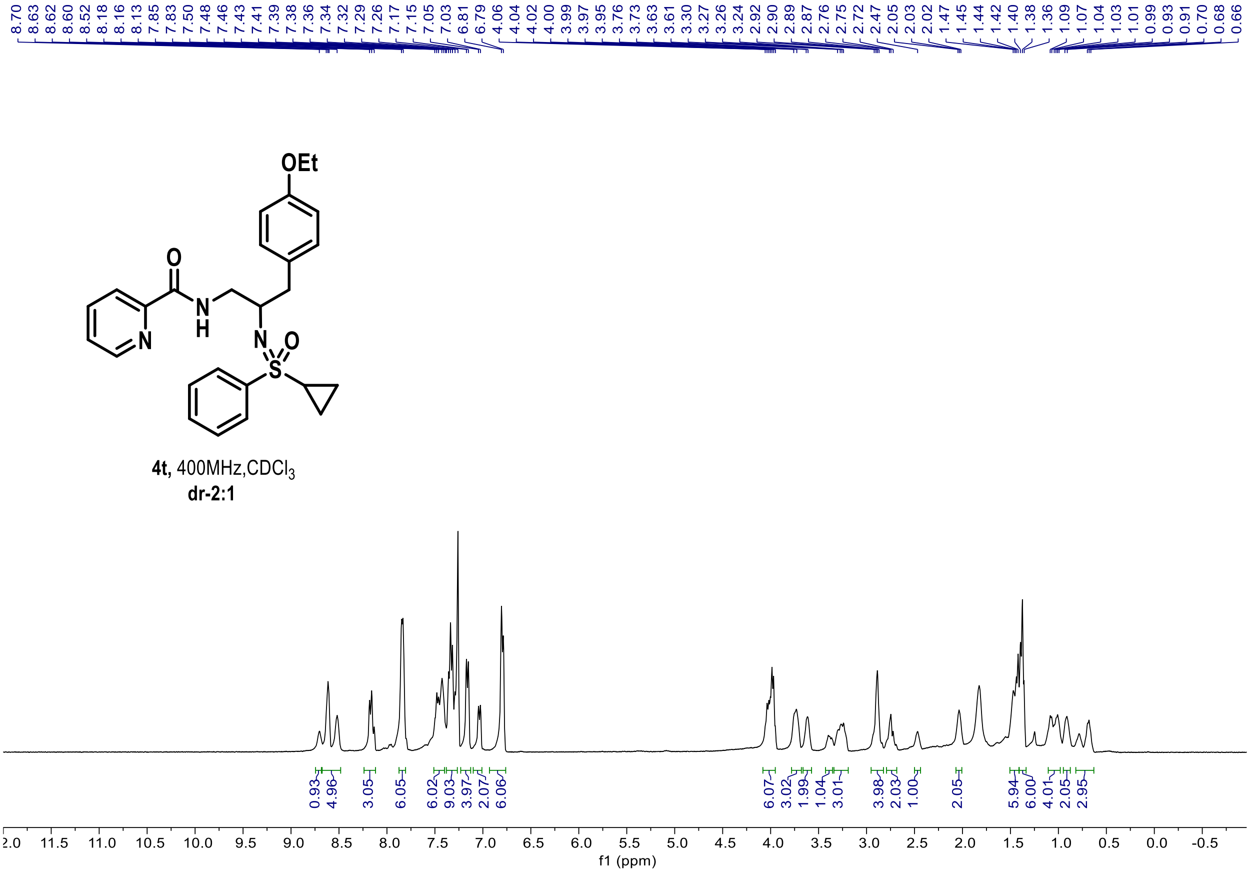
8.70 8.63 8.62 8.60 8.52 8.18 8.16 8.13 7.85 7.83 7.50 7.48 7.46 7.43 7.41 7.39 7.38 7.36 7.34 7.32 7.29 7.26 7.17 7.15 7.05 7.03 6.81 6.79 4.06 4.04 4.02 4.00 3.99 3.97 3.95 3.76 3.73 3.63 3.61 3.30 3.27 3.26 3.24 2.92 2.90 2.89 2.87 2.76 2.75 2.72 2.47 2.05 2.03 2.02 1.47 1.45 1.44 1.42 1.40 1.38 1.36 1.09 1.07 1.04 1.03 1.01 0.99 0.93 0.91 0.70 0.68 0.66
OEt
O
N
H
N
O
S
4t, 400MHz,CDCl3
dr-2:1
0.93 4.96 3.05 6.05 6.02 9.03 3.97 2.07 6.06 6.07 3.02 1.99 1.04 3.01 3.98 2.03 1.00 2.05 5.94 6.00 4.01 2.05 2.95
f1 (ppm)

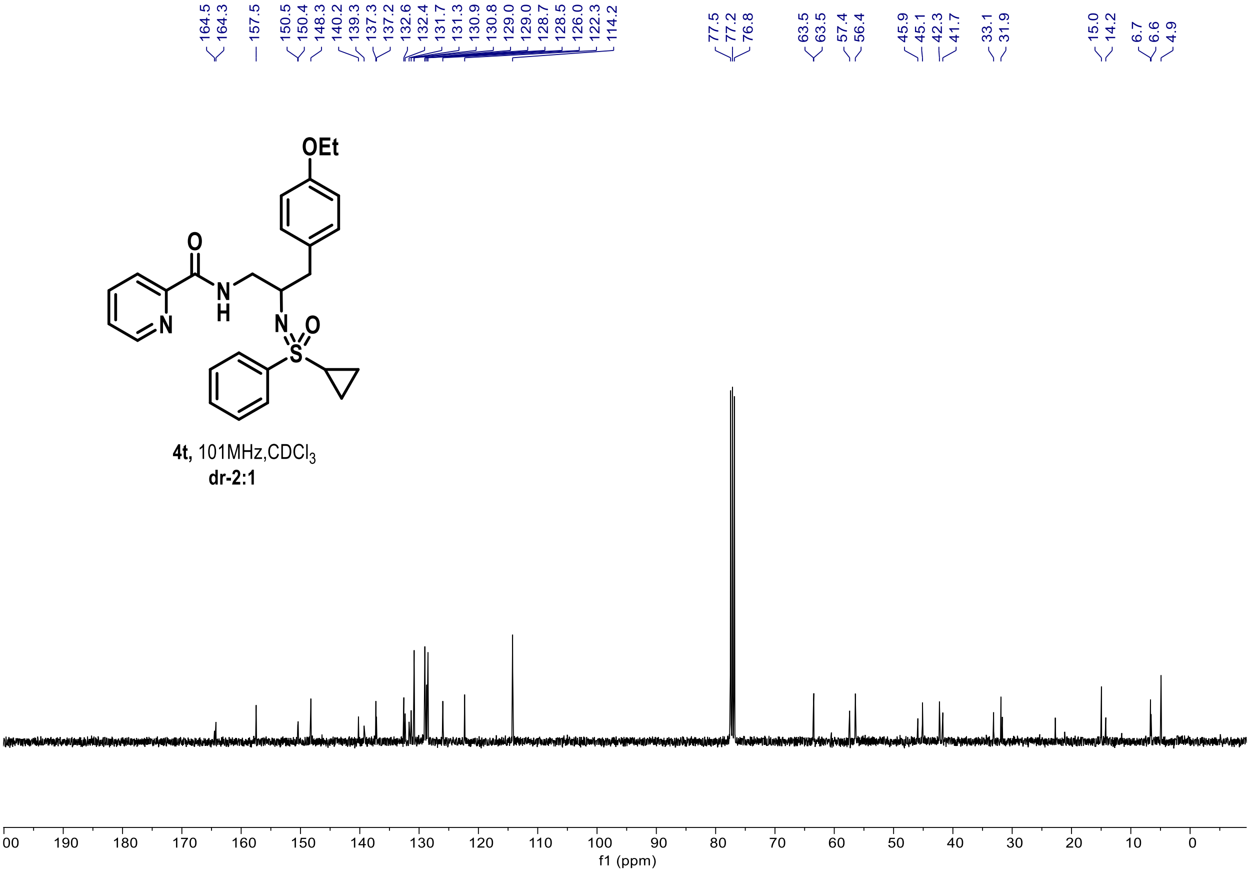
164.5 164.3 157.5 150.5 150.4 148.3 140.2 139.3 137.3 137.2 132.6 132.4 131.7 131.3 130.9 130.8 129.0 128.7 128.5 126.0 122.3 114.2 77.5 77.2 76.8 63.5 63.5 57.4 56.4 45.9 45.1 42.3 41.7 33.1 31.9 15.0 14.2 6.7 6.6 4.9
OEt
O
N
H
N
O
S
4t, 101MHz,CDCl3
dr-2:1
f1 (ppm)

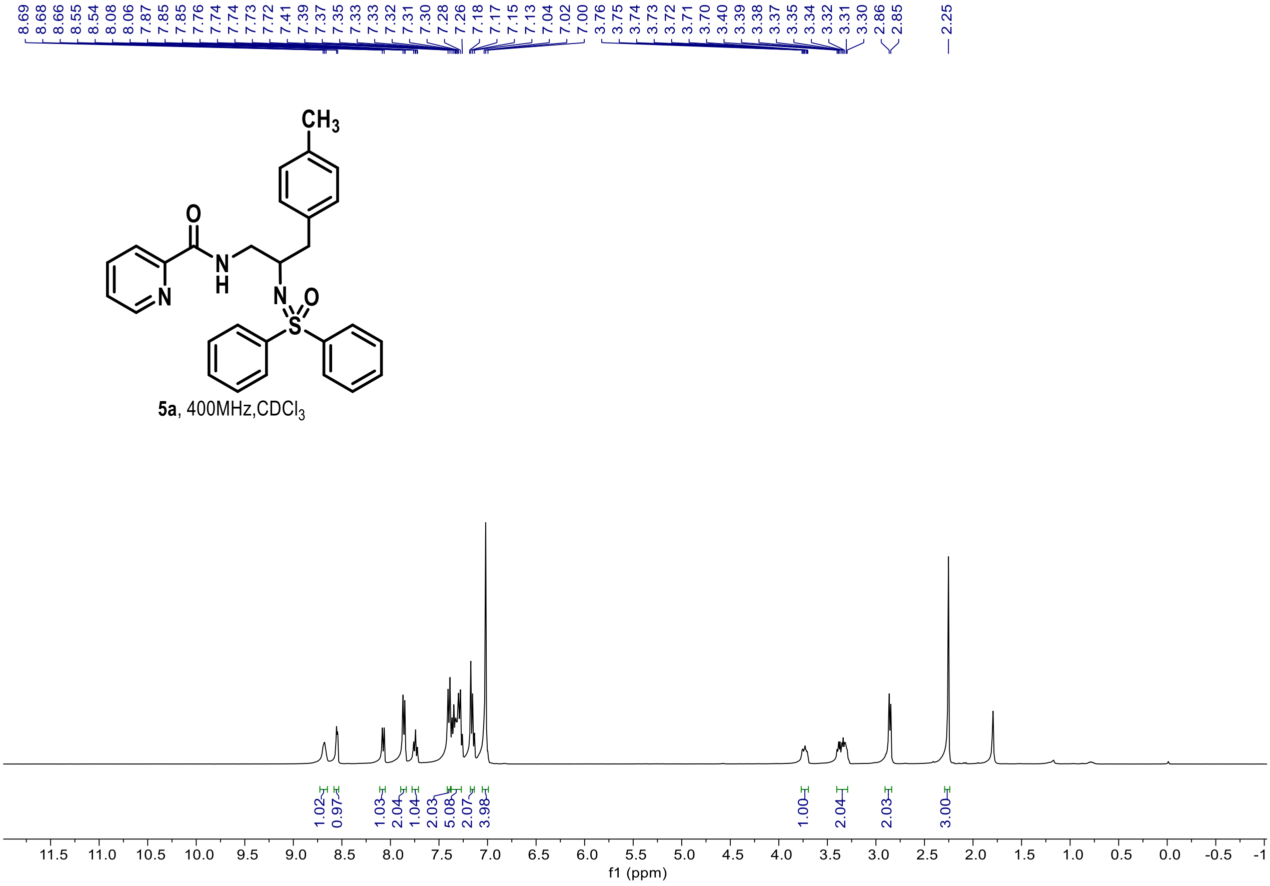
8.69 8.68 8.66 8.55 8.54 8.08 8.06 7.87 7.85 7.85 7.76 7.74 7.74 7.73 7.72 7.41 7.39 7.37 7.35 7.33 7.33 7.32 7.31 7.30 7.28 7.26 7.18 7.17 7.15 7.13 7.04 7.02 7.00 3.76 3.75 3.74 3.73 3.72 3.71 3.70 3.40 3.39 3.38 3.37 3.35 3.34 3.32 3.31 3.30 2.86 2.85 2.25
5a, 400MHz,CDCl3
1.02 0.97 1.03 2.04 1.04 2.03 5.08 2.07 3.98 1.00 2.04 2.03 3.00
f1 (ppm)

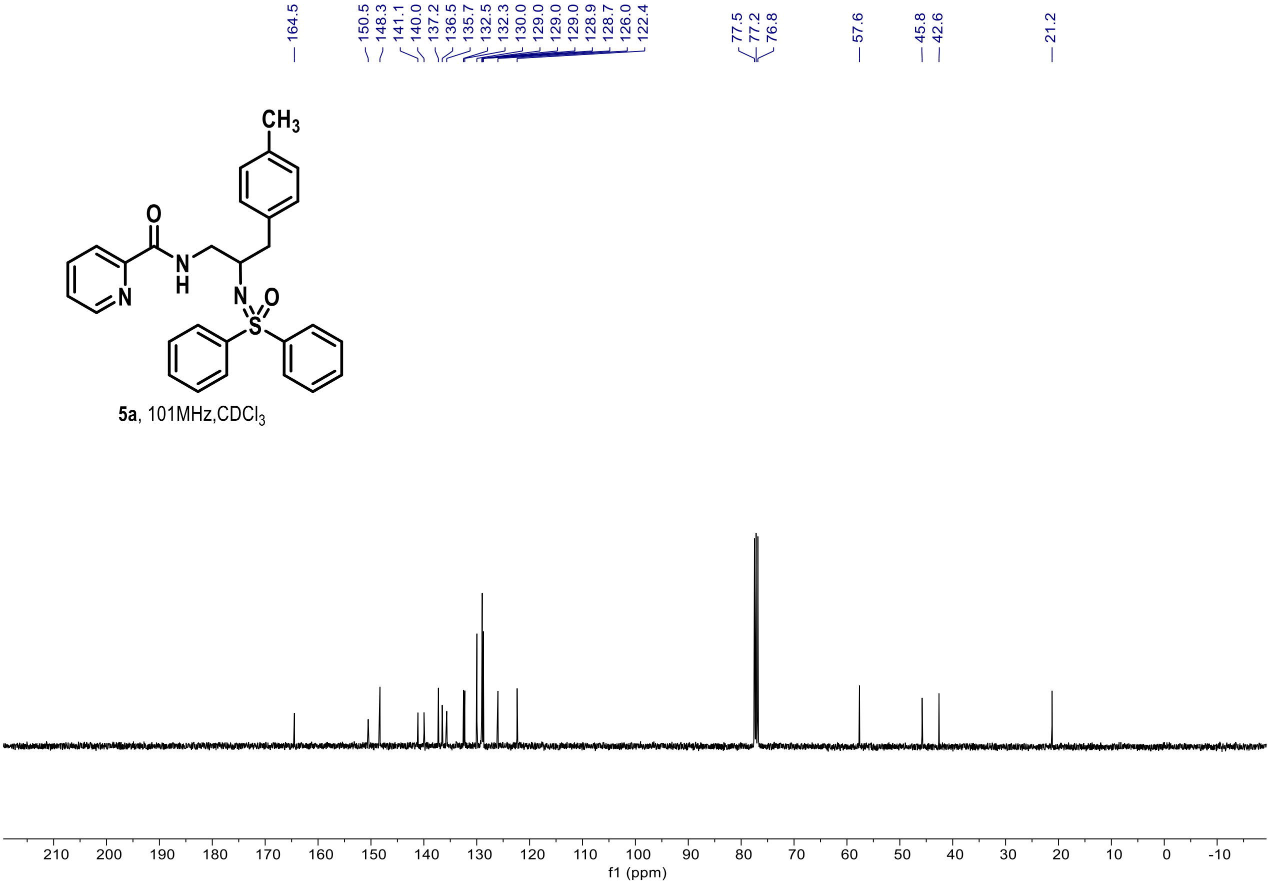
164.5 150.5 148.3 141.1 140.0 137.2 136.5 135.7 132.5 132.3 130.0 129.0 129.0 129.0 128.9 128.7 126.0 122.4 77.5 77.2 76.8 57.6 45.8 42.6 21.2
5a, 101MHz,CDCl3
f1 (ppm)

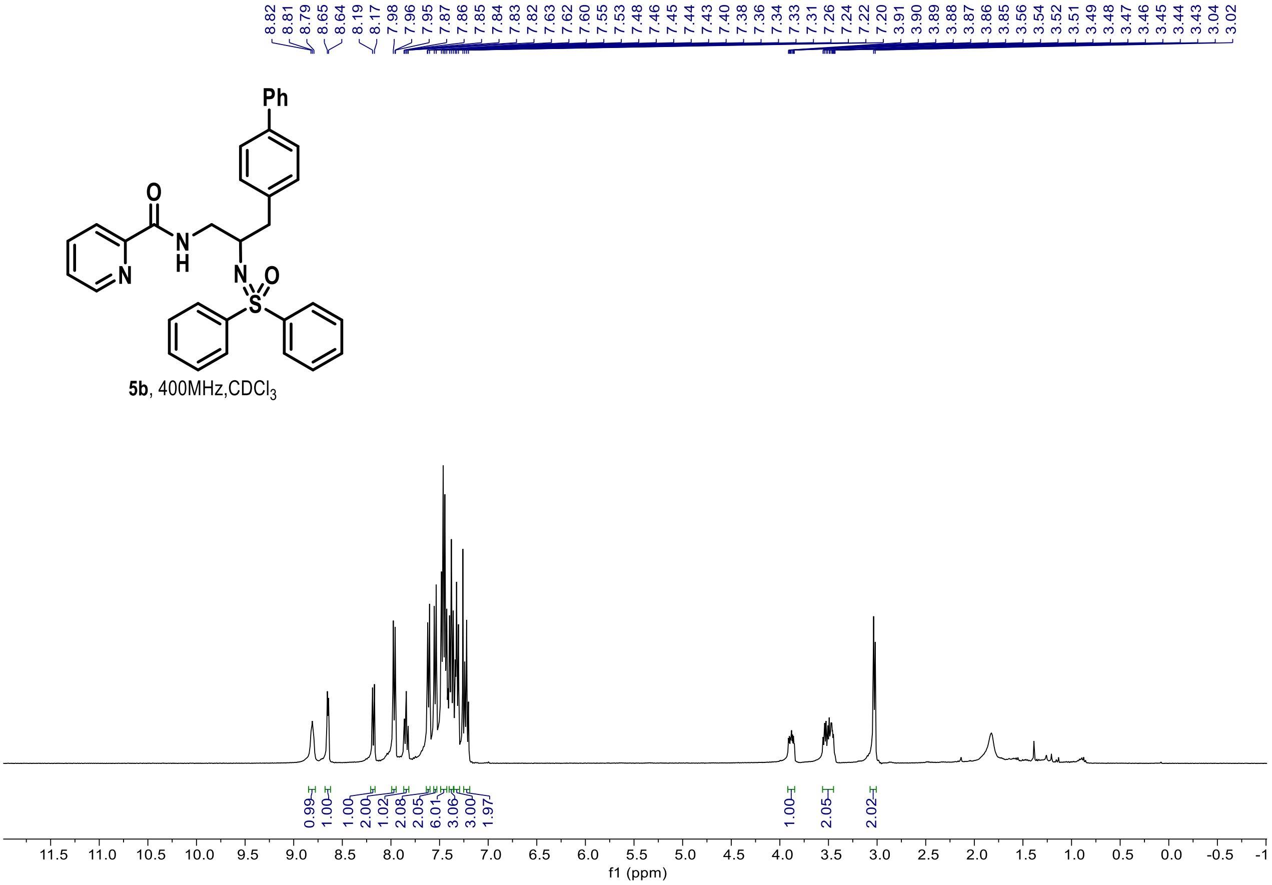
8.82 8.81 8.79 8.65 8.64 8.19 8.17 7.98 7.96 7.95 7.87 7.86 7.85 7.84 7.83 7.82 7.63 7.62 7.60 7.55 7.53 7.48 7.46 7.45 7.44 7.43 7.40 7.38 7.36 7.34 7.33 7.31 7.26 7.24 7.22 7.20 3.91 3.90 3.89 3.88 3.87 3.86 3.85 3.56 3.54 3.52 3.51 3.49 3.48 3.47 3.46 3.45 3.44 3.43 3.04 3.02
Ph
O
N
H
N
N
S
O
5b, 400MHz,CDCl$_3$
0.99 1.00 1.00 2.00 1.02 2.08 2.05 6.01 3.06 3.00 1.97 1.00 2.05 2.02
f1 (ppm)

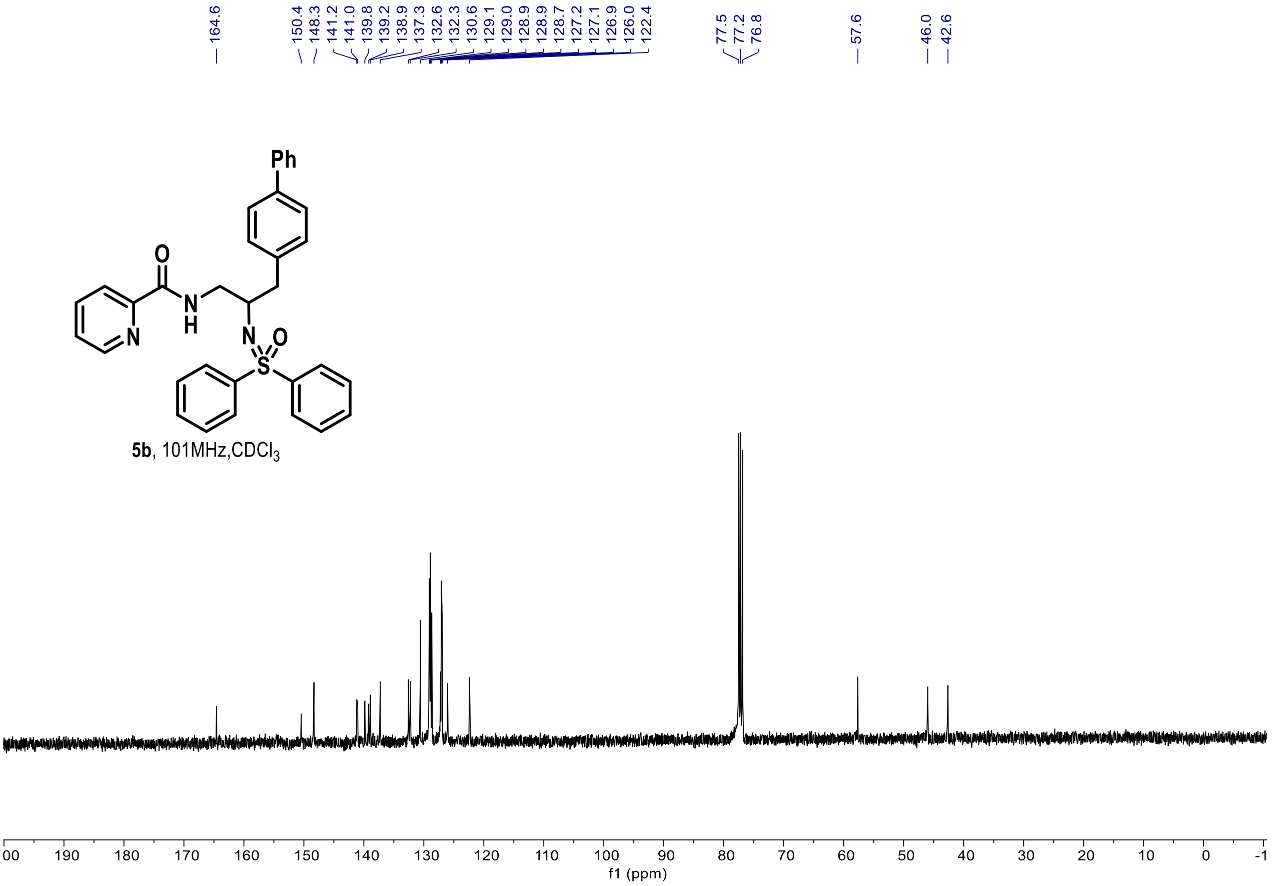
164.6 150.4 148.3 141.2 141.0 139.8 139.2 138.9 137.3 132.6 132.3 130.6 129.1 129.0 128.9 128.9 128.7 127.2 127.1 126.9 126.0 122.4 77.5 77.2 76.8 57.6 46.0 42.6
Ph
O
N
H
N
N
S
O
5b, 101MHz,CDCl$_3$
f1 (ppm)

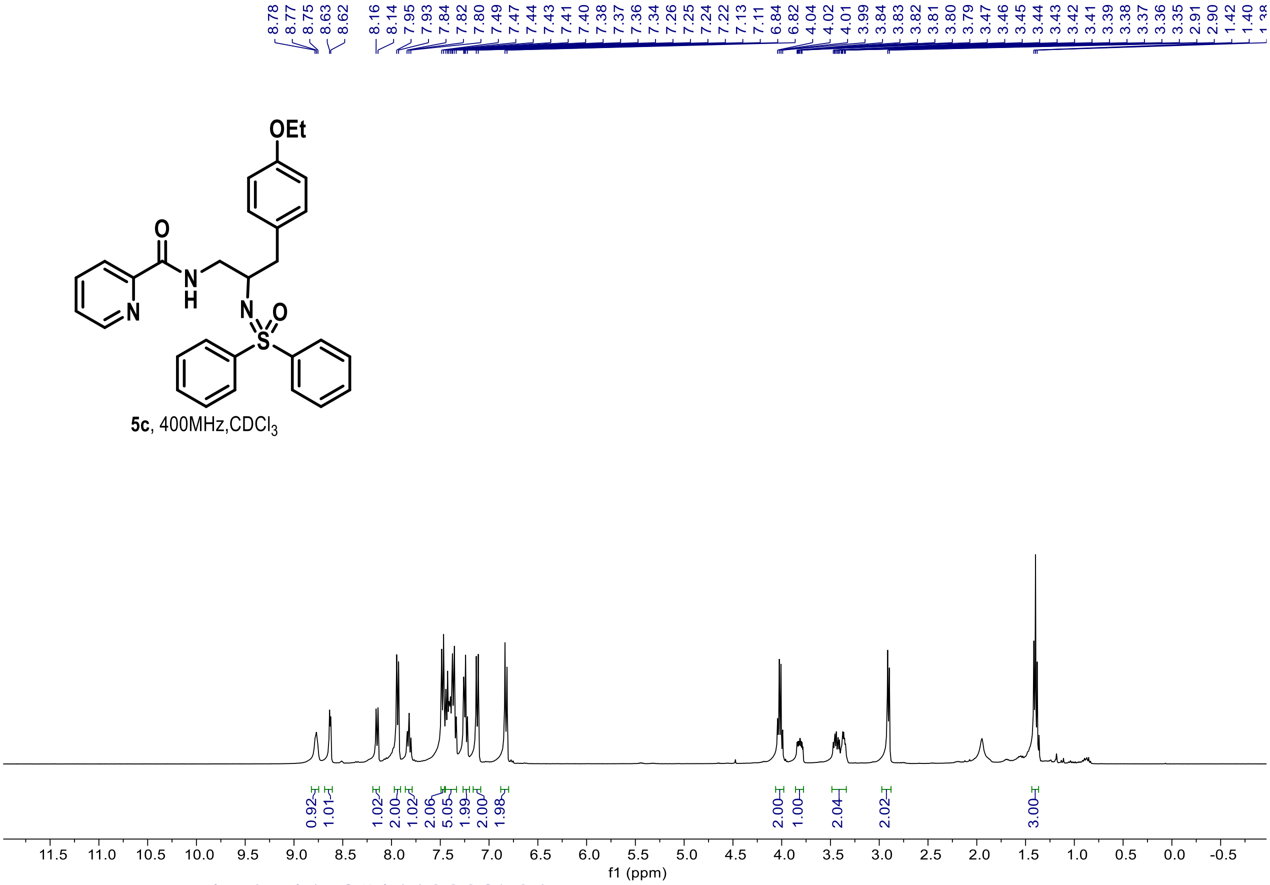
8.78 8.77 8.75 8.63 8.62 8.16 8.14 7.95 7.93 7.84 7.82 7.80 7.49 7.47 7.44 7.43 7.41 7.40 7.38 7.37 7.36 7.34 7.26 7.25 7.24 7.22 7.13 7.11 6.84 6.82 4.04 4.02 4.01 3.99 3.84 3.83 3.82 3.81 3.80 3.79 3.47 3.46 3.45 3.44 3.43 3.42 3.41 3.39 3.38 3.37 3.36 3.35 2.91 2.90 1.42 1.40
OEt
O
N
H
N
N
O
S
5c, 400MHz,CDCl$_3$
0.92 1.01 1.02 2.00 1.02 2.06 5.05 1.99 2.00 1.98 2.00 1.00 2.04 2.02 3.00
11.5 11.0 10.5 10.0 9.5 9.0 8.5 8.0 7.5 7.0 6.5 6.0 5.5 5.0 4.5 4.0 3.5 3.0 2.5 2.0 1.5 1.0 0.5 0.0 -0.5
f1 (ppm)

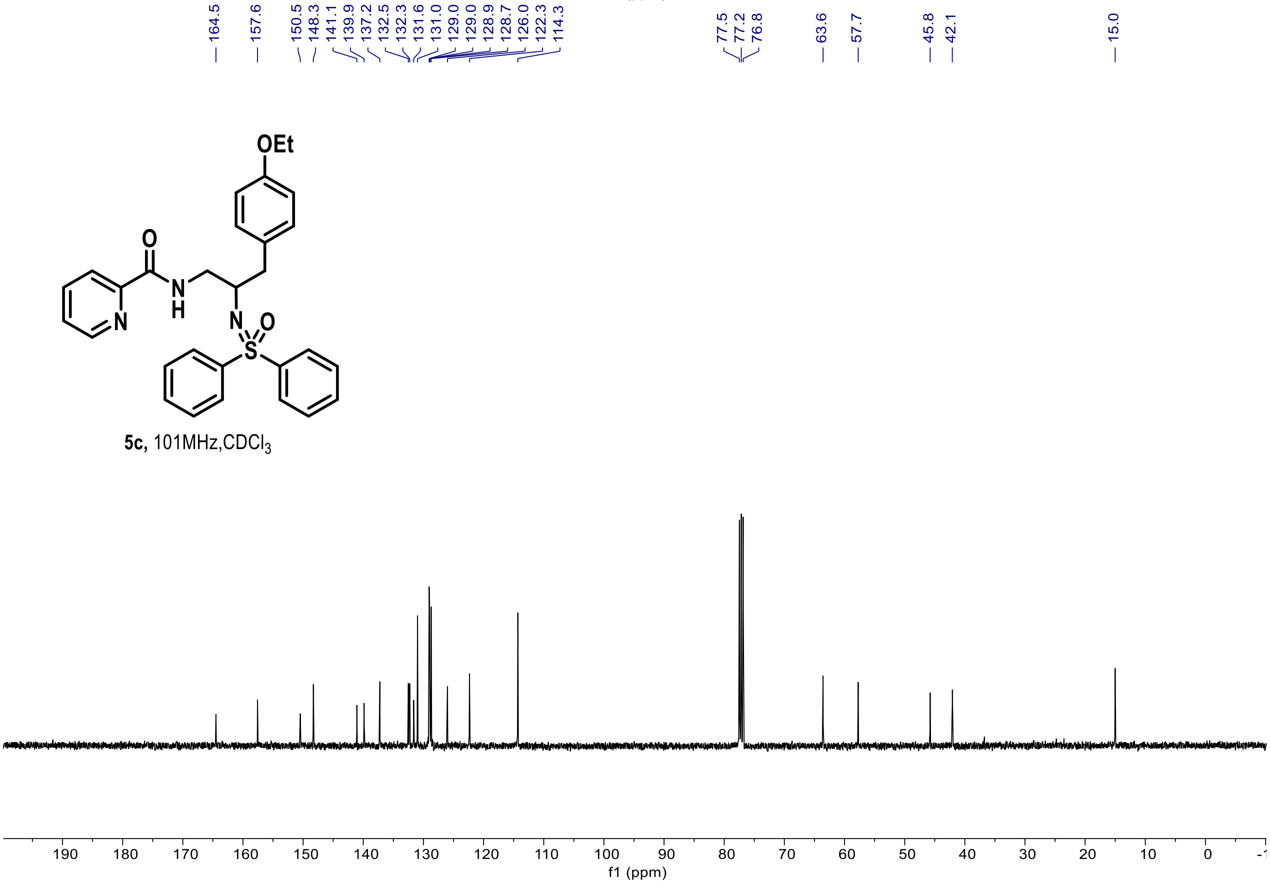
164.5 157.6 150.5 148.3 141.1 139.9 137.2 132.5 132.3 131.6 131.0 129.0 129.0 128.9 128.7 126.0 122.3 114.3 77.5 77.2 76.8 63.6 57.7 45.8 42.1 15.0
OEt
O
N
H
N
N
O
S
5c, 101MHz,CDCl$_3$
190 180 170 160 150 140 130 120 110 100 90 80 70 60 50 40 30 20 10 0
f1 (ppm)

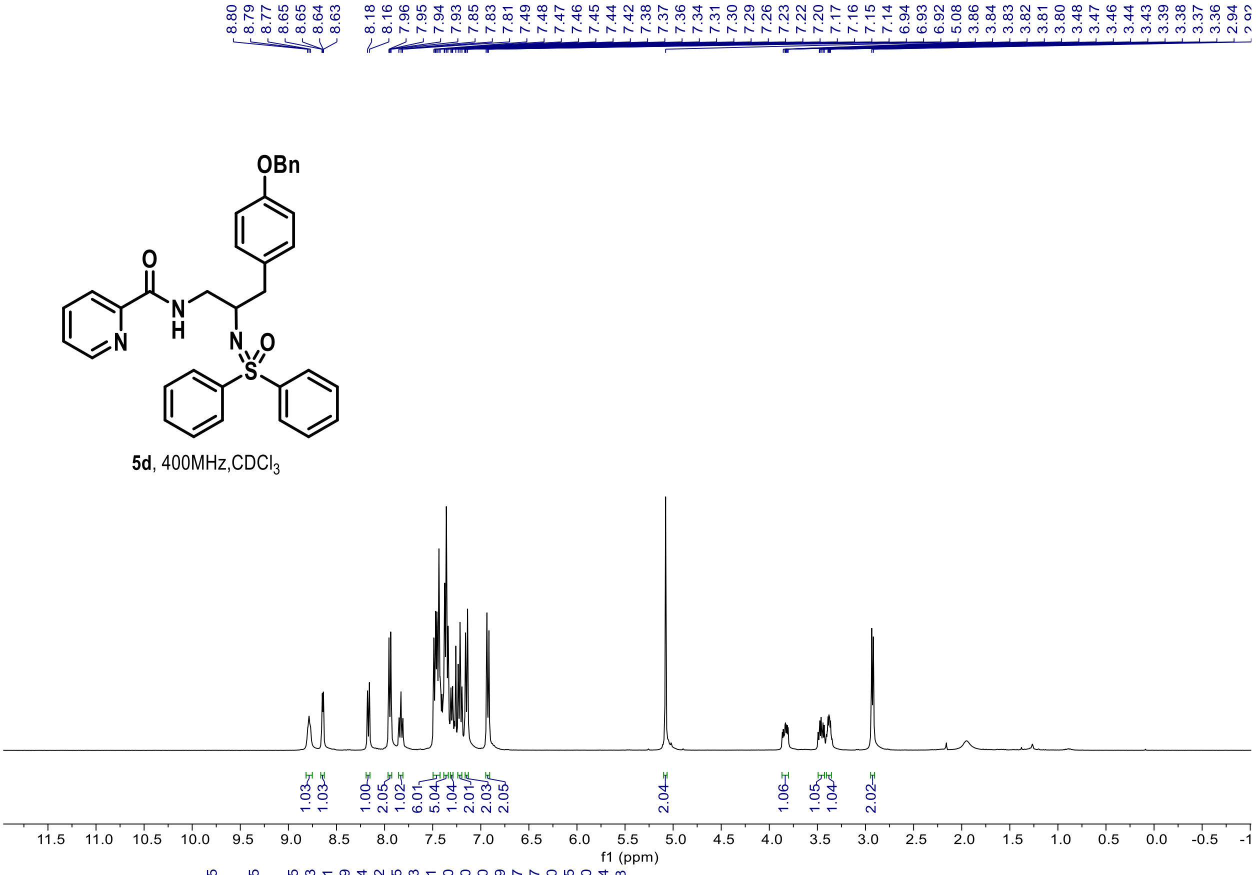
8.80 8.79 8.77 8.65 8.65 8.64 8.63 8.18 8.16 7.96 7.95 7.94 7.93 7.85 7.83 7.81 7.49 7.48 7.47 7.46 7.45 7.44 7.42 7.38 7.37 7.36 7.34 7.31 7.30 7.29 7.26 7.23 7.22 7.20 7.17 7.16 7.15 7.14 6.94 6.93 6.92 5.08 3.86 3.84 3.83 3.82 3.81 3.80 3.48 3.47 3.46 3.44 3.43 3.39 3.38 3.37 3.36 2.94
OBn
O
N
H
N
N
O
S
5d, 400MHz,CDCl$_3$
1.03 1.03 1.00 2.05 1.02 6.01 5.04 1.04 2.01 2.03 2.05 2.04 1.06 1.05 1.04 2.02
11.5 11.0 10.5 10.0 9.5 9.0 8.5 8.0 7.5 7.0 6.5 6.0 5.5 5.0 4.5 4.0 3.5 3.0 2.5 2.0 1.5 1.0 0.5 0.0 -0.5 -1
f1 (ppm)

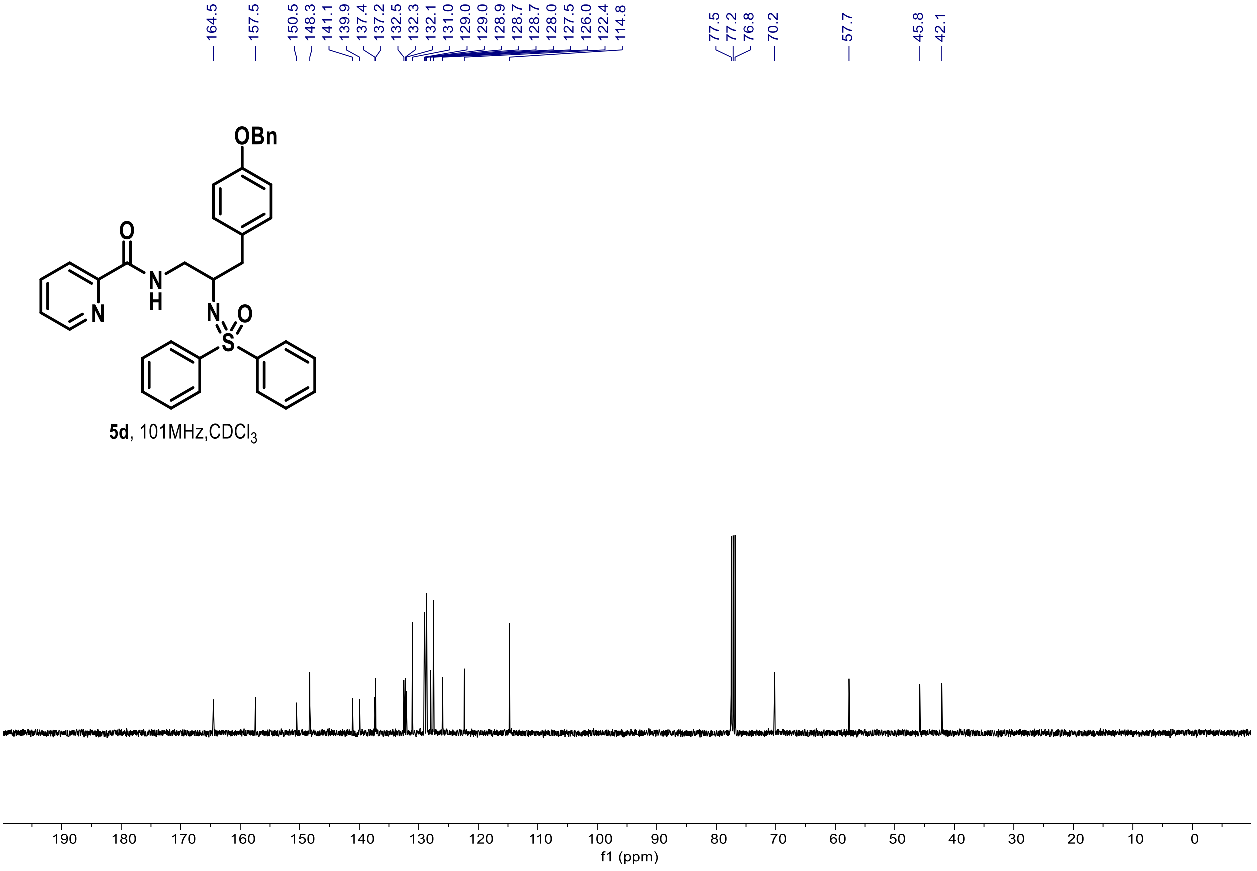
164.5 157.5 150.5 148.3 141.1 139.9 137.4 137.2 132.5 132.3 132.1 131.0 129.0 129.0 128.9 128.7 128.7 128.0 127.5 126.0 122.4 114.8 77.5 77.2 76.8 70.2 57.7 45.8 42.1
OBn
O
N
H
N
N
O
S
5d, 101MHz,CDCl$_3$
190 180 170 160 150 140 130 120 110 100 90 80 70 60 50 40 30 20 10 0
f1 (ppm)

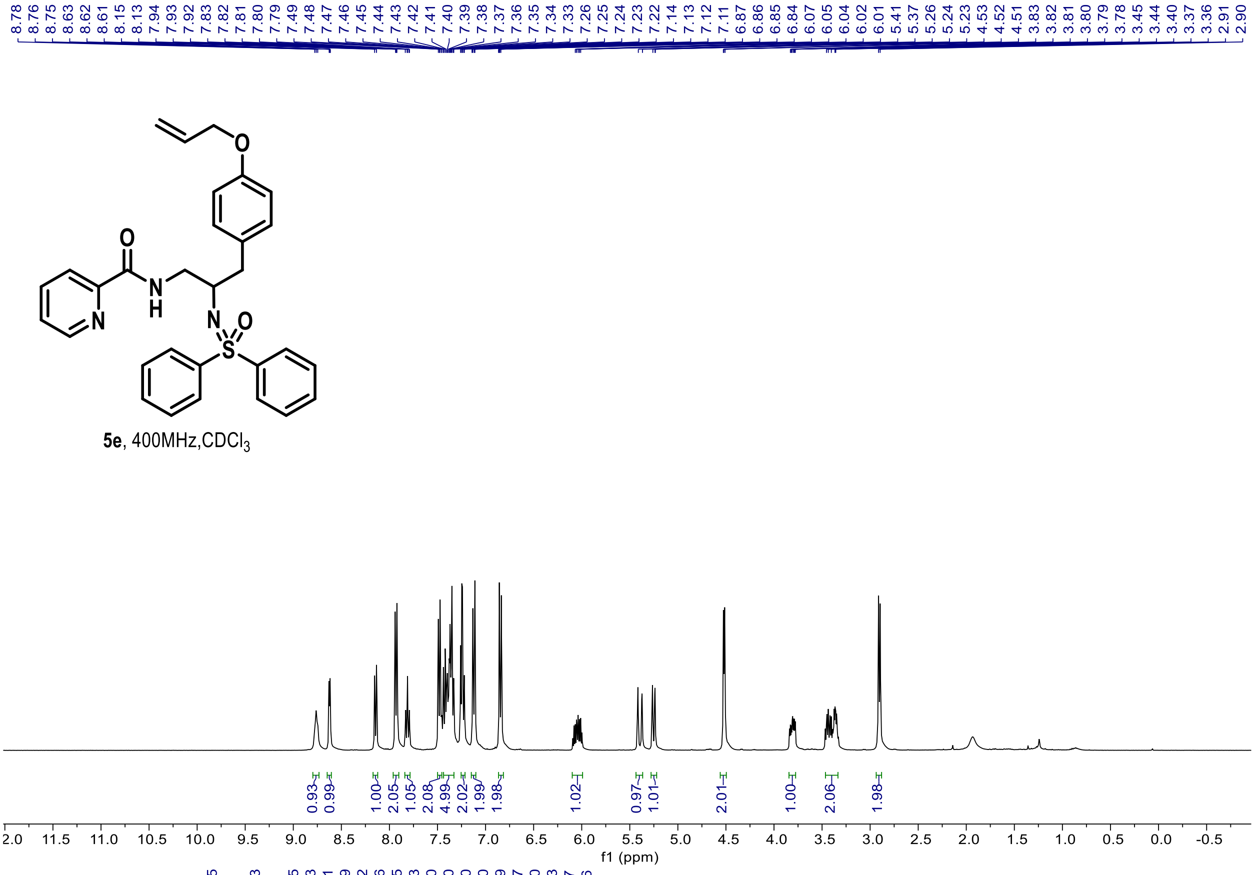
5e, 400MHz, $CDCl_3$
f1 (ppm)

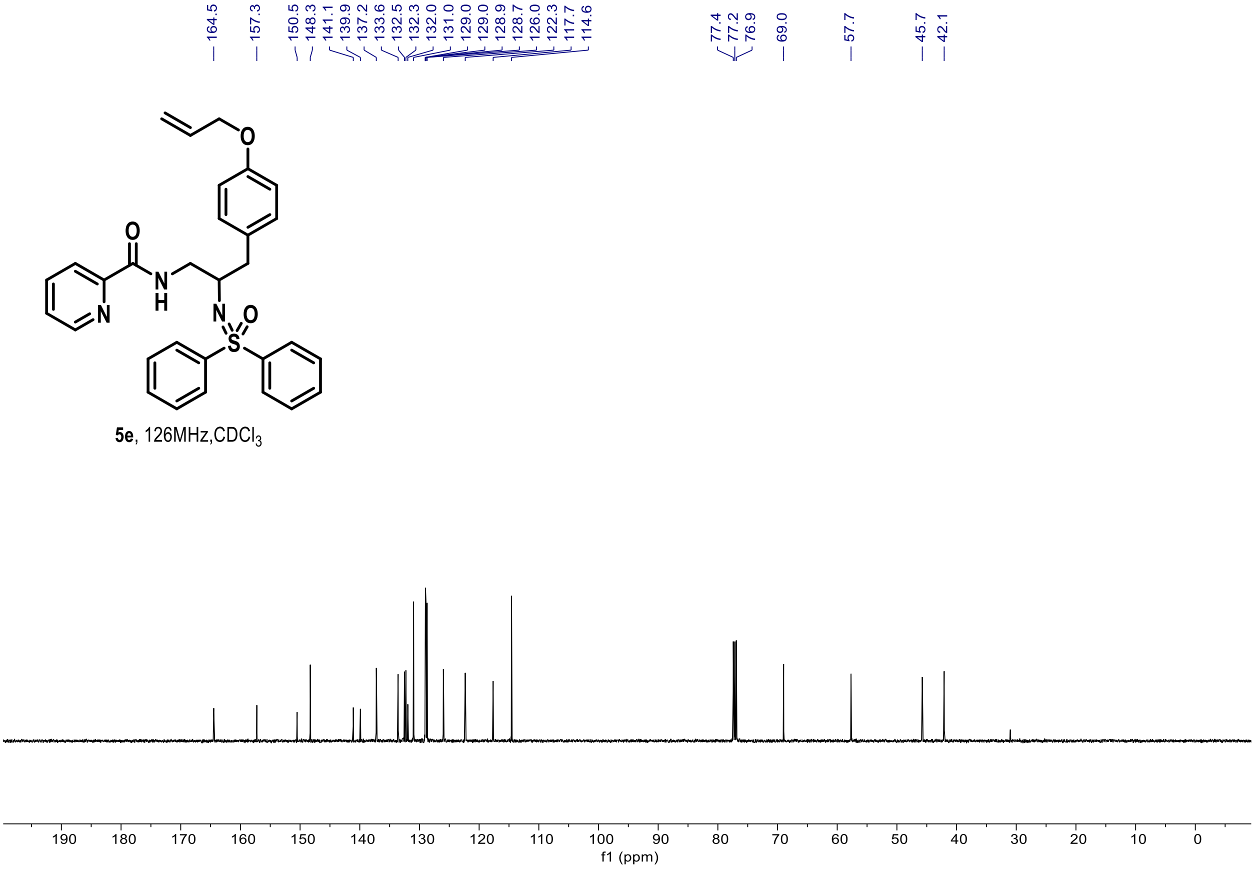
5e, 126MHz, $CDCl_3$
164.5
157.3
150.5
148.3
141.1
139.9
137.2
133.6
132.5
132.3
132.0
131.0
129.0
129.0
128.9
128.7
126.0
122.3
117.7
114.6
77.4
77.2
76.9
69.0
57.7
45.7
42.1
f1 (ppm)

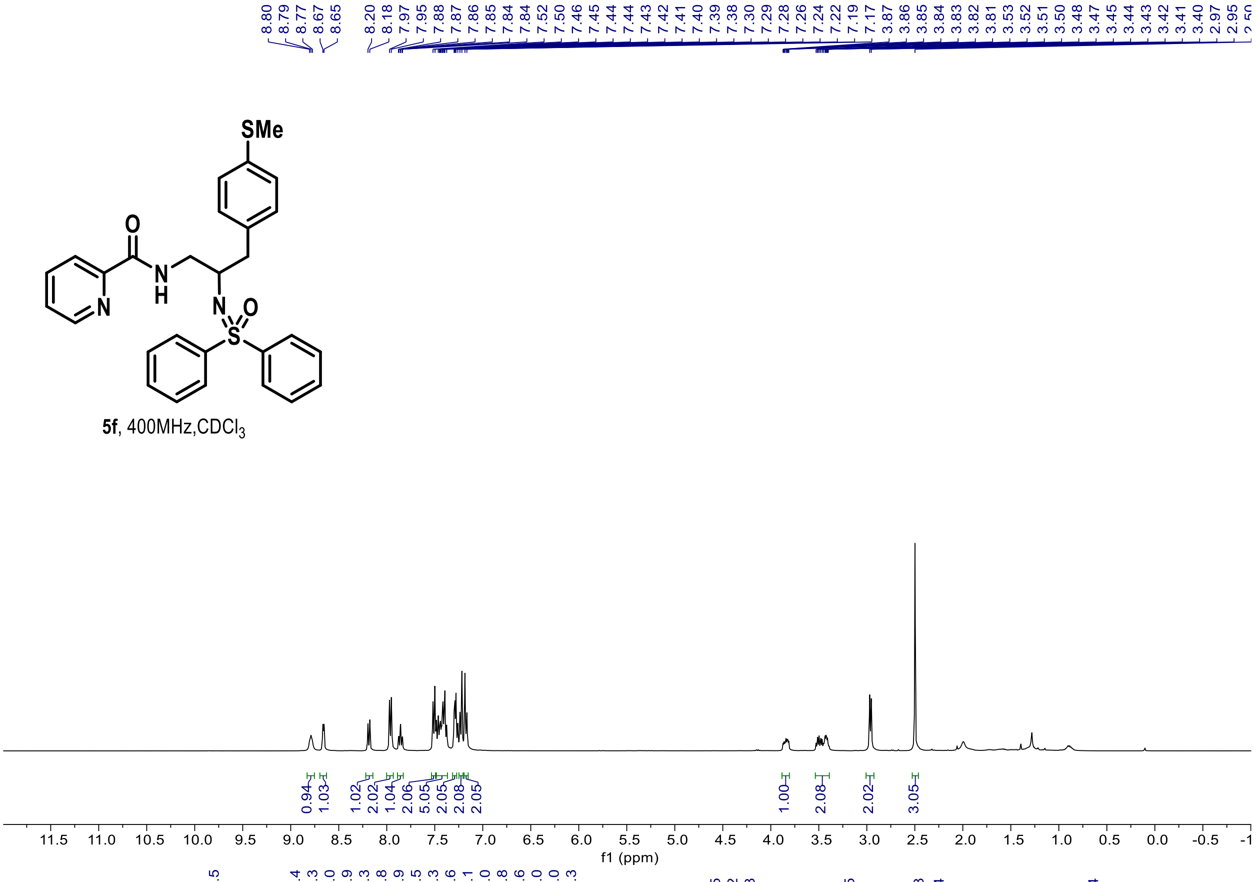
SMe
O
N
H
N
N
O
S
5f, 400MHz,CDCl3
f1 (ppm)

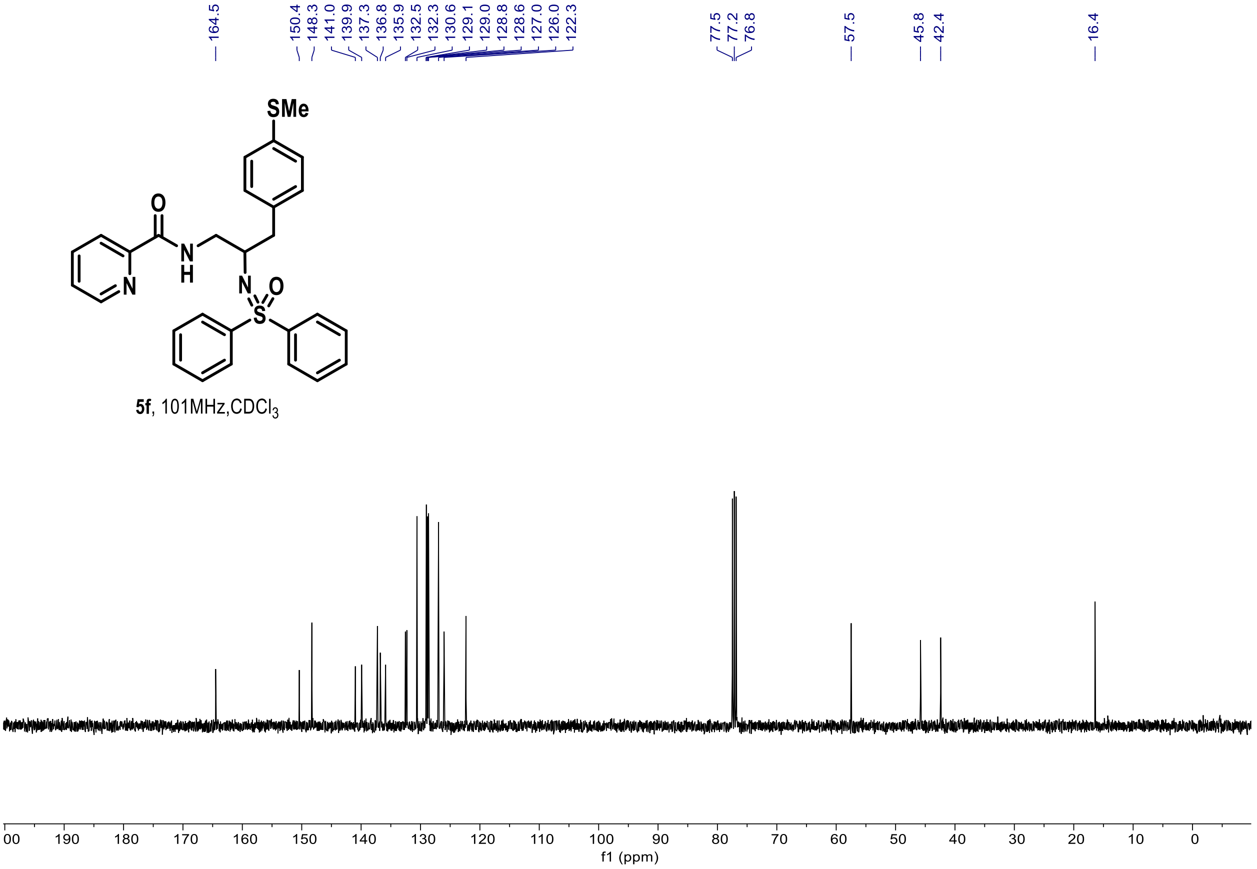
SMe
O
N
H
N
N
O
S
5f, 101MHz,CDCl3
f1 (ppm)

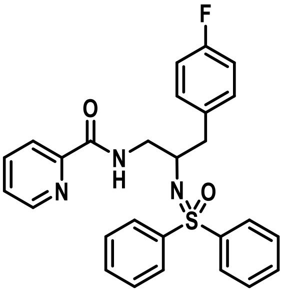

5g, 500MHz,CDCl$_3$

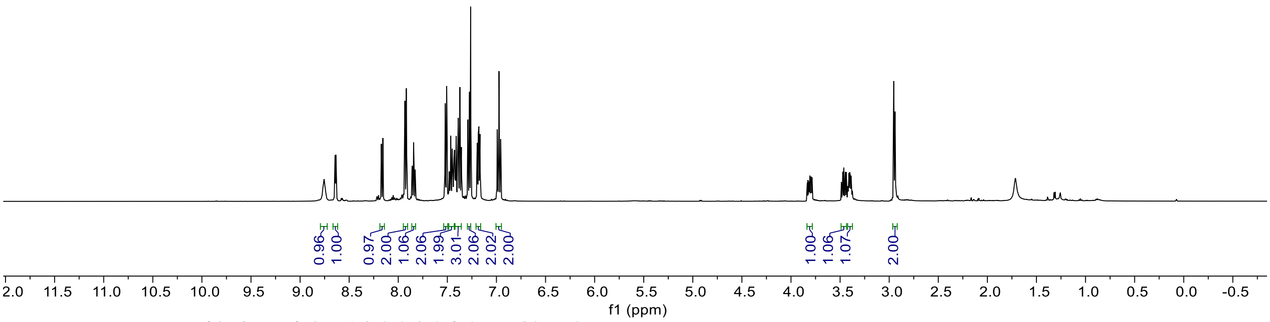

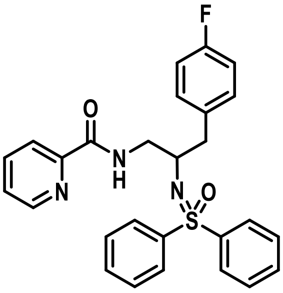

5g, 126MHz,CDCl$_3$

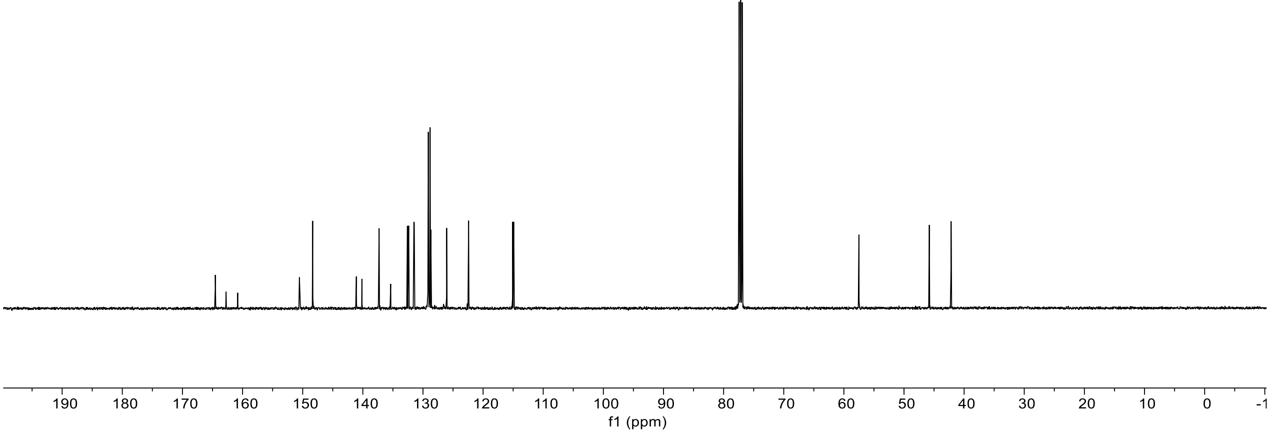

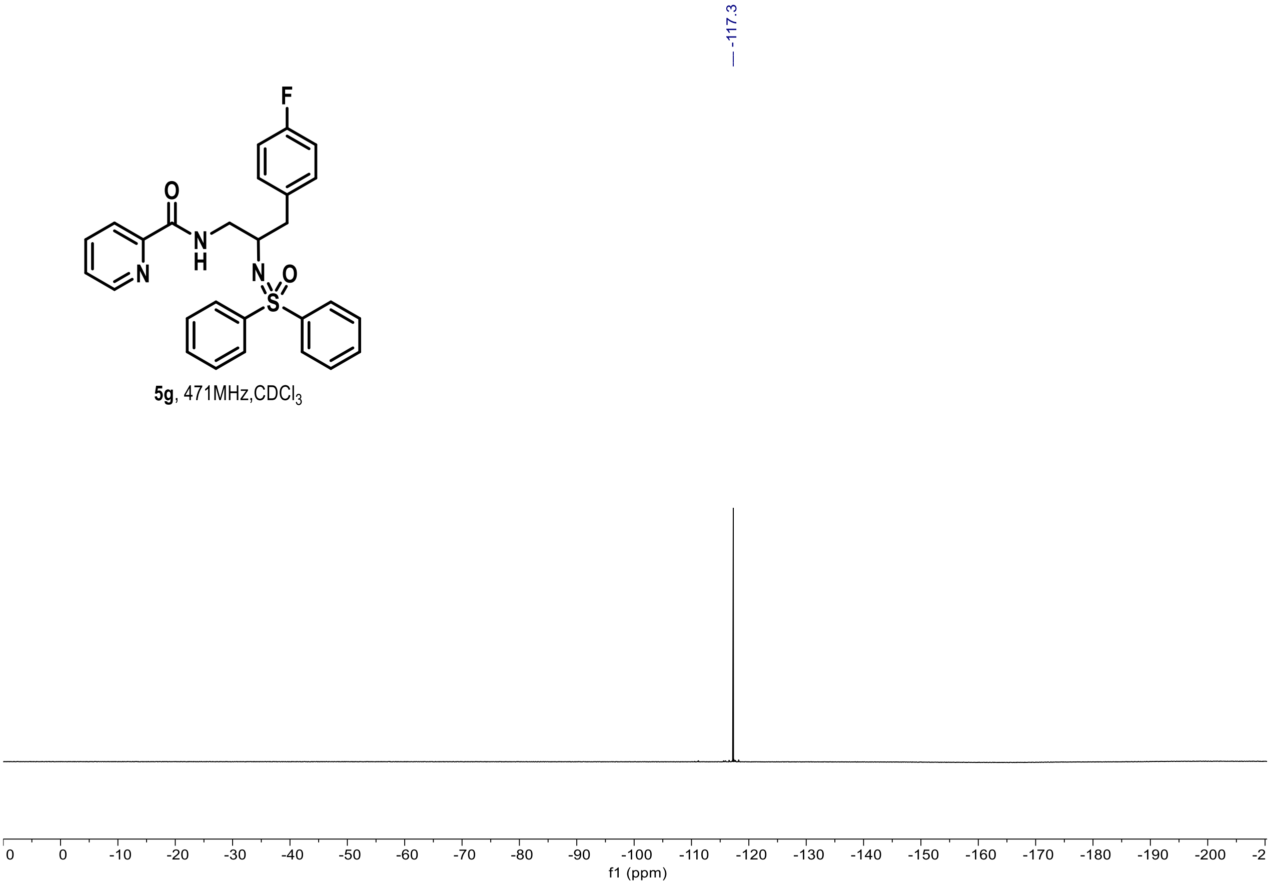

5g, 471MHz,$CDCl_3$

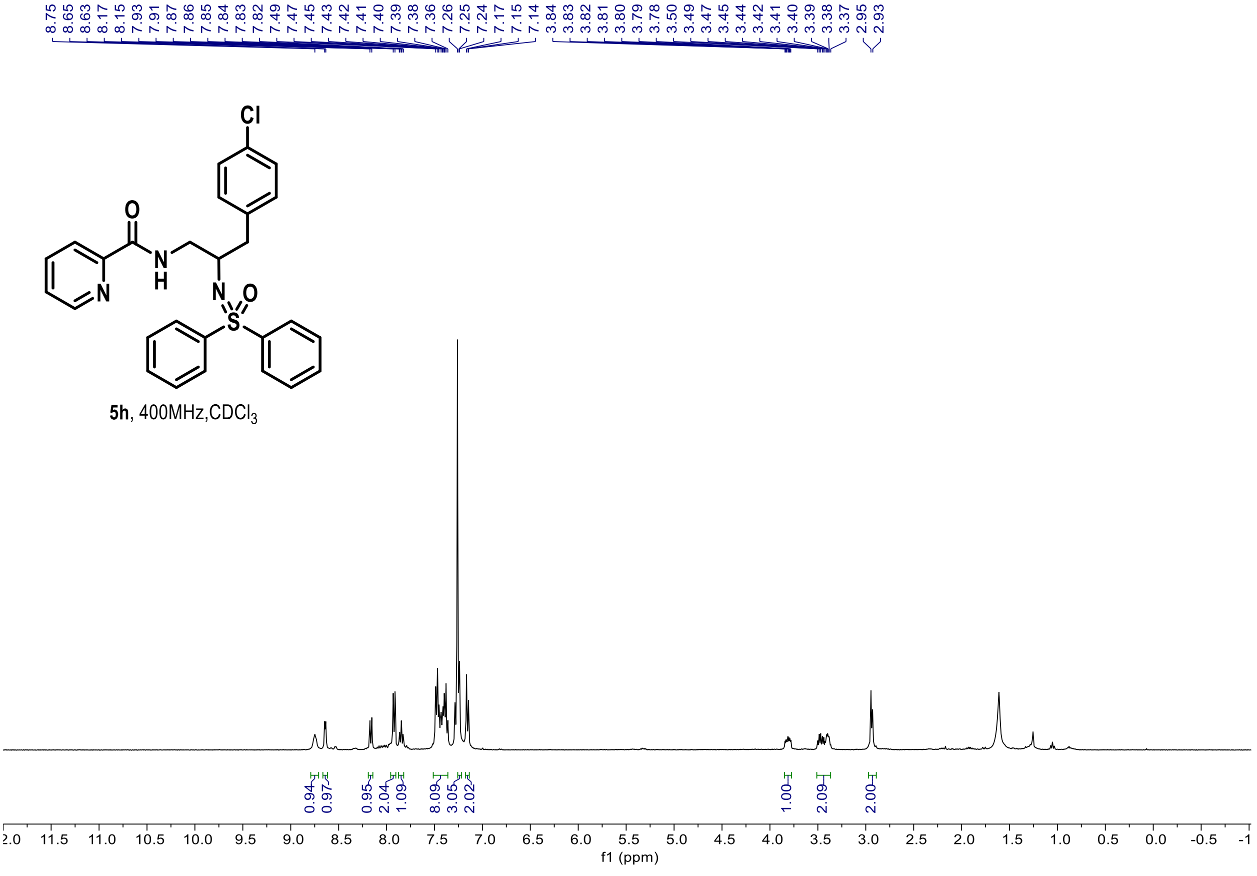

5h, 400MHz,$CDCl_3$

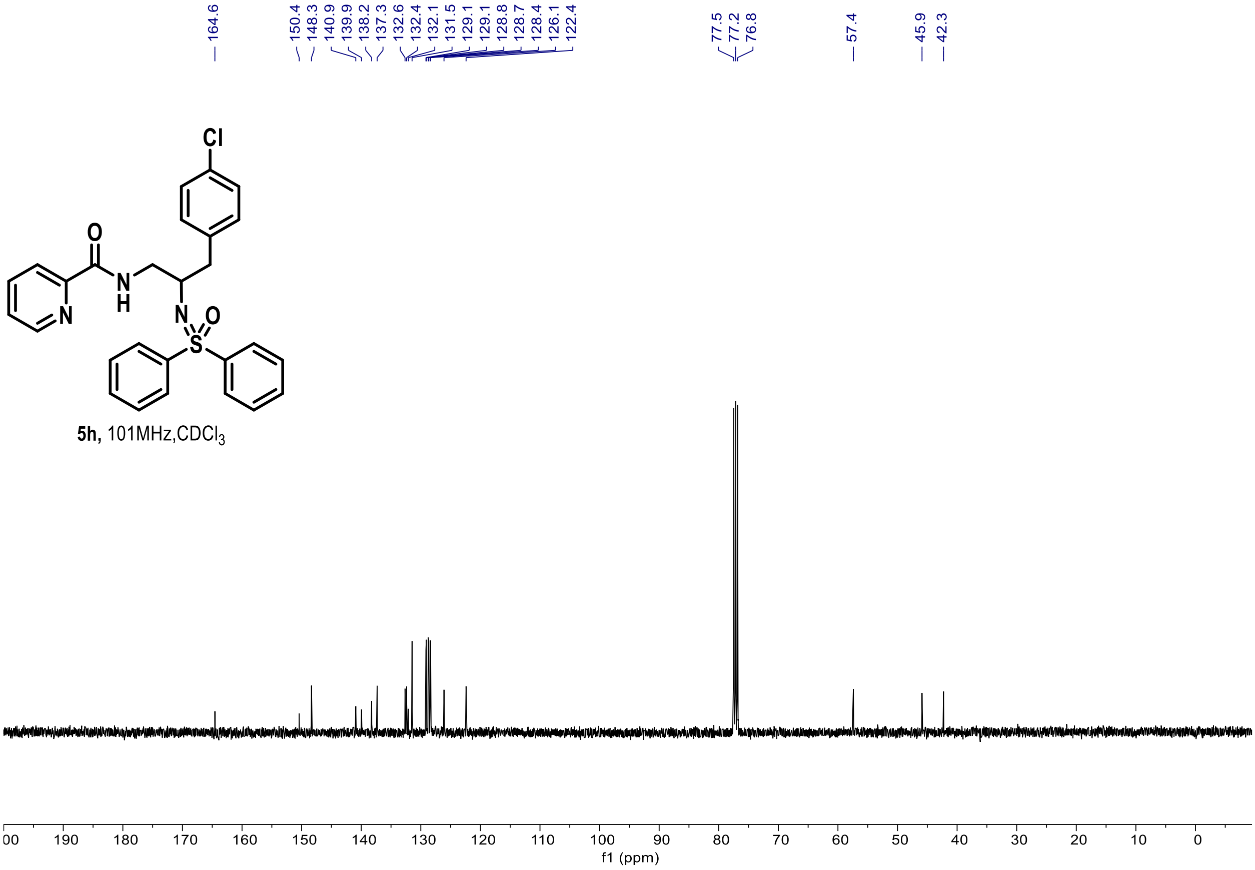

5h, 101MHz,$CDCl_3$

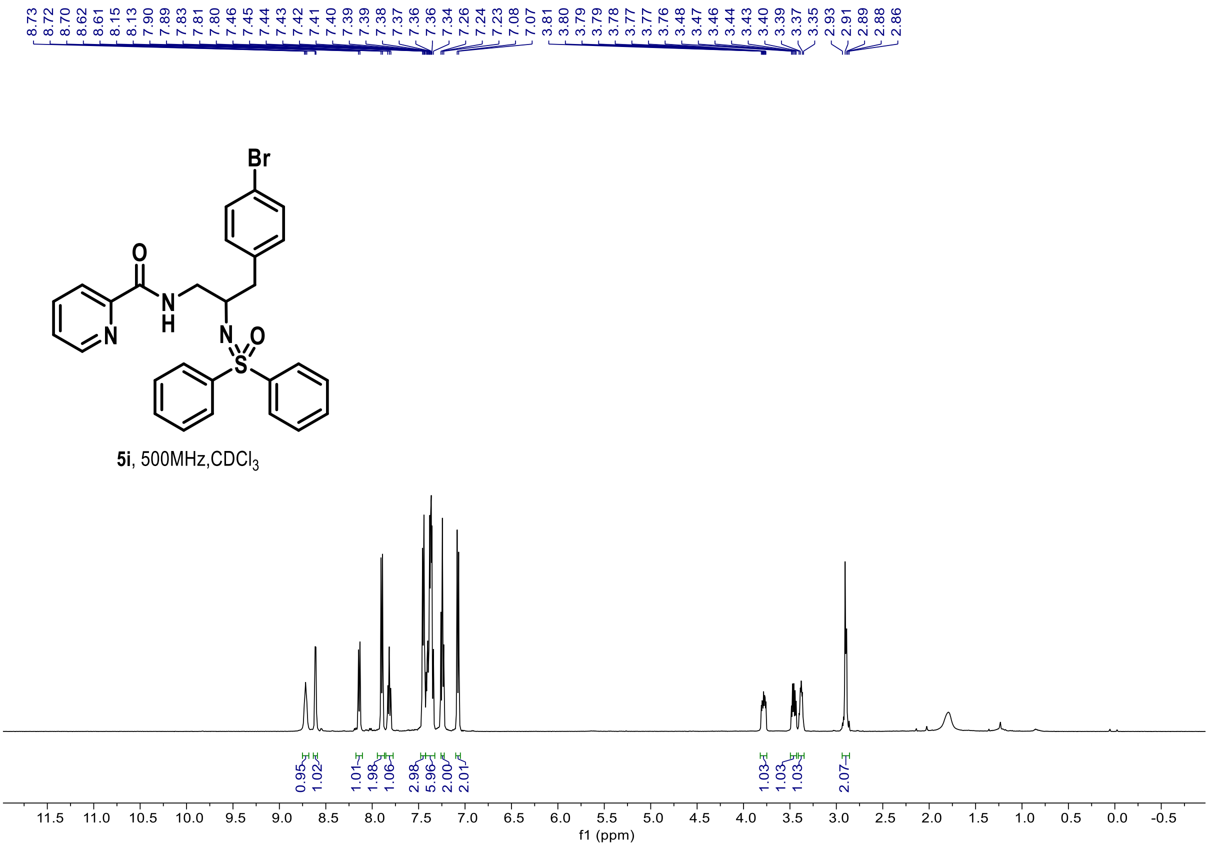

5i, 500MHz,$CDCl_3$

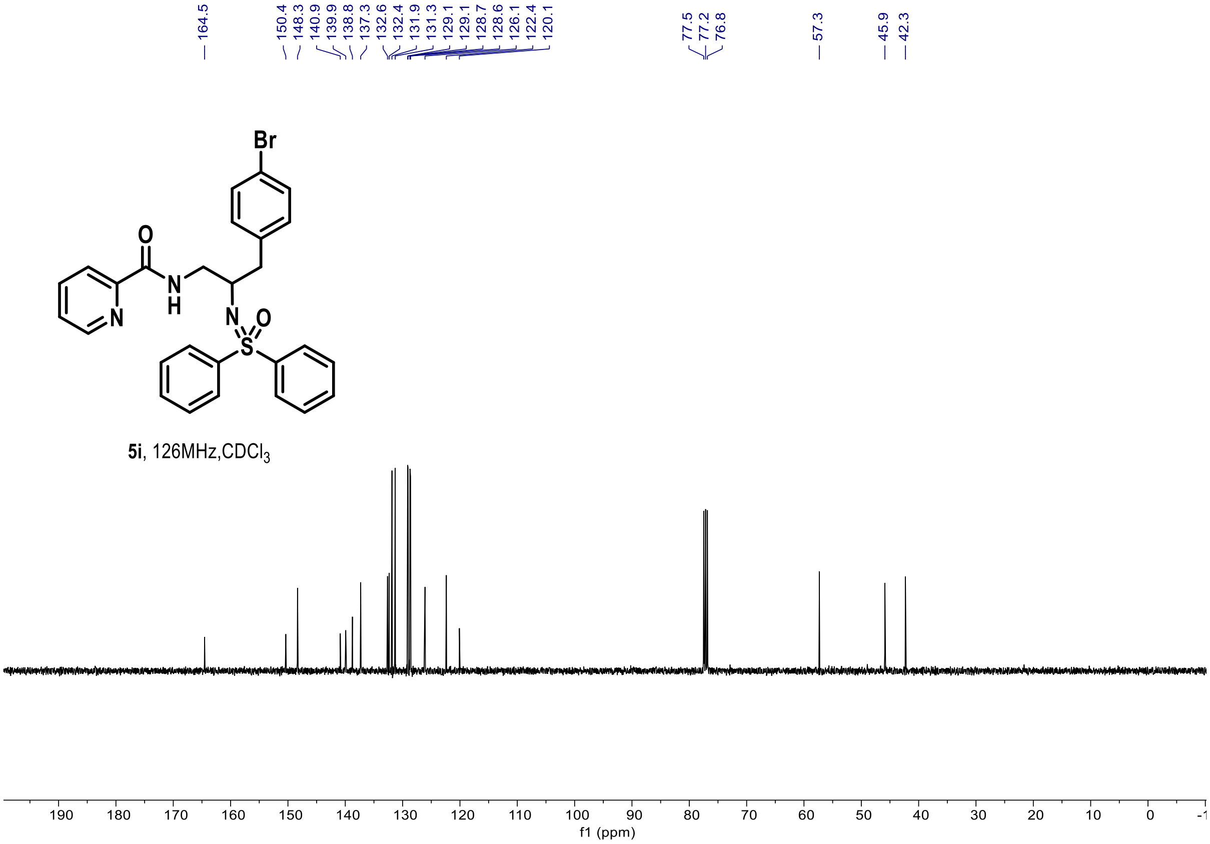
164.5
150.4
148.3
140.9
139.9
138.8
137.3
132.6
132.4
131.9
131.3
129.1
129.1
128.7
128.6
126.1
122.4
120.1
77.5
77.2
76.8
57.3
45.9
42.3
Br
O
N
H
N
N
O
S
5i, 126MHz,CDCl$_3$
f1 (ppm)

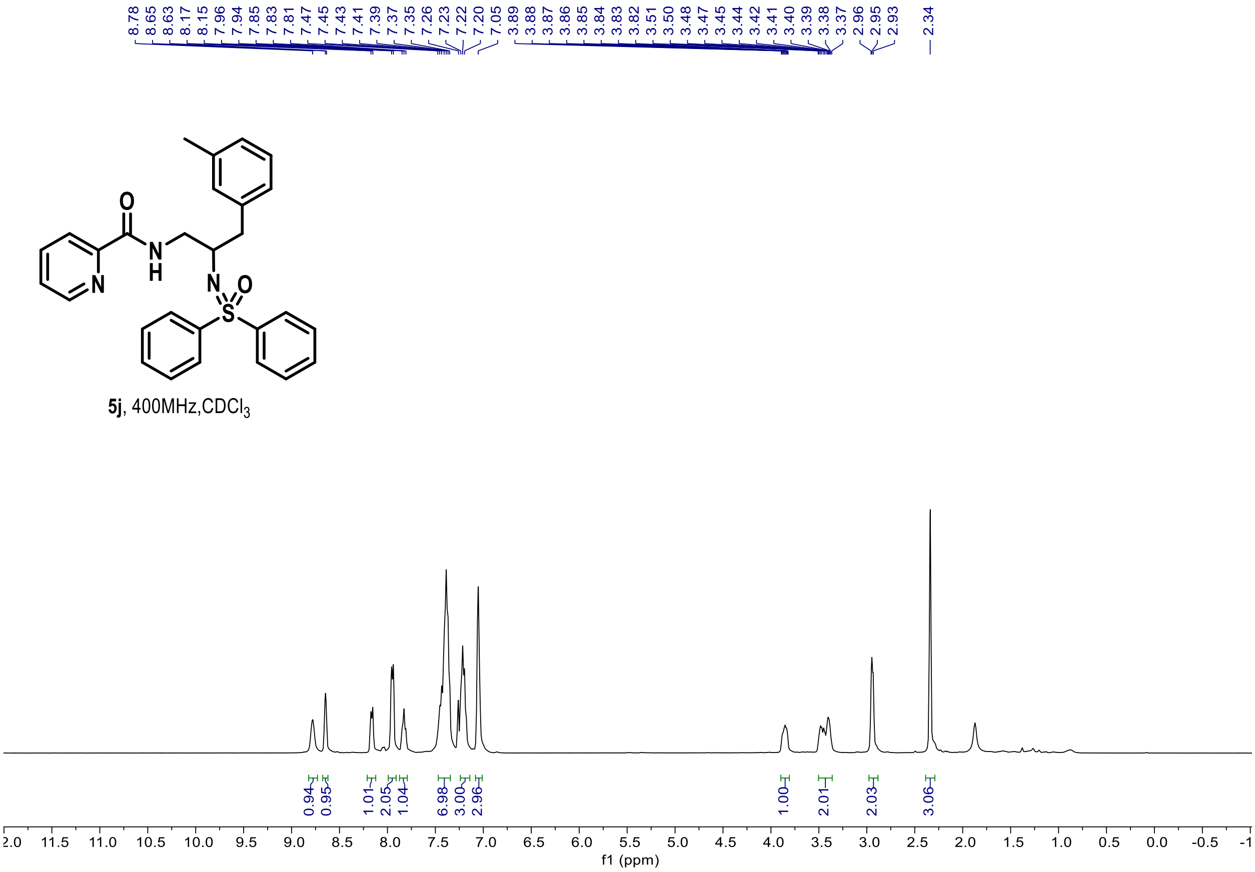
8.78
8.65
8.63
8.17
8.15
7.96
7.94
7.85
7.83
7.81
7.47
7.45
7.43
7.41
7.39
7.37
7.35
7.26
7.23
7.22
7.20
7.05
3.89
3.88
3.87
3.86
3.85
3.84
3.84
3.83
3.82
3.51
3.50
3.48
3.47
3.45
3.44
3.42
3.41
3.40
3.39
3.38
3.37
2.96
2.95
2.93
2.34
O
N
H
N
N
O
S
5j, 400MHz,CDCl$_3$
0.94
0.95
1.01
2.05
1.04
6.98
3.00
2.96
1.00
2.01
2.03
3.06
f1 (ppm)

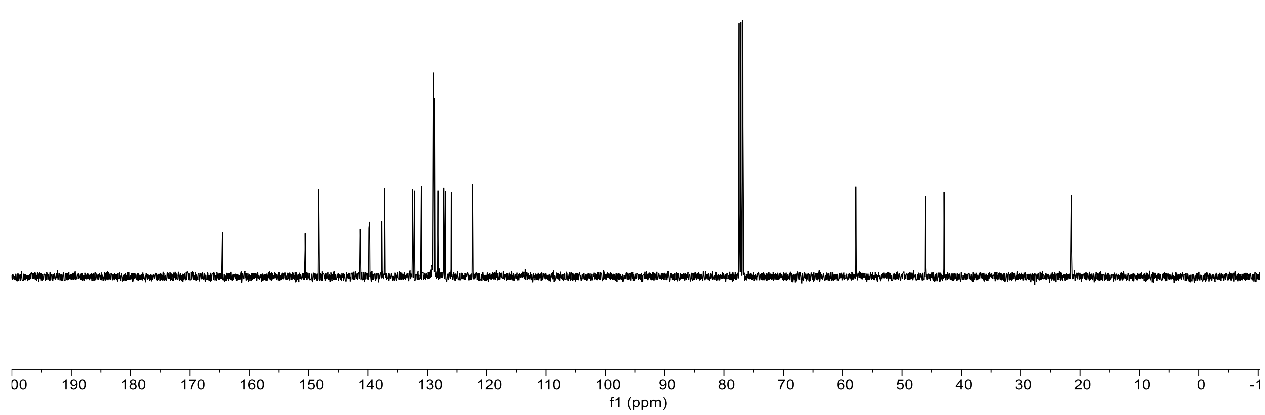

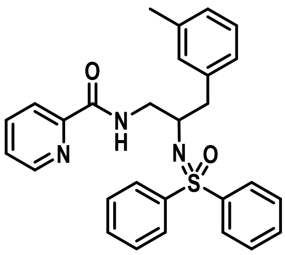

5j, 101MHz,$CDCl_3$

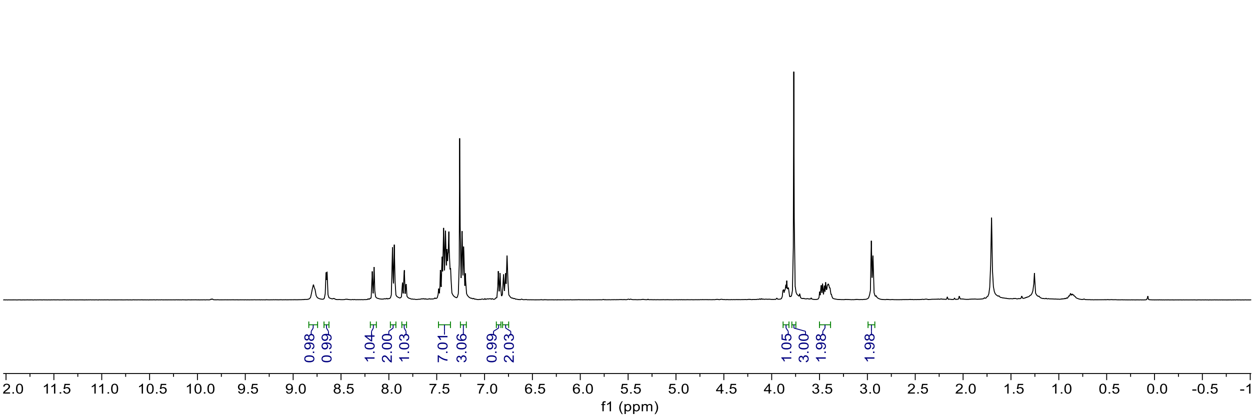

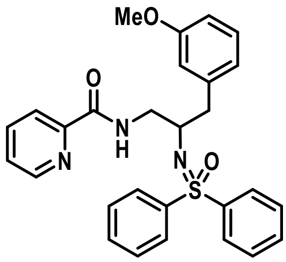

5k, 400MHz,$CDCl_3$

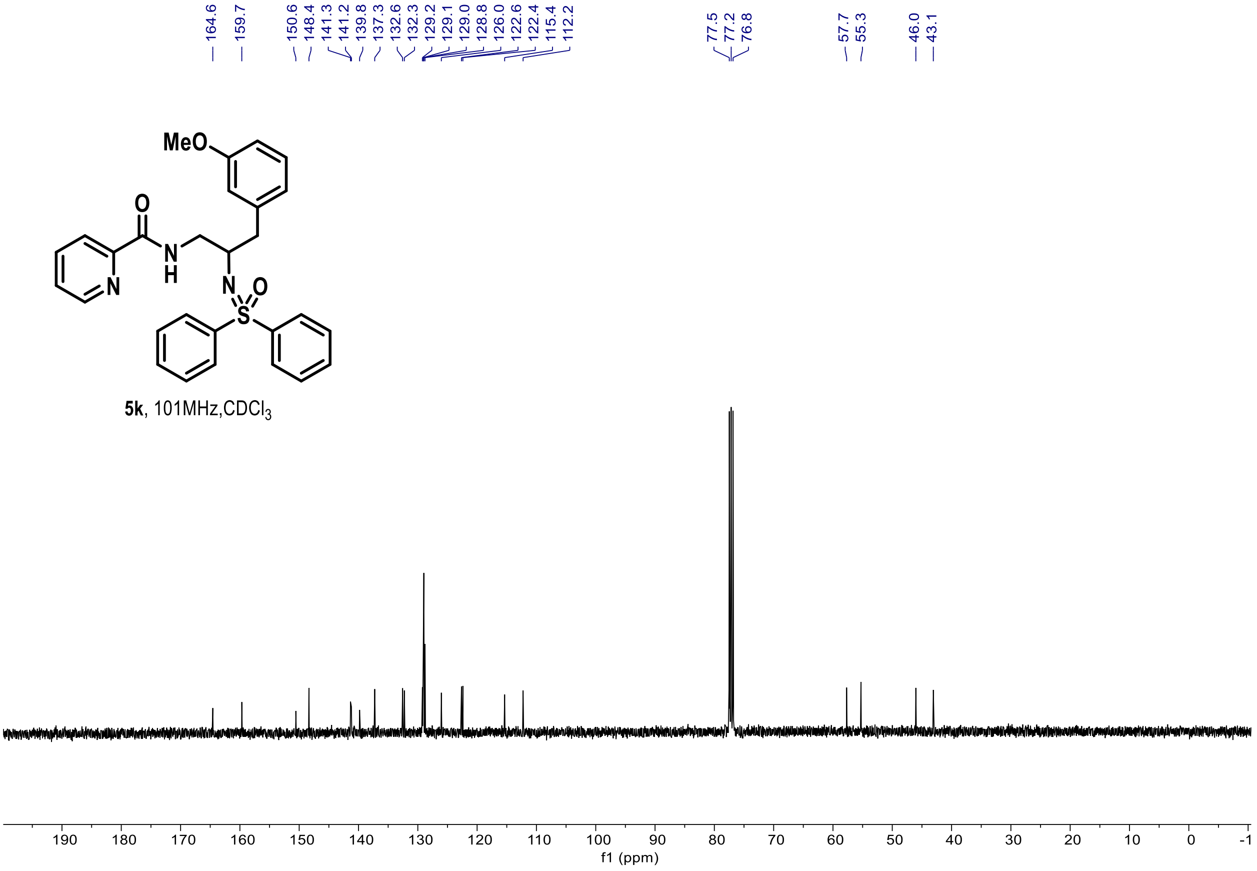

5k, 101MHz,$CDCl_3$

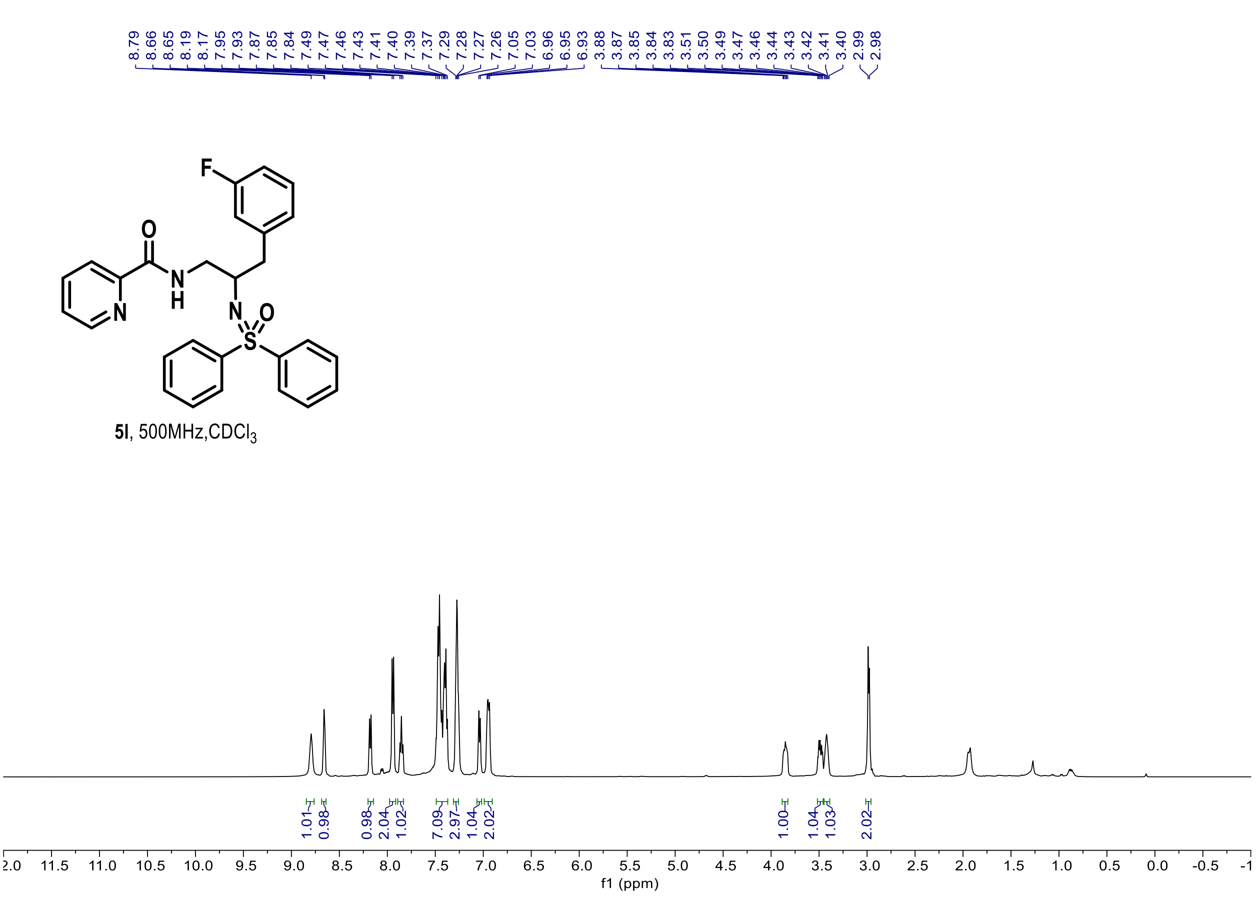

5l, 500MHz,$CDCl_3$

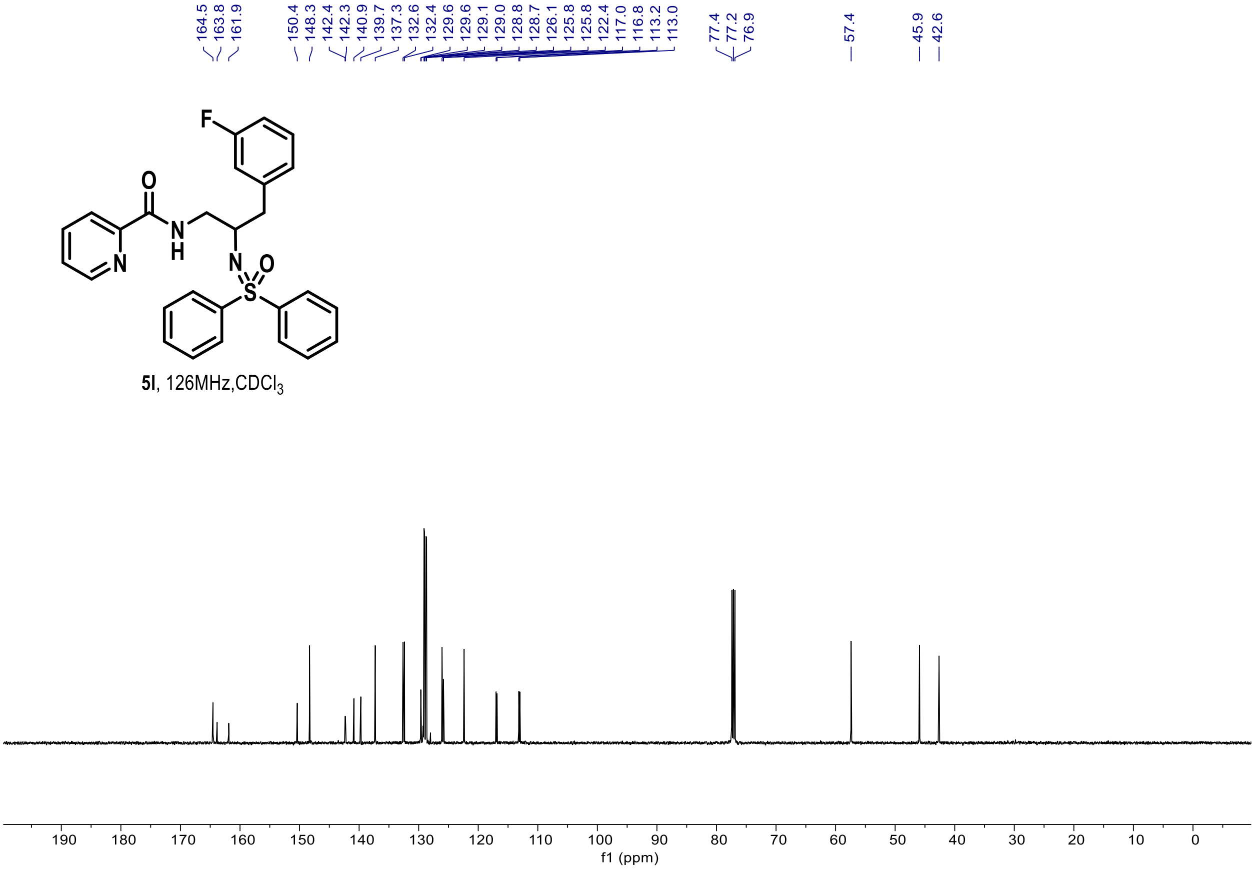
164.5
163.8
161.9
150.4
148.3
142.4
142.3
140.9
139.7
137.3
132.6
132.4
129.6
129.6
129.1
129.0
128.8
128.7
126.1
125.8
125.8
122.4
117.0
116.8
113.2
113.0
77.4
77.2
76.9
57.4
45.9
42.6
F
O
N
H
N
N
O
S
5l, 126MHz,CDCl3
190 180 170 160 150 140 130 120 110 100 90 80 70 60 50 40 30 20 10 0
f1 (ppm)

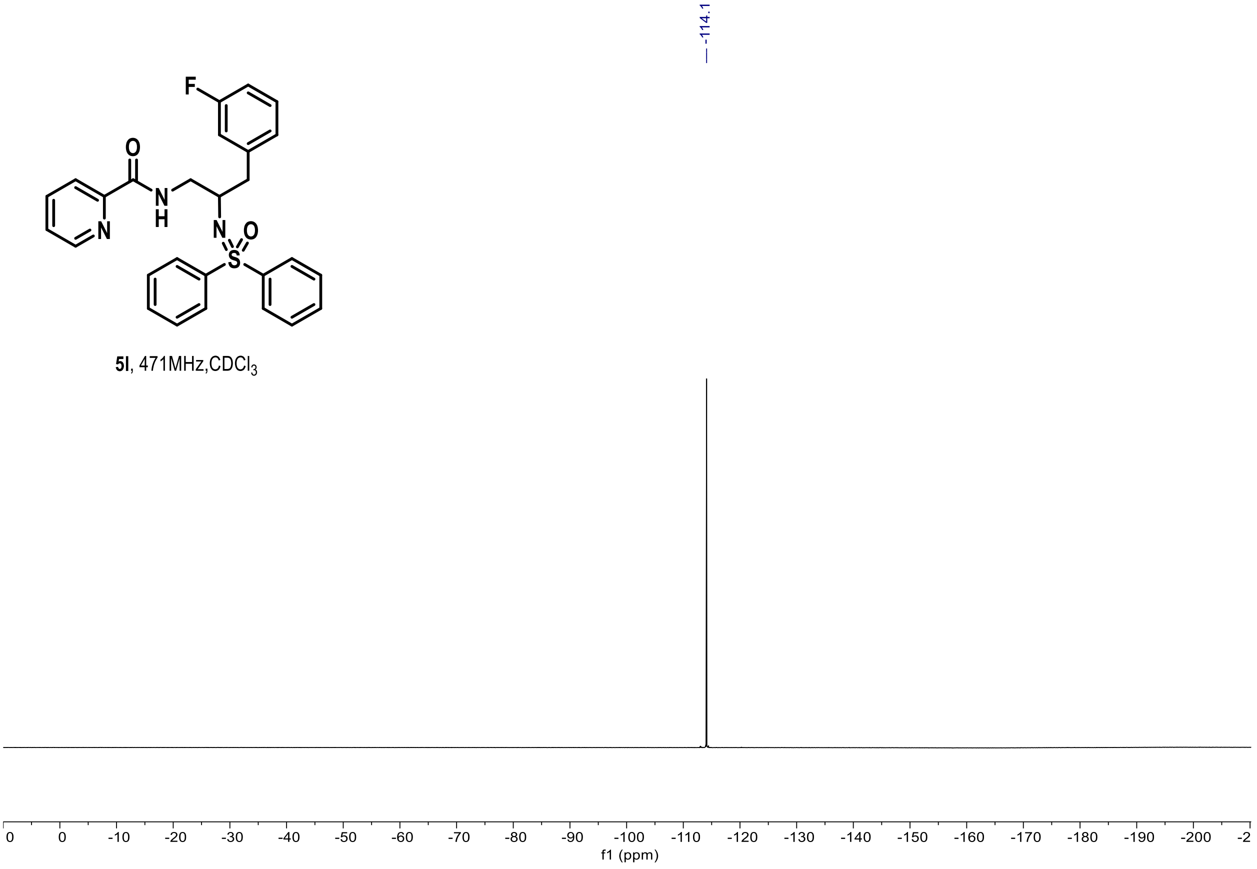
-114.1
F
O
N
H
N
N
O
S
5l, 471MHz,CDCl3
0 0 -10 -20 -30 -40 -50 -60 -70 -80 -90 -100 -110 -120 -130 -140 -150 -160 -170 -180 -190 -200 -2
f1 (ppm)

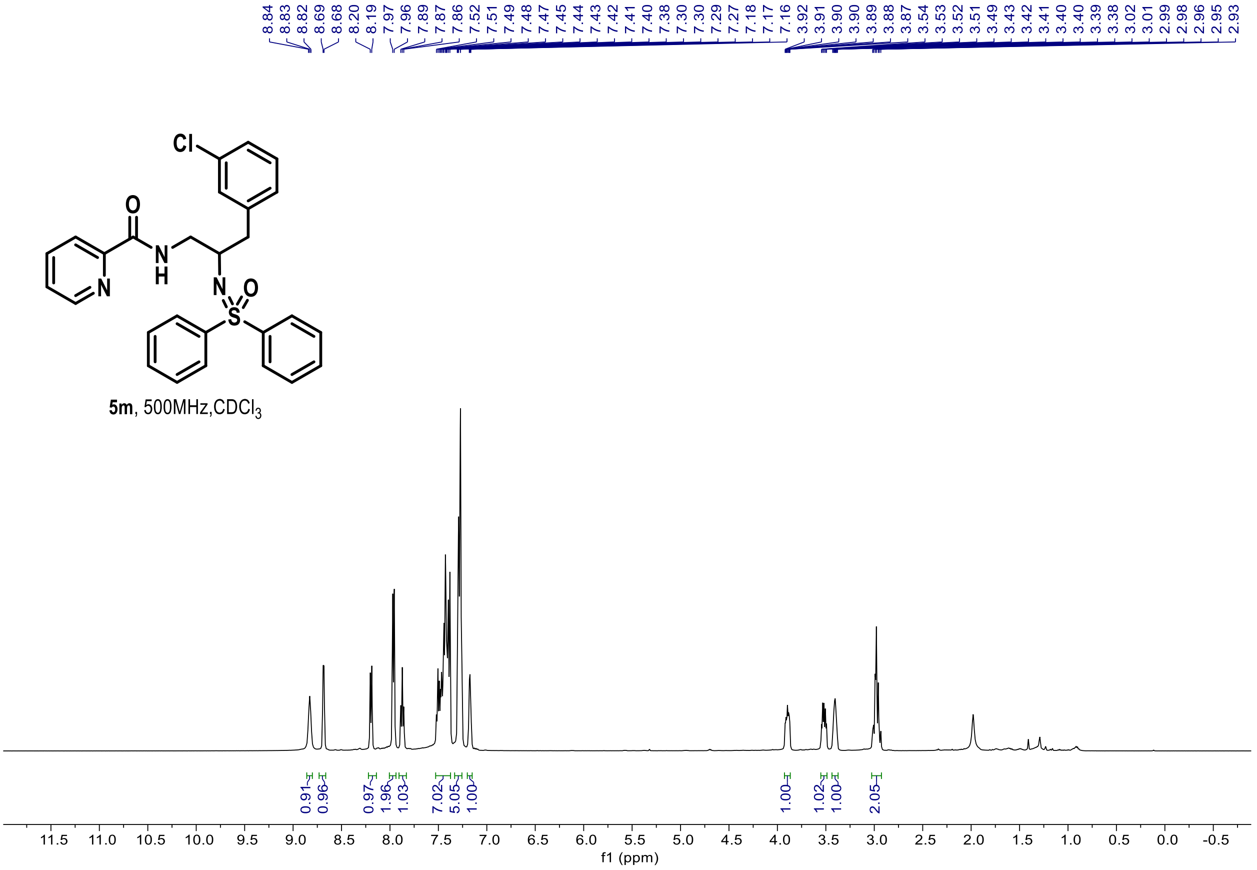
8.84 8.83 8.82 8.69 8.68 8.20 8.19 7.97 7.96 7.89 7.87 7.86 7.52 7.51 7.49 7.48 7.47 7.45 7.44 7.43 7.42 7.41 7.40 7.38 7.30 7.30 7.29 7.27 7.18 7.17 7.16 3.92 3.91 3.90 3.89 3.88 3.87 3.54 3.53 3.52 3.51 3.49 3.43 3.42 3.41 3.40 3.40 3.39 3.38 3.02 3.01 2.99 2.98 2.96 2.95 2.93
Cl
O
N
H
N
N
O
S
5m, 500MHz,CDCl3
0.91 0.96 0.97 1.96 1.03 7.02 5.05 1.00 1.00 1.02 1.00 2.05
11.5 11.0 10.5 10.0 9.5 9.0 8.5 8.0 7.5 7.0 6.5 6.0 5.5 5.0 4.5 4.0 3.5 3.0 2.5 2.0 1.5 1.0 0.5 0.0 -0.5
f1 (ppm)

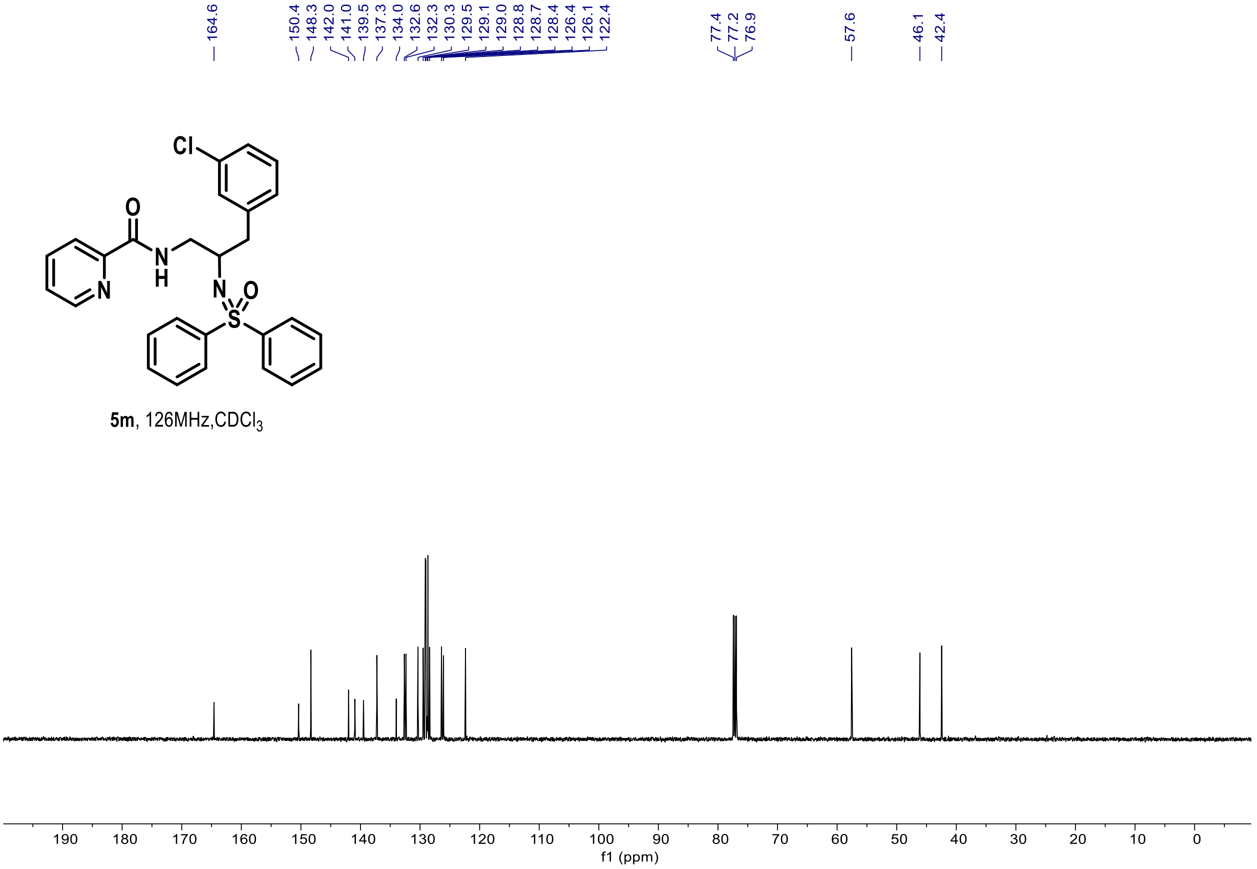
164.6 150.4 148.3 142.0 141.0 139.5 137.3 134.0 132.6 132.3 130.3 129.5 129.1 129.0 128.8 128.7 128.4 126.4 126.1 122.4 77.4 77.2 76.9 57.6 46.1 42.4
Cl
O
N
H
N
N
O
S
5m, 126MHz,CDCl3
190 180 170 160 150 140 130 120 110 100 90 80 70 60 50 40 30 20 10 0
f1 (ppm)

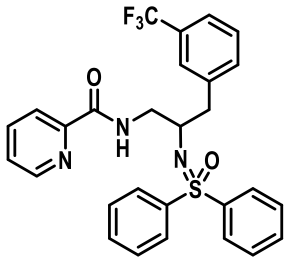

5n, 500MHz,$CDCl_3$

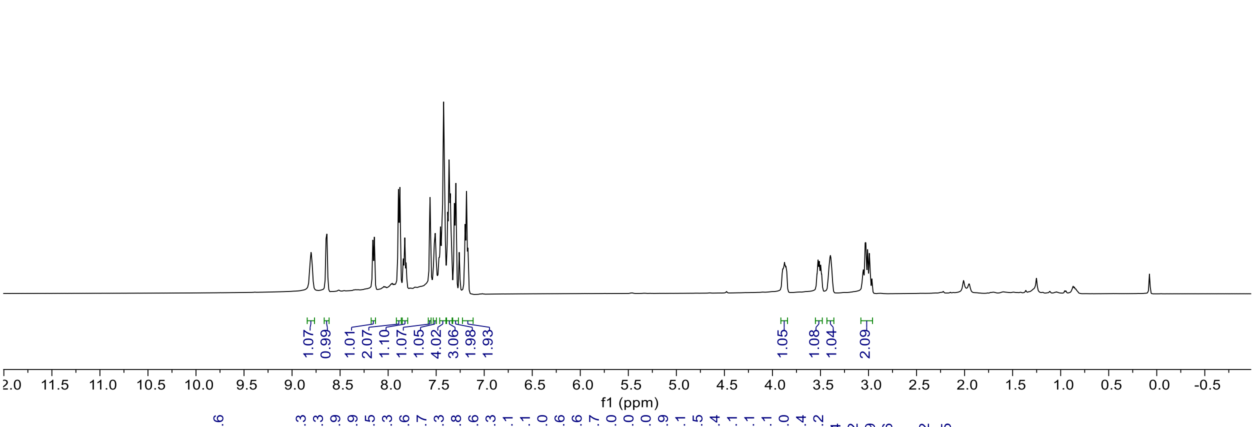

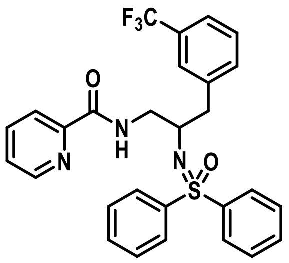

5n, 126MHz,$CDCl_3$

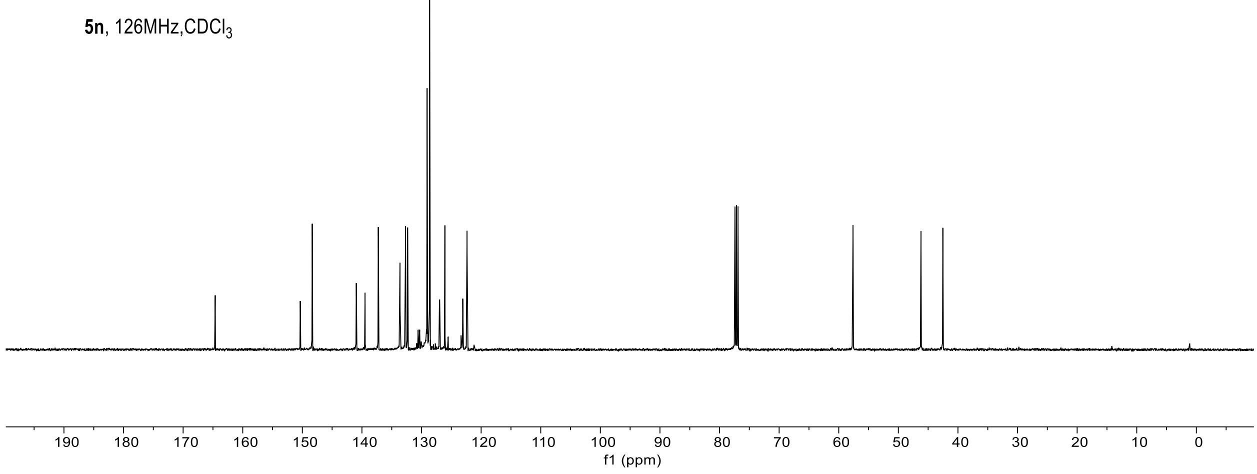

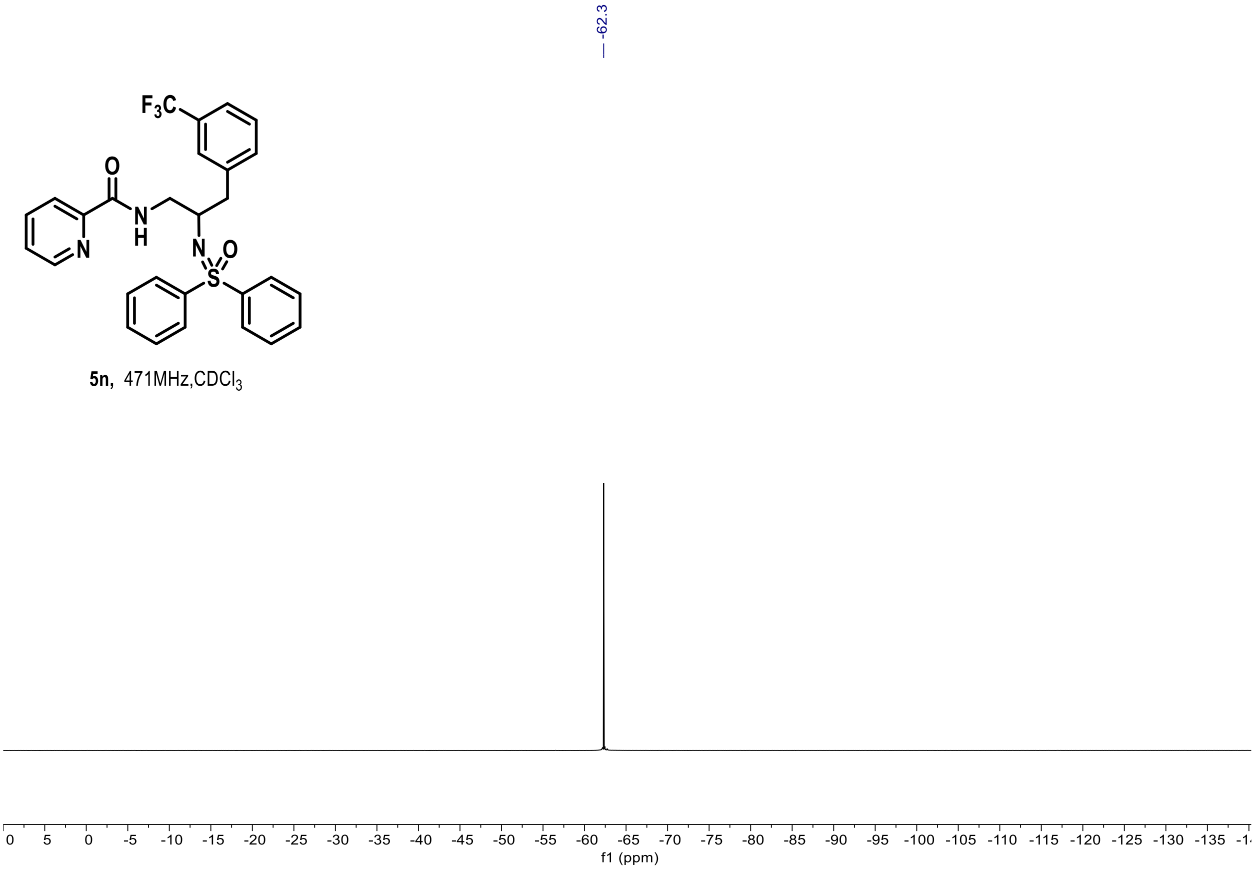

5n, 471MHz,$CDCl_3$

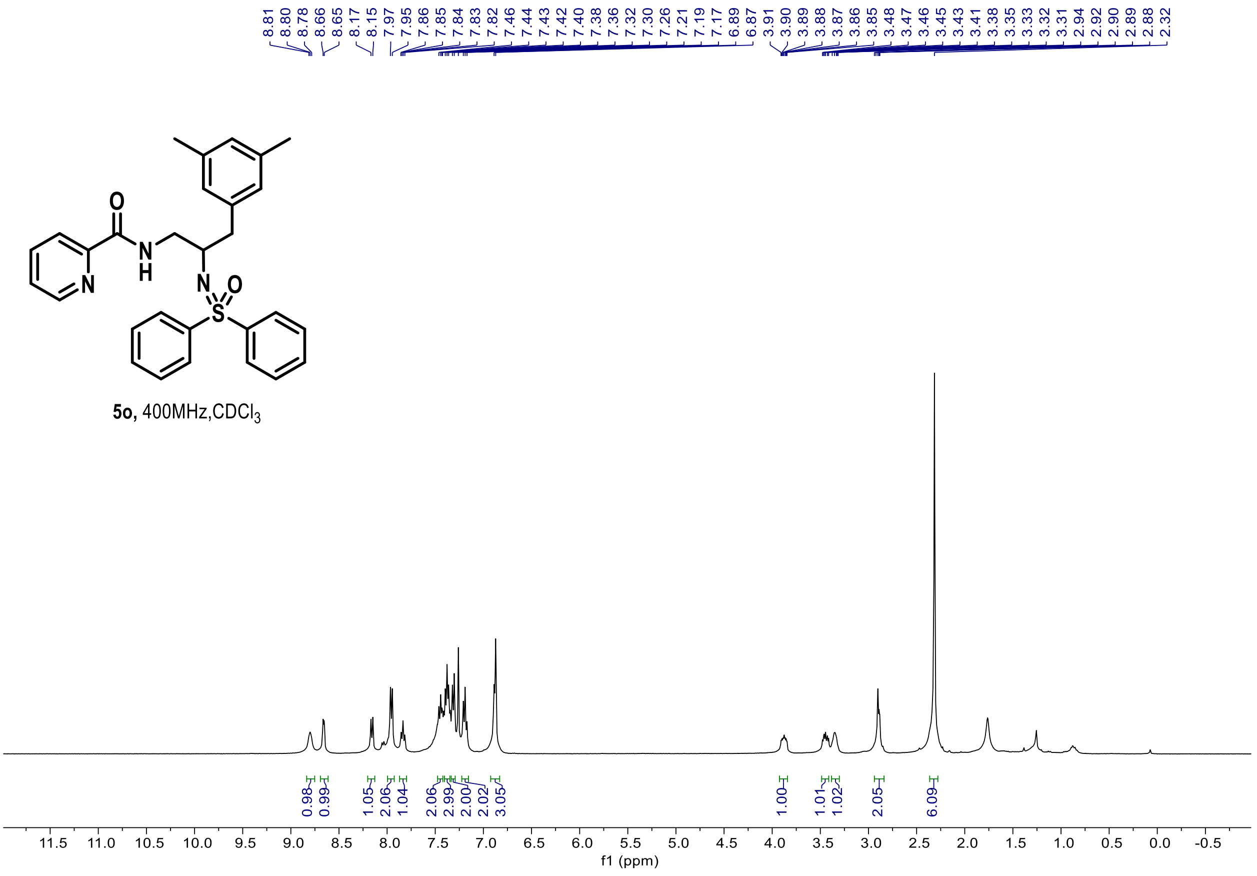

5o, 400MHz,$CDCl_3$

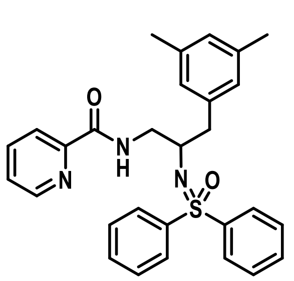

5o, 101MHz,CDCl$_3$

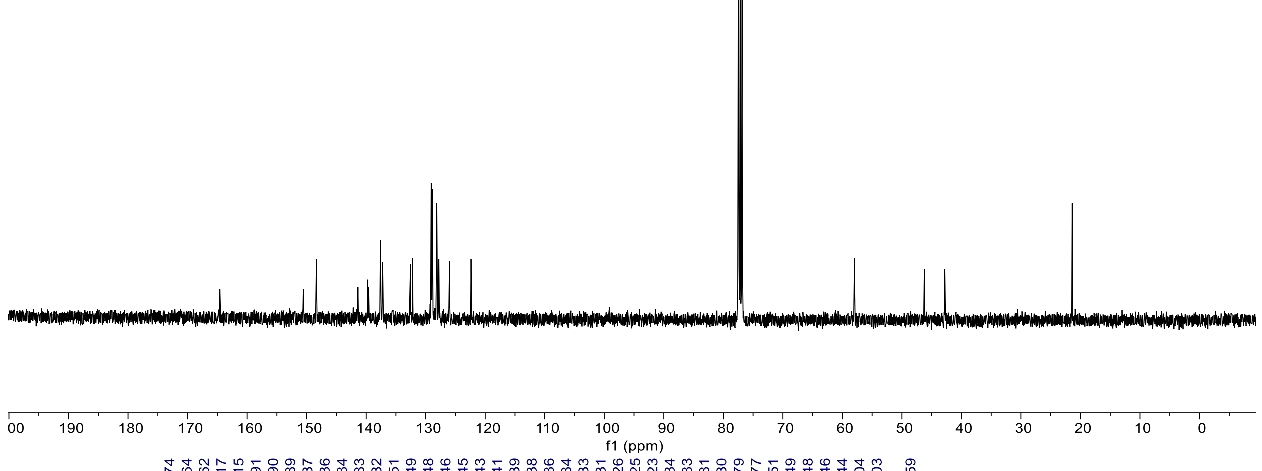

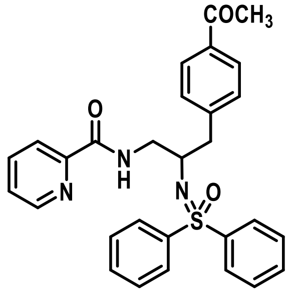

5p, 400MHz,CDCl$_3$

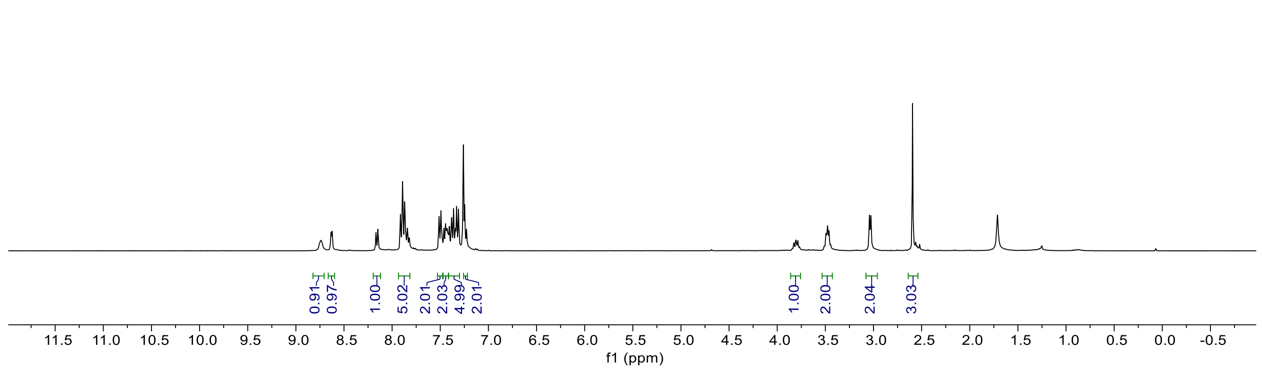

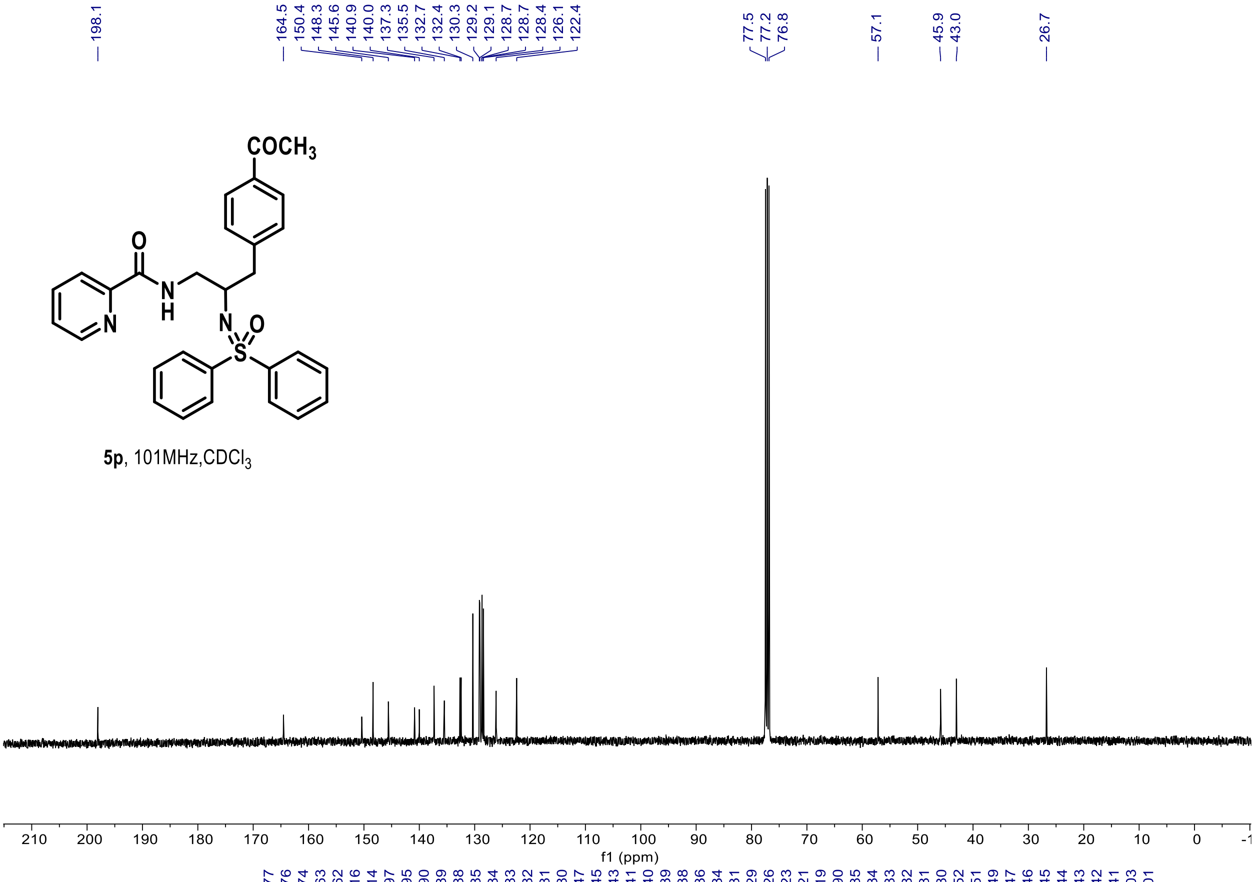
198.1
164.5
150.4
148.3
145.6
140.9
140.0
137.3
135.5
132.7
132.4
130.3
129.2
129.1
128.7
128.7
128.4
126.1
122.4
77.5
77.2
76.8
57.1
45.9
43.0
26.7
COCH3
O
N
H
N
N
O
S
5p, 101MHz,CDCl3
f1 (ppm)

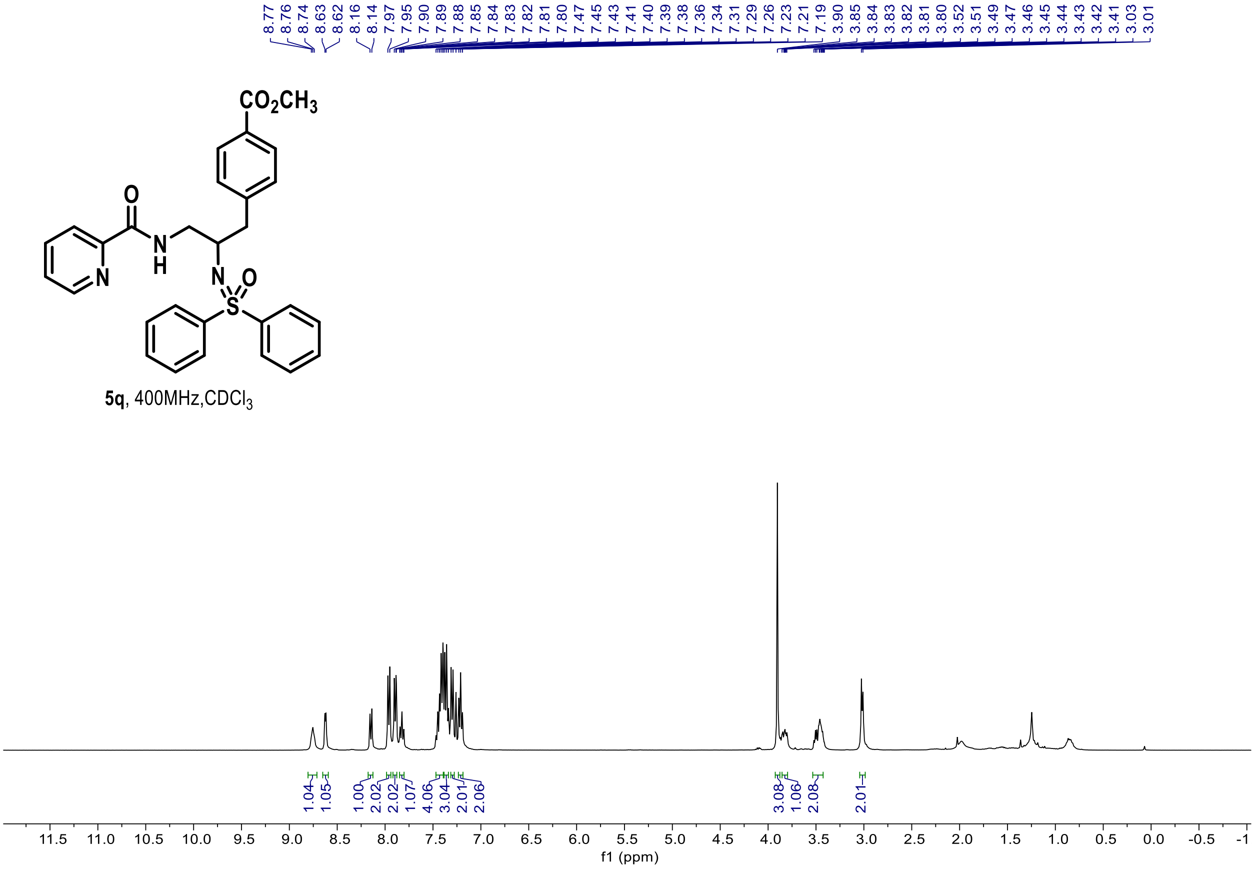
8.77
8.76
8.74
8.63
8.62
8.16
8.14
7.97
7.95
7.90
7.89
7.88
7.85
7.84
7.83
7.82
7.81
7.80
7.47
7.45
7.43
7.41
7.40
7.39
7.38
7.36
7.34
7.31
7.29
7.26
7.23
7.21
7.19
3.90
3.85
3.84
3.83
3.82
3.81
3.80
3.52
3.51
3.49
3.47
3.46
3.45
3.44
3.43
3.42
3.41
3.03
3.01
CO2CH3
O
N
H
N
N
O
S
5q, 400MHz,CDCl3
1.04
1.05
1.00
2.02
2.02
1.07
4.06
3.04
2.01
2.06
3.08
1.06
2.08
2.01
f1 (ppm)

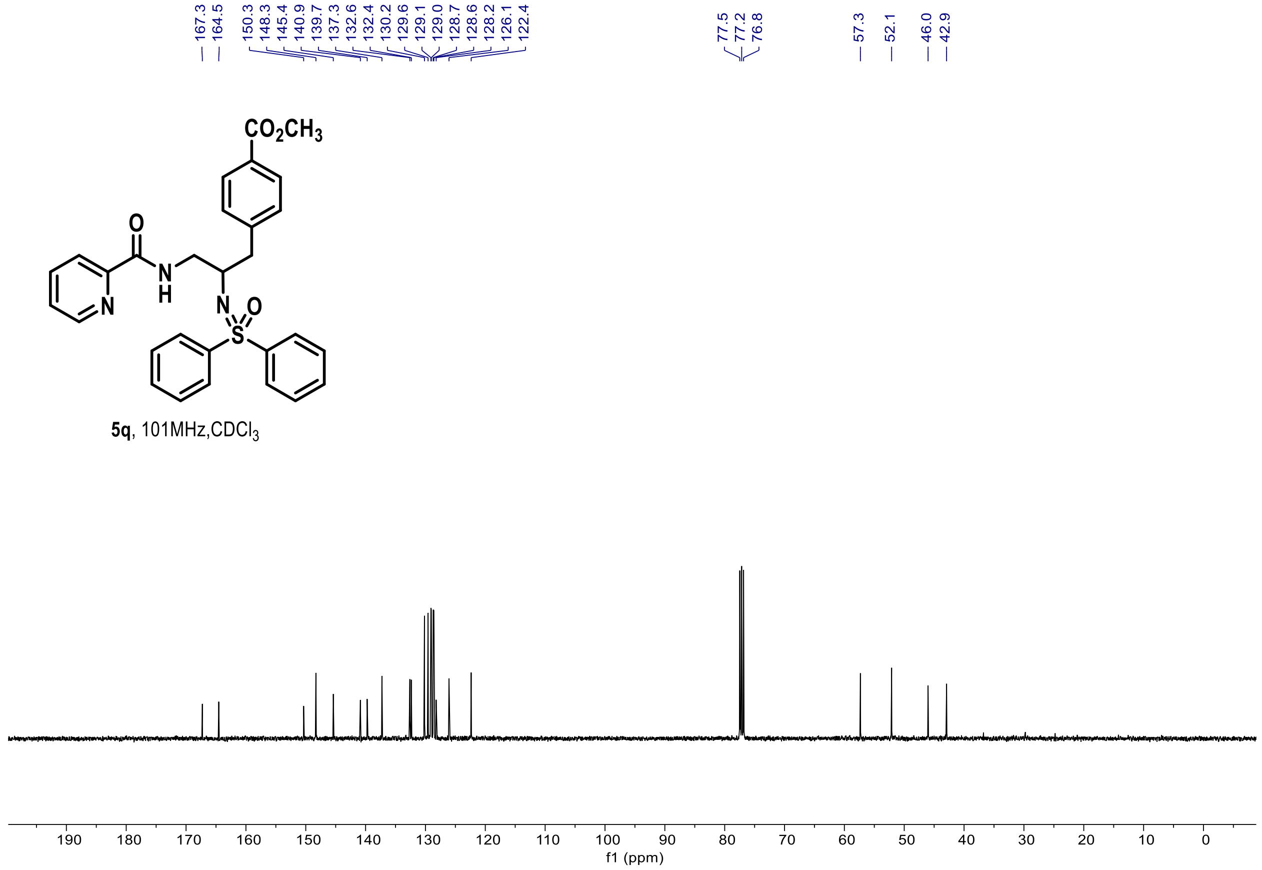
167.3
164.5
150.3 148.3 145.4 140.9 139.7 137.3 132.6 132.4 130.2 129.6 129.1 129.0 128.7 128.6 128.2 126.1 122.4
77.5 77.2 76.8
57.3
52.1
46.0
42.9
CO_2CH_3
O
N
H
N
N
S
O
5q, 101MHz,$CDCl_3$
f1 (ppm)

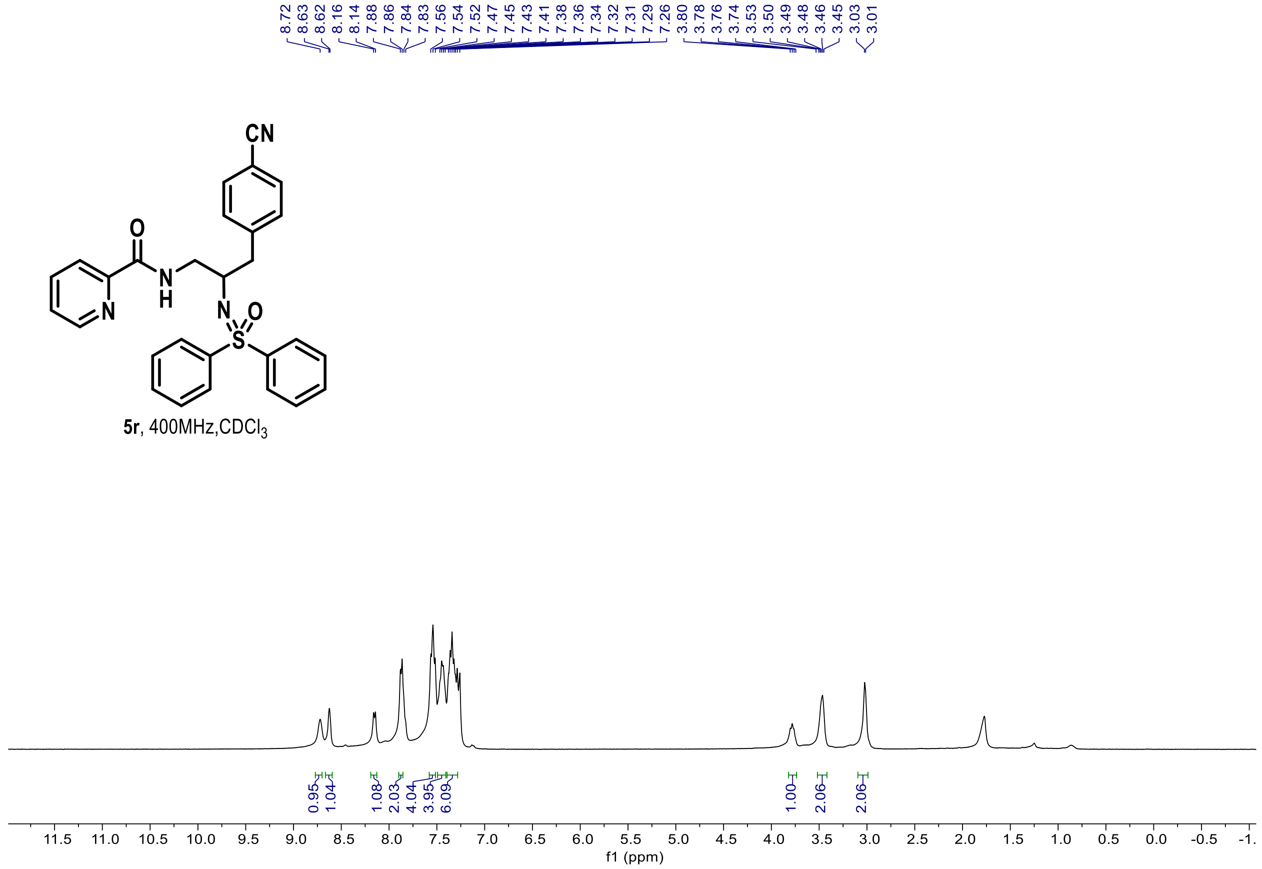
8.72 8.63 8.62 8.16 8.14 7.88 7.86 7.84 7.83 7.56 7.54 7.52 7.47 7.45 7.43 7.41 7.38 7.36 7.34 7.32 7.31 7.29 7.26 3.80 3.78 3.76 3.74 3.53 3.50 3.49 3.48 3.46 3.45 3.03 3.01
CN
O
N
H
N
N
S
O
5r, 400MHz,$CDCl_3$
0.95 1.04 1.08 2.03 4.04 3.95 6.09 1.00 2.06 2.06
f1 (ppm)

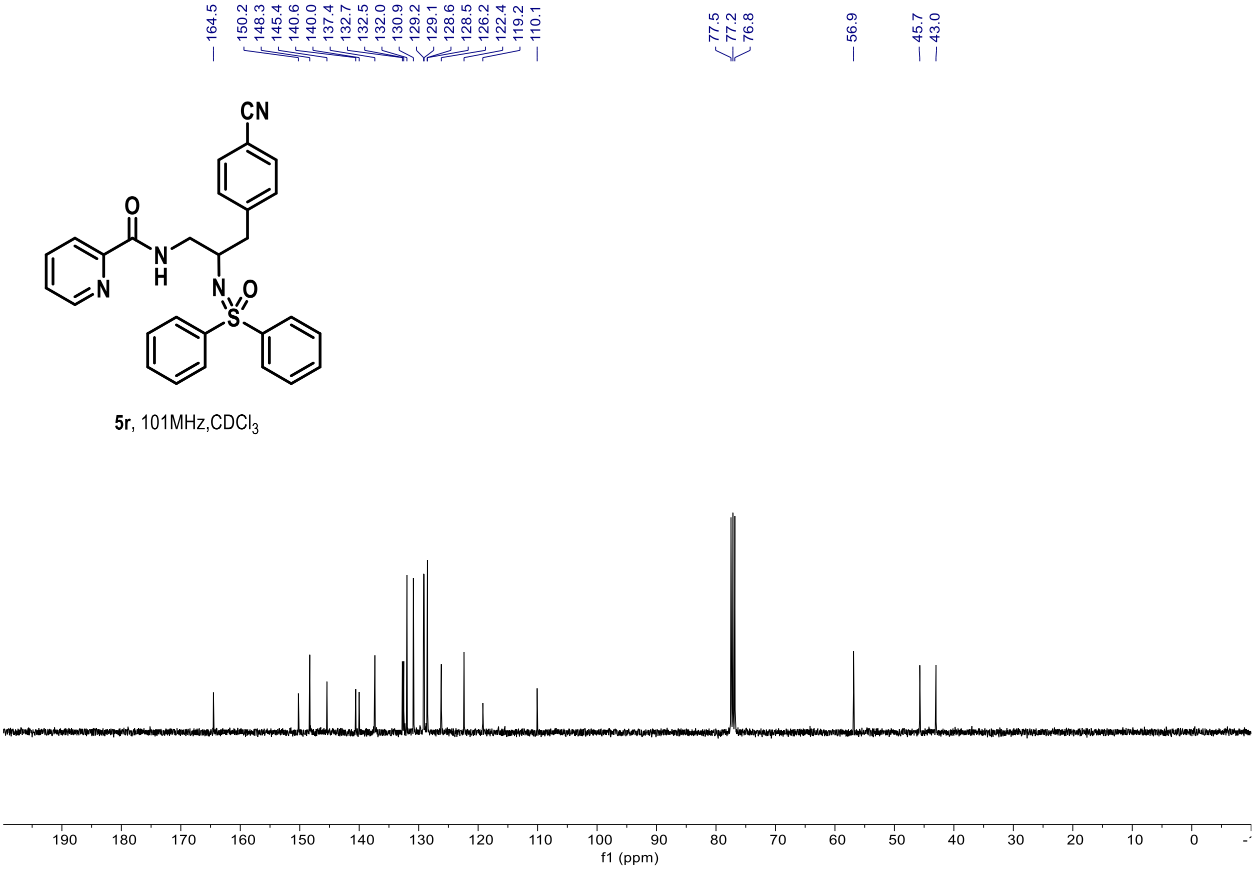
164.5 150.2 148.3 145.4 140.6 140.0 137.4 132.7 132.5 132.0 130.9 129.2 129.1 128.6 128.5 126.2 122.4 119.2 110.1
77.5 77.2 76.8
56.9
45.7 43.0
CN
O
N
H
N
N
O
S
5r, 101MHz,CDCl3
190 180 170 160 150 140 130 120 110 100 90 80 70 60 50 40 30 20 10 0
f1 (ppm)

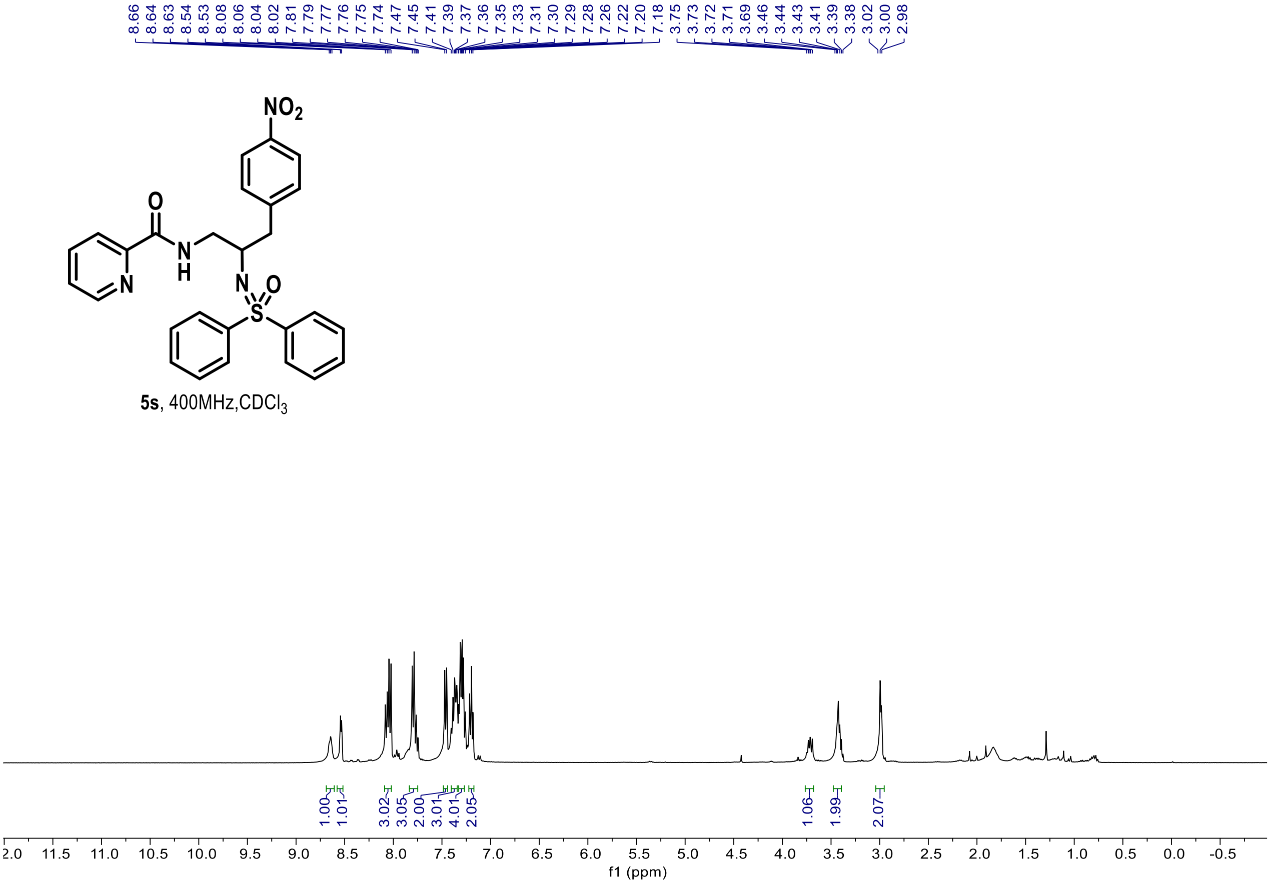
8.66 8.64 8.63 8.54 8.53 8.08 8.06 8.04 8.02 7.81 7.79 7.77 7.76 7.75 7.74 7.47 7.45 7.41 7.39 7.37 7.36 7.35 7.33 7.31 7.30 7.29 7.28 7.26 7.22 7.20 7.18 3.75 3.73 3.72 3.71 3.69 3.46 3.44 3.43 3.41 3.39 3.38 3.02 3.00 2.98
NO2
O
N
H
N
N
O
S
5s, 400MHz,CDCl3
1.00 1.01 3.02 3.05 2.00 3.01 4.01 2.05 1.06 1.99 2.07
2.0 11.5 11.0 10.5 10.0 9.5 9.0 8.5 8.0 7.5 7.0 6.5 6.0 5.5 5.0 4.5 4.0 3.5 3.0 2.5 2.0 1.5 1.0 0.5 0.0 -0.5
f1 (ppm)

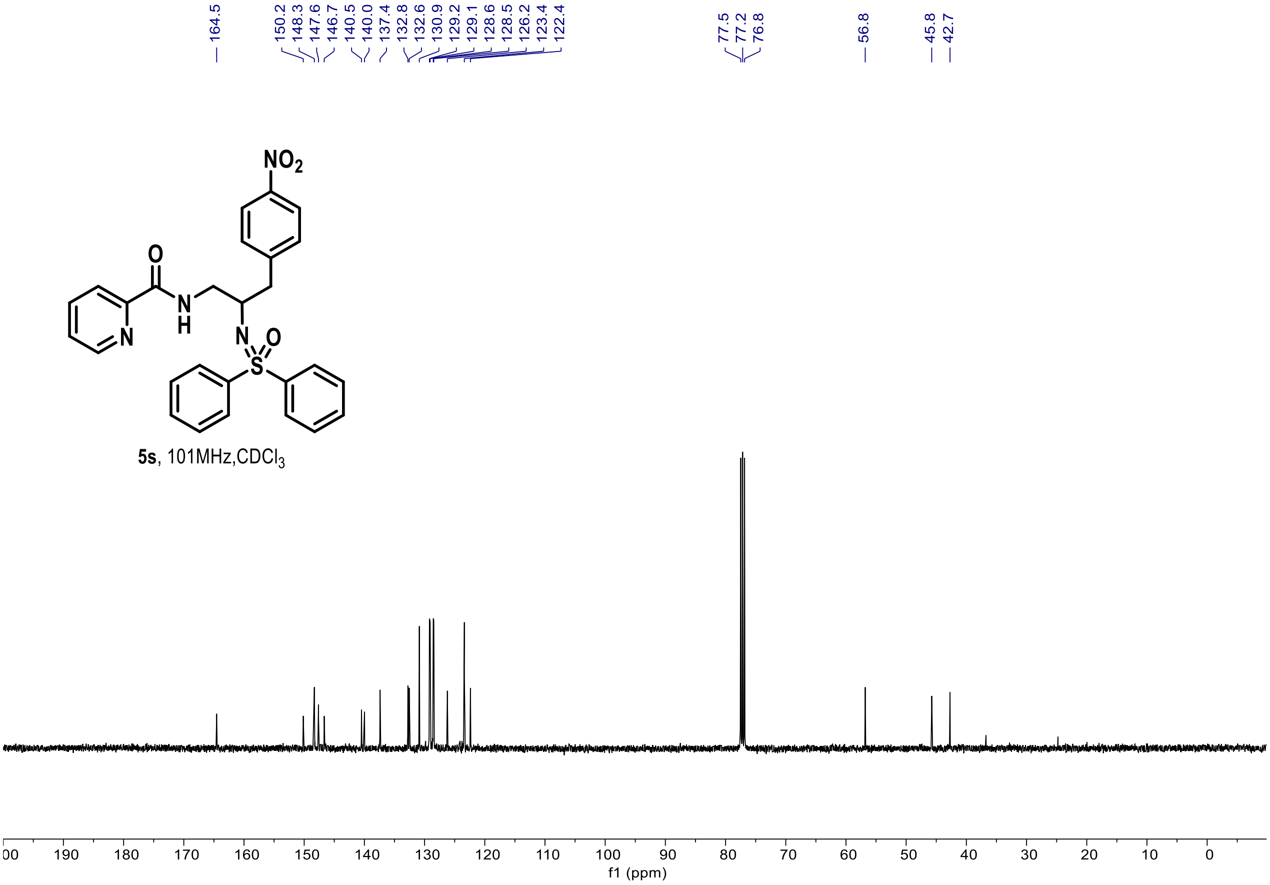

5s, 101MHz,$CDCl_3$

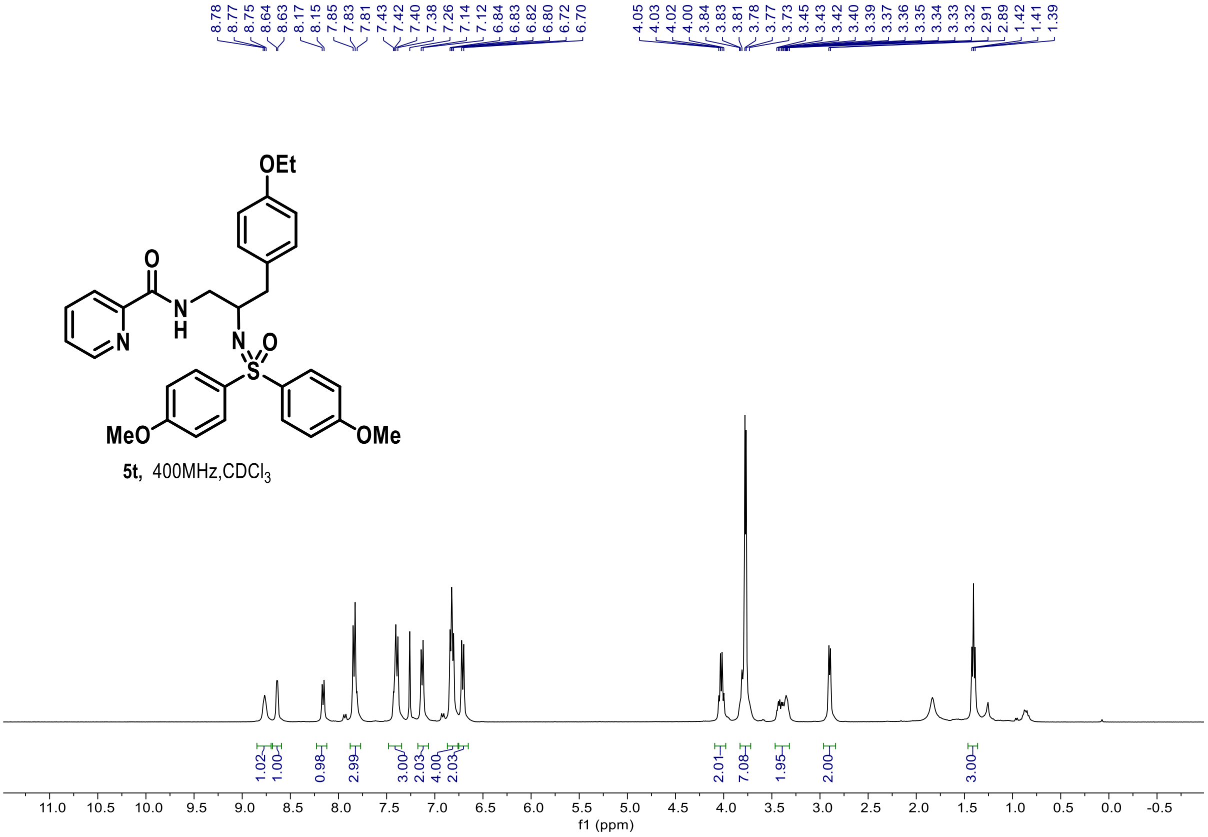

5t, 400MHz,$CDCl_3$

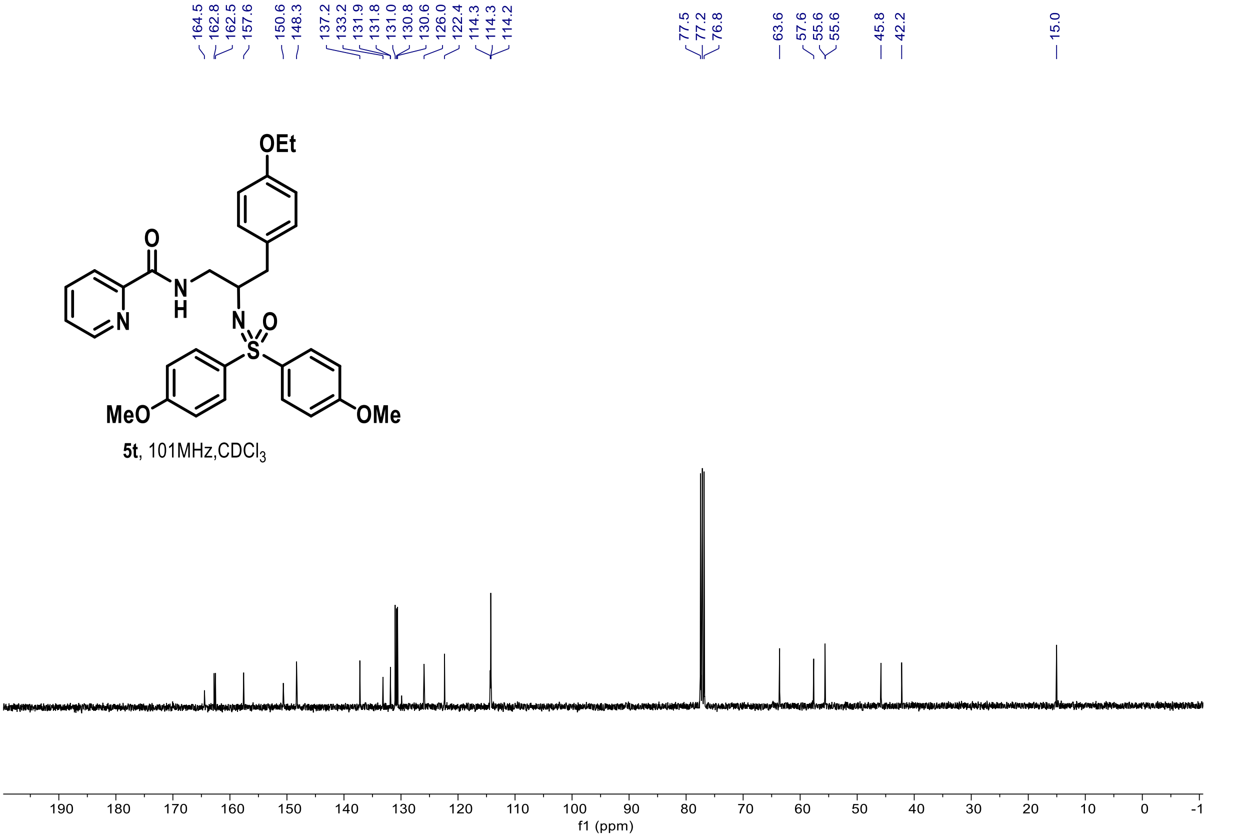
164.5
162.8
162.5
157.6
150.6
148.3
137.2
133.2
131.9
131.8
131.0
130.8
130.6
126.0
122.4
114.3
114.3
114.2
77.5
77.2
76.8
63.6
57.6
55.6
55.6
45.8
42.2
15.0
OEt
O
N
H
N
N
S
O
MeO
OMe
5t, 101MHz,CDCl3
f1 (ppm)

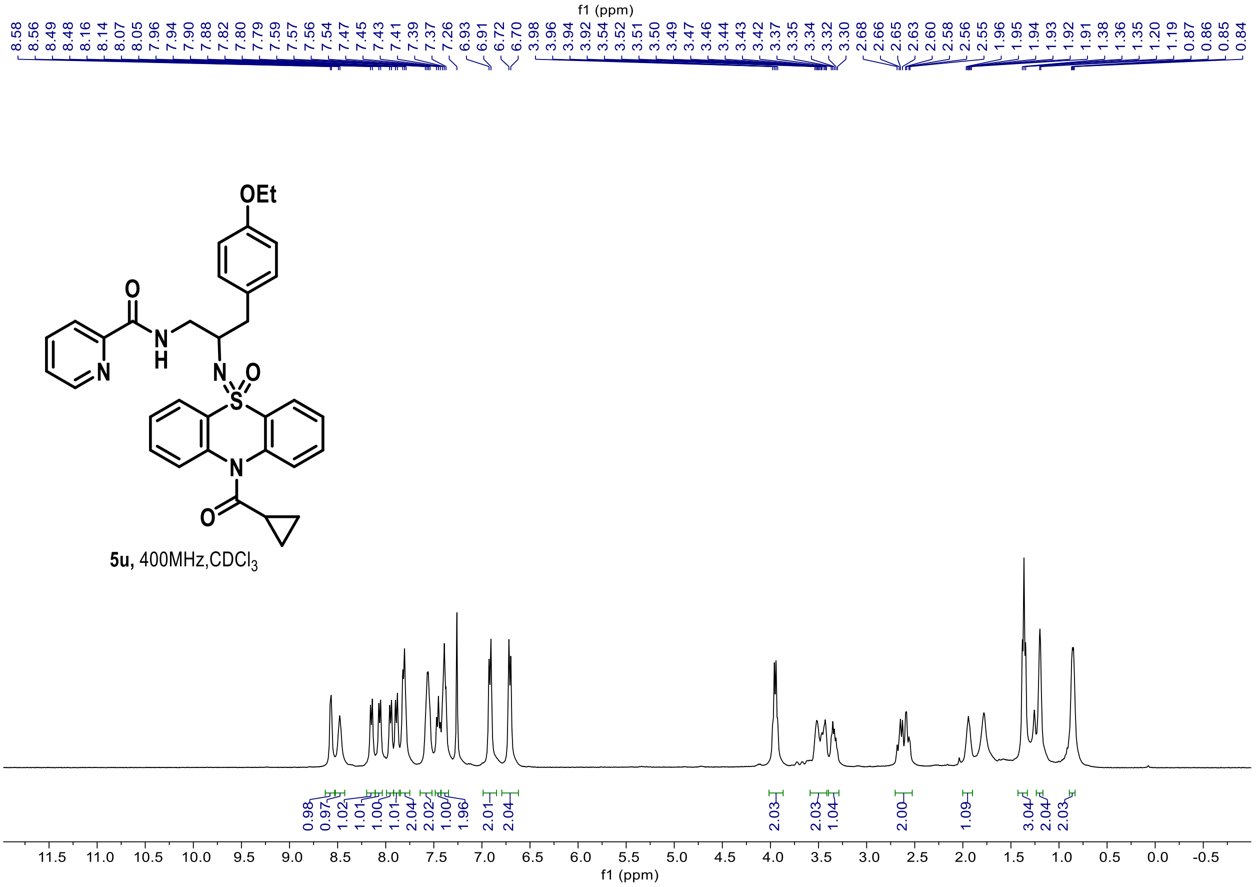
8.58
8.56
8.49
8.48
8.16
8.14
8.07
8.05
7.96
7.94
7.90
7.88
7.82
7.80
7.79
7.59
7.57
7.56
7.54
7.47
7.45
7.43
7.41
7.39
7.37
7.26
6.93
6.91
6.72
6.70
3.98
3.96
3.94
3.92
3.54
3.52
3.51
3.50
3.49
3.47
3.46
3.44
3.43
3.42
3.37
3.35
3.34
3.32
3.30
2.68
2.66
2.65
2.63
2.60
2.58
2.56
2.55
1.96
1.95
1.94
1.93
1.92
1.91
1.38
1.36
1.35
1.20
1.19
0.87
0.86
0.85
0.84
OEt
O
N
H
N
N
S
O
N
O
5u, 400MHz,CDCl3
0.98
0.97
1.02
1.01
1.00
1.01
2.04
2.02
1.00
1.96
2.01
2.04
2.03
2.03
1.04
2.00
1.09
3.04
2.04
2.03
f1 (ppm)

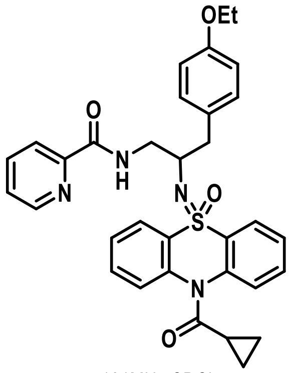

5u, 101MHz,CDCl$_3$

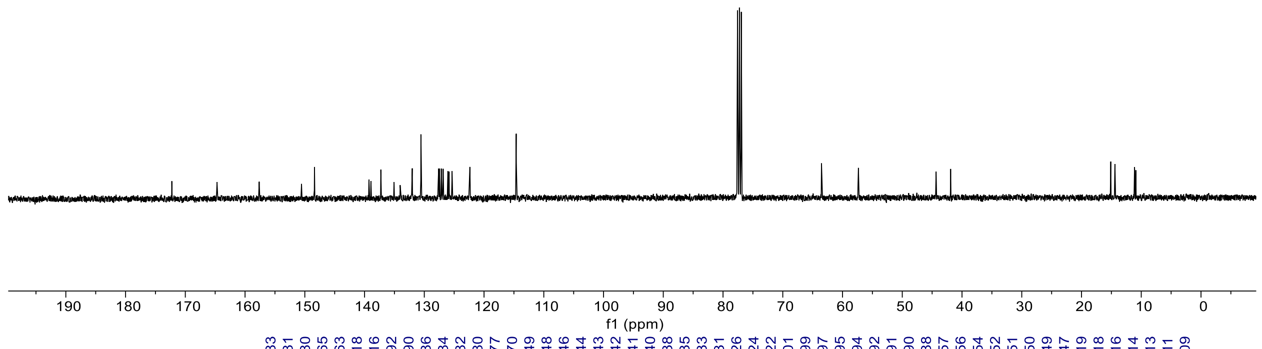

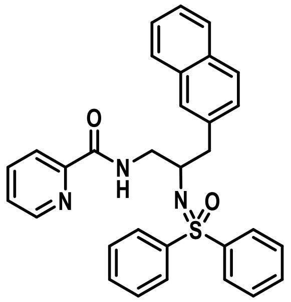

5v, 400MHz,CDCl$_3$

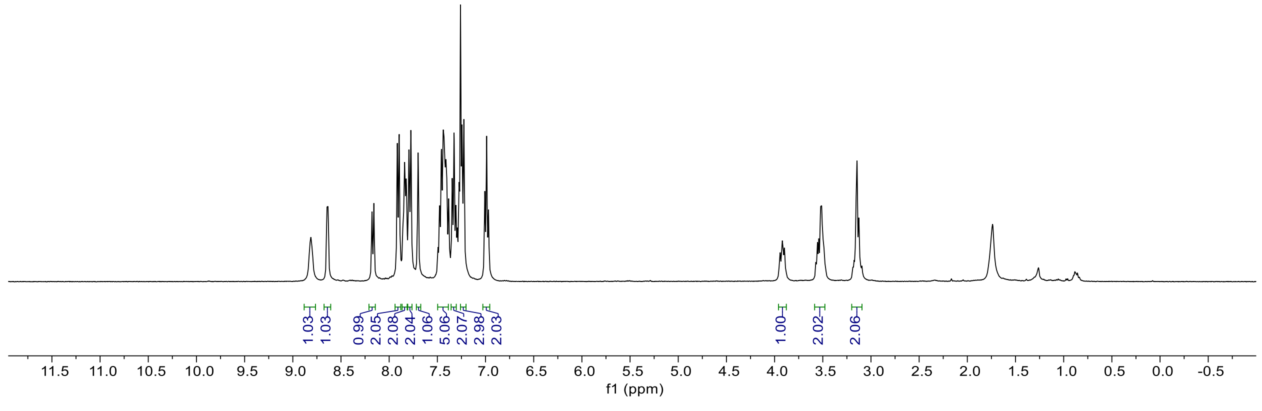

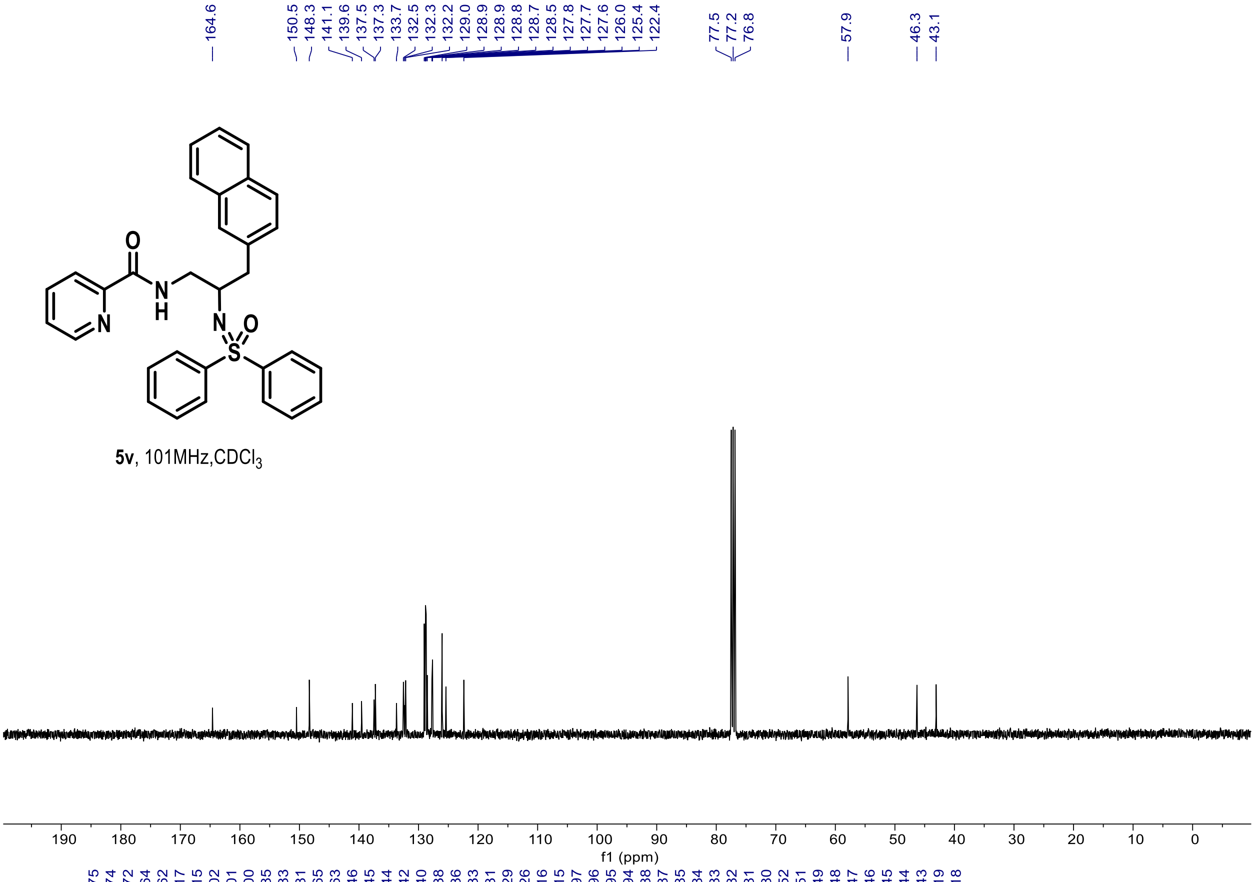

5v, 101MHz,$CDCl_3$

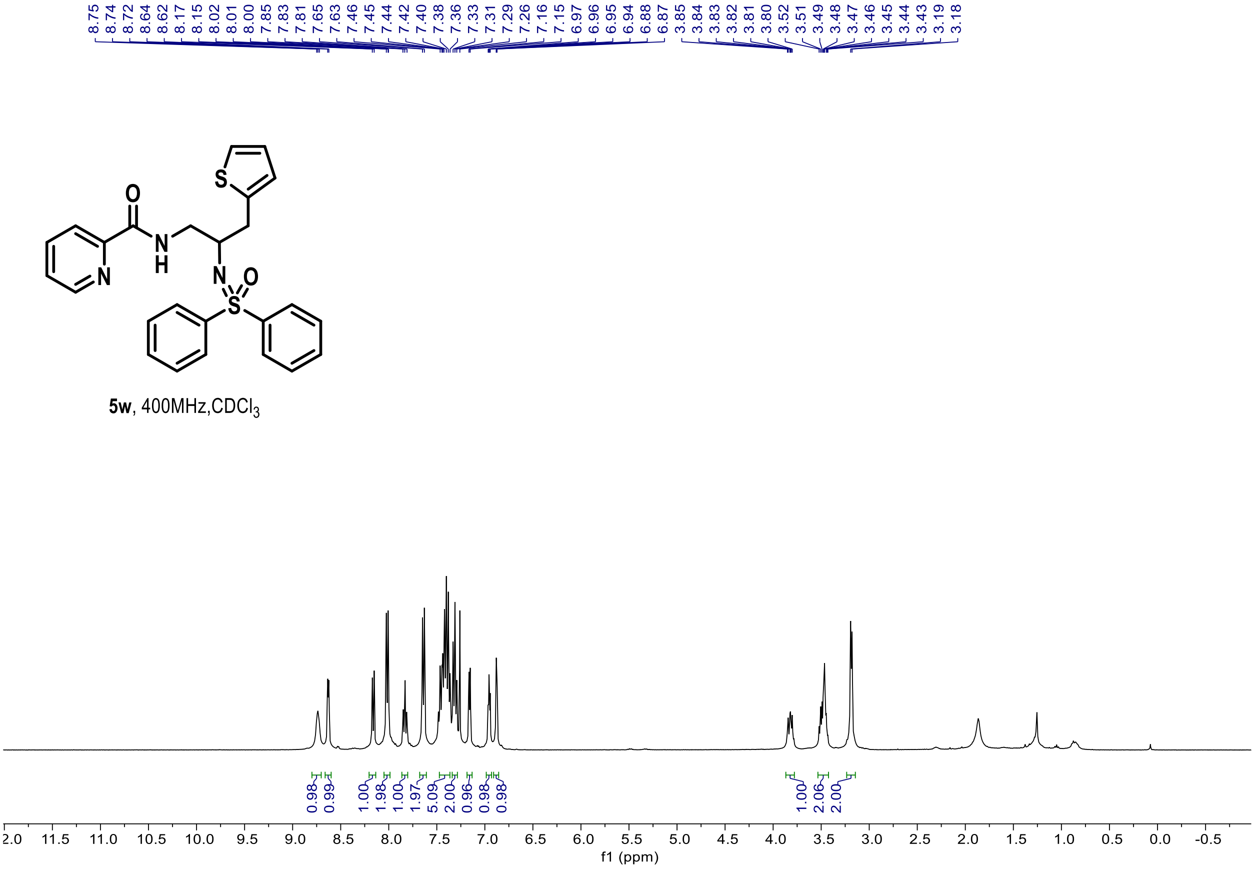

5w, 400MHz,$CDCl_3$

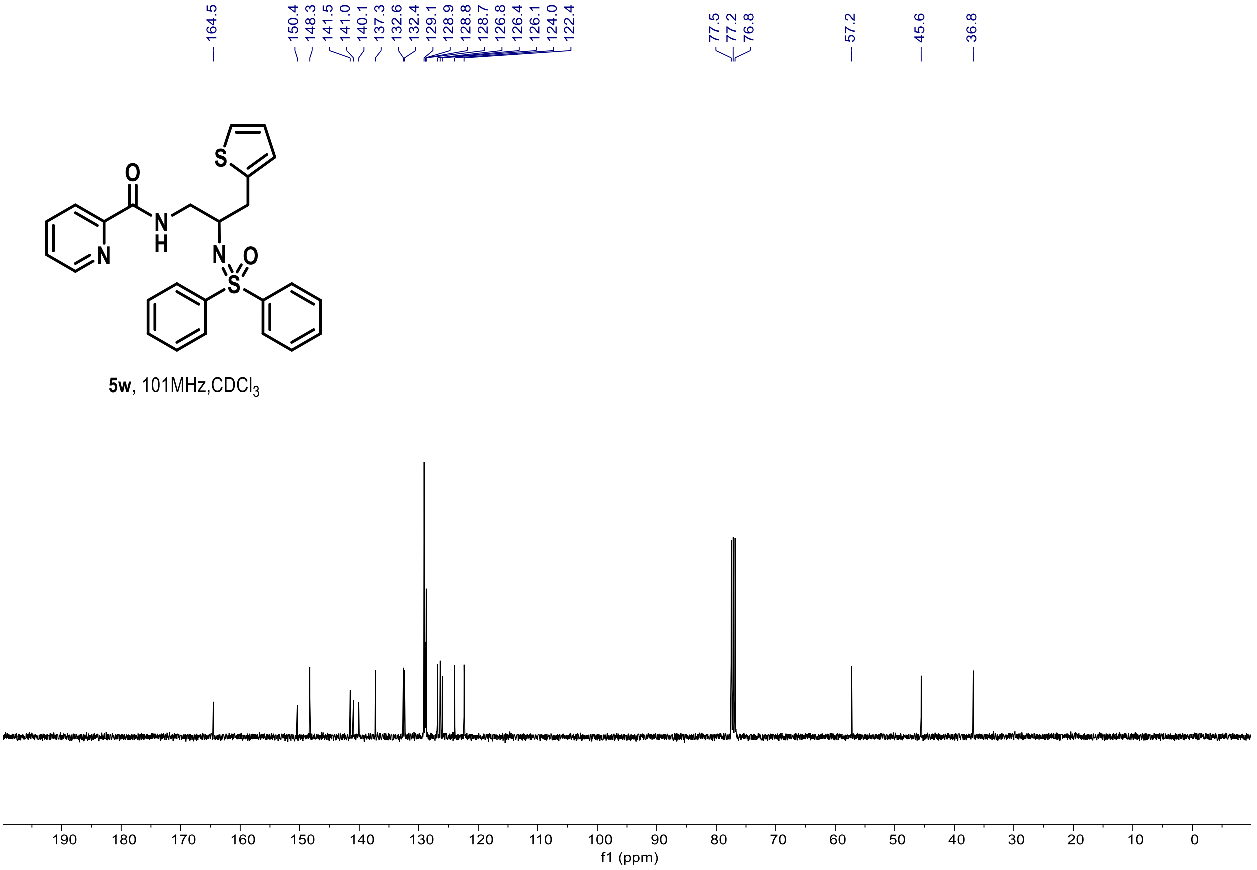

5w, 101MHz,$CDCl_3$

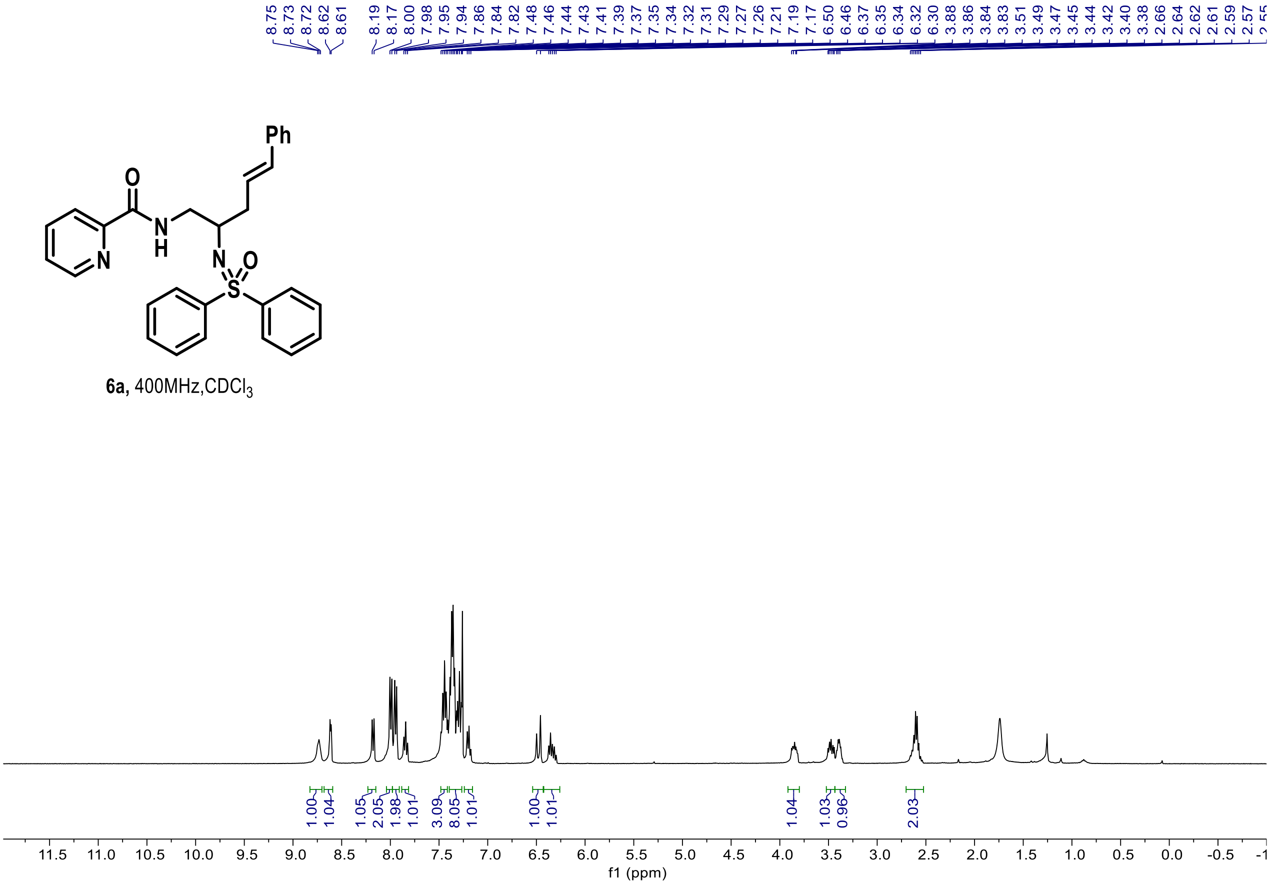

6a, 400MHz,$CDCl_3$

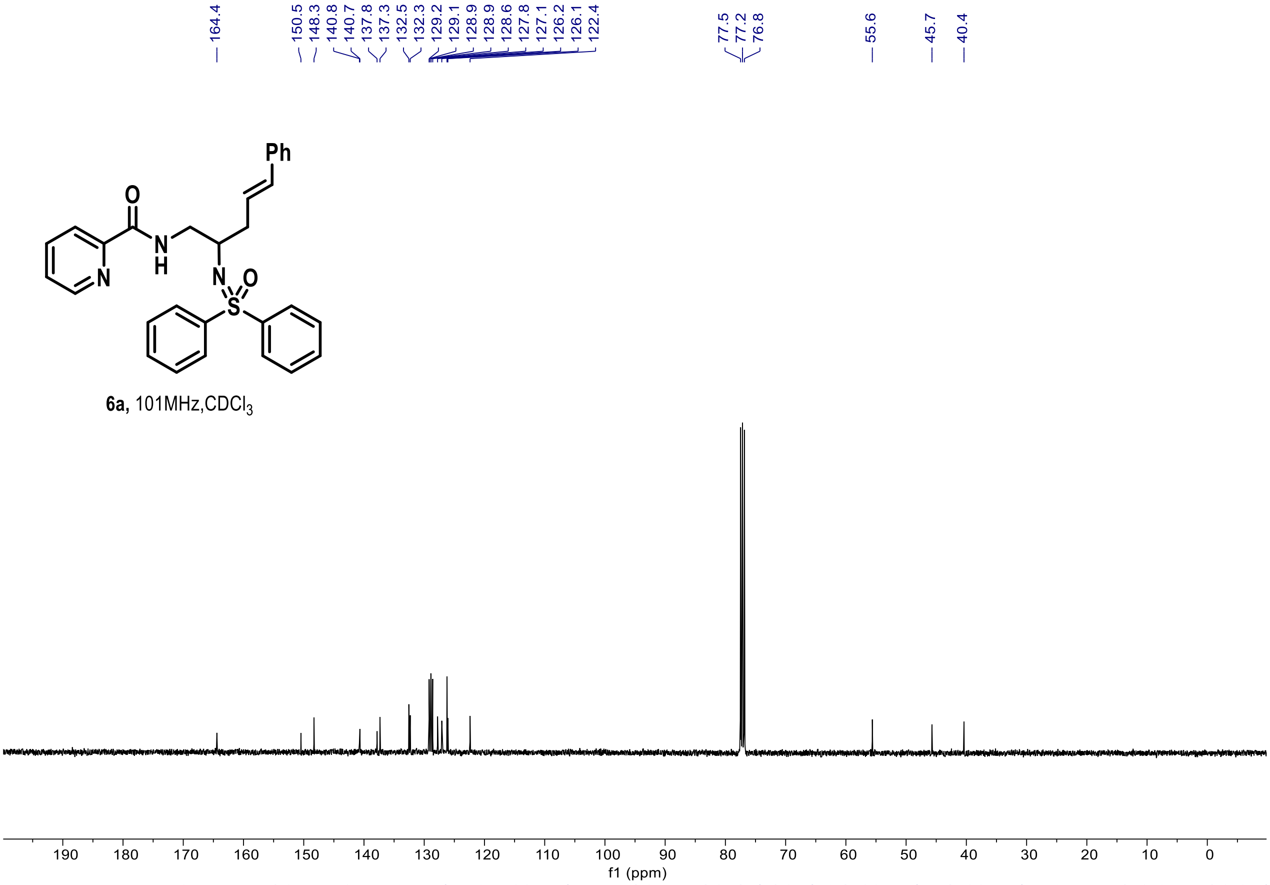

6a, 101MHz,$CDCl_3$

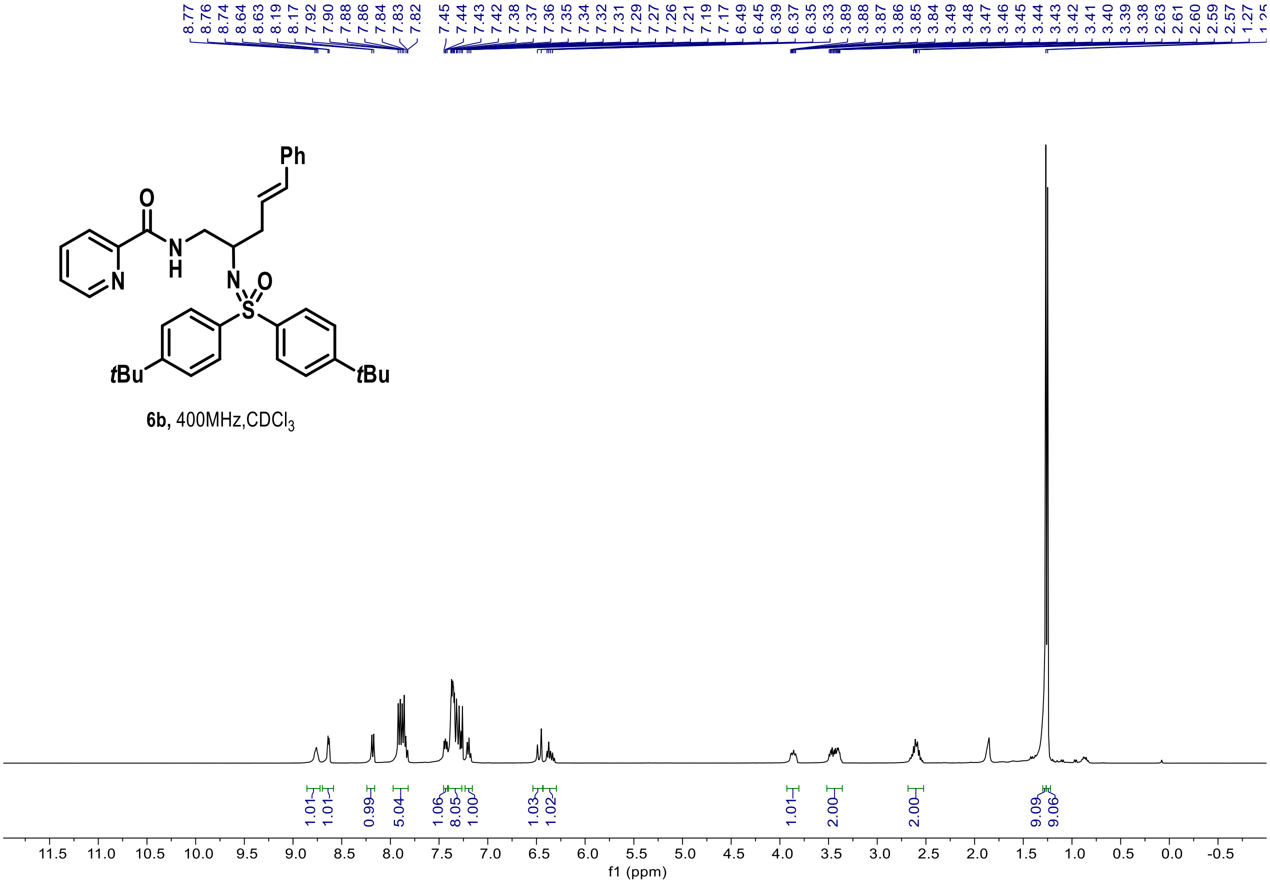

6b, 400MHz,$CDCl_3$

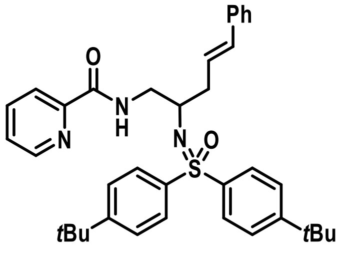

6b, 101MHz,$CDCl_3$

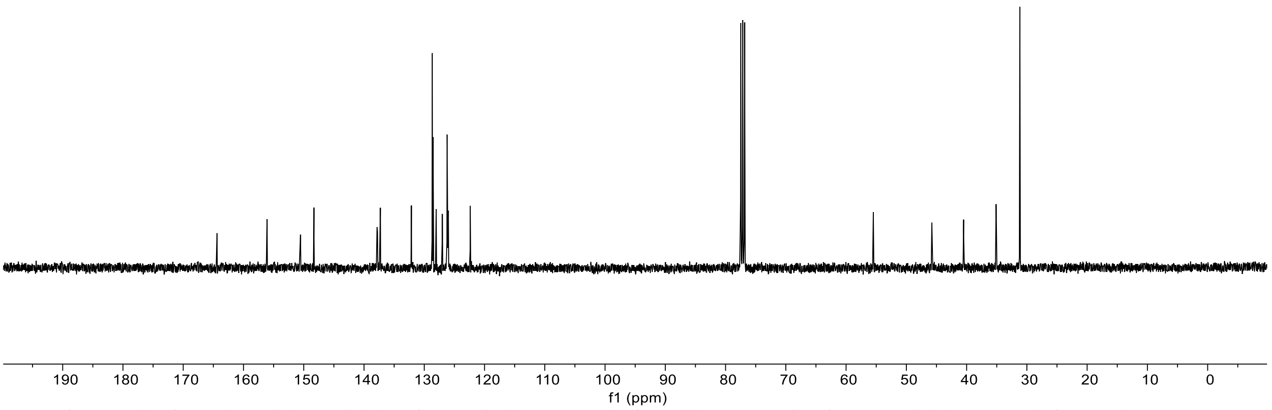

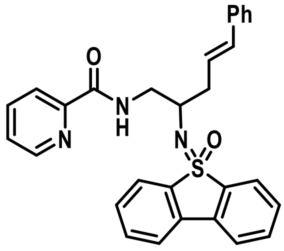

6c, 400MHz,$CDCl_3$

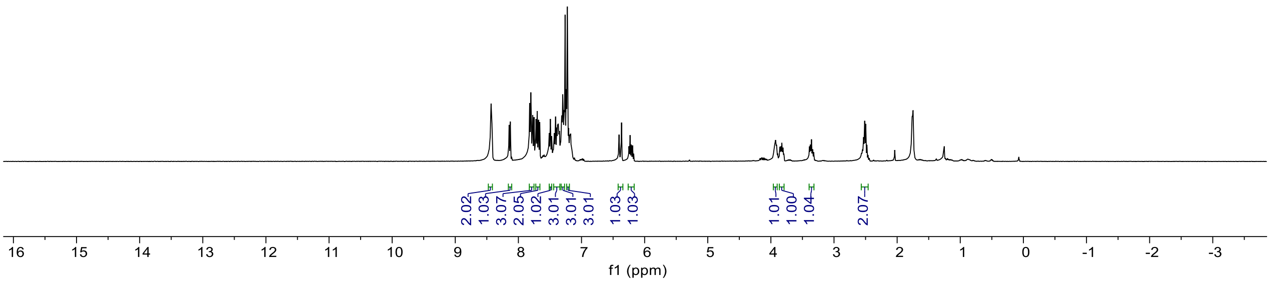

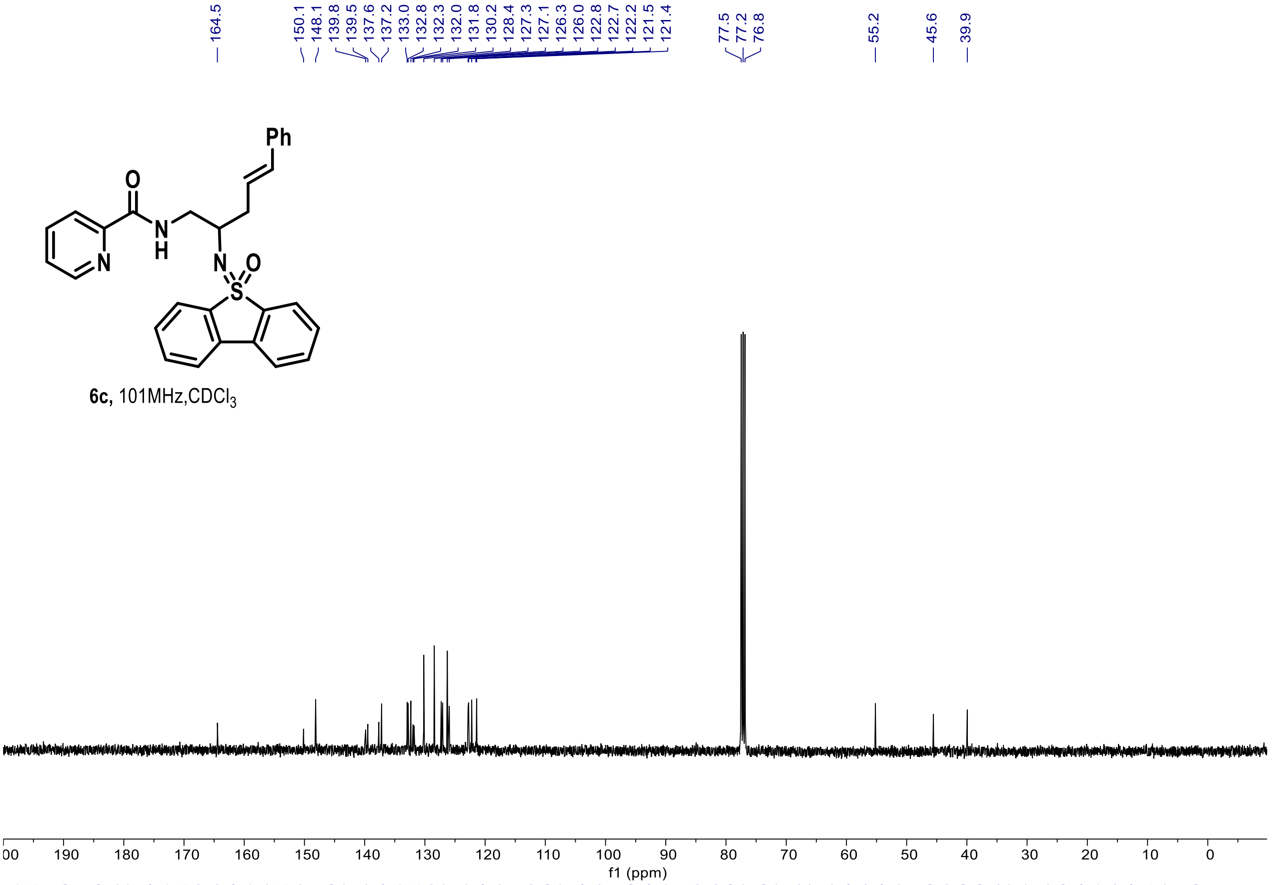

6c, 101MHz,$CDCl_3$

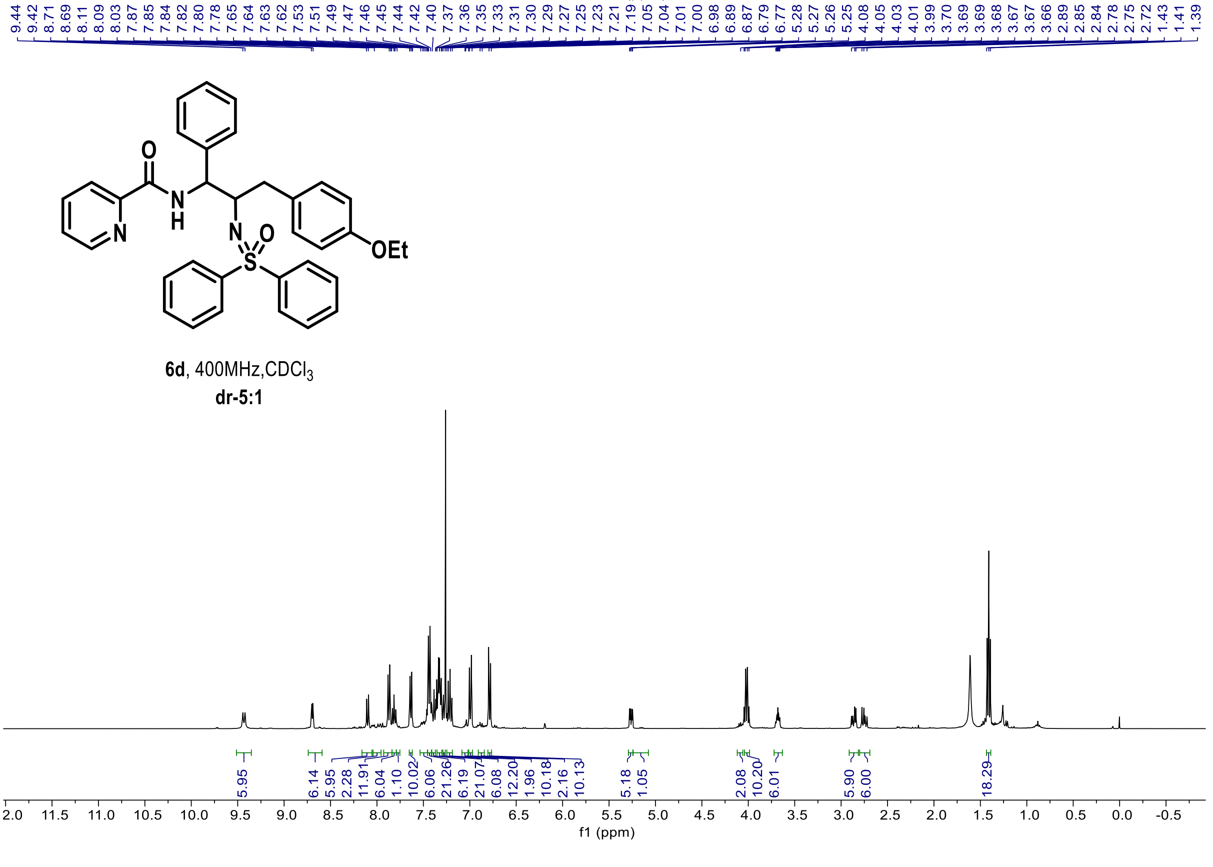

6d, 400MHz,$CDCl_3$

dr-5:1

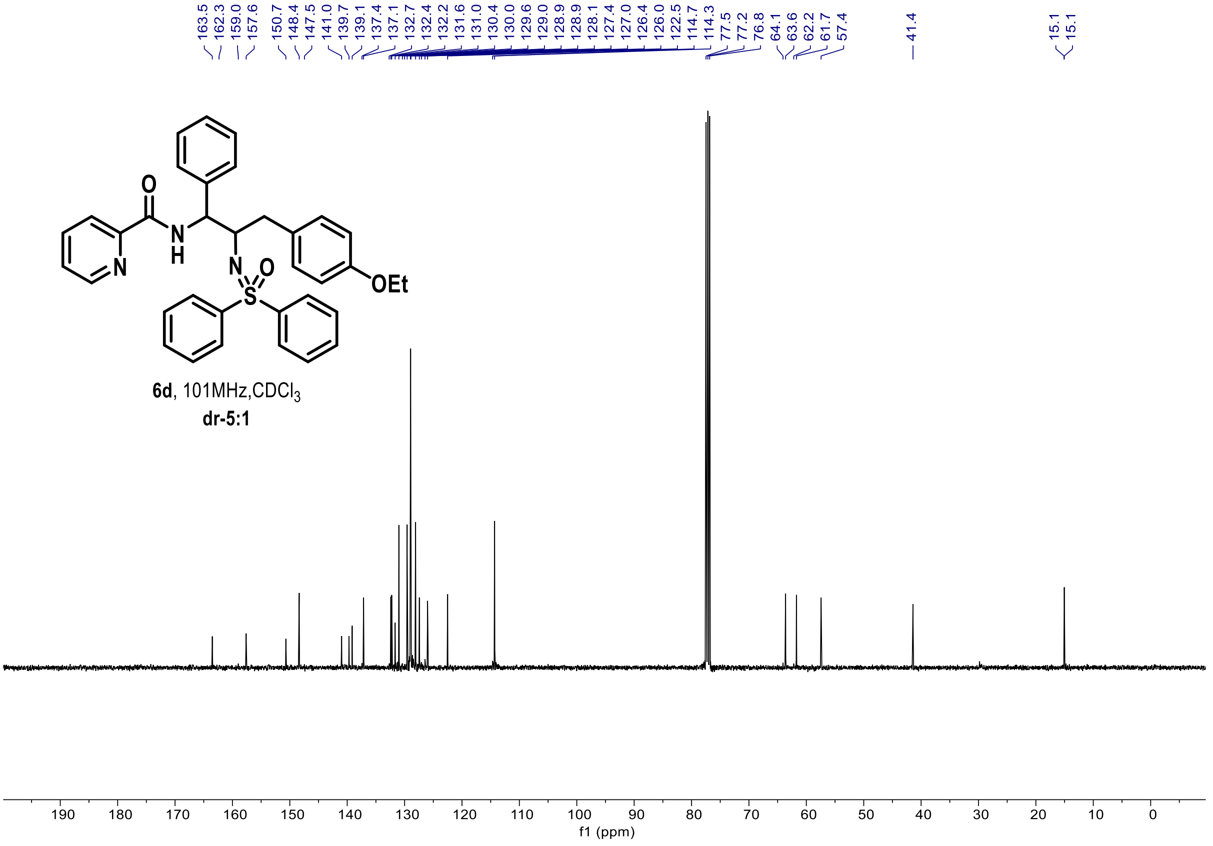

6d, 101MHz,CDCl$_3$
dr-5:1

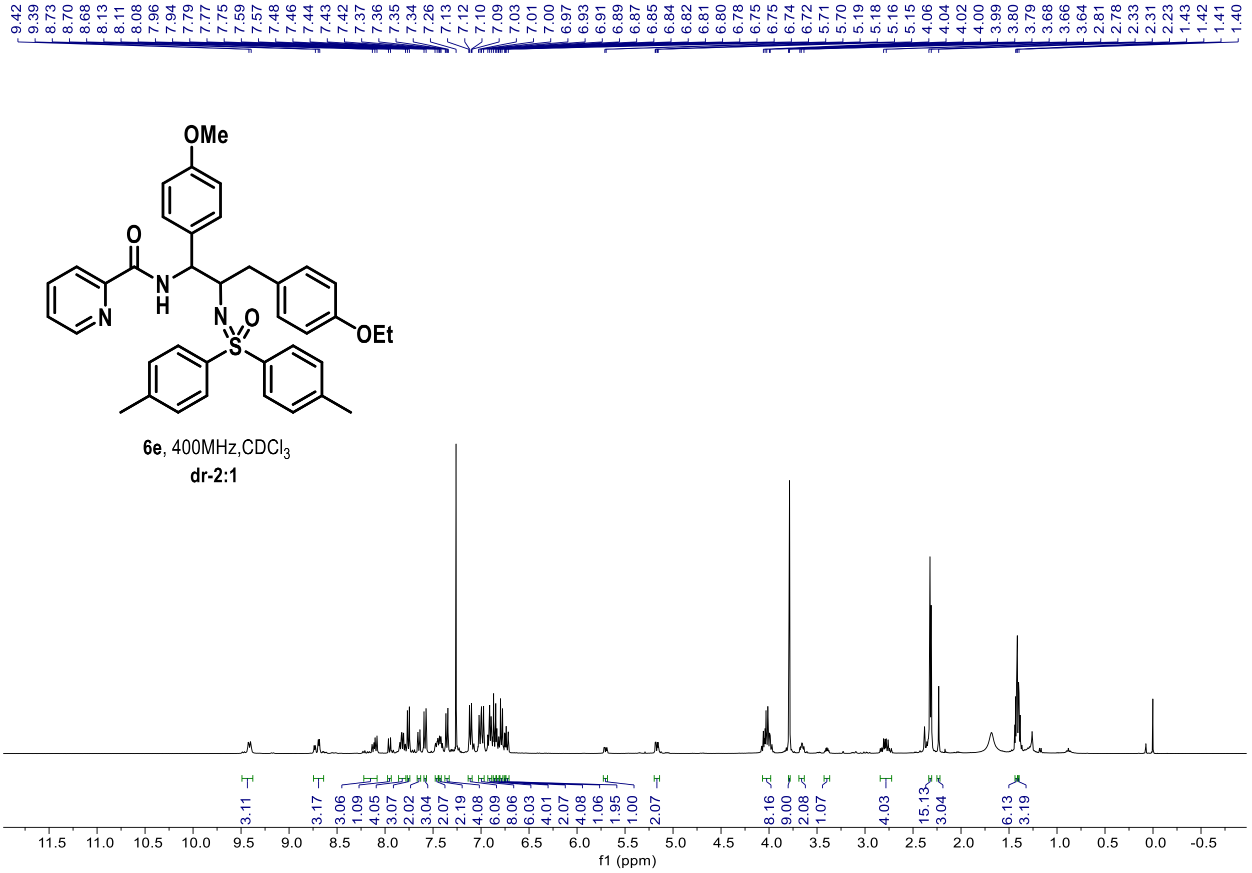

6e, 400MHz,CDCl$_3$
dr-2:1

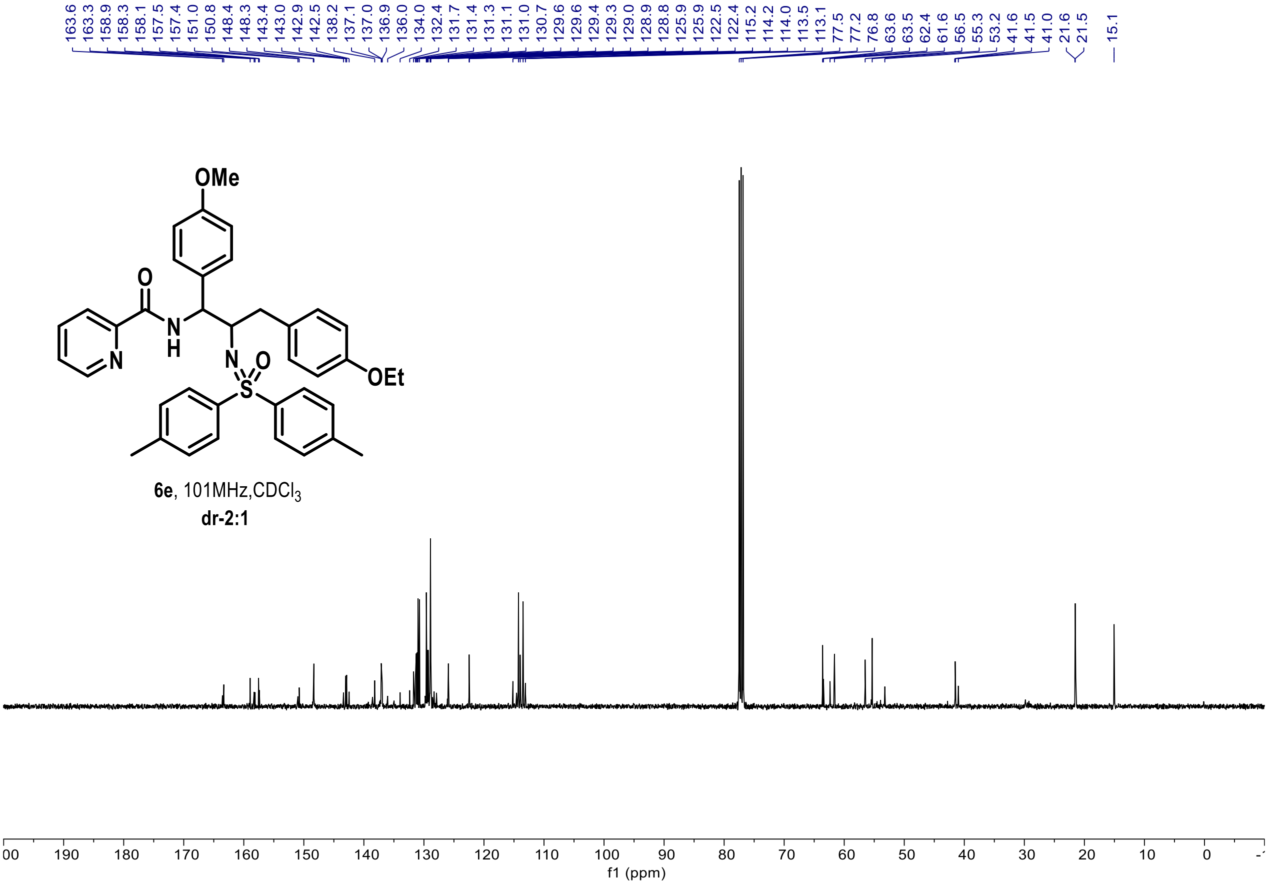

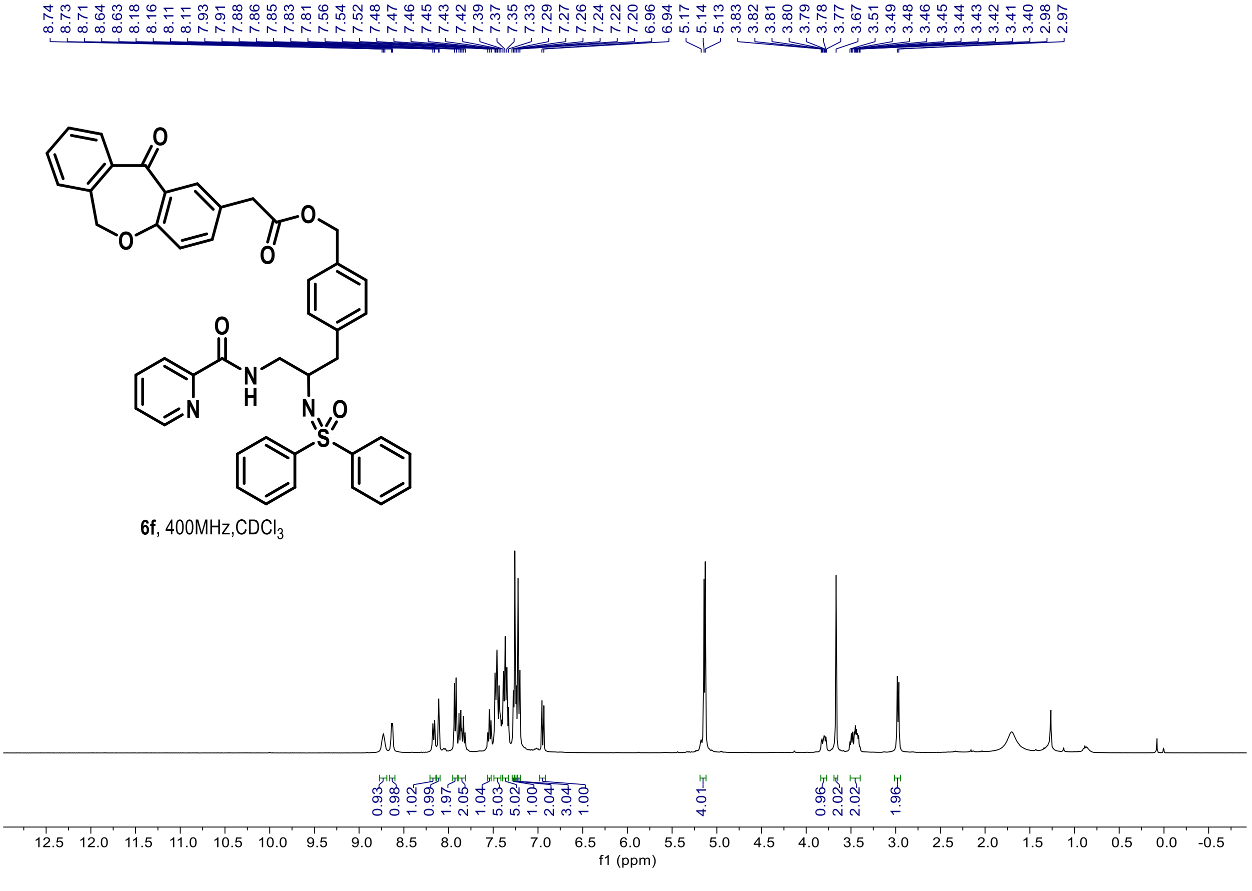

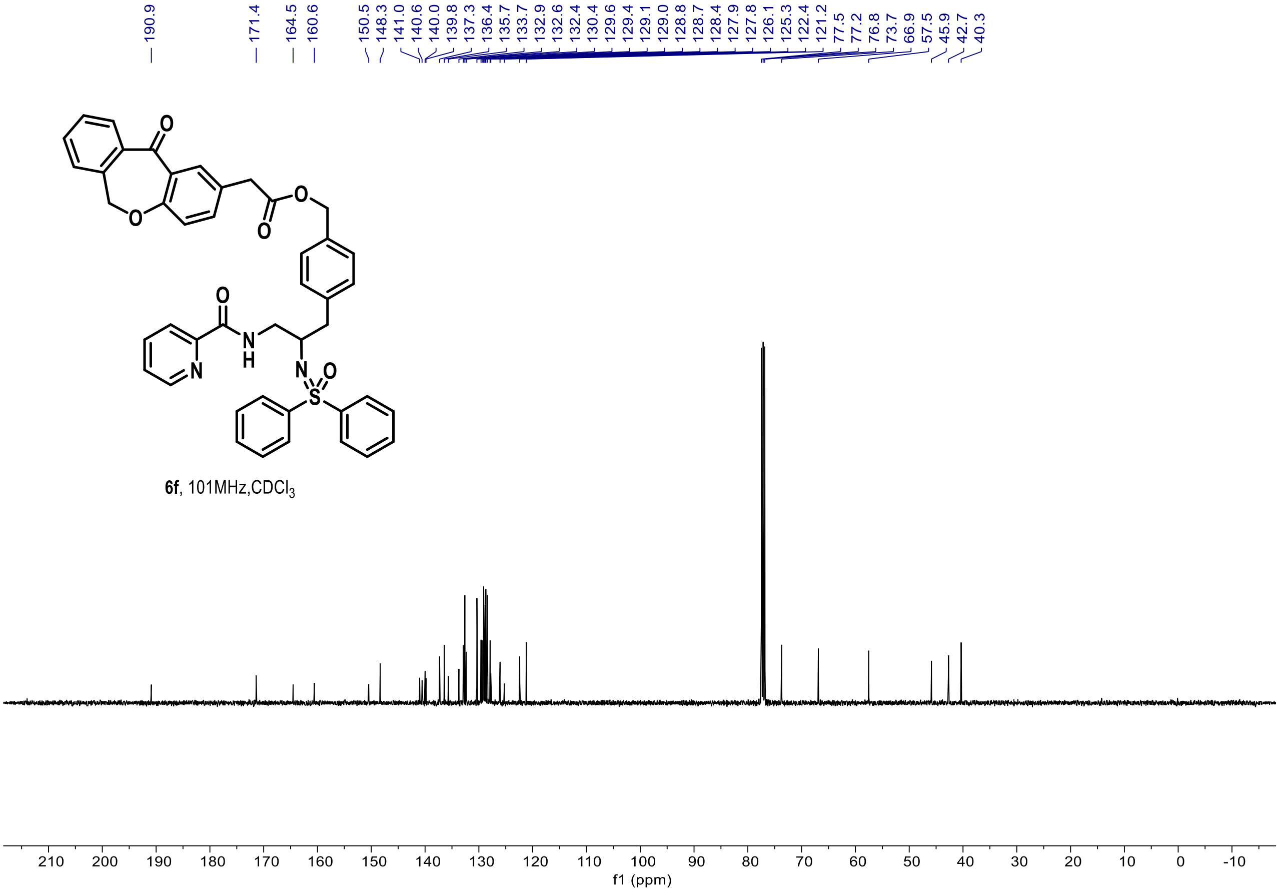
190.9
171.4
164.5
160.6
150.5
148.3
141.0
140.6
140.0
139.8
137.3
136.4
135.7
133.7
132.9
132.6
132.4
130.4
129.6
129.4
129.1
129.0
128.8
128.7
128.4
127.9
127.8
126.1
125.3
122.4
121.2
77.5
77.2
76.8
73.7
66.9
57.5
45.9
42.7
40.3
O
O
O
O
O
N
H
N
N
S
O
6f, 101MHz,CDCl$_3$
210 200 190 180 170 160 150 140 130 120 110 100 90 80 70 60 50 40 30 20 10 0 -10
f1 (ppm)

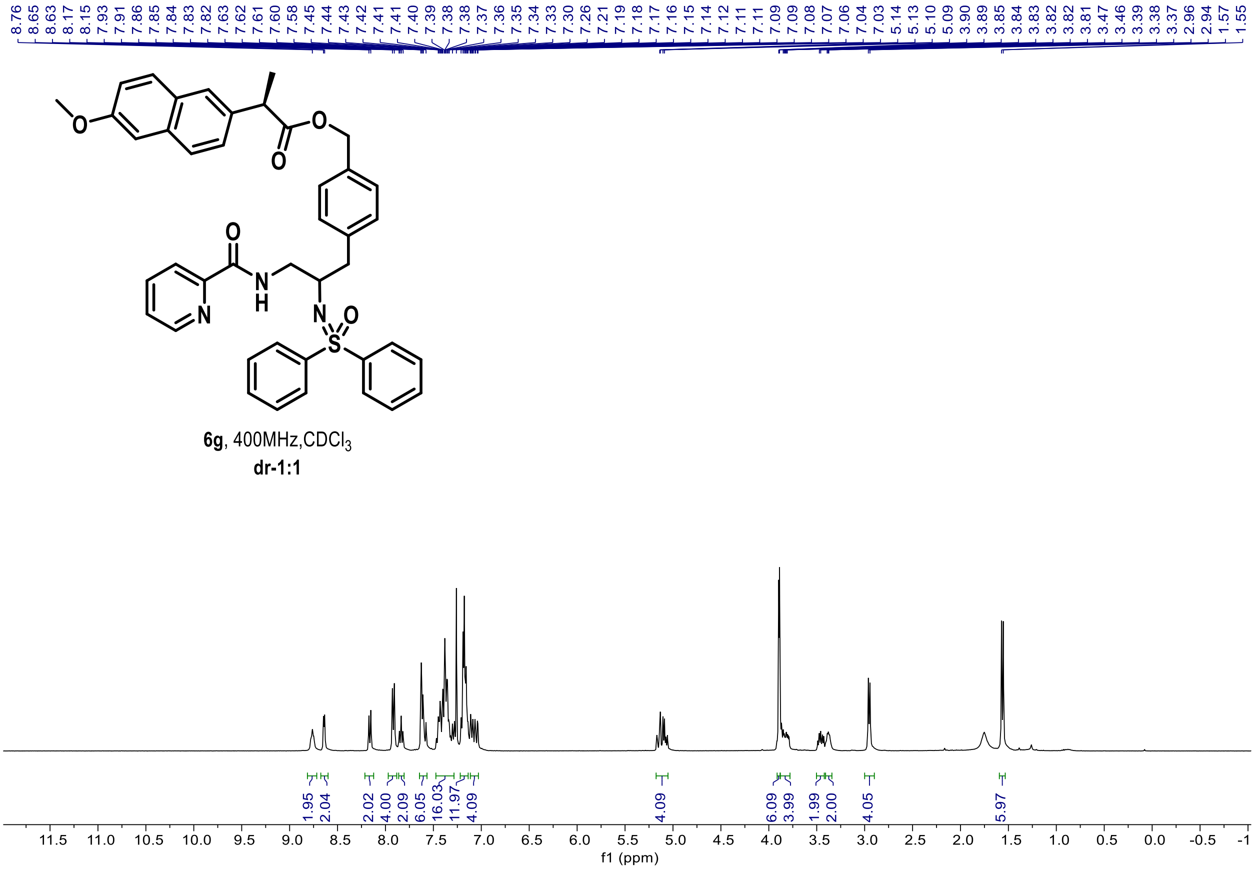
8.76 8.65 8.63 8.17 8.15 7.93 7.91 7.86 7.85 7.84 7.83 7.82 7.63 7.62 7.61 7.60 7.58 7.45 7.44 7.43 7.42 7.41 7.41 7.40 7.39 7.38 7.38 7.37 7.36 7.35 7.34 7.33 7.30 7.26 7.21 7.19 7.18 7.17 7.16 7.15 7.14 7.12 7.11 7.11 7.09 7.09 7.08 7.07 7.06 7.04 7.03 5.14 5.13 5.10 5.09 3.90 3.89 3.85 3.84 3.83 3.82 3.81 3.47 3.46 3.39 3.38 3.37 2.96 2.94 1.57 1.55
O
O
O
O
N
H
N
N
S
O
6g, 400MHz,CDCl$_3$
dr-1:1
1.95 2.04 2.02 4.00 2.09 6.05 16.03 11.97 4.09 4.09 6.09 3.99 1.99 2.00 4.05 5.97
11.5 11.0 10.5 10.0 9.5 9.0 8.5 8.0 7.5 7.0 6.5 6.0 5.5 5.0 4.5 4.0 3.5 3.0 2.5 2.0 1.5 1.0 0.5 0.0 -0.5 -1
f1 (ppm)

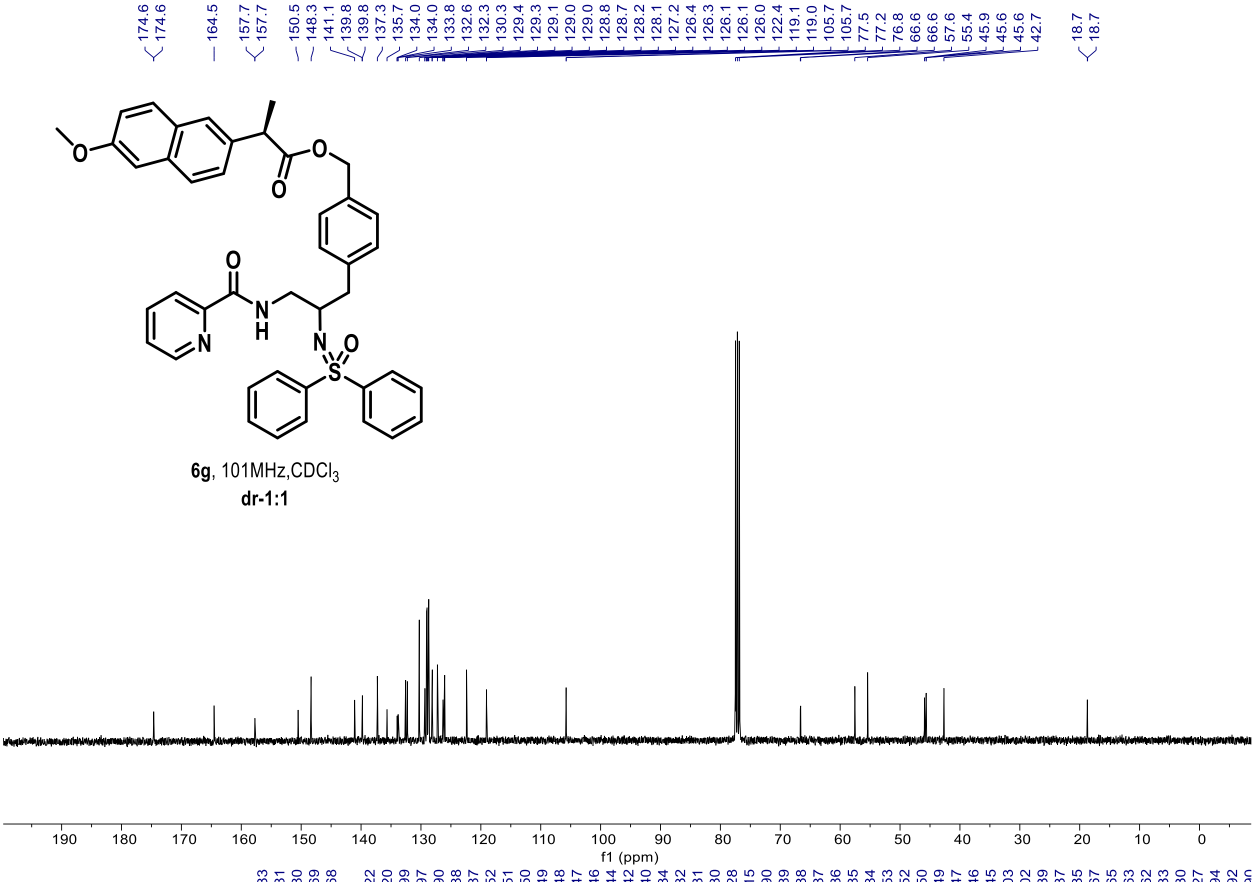
174.6
174.6
164.5
157.7
157.7
150.5
148.3
141.1
139.8
139.8
137.3
135.7
134.0
134.0
133.8
132.6
132.3
130.3
129.4
129.3
129.1
129.0
129.0
128.8
128.7
128.2
128.1
127.2
126.4
126.3
126.1
126.1
126.0
122.4
119.1
119.0
105.7
105.7
77.5
77.2
76.8
66.6
66.6
57.6
55.4
45.9
45.6
45.6
42.7
18.7
18.7
6g, 101MHz,CDCl3
dr-1:1
190 180 170 160 150 140 130 120 110 100 90 80 70 60 50 40 30 20 10 0
f1 (ppm)

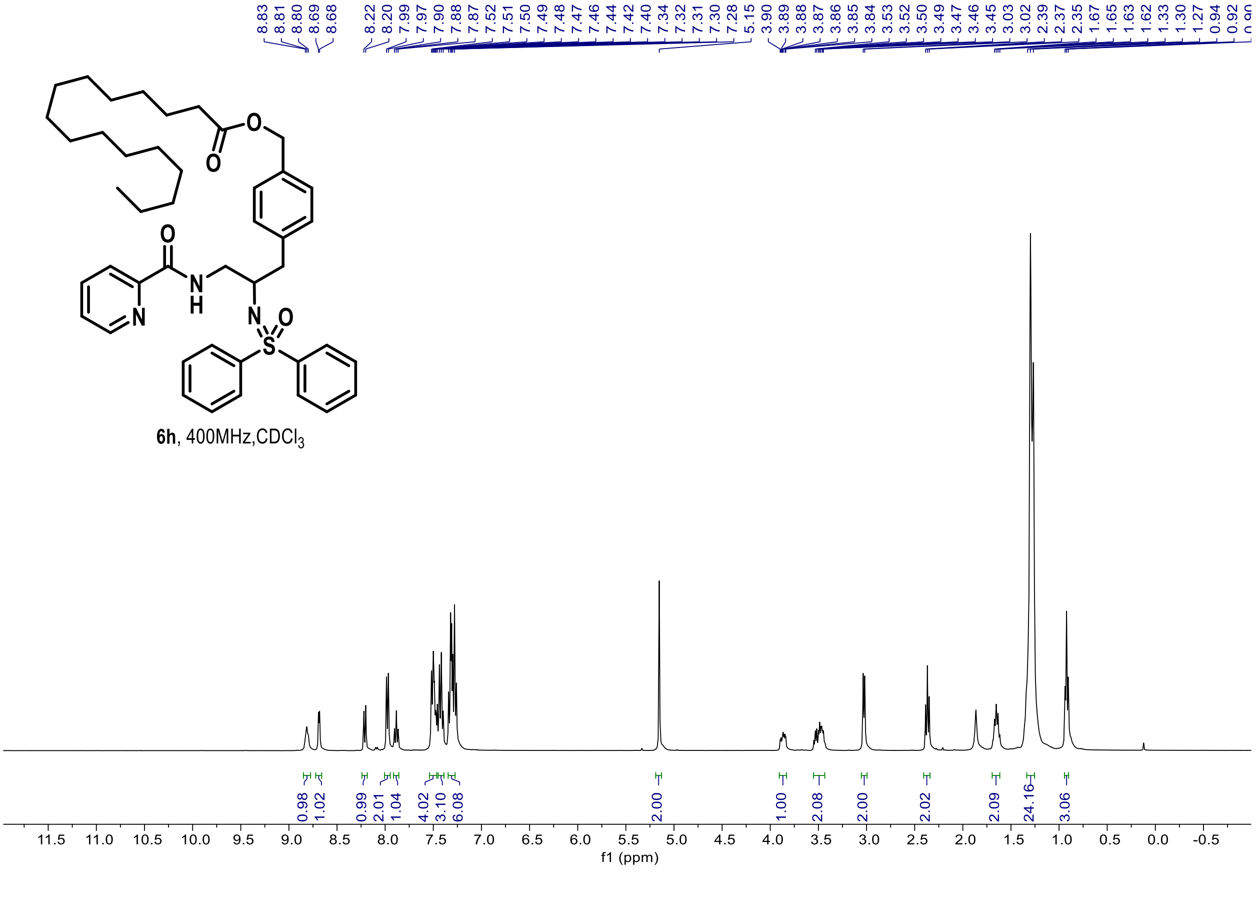
8.83
8.81
8.80
8.69
8.68
8.22
8.20
7.99
7.97
7.90
7.88
7.87
7.52
7.51
7.50
7.49
7.47
7.46
7.44
7.42
7.40
7.34
7.32
7.31
7.30
7.28
5.15
3.90
3.89
3.88
3.87
3.86
3.85
3.84
3.53
3.52
3.50
3.49
3.47
3.46
3.45
3.03
3.02
2.39
2.37
2.35
1.67
1.65
1.63
1.62
1.33
1.30
1.27
0.94
0.92
6h, 400MHz,CDCl3
0.98
1.02
0.99
2.01
1.04
4.02
3.10
6.08
2.00
1.00
2.08
2.00
2.02
2.09
24.16
3.06
11.5 11.0 10.5 10.0 9.5 9.0 8.5 8.0 7.5 7.0 6.5 6.0 5.5 5.0 4.5 4.0 3.5 3.0 2.5 2.0 1.5 1.0 0.5 0.0 -0.5
f1 (ppm)

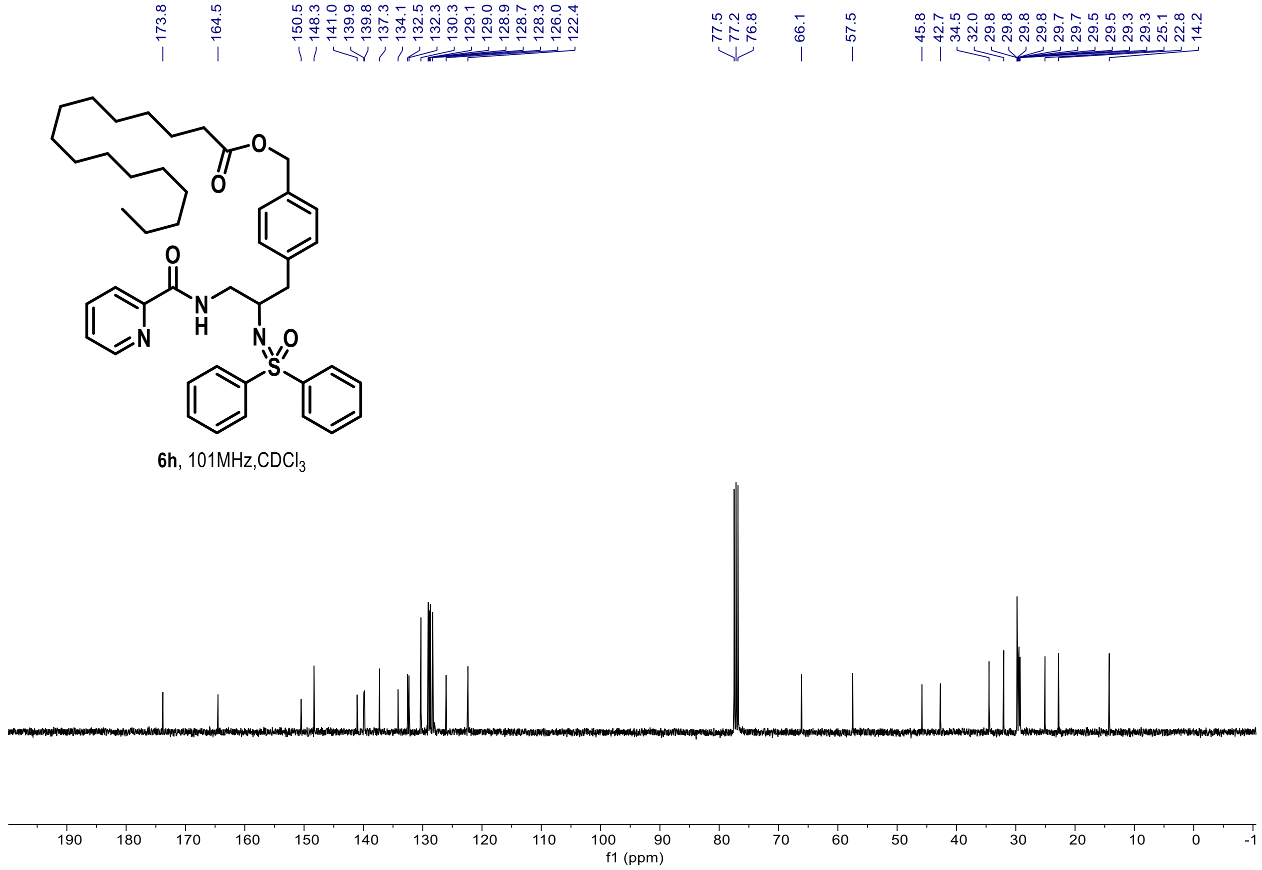
173.8
164.5
150.5
148.3
141.0
139.9
139.8
137.3
134.1
132.5
132.3
130.3
129.1
129.0
128.9
128.7
128.3
126.0
122.4
77.5
77.2
76.8
66.1
57.5
45.8
42.7
34.5
32.0
29.8
29.8
29.8
29.8
29.7
29.7
29.5
29.5
29.3
29.3
25.1
22.8
14.2
6h, 101MHz,CDCl3
f1 (ppm)

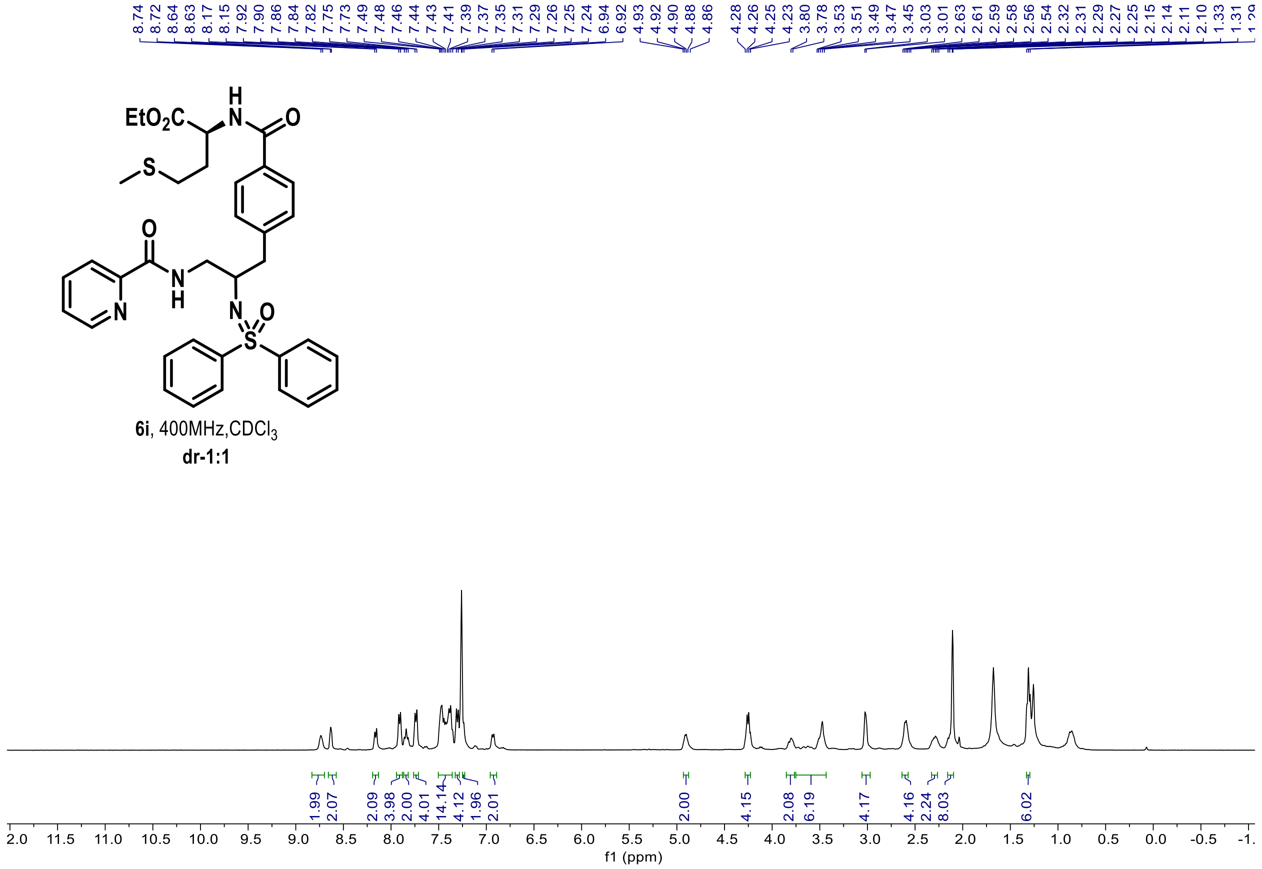
8.74
8.72
8.64
8.63
8.17
8.15
7.92
7.90
7.86
7.84
7.82
7.75
7.73
7.49
7.48
7.46
7.44
7.43
7.41
7.39
7.37
7.35
7.31
7.29
7.26
7.25
7.24
6.94
6.92
4.93
4.92
4.90
4.88
4.86
4.28
4.26
4.25
4.23
3.80
3.78
3.53
3.51
3.49
3.47
3.45
3.03
3.01
2.63
2.61
2.59
2.58
2.56
2.54
2.32
2.31
2.29
2.27
2.25
2.15
2.14
2.11
2.10
1.33
1.31
EtO2C
6i, 400MHz,CDCl3
dr-1:1
1.99
2.07
2.09
3.98
2.00
4.01
14.14
4.12
1.96
2.01
2.00
4.15
2.08
6.19
4.17
4.16
2.24
8.03
6.02
f1 (ppm)

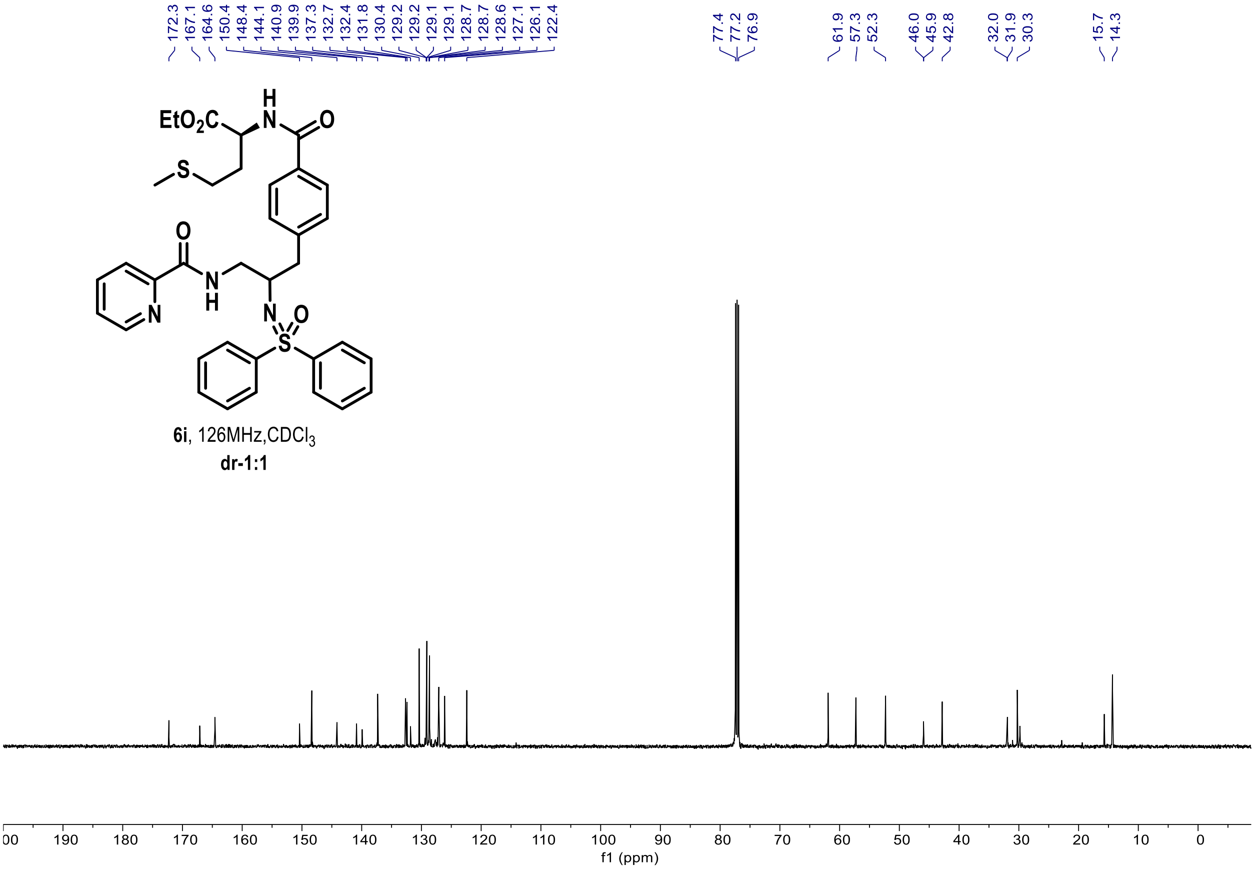
172.3
167.1
164.6
150.4
148.4
144.1
140.9
139.9
137.3
132.7
132.4
131.8
130.4
129.2
129.2
129.1
129.1
128.7
128.7
128.6
127.1
126.1
122.4
77.4
77.2
76.9
61.9
57.3
52.3
46.0
45.9
42.8
32.0
31.9
30.3
15.7
14.3
EtO$_2$C
6i, 126MHz,CDCl$_3$
dr-1:1
f1 (ppm)

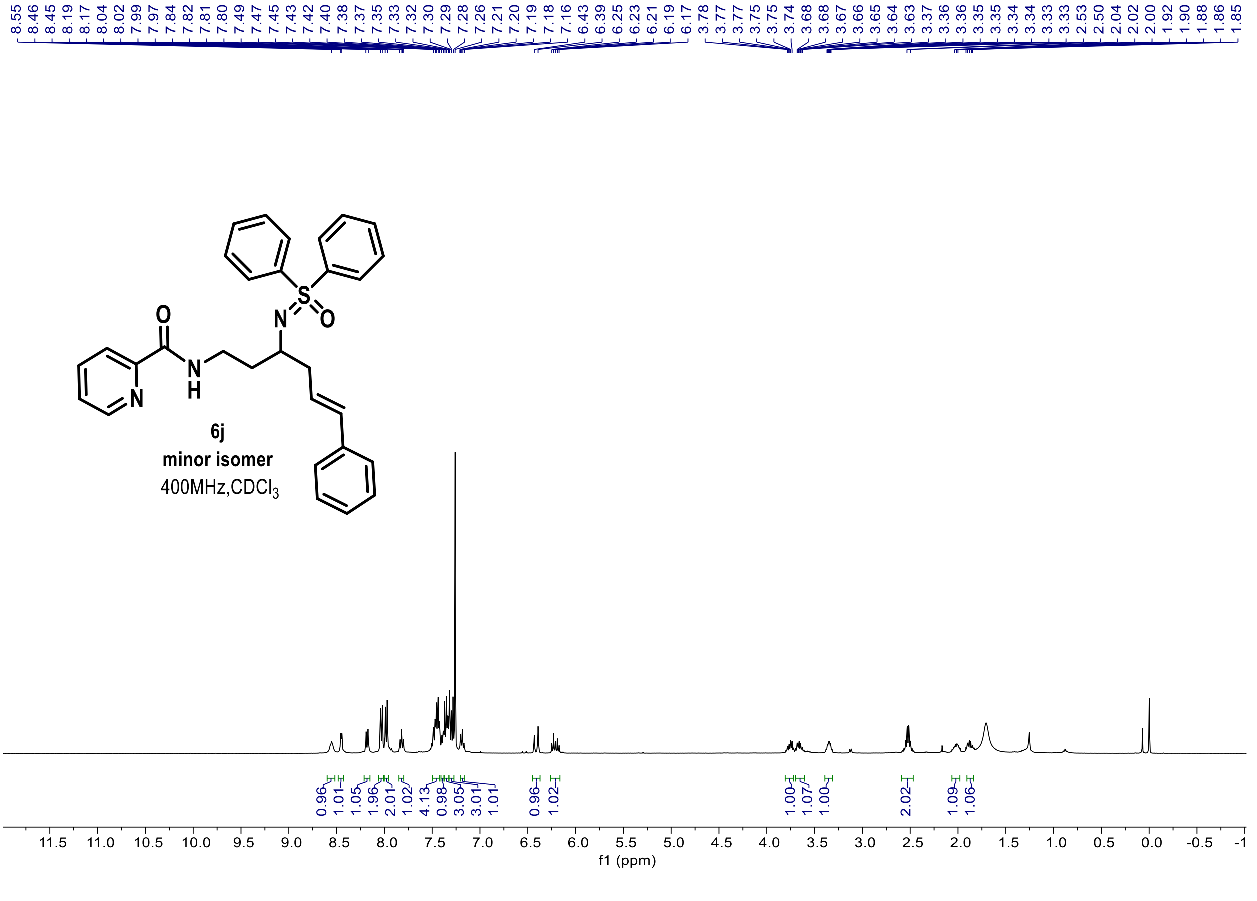
8.55
8.46
8.45
8.19
8.17
8.04
8.02
7.99
7.97
7.84
7.82
7.81
7.80
7.49
7.47
7.45
7.43
7.42
7.40
7.38
7.37
7.35
7.33
7.32
7.30
7.29
7.28
7.26
7.21
7.20
7.19
7.18
7.16
6.43
6.39
6.25
6.23
6.21
6.19
6.17
3.78
3.77
3.75
3.74
3.68
3.67
3.66
3.65
3.64
3.63
3.37
3.36
3.36
3.35
3.35
3.34
3.34
3.33
3.33
2.53
2.50
2.04
2.02
2.00
1.92
1.90
1.88
1.86
1.85
6j
minor isomer
400MHz,CDCl$_3$
0.96
1.01
1.05
1.96
2.01
1.02
4.13
0.98
3.05
3.01
1.01
0.96
1.02
1.00
1.07
1.00
2.02
1.09
1.06
f1 (ppm)

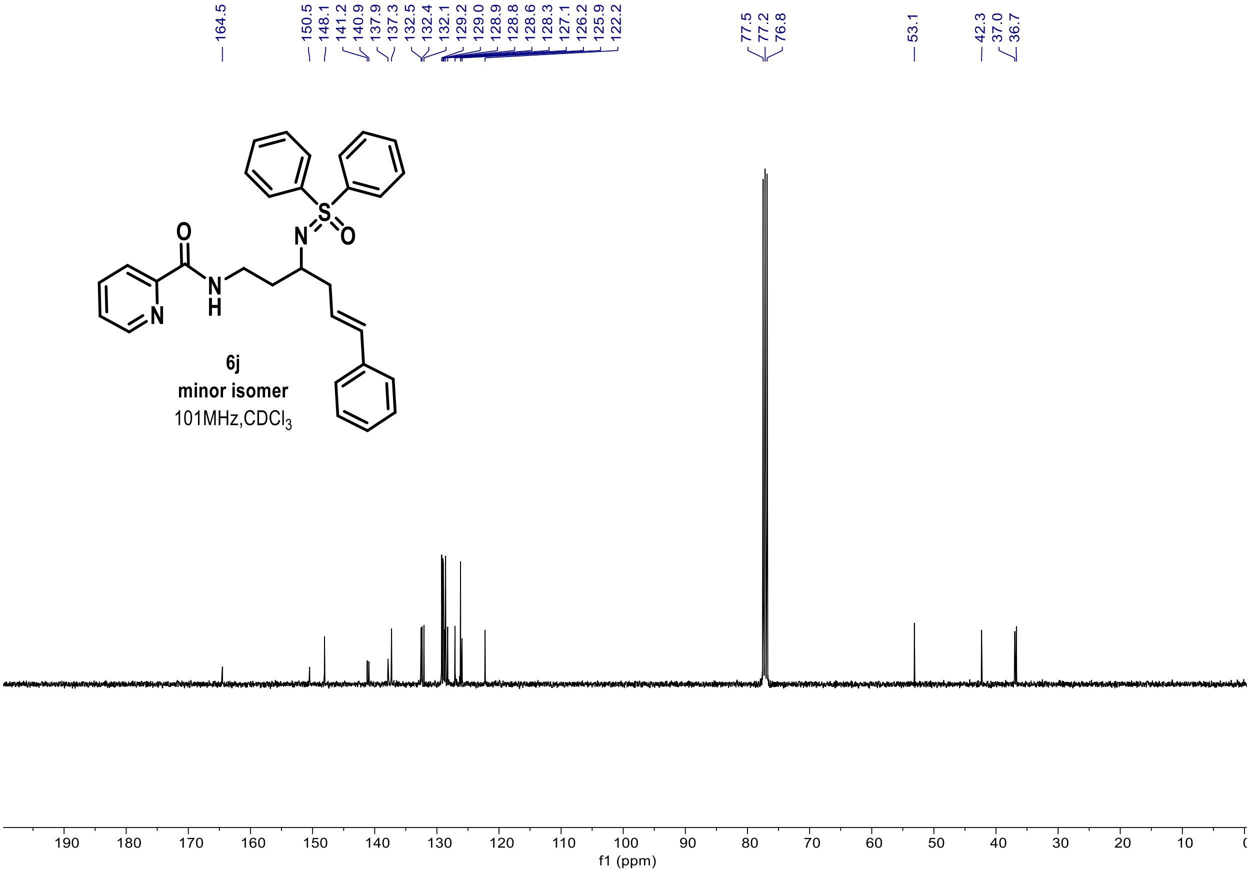
164.5
150.5
148.1
141.2
140.9
137.9
137.3
132.5
132.4
132.1
129.2
129.0
128.9
128.8
128.6
128.3
127.1
126.2
125.9
122.2
77.5
77.2
76.8
53.1
42.3
37.0
36.7
6j
minor isomer
101MHz,CDCl$_3$
f1 (ppm)

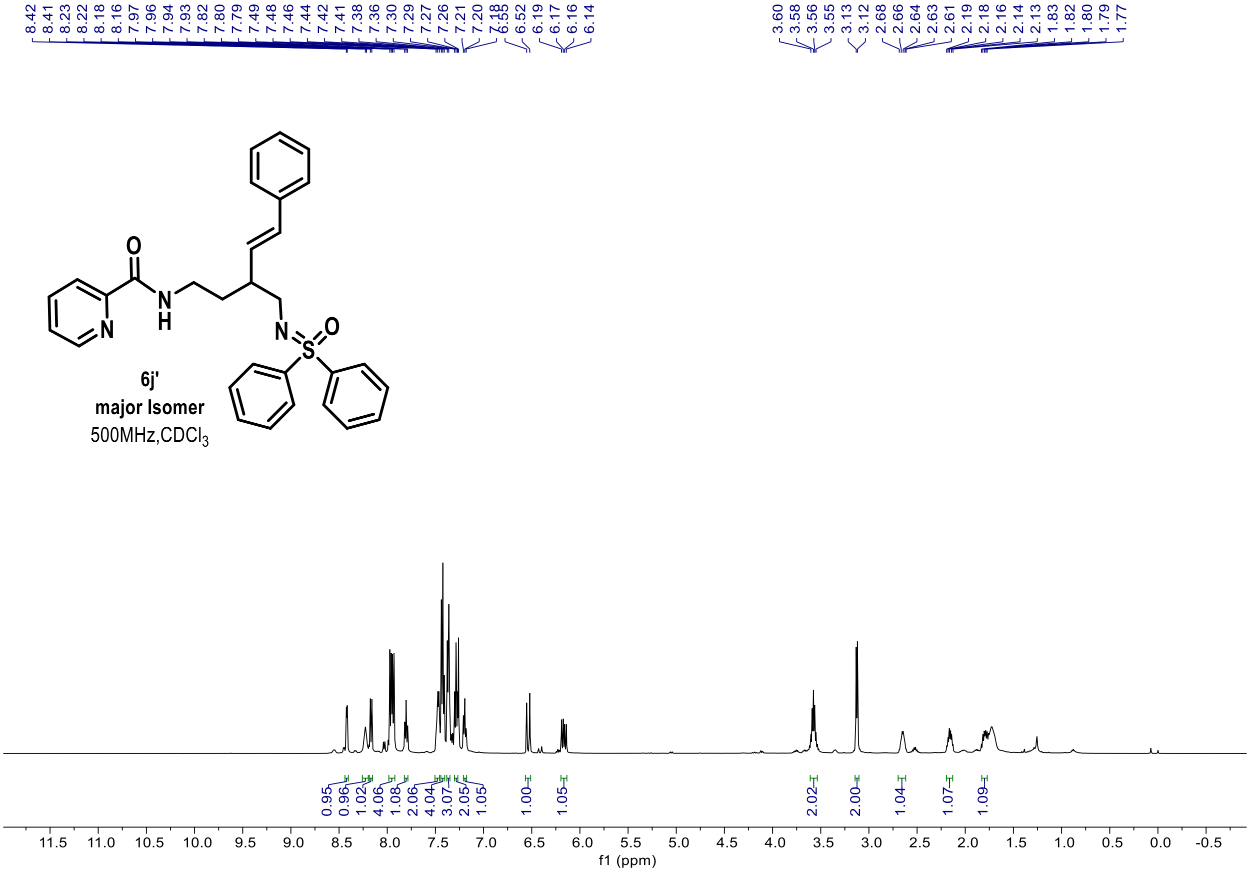
8.42
8.41
8.23
8.22
8.18
8.16
7.97
7.96
7.94
7.93
7.82
7.80
7.79
7.49
7.48
7.46
7.44
7.42
7.41
7.38
7.36
7.30
7.29
7.27
7.26
7.21
7.20
7.18
6.55
6.52
6.19
6.17
6.16
6.14
3.60
3.58
3.56
3.55
3.13
3.12
2.68
2.66
2.64
2.63
2.61
2.19
2.18
2.16
2.14
2.13
1.83
1.82
1.80
1.79
1.77
6j'
major Isomer
500MHz,CDCl$_3$
0.95
0.96
1.02
4.06
1.08
2.06
4.04
3.07
2.05
1.05
1.00
1.05
2.02
2.00
1.04
1.07
1.09
f1 (ppm)

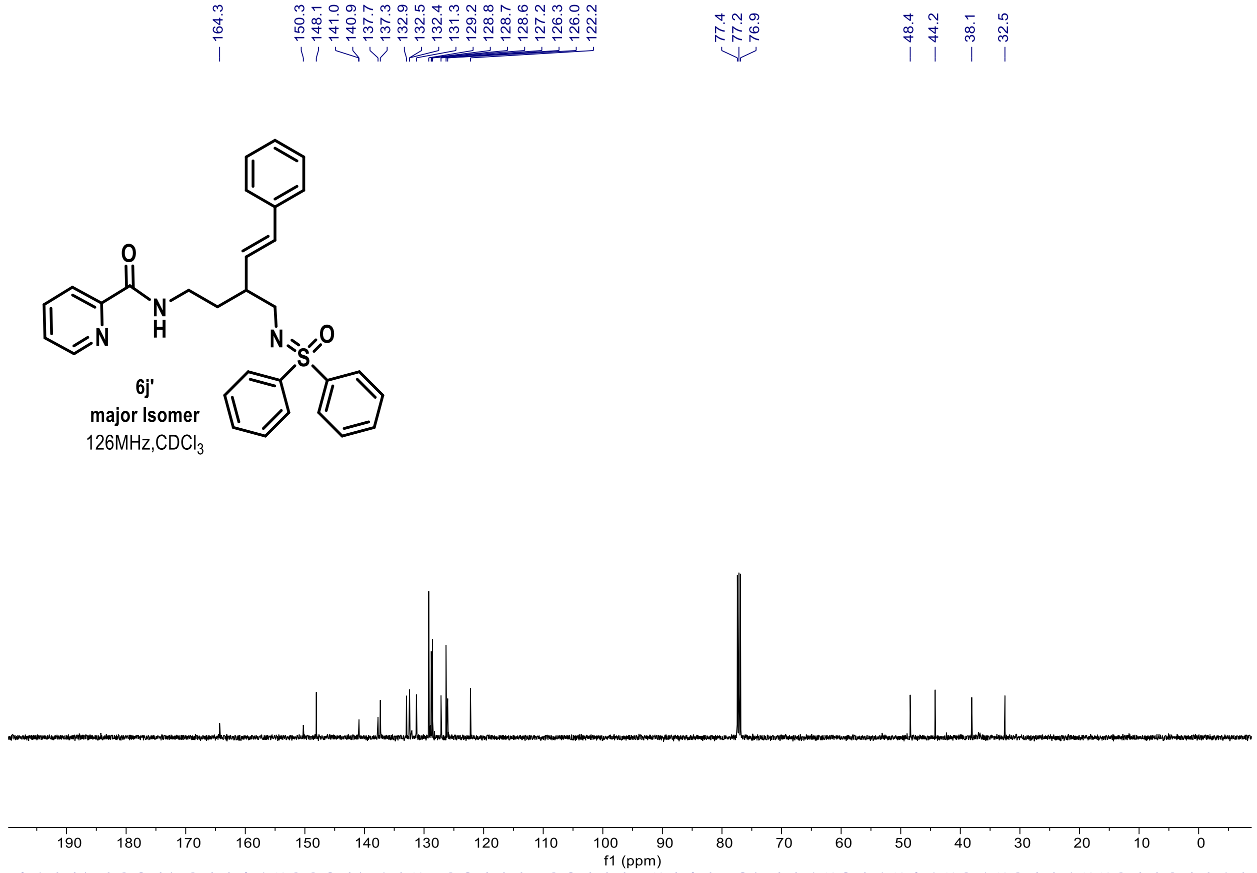
164.3
150.3
148.1
141.0
140.9
137.7
137.3
132.9
132.5
132.4
131.3
129.2
128.8
128.7
128.6
127.2
126.3
126.0
122.2
77.4
77.2
76.9
48.4
44.2
38.1
32.5
O
N
H
N
N
S
O
6j'
major Isomer
126MHz,CDCl3
190
180
170
160
150
140
130
120
110
100
90
80
70
60
50
40
30
20
10
0
f1 (ppm)

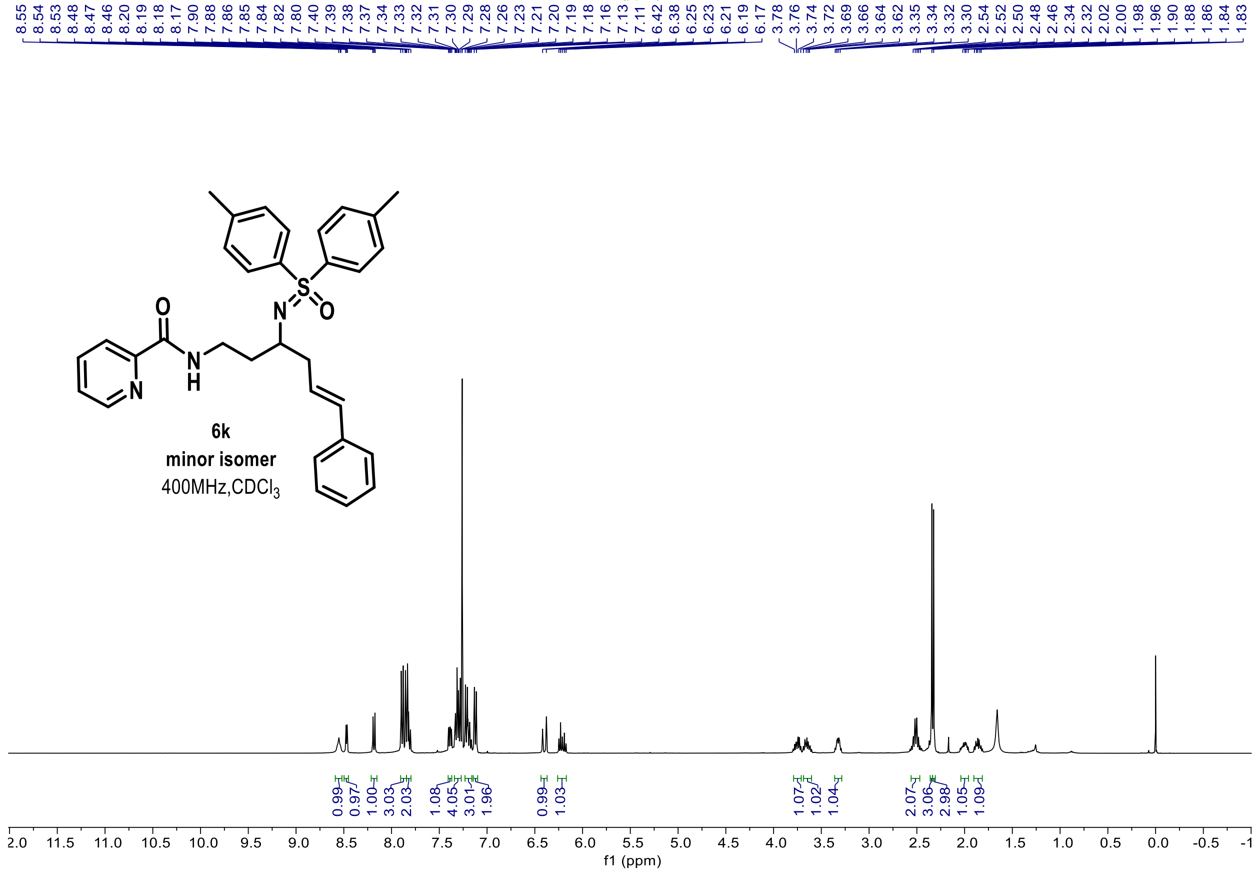
8.55
8.54
8.53
8.48
8.47
8.46
8.20
8.19
8.18
8.17
7.90
7.88
7.86
7.85
7.84
7.82
7.80
7.40
7.39
7.38
7.37
7.34
7.33
7.32
7.31
7.30
7.29
7.28
7.26
7.23
7.21
7.20
7.19
7.18
7.16
7.13
7.11
6.42
6.38
6.25
6.23
6.21
6.19
6.17
3.78
3.76
3.74
3.72
3.69
3.66
3.64
3.62
3.35
3.34
3.32
3.30
2.54
2.52
2.50
2.48
2.46
2.34
2.32
2.02
2.00
1.98
1.96
1.90
1.88
1.86
1.84
1.83
O
N
H
N
N
S
O
6k
minor isomer
400MHz,CDCl3
0.99
0.97
1.00
3.03
2.03
1.08
4.05
3.01
1.96
0.99
1.03
1.07
1.02
1.04
2.07
3.06
2.98
1.05
1.09
12.0
11.5
11.0
10.5
10.0
9.5
9.0
8.5
8.0
7.5
7.0
6.5
6.0
5.5
5.0
4.5
4.0
3.5
3.0
2.5
2.0
1.5
1.0
0.5
0.0
-0.5
-1
f1 (ppm)

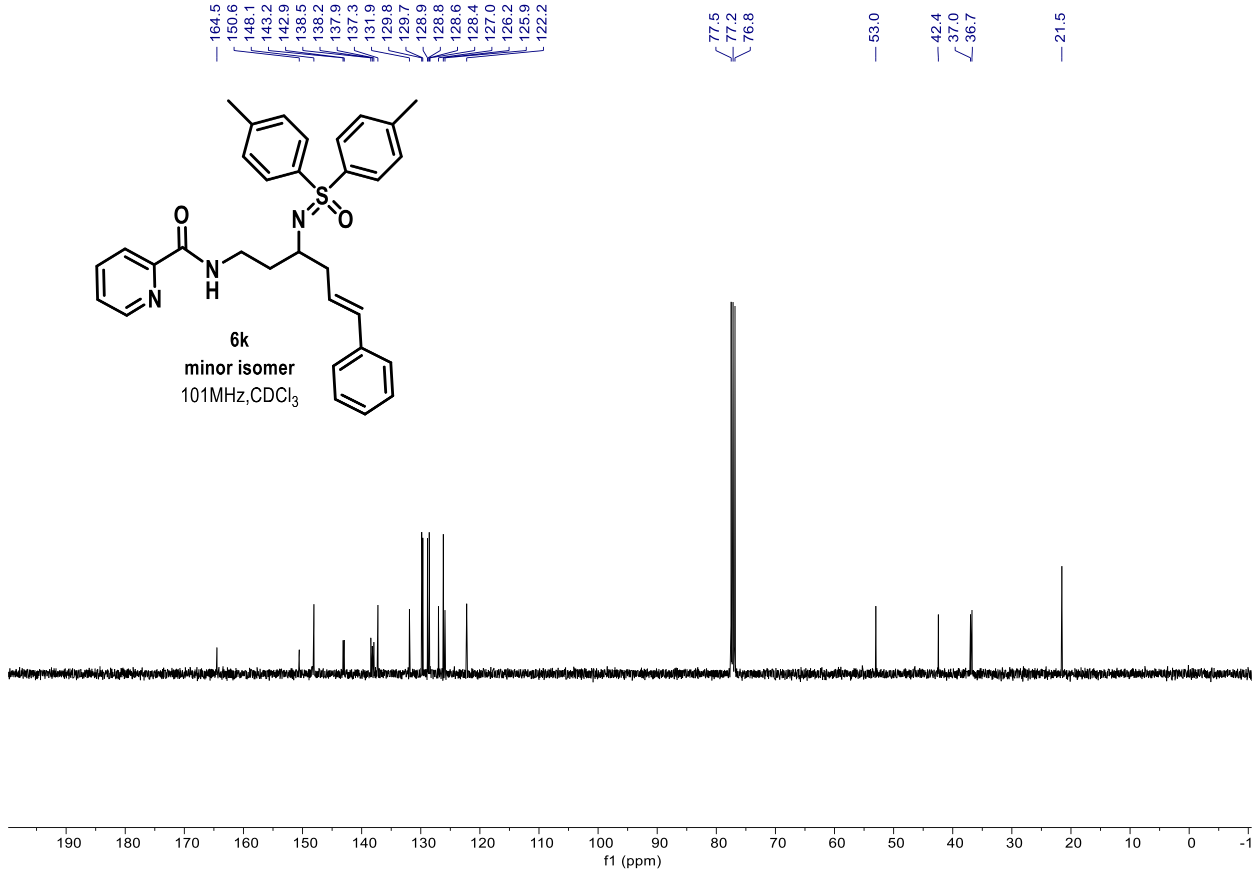

164.5
150.6
148.1
143.2
142.9
138.5
138.2
137.9
137.3
131.9
129.8
129.7
128.9
128.8
128.6
128.4
127.0
126.2
125.9
122.2
77.5
77.2
76.8
53.0
42.4
37.0
36.7
21.5
6k
minor isomer
101MHz,CDCl3
f1 (ppm)

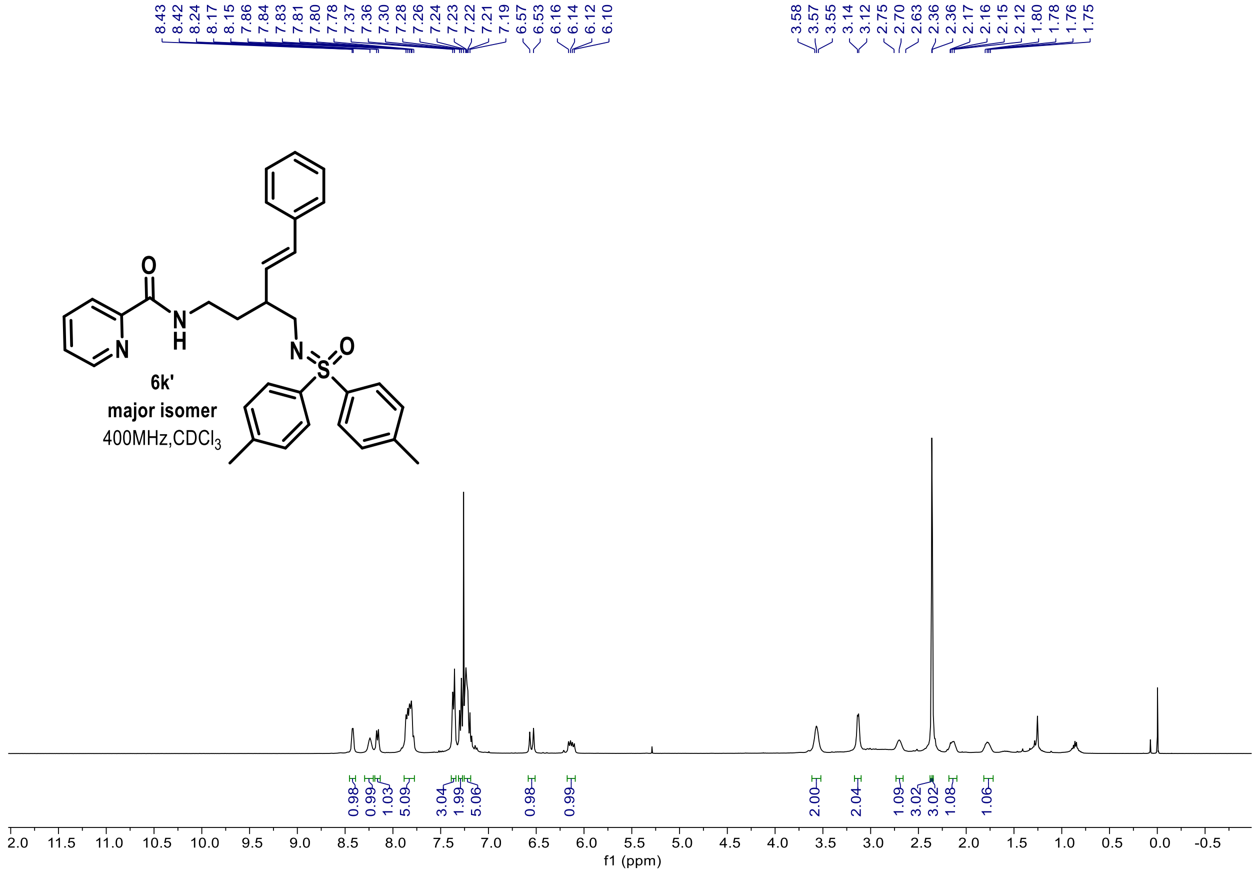

8.43
8.42
8.24
8.17
8.15
7.86
7.84
7.83
7.81
7.80
7.78
7.37
7.36
7.30
7.28
7.26
7.24
7.23
7.22
7.21
7.19
6.57
6.53
6.16
6.14
6.12
6.10
3.58
3.57
3.55
3.14
3.12
2.75
2.70
2.63
2.36
2.36
2.17
2.16
2.15
2.12
1.80
1.78
1.76
1.75
6k'
major isomer
400MHz,CDCl3
0.98
0.99
1.03
5.09
3.04
1.99
5.06
0.98
0.99
2.00
2.04
1.09
3.02
3.02
1.08
1.06
f1 (ppm)

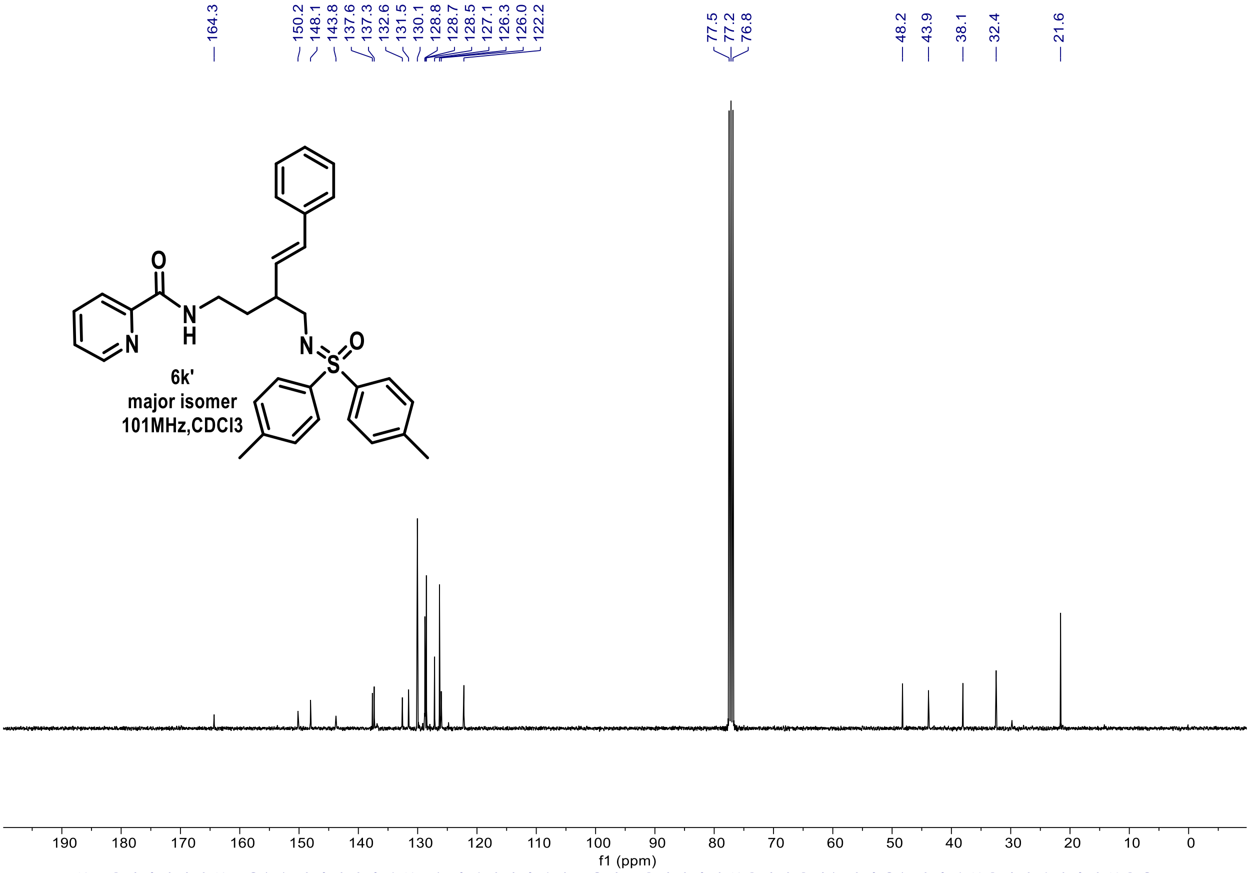
6k'
major isomer
101MHz,CDCl3
f1 (ppm)

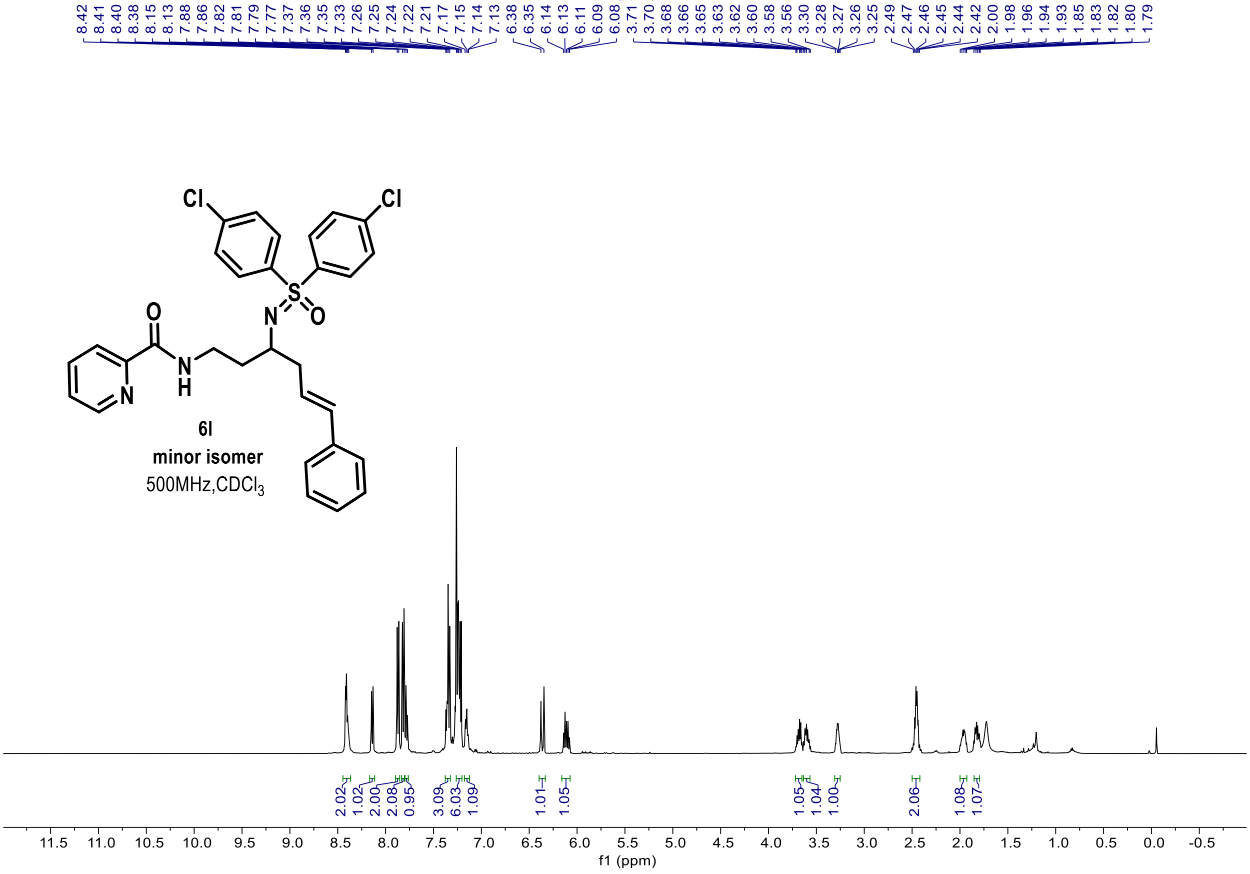
6l
minor isomer
500MHz,CDCl3
f1 (ppm)

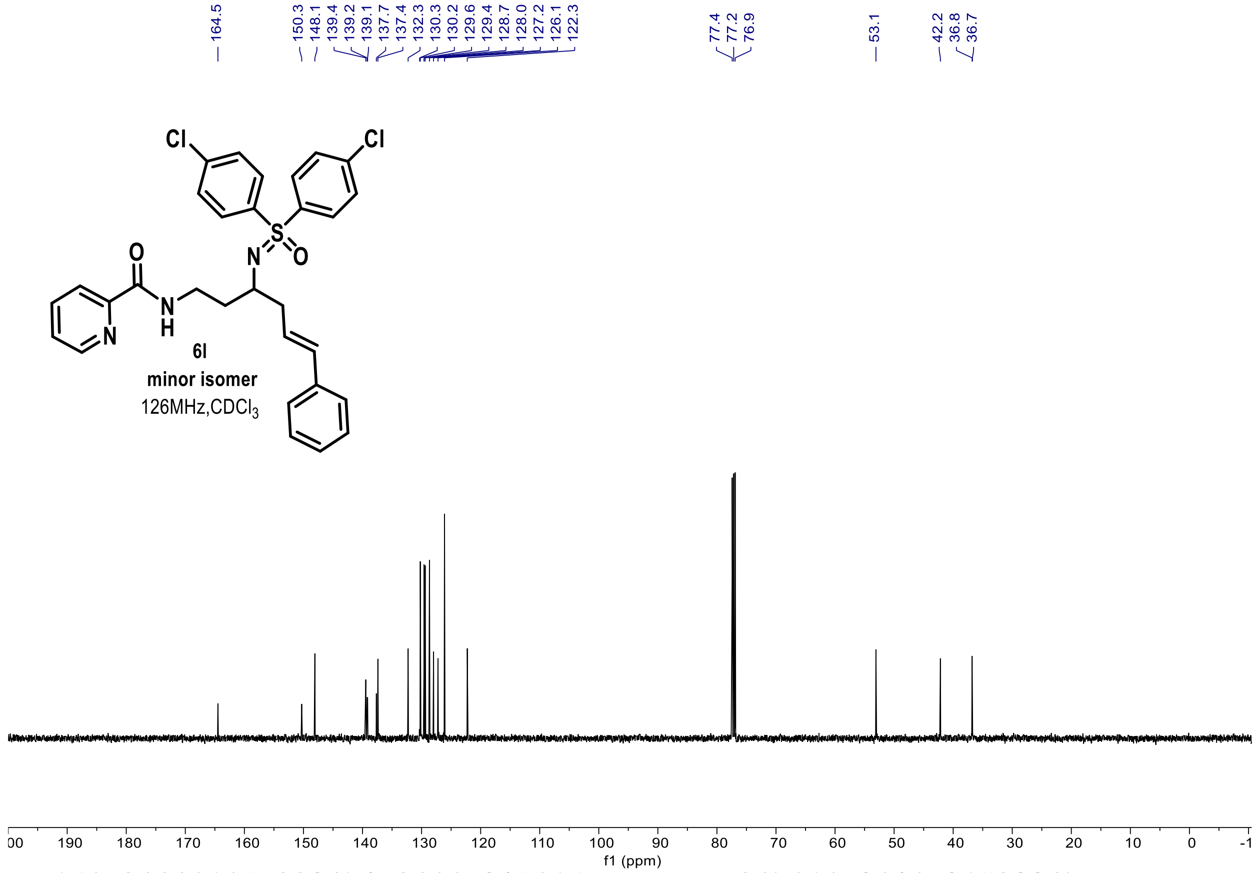

164.5
150.3
148.1
139.4
139.2
139.1
137.7
137.4
132.3
130.3
130.2
129.6
129.4
128.7
128.0
127.2
126.1
122.3
77.4
77.2
76.9
53.1
42.2
36.8
36.7
6l
minor isomer
126MHz,CDCl3
f1 (ppm)

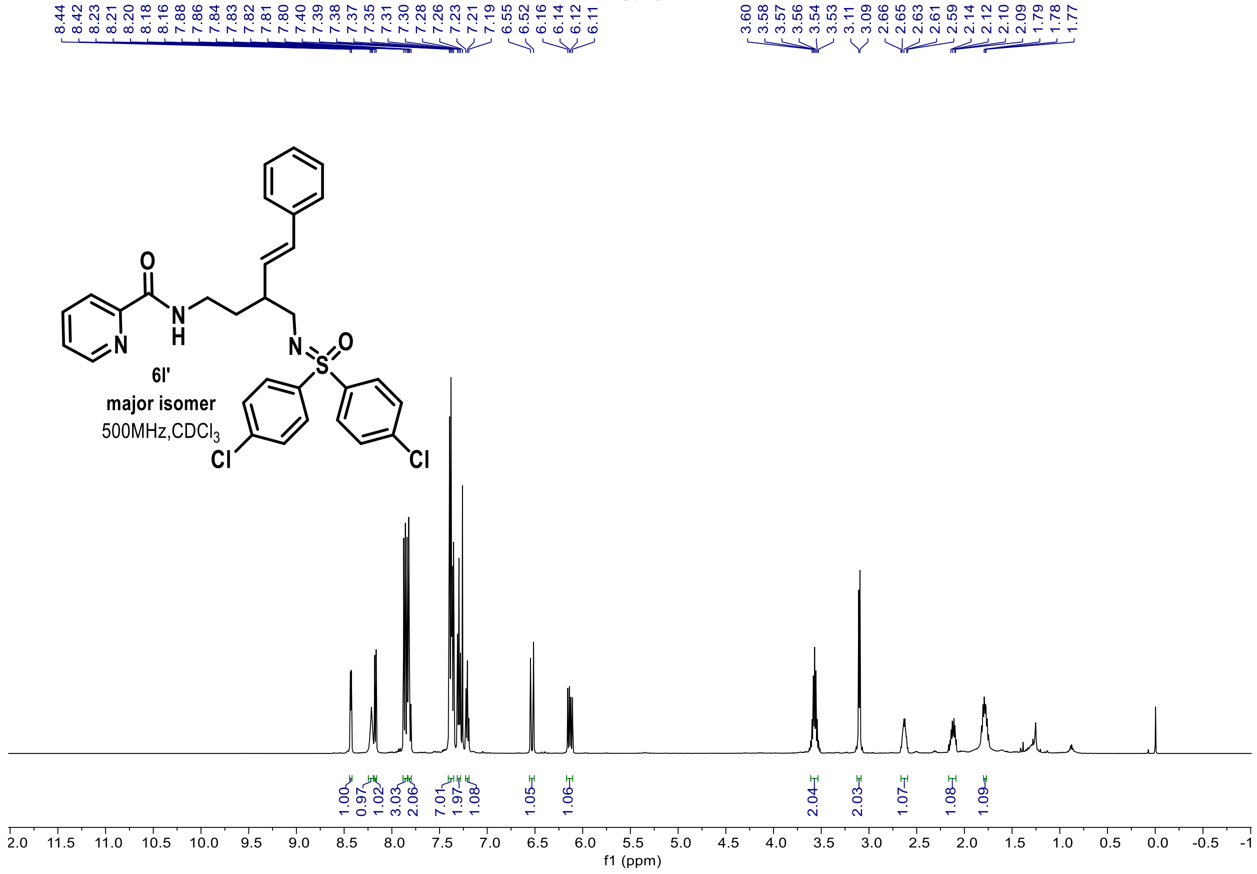

8.44
8.42
8.23
8.21
8.20
8.18
8.16
7.88
7.86
7.84
7.83
7.82
7.81
7.80
7.40
7.39
7.38
7.37
7.35
7.31
7.30
7.28
7.26
7.23
7.21
7.19
6.55
6.52
6.16
6.14
6.12
6.11
3.60
3.58
3.57
3.56
3.54
3.53
3.11
3.09
2.66
2.65
2.63
2.61
2.59
2.14
2.12
2.10
2.09
1.79
1.78
1.77
6l'
major isomer
500MHz,CDCl3
1.00
0.97
1.02
3.03
2.06
7.01
1.97
1.08
1.05
1.06
2.04
2.03
1.07
1.08
1.09
f1 (ppm)

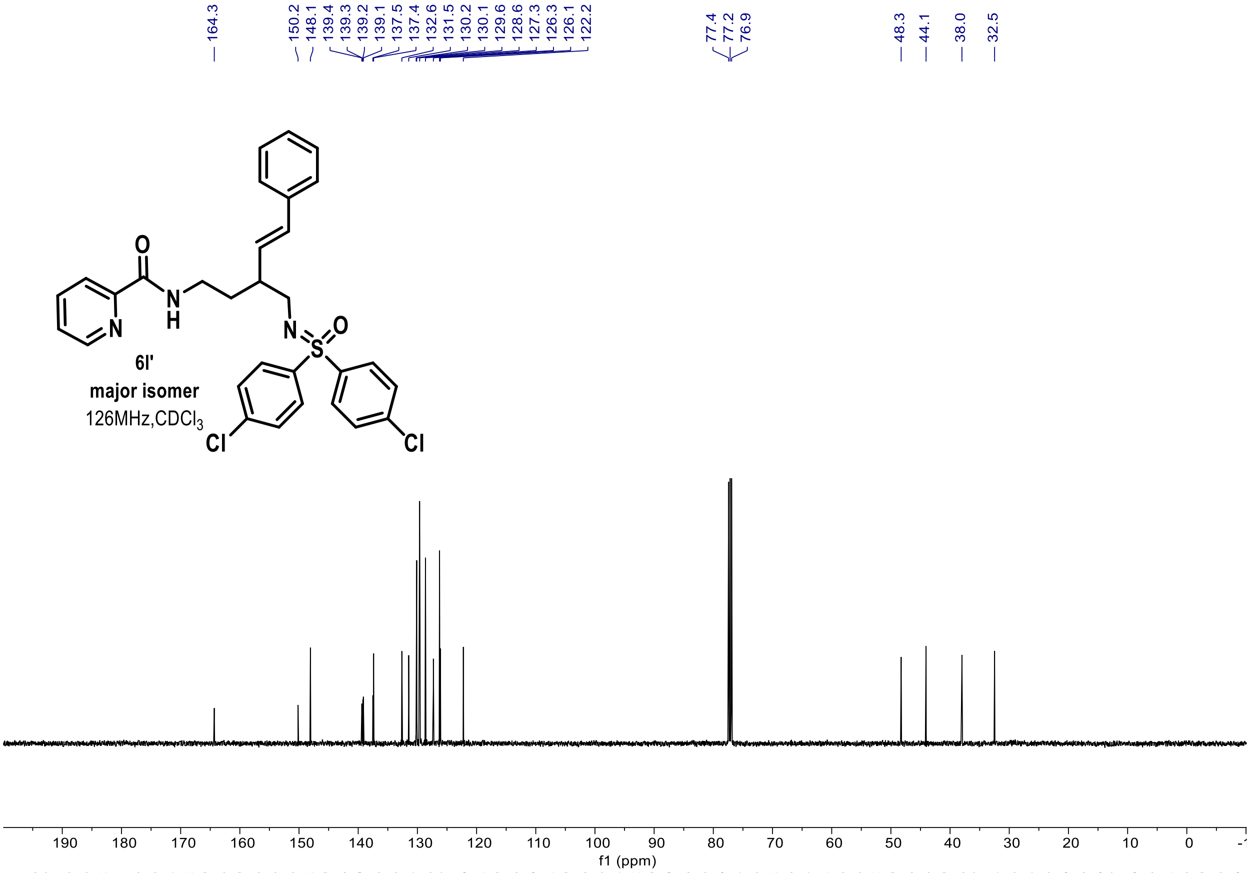
6l'
major isomer
126MHz,CDCl3
f1 (ppm)

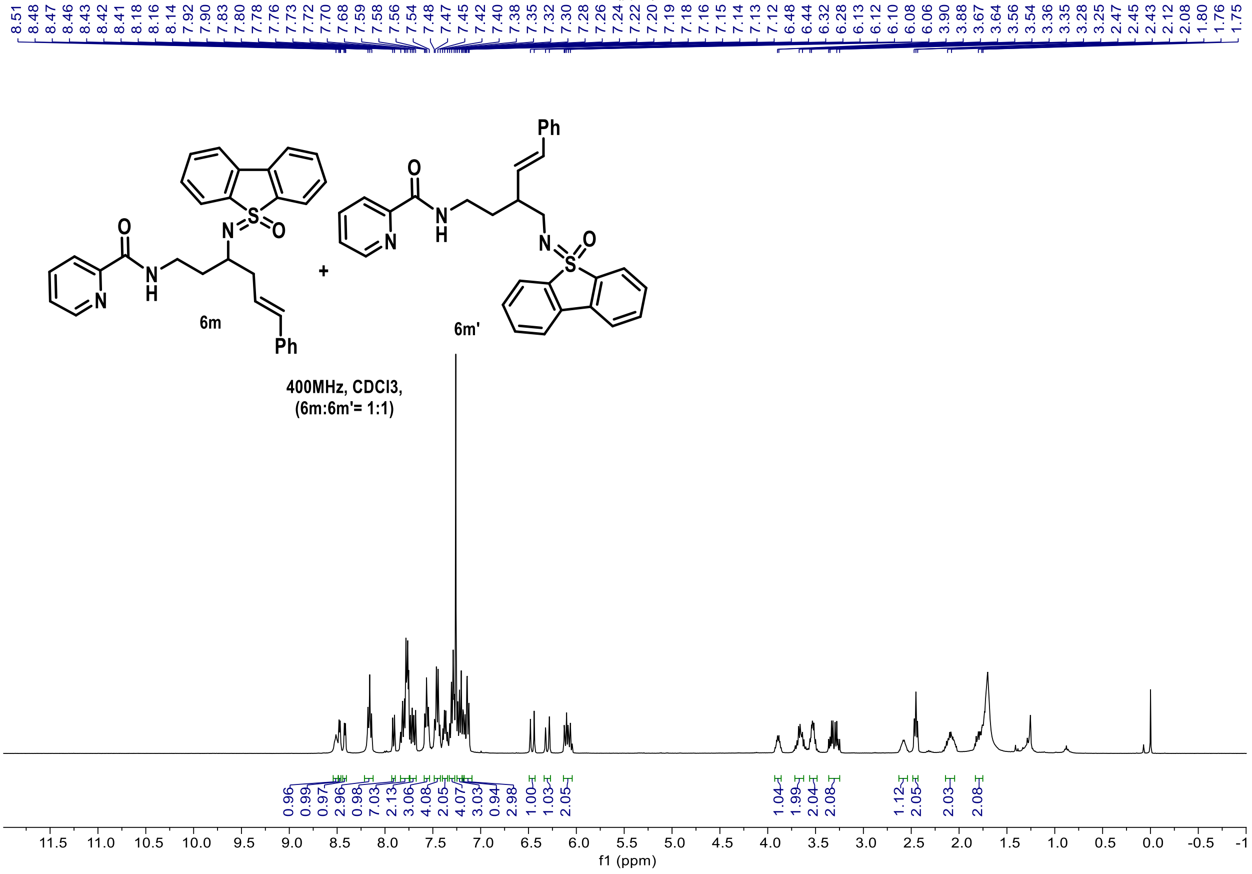
6m
6m'
400MHz, CDCl3,
(6m:6m'= 1:1)
f1 (ppm)

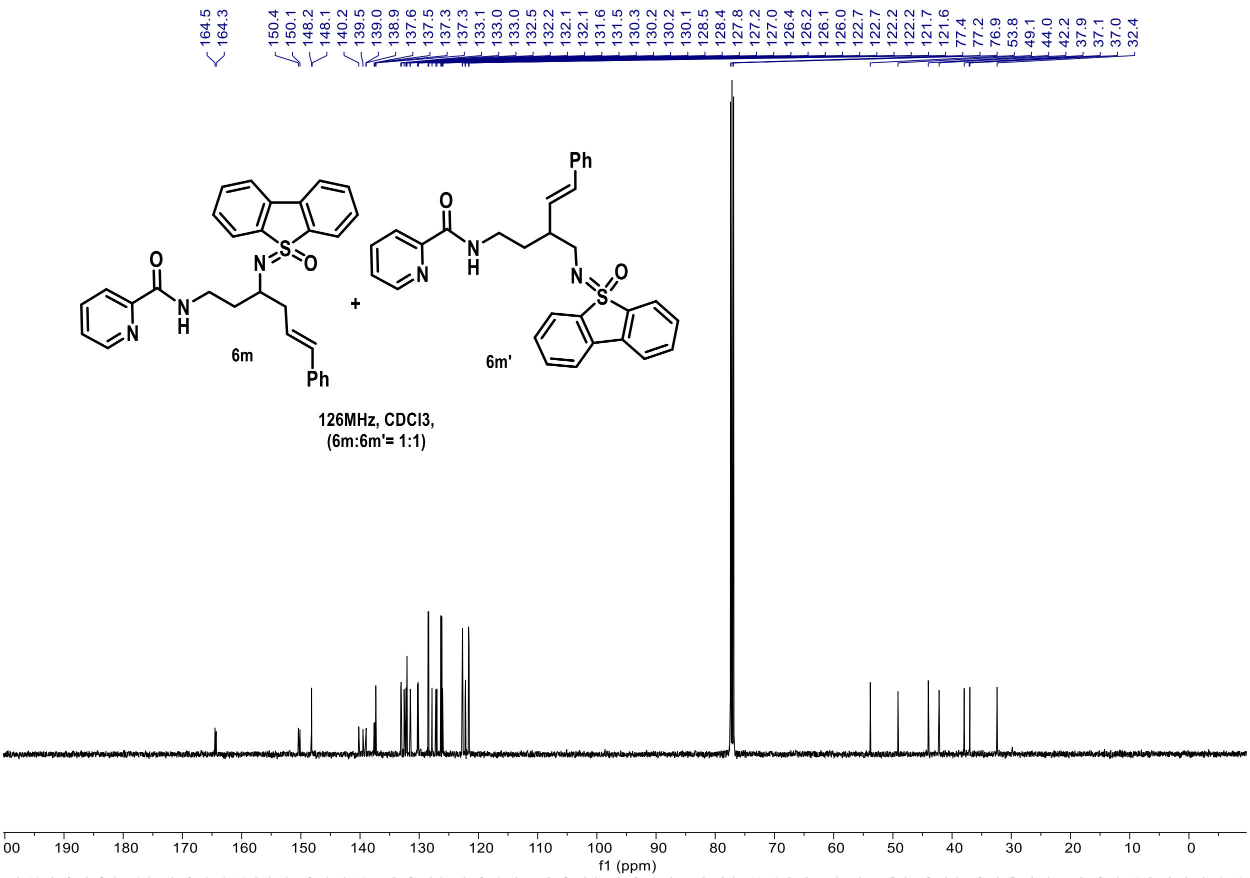
164.5
164.3
150.4
150.1
148.2
148.1
140.2
139.5
139.0
138.9
137.6
137.5
137.3
137.3
133.1
133.0
133.0
132.5
132.2
132.1
132.1
131.6
131.5
130.3
130.2
130.1
130.1
128.5
128.4
127.8
127.2
127.0
126.4
126.2
126.1
126.0
122.7
122.7
122.2
122.2
121.7
121.6
77.4
77.2
76.9
53.8
49.1
44.0
42.2
37.9
37.1
37.0
32.4
Ph
O
N
S
O
N
H
N
+
6m
Ph
6m'
126MHz, CDCl3,
(6m:6m'= 1:1)
f1 (ppm)

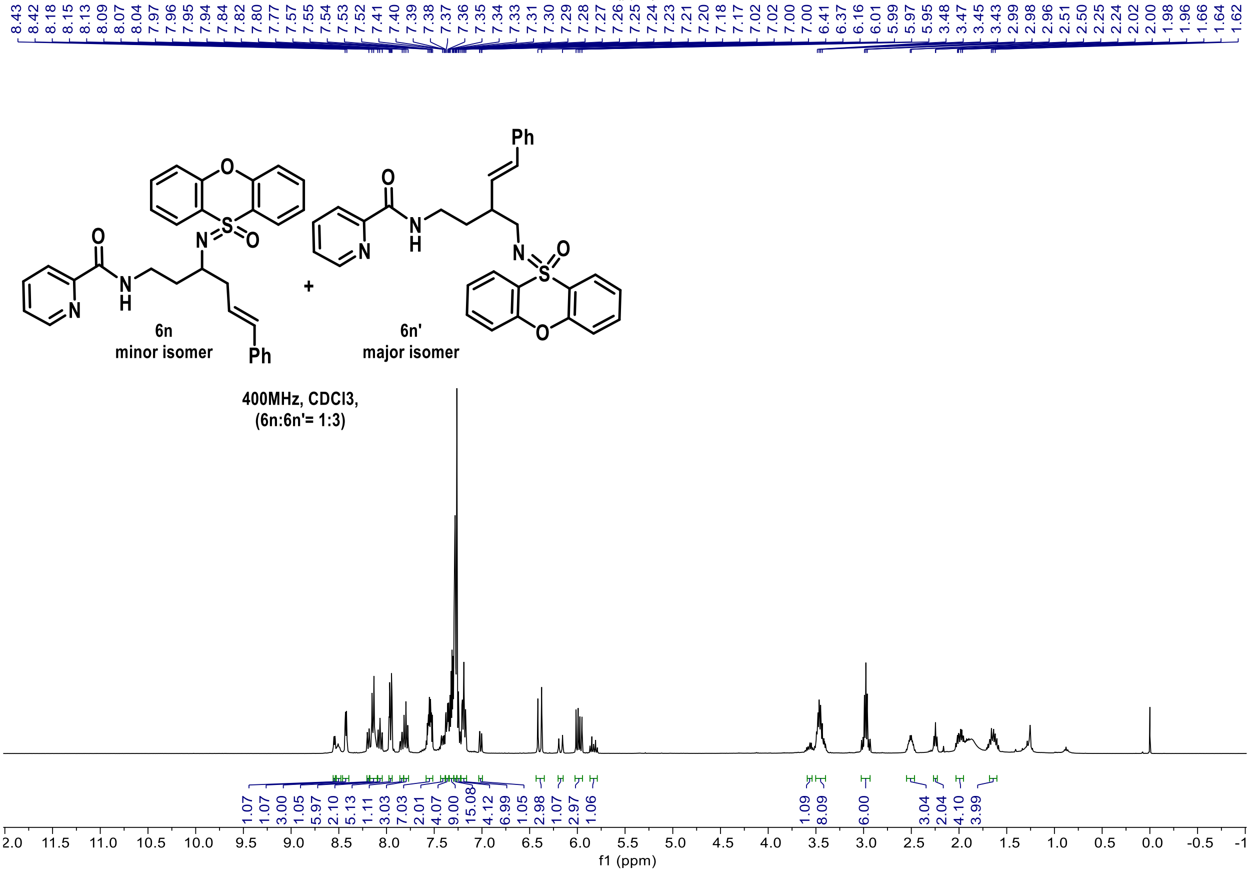
8.43
8.42
8.18
8.15
8.13
8.09
8.07
8.04
7.97
7.96
7.95
7.94
7.84
7.82
7.80
7.77
7.57
7.55
7.54
7.53
7.52
7.41
7.40
7.39
7.38
7.37
7.36
7.35
7.34
7.33
7.31
7.30
7.29
7.28
7.27
7.26
7.25
7.24
7.23
7.21
7.20
7.18
7.17
7.02
7.02
7.00
7.00
6.41
6.37
6.16
6.01
5.99
5.97
5.95
3.48
3.47
3.45
3.43
2.99
2.98
2.96
2.51
2.50
2.25
2.24
2.02
2.00
1.98
1.96
1.66
1.64
1.62
Ph
O
+
6n
minor isomer
Ph
6n'
major isomer
400MHz, CDCl3,
(6n:6n'= 1:3)
1.07
1.07
3.00
1.05
5.97
2.10
5.13
1.11
3.03
7.03
2.01
4.07
9.00
15.08
4.12
6.99
1.05
2.98
1.07
2.97
1.06
1.09
8.09
6.00
3.04
2.04
4.10
3.99
f1 (ppm)

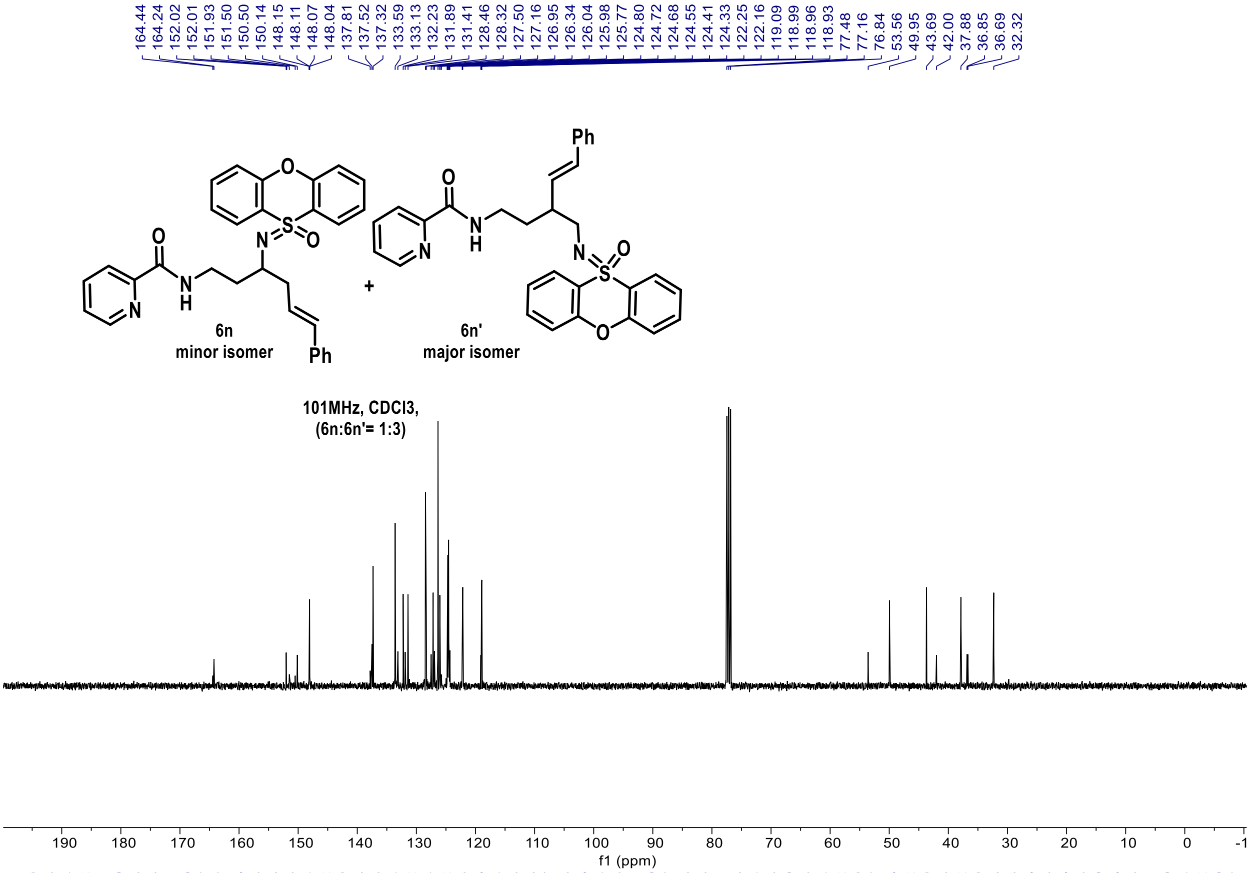
164.44
164.24
152.02
152.01
151.93
151.50
150.50
150.14
148.15
148.11
148.07
148.04
137.81
137.52
137.32
133.59
133.13
132.23
131.89
131.41
128.46
128.32
127.50
127.16
126.95
126.34
126.04
125.98
125.77
124.80
124.72
124.68
124.55
124.41
124.33
122.25
122.16
119.09
118.99
118.96
118.93
77.48
77.16
76.84
53.56
49.95
43.69
42.00
37.88
36.85
36.69
32.32
6n
minor isomer
6n'
major isomer
101MHz, CDCl3,
(6n:6n'= 1:3)
f1 (ppm)

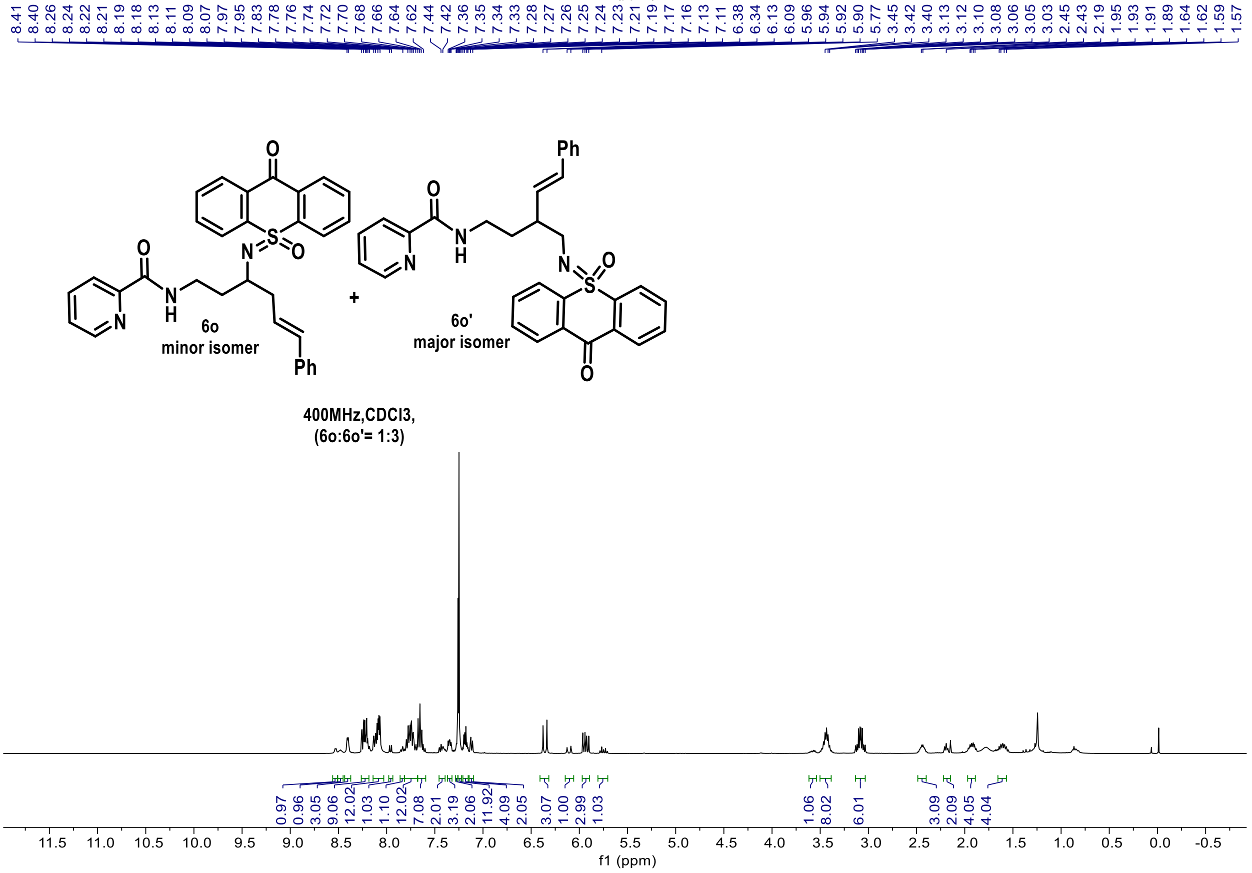
8.41
8.40
8.26
8.24
8.22
8.21
8.19
8.18
8.13
8.11
8.09
8.07
7.97
7.95
7.83
7.78
7.76
7.74
7.72
7.70
7.68
7.66
7.64
7.62
7.44
7.42
7.36
7.35
7.34
7.33
7.28
7.27
7.26
7.25
7.24
7.23
7.21
7.19
7.17
7.16
7.13
7.11
6.38
6.34
6.13
6.09
5.96
5.94
5.92
5.90
5.77
3.45
3.42
3.40
3.13
3.12
3.10
3.08
3.06
3.05
3.03
2.45
2.43
2.19
1.95
1.93
1.91
1.89
1.64
1.62
1.59
1.57
6o
minor isomer
6o'
major isomer
400MHz,CDCl3,
(6o:6o'= 1:3)
0.97
0.96
3.05
9.06
12.02
1.03
1.10
12.02
7.08
2.01
3.19
2.06
11.92
4.09
2.05
3.07
1.00
2.99
1.03
1.06
8.02
6.01
3.09
2.09
4.05
4.04
f1 (ppm)

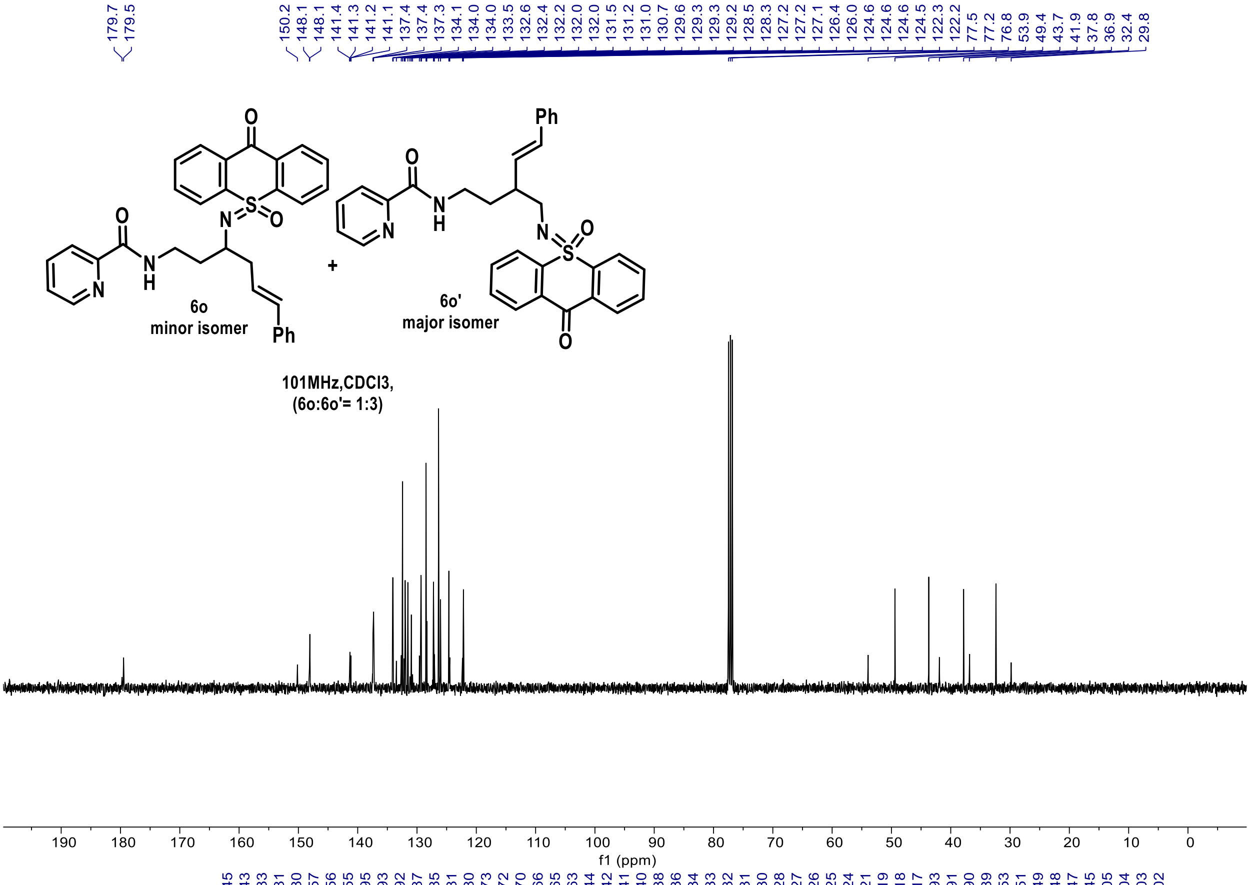
179.7
179.5
150.2
148.1
148.1
141.4
141.3
141.2
141.1
137.4
137.4
137.3
134.1
134.0
134.0
133.5
132.6
132.4
132.2
132.0
132.0
131.5
131.2
131.0
130.7
129.6
129.3
129.3
129.2
128.5
128.3
127.2
127.2
127.1
126.4
126.0
124.6
124.6
124.6
124.5
122.3
122.2
77.5
77.2
76.8
53.9
49.4
43.7
41.9
37.8
36.9
32.4
29.8
6o
minor isomer
6o'
major isomer
101MHz,CDCl3,
(6o:6o'= 1:3)
f1 (ppm)

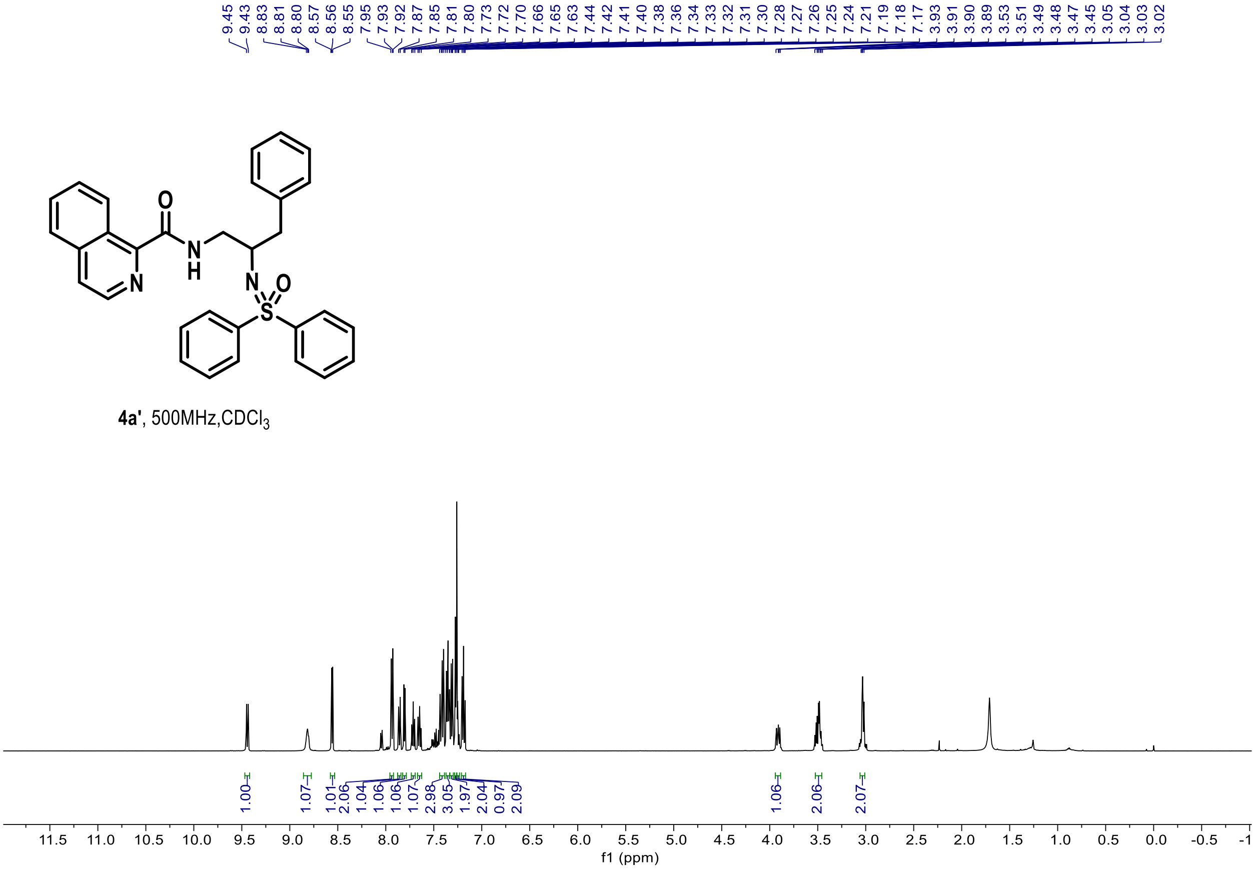
9.45
9.43
8.83
8.81
8.80
8.57
8.56
8.55
7.95
7.93
7.92
7.87
7.85
7.81
7.80
7.73
7.72
7.70
7.66
7.65
7.63
7.44
7.42
7.41
7.40
7.38
7.36
7.34
7.33
7.32
7.31
7.30
7.28
7.27
7.26
7.25
7.24
7.21
7.19
7.18
7.17
3.93
3.91
3.90
3.89
3.53
3.51
3.49
3.48
3.47
3.45
3.05
3.04
3.03
3.02
4a', 500MHz,CDCl3
1.00
1.07
1.01
2.06
1.04
1.06
1.06
1.07
2.98
3.05
1.97
2.04
0.97
2.09
1.06
2.06
2.07
f1 (ppm)

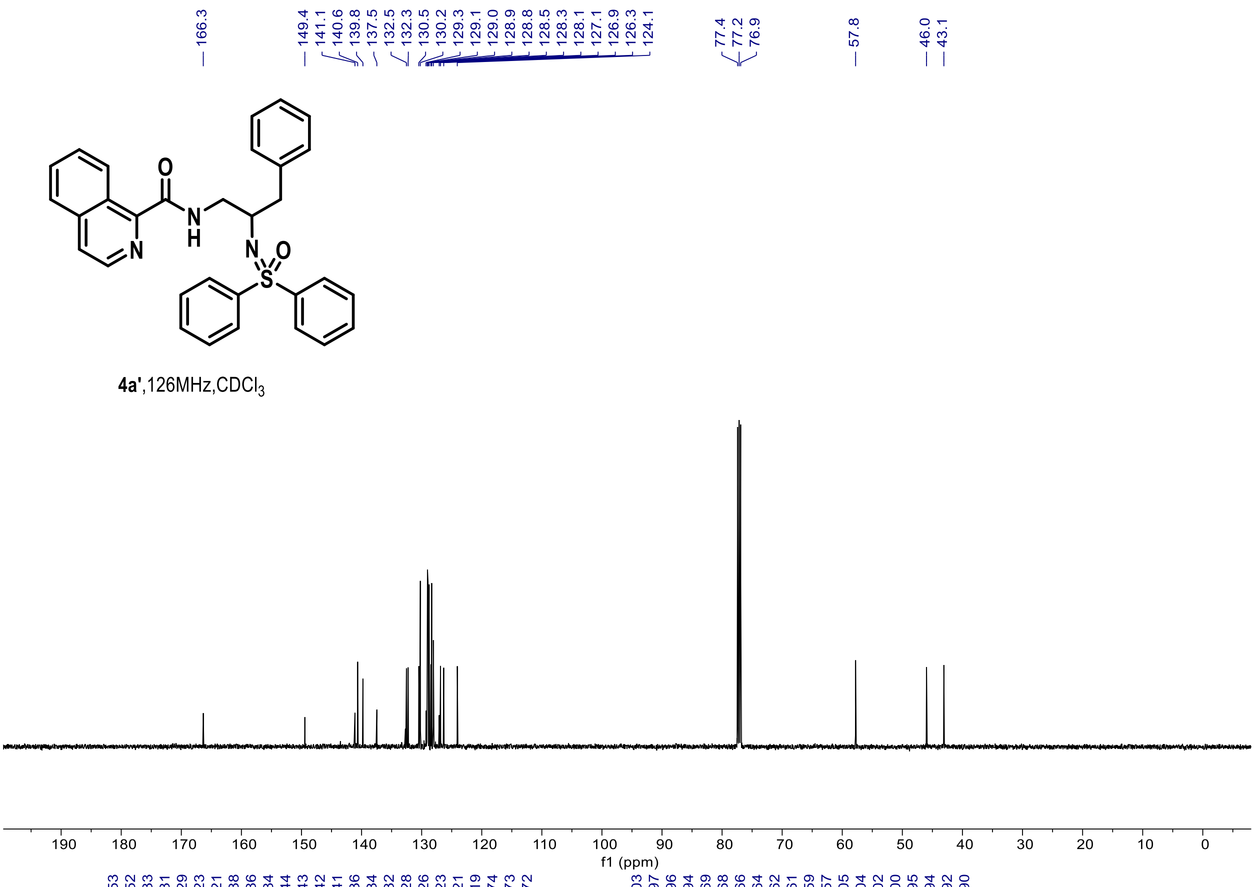
166.3
149.4
141.1
140.6
139.8
137.5
132.5
132.3
130.5
130.2
129.3
129.1
129.0
128.9
128.8
128.5
128.3
128.1
127.1
126.9
126.3
124.1
77.4
77.2
76.9
57.8
46.0
43.1
O
N
H
N
N
S
O
4a',126MHz,CDCl3
190 180 170 160 150 140 130 120 110 100 90 80 70 60 50 40 30 20 10 0
f1 (ppm)

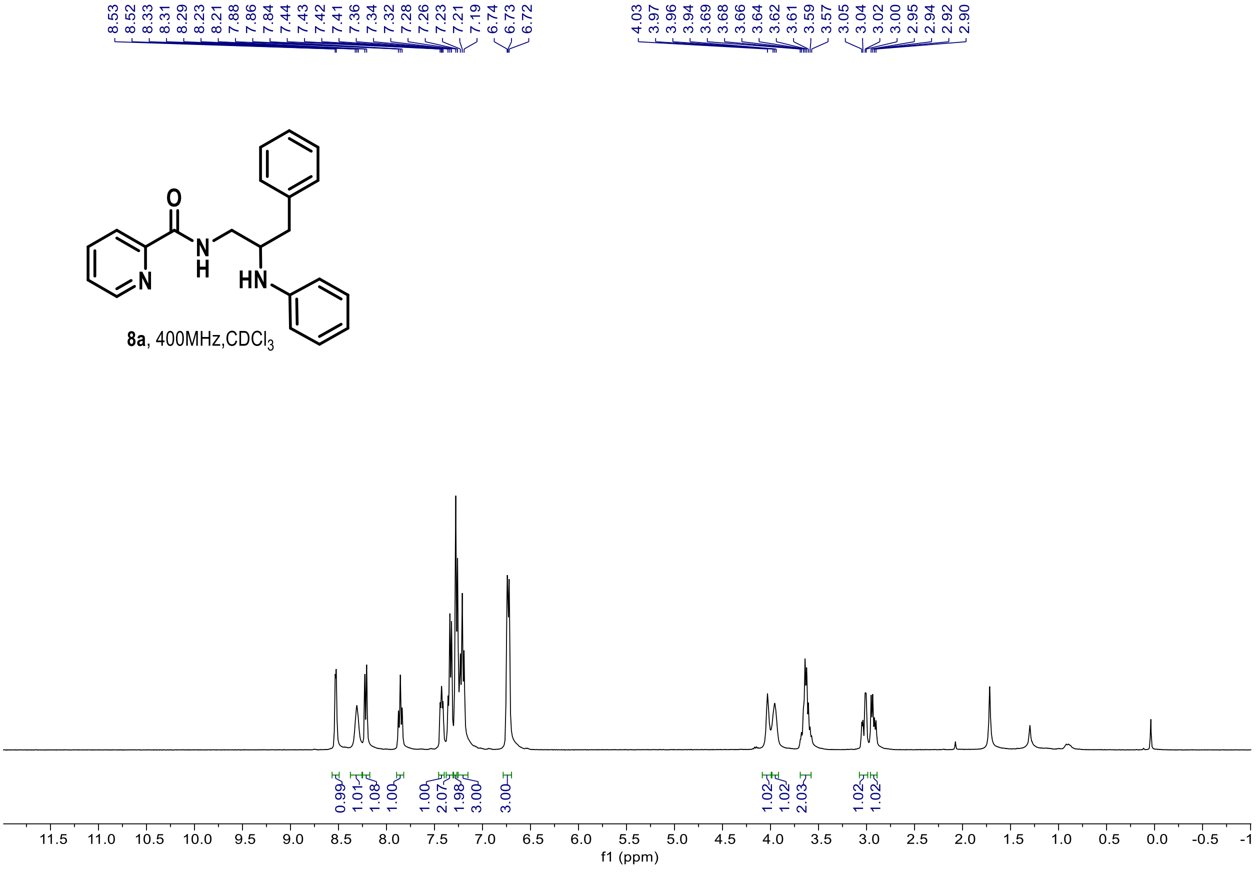
8.53
8.52
8.33
8.31
8.29
8.23
8.21
7.88
7.86
7.84
7.44
7.43
7.42
7.41
7.36
7.34
7.32
7.28
7.26
7.23
7.21
7.19
6.74
6.73
6.72
4.03
3.97
3.96
3.94
3.69
3.68
3.66
3.64
3.62
3.61
3.59
3.57
3.05
3.04
3.02
3.00
2.95
2.94
2.92
2.90
O
N
H
N
HN
8a, 400MHz,CDCl3
0.99
1.01
1.08
1.00
1.00
2.07
1.98
3.00
3.00
1.02
1.02
2.03
1.02
1.02
11.5 11.0 10.5 10.0 9.5 9.0 8.5 8.0 7.5 7.0 6.5 6.0 5.5 5.0 4.5 4.0 3.5 3.0 2.5 2.0 1.5 1.0 0.5 0.0 -0.5 -1
f1 (ppm)

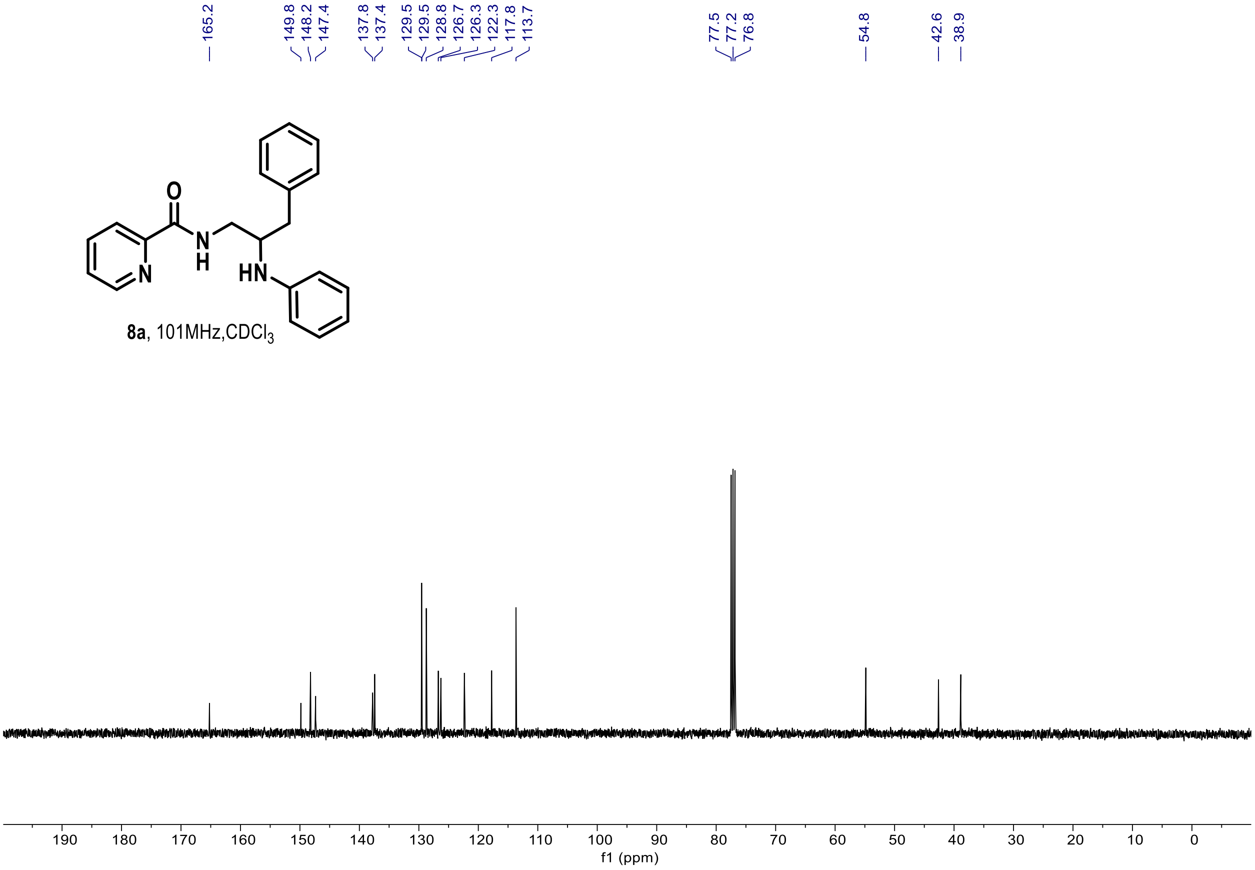
165.2
149.8
148.2
147.4
137.8
137.4
129.5
129.5
128.8
126.7
126.3
122.3
117.8
113.7
77.5
77.2
76.8
54.8
42.6
38.9
O
N
N
H
HN
8a, 101MHz,CDCl3
f1 (ppm)

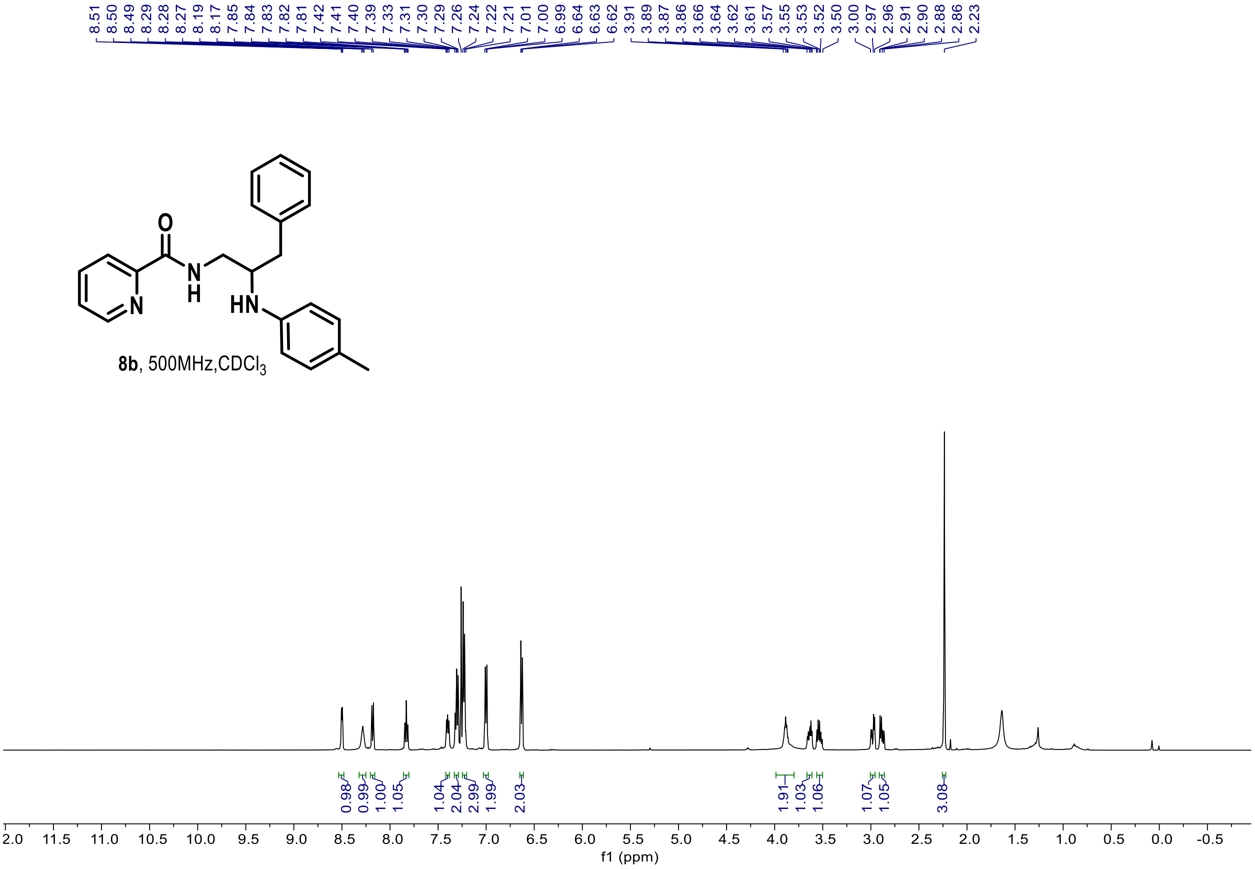
8.51 8.50 8.49 8.29 8.28 8.27 8.19 8.17 7.85 7.84 7.83 7.82 7.81 7.42 7.41 7.40 7.39 7.33 7.31 7.30 7.29 7.26 7.24 7.22 7.21 7.01 7.00 6.99 6.64 6.63 6.62 3.91 3.89 3.87 3.86 3.66 3.64 3.62 3.61 3.57 3.55 3.53 3.52 3.50 3.00 2.97 2.96 2.91 2.90 2.88 2.86 2.23
O
N
N
H
HN
8b, 500MHz,CDCl3
0.98 0.99 1.00 1.05 1.04 2.04 2.99 1.99 2.03 1.91 1.03 1.06 1.07 1.05 3.08
f1 (ppm)

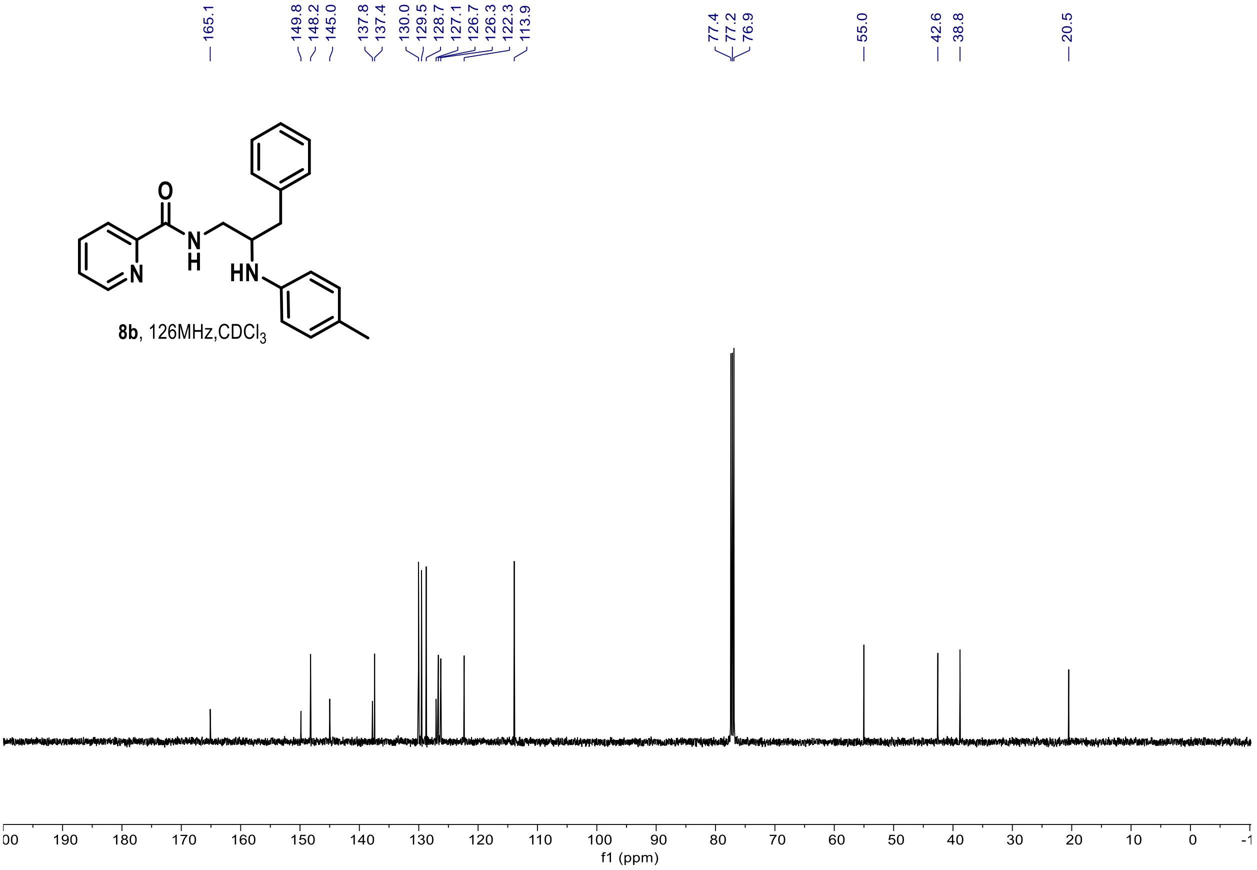
165.1
149.8
148.2
145.0
137.8
137.4
130.0
129.5
128.7
127.1
126.7
126.3
122.3
113.9
77.4
77.2
76.9
55.0
42.6
38.8
20.5
8b, 126MHz,CDCl3
f1 (ppm)

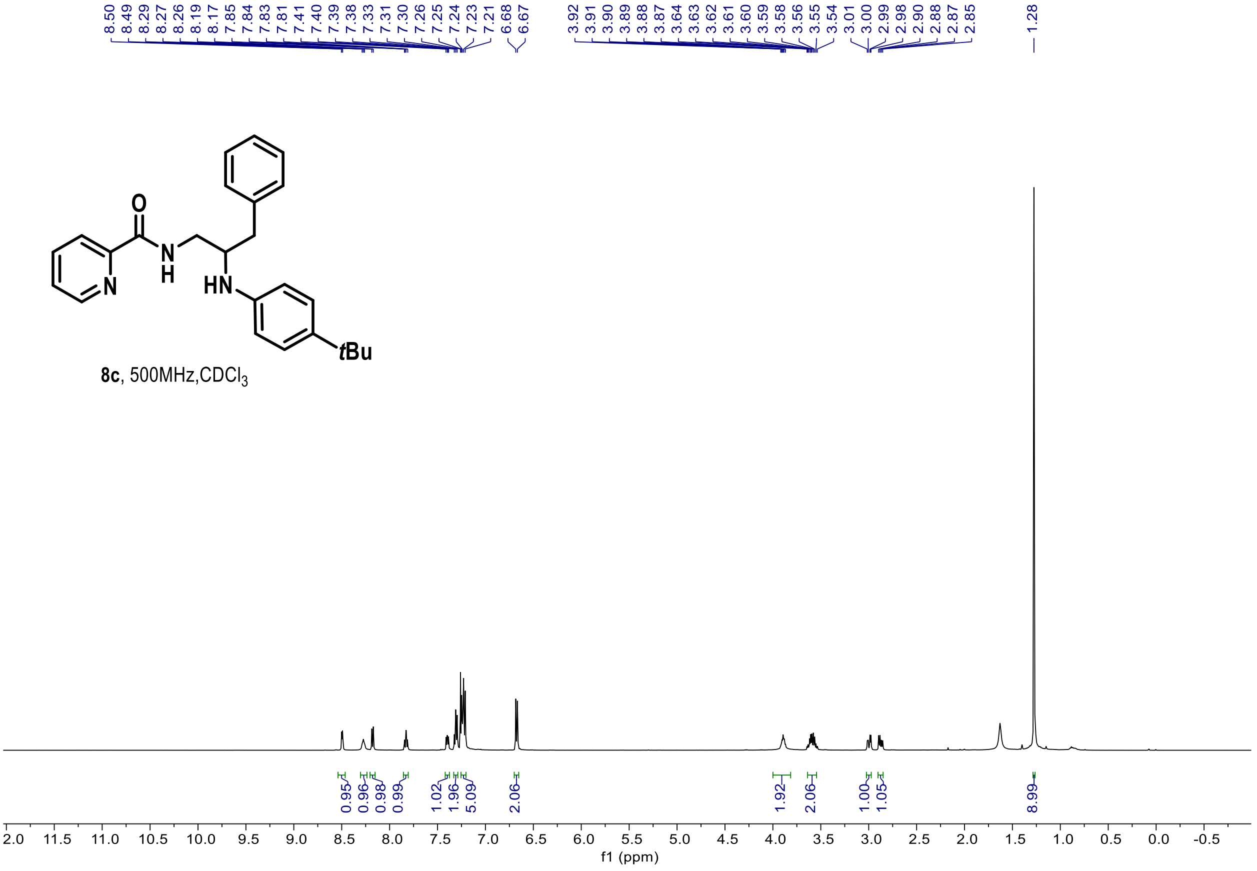
8.50 8.49 8.29 8.27 8.26 8.19 8.17 7.85 7.84 7.83 7.81 7.41 7.40 7.39 7.38 7.33 7.31 7.30 7.26 7.25 7.24 7.23 7.21 6.68 6.67
3.92 3.91 3.90 3.89 3.88 3.87 3.64 3.63 3.62 3.61 3.60 3.59 3.58 3.56 3.55 3.54 3.01 3.00 2.99 2.98 2.90 2.88 2.87 2.85
1.28
tBu
8c, 500MHz,CDCl3
0.95 0.96 0.98 0.99 1.02 1.96 5.09 2.06 1.92 2.06 1.00 1.05 8.99
f1 (ppm)

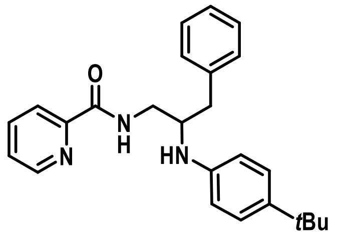

8c, 126MHz,$CDCl_3$

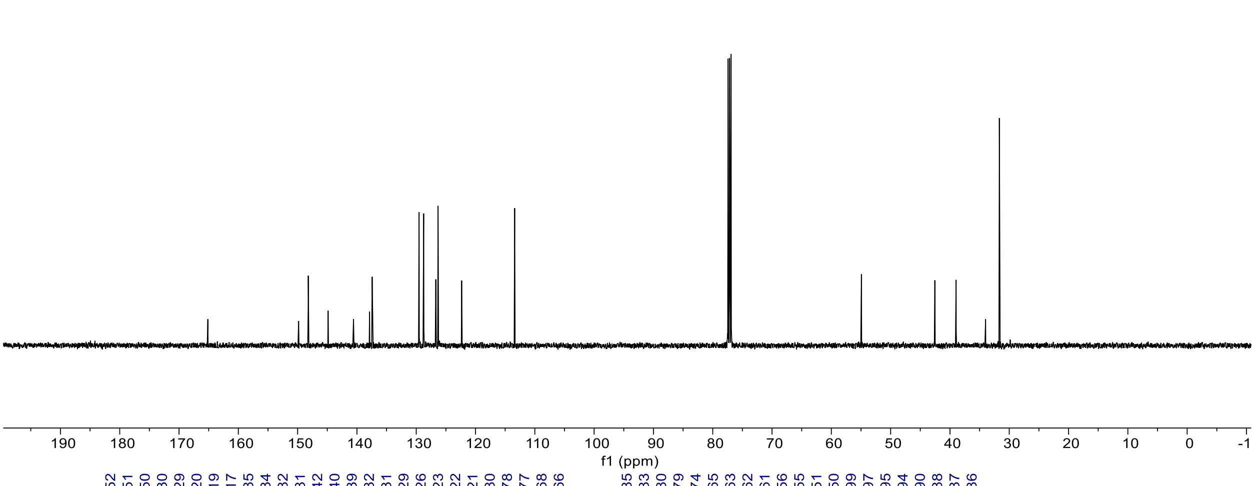

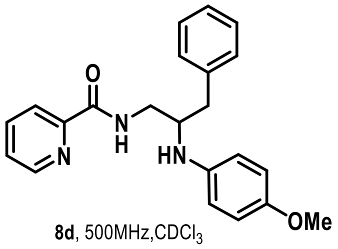

8d, 500MHz,$CDCl_3$

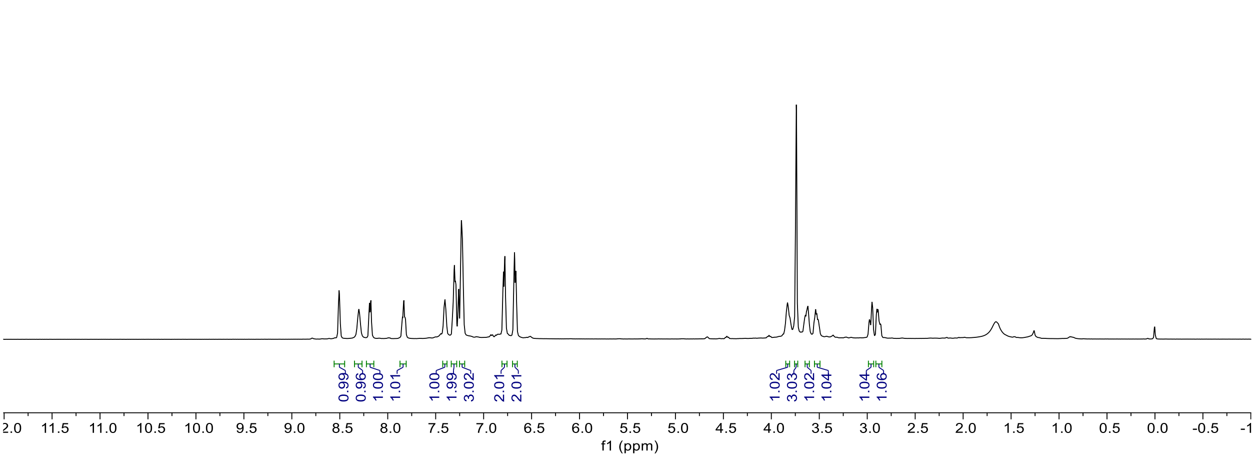

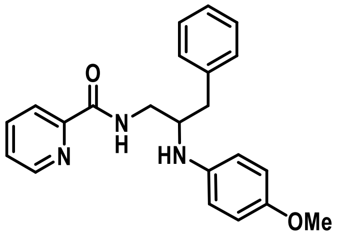

8d, 126MHz,$CDCl_3$

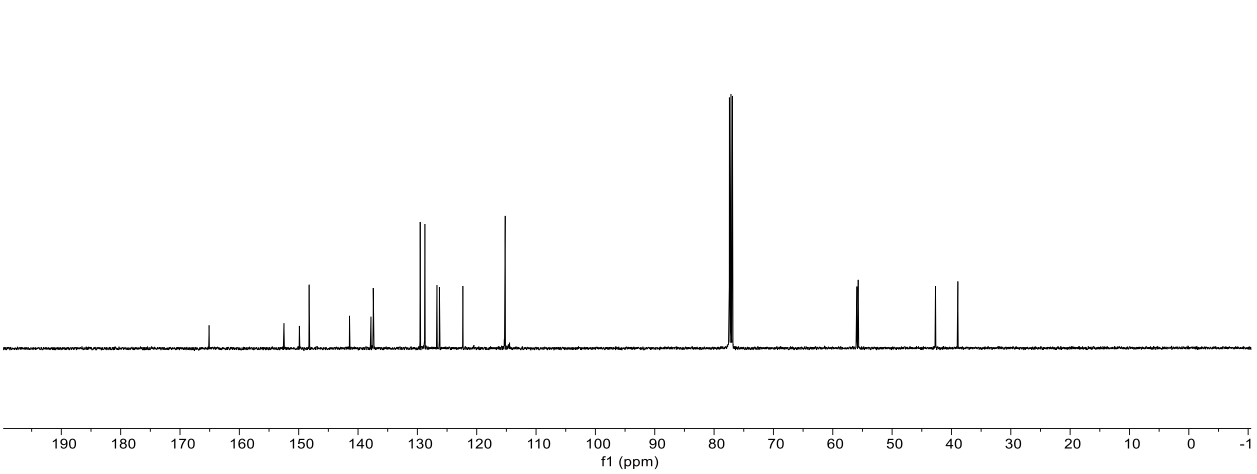

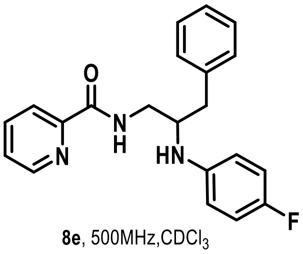

8e, 500MHz,$CDCl_3$

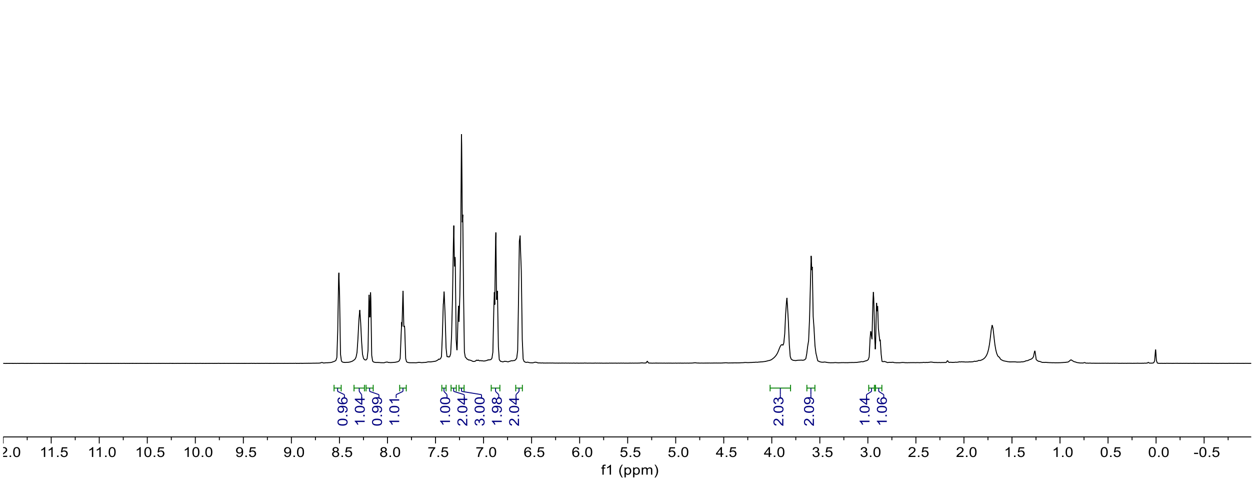

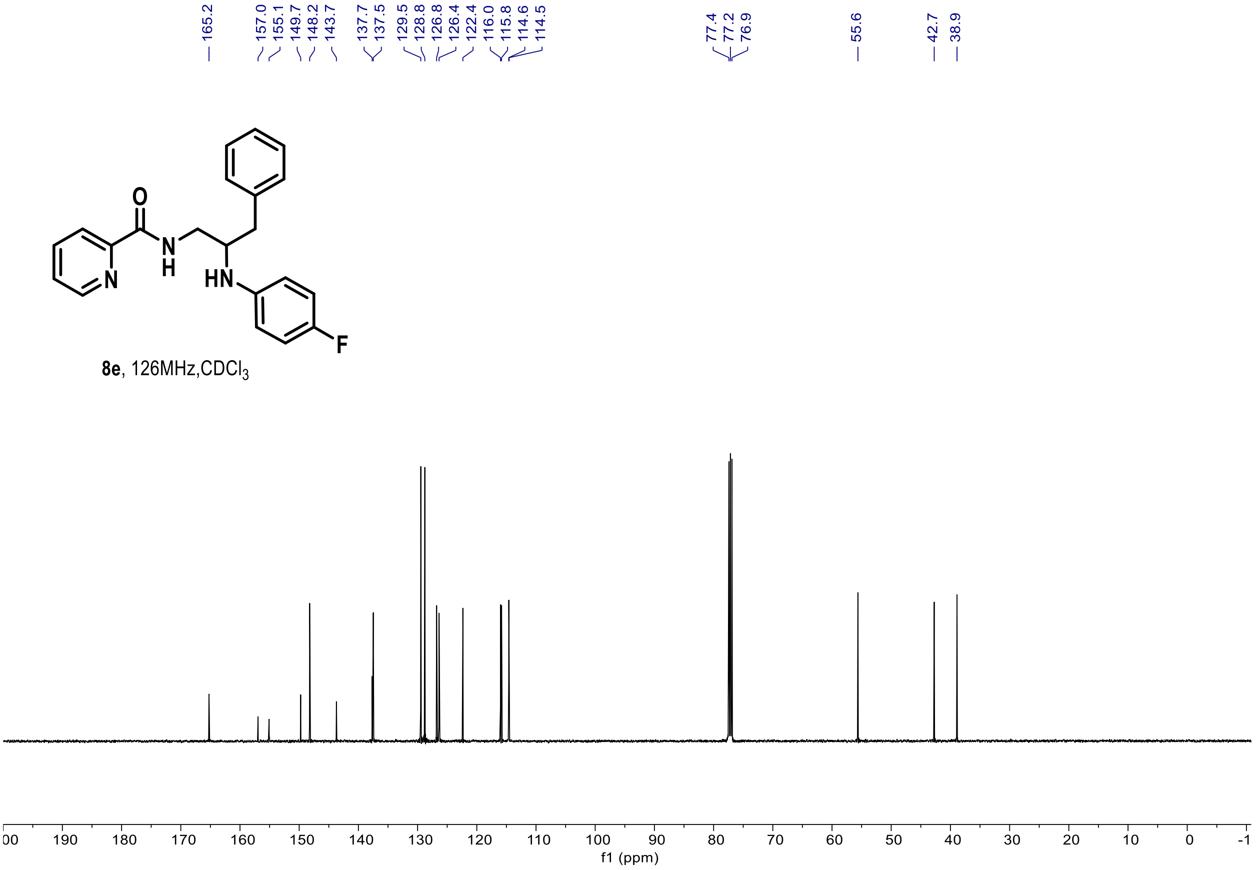

8e, 126MHz,CDCl$_3$

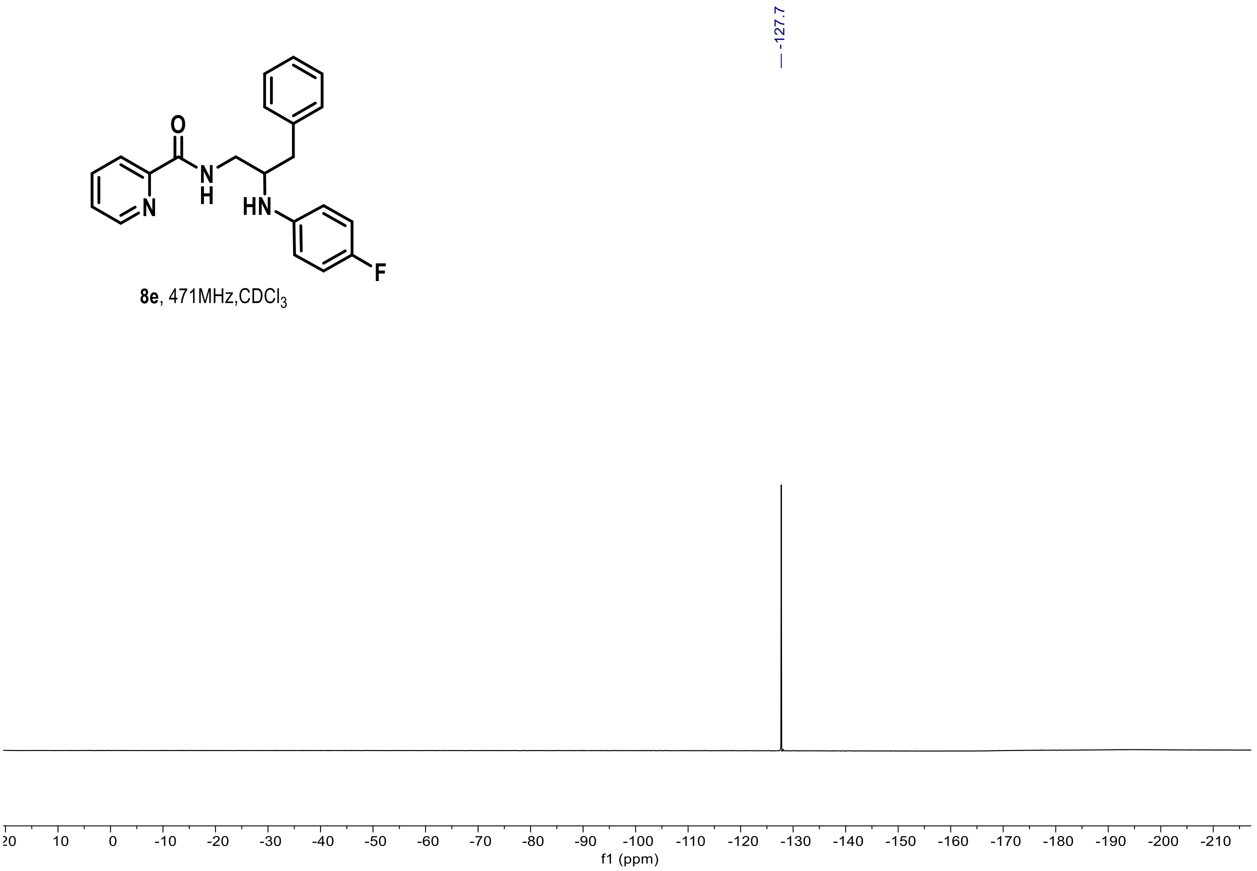

8e, 471MHz,CDCl$_3$

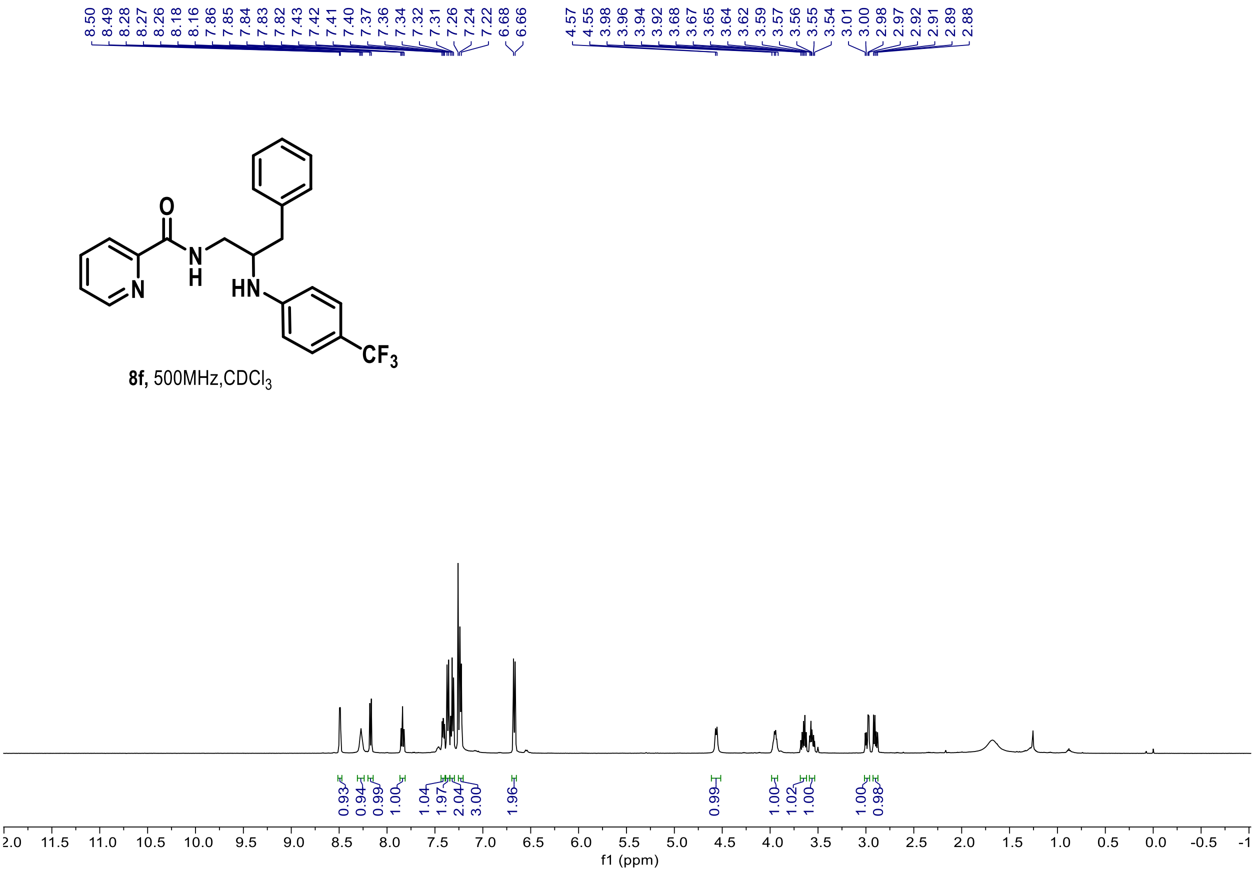
8f, 500MHz,CDCl3
f1 (ppm)

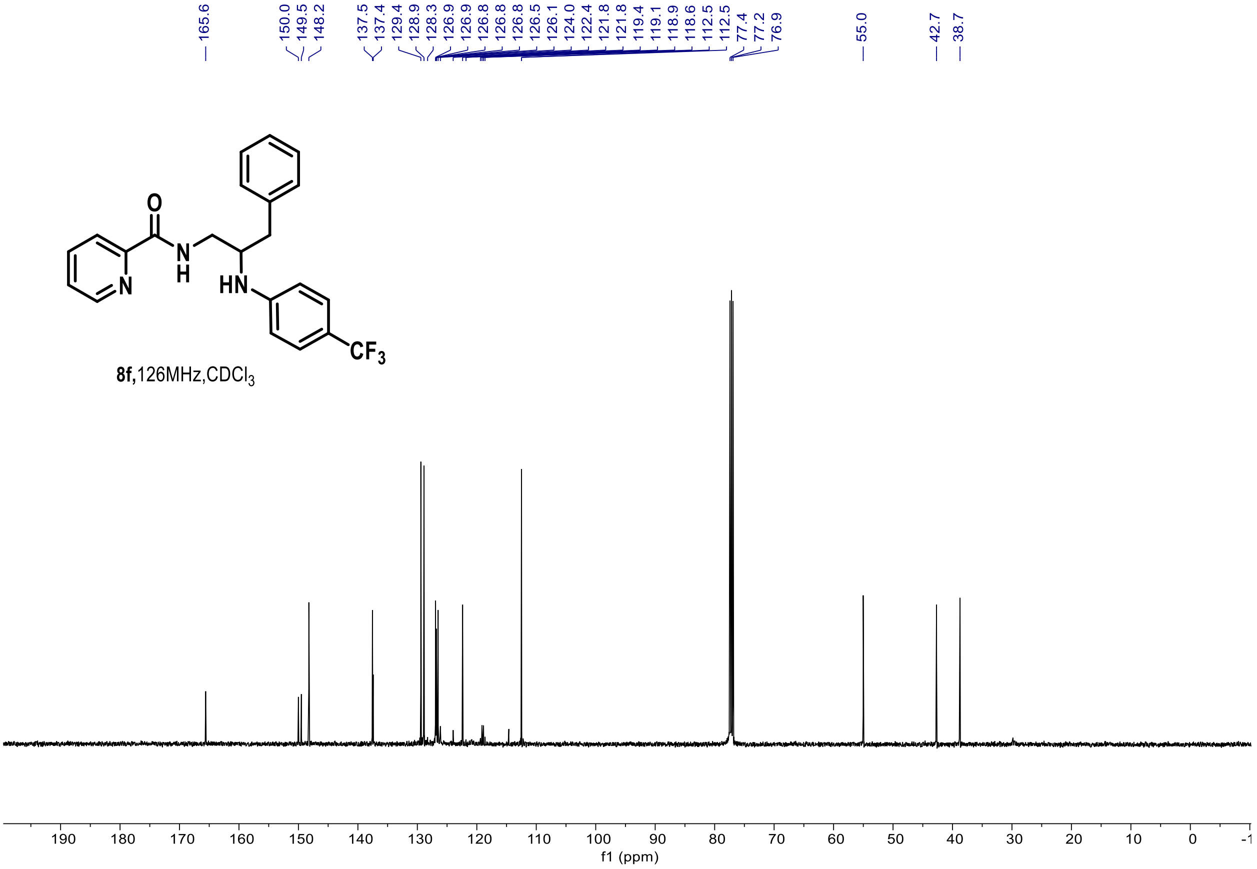
8f,126MHz,CDCl3
f1 (ppm)

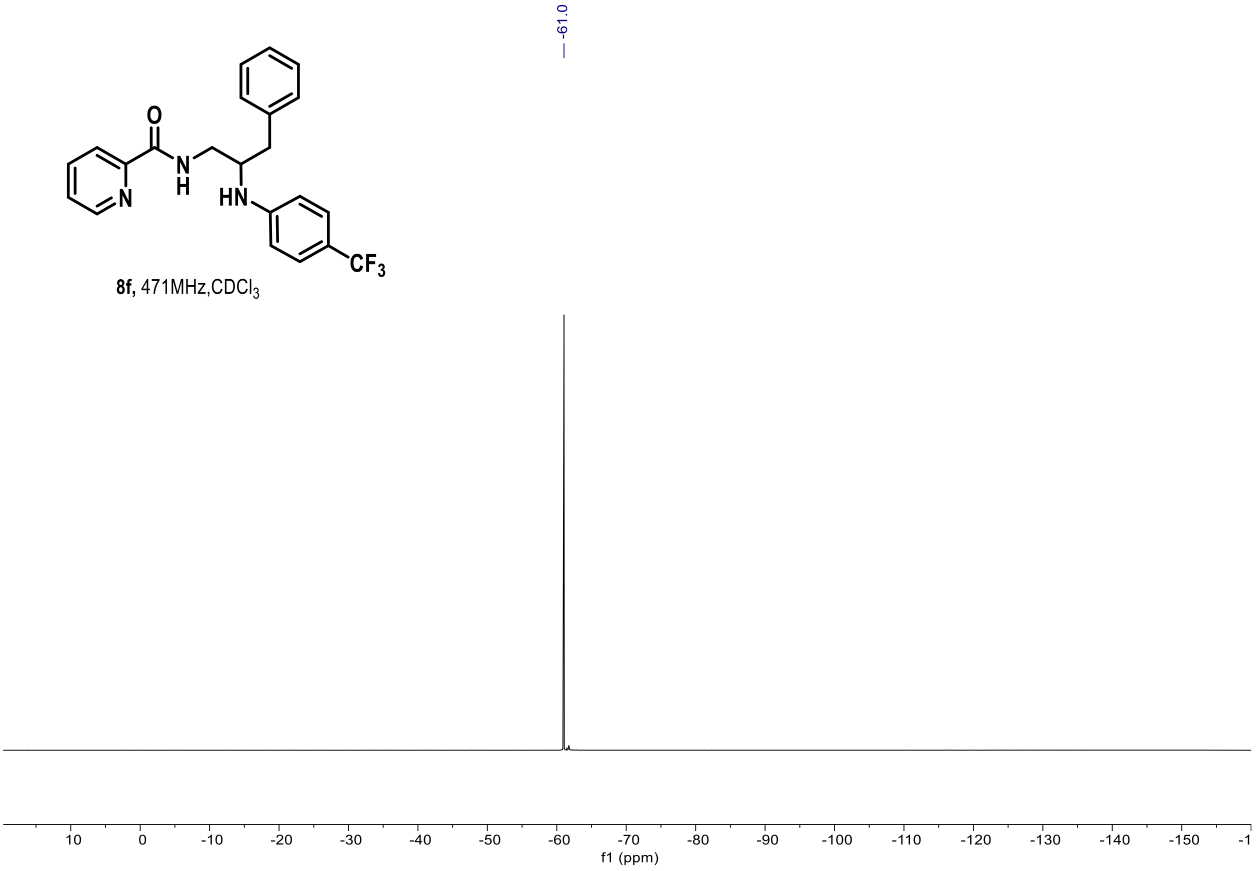

8f, 471MHz,$CDCl_3$

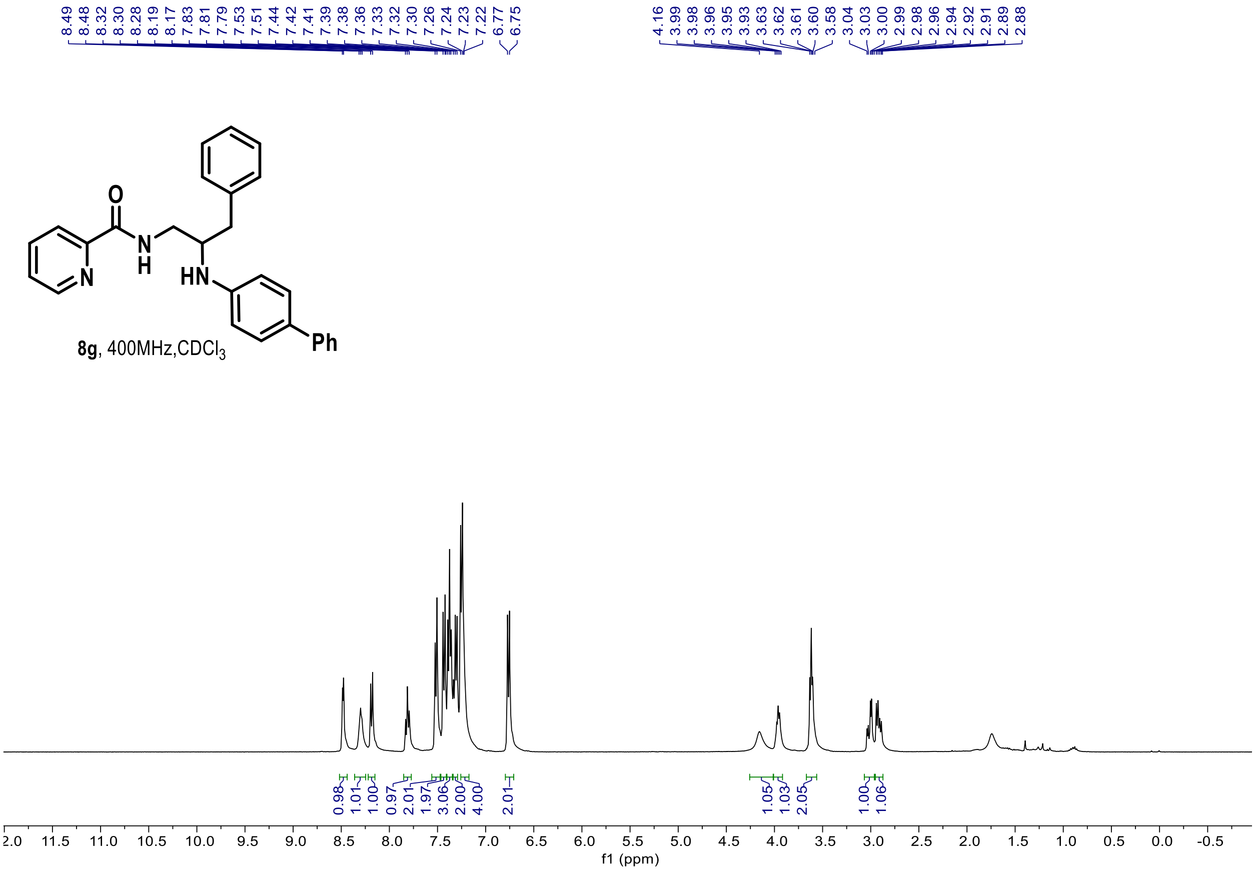

8g, 400MHz,$CDCl_3$

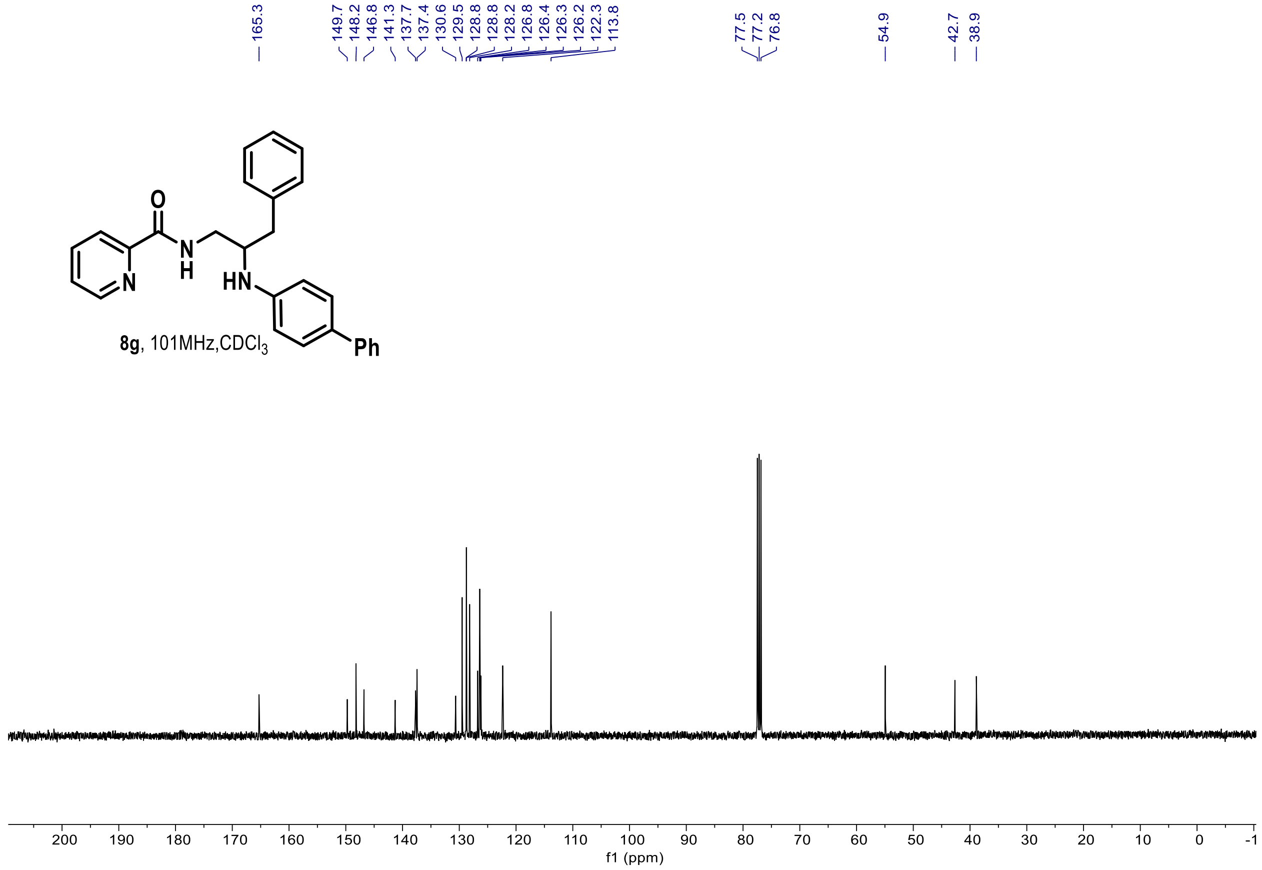

8g, 101MHz,$CDCl_3$

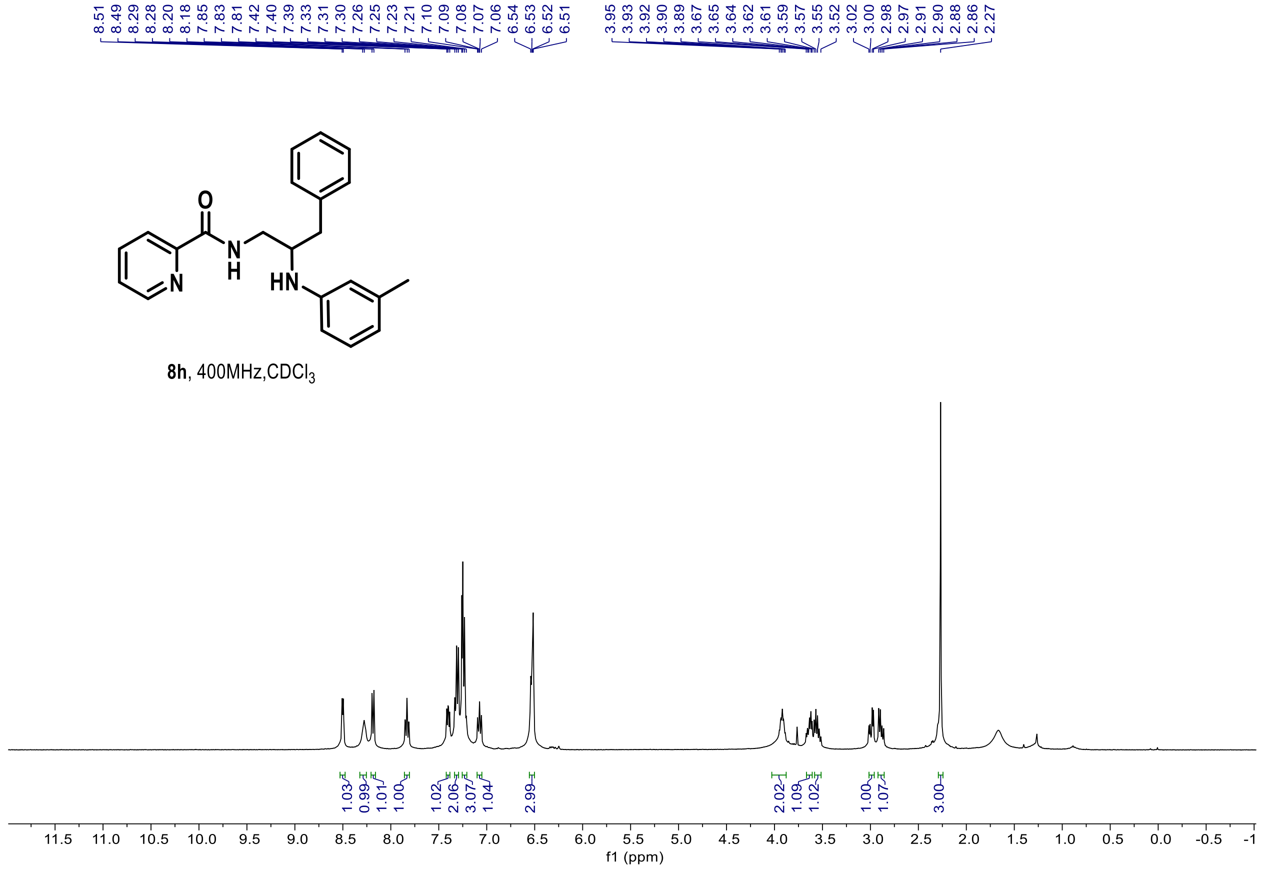

8h, 400MHz,$CDCl_3$

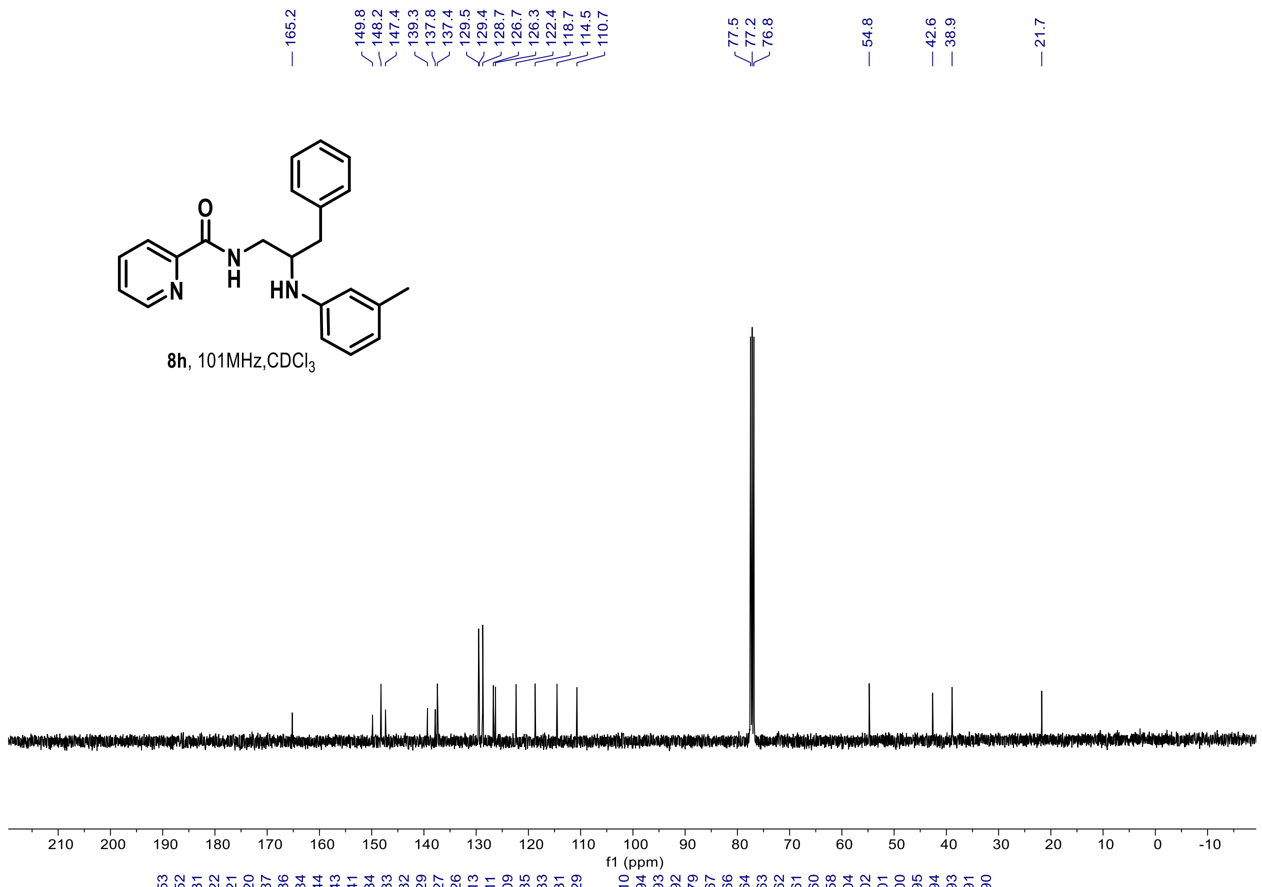

8h, 101MHz,$CDCl_3$

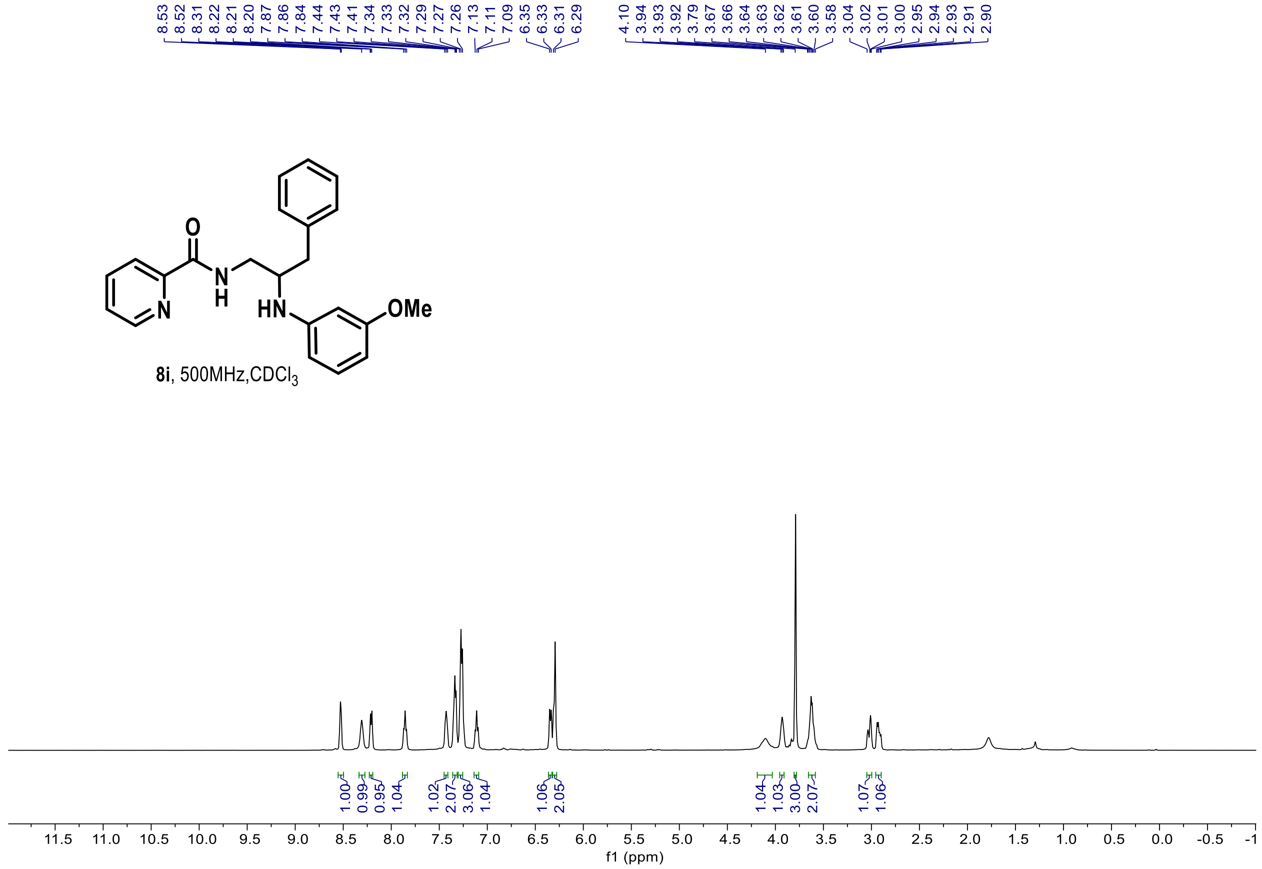

8i, 500MHz,$CDCl_3$

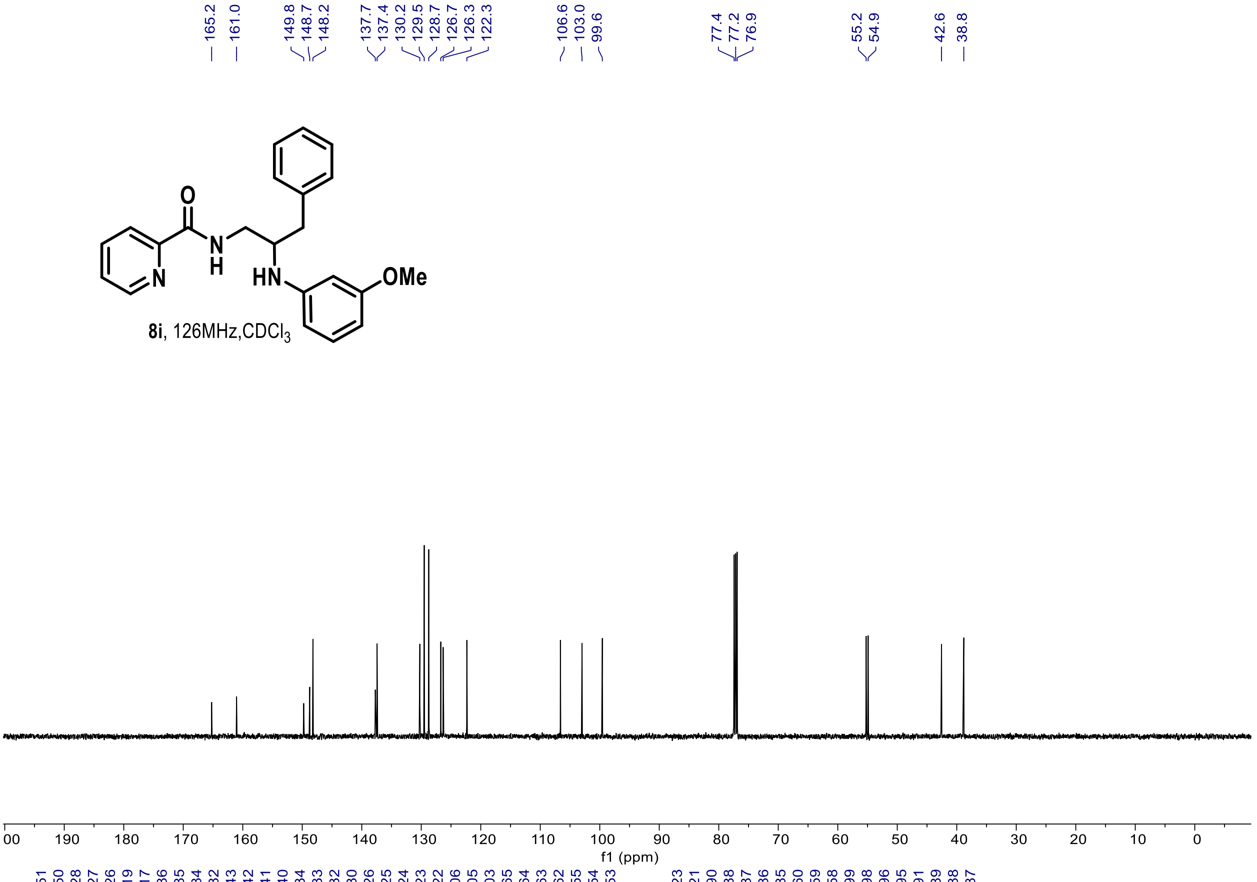

8i, 126MHz,$CDCl_3$

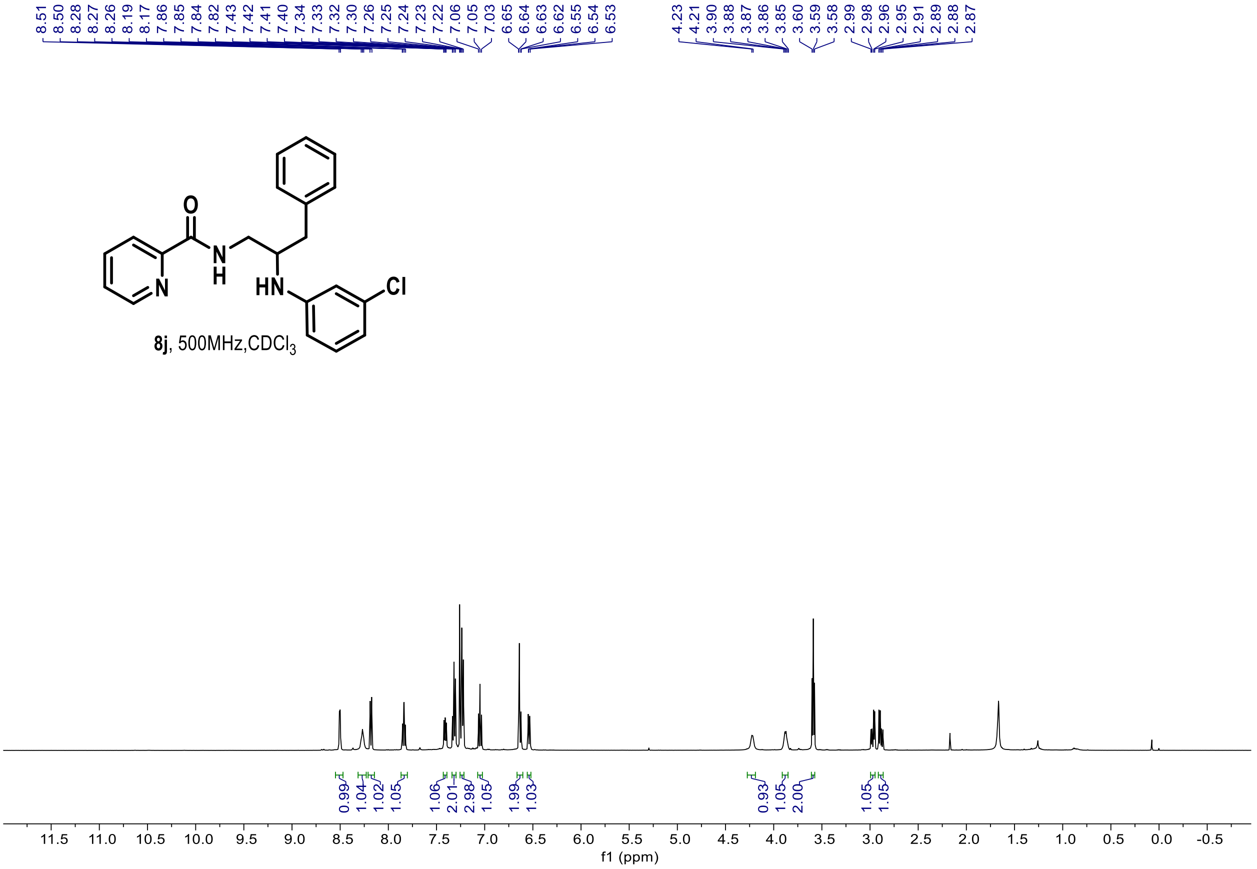

8j, 500MHz,$CDCl_3$

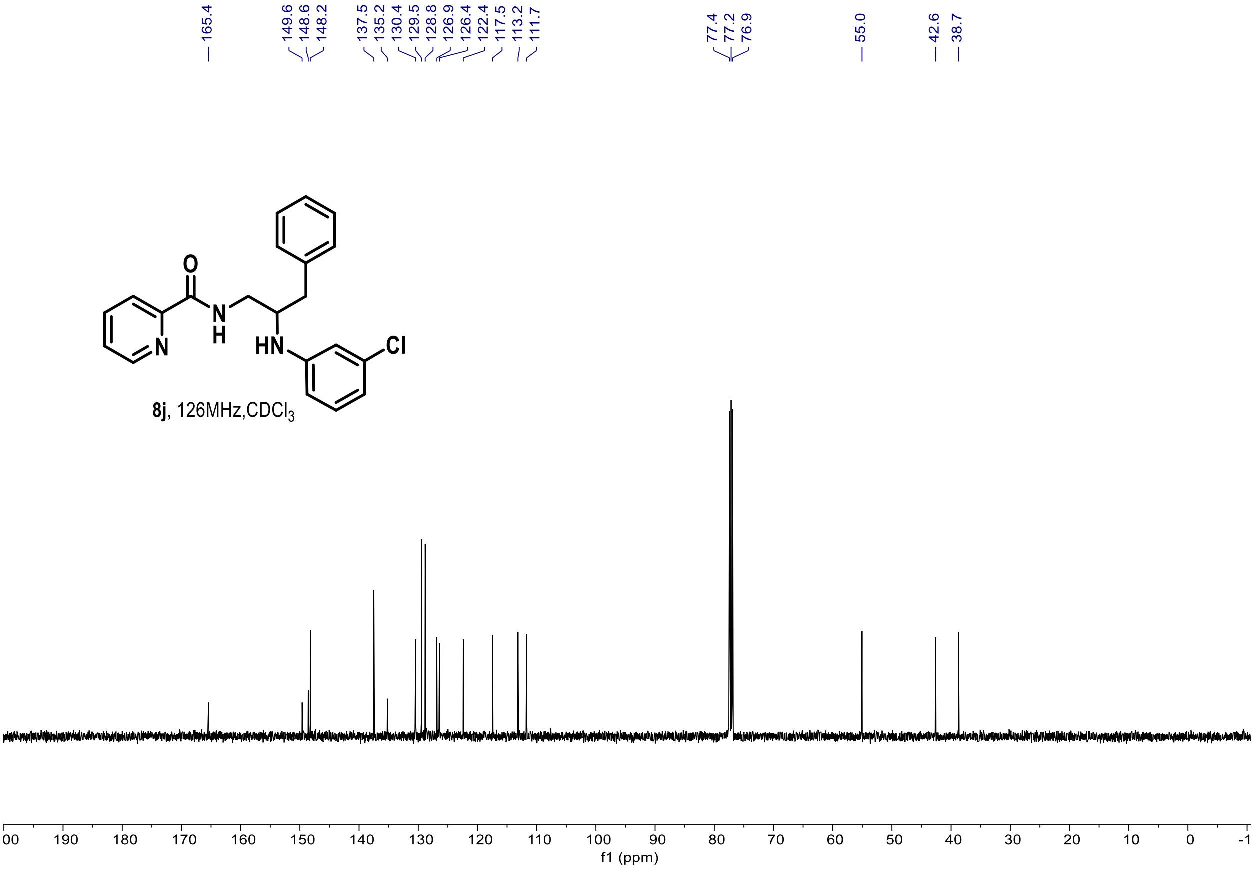
165.4
149.6
148.6
148.2
137.5
135.2
130.4
129.5
128.8
126.9
126.4
122.4
117.5
113.2
111.7
77.4
77.2
76.9
55.0
42.6
38.7
O
N
H
N
HN
Cl
8j, 126MHz,CDCl3
f1 (ppm)

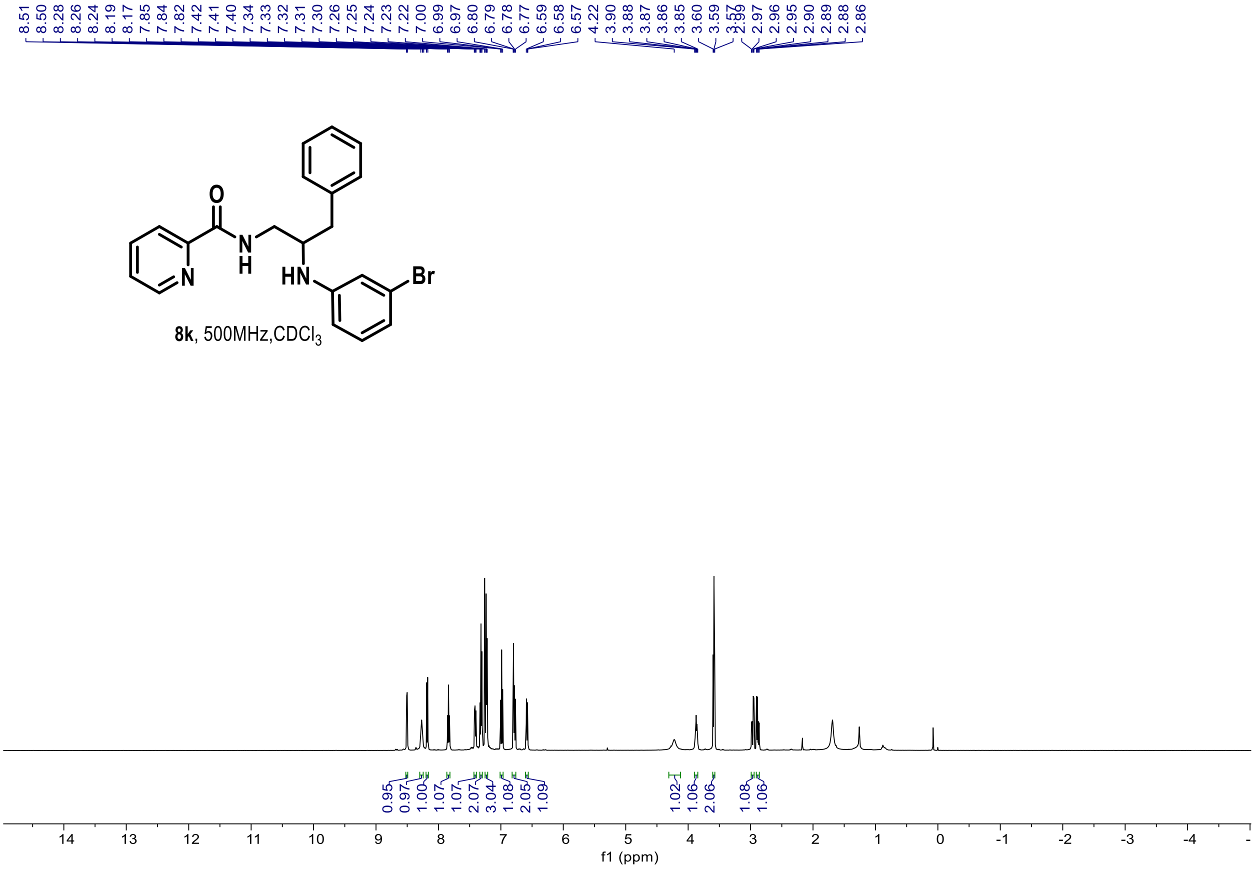
8.51
8.50
8.28
8.26
8.24
8.19
8.17
7.85
7.84
7.82
7.42
7.41
7.40
7.34
7.33
7.32
7.31
7.30
7.26
7.25
7.24
7.23
7.22
7.00
6.99
6.97
6.80
6.79
6.78
6.77
6.59
6.58
6.57
4.22
3.90
3.88
3.87
3.86
3.85
3.60
3.59
3.57
2.99
2.97
2.96
2.95
2.90
2.89
2.88
2.86
O
N
H
N
HN
Br
8k, 500MHz,CDCl3
0.95
0.97
1.00
1.07
1.07
2.07
3.04
1.08
2.05
1.09
1.02
1.06
2.06
1.08
1.06
f1 (ppm)

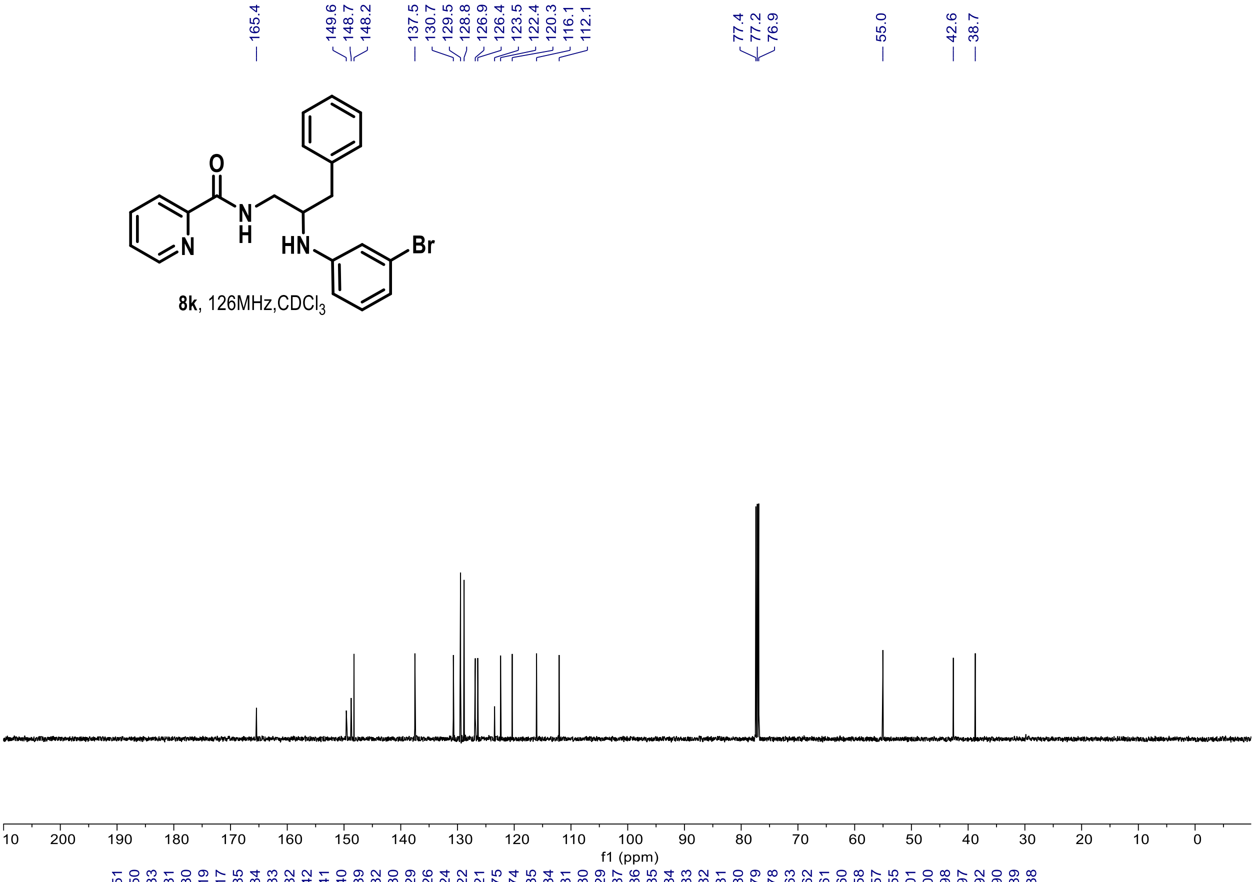
165.4
149.6
148.7
148.2
137.5
130.7
129.5
128.8
126.9
126.4
123.5
122.4
120.3
116.1
112.1
77.4
77.2
76.9
55.0
42.6
38.7
8k, 126MHz,CDCl3
f1 (ppm)

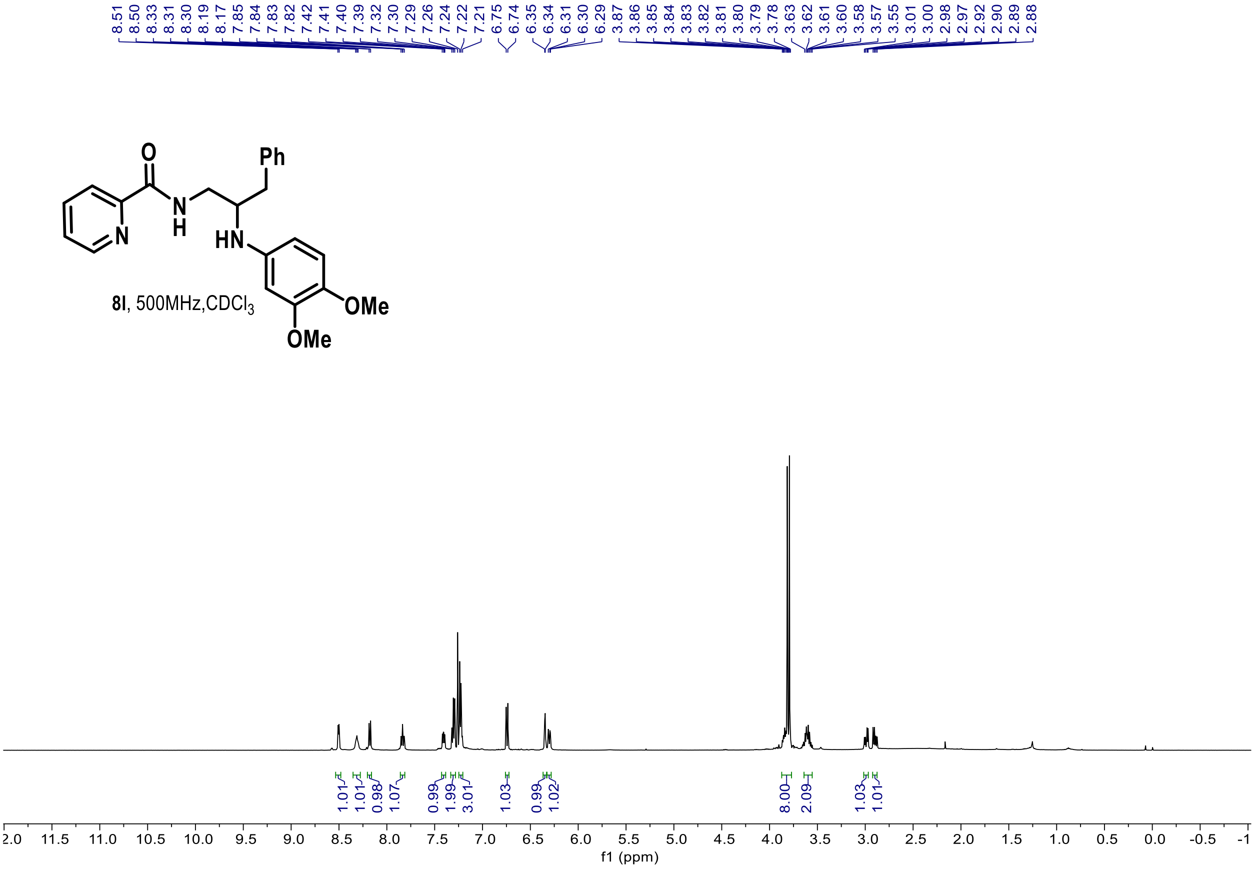
8.51
8.50
8.33
8.31
8.30
8.19
8.17
7.85
7.84
7.83
7.82
7.42
7.41
7.40
7.39
7.32
7.30
7.29
7.26
7.24
7.22
7.21
6.75
6.74
6.35
6.34
6.31
6.30
6.29
3.87
3.86
3.85
3.84
3.83
3.82
3.81
3.80
3.79
3.78
3.63
3.62
3.61
3.60
3.58
3.57
3.55
3.01
3.00
2.98
2.97
2.92
2.90
2.89
2.88
8l, 500MHz,CDCl3
1.01
1.01
0.98
1.07
0.99
1.99
3.01
1.03
0.99
1.02
8.00
2.09
1.03
1.01
f1 (ppm)

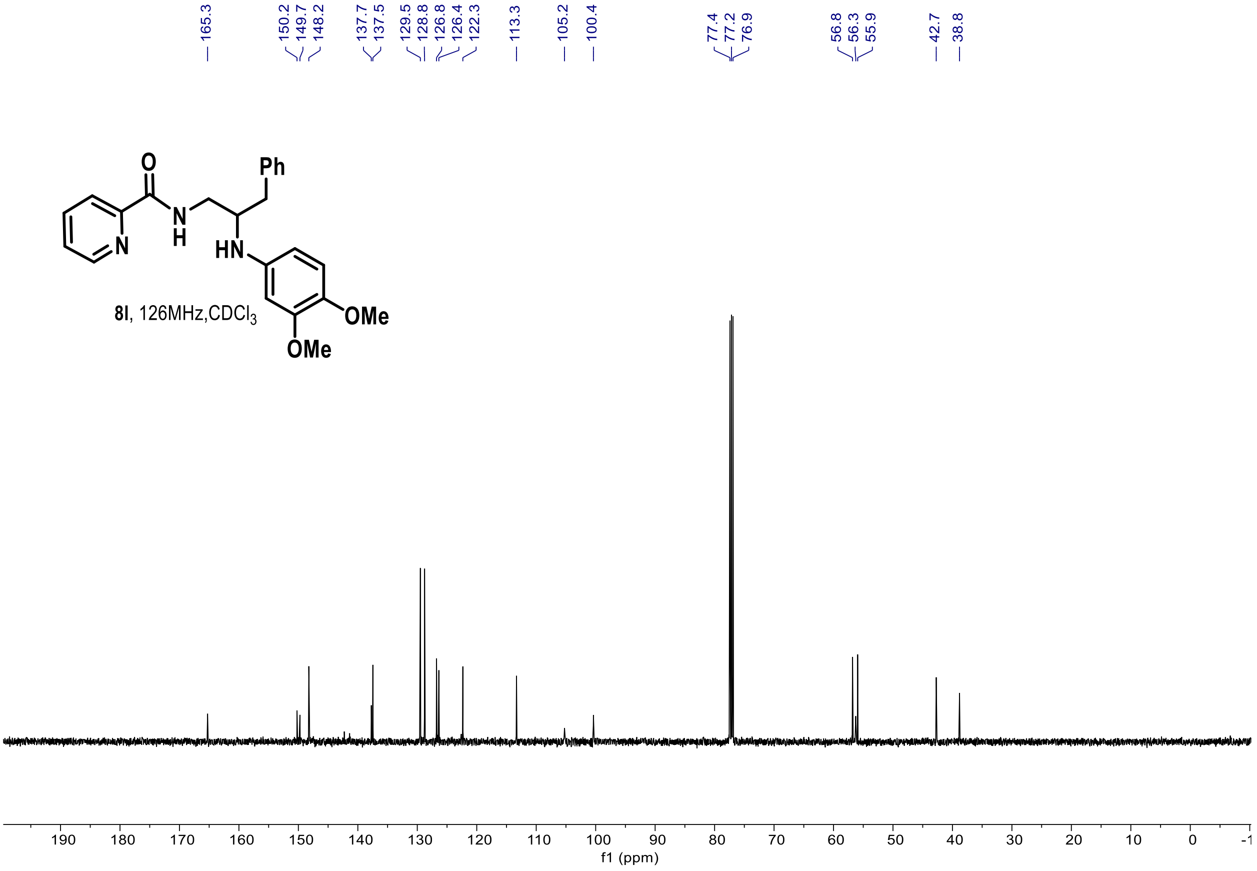
165.3
150.2
149.7
148.2
137.7
137.5
129.5
128.8
126.8
126.4
122.3
113.3
105.2
100.4
77.4
77.2
76.9
56.8
56.3
55.9
42.7
38.8
O
Ph
N
H
N
HN
OMe
OMe
8l, 126MHz,CDCl$_3$
190 180 170 160 150 140 130 120 110 100 90 80 70 60 50 40 30 20 10 0 -1
f1 (ppm)

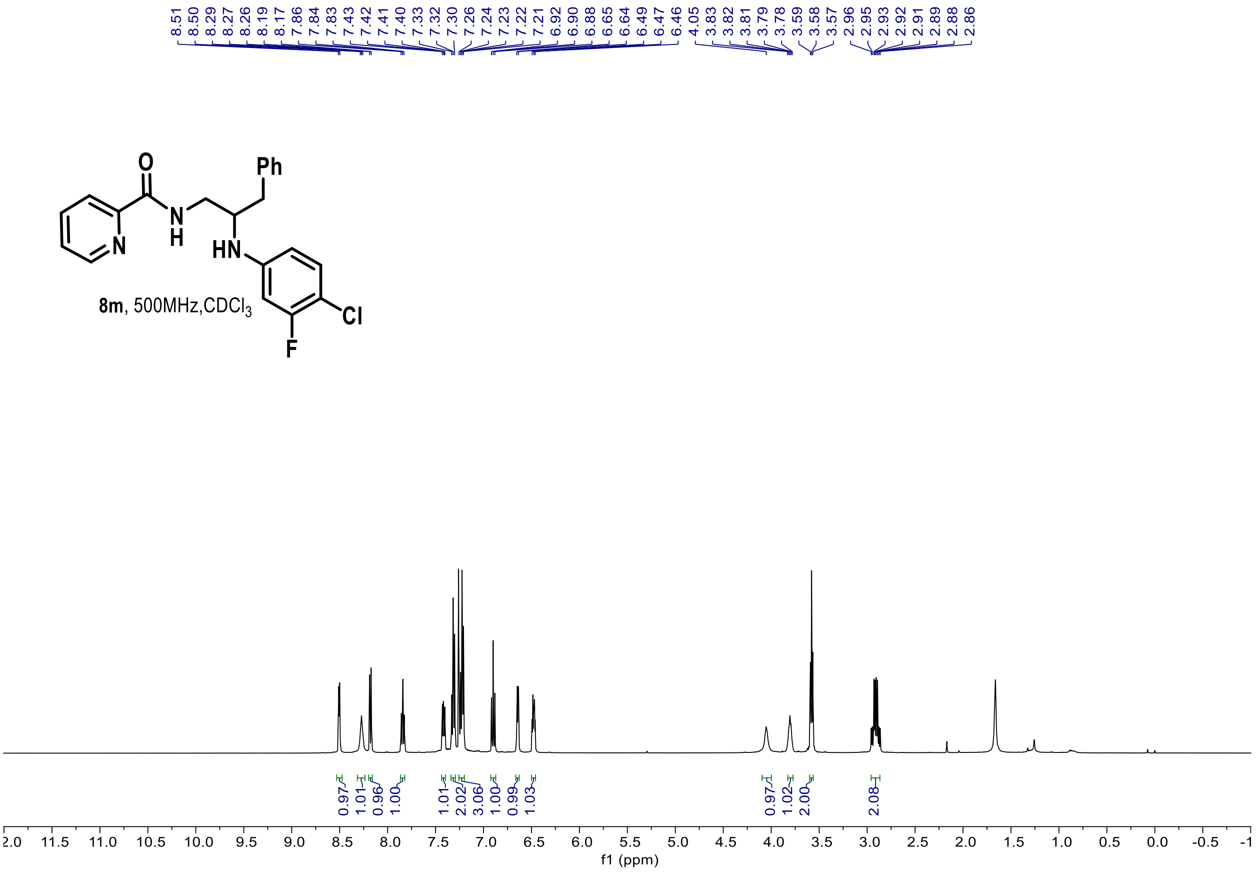
8.51 8.50 8.29 8.27 8.26 8.19 8.17 7.86 7.84 7.83 7.43 7.42 7.41 7.40 7.33 7.32 7.30 7.26 7.24 7.23 7.22 7.21 6.92 6.90 6.88 6.65 6.64 6.49 6.47 6.46 4.05 3.83 3.82 3.81 3.79 3.78 3.59 3.58 3.57 2.96 2.95 2.93 2.92 2.91 2.89 2.88 2.86
O
Ph
N
H
N
HN
Cl
F
8m, 500MHz,CDCl$_3$
0.97 1.01 0.96 1.00 1.01 2.02 3.06 1.00 0.99 1.03 0.97 1.02 2.00 2.08
2.0 11.5 11.0 10.5 10.0 9.5 9.0 8.5 8.0 7.5 7.0 6.5 6.0 5.5 5.0 4.5 4.0 3.5 3.0 2.5 2.0 1.5 1.0 0.5 0.0 -0.5 -1
f1 (ppm)

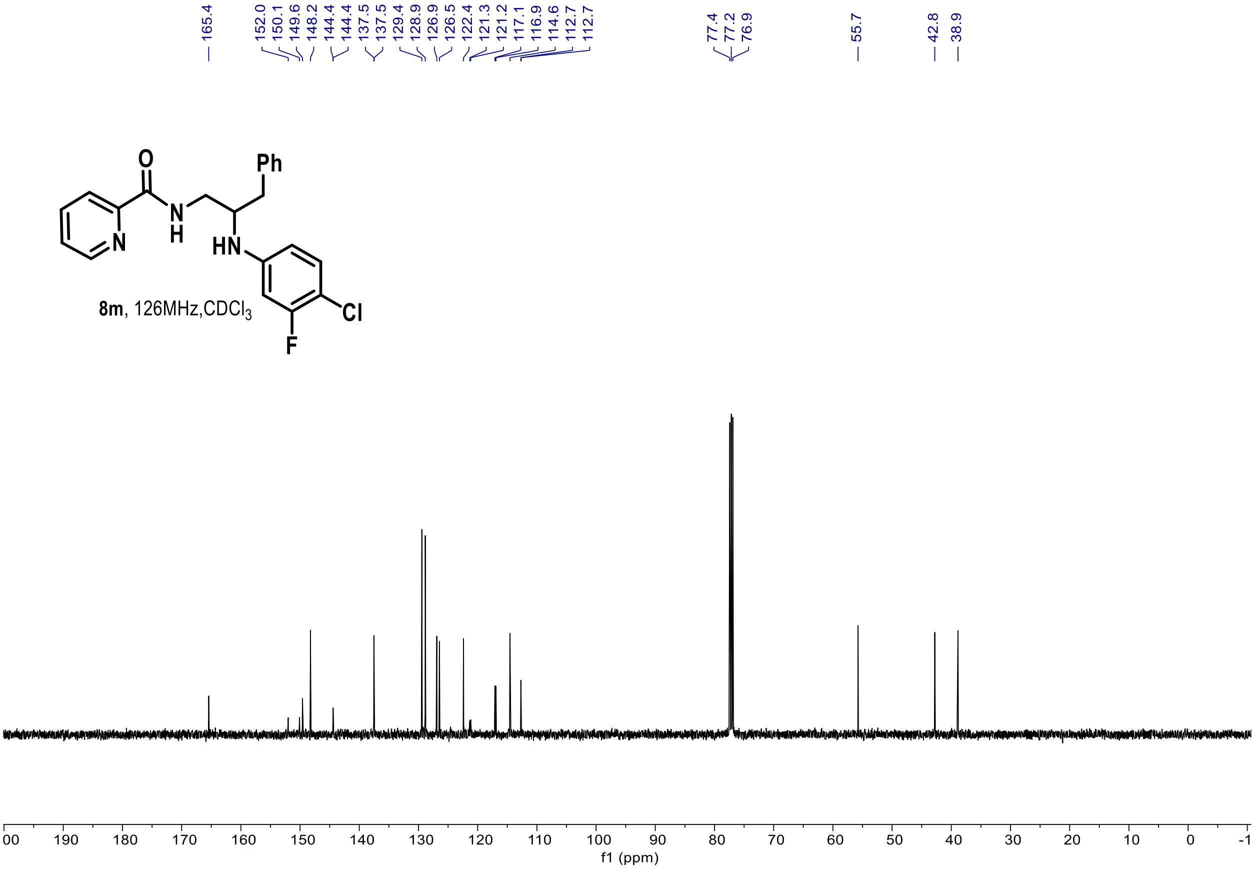
165.4
152.0
150.1
149.6
148.2
144.4
144.4
137.5
137.5
129.4
128.9
126.9
126.5
122.4
121.3
121.2
117.1
116.9
114.6
112.7
112.7
77.4
77.2
76.9
55.7
42.8
38.9
Ph
O
N
H
N
HN
Cl
F
8m, 126MHz,CDCl3
f1 (ppm)

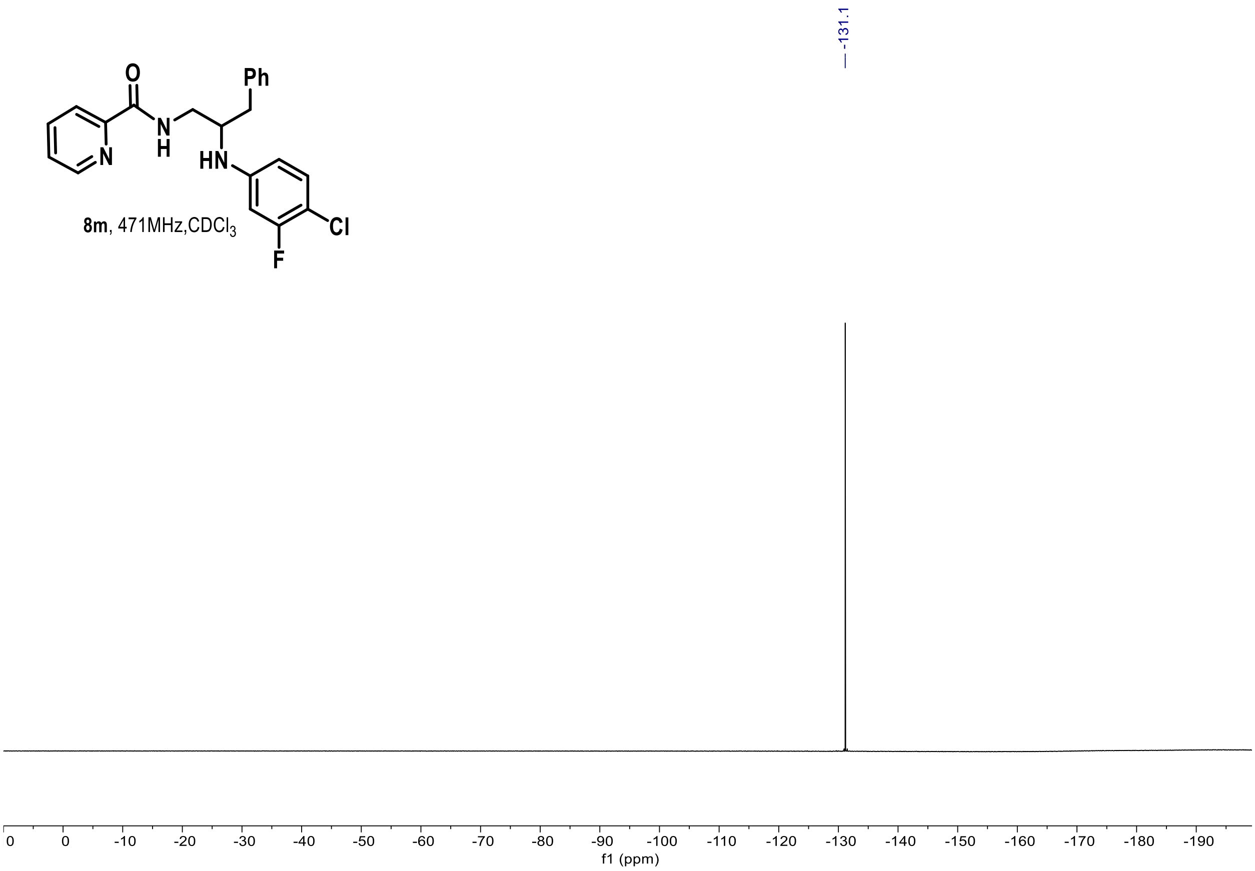
-131.1
Ph
O
N
H
N
HN
Cl
F
8m, 471MHz,CDCl3
f1 (ppm)

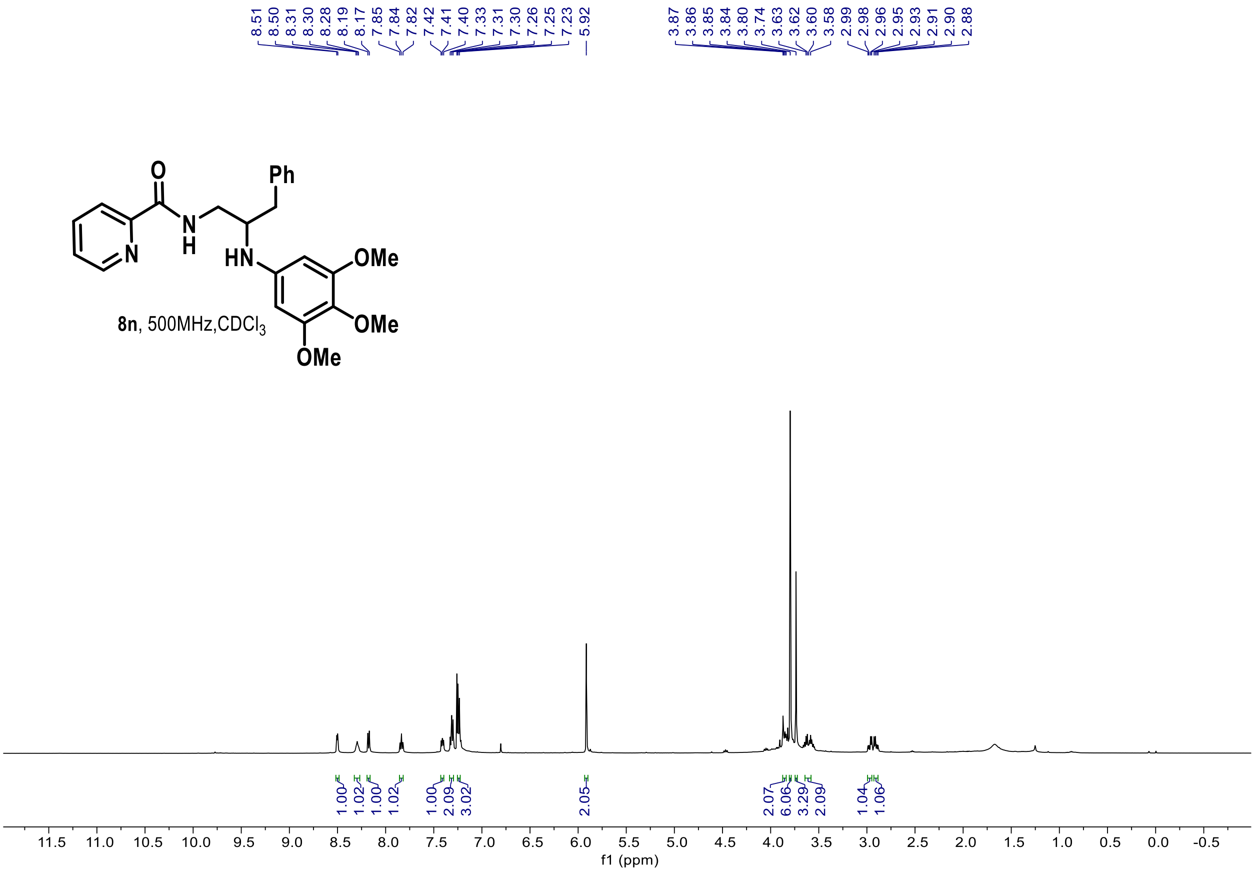
8.51 8.50 8.31 8.30 8.28 8.19 8.17 7.85 7.84 7.82 7.42 7.41 7.40 7.33 7.31 7.30 7.26 7.25 7.23 5.92
3.87 3.86 3.85 3.84 3.80 3.74 3.63 3.62 3.60 3.58 2.99 2.98 2.96 2.95 2.93 2.91 2.90 2.88
Ph
O
N
H
N
HN
OMe
OMe
OMe
8n, 500MHz,CDCl3
1.00 1.02 1.00 1.02 1.00 2.09 3.02 2.05 2.07 6.06 3.29 2.09 1.04 1.06
f1 (ppm)

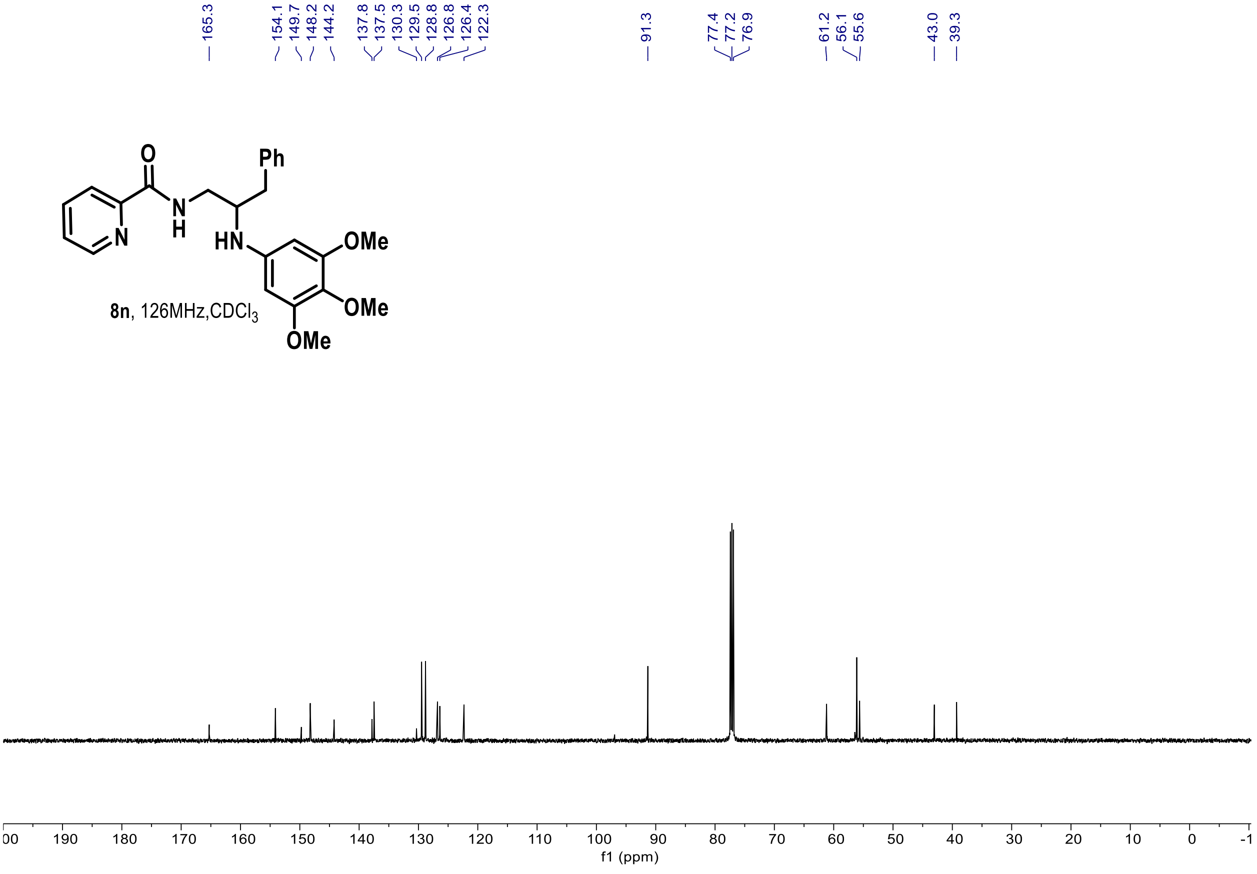
165.3 154.1 149.7 148.2 144.2 137.8 137.5 130.3 129.5 128.8 126.8 126.4 122.3 91.3 77.4 77.2 76.9 61.2 56.1 55.6 43.0 39.3
Ph
O
N
H
N
HN
OMe
OMe
OMe
8n, 126MHz,CDCl3
f1 (ppm)

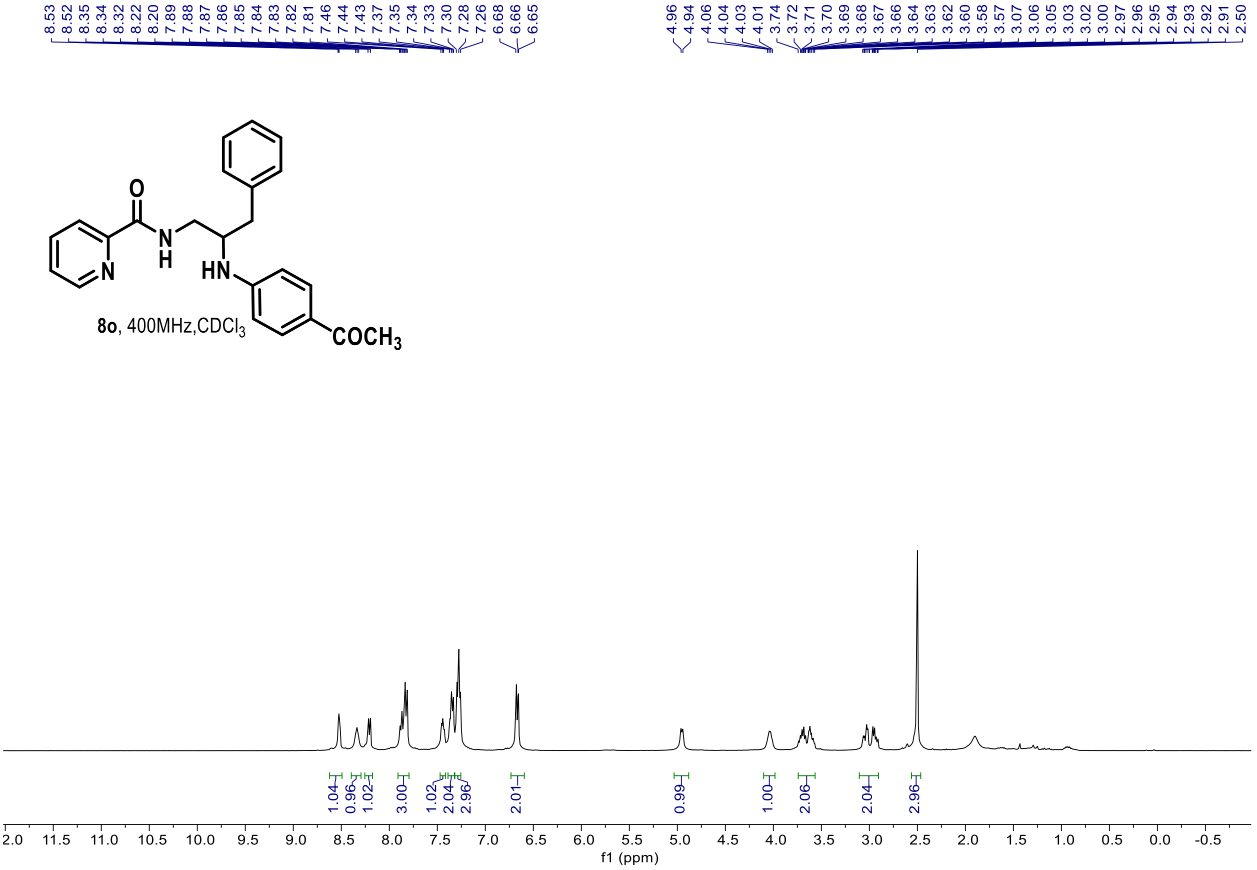

8o, 400MHz,CDCl$_3$

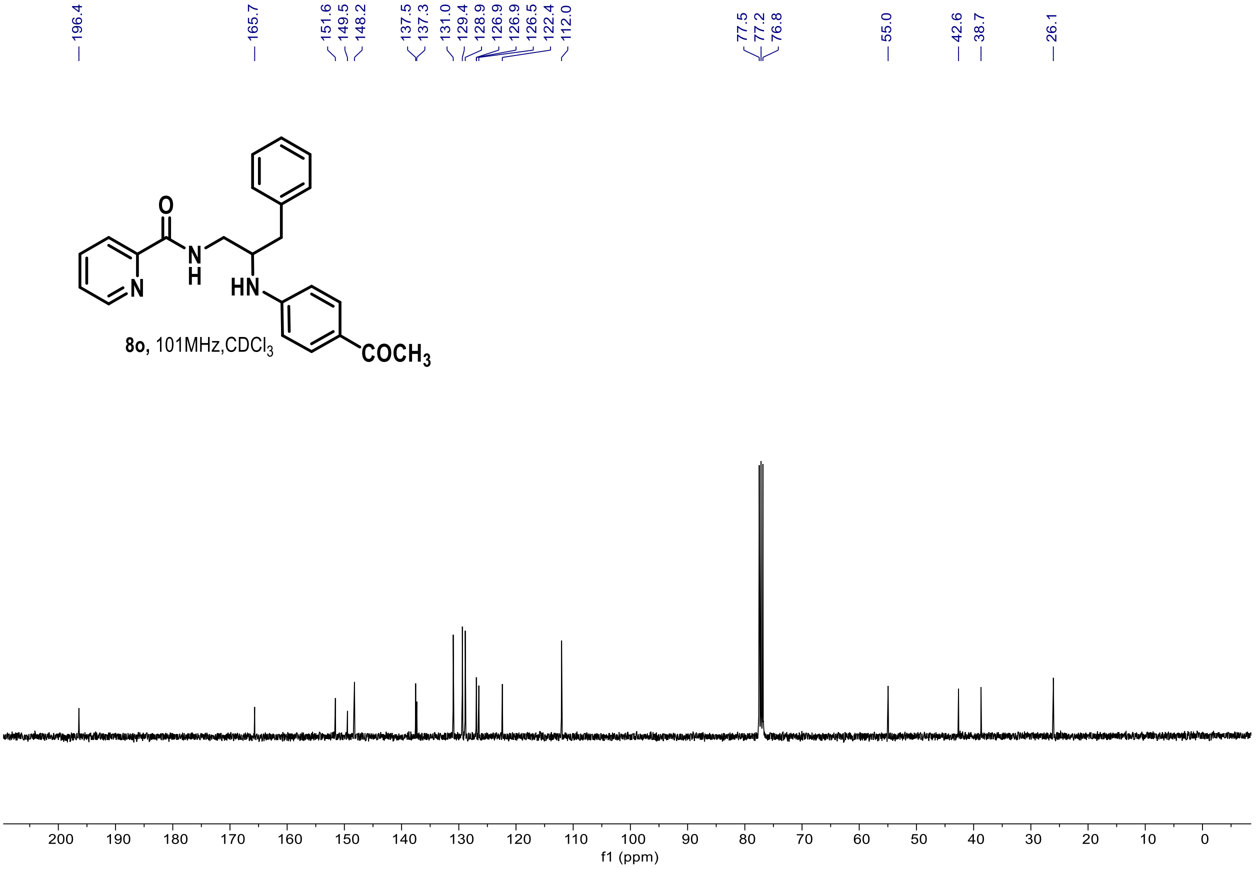

8o, 101MHz,CDCl$_3$

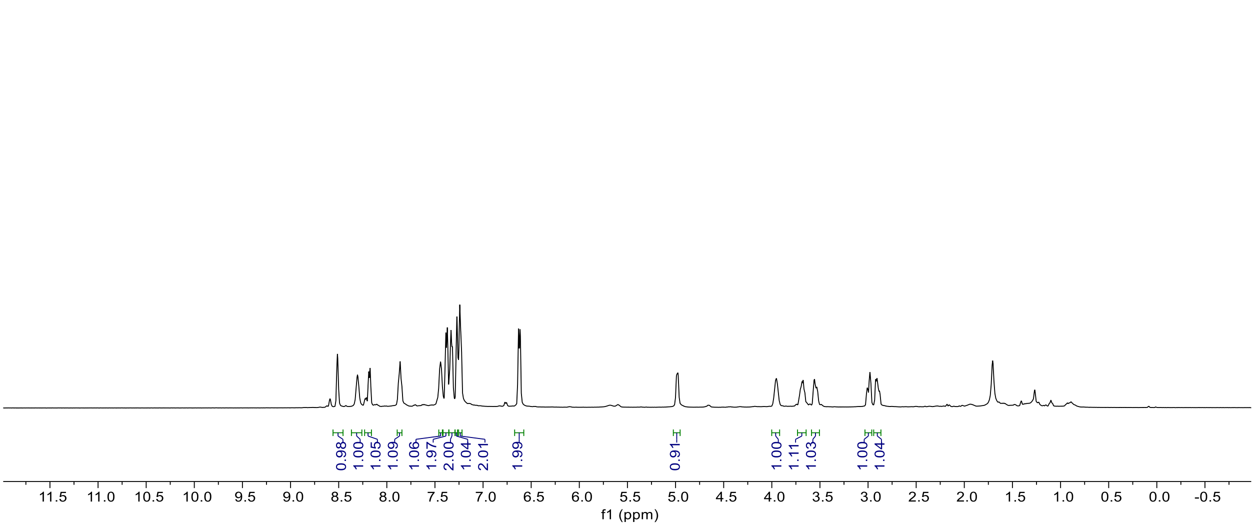

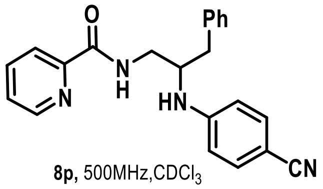

8p, 500MHz,CDCl$_3$

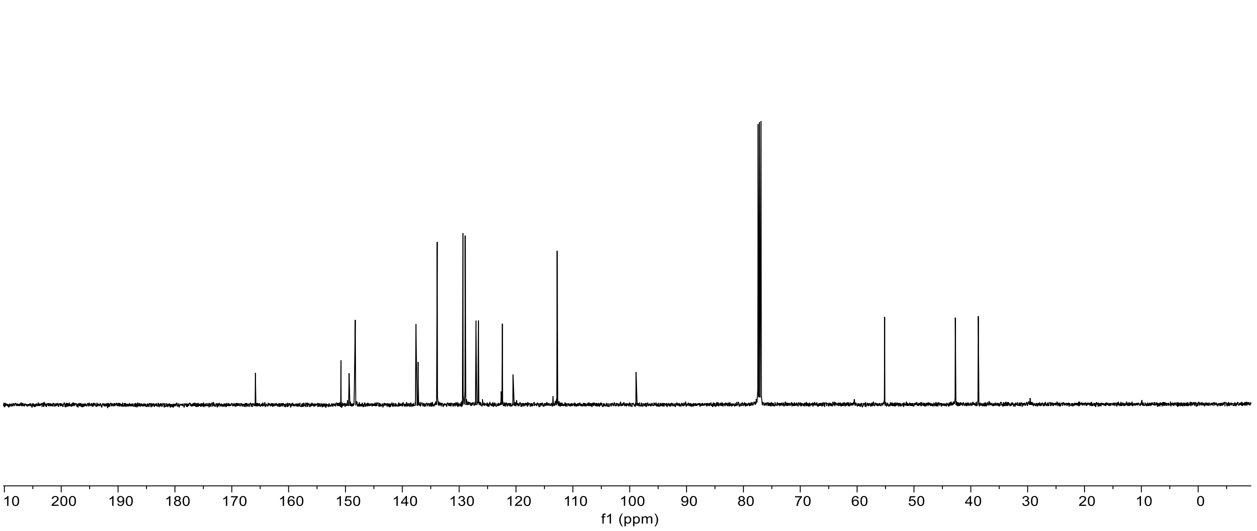

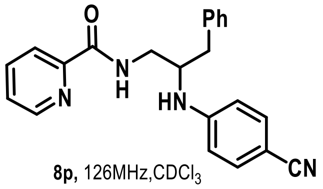

8p, 126MHz,CDCl$_3$

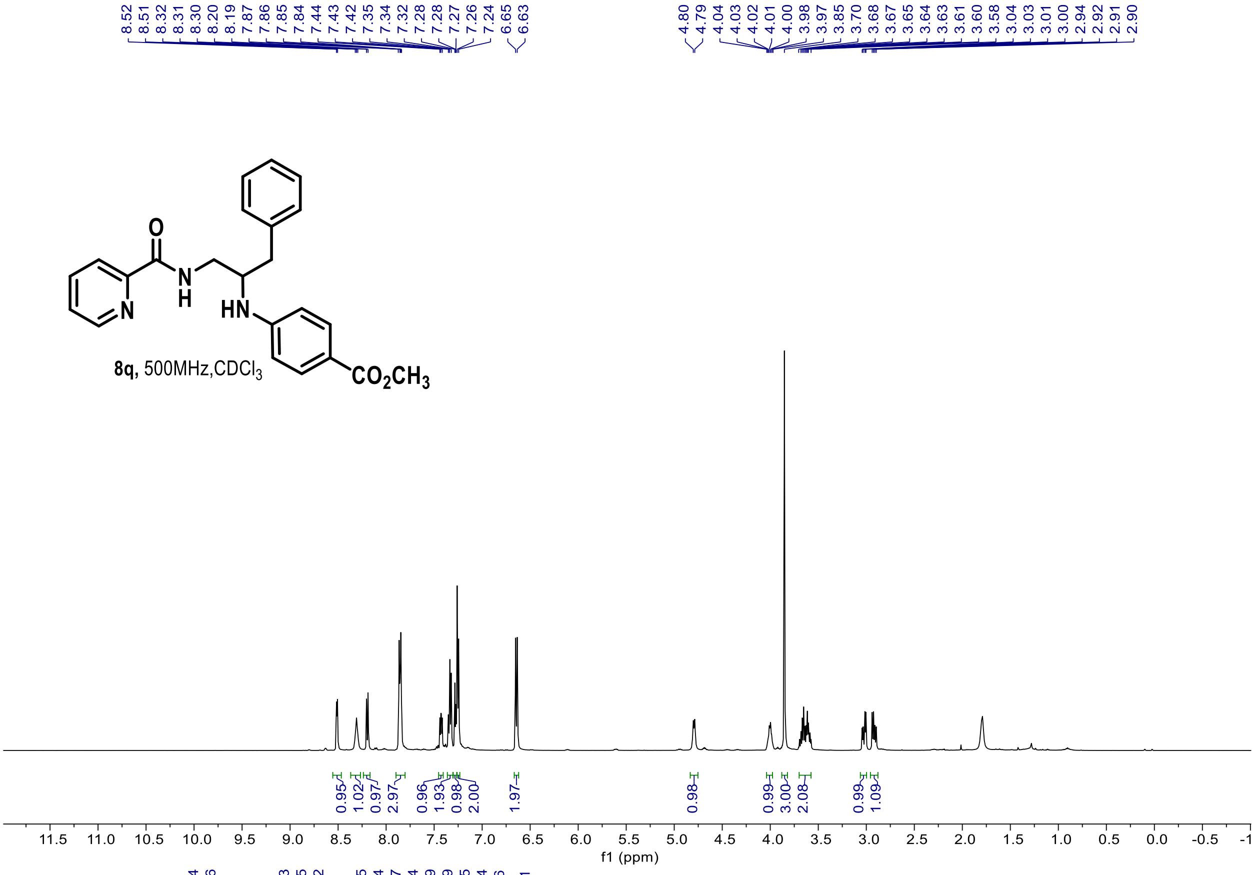
8q, 500MHz,CDCl3
f1 (ppm)

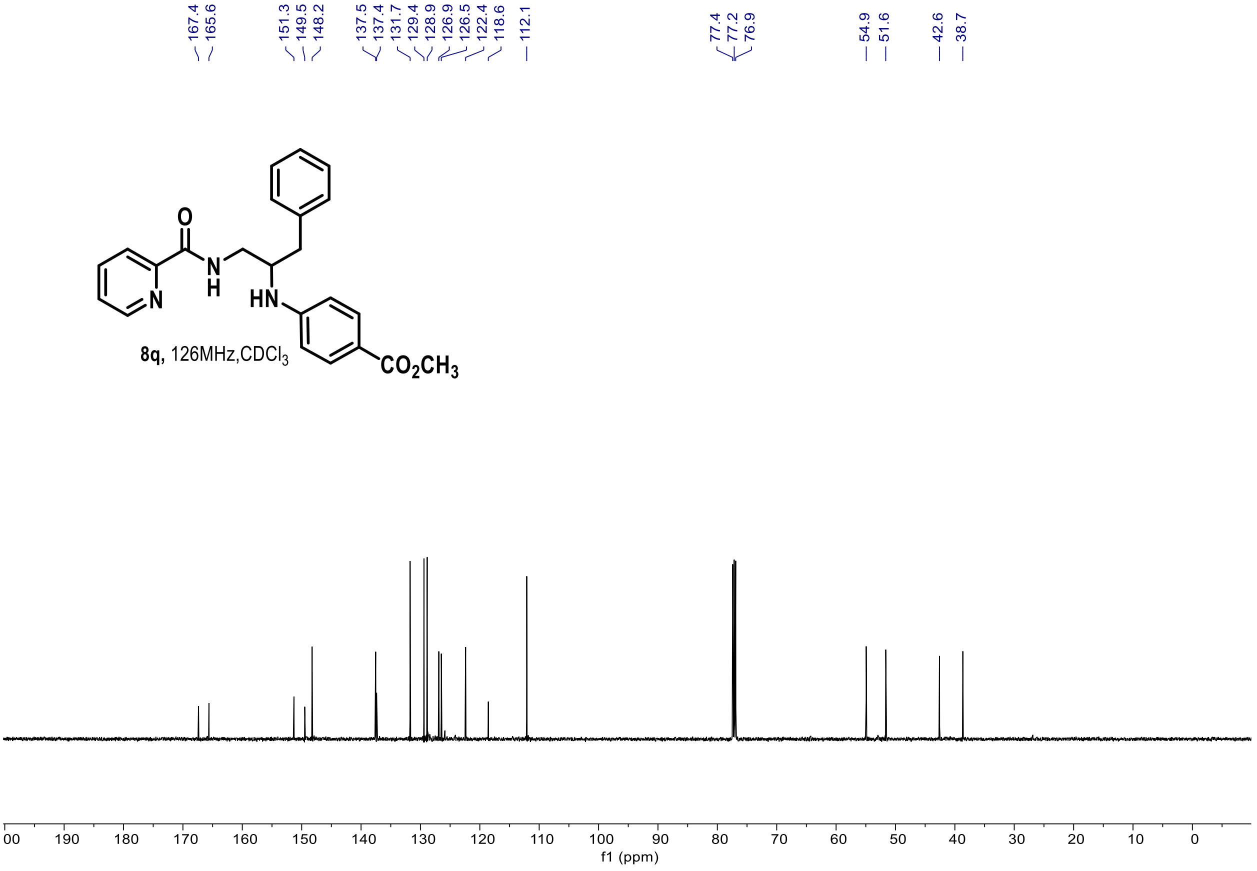
8q, 126MHz,CDCl3
f1 (ppm)

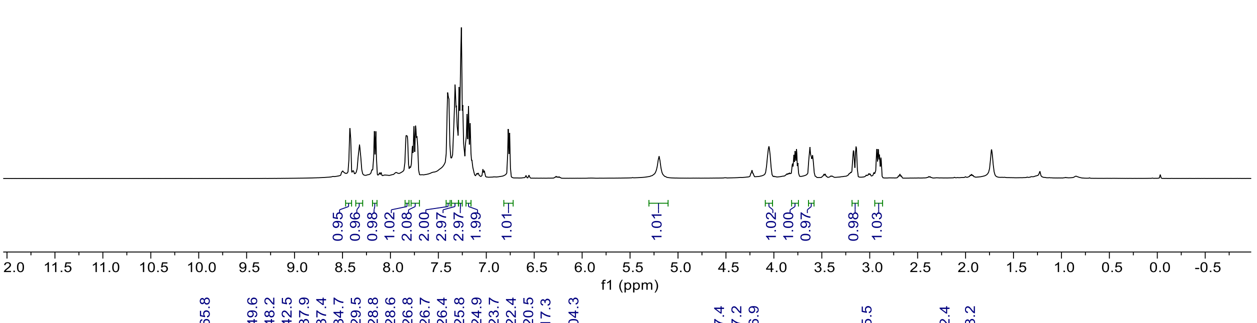

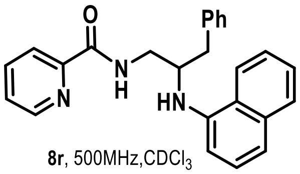

8r, 500MHz,$CDCl_3$

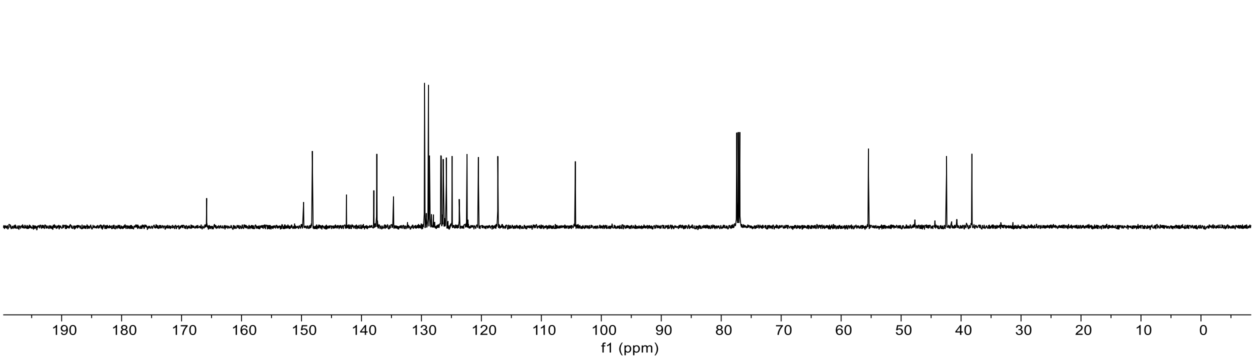

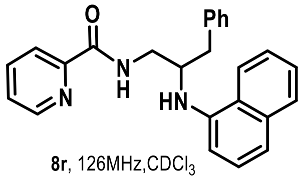

8r, 126MHz,$CDCl_3$

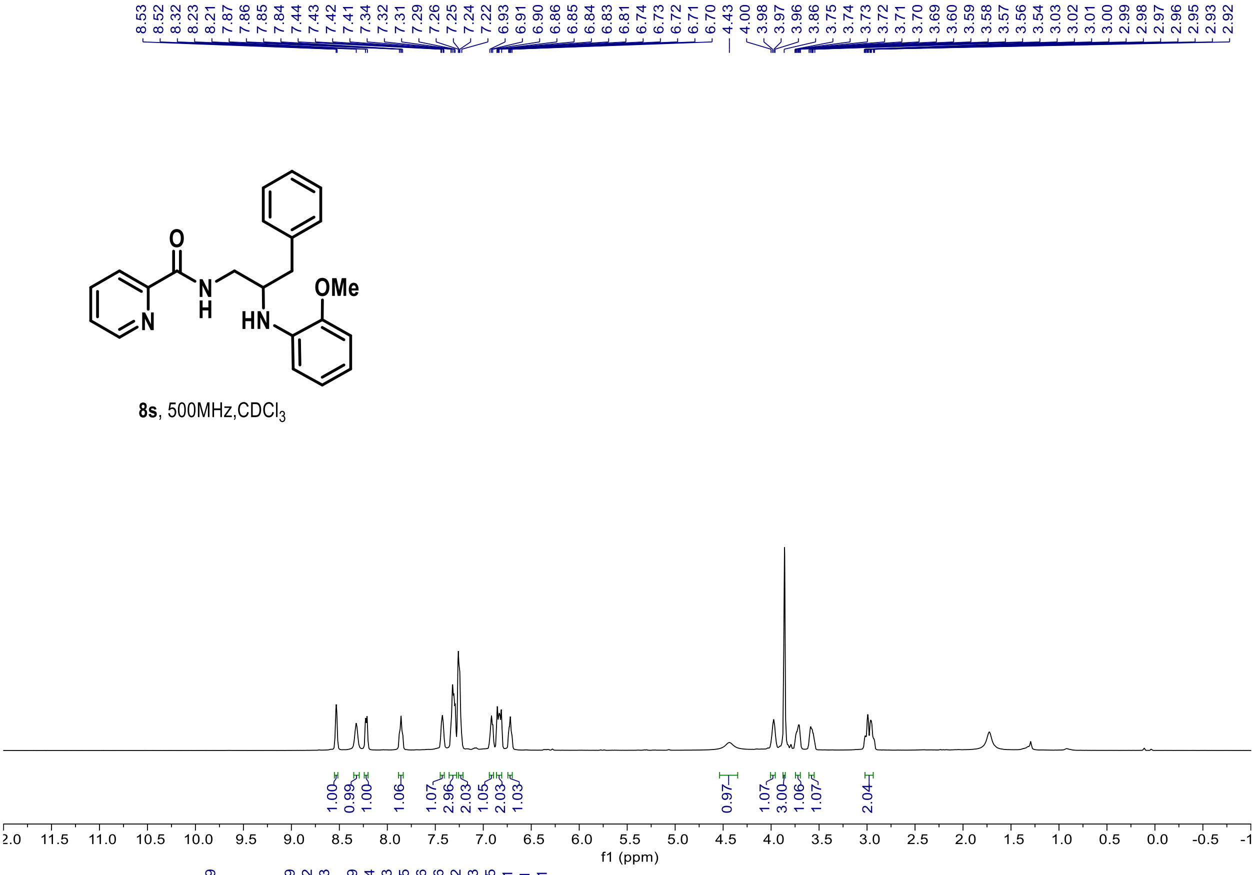
8.53 8.52 8.32 8.23 8.21 7.87 7.86 7.85 7.84 7.44 7.43 7.42 7.41 7.34 7.32 7.31 7.29 7.26 7.25 7.24 7.22 6.93 6.91 6.90 6.86 6.85 6.84 6.83 6.81 6.74 6.73 6.72 6.71 6.70 4.43 4.00 3.98 3.97 3.96 3.86 3.75 3.74 3.73 3.72 3.71 3.70 3.69 3.60 3.59 3.58 3.57 3.56 3.54 3.03 3.02 3.01 3.00 2.99 2.98 2.97 2.96 2.95 2.93 2.92
O
N
H
N
HN
OMe
8s, 500MHz,CDCl$_3$
1.00 0.99 1.00 1.06 1.07 2.96 2.03 1.05 2.03 1.03 0.97 1.07 3.00 1.06 1.07 2.04
f1 (ppm)

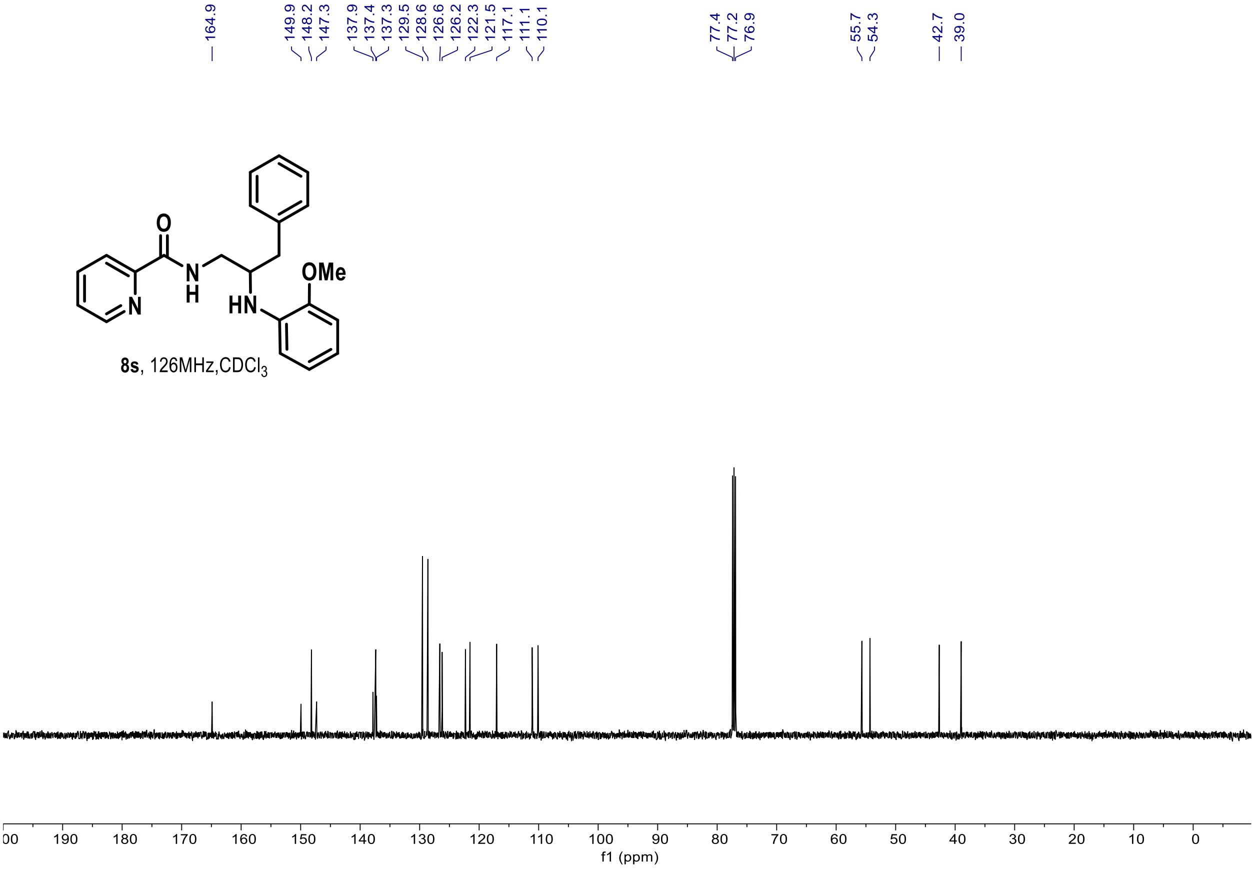
164.9 149.9 148.2 147.3 137.9 137.4 137.3 129.5 128.6 126.6 126.2 122.3 121.5 117.1 111.1 110.1 77.4 77.2 76.9 55.7 54.3 42.7 39.0
O
N
H
N
HN
OMe
8s, 126MHz,CDCl$_3$
f1 (ppm)

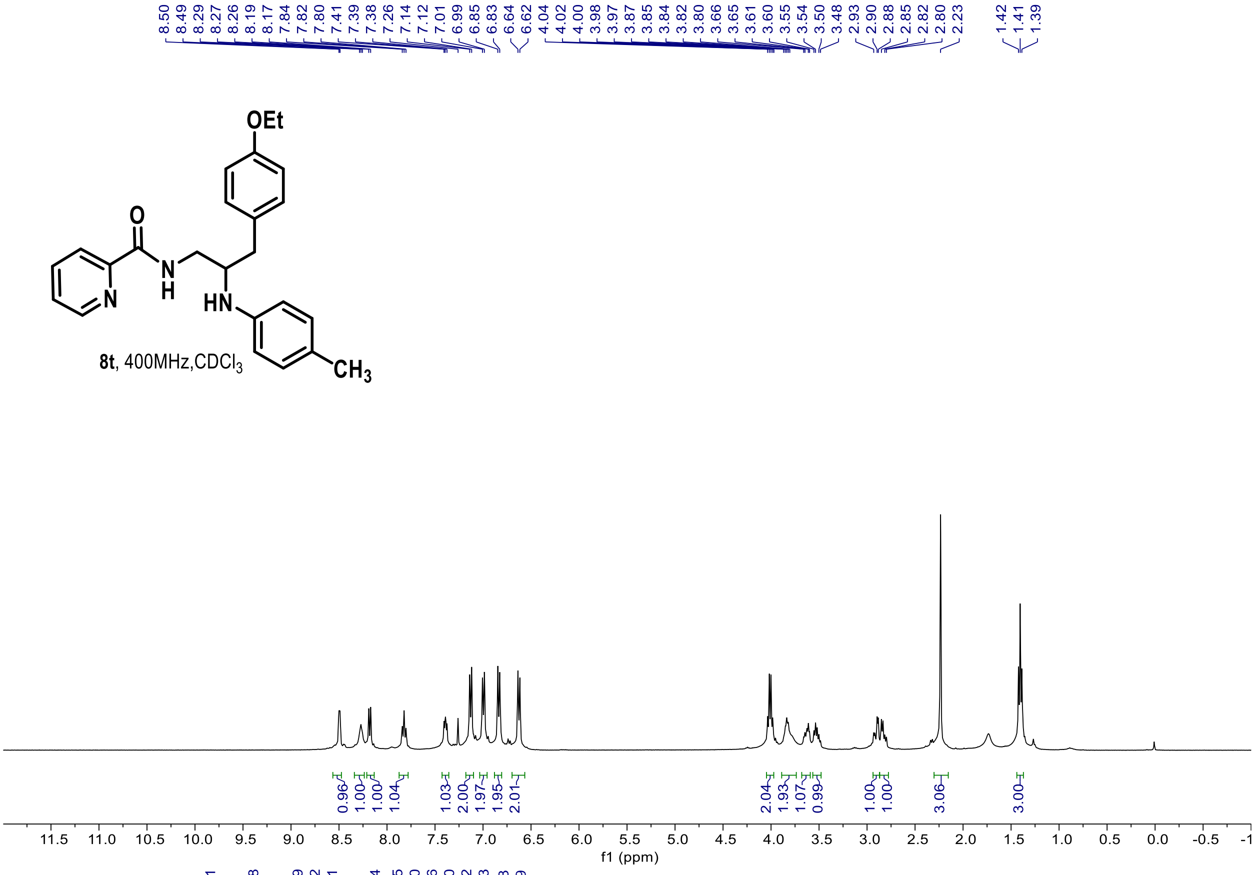
8.50
8.49
8.29
8.27
8.26
8.19
8.17
7.84
7.82
7.80
7.41
7.39
7.38
7.26
7.14
7.12
7.01
6.99
6.85
6.83
6.64
6.62
4.04
4.02
4.00
3.98
3.97
3.87
3.85
3.84
3.82
3.80
3.66
3.65
3.61
3.60
3.55
3.54
3.50
3.48
2.93
2.90
2.88
2.85
2.82
2.80
2.23
1.42
1.41
1.39
OEt
O
N
H
N
HN
CH3
8t, 400MHz,CDCl3
0.96
1.00
1.00
1.04
1.03
2.00
1.97
1.95
2.01
2.04
1.93
1.07
0.99
1.00
1.00
3.06
3.00
f1 (ppm)

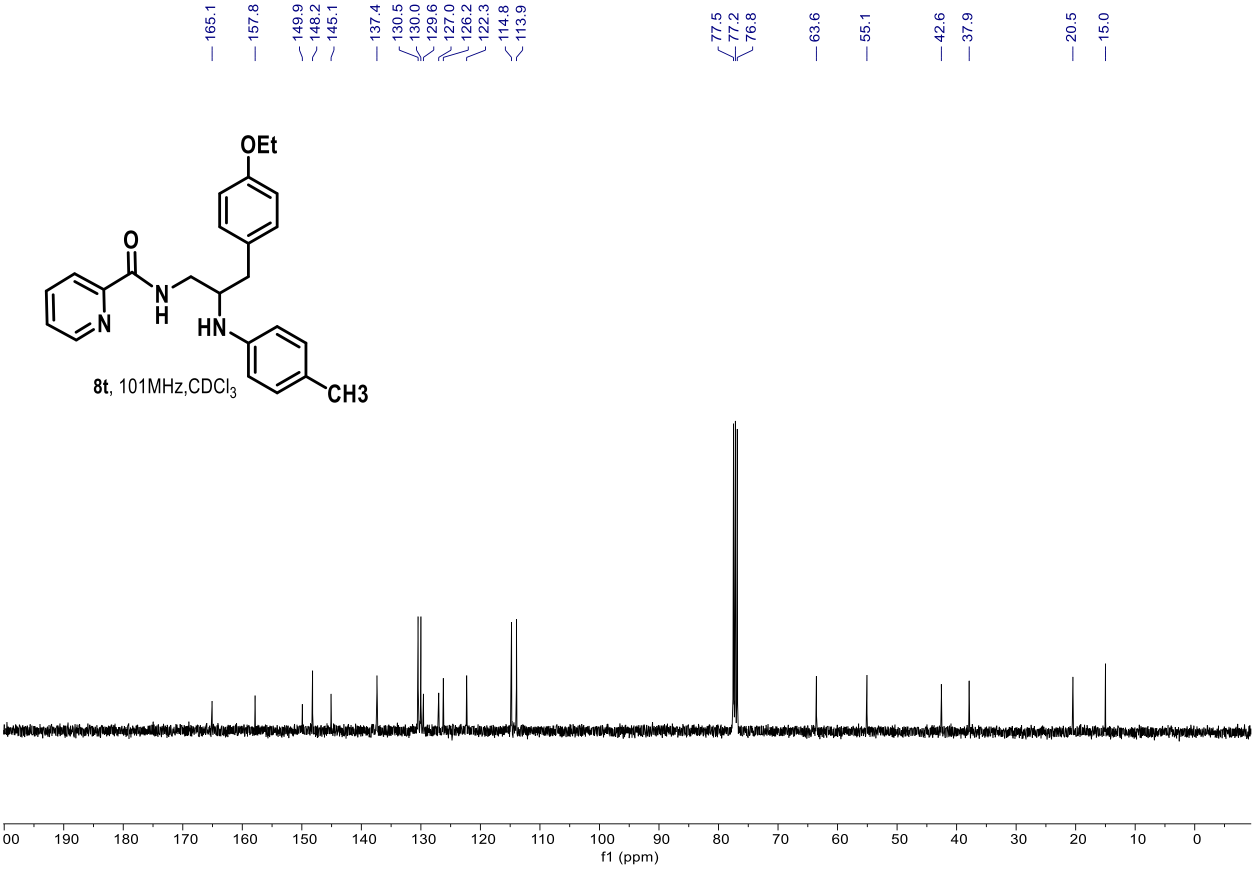
165.1
157.8
149.9
148.2
145.1
137.4
130.5
130.0
129.6
127.0
126.2
122.3
114.8
113.9
77.5
77.2
76.8
63.6
55.1
42.6
37.9
20.5
15.0
OEt
O
N
H
N
HN
CH3
8t, 101MHz,CDCl3
f1 (ppm)

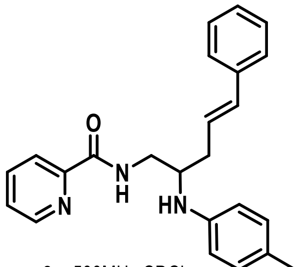

8u, 500MHz,CDCl$_3$

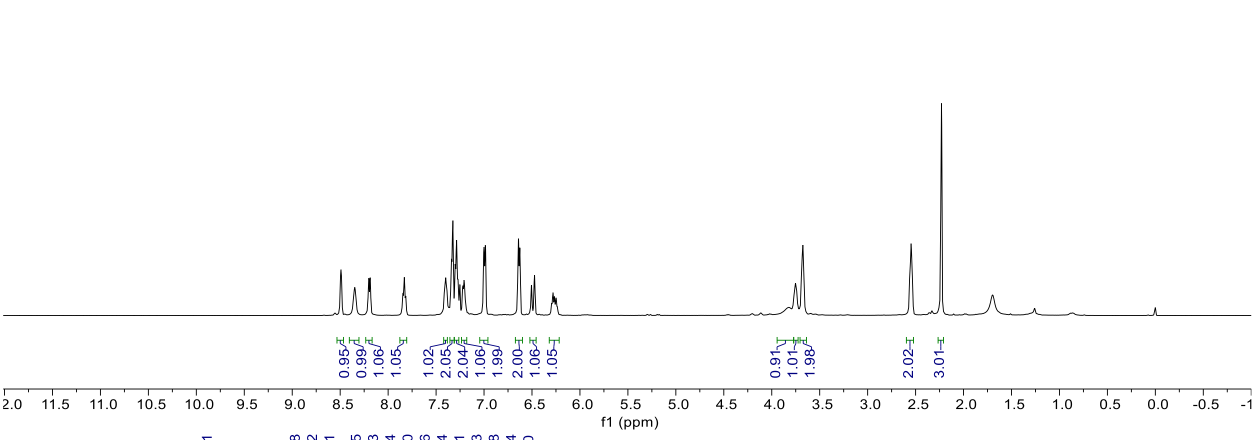

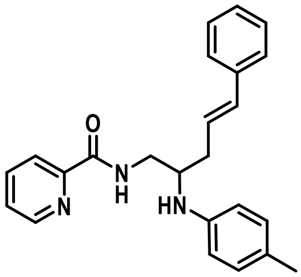

8u, 126MHz,CDCl$_3$

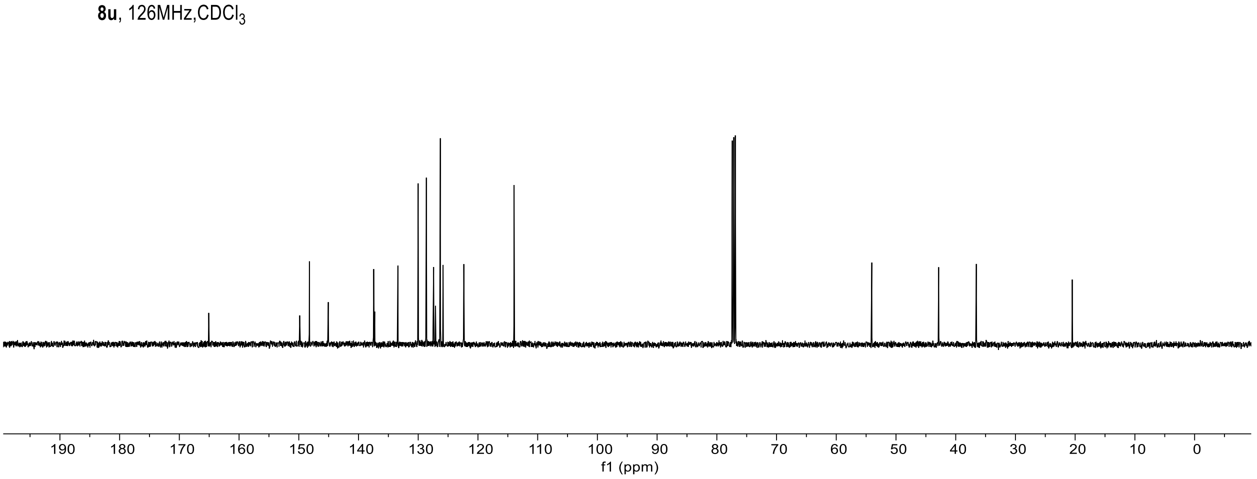

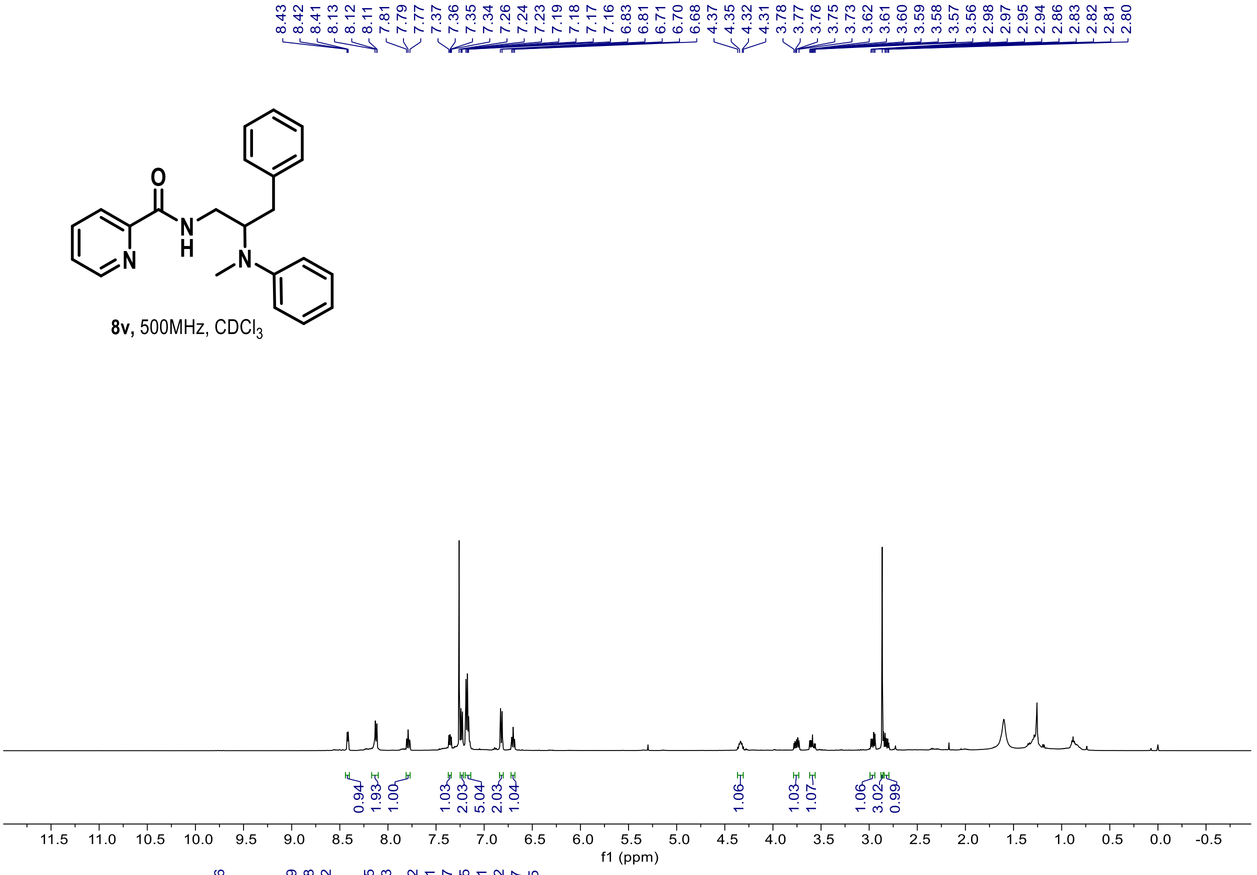

8v, 500MHz, $CDCl_3$

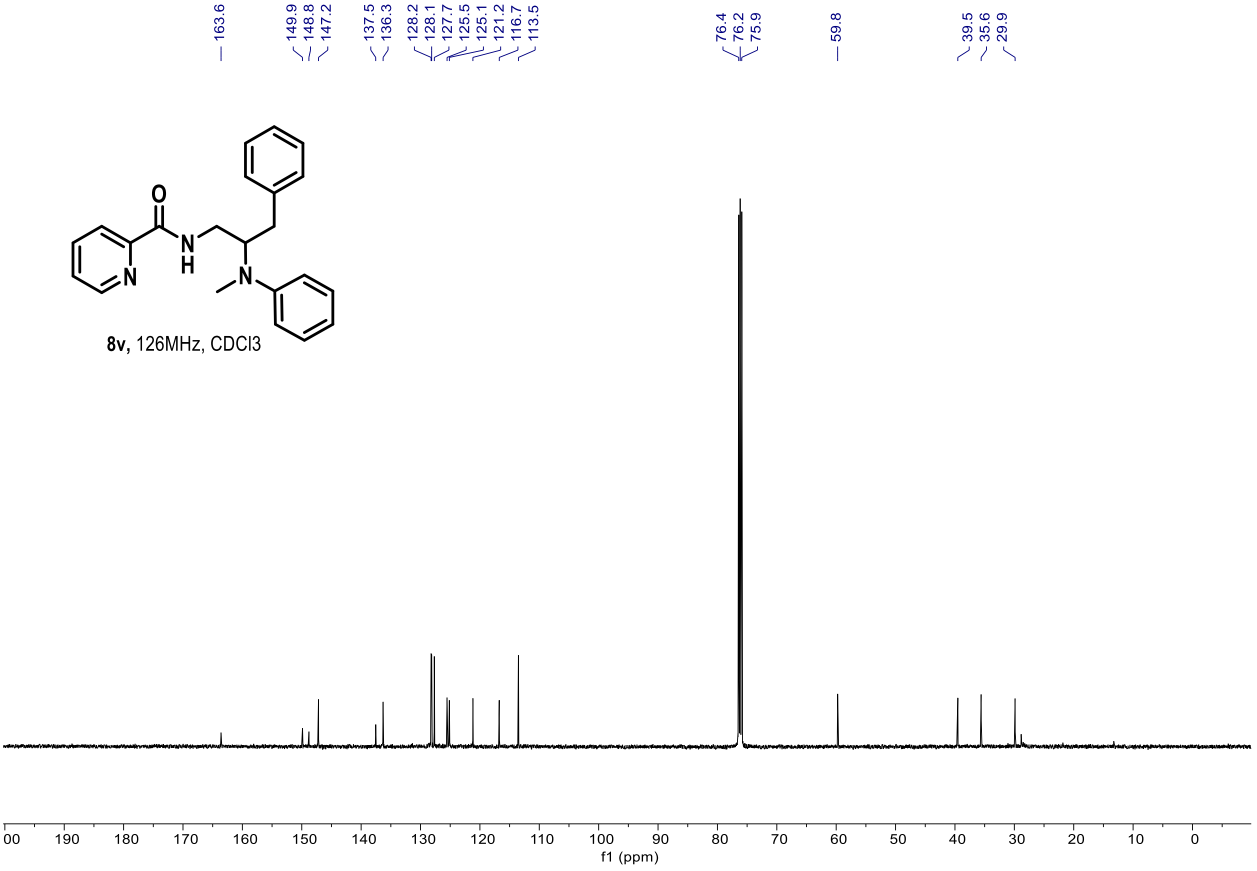

8v, 126MHz, CDCl3

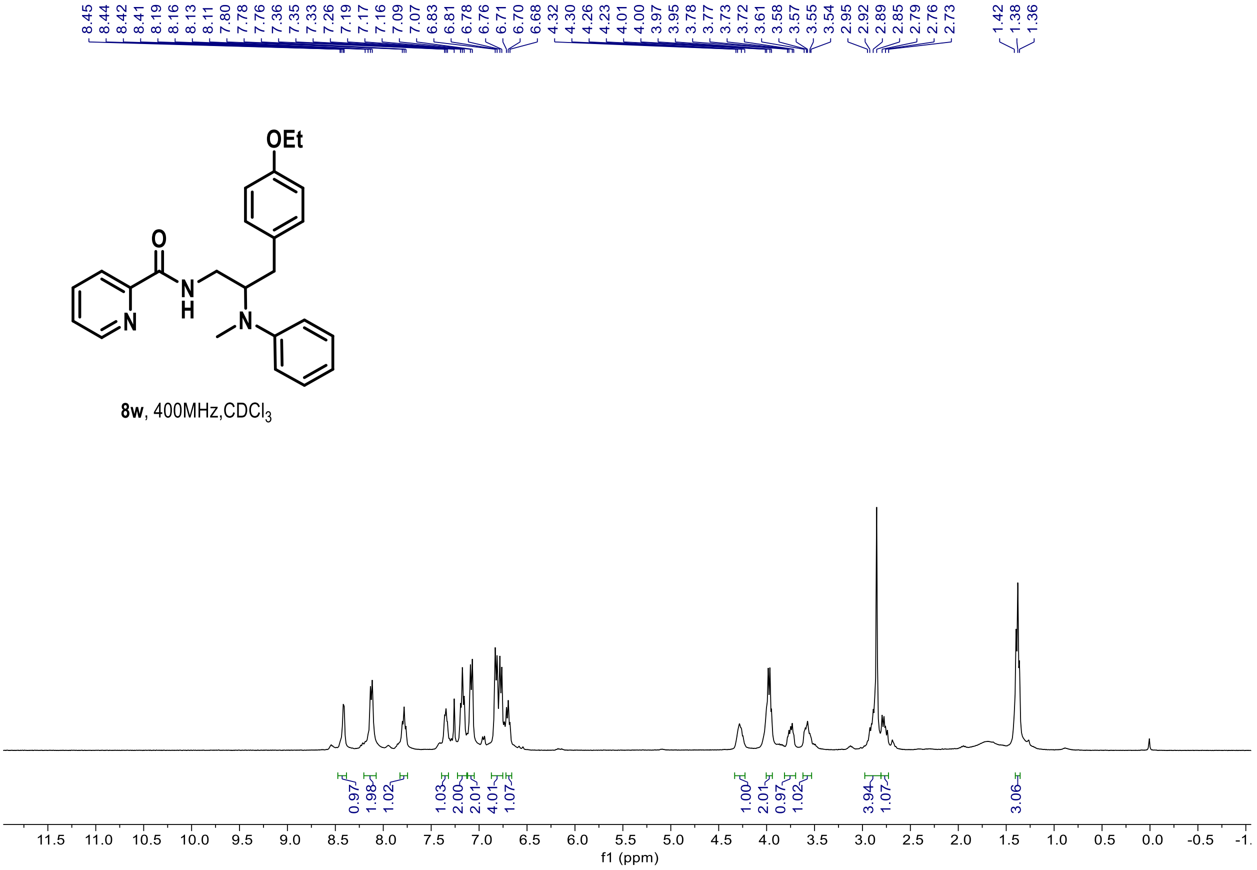
8.45 8.44 8.42 8.41 8.19 8.16 8.13 8.11 7.80 7.78 7.76 7.36 7.35 7.33 7.26 7.19 7.17 7.16 7.09 7.07 6.83 6.81 6.78 6.76 6.71 6.70 6.68 4.32 4.30 4.26 4.23 4.01 4.00 3.97 3.95 3.78 3.77 3.73 3.72 3.61 3.58 3.57 3.55 3.54 2.95 2.92 2.89 2.85 2.79 2.76 2.73 1.42 1.38 1.36
OEt
O
N
H
N
N
8w, 400MHz,CDCl3
0.97 1.98 1.02 1.03 2.00 2.01 4.01 1.07 1.00 2.01 0.97 1.02 3.94 1.07 3.06
11.5 11.0 10.5 10.0 9.5 9.0 8.5 8.0 7.5 7.0 6.5 6.0 5.5 5.0 4.5 4.0 3.5 3.0 2.5 2.0 1.5 1.0 0.5 0.0 -0.5 -1.
f1 (ppm)

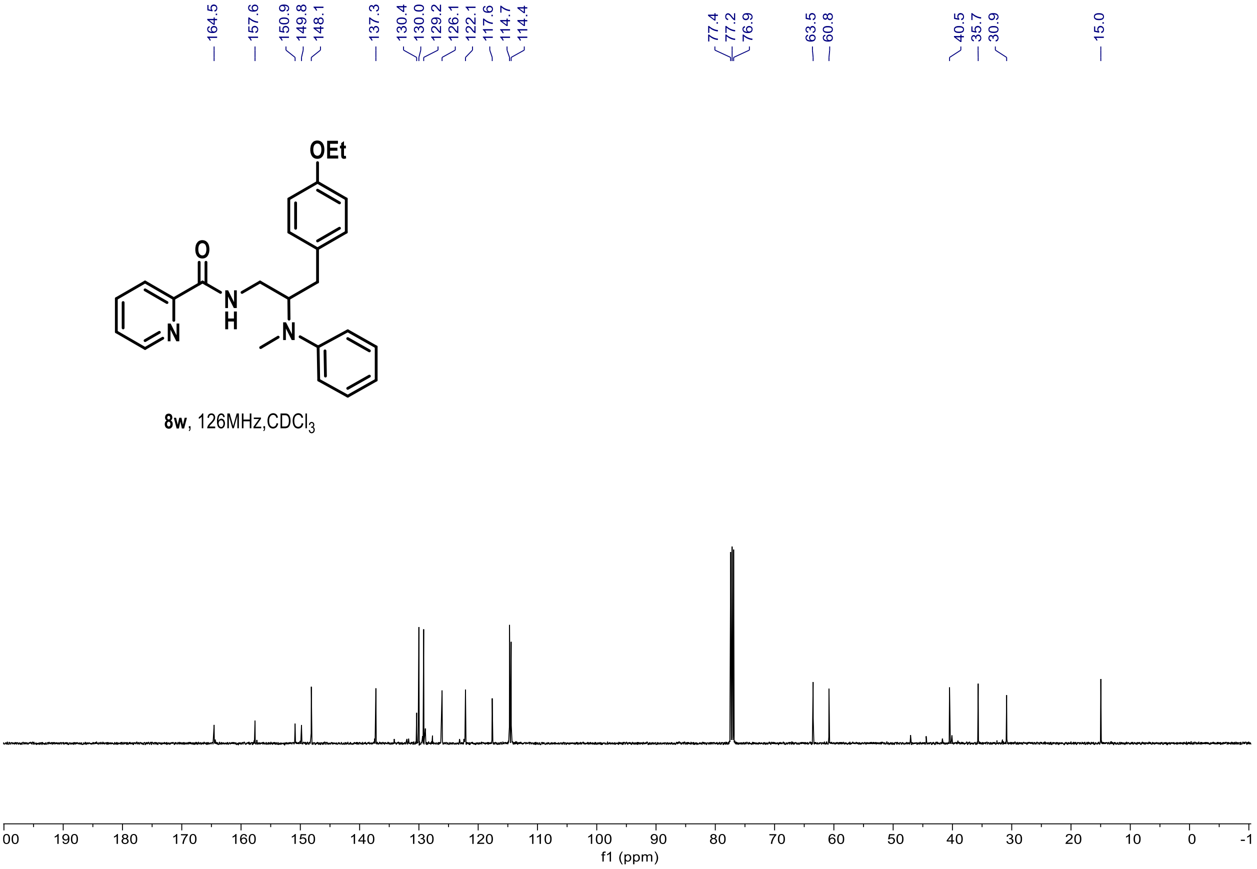
164.5 157.6 150.9 149.8 148.1 137.3 130.4 130.0 129.2 126.1 122.1 117.6 114.7 114.4 77.4 77.2 76.9 63.5 60.8 40.5 35.7 30.9 15.0
OEt
O
N
H
N
N
8w, 126MHz,CDCl3
00 190 180 170 160 150 140 130 120 110 100 90 80 70 60 50 40 30 20 10 0 -1
f1 (ppm)

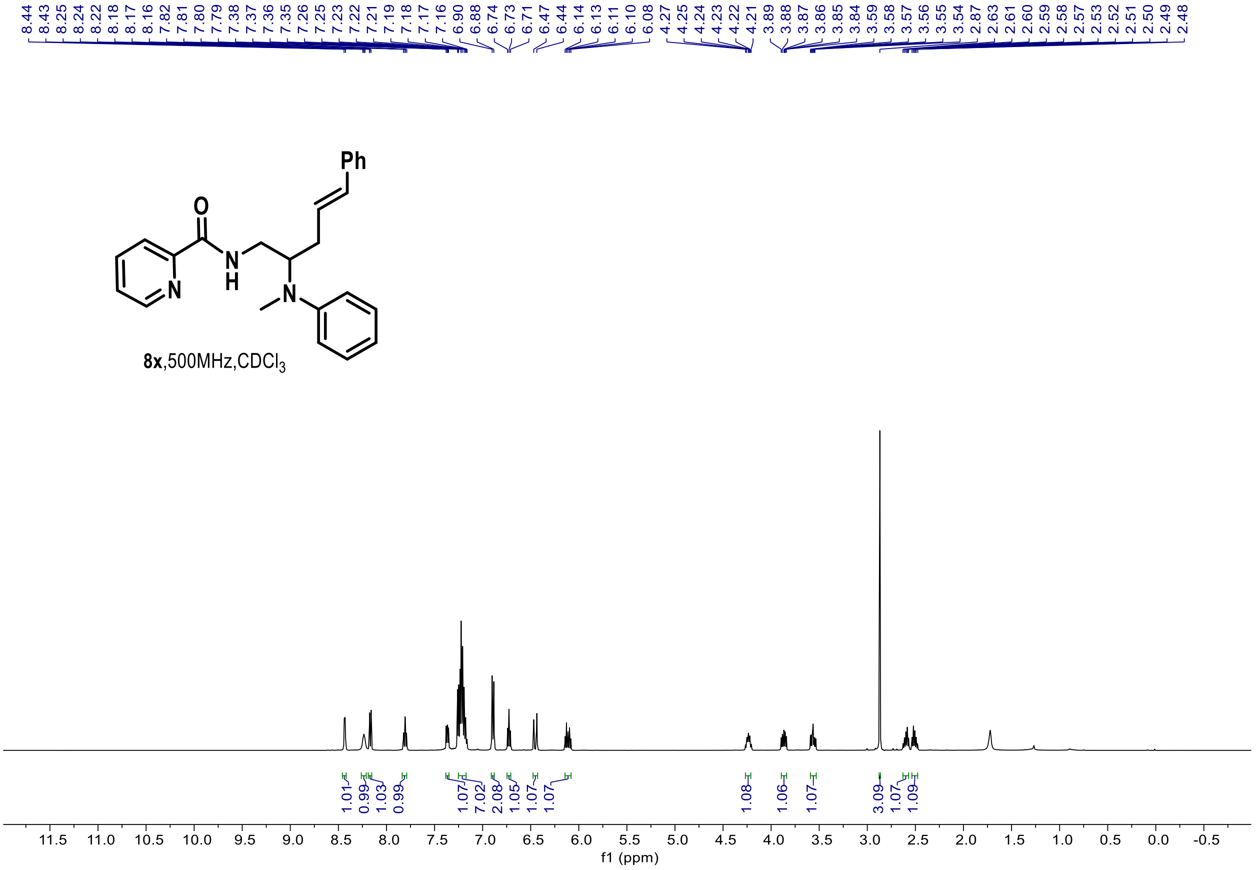

8x,500MHz,$CDCl_3$

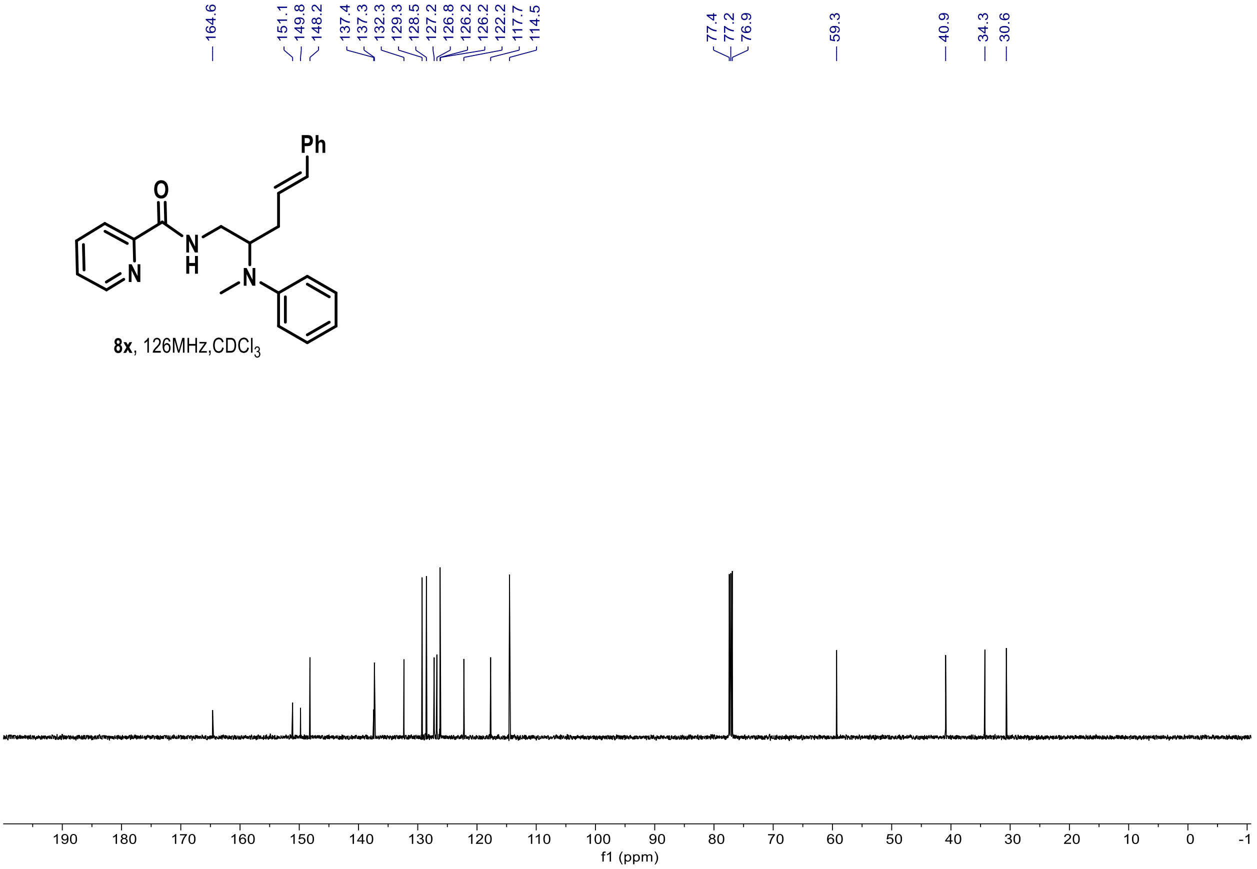

8x, 126MHz,$CDCl_3$

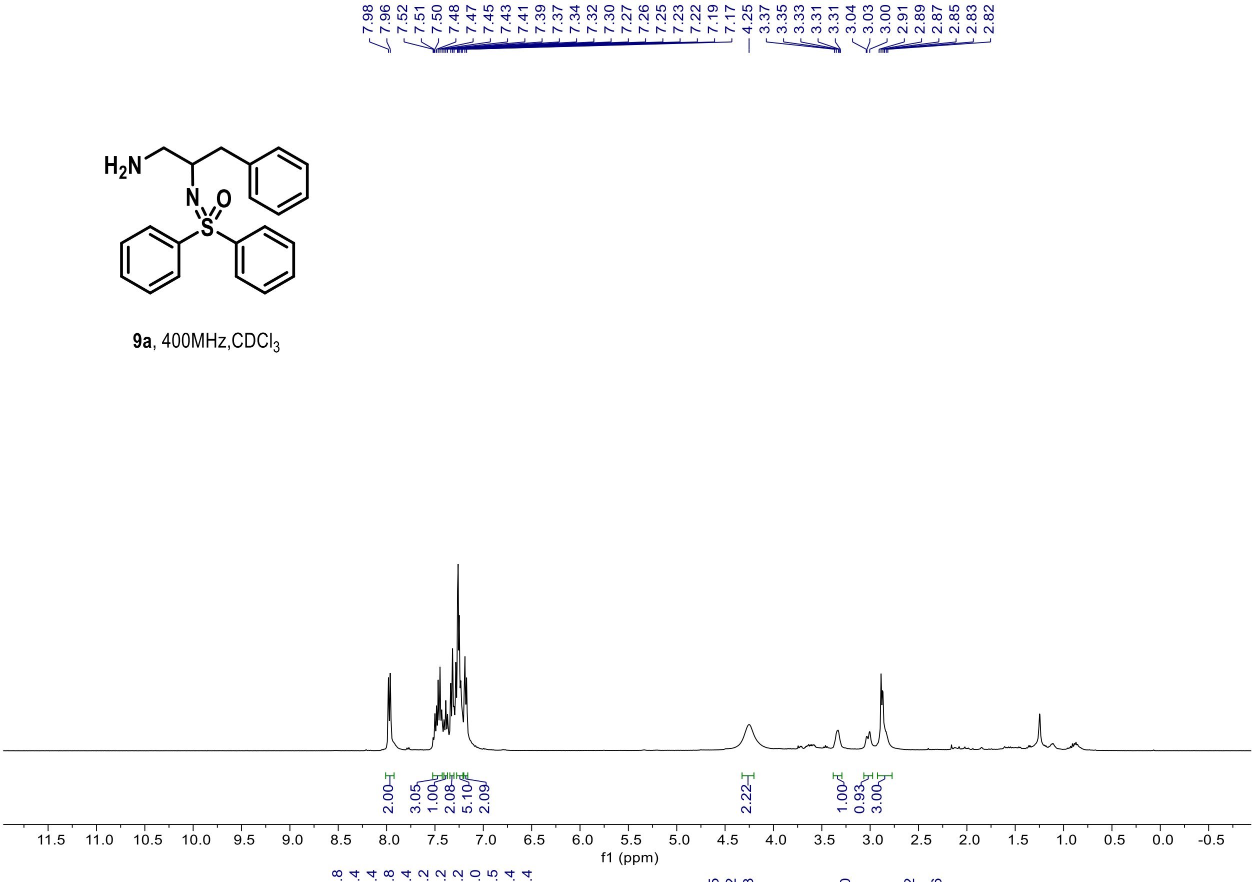

9a, 400MHz,$CDCl_3$

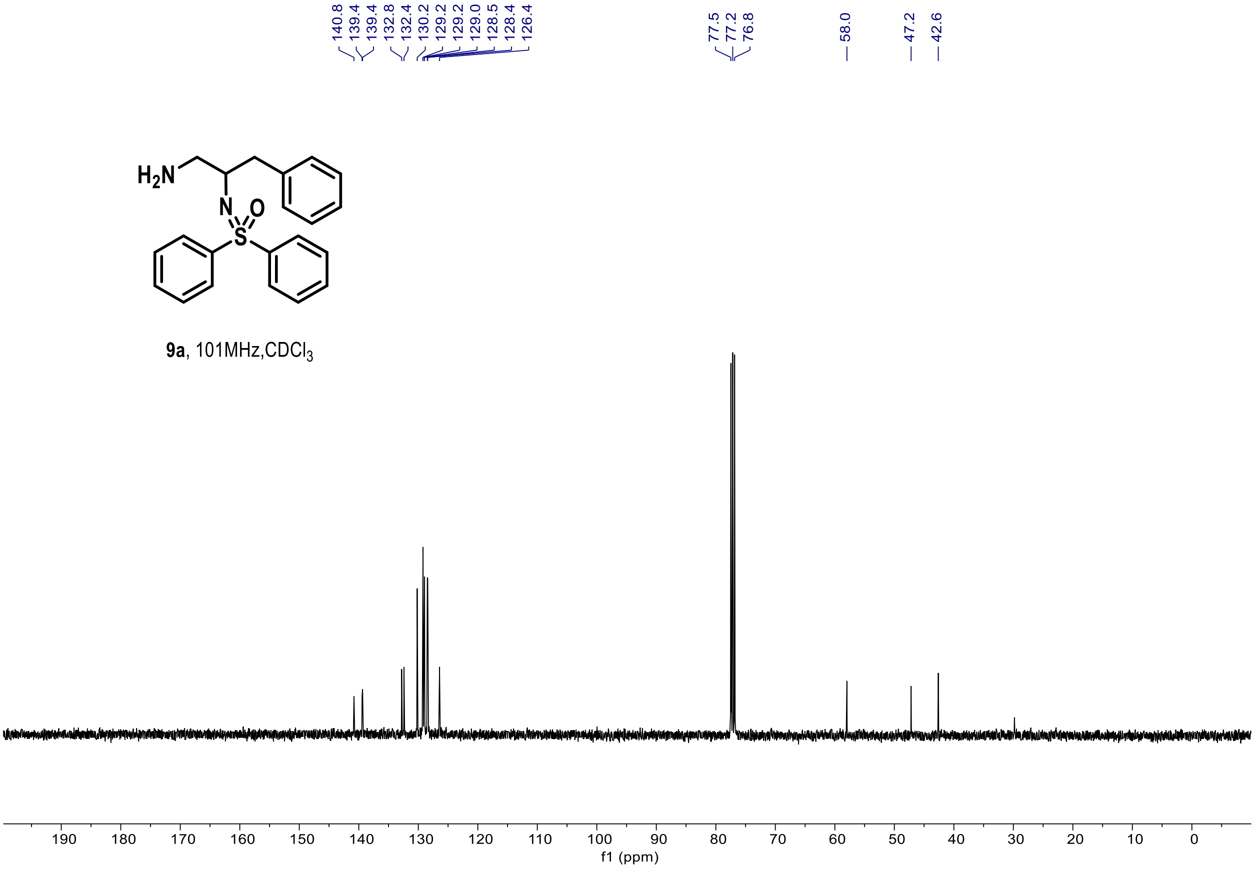

9a, 101MHz,$CDCl_3$

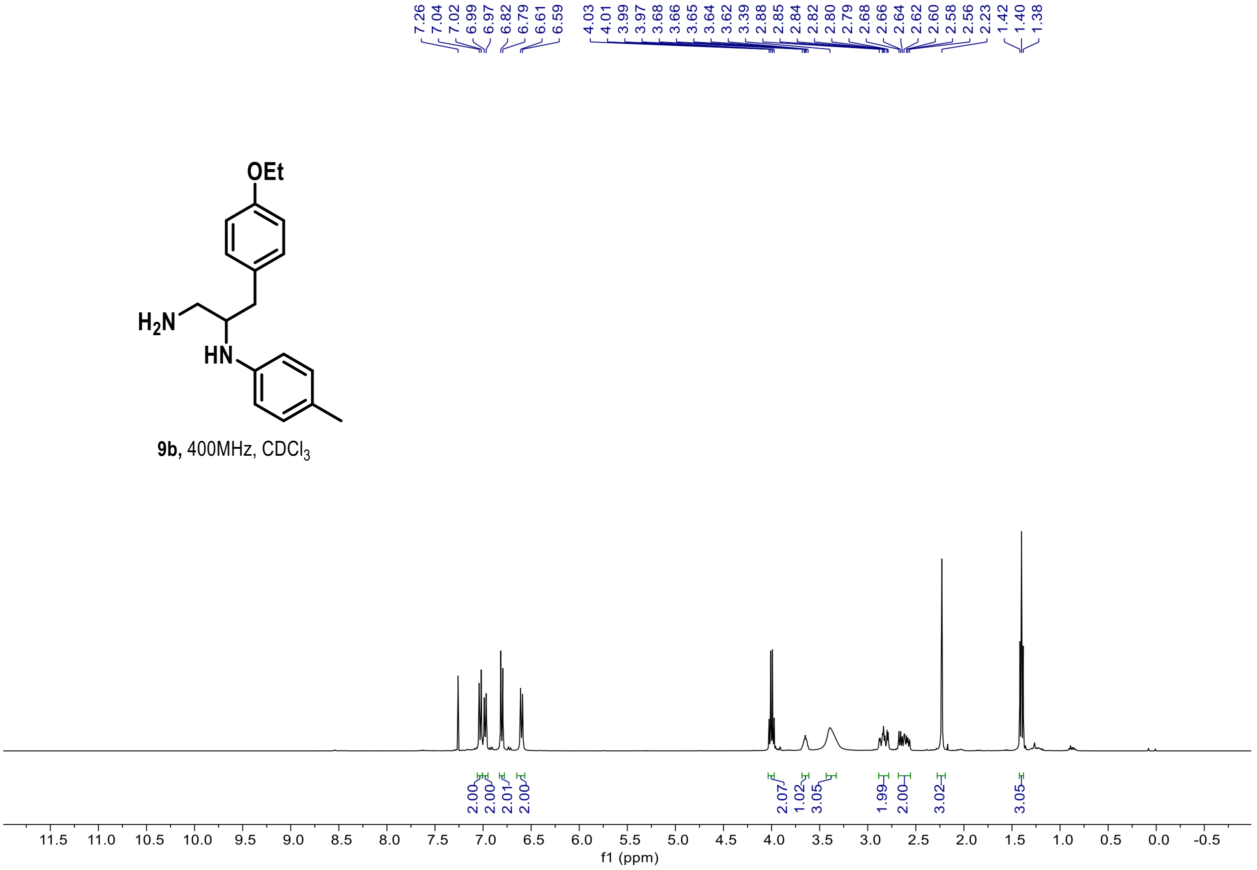

9b, 400MHz, $CDCl_3$

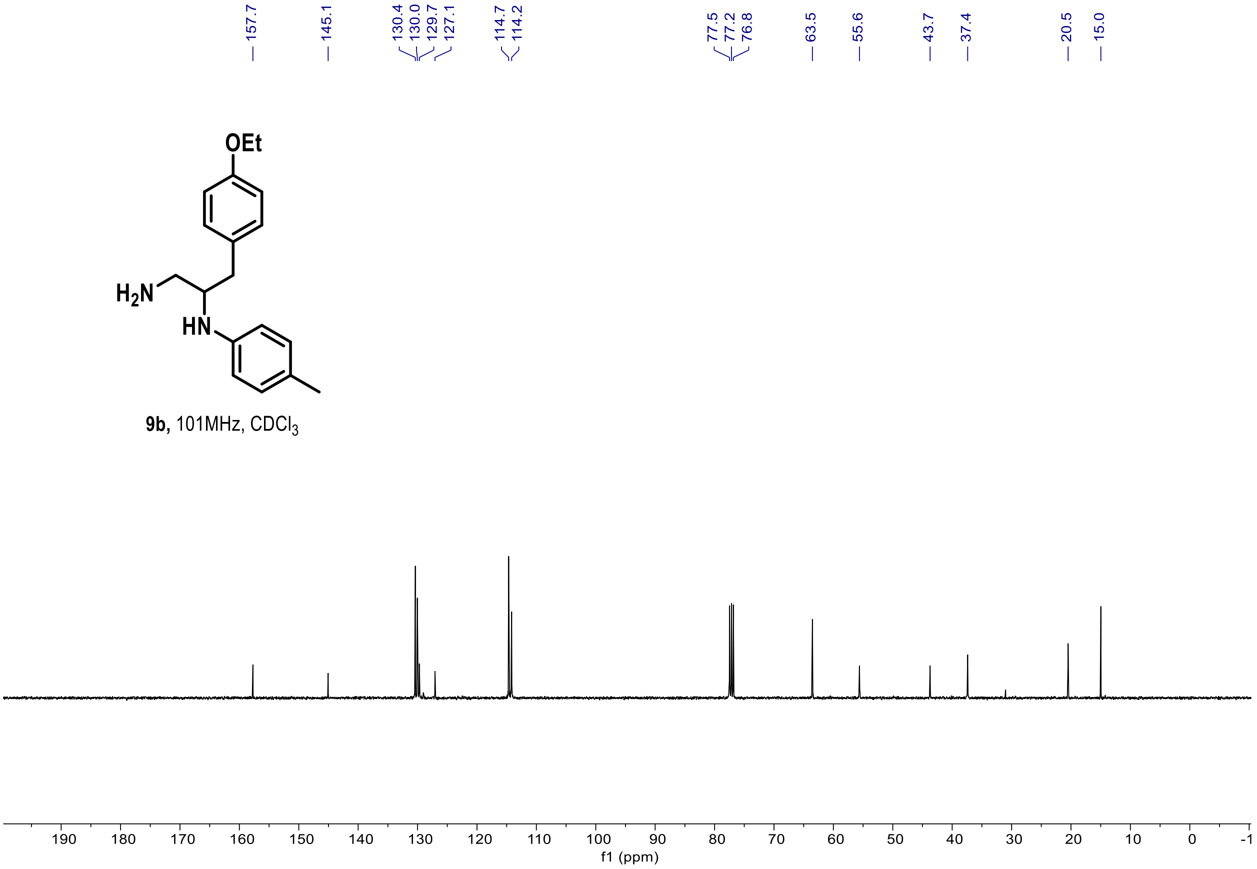

9b, 101MHz, $CDCl_3$

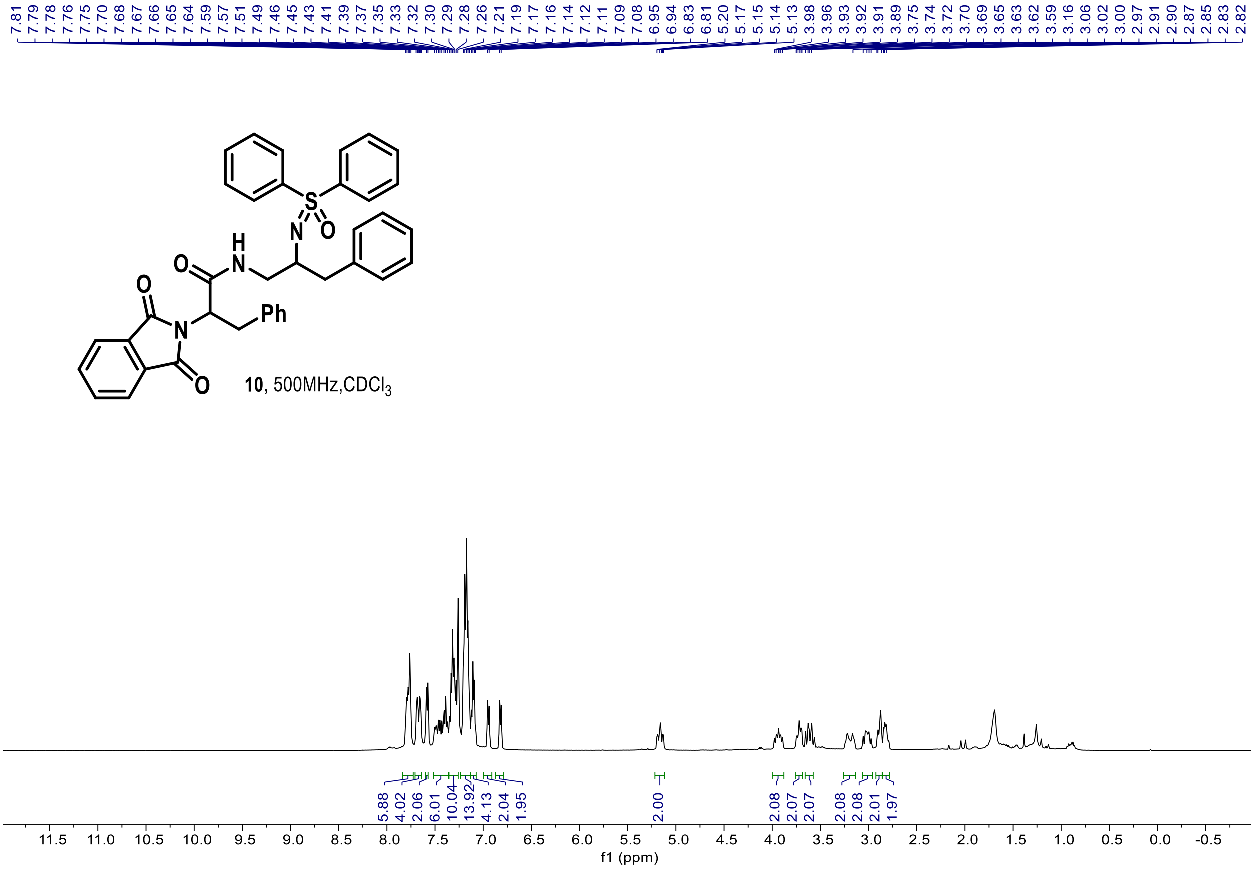
7.81 7.79 7.78 7.76 7.75 7.70 7.68 7.67 7.66 7.65 7.64 7.59 7.57 7.51 7.49 7.46 7.45 7.43 7.41 7.39 7.37 7.35 7.33 7.32 7.30 7.29 7.28 7.26 7.21 7.19 7.17 7.16 7.14 7.12 7.11 7.09 7.08 6.95 6.94 6.83 6.81 5.20 5.17 5.15 5.14 5.13 3.98 3.96 3.93 3.92 3.91 3.89 3.75 3.74 3.72 3.70 3.69 3.65 3.63 3.62 3.59 3.16 3.06 3.02 3.00 2.97 2.91 2.90 2.87 2.85 2.83 2.82
H
O
N
S
O
Ph
10, 500MHz,CDCl3
5.88 4.02 2.06 6.01 10.04 13.92 4.13 2.04 1.95
2.00
2.08 2.07 2.07
2.08 2.08 2.01 1.97
11.5 11.0 10.5 10.0 9.5 9.0 8.5 8.0 7.5 7.0 6.5 6.0 5.5 5.0 4.5 4.0 3.5 3.0 2.5 2.0 1.5 1.0 0.5 0.0 -0.5
f1 (ppm)

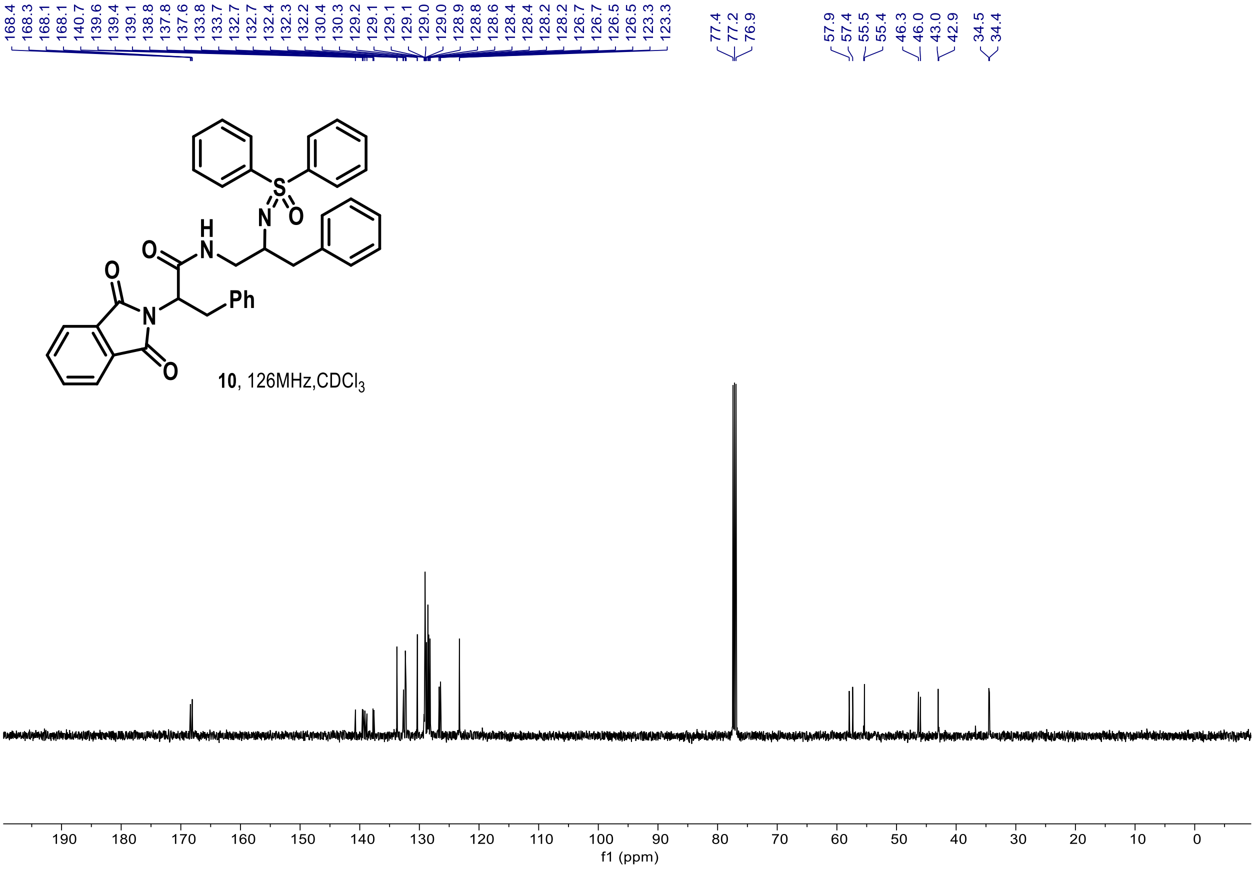
168.4 168.3 168.1 168.1 140.7 139.6 139.4 139.1 138.8 137.8 137.6 133.8 133.7 132.7 132.4 132.3 132.2 130.4 130.3 129.2 129.1 129.1 129.1 129.0 129.0 128.9 128.8 128.6 128.4 128.2 128.2 126.7 126.7 126.5 126.5 123.3 123.3
77.4 77.2 76.9
57.9 57.4 55.5 55.4 46.3 46.0 43.0 42.9 34.5 34.4
H
O
N
S
O
Ph
10, 126MHz,CDCl3
190 180 170 160 150 140 130 120 110 100 90 80 70 60 50 40 30 20 10 0
f1 (ppm)

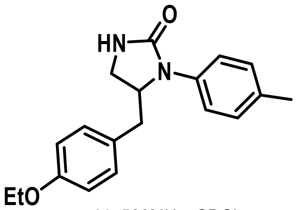

11, 500MHz, $CDCl_3$

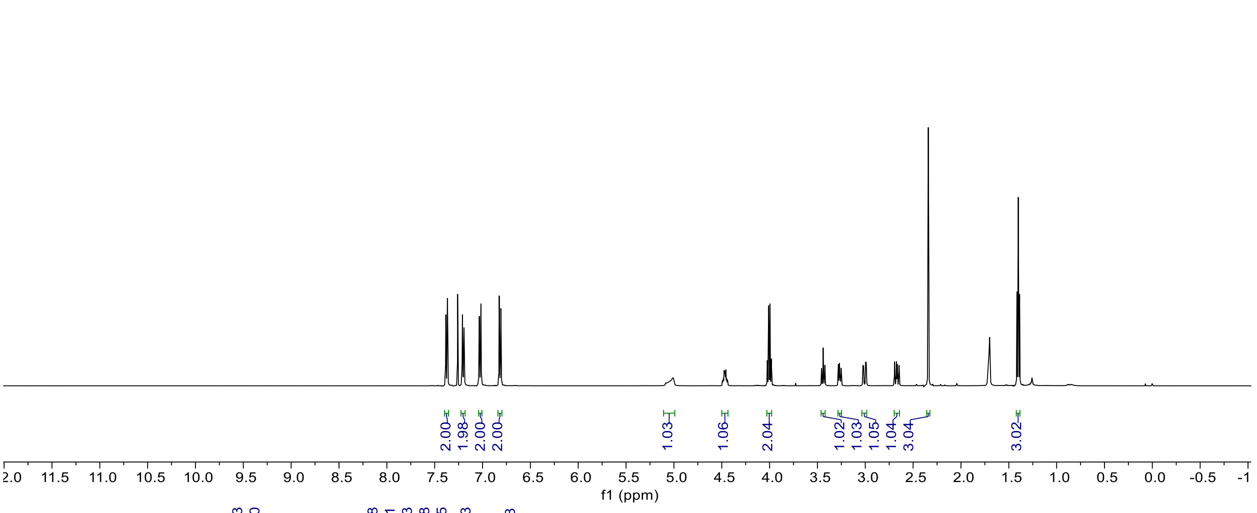

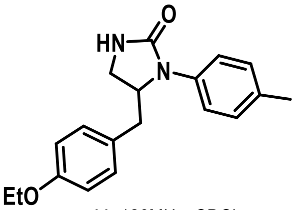

11, 126MHz, $CDCl_3$

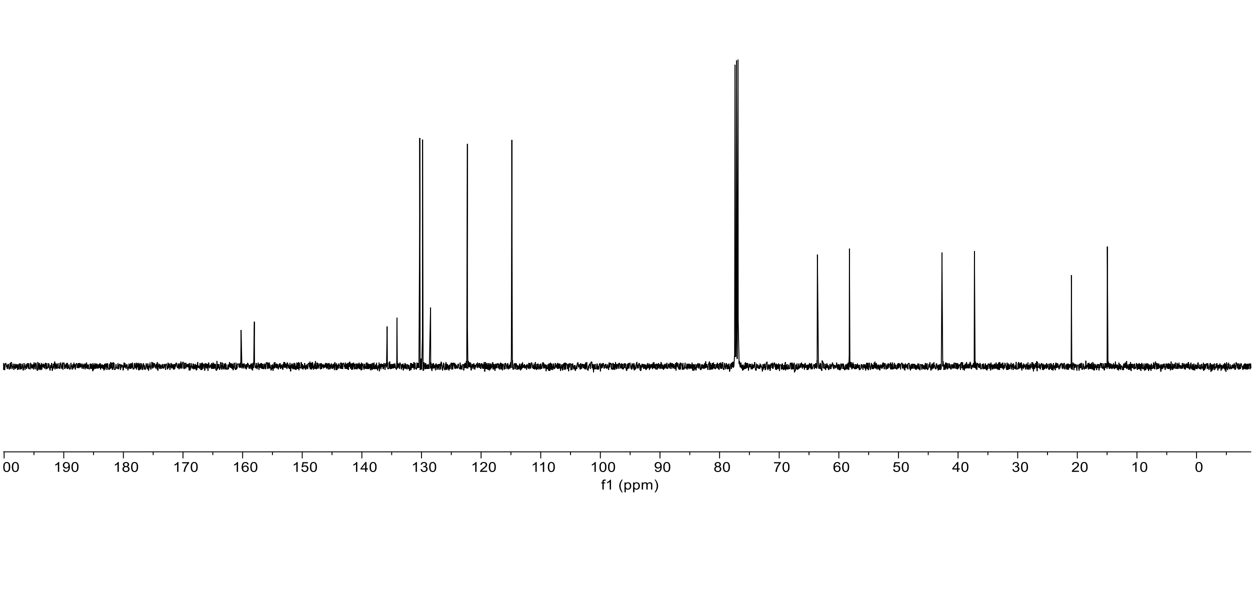

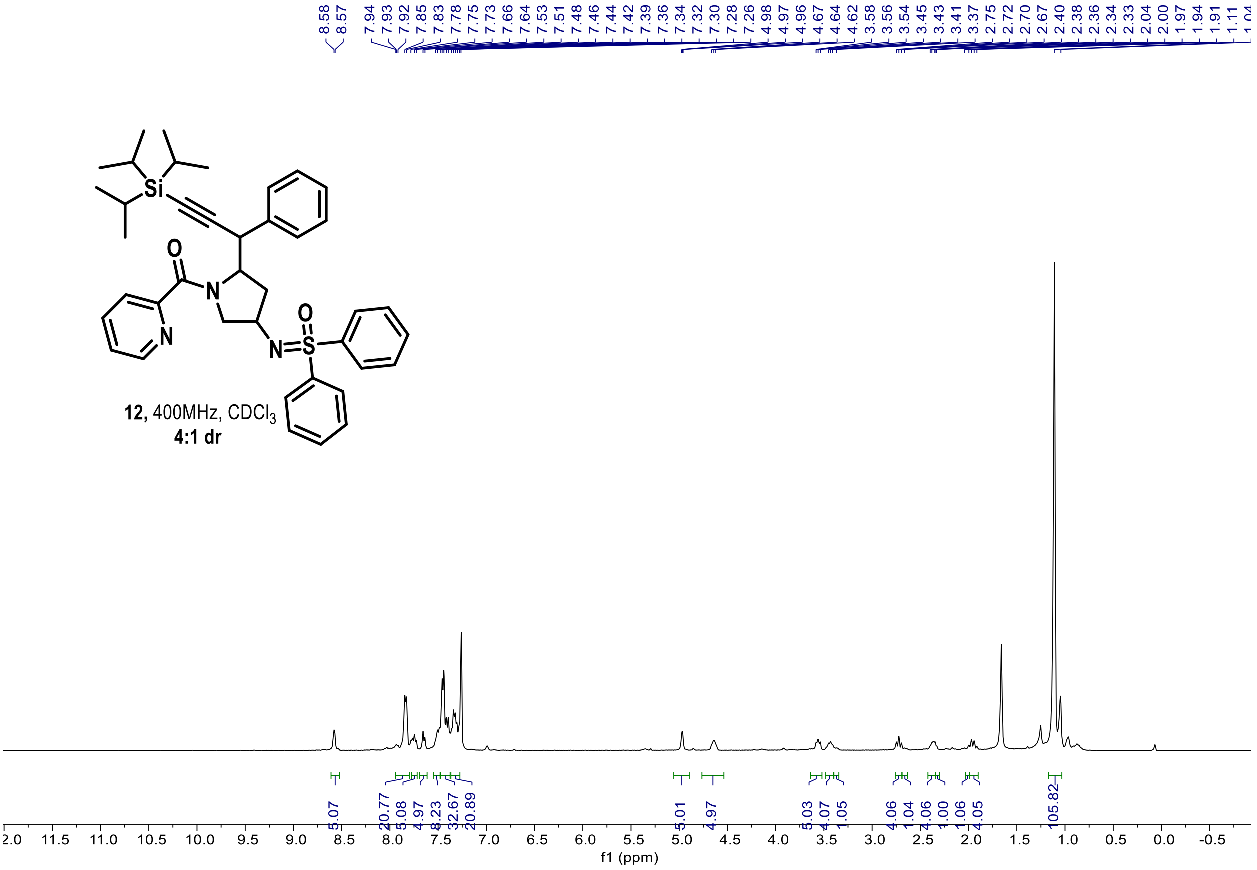
8.58 8.57 7.94 7.93 7.92 7.85 7.83 7.78 7.75 7.73 7.66 7.64 7.53 7.51 7.48 7.46 7.44 7.42 7.39 7.36 7.34 7.32 7.30 7.28 7.26 4.98 4.97 4.96 4.67 4.64 4.62 3.58 3.56 3.54 3.45 3.43 3.41 3.37 2.75 2.72 2.70 2.67 2.40 2.38 2.36 2.34 2.33 2.04 2.00 1.97 1.94 1.91 1.11
12, 400MHz, CDCl3
4:1 dr
5.07 20.77 5.08 4.97 8.23 32.67 20.89 5.01 4.97 5.03 4.07 1.05 4.06 1.04 4.06 1.00 1.06 4.05 105.82
f1 (ppm)
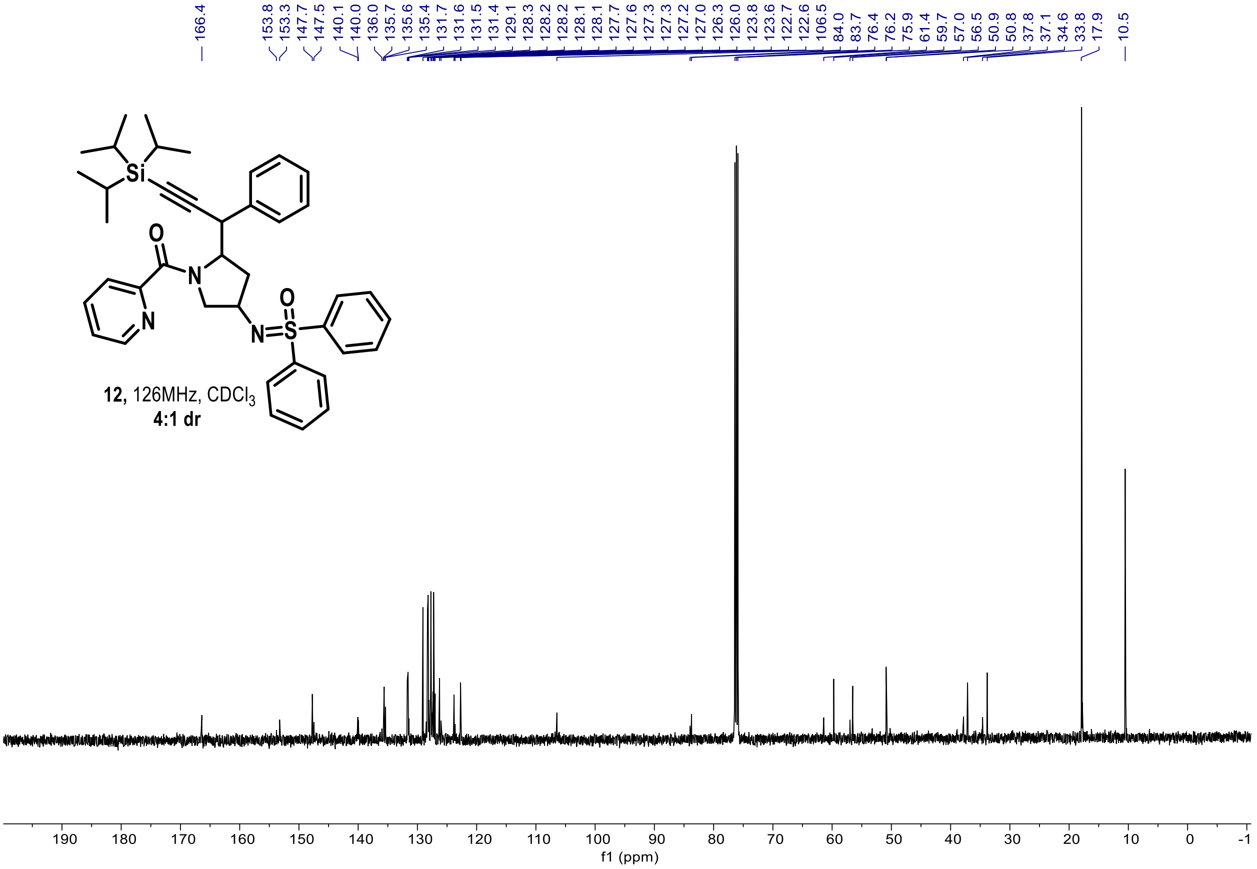
166.4 153.8 153.3 147.7 147.5 140.1 140.0 136.0 135.7 135.6 135.4 131.7 131.6 131.5 131.4 129.1 128.3 128.2 128.2 128.1 128.1 127.7 127.6 127.3 127.3 127.2 127.0 126.3 126.0 123.8 123.6 122.7 122.6 106.5 84.0 83.7 76.4 76.2 75.9 61.4 59.7 57.0 56.5 50.9 50.8 37.8 37.1 34.6 33.8 17.9 10.5
12, 126MHz, CDCl3
4:1 dr
f1 (ppm)

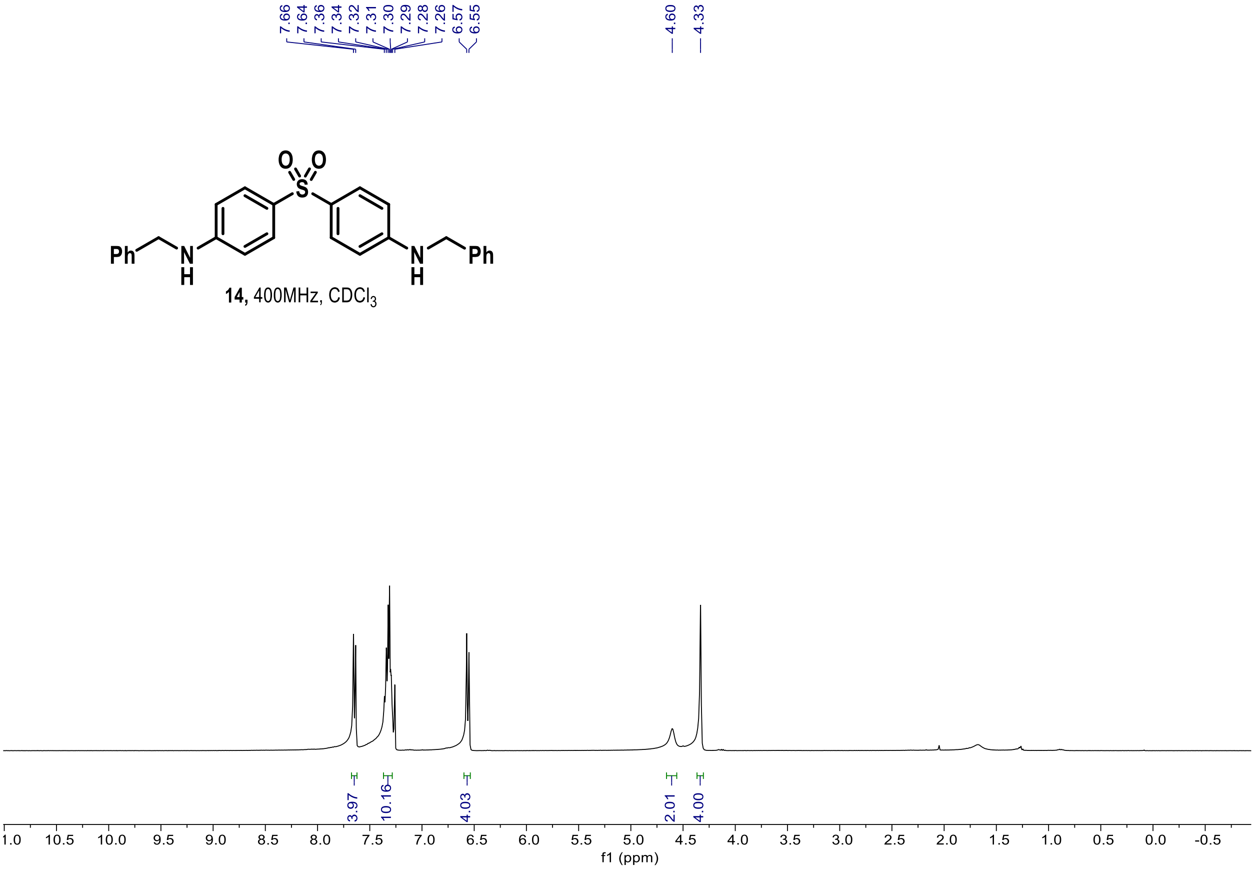
7.66 7.64 7.36 7.34 7.32 7.31 7.30 7.29 7.28 7.26 6.57 6.55
4.60
4.33
14, 400MHz, CDCl3
3.97
10.16
4.03
2.01
4.00
f1 (ppm)

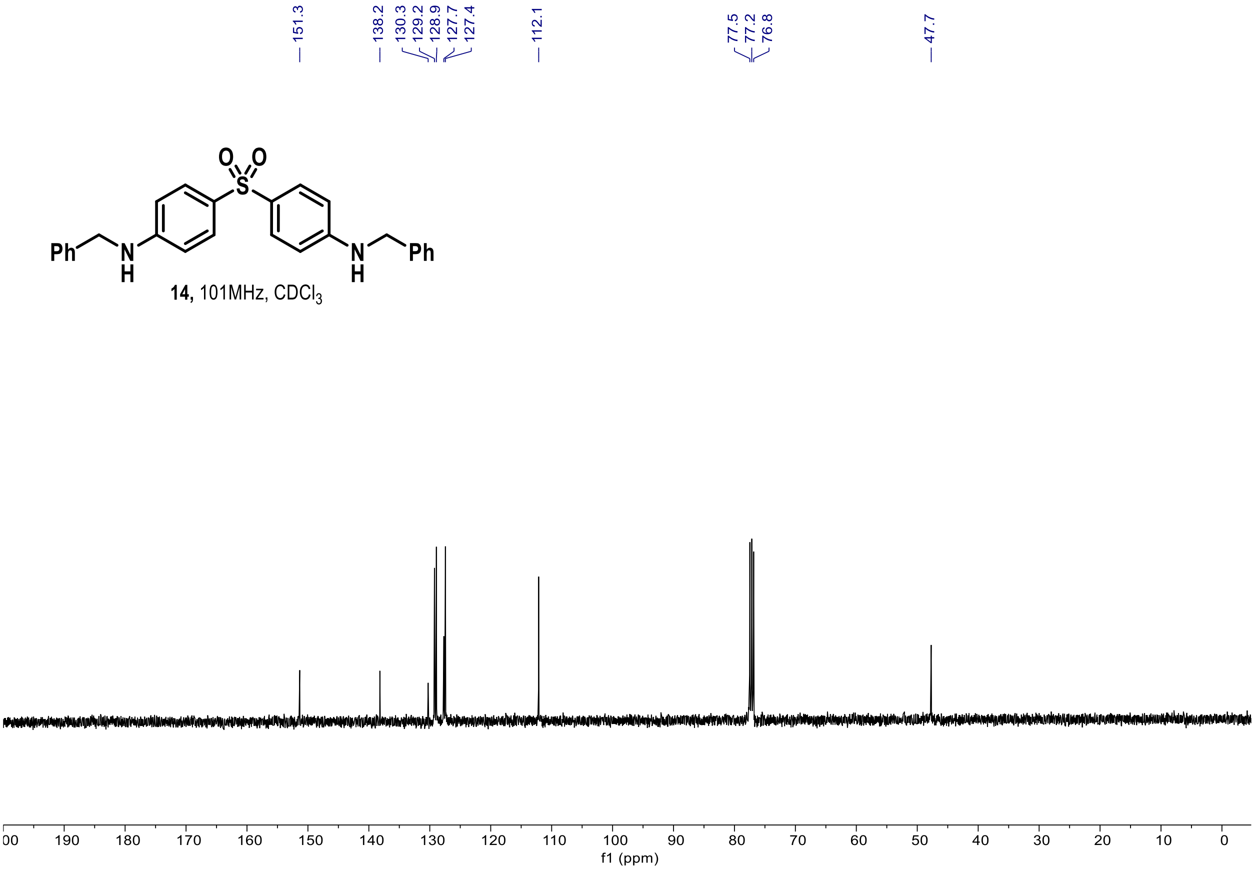
151.3
138.2 130.3 129.2 128.9 127.7 127.4
112.1
77.5 77.2 76.8
47.7
14, 101MHz, CDCl3
f1 (ppm)

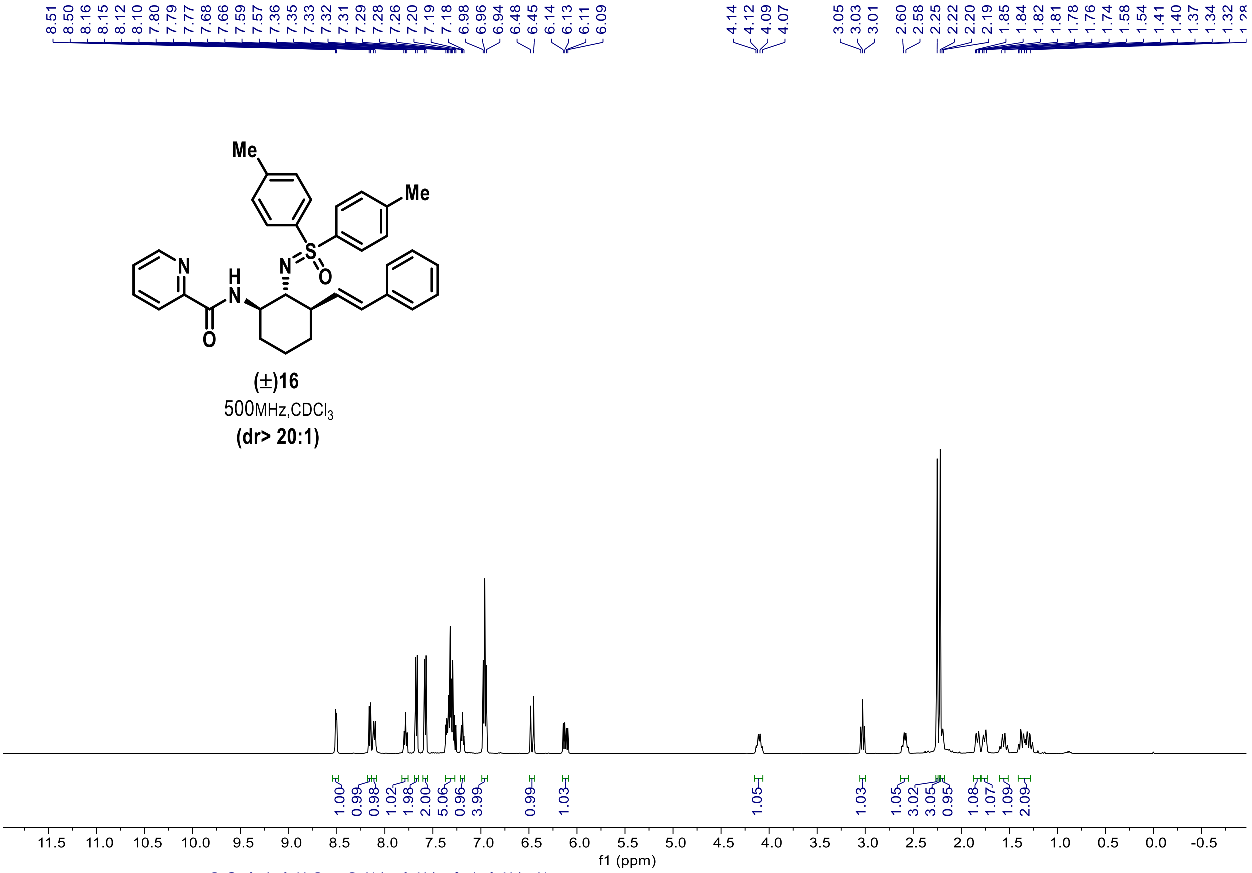
8.51 8.50 8.16 8.15 8.12 8.10 7.80 7.79 7.77 7.68 7.66 7.59 7.57 7.36 7.35 7.33 7.32 7.31 7.29 7.28 7.26 7.20 7.19 7.18 6.98 6.96 6.94 6.48 6.45 6.14 6.13 6.11 6.09
4.14 4.12 4.09 4.07
3.05 3.03 3.01 2.60 2.58 2.25 2.22 2.20 2.19 1.85 1.84 1.82 1.81 1.78 1.76 1.74 1.58 1.54 1.41 1.40 1.37 1.34 1.32
Me
Me
N
H
N
N
S
O
O
(±)16
500MHz,CDCl3
(dr> 20:1)
1.00 0.99 0.98 1.02 1.98 2.00 5.06 0.96 3.99 0.99 1.03 1.05 1.03 1.05 3.02 3.05 0.95 1.08 1.07 1.09 2.09
11.5 11.0 10.5 10.0 9.5 9.0 8.5 8.0 7.5 7.0 6.5 6.0 5.5 5.0 4.5 4.0 3.5 3.0 2.5 2.0 1.5 1.0 0.5 0.0 -0.5
f1 (ppm)

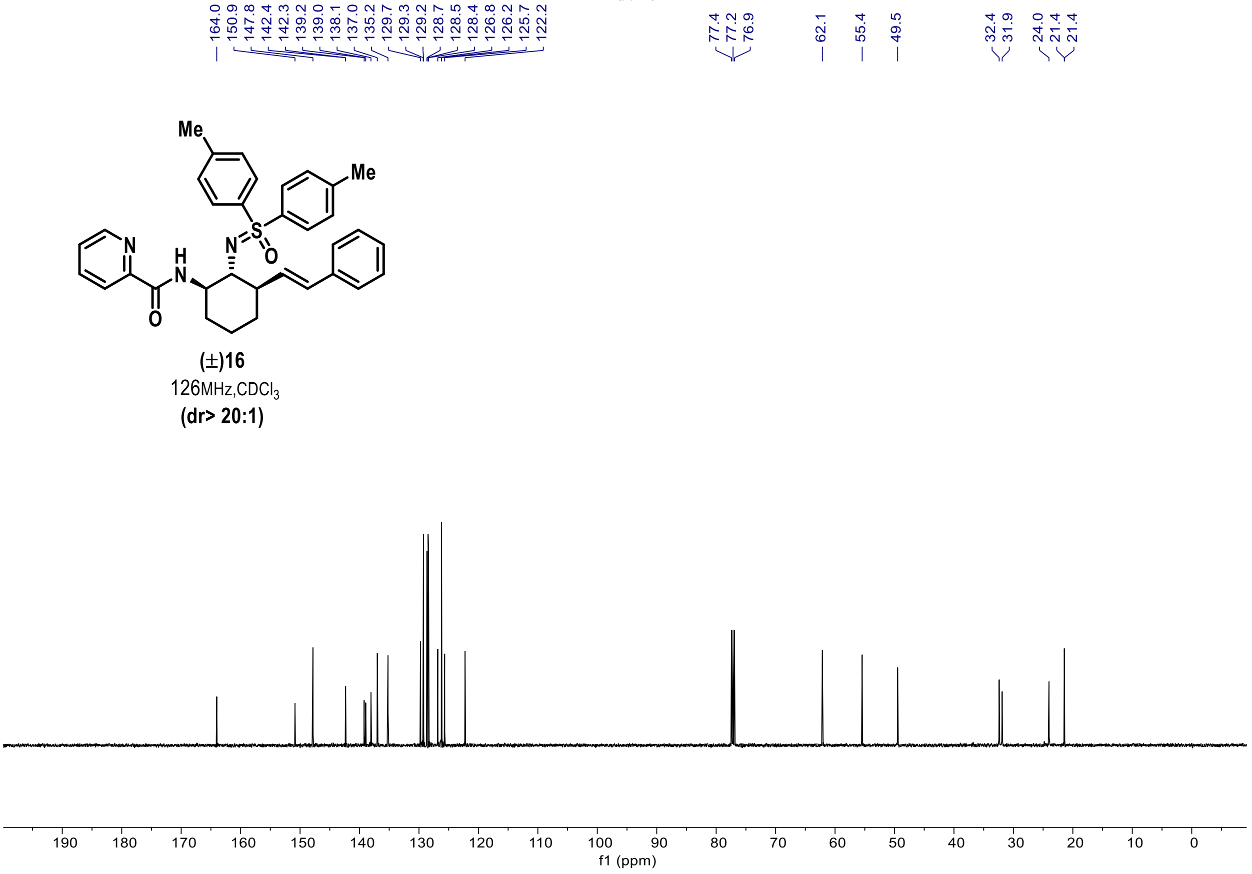
164.0 150.9 147.8 142.4 142.3 139.2 139.0 138.1 137.0 135.2 129.7 129.3 129.2 128.7 128.5 128.4 126.8 126.2 125.7 122.2
77.4 77.2 76.9
62.1
55.4
49.5
32.4 31.9
24.0 21.4 21.4
Me
Me
N
H
N
N
S
O
O
(±)16
126MHz,CDCl3
(dr> 20:1)
190 180 170 160 150 140 130 120 110 100 90 80 70 60 50 40 30 20 10 0
f1 (ppm)

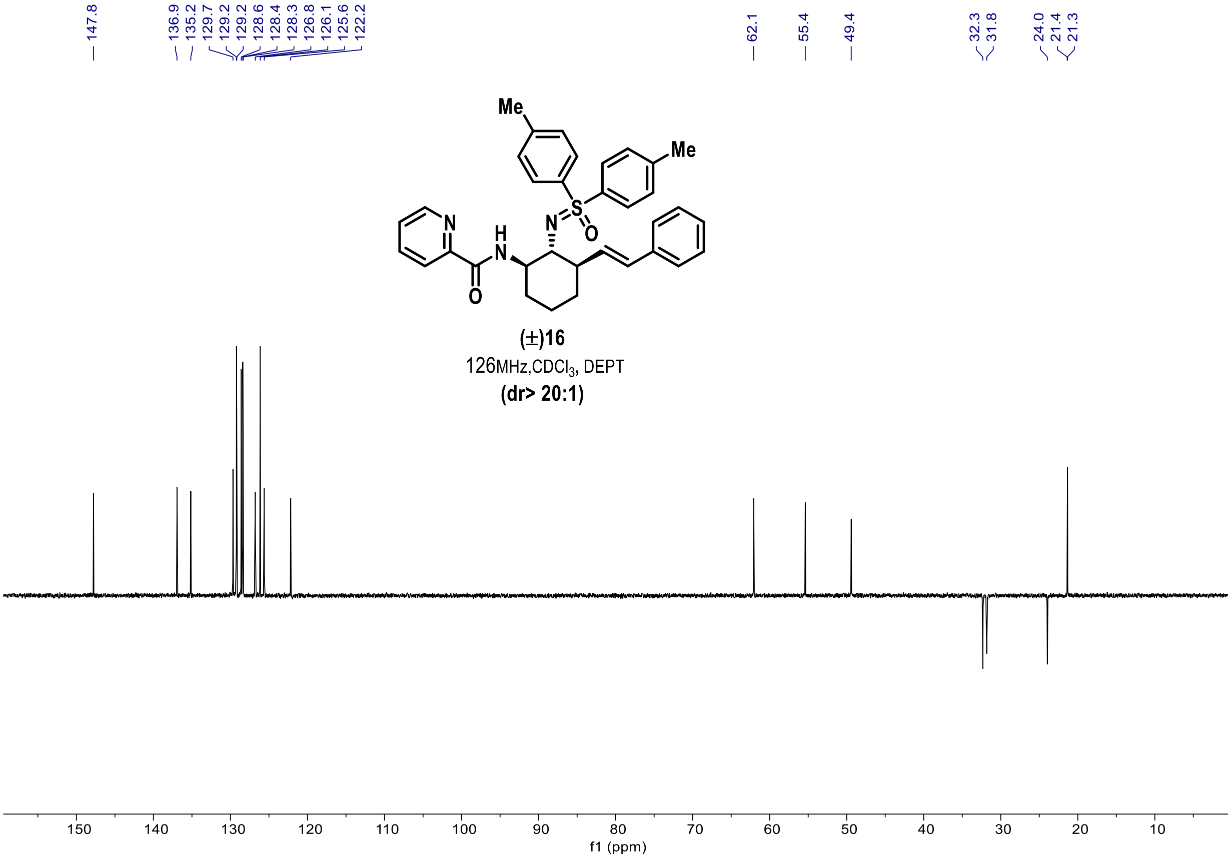
147.8
136.9
135.2
129.7
129.2
129.2
128.6
128.4
128.3
126.8
126.1
125.6
122.2
62.1
55.4
49.4
32.3
31.8
24.0
21.4
21.3
Me
Me
N
H
N
N
S
O
O
(±)16
126MHz,CDCl3, DEPT
(dr> 20:1)
150
140
130
120
110
100
90
80
70
60
50
40
30
20
10
f1 (ppm)

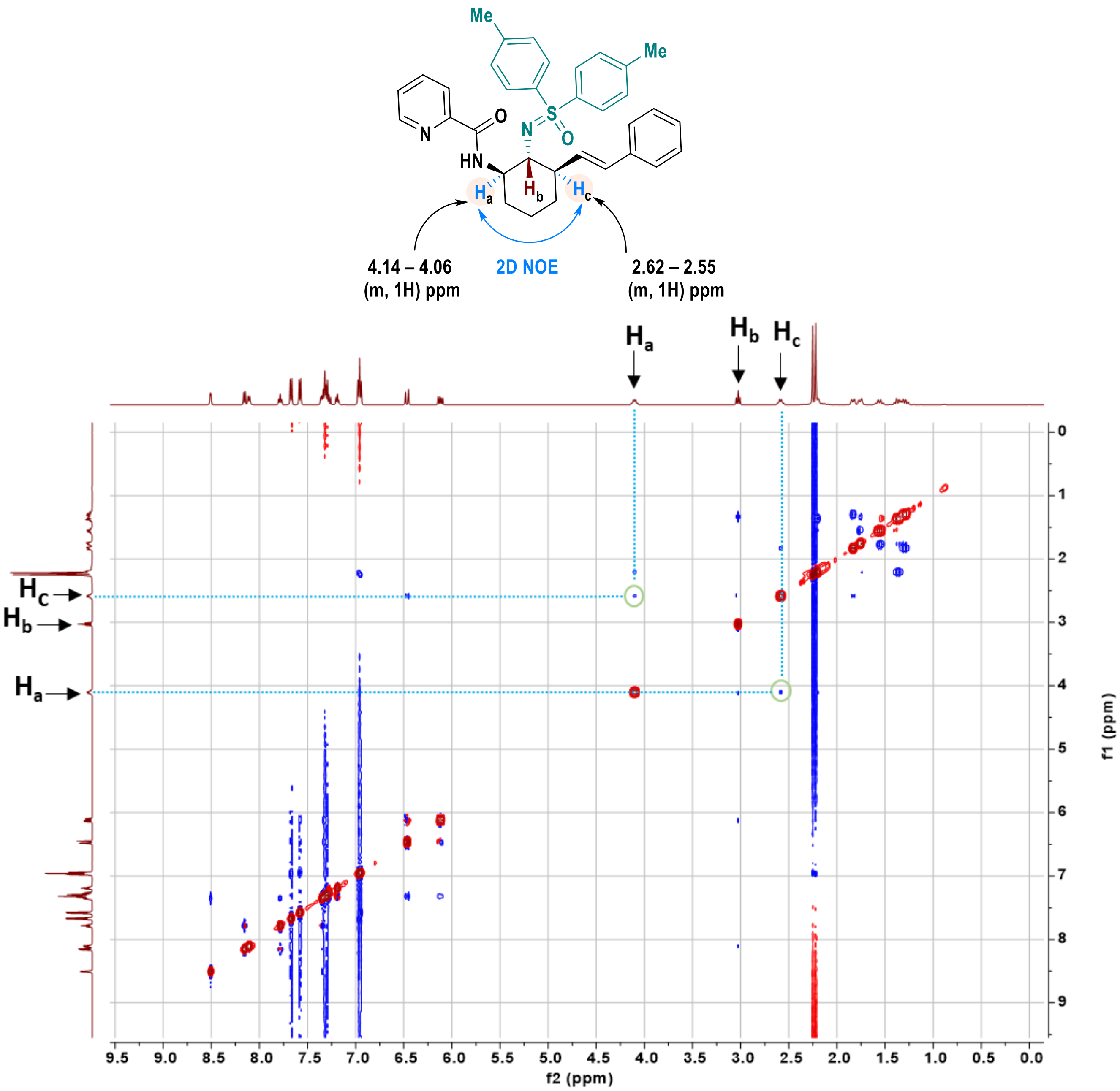
Me
Me
N
O
N
S
O
HN
H_a
H_b
H_c
4.14 – 4.06
(m, 1H) ppm
2D NOE
2.62 – 2.55
(m, 1H) ppm
H_a
H_b
H_c
f2 (ppm)
f1 (ppm)
